# Pathodashboard: Atlas der Epidemiologie für die Tumordiagnostik

**DOI:** 10.1007/s00292-026-01555-w

**Published:** 2026-04-30

**Authors:** Tiemo S. Gerber, Stephanie Strobl, Patrick Focke, Stefan Porubsky, Wilfried Roth, Beate K. Straub

**Affiliations:** 1 Institut für Pathologie, Klinikum Worms gGmbH, Gabriel-von-Seidl-Straße 81, 67550 Worms, Deutschland; 2 Institut für Pathologie, Universitätsmedizin der Johannes Gutenberg-Universität Mainz, Langenbeckstraße 1, 55131 Mainz, Deutschland

**Keywords:** Morphologische Phänotypen, Tumorhäufigkeit, ICD-O-Klassifizierung, Seltene Erkrankungen, Datenvisualisierung, Morphological Phenotypes, Tumour frequency, ICD-O classification, Rare diseases, Data visualisation

## Abstract

**Hintergrund:**

Die Verfügbarkeit vergleichbarer epidemiologischer Daten zu Malignomen ist durch heterogene Klassifikationssysteme und variierende Referenzrahmen erheblich eingeschränkt. Unvollständige Datenerhebungen sowie ein häufig einseitiger Fokus auf spezifische Subtypen beeinträchtigen zudem die Repräsentativität der Ergebnisse. Diese methodischen Diskrepanzen erschweren eine verlässliche quellenübergreifende Analyse von Tumorhäufigkeiten. Ziel dieser Arbeit ist die Auswertung umfangreicher Krebsregisterdaten, um epidemiologische Vergleiche über Lokalisationen hinweg zu ermöglichen und differentialdiagnostische Entscheidungen durch Einbeziehung von Alter, Geschlecht und Liniendifferenzierung zu untermauern.

**Methodik:**

Daten aus dem Programm Surveillance, Epidemiology, and End Results (SEER; 2000–2019, *n* = 12.809.525) und dem Deutschen Zentrum für Krebsregisterdaten (ZfKD; 2000–2019, *n* = 9.754.219) wurden zusammengeführt. Die Klassifizierung der Tumoren erfolgte gemäß ICD-O‑3.2. Unvollständige und unspezifische Datensätze wurden entfernt, und die verbleibenden Daten visualisiert.

**Ergebnisse und Diskussion:**

Die aufbereiteten Datensätze wurden sowohl online als auch offline als Download bereitgestellt und ermöglichen eine aggregierte Übersichtsdarstellung auf Basis gruppierter ICD-O‑3.2‑Codes sowie eine differenzierte Analyse nach morphologischen Phänotypen über 73 anatomische Lokalisationen hinweg. Die resultierenden Darstellungen liefern eine einheitliche epidemiologische Basis für vergleichende Tumoranalysen über ein breites Spektrum von Lokalisationen und Entitäten hinweg.

**Zusatzmaterial online:**

Die Online-Version dieses Artikels (10.1007/s00292-026-01555-w) enthält zwei Reports der Atlas-Version als supplementäre Downloads.

## Abstract (en)

**Background:**

The availability of comparable epidemiological data on malignant tumours remains limited due to heterogeneous classification systems, divergent reference frameworks, and inconsistent case definitions. Incomplete data collection and an often biased focus on specific subtypes also impair the representativeness of the results. These methodological discrepancies make it difficult to perform a reliable cross-source analysis of tumour frequencies. This study aims to evaluate extensive cancer registry data to enable epidemiological comparisons across locations and to support differential diagnostic decisions by including age, gender, and line differentiation.

**Methodology:**

Data from the Surveillance, Epidemiology, and End Results (SEER) program (2000–2019, *n* = 12,809,525) and the German Centre for Cancer Registry Data (ZfKD; 2000–2019, *n* = 9,754,219) were merged. Tumours were classified according to ICD O 3.2. Incomplete and nonspecific datasets were removed and the remaining data visualised.

**Results and discussion:**

The processed datasets were made available both online and as offline downloads, enabling an aggregated overview based on grouped ICD-O‑3.2 codes as well as a differentiated analysis of morphological phenotypes across 73 anatomical sites. The resulting visualisations provide a uniform epidemiological basis for comparative tumour analyses across a broad spectrum of locations and entities.

## Hintergrund

Eine präzise epidemiologische Charakterisierung maligner Tumoren erfordert vollständige, valide und konsistente Daten. Klassische Referenzquellen wie WHO-Klassifikationen oder Lehrbücher stützen sich jedoch häufig auf heterogene Einzelpublikationen, die sich teilweise erheblich hinsichtlich verwendeter Klassifikationssysteme, Vergleichsgruppen und Falldefinitionen unterscheiden. Insbesondere für seltene Tumoren erschweren veraltete Studien, uneinheitliche Tumorbezeichnungen, variierende Einschlusskriterien und eingeschränkte Zugänglichkeit der zugrunde liegenden Daten eine verlässliche Datenanalyse.

Zur Bereitstellung einer breiten Datenbasis für die klinische Anwendung integrierten wir umfangreiche Krebsregisterdaten, die epidemiologische Vergleiche über anatomische Regionen und Tumortypen hinweg ermöglichen. Ziel war die Entwicklung einer Anwendung, die sowohl in der Pathologie und Onkologie als auch für epidemiologisch orientierte Forschungsfragestellungen als strukturiertes Nachschlagewerk dienen kann. Der Fokus liegt dabei auf einer möglichst übersichtlichen Darstellung epidemiologischer Basisdaten von Tumorentitäten in Abhängigkeit von Lokalisation, Geschlecht und Alter.

Die praktische Relevanz dieser Datengrundlage wird im Folgenden exemplarisch am Beispiel der Thymustumoren demonstriert. Dabei werden die sich ergänzenden Ansätze der beiden Veröffentlichungsformate verdeutlicht: Während die statische Atlas-Version sowohl eine aggregierte Übersicht als auch eine detaillierte Aufschlüsselung nach originalen ICD-O‑3.2‑Codes bietet, erlaubt das interaktive Pathodashboard eine dynamische, nutzerdefinierte Datenabfrage. Anhand des Thymus wird aufgezeigt, wie je nach Fragestellung – von der schnellen epidemiologischen Einordnung bis zur differenzierten Analyse seltener Subtypen – beide Darstellungsformen effektiv genutzt werden können. Das interaktive Dashboard finden Sie online verfügbar unter https://mpcdashboard.unimedizin-mainz.de [1], während die Atlas-Version als Zusatzmaterial online als Download zur Verfügung steht.

## Methodik

### Datenquellen

Die Analyse basierte auf zwei bevölkerungsbasierten Krebsregistersystemen, dem Surveillance, Epidemiology, and End Results(SEER)-Programm aus den USA und dem Zentrum für Krebsregisterdaten (ZfKD) aus Deutschland. Insgesamt standen 22.563.744 Datensätze zur Verfügung. SEER-Daten (*n* = 12.809.525) wurden über SEER*Stat Version 8.4.0.1(National Cancer Institute, Bethesda, Maryland, USA) [2] aus der „Incidence – SEER Research Data, 22 Registries, Nov 2021 Sub (2000–2019)“ [3] abgerufen. Die deutschen Daten entstammen den staatlichen Krebsregistern von 2000 bis 2019 [4]. Hämatopoetische Tumoren sind im verfügbaren ZfKD-Datensatz nicht enthalten. Alle Tumoren wurden gemäß ICD-O‑3.2 verschlüsselt. Eingeschlossen wurden ausschließlich maligne Tumoren (Dignitätscode „/3“).

### Datenprozessierung für die statische Atlas-Version

Die Prozessierung der Daten und die statistischen Analysen erfolgten in R (Version 2025.09.2 + 418). Daten aus beiden Quellen wurden mithilfe definierter Mappingtabellen vereinheitlicht. Die Entitäten der ICD-O‑3.2 wurden übergeordneten Gruppen zugeordnet. Beispielsweise wurden die Codes 8070/3 bis 8086/3 als Plattenepithelkarzinome gruppiert. Die Primärtumorlokalisation wurde anhand der Site Recode ICD-O-3/WHO 2008 Definition (inkl. erweiterter Revision 2023) bzw. ergänzend für Knochentumoren anhand der ICD-O‑3.2‑Topographiecodes erfasst. Das Alter wurde in 18 standardisierte 5‑Jahres-Gruppen eingeordnet. Zur Sicherstellung der Datenqualität und -validität wurde eine ergänzende Filterung durchgeführt. Es wurden Fälle ausgeschlossen, wenn essenzielle Variablen fehlten, etwa Alter, Geschlecht, Topographie oder Morphologie. Es wurde eine detaillierte Ausschlussanalyse erstellt, um die Anzahl und Charakteristika der ausgeschlossenen Fälle zu dokumentieren.

Für spezifische anatomische Lokalisationen wurde eine hierarchische Gruppierung implementiert, um verwandte Strukturen zu konsolidieren und zugleich klinisch relevante Unterschiede zu bewahren. Tumoren der Leber und der intrahepatischen Gallengänge wurden zu einer gemeinsamen Kategorie zusammengeführt, ebenso Neoplasien der Niere und des Nierenbeckens sowie verschiedene Hautlokalisationen. Skeletttumoren (ICD-O‑3.2‑Topographiecodes C400–C419) behielten hingegen ihre spezifischen anatomischen Klassifikationen bei; die Codes C408, C409, C418 und C419 wurden als „Knochen“ gruppiert. In die Analyse wurden ausschließlich morphologische Entitäten mit mindestens 20 Fällen einbezogen. Es wurden zwei verschiedene Analyseberichte erstellt.

### Übersichtsbericht und detaillierter Bericht der statischen Atlas-Version

In Anbetracht der Tatsache, dass eine originalgetreue ICD-O‑3.2‑basierte Darstellung nicht für jede Fragestellung sinnvoll ist, stehen zwei komplementäre Versionen zur Verfügung. Dies zeigt sich beispielsweise an den 17 verschiedenen Codiermöglichkeiten der Plattenepithelkarzinome [5], die für Pathologen ohne Zusatznutzen sind. Eine detaillierte Aufschlüsselung der unterschiedlichen Adenokarzinome hingegen kann äußerst hilfreich sein.

Im Übersichtsbericht werden die Fälle nach Primärlokalisation und übergeordneten morphologischen Gruppen stratifiziert, während die detaillierten Berichte eine differenzierte Analyse nach Primärlokalisation, phänotypischer Klassifikation und morphologischem Subtyp ermöglichen. Die phänotypische Einteilung umfasst epitheliale, mesenchymale, neuroektodermale und hämatopoetische Phänotypen sowie eine Sammelgruppe, die Keimzelltumoren ebenso wie gemischte und aus Stammzellen hervorgehende Phänotypen einschließt. Für den detaillierten Bericht wurde ein adaptiver Algorithmus zur Phänotypgruppierung implementiert, der die Visualisierung seltener Tumortypen optimiert und gleichzeitig die Übersichtlichkeit erhält. Hierfür kam ein dreistufiger hierarchischer Ansatz zum Einsatz: Erstens prüfte der Algorithmus, ob Keimzelltumoren, neuroektodermale Tumoren, gemischte oder multipotente Stammzellneoplasien, hämatopoetische sowie mesenchymale Tumoren zu Gruppen zusammengeführt werden konnten (≤ 25 Entitäten). Wurde dieser Schwellenwert überschritten, erfolgte eine zweite Gruppierungsstufe, welche mesenchymale Tumoren ausschloss. Überschritt auch diese Gruppierung weiterhin den Grenzwert, wurde eine dritte Stufe getestet, die ausschließlich Keimzelltumoren, neuroektodermale Tumoren sowie gemischte oder multipotente Stammzellneoplasien zusammenfasste. Lokalisationen, für die alle drei Gruppierungskriterien scheiterten, stellten jeden Phänotyp separat dar.

Die Visualisierungen basieren auf einem mehrteiligen Layout, das morphologische Häufigkeitsverteilungen, geschlechtsspezifische Anteile sowie altersstratifizierte Heatmaps darstellte. Die Kategorien wurden nach Häufigkeit geordnet; pro Seite wurden die 25 häufigsten Entitäten dargestellt. Die prozentualen Anteile einzelner morphologischer Entitäten bezogen sich auf die Gesamtfallzahl vor Anwendung der Mindestfallfilterung, um eine korrekte Abbildung der relativen Häufigkeiten innerhalb der gesamten Kohorte sicherzustellen. Für alle Darstellungen wurde eine farbfehlsichttaugliche Palette verwendet, die aus dem Magma-Farbschema von R abgeleitet ist.

### Datenbankstruktur für das interaktive Pathodashboard

Die Datenbank und der C#-Servercode (Backend) sind in einer virtuellen Maschine (VM) innerhalb der sogenannten demilitarisierten Zone (DMZ) der Universitätsmedizin Mainz platziert. Durch die Verwendung einer VM wird die Datenbank isoliert von anderen virtuellen Maschinen gehalten. Die DMZ ist ein Bereich im Intranet der Universitätsmedizin, der durch eine Firewall vom übrigen Unternehmensnetzwerk abgeschirmt ist. Die Datenbank wird über einen passwortgeschützten SQL-Server betrieben, der als Datenbankmanagementsystem eine sichere Verwaltung strukturierter Daten ermöglicht. Um eine effektive Zugriffskontrolle zu gewährleisten, wurden die für die Anwendung notwendigen Informationen über eine API (Application Programming Interface) der C#-Anwendung bereitgestellt. Eine eindeutige Trennung zwischen der Homepage (Frontend) und dem Backend kann durch Verwendung einer API sichergestellt werden, da diese als Zwischenschicht zwischen beiden fungiert. Die resultierende Abstraktion der Datenbank von der Homepage erhöht die Sicherheit, da das Frontend nicht direkt auf das Backend zugreifen muss. Darüber hinaus übernimmt die API sowohl Authentifizierung als auch Autorisierung, um sicherzustellen, dass der Zugriff nur durch berechtigte Nutzer erfolgt. Sie prüft Anfragen auf ihre Korrektheit und stellt sicher, dass die Daten korrekt formatiert sind, bevor sie an das Backend übermittelt werden. Die Realisierung der Homepage des Pathodashboards erfolgte mit .NET Blazor [6], das als Framework von Microsoft die Erstellung interaktiver Webanwendungen ermöglicht. Durch die Erweiterung um Blazor Hybrid wird zudem die Erstellung übergreifender Anwendungen, beispielsweise für Desktop oder mobile Endgeräte ermöglicht. Zur Vermeidung einer Reidentifikation von Patienten seitens des Anwenders wird die Datenausgabe nur unter bestimmten Einschränkungen ausgegeben. Bei Datenanfragen, die unter fünf Patienten ausgeben, wird, angeglichen an das Vorgehen des RKI, „< 5“ angegeben. Die Struktur der Altersverteilung wird in 5‑Jahres-Schritten ausgegeben.

## Ergebnisse

Insgesamt wurden vom SEER-Programm 12.809.525 und beim ZfKD 9.754.219 Datensätze ausgewertet und zusammengefasst für die Erstellung einer Datenbank verwendet. Anschließend wurden unspezifische Einträge entfernt. Datensätze, in denen die Morphologie mit „Neoplasie“ oder „Karzinom“ codiert war, wurden ausgeschlossen, um eine ausreichende Spezifität zu gewährleisten. Dies betraf 10,92 % der Ausgangsdaten. Anschließend wurden Fälle ausgeschlossen, bei denen wesentliche Variablen fehlten: 0,05 % der Fälle aufgrund einer fehlenden Altersgruppe und 0,22 % aufgrund fehlender morphologischer Angaben. Es wurden insgesamt 2.518.109 Datensätze ausgeschlossen (11,16 % der Ausgangskohorte). Die finale Studienkohorte umfasste 20.045.635 Datensätze (88,84 % der kombinierten Ausgangsdaten) und repräsentiert sämtliche malignen Tumoren, die alle Einschluss- und Qualitätskriterien erfüllten. Diese Kohorte diente als Grundlage für die weiteren statistischen Analysen und Visualisierungen.

**Abb. 1:**
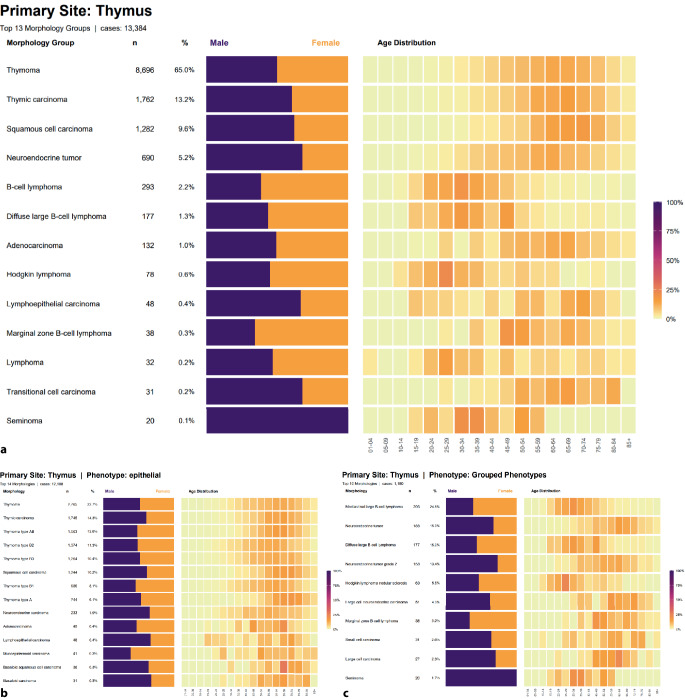
Bevölkerungsbasierte Tumorhäufigkeiten von Thymustumoren in der Atlas-Version. Dargestellt sind absolute Fallzahlen, relative Häufigkeiten (in Prozent), die Geschlechtsverteilung sowie altersspezifische Verteilungsmuster in 5‑Jahres-Intervallen. Die Geschlechtsverteilung wird durch *horizontale* Balkensegmente visualisiert, die Altersverteilung als Heatmap. Im *oberen* Abschnitt der Abbildung ist eine aggregierte Übersicht dargestellt, in der unter anderem verschiedene Thymom-Subtypen, einschließlich Thymom (NOS), zusammengefasst sowie Plattenepithelkarzinome gruppiert dargestellt sind. Der *untere* Abschnitt zeigt die originalen ICD-O‑3.2‑Codes in Textform sortiert nach Liniendifferenzierung (*links* epitheliale Tumoren, *rechts* zusammengefasst alle weiteren Phänotypen). Diese Darstellung ermöglicht eine detaillierte Einordnung der einzelnen Entitäten

Am Beispiel der Thymustumoren lässt sich das Verteilungsmuster verschiedener Tumorentitäten in Abhängigkeit von Geschlecht und morphologischer Differenzierung exemplarisch darstellen (Abb. 1). Die Daten zeigen eine klare Dominanz von Thymomen, die in der aggregierten Darstellung 65 % aller erfassten Fälle ausmachen, gefolgt von Thymuskarzinomen. Innerhalb der detaillierten Morphologieansicht verteilen sich die Thymome auf mehrere Subtypen (Thymom [NOS] und die Typen AB, B2, B3, B1 und A). In der Übersicht werden diese als Thymome aggregiert. Die Gegenüberstellung aggregierter und detaillierter Darstellungen verdeutlicht zugleich methodische Aspekte und Limitationen der Klassifikation. Während die aggregierte Ansicht eine übersichtliche Einordnung der relativen Häufigkeiten erlaubt, werden in der detaillierten Morphologieansicht zahlreiche Entitäten mit geringer Fallzahl sichtbar, darunter neuroendokrine Tumoren, verschiedene Lymphom-Subtypen sowie seltene Keimzelltumoren.

Gleichzeitig offenbaren sich Unsicherheiten der Codierung: So werden plattenepitheliale Karzinome sowohl als eigenständige Entität als auch innerhalb der Gruppe der Thymuskarzinome oder unter anderen Karzinomcodes (z. B. basaloides Plattenepithelkarzinom) geführt, was eine eindeutige Zuordnung erschwert. Auch unspezifische Kategorien wie „Neoplasm, malignant“ oder „Carcinoma, NOS“ weisen auf begrenzte morphologische Differenzierung oder heterogene Verwendung der ICD-O‑3.2‑Codes hin. Die Interpretation der dargestellten Häufigkeiten sollte daher stets im klinisch-pathologischen Kontext erfolgen. Die Visualisierung der bevölkerungsbasierten Verteilungen kann, insbesondere bei unklarer histomorphologischer Differenzierung, dazu beitragen, die Differenzialdiagnose statistisch einzugrenzen und wahrscheinliche Entitäten zu priorisieren – im Sinne des Leitsatzes: „Häufiges ist häufig, Seltenes ist selten“.

**Abb. 2:**
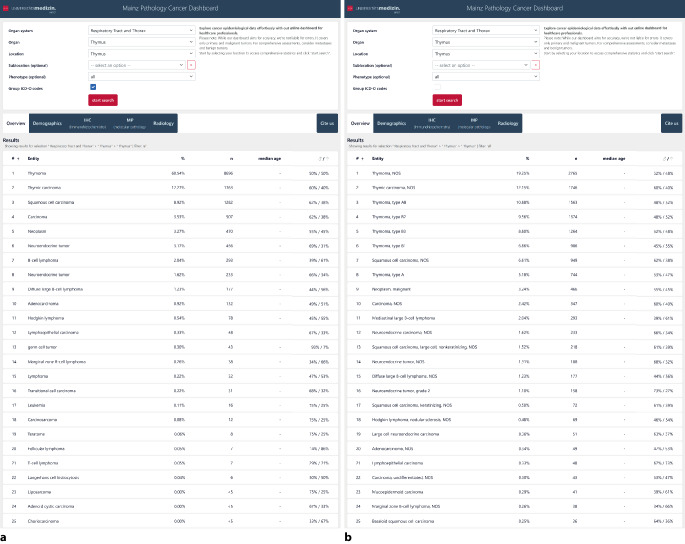
*Visualisierung bevölkerungsbasierter Tumorhäufigkeiten von Thymustumoren im Pathodashboard*. Dargestellt sind absolute und relative Fallzahlen sowie das Medianalter, wobei zwischen aggregierten Tumorgruppen (**a**) und der Darstellung anhand der originalen ICD-O‑3.2‑Codes (**b**) gewechselt werden kann. Alters- und geschlechtsspezifische Verteilungen sind über den Reiter „Demographie“ separat abrufbar. Im Gegensatz zur Atlas-Darstellung können phänotypische Gruppen (z. B. mesenchymal oder hämatopoetisch) hier separat ausgewählt werden, was insbesondere für die Analyse sehr seltener Entitäten von Bedeutung ist

Im direkten Vergleich visualisiert das interaktive Pathodashboard die Daten in vergleichbarer Form (Abb. 2), bietet jedoch entscheidende Vorteile. So erlaubt die Plattform grundsätzlich eine dynamische Aktualisierung, sobald neue Datensätze des ZfKD oder des SEER-Programms verfügbar sind. Darüber hinaus ermöglicht die Plattform die Integration multimodaler Parameter: So lassen sich immunhistochemische und molekularpathologische Profile ebenso implementieren wie radiologische Merkmale (z. B. Kontrastmittelverhalten, Kapselbildung oder Kalzifizierungen). Voraussetzung hierfür ist eine eindeutige Zuordnung dieser Daten mit den entsprechenden ICD-O-Entitäten. Ein weiterer Vorteil ist die webbasierte Verfügbarkeit, die einen ortsunabhängigen Zugriff ohne vorherigen Download ermöglicht.

## Diskussion

Die vorliegende Arbeit bietet eine bevölkerungsbasierte Charakterisierung maligner Tumoren und zeigt, dass Krebsregisterdaten belastbar und standardisiert für morphologische Analysen über zahlreiche anatomische Lokalisationen hinweg nutzbar gemacht werden können. Der integrierte Datensatz vereint zwei Jahrzehnte Krebsregisterdaten von 2000 bis 2019 aus dem SEER-Programm (12.809.525 Fälle) aus den USA und des ZfKD (9.754.219 Fälle) aus Deutschland, entsprechend einem Ausgangsdatensatz von 22.563.744 Malignomen. Diese Kombination zweier großer Gesundheitssysteme erlaubt eine Vergleichbarkeit epidemiologischer Daten in hochentwickelten westlichen Populationen. Die Studie generierte zwei Darstellungen, die unterschiedlichen Verwendungszwecken dienen. Der Übersichtsbericht zeigt insbesondere seltene Tumorentitäten, während in der Detailansicht die Zuordnung zu den ursprünglichen ICD-O‑3.2‑Codes erhalten bleibt.

Die vorliegende Analyse bietet mehrere Vorteile gegenüber den Angaben der WHO-Tumorserie oder fachspezifischer Lehrwerke, die häufig auf Metaanalysen heterogener Fallserien beruhen. Diese unterscheiden sich unter anderem hinsichtlich Alterskategorisierung, Klassifikationsschemata und Studiendesign. So geht aus der WHO-Klassifikation der Tumoren des Thymus hervor, dass die relative Häufigkeit einzelner histologischer Subtypen aufgrund der Vielzahl zitierter Studien erheblich variiert. Exemplarisch zeigen Thymome des Typs B1 Anteile zwischen 5,9 % und 52,8 % aller Thymome, was die eingeschränkte Vergleichbarkeit aggregierter Literaturangaben verdeutlicht [7]. Solcherart relative und zum Teil kontextabhängige Angaben erschweren valide tumorübergreifende Vergleiche und führen insbesondere bei seltenen Tumorarten häufig zu verzerrten oder nur eingeschränkt generalisierbaren Ergebnissen. Dies wird dadurch verstärkt, dass epidemiologische Studien zu seltenen Tumoren aufgrund ihrer geringen Inzidenz oftmals keine ausreichend großen Kohorten generieren können [8]. Hierzu muss man einschränkend sagen, dass der Begriff des seltenen Tumors dabei selbst potenziell missverständlich ist: Trotz niedriger Inzidenzraten entfallen insgesamt etwa 22 % aller Krebsdiagnosen in diese Kategorie. Innerhalb dieser Gruppe weisen 30 % der Fälle eine äußerst geringe Inzidenz von < 1/100.000 auf [9]. Diese ausgeprägte Heterogenität erschwert sowohl die diagnostische Einordnung als auch die klinische Entscheidungsfindung. Eine präzisere Klassifikation nach relativer Häufigkeit könnte die klinische Bewertung unterstützen. Der Ansatz der vorliegenden Studie adressiert diese Limitationen durch die einheitliche Anwendung der ICD-O‑3.2‑Morphologiecodierung und des strukturierten Ausgabeformates mit einheitlichen Altersgruppen und Geschlechterangaben. Dadurch wird es möglich, demografische und morphologische Muster systematisch und direkt über verschiedene anatomische Lokalisationen hinweg zu vergleichen.

## Limitationen

Als Sekundärdatenanalyse ist diese Studie grundsätzlich auf die Qualität und Vollständigkeit der Primärdokumentation angewiesen und unterliegt deren potenziellen Codier- und Erfassungsbias. Eine wesentliche Einschränkung ergibt sich aus der fehlenden Erfassung hämatopoetischer Malignome innerhalb der ZfKD-Daten. Diese Neoplasien – darunter Leukämien, Hodgkin- und Non-Hodgkin-Lymphome sowie Plasmazellneoplasien – machen in Deutschland rund 7,92 % aller Krebsdiagnosen aus [10]. Ihr Ausschluss führt zwangsläufig zu einer Überrepräsentation solider Tumoren im deutschen Teil der Kohorte und limitiert die Vergleichbarkeit bestimmter morphologischer Muster zwischen den beiden Gesundheitssystemen. Methodisch bedingt resultiert dies entsprechend in einer Unterschätzung hämatopoetischer Entitäten in der Gesamtauswertung. Darüber hinaus sind einzelne Tumoren in den Registerdaten lediglich als unspezifiziertes Karzinom oder als Neoplasie ohne differenzierte histologische Subtypisierung codiert, was zu einer gewissen diagnostischen Unschärfe in der morphologischen Zuordnung führt. Zudem ist zu berücksichtigen, dass weder Metastasen noch nichtmelanozytäre Hauttumoren gesondert erfasst wurden, letztere mit Ausnahme prognostisch ungünstiger Stadien, was aus einer fehlenden Meldepflicht in Deutschland und den USA beruht. Auf methodischer Ebene geht die Gruppierung morphologischer Codes mit einem unvermeidbaren Informationsverlust einher und basiert auf zum Teil subjektiven Bewertungen. Trotz definierter Kriterien beruhen einzelne Zuordnungen auf konzeptionellen Entscheidungen, sodass alternative Klassifikationsansätze in Teilen vertretbar gewesen wären. In der Folge könnten biologisch heterogene Subtypen zusammengefasst worden sein, die aus klinischer oder molekularer Perspektive differenzierter zu betrachten wären. Zur Sicherstellung größtmöglicher Transparenz wurden sämtliche verwendeten Mappingtabellen öffentlich zugänglich gemacht. Zudem können systembedingte Unterschiede in Datenstruktur, Variablendefinition und technischer Umsetzung zwischen den zugrunde liegenden Registern sowie den daraus generierten Ausgabeformaten (Dashboard und Atlas) zu geringfügigen Abweichungen in aggregierten Darstellungen führen. In der Interpretation der Ergebnisse ist zu berücksichtigen, dass diese primär deskriptiver Natur sind und die bereitgestellte Visualisierung einer explorativen, quellenübergreifenden Darstellung der Verteilung bestimmter Entitäten ermöglichen, jedoch keine populationsbezogene Inzidenzanalyse mit standardisierter Altersadjustierung ersetzen. Ungeachtet dieser Einschränkungen bleiben die Daten für den intendierten Zweck – die standardisierte morphologische Charakterisierung maligner Tumoren über anatomische Lokalisationen hinweg – uneingeschränkt nutzbar, da die analytische Methodik einheitlich auf alle eingeschlossenen Tumorarten angewendet wurde. Die zentrale Stärke der Studie liegt in der Kombination dreier Komponenten: einer außergewöhnlich großen Fallzahl, einer definierten Methodik und der Integration zweier unabhängiger, bevölkerungsbasierter Krebsregister. Diese Konstellation reduziert Selektionsverzerrungen, erhöht die statistische Verlässlichkeit, insbesondere für seltene Entitäten, und ermöglicht eine epidemiologische Vergleichbarkeit, die in dieser Form bislang nur eingeschränkt verfügbar war.

## Ausblick

Die vorliegenden Ergebnisse verdeutlichen, dass bevölkerungsbasierte Krebsregisterdaten eine robuste Grundlage für die standardisierte Beschreibung morphologischer Muster über zahlreiche anatomische Lokalisationen hinweg bieten. Die Integration von epidemiologischen Daten und Morphologie kann die diagnostische Tätigkeit im Alltag erleichtern und zu einer schnelleren und präziseren Diagnostik beitragen. Weitere Analysen sind derzeit in Arbeit, die zusätzliche Daten zur Immunhistochemie und Molekularpathologie im Pathodashboard integrieren, sowie eine Auswertung einer Umfrage unter Pathologen mit anwendungsbasierten Beispielen.

## Fazit für die Praxis

- Standardisierte Krebsregisterdaten ermöglichen eine Einordnung der Häufigkeiten und Alters- und Geschlechterverteilung maligner Tumoren über anatomische Lokalisationen hinweg.
- Seltene Tumorentitäten lassen sich durch die hohe Fallzahl zuverlässiger abbilden als in vielen traditionellen Fallserien.
- Die Ergebnisse können als praktisches Nachschlagewerk für Pathologie, Onkologie und Epidemiologie dienen, insbesondere bei der Beurteilung ungewöhnlicher oder seltener Tumorformen.

## Supplementary Information

ESM1: Bericht 1: Primärtumoranalyse (gruppierte Morphologie)

ESM2: Bericht 2: Primärtumor- und Phänotypenanalyse (detaillierte Morphologiecodierung)

## Data Availability

Die in dieser Studie analysierten epidemiologischen Primärdatensätze wurden aus dem SEER-Programm und vom ZfKD bezogen. Aufgrund strenger datenschutzrechtlicher Bestimmungen und der Nutzungsvereinbarungen mit den Registern können die individuellen Rohdaten nicht öffentlich zugänglich gemacht oder durch die Autoren weitergegeben werden. Sämtliche aggregierte und visualisierte Daten, die den Ergebnissen dieser Studie zugrunde liegen, sind als Supplement in dieser Veröffentlichung enthalten und frei verfügbar. Der zur Analyse verwendete R‑Code sowie die hierfür erforderlichen Mappingdateien sind im GitHub-Repository unter https://github.com/Pathoatlas/Pathoatlas frei zugänglich.
