# Supplementary material for: Pathodashboard: an epidemiological atlas for tumour diagnostics. German version
Source: Pathologie (Heidelb). 2026 Apr 30;47(4):287–94. [Article in German] doi: 10.1007/s00292-026-01555-w (PMC13309514; doi:10.1007/s00292-026-01555-w)

Report 1: Primary Site Analysis (Morphology Groups)

Table of Contents

Adnexa Other and Genital Female Other 4
Adrenal Gland 5
Ampulla of Vater 6
Appendix 7
Biliary Other 8
Bones 9
Bones of skull and face and associated joints 10
Brain 11
Breast 12
Buccal Mucosa 13
CNS Other 14
Cervix uteri 15
Colon and Rectum (excluding Appendix) 16
Corpus uteri 17
Digestive Other 18
Endocrine Other 19
Epididymis 20
Esophagus 21
Extrahepatic Bile Ducts 22
Eye and Orbit 23
Fallopian Tube 24
Floor of Mouth 25
Gallbladder 26
Gum 27
Heart 28
Hematopoetic and reticuloendothelial 29
Hypopharynx 30
Intracranial Gland 31
Kidney and Renal Pelvis 32
Larynx 33
Lesions of anus and anal canal 34
Lip 35
Liver and Intrahepatic Bile Ducts 36
Long bones of lower limb and associated joints 37
Long bones of upper limb and scapula and associated joints 38

Report 1: Primary Site Analysis (Morphology Groups)

Table of Contents

Lung and Bronchus 39
Major Salivary Glands 40
Mandible 41
Meninges 42
Mouth Other 43
Nasal Cavity and Paranasal Sinuses 44
Nasopharynx 45
Oropharynx 46
Ovary 47
Palate excluding Soft and Uvula 48
Pancreas 49
Parathyroid gland 50
Pelvic bones and sacrum and coccyx and associated joints a 51
Penis 52
Peripheral nerves and autonomic nervous system 53
Pharynx and Oral Cavity Other 54
Placenta 55
Prostate 56
Retroperitoneum and Peritoneum 57
Rib and sternum and clavicle and associated joints 58
Short bones of lower limb and associated joints 59
Short bones of upper limb and associated joints 60
Sinus Other 61
Skin 62
Small intestine 63
Stomach 64
Testis 65
Thymus 66
Thyroid 67
Tongue 68
Trachea 69
Ureter 70
Urethra 71
Urinary Bladder 72
Urinary Other 73

Report 1: Primary Site Analysis (Morphology Groups)

Table of Contents

Vagina 74
Vertebral column 75
Vulva 76

Primary Site: Adnexa Other and Genital Female Other

Top 13 Morphology Groups | cases: 6,305

Morphology Group	n	%
Adenocarcinoma	4,621	73.3%
Squamous cell carcinoma	562	8.9%
Leiomyosarcoma	208	3.3%
Mullerian mixed tumor	205	3.3%
Carcinosarcoma	199	3.2%
Neuroendocrine tumor	89	1.4%
Sarcoma	56	0.9%
Diffuse large B-cell lymphoma	43	0.7%
germ cell tumor	34	0.5%
Choriocarcinoma	32	0.5%
Mesodermal mixed tumor	27	0.4%
Mesonephroma	24	0.4%
Adenosarcoma	23	0.4%

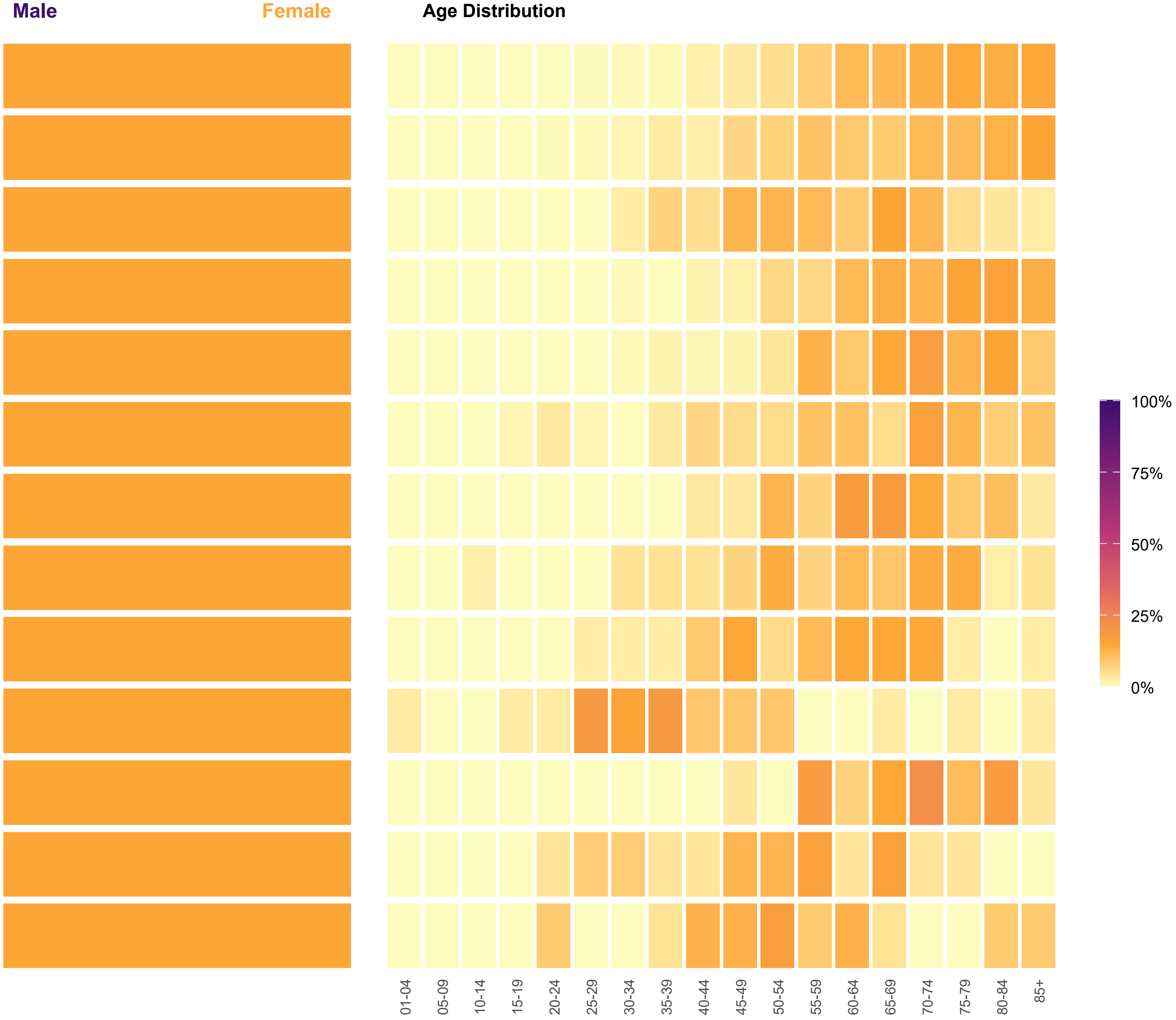

Primary Site: Adrenal Gland

Top 13 Morphology Groups | cases: 10,717

Morphology Group	n	%
Adrenal cortical carcinoma	4,687	43.7%
Neuroblastoma	2,527	23.6%
Pheochromocytoma	1,738	16.2%
Diffuse large B-cell lymphoma	342	3.2%
Adenocarcinoma	332	3.1%
Ganglioneuroblastoma	327	3.1%
Neuroendocrine tumor	171	1.6%
Leiomyosarcoma	121	1.1%
Hemangiosarcoma	89	0.8%
Sarcoma	64	0.6%
Paraganglioma	52	0.5%
Lymphoma	51	0.5%
Liposarcoma	22	0.2%

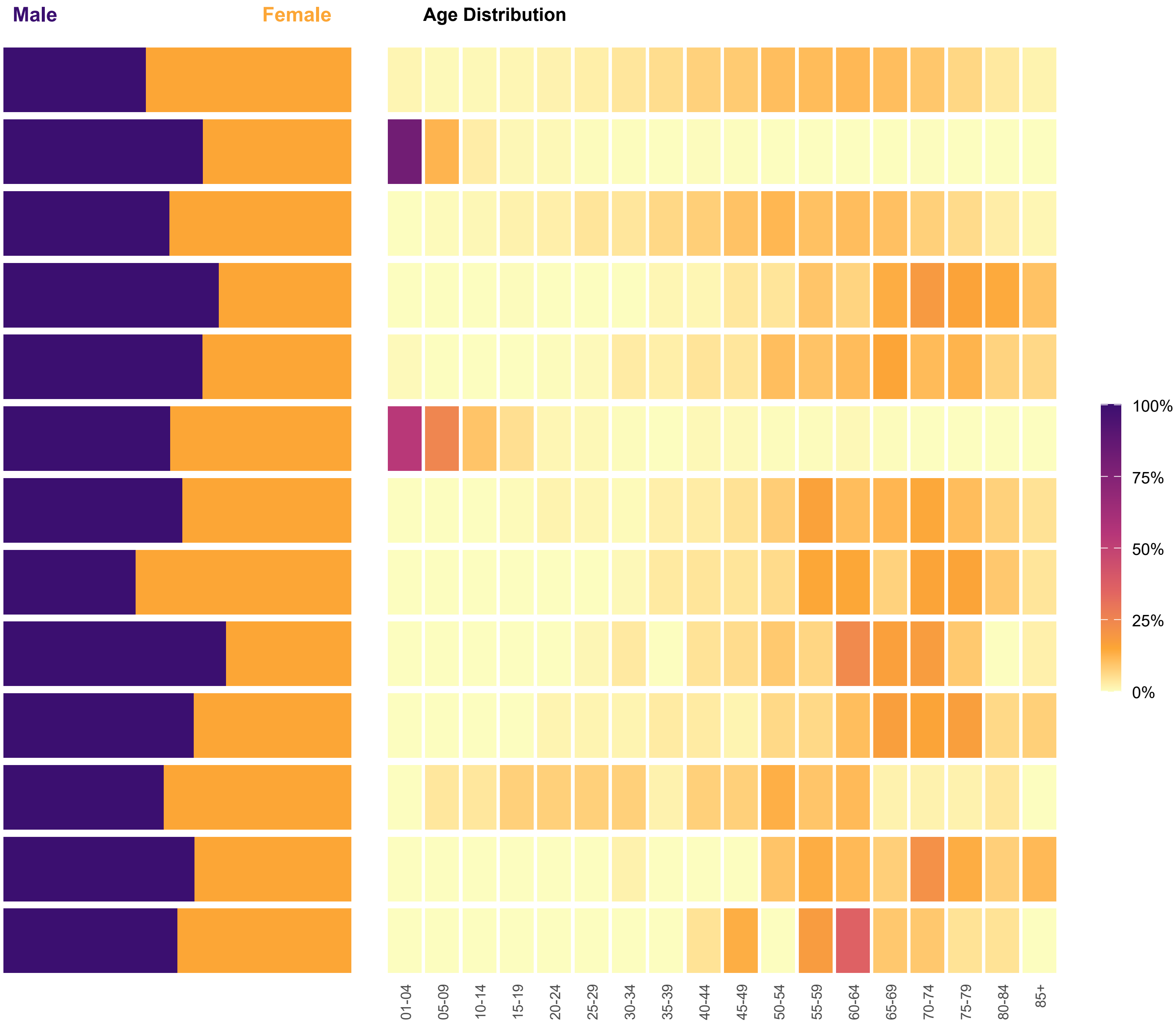

Primary Site: Ampulla of Vater

Top 5 Morphology Groups | cases: 27,826

Morphology Group	n	%
Adenocarcinoma	26,475	95.1%
Neuroendocrine tumor	1,210	4.3%
Squamous cell carcinoma	30	0.1%
Diffuse large B-cell lymphoma	26	<0.1%
Follicular lymphoma	21	<0.1%

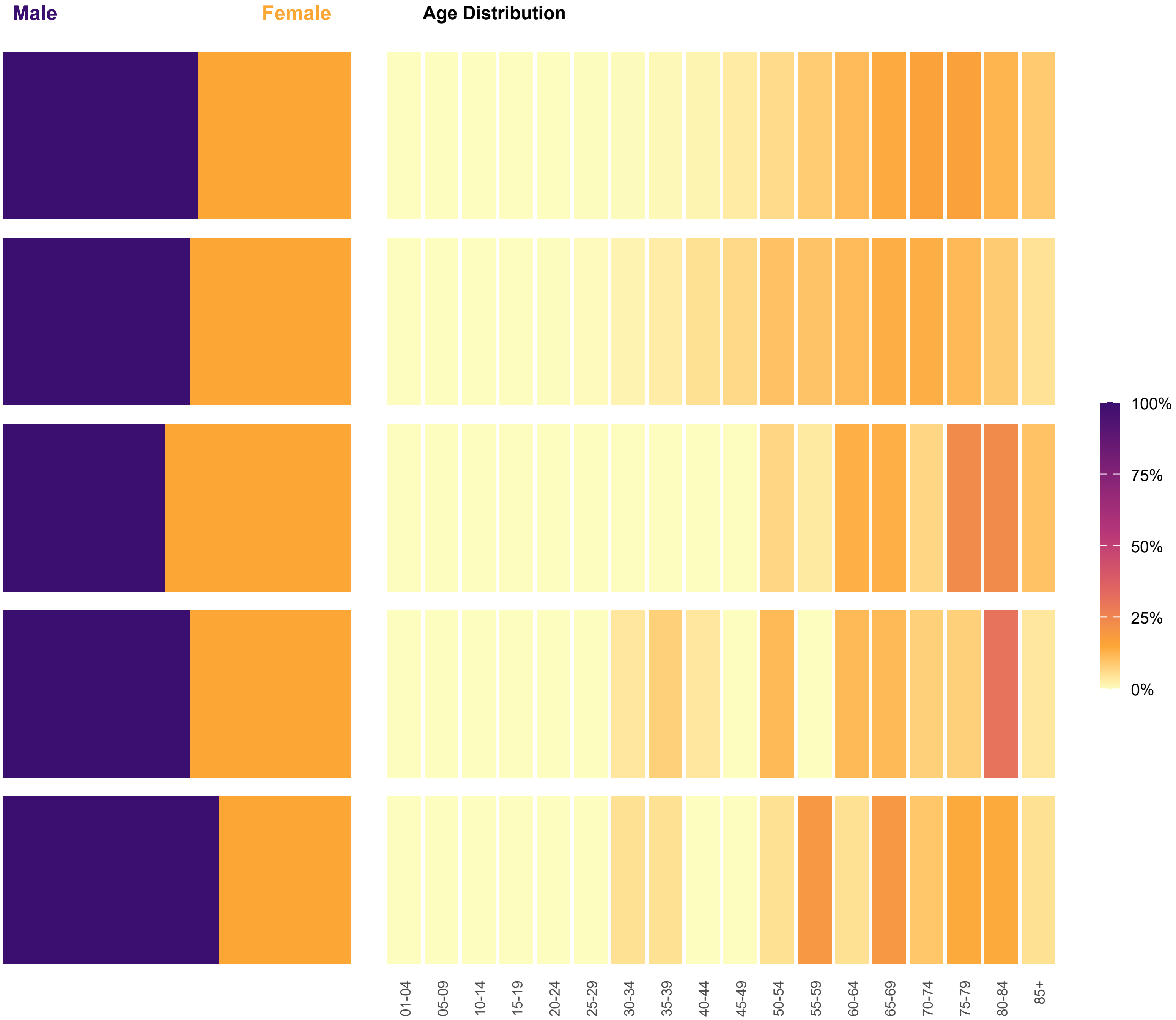

Primary Site: Appendix

Top 9 Morphology Groups | cases: 41,406

Morphology Group	n	%
Adenocarcinoma	20,478	49.5%
Neuroendocrine tumor	16,619	40.1%
Goblet cell carcinoid	3,934	9.5%
Diffuse large B-cell lymphoma	120	0.3%
Burkitt lymphoma	56	0.1%
Gastrointestinal stromal tumor	43	0.1%
Follicular lymphoma	37	<0.1%
Marginal zone B-cell lymphoma	25	<0.1%
Mantle cell lymphoma	21	<0.1%

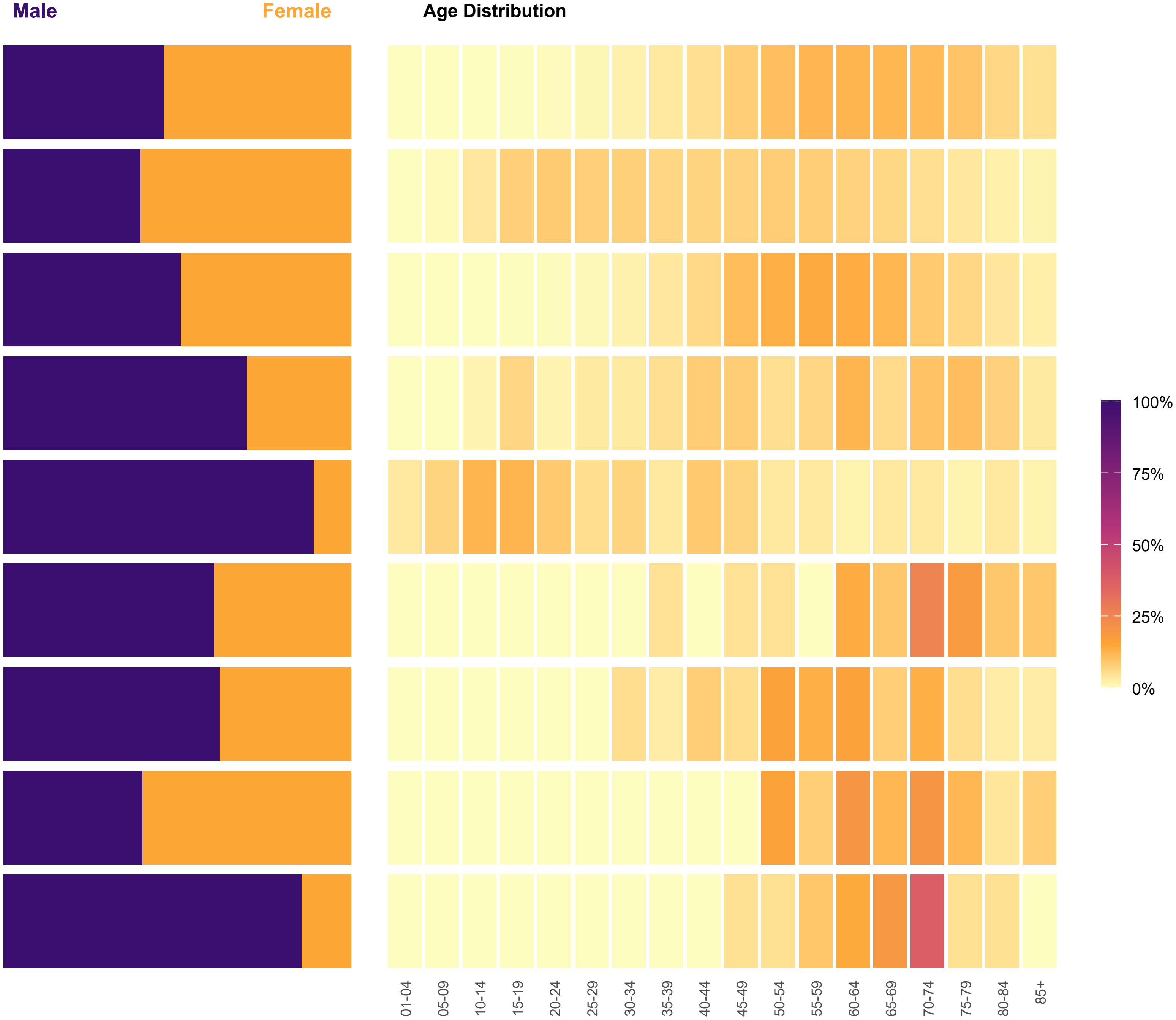

Primary Site: Biliary Other

Top 4 Morphology Groups | cases: 10,069

Morphology Group	n	%
Adenocarcinoma	9,889	98.2%
Neuroendocrine tumor	90	0.9%
Squamous cell carcinoma	36	0.4%
Combined hepatocellular carcinoma…	28	0.3%

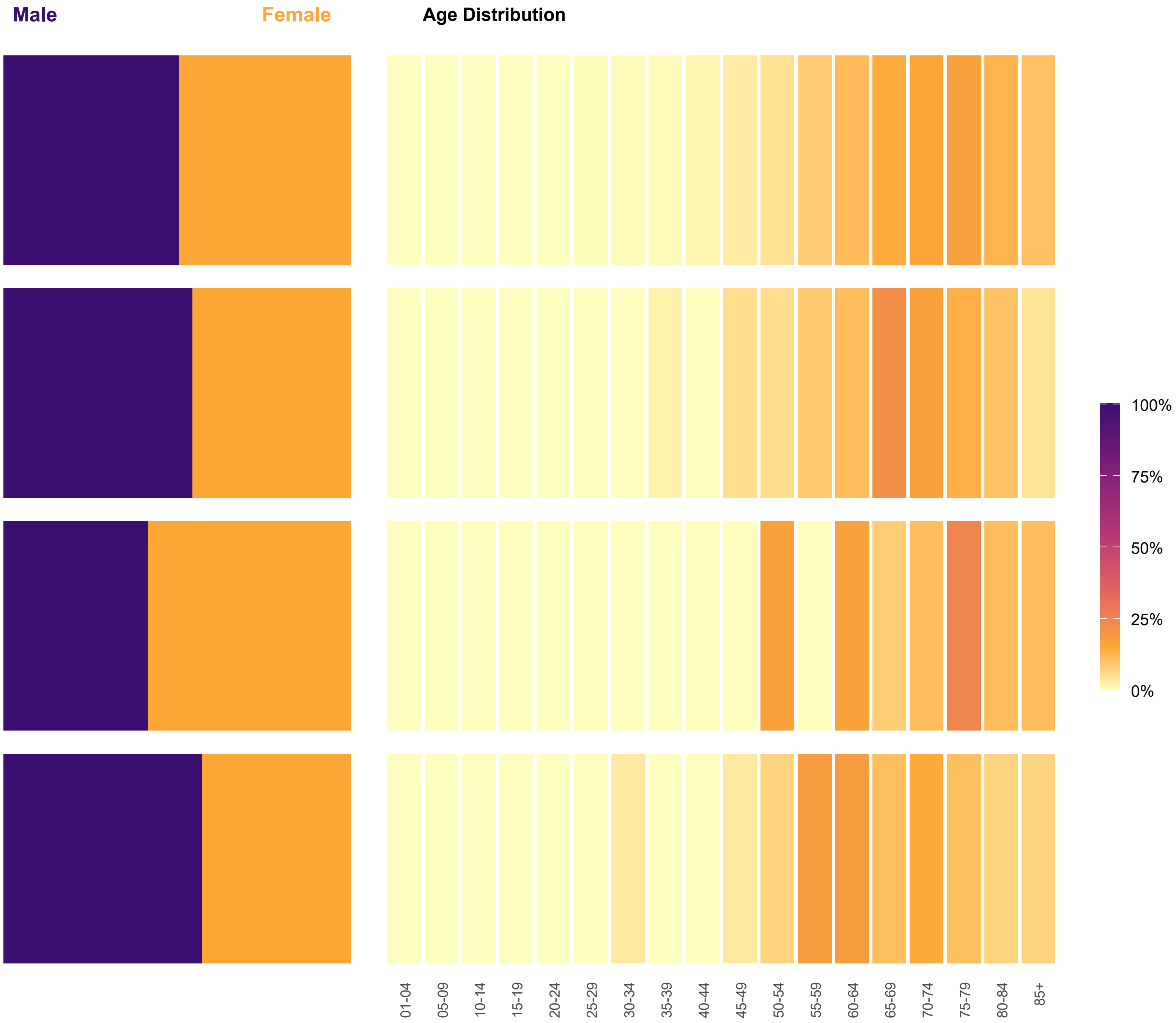

Primary Site: Bones

Top 12 Morphology Groups | cases: 3,100

Morphology Group	n	%
Osteosarcoma	683	22.0%
Chondrosarcoma	638	20.6%
Plasmacytoma	607	19.6%
Diffuse large B-cell lymphoma	321	10.4%
Langerhans cell histiocytosis	188	6.1%
Sarcoma	125	4.0%
Chordoma	70	2.3%
Lymphoma	68	2.2%
Myxoid chondrosarcoma	63	2.0%
Follicular lymphoma	47	1.5%
malignant giant cell tumor of bone	44	1.4%
Hemangiosarcoma	27	0.9%

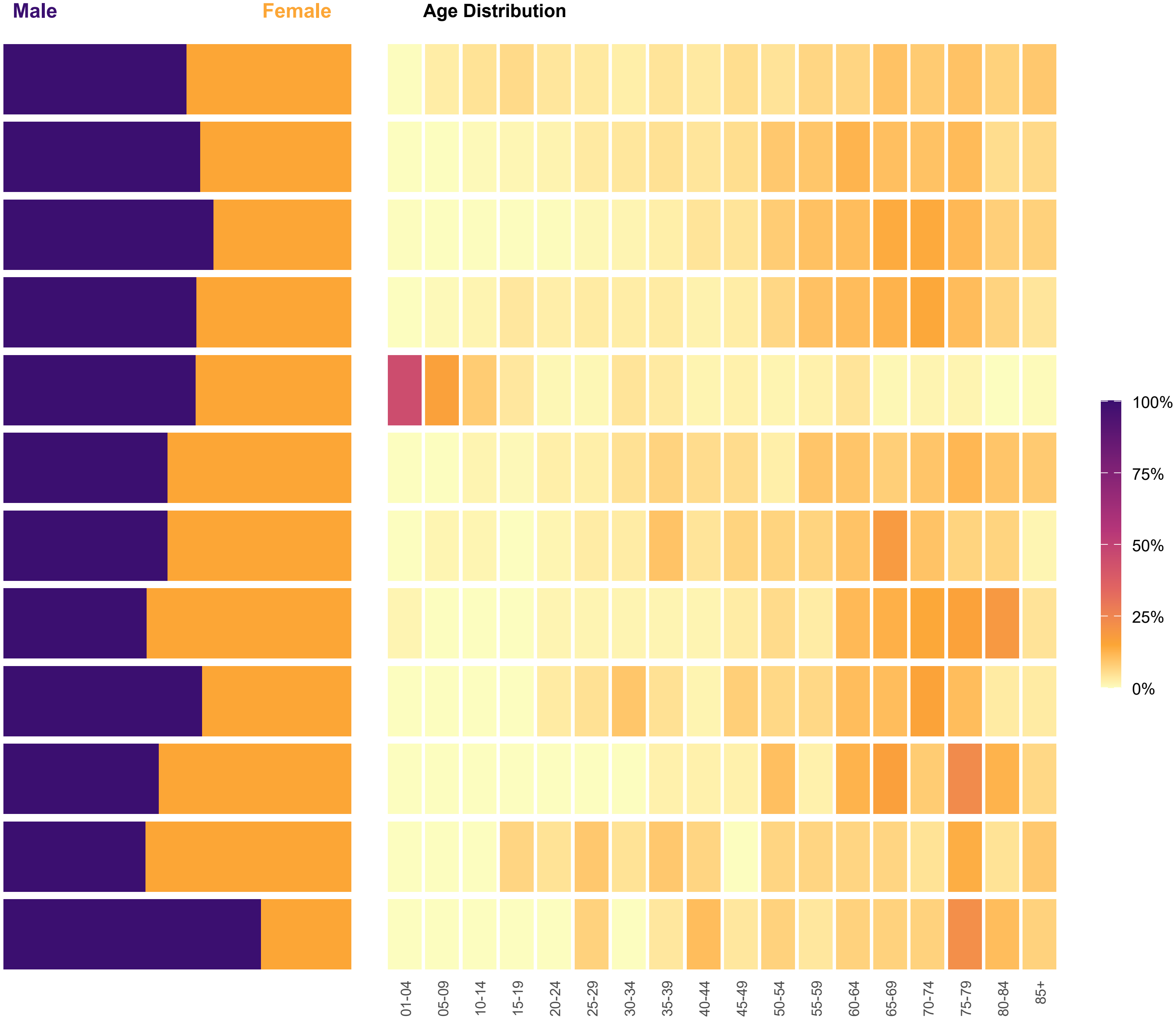

Primary Site: Bones of skull and face and associated joints

Top 16 Morphology Groups | cases: 4,846

Morphology Group	n	%
Chordoma	1,242	25.6%
Chondrosarcoma	792	16.3%
Osteosarcoma	678	14.0%
Langerhans cell histiocytosis	671	13.8%
Plasmacytoma	445	9.2%
Diffuse large B-cell lymphoma	199	4.1%
Sarcoma	124	2.6%
Ameloblastoma	106	2.2%
Myxoid chondrosarcoma	98	2.0%
Squamous cell carcinoma	84	1.7%
Odontogenic tumor	80	1.7%
Lymphoma	42	0.9%
Ewing sarcoma	26	0.5%
Follicular lymphoma	26	0.5%
malignant giant cell tumor of bone	21	0.4%
Fibrosarcoma	20	0.4%

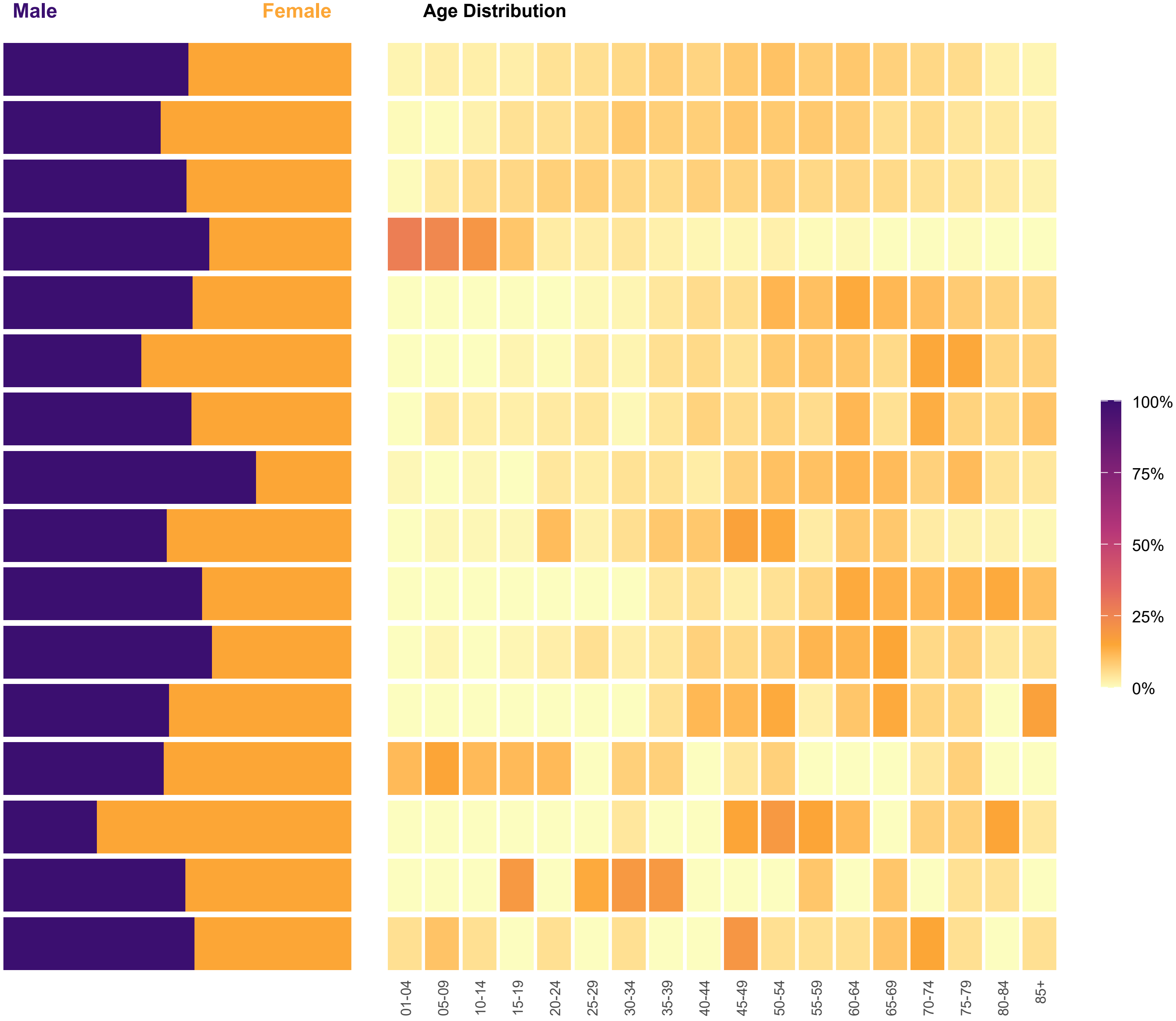

Primary Site: Brain

Top 25 Morphology Groups | cases: 257,541

Morphology Group	n	%
Glioblastoma	155,645	60.4%
Astrocytoma	40,446	15.7%
Glioma	14,402	5.6%
Oligodendroglioma	13,995	5.4%
Diffuse large B-cell lymphoma	7,889	3.1%
Oligoastrocytoma	5,920	2.3%
Medulloblastoma	5,522	2.1%
Ependymoma	5,502	2.1%
Lymphoma	2,127	0.8%
CNS embryonal tumor	1,169	0.5%
Germinoma	849	0.3%
Rhabdoid tumor	577	0.2%
Ganglioglioma	382	0.1%
Meningioma	275	0.1%
Choroid plexus carcinoma	241	<0.1%
Chordoma	213	<0.1%
Sarcoma	165	<0.1%
Neuroblastoma	156	<0.1%
Astroblastoma	141	<0.1%
Marginal zone B-cell lymphoma	133	<0.1%
Malignant peripheral nerve sheath…	131	<0.1%
Mixed germ cell tumor	123	<0.1%
T-cell lymphoma	105	<0.1%
Solitary fibrous tumor	89	<0.1%
Neuroepithelioma	89	<0.1%

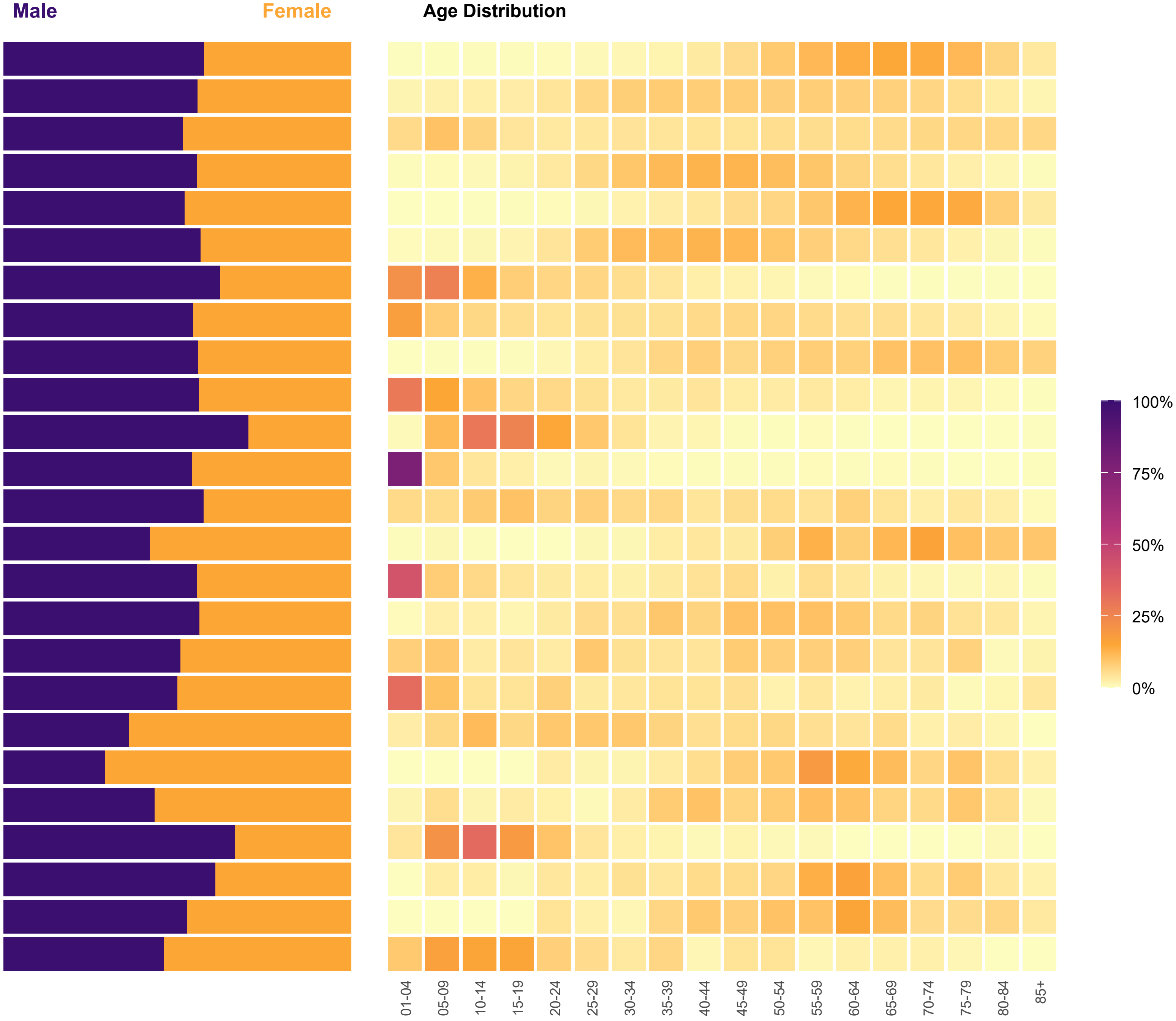

Primary Site: Breast

Top 25 Morphology Groups | cases: 2,890,779

Morphology Group	n	%
Adenocarcinoma	2,870,046	99.3%
Phyllodes tumor	5,116	0.2%
Adenoid cystic carcinoma	2,200	<0.1%
Neuroendocrine tumor	2,161	<0.1%
Hemangiosarcoma	1,863	<0.1%
Squamous cell carcinoma	1,655	<0.1%
Diffuse large B-cell lymphoma	1,586	<0.1%
Marginal zone B-cell lymphoma	1,123	<0.1%
Sarcoma	1,045	<0.1%
Follicular lymphoma	693	<0.1%
Carcinosarcoma	563	<0.1%
Lymphoma	421	<0.1%
Myoepithelial carcinoma	348	<0.1%
Leiomyosarcoma	224	<0.1%
Anaplastic large cell lymphoma	147	<0.1%
Liposarcoma	131	<0.1%
Chronic lymphocytic leukemia	103	<0.1%
Fibrosarcoma	103	<0.1%
Medullary thyroid carcinoma	96	<0.1%
Osteosarcoma	93	<0.1%
lymphoma non-Hodgkin	90	<0.1%
T-cell lymphoma	82	<0.1%
Transitional cell carcinoma	60	<0.1%
Mantle cell lymphoma	59	<0.1%
Burkitt lymphoma	58	<0.1%

Male Female

Age Distribution

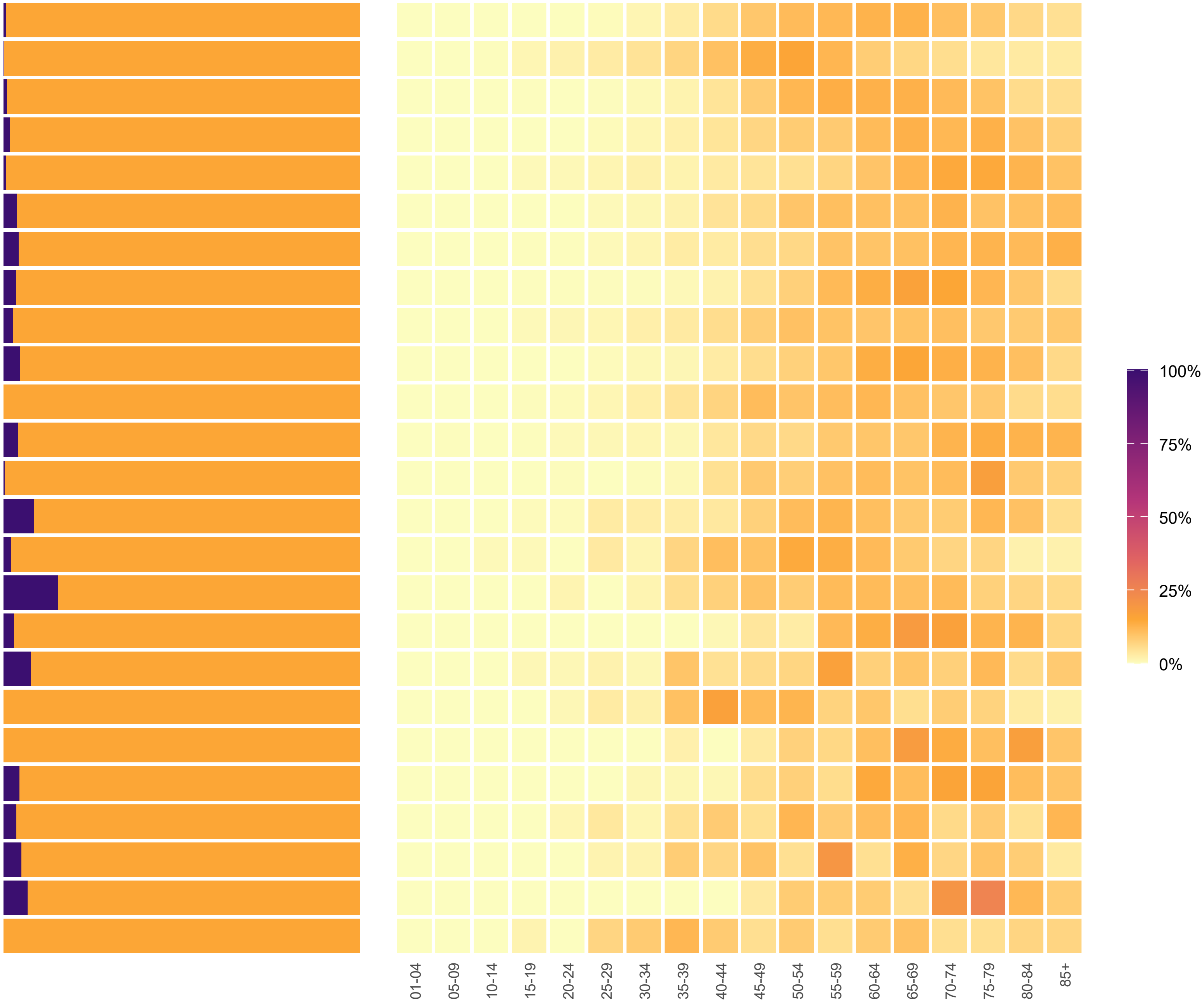

Primary Site: Buccal Mucosa

Top 8 Morphology Groups | cases: 12,045

Morphology Group	n	%
Squamous cell carcinoma	10,042	83.4%
Adenocarcinoma	1,025	8.5%
Adenoid cystic carcinoma	331	2.7%
Diffuse large B-cell lymphoma	135	1.1%
Marginal zone B-cell lymphoma	98	0.8%
Follicular lymphoma	78	0.6%
Melanoma	75	0.6%
Lymphoma	42	0.3%

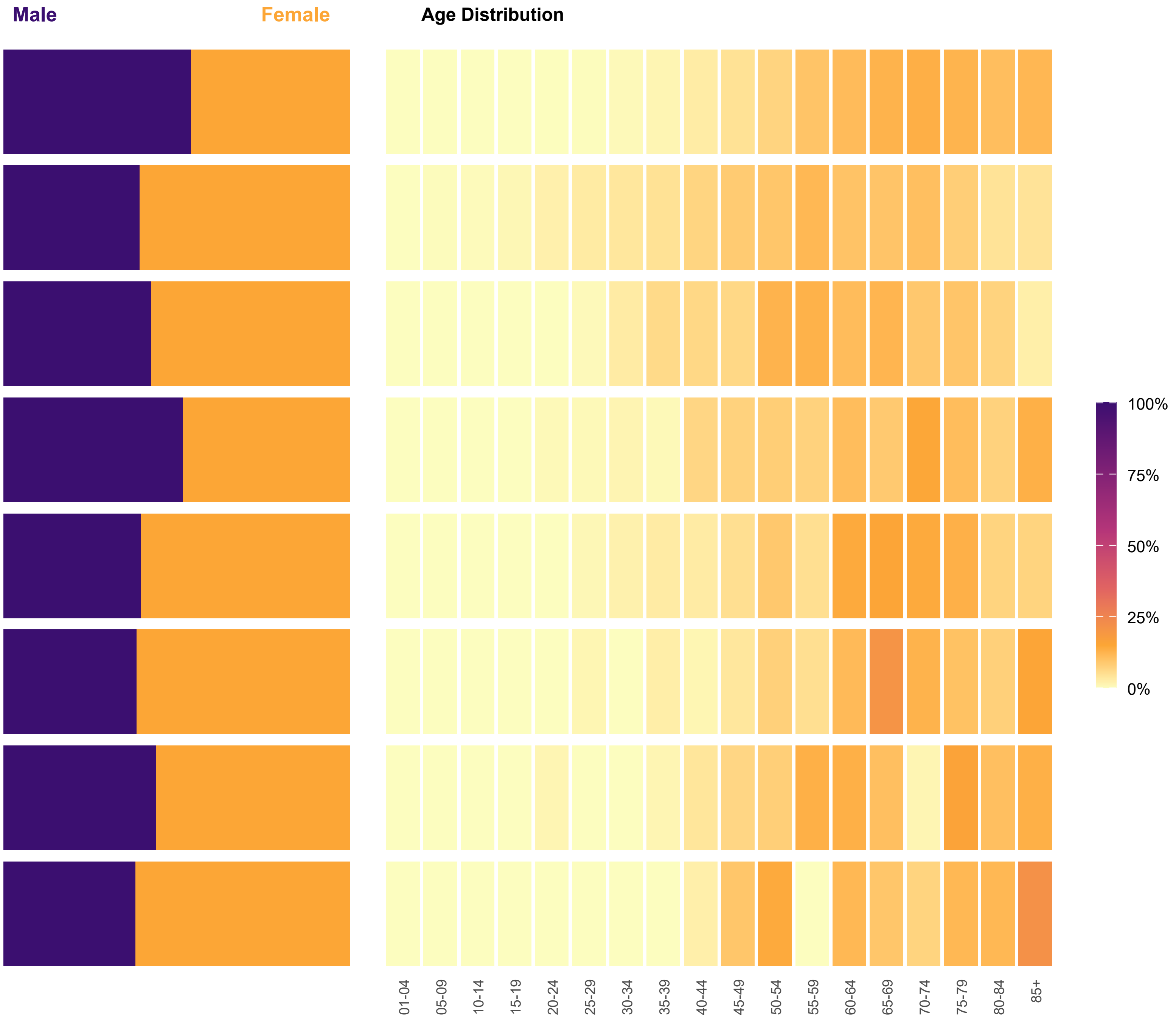

Primary Site: CNS Other

Top 25 Morphology Groups | cases: 11,414

Morphology Group	n	%
Ependymoma	4,489	39.3%
Glioma	1,891	16.6%
Diffuse large B-cell lymphoma	1,221	10.7%
Astrocytoma	887	7.8%
Lymphoma	590	5.2%
Malignant peripheral nerve sheath…	575	5.0%
Glioblastoma	372	3.3%
Chordoma	178	1.6%
Follicular lymphoma	162	1.4%
CNS embryonal tumor	135	1.2%
Neuroblastoma	126	1.1%
Plasmacytoma	55	0.5%
Burkitt lymphoma	53	0.5%
Marginal zone B-cell lymphoma	51	0.4%
Rhabdoid tumor	49	0.4%
Germinoma	49	0.4%
Melanoma	36	0.3%
Sarcoma	36	0.3%
Oligoastrocytoma	32	0.3%
Oligodendroglioma	29	0.3%
Ganglioneuroblastoma	29	0.3%
Myeloid sarcoma	25	0.2%
Olfactory neurogenic tumor	25	0.2%
Paraganglioma	23	0.2%
Leukemia	21	0.2%

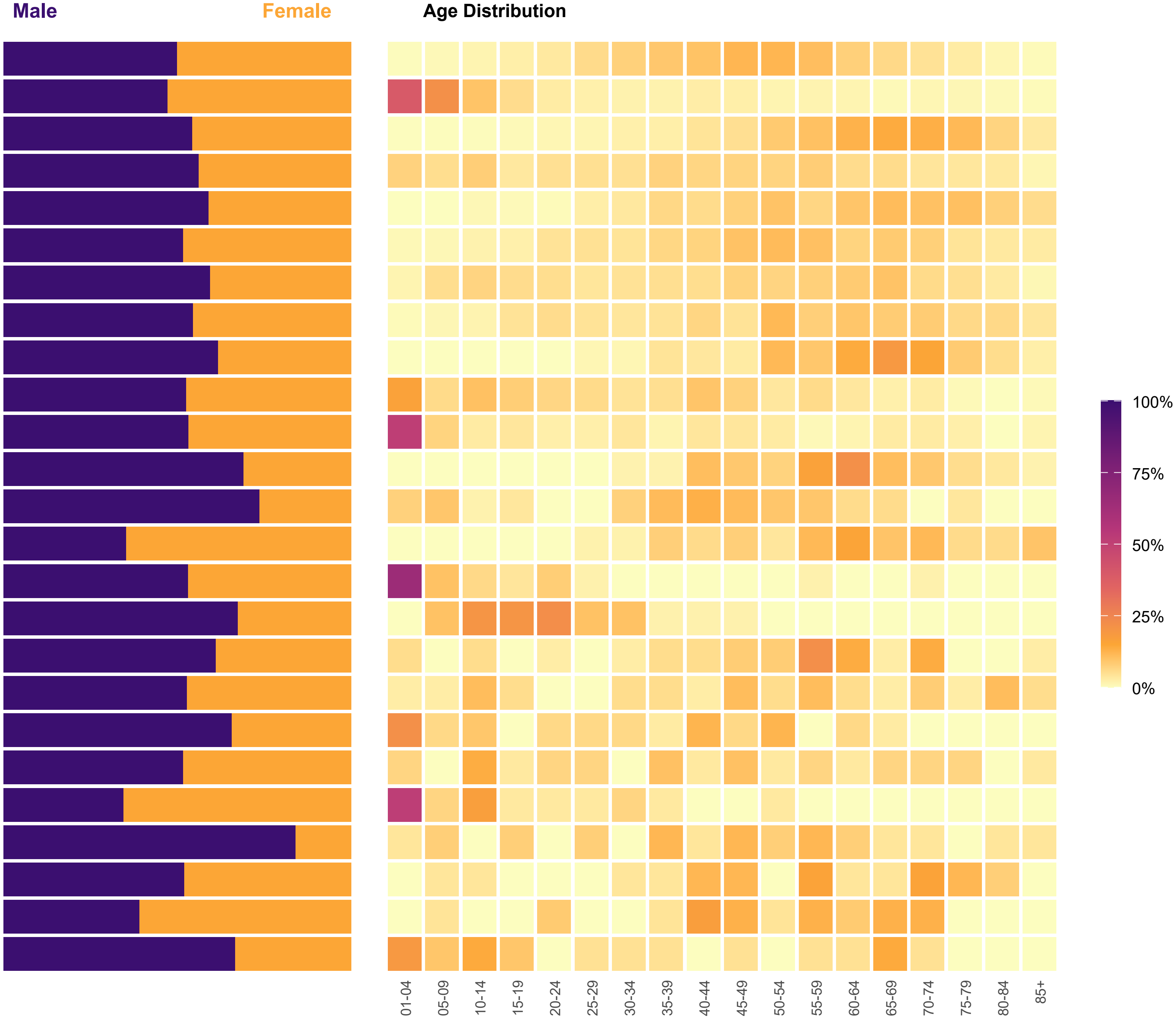

Primary Site: Cervix uteri

Top 21 Morphology Groups | cases: 176,010

Morphology Group	n	%
Squamous cell carcinoma	131,048	74.5%
Adenocarcinoma	39,792	22.6%
Neuroendocrine tumor	2,234	1.3%
Carcinosarcoma	491	0.3%
Leiomyosarcoma	364	0.2%
Adenosarcoma	269	0.2%
Mullerian mixed tumor	248	0.1%
Adenoid basal carcinoma	194	0.1%
Diffuse large B-cell lymphoma	192	0.1%
Rhabdomyosarcoma	167	<0.1%
Sarcoma	151	<0.1%
Mesonephroma	139	<0.1%
Lymphoepithelial carcinoma	120	<0.1%
Melanoma	114	<0.1%
Transitional cell carcinoma	90	<0.1%
Adenoid cystic carcinoma	83	<0.1%
Mesodermal mixed tumor	63	<0.1%
Lymphoma	32	<0.1%
Choriocarcinoma	23	<0.1%
Endometrial stromal sarcoma	21	<0.1%
Follicular lymphoma	20	<0.1%

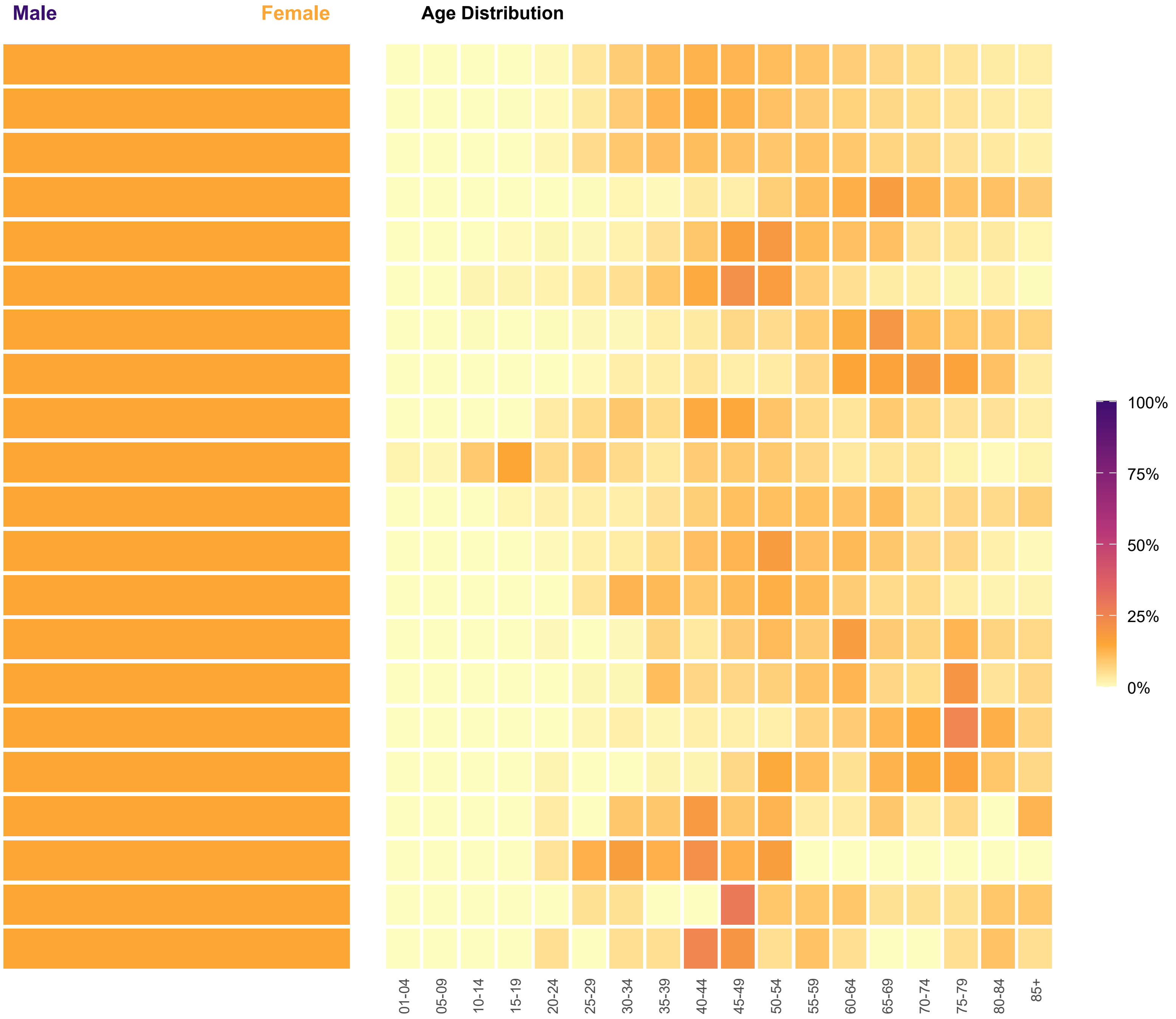

Primary Site: Colon and Rectum (excluding Appendix)

Top 25 Morphology Groups | cases: 2,021,688

Morphology Group	n	%
Adenocarcinoma	1,947,054	96.3%
Neuroendocrine tumor	53,408	2.6%
Squamous cell carcinoma	8,057	0.4%
Diffuse large B-cell lymphoma	3,119	0.2%
Gastrointestinal stromal tumor	1,721	<0.1%
Marginal zone B-cell lymphoma	1,494	<0.1%
Melanoma	949	<0.1%
Follicular lymphoma	859	<0.1%
Lymphoma	790	<0.1%
Mantle cell lymphoma	771	<0.1%
Leiomyosarcoma	629	<0.1%
Burkitt lymphoma	408	<0.1%
Sarcoma	390	<0.1%
Transitional cell carcinoma	342	<0.1%
Goblet cell carcinoid	241	<0.1%
Plasmacytoma	173	<0.1%
Kaposi sarcoma	171	<0.1%
Adenoid cystic carcinoma	130	<0.1%
T-cell lymphoma	113	<0.1%
lymphoma non-Hodgkin	77	<0.1%
Chronic lymphocytic leukemia	73	<0.1%
Liposarcoma	66	<0.1%
Carcinosarcoma	61	<0.1%
Hodgkin lymphoma	55	<0.1%
Hemangiosarcoma	50	<0.1%

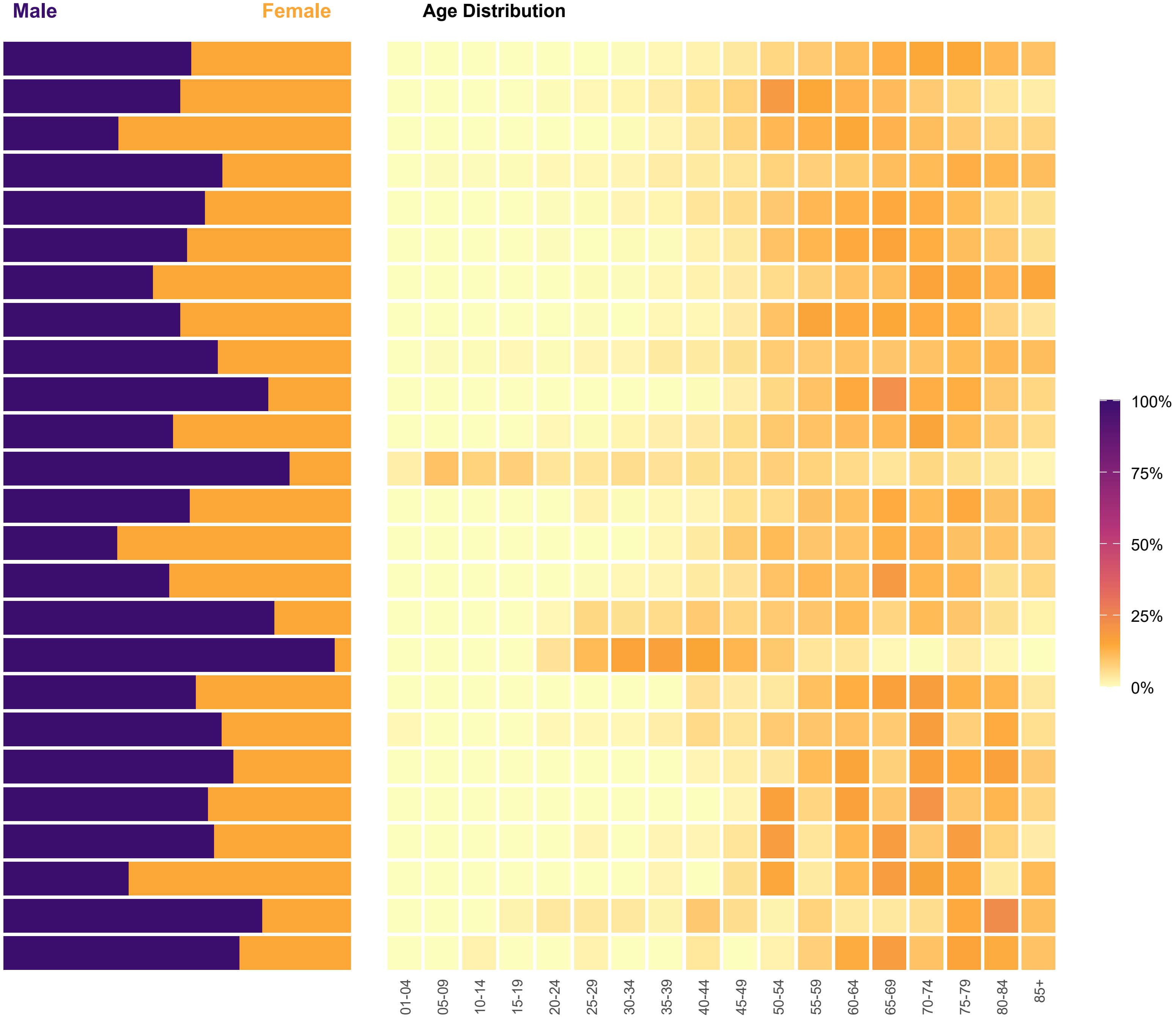

Primary Site: Corpus uteri

Top 25 Morphology Groups | cases: 544,523

Morphology Group	n	%
Adenocarcinoma	490,503	90.1%
Carcinosarcoma	15,985	2.9%
Leiomyosarcoma	11,596	2.1%
Mullerian mixed tumor	8,822	1.6%
Endometrial stromal sarcoma	6,396	1.2%
Adenosarcoma	2,577	0.5%
Sarcoma	2,468	0.5%
Squamous cell carcinoma	2,208	0.4%
Neuroendocrine tumor	927	0.2%
Mesodermal mixed tumor	924	0.2%
Rhabdomyosarcoma	509	<0.1%
Undifferentiated sarcoma	374	<0.1%
Choriocarcinoma	371	<0.1%
Diffuse large B-cell lymphoma	130	<0.1%
Mesonephroma	75	<0.1%
Trophoblastic tumor	70	<0.1%
Transitional cell carcinoma	48	<0.1%
Mesenchymoma	42	<0.1%
CNS embryonal tumor	37	<0.1%
Marginal zone B-cell lymphoma	33	<0.1%
Lymphoma	30	<0.1%
Rhabdoid tumor	28	<0.1%
Liposarcoma	27	<0.1%
Hemangiosarcoma	26	<0.1%
Ewing sarcoma	26	<0.1%

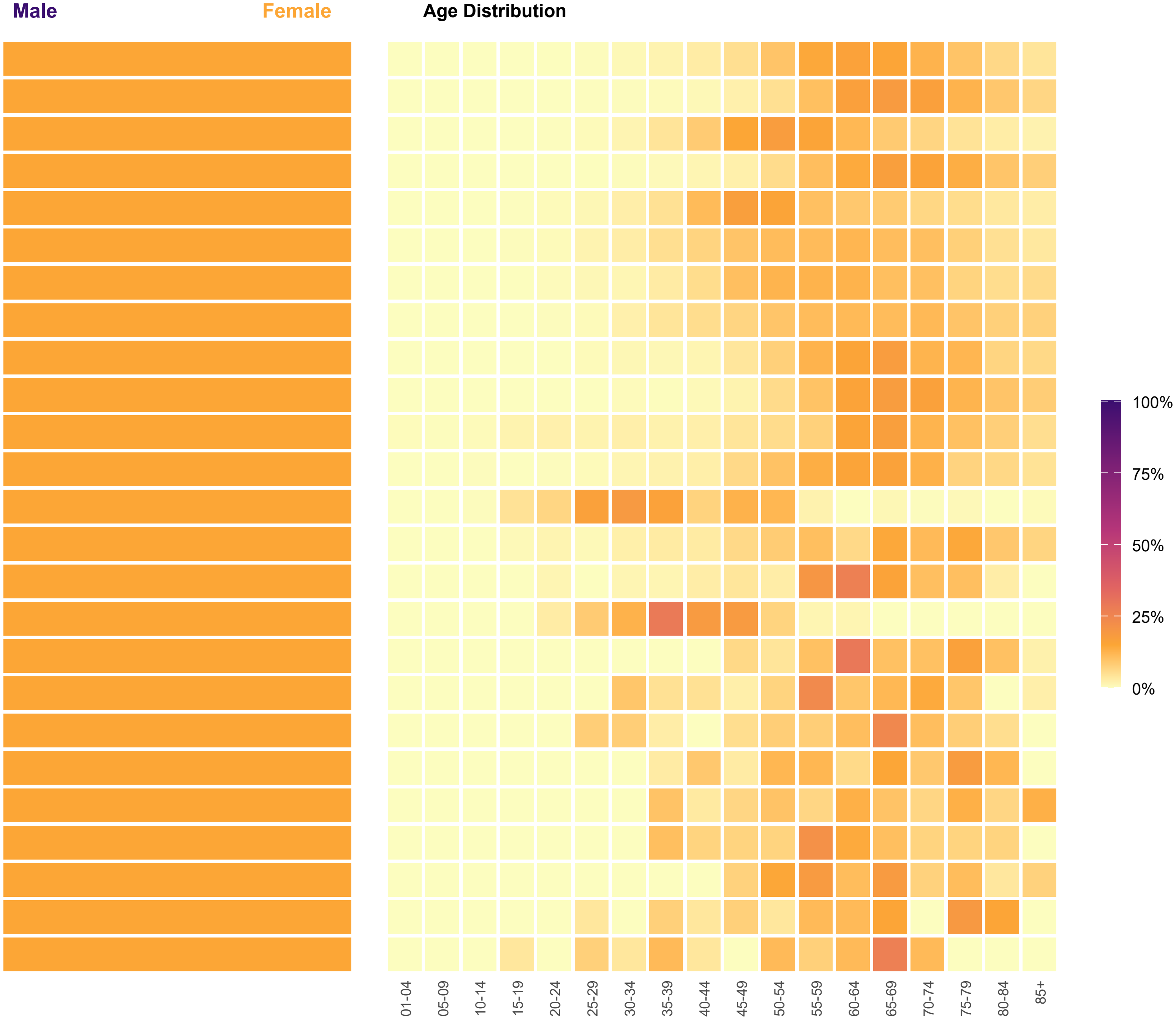

Primary Site: Digestive Other

Top 14 Morphology Groups | cases: 22,494

Morphology Group	n	%
Adenocarcinoma	17,076	75.9%
Neuroendocrine tumor	2,450	10.9%
Gastrointestinal stromal tumor	1,882	8.4%
Diffuse large B-cell lymphoma	246	1.1%
Sarcoma	122	0.5%
Mantle cell lymphoma	109	0.5%
Marginal zone B-cell lymphoma	104	0.5%
Squamous cell carcinoma	88	0.4%
Kaposi sarcoma	76	0.3%
Lymphoma	59	0.3%
T-cell lymphoma	46	0.2%
Follicular lymphoma	39	0.2%
Burkitt lymphoma	37	0.2%
Leiomyosarcoma	27	0.1%

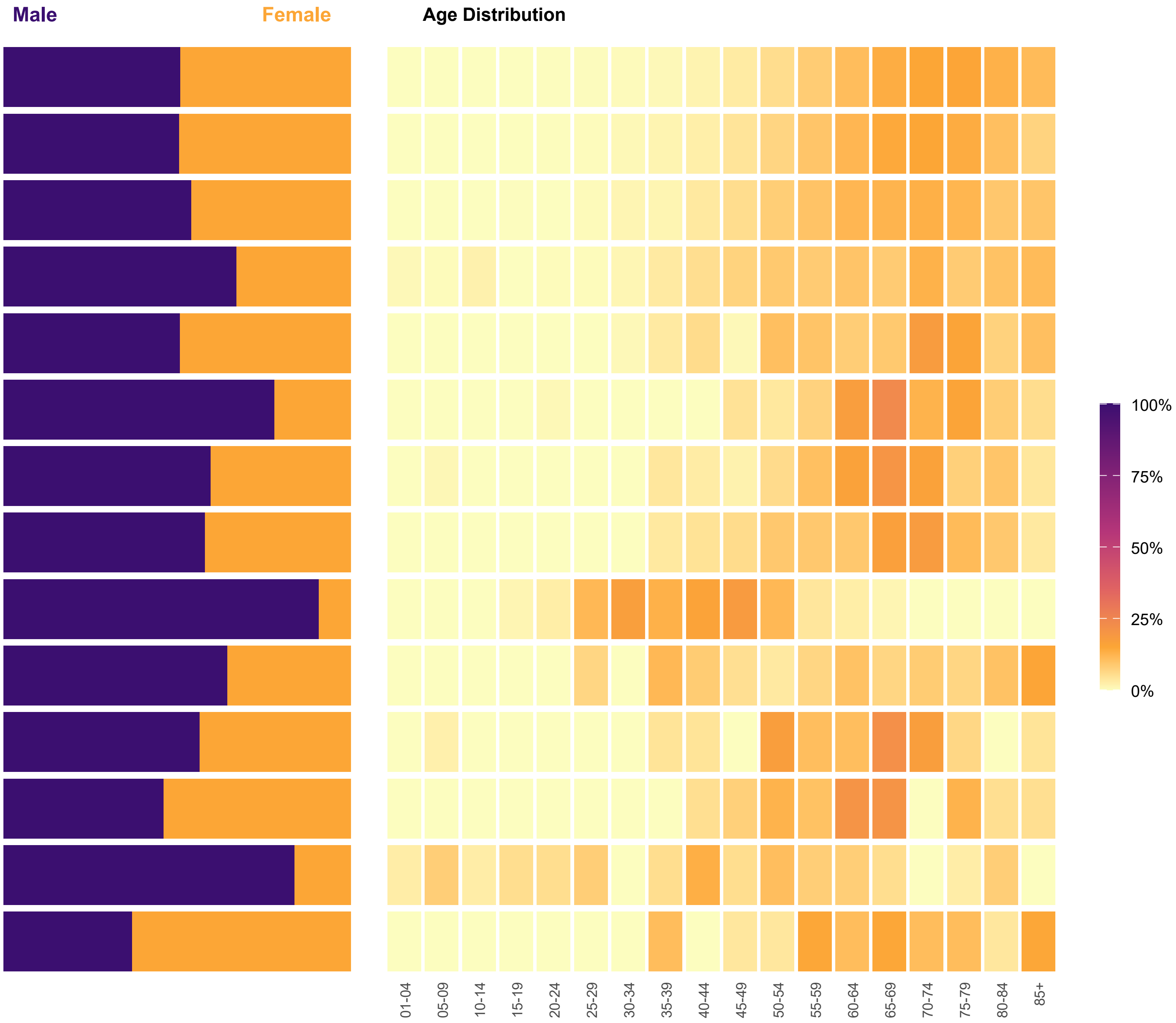

Primary Site: Endocrine Other

Top 3 Morphology Groups | cases: 897

Morphology Group	n	%
Paraganglioma	564	62.9%
Neuroendocrine tumor	202	22.5%
Adenocarcinoma	57	6.4%

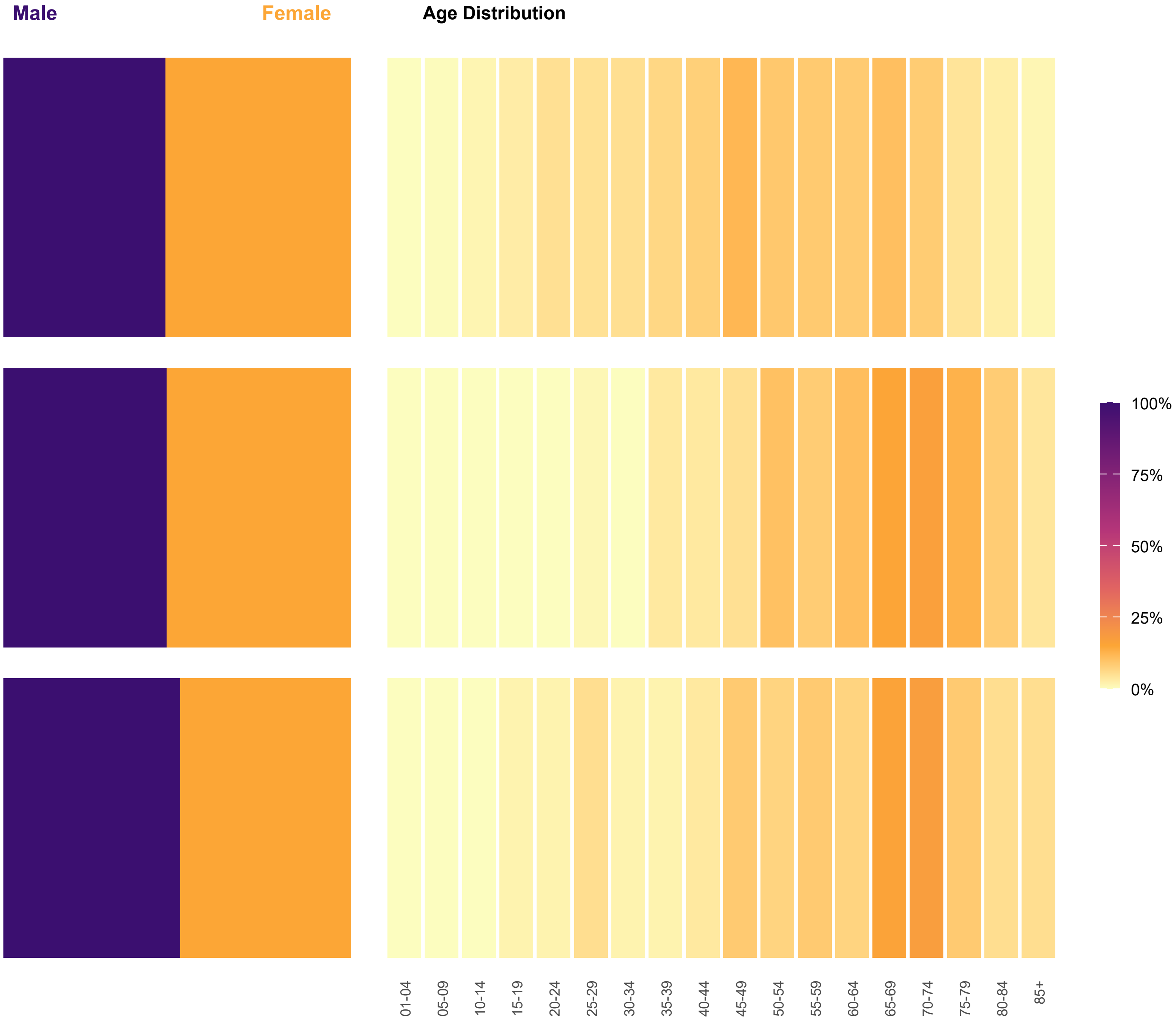

Primary Site: Epididymis

Top 15 Morphology Groups | cases: 4,886

Morphology Group	n	%
Squamous cell carcinoma	1,026	21.0%
Liposarcoma	792	16.2%
Adenocarcinoma	722	14.8%
Basal cell carcinoma	659	13.5%
Leiomyosarcoma	467	9.6%
Dedifferentiated liposarcoma	340	7.0%
Rhabdomyosarcoma	154	3.2%
Mesothelioma	141	2.9%
Sarcoma	138	2.8%
Melanoma	102	2.1%
Myxoid liposarcoma	54	1.1%
Transitional cell carcinoma	29	0.6%
Diffuse large B-cell lymphoma	23	0.5%
Neuroendocrine tumor	22	0.5%
Seminoma	22	0.5%

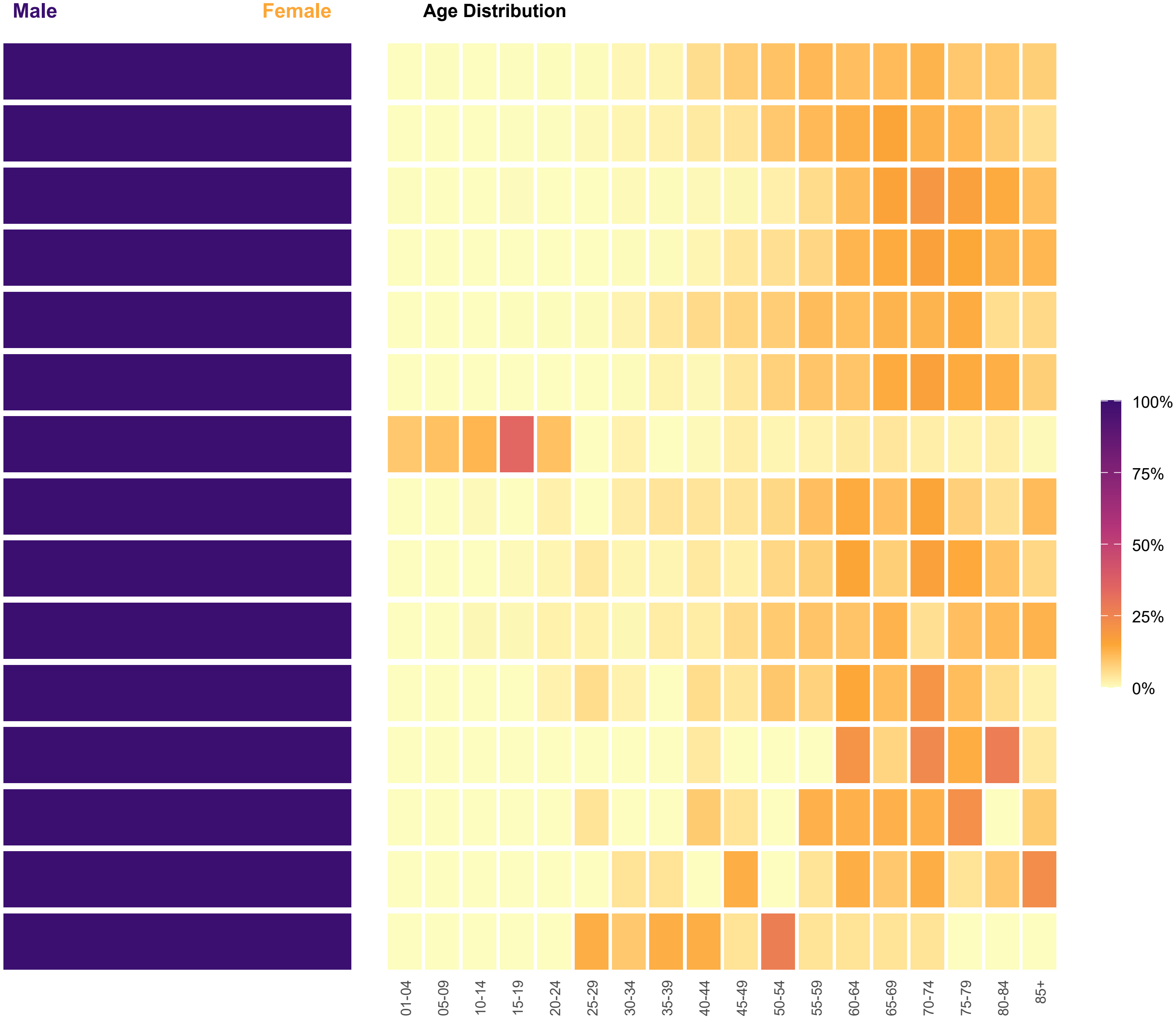

Primary Site: Esophagus

Top 14 Morphology Groups | cases: 203,883

Morphology Group	n	%
Adenocarcinoma	107,528	52.7%
Squamous cell carcinoma	92,791	45.5%
Neuroendocrine tumor	2,376	1.2%
Gastrointestinal stromal tumor	251	0.1%
Melanoma	191	<0.1%
Diffuse large B-cell lymphoma	139	<0.1%
Sarcoma	99	<0.1%
Leiomyosarcoma	77	<0.1%
Carcinosarcoma	57	<0.1%
Adenoid cystic carcinoma	51	<0.1%
Lymphoma	46	<0.1%
Marginal zone B-cell lymphoma	46	<0.1%
Lymphoepithelial carcinoma	23	<0.1%
Transitional cell carcinoma	23	<0.1%

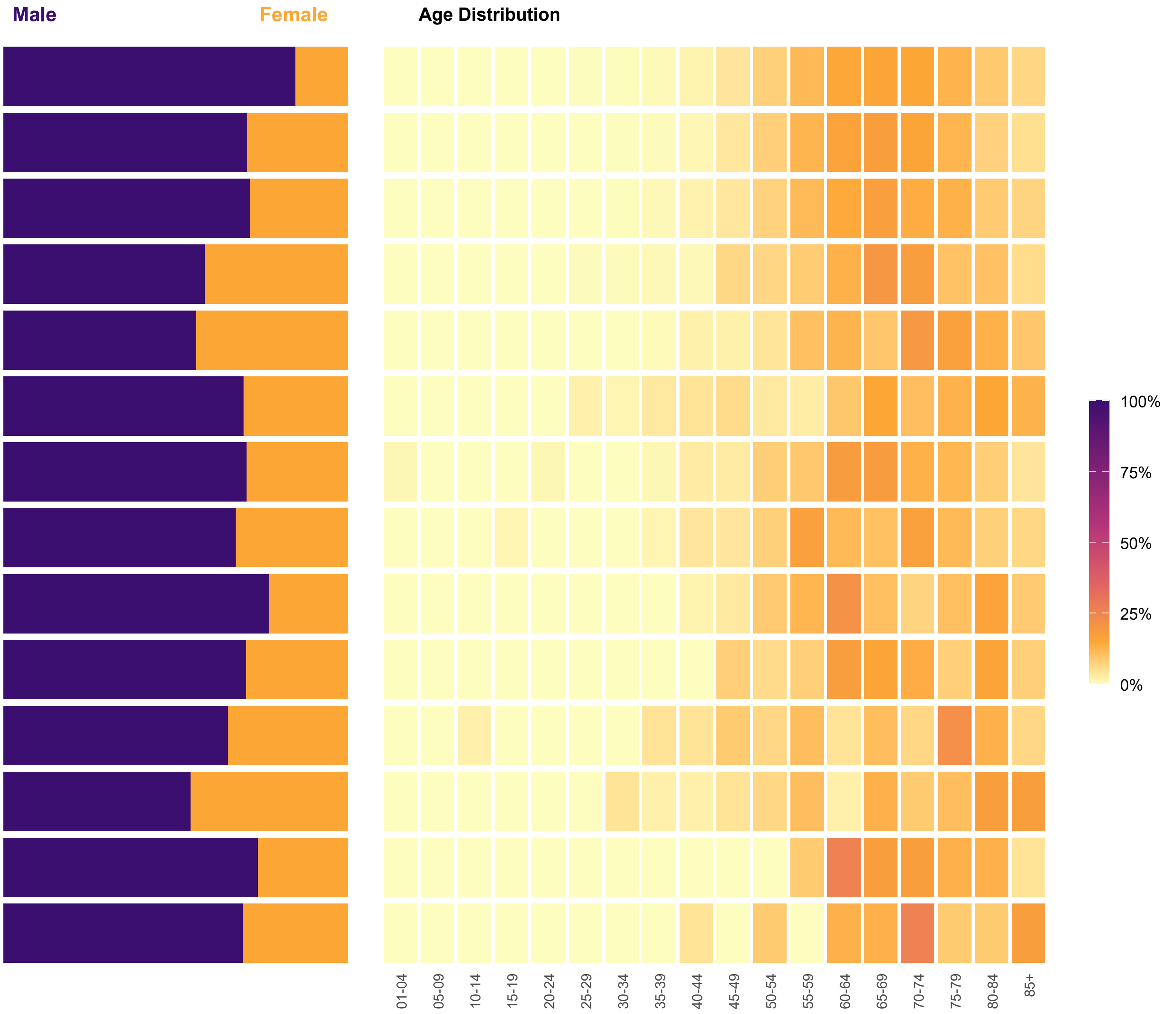

Primary Site: Extrahepatic Bile Ducts

Top 5 Morphology Groups | cases: 50,732

Morphology Group	n	%
Adenocarcinoma	50,183	98.9%
Neuroendocrine tumor	354	0.7%
Squamous cell carcinoma	60	0.1%
Combined hepatocellular carcinoma…	43	<0.1%
Hepatocellular carcinoma	31	<0.1%

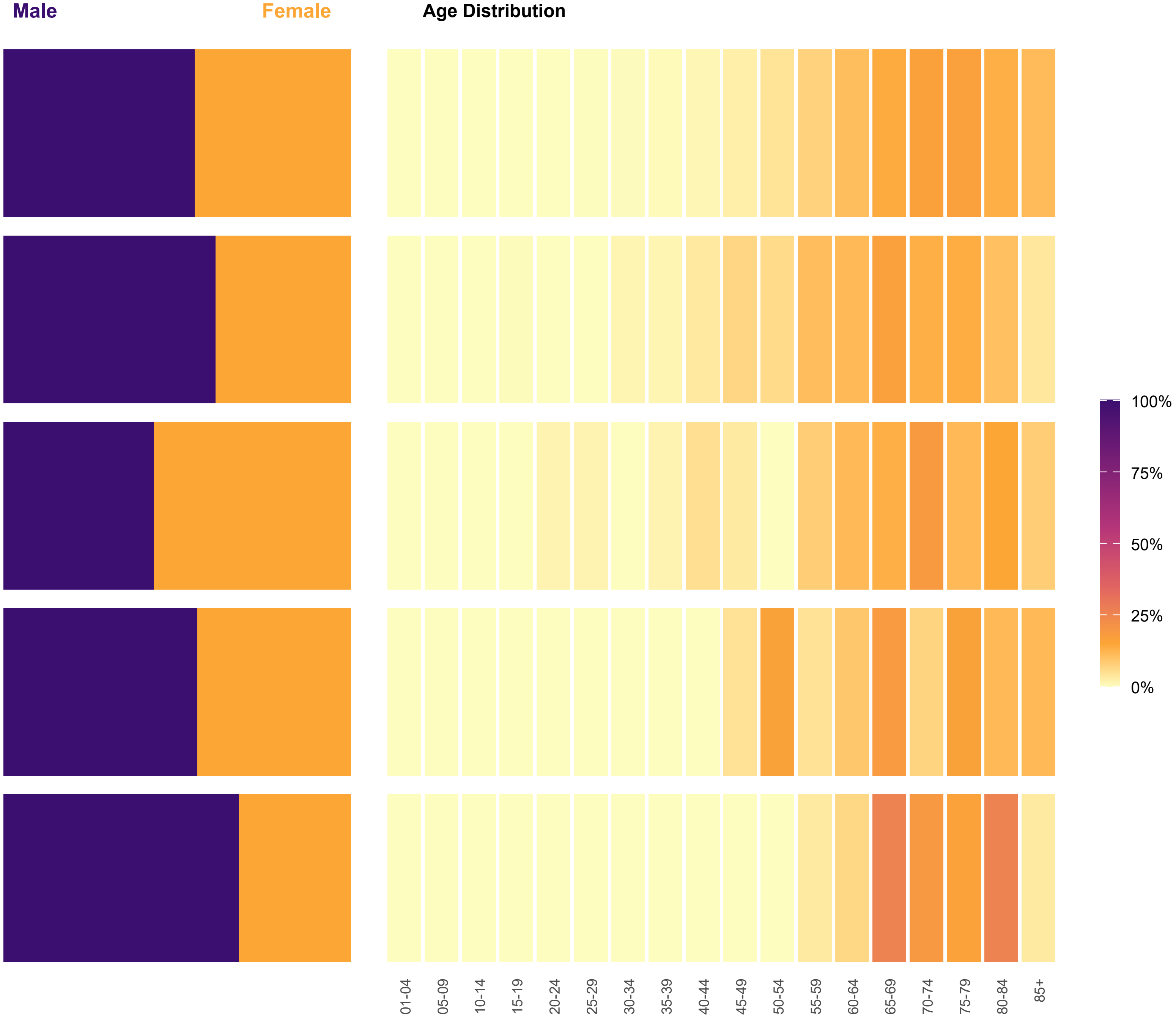

Primary Site: Eye and Orbit

Top 25 Morphology Groups | cases: 42,407

Morphology Group	n	%
Melanoma	28,094	66.2%
Marginal zone B-cell lymphoma	3,742	8.8%
Squamous cell carcinoma	3,722	8.8%
Retinoblastoma	2,037	4.8%
Diffuse large B-cell lymphoma	907	2.1%
Lymphoma	901	2.1%
Follicular lymphoma	725	1.7%
Adenocarcinoma	480	1.1%
Rhabdomyosarcoma	318	0.7%
Adenoid cystic carcinoma	292	0.7%
Mantle cell lymphoma	206	0.5%
lymphoma non-Hodgkin	163	0.4%
Sarcoma	72	0.2%
Plasmacytoma	65	0.2%
Basal cell carcinoma	55	0.1%
Lymphoplasmacytic lymphoma	54	0.1%
Chronic lymphocytic leukemia	50	0.1%
Neuroendocrine tumor	44	0.1%
Langerhans cell histiocytosis	36	<0.1%
Transitional cell carcinoma	34	<0.1%
Solitary fibrous tumor	30	<0.1%
Leiomyosarcoma	26	<0.1%
Myeloid sarcoma	26	<0.1%
Liposarcoma	26	<0.1%
T-cell lymphoma	23	<0.1%

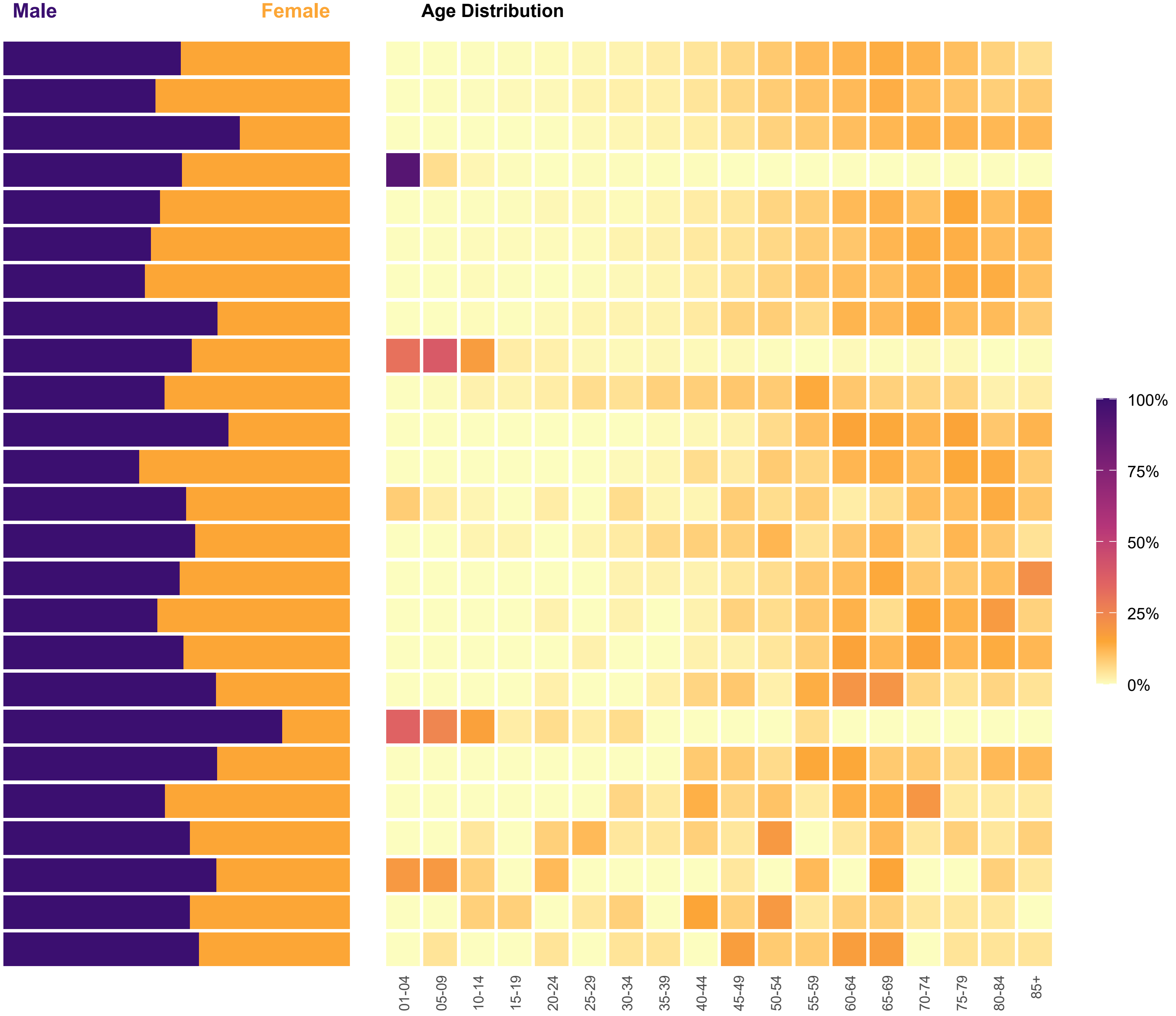

Primary Site: Fallopian Tube

Top 7 Morphology Groups | cases: 17,734

Morphology Group	n	%
Adenocarcinoma	16,945	95.6%
Carcinosarcoma	352	2.0%
Mullerian mixed tumor	180	1.0%
Transitional cell carcinoma	60	0.3%
Choriocarcinoma	35	0.2%
Neuroendocrine tumor	34	0.2%
Squamous cell carcinoma	23	0.1%

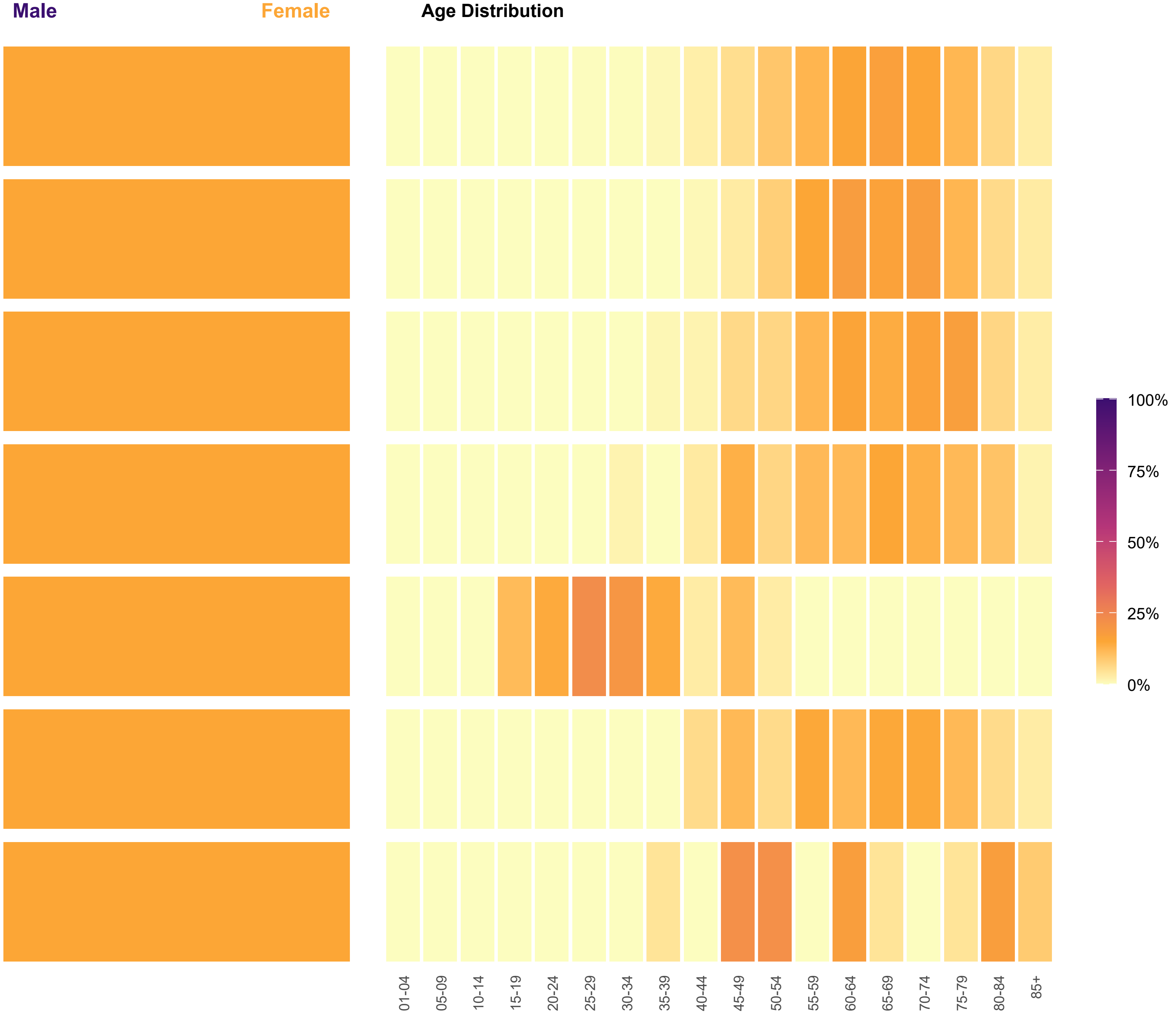

Primary Site: Floor of Mouth

Top 5 Morphology Groups | cases: 37,432

Morphology Group	n	%
Squamous cell carcinoma	36,487	97.5%
Adenocarcinoma	488	1.3%
Adenoid cystic carcinoma	245	0.7%
Diffuse large B-cell lymphoma	24	<0.1%
Neuroendocrine tumor	23	<0.1%

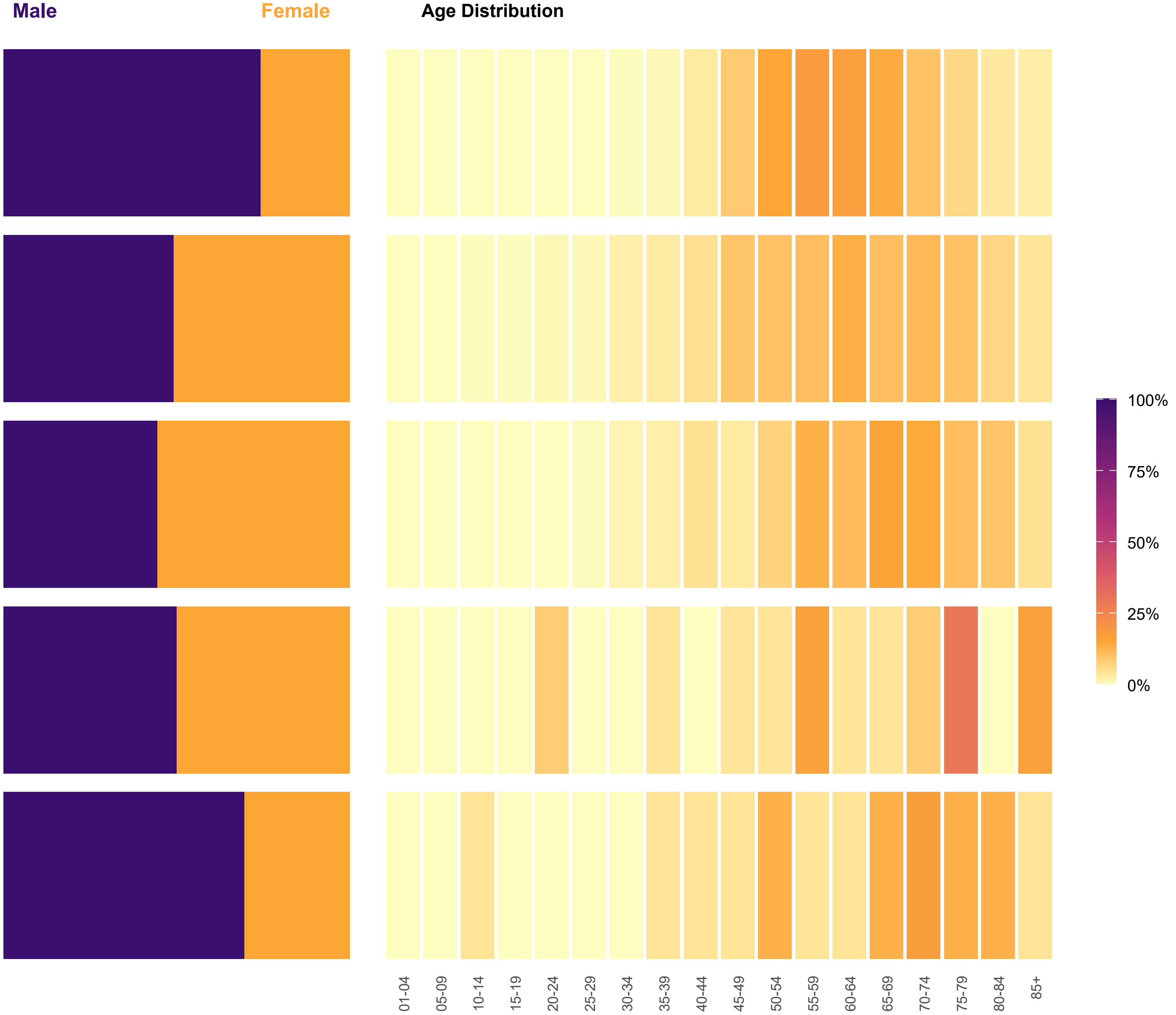

Primary Site: Gallbladder

Top 11 Morphology Groups | cases: 50,131

Morphology Group	n	%
Adenocarcinoma	47,537	94.8%
Neuroendocrine tumor	1,272	2.5%
Squamous cell carcinoma	781	1.6%
Carcinosarcoma	109	0.2%
Diffuse large B-cell lymphoma	73	0.1%
Marginal zone B-cell lymphoma	51	0.1%
Follicular lymphoma	49	<0.1%
Sarcoma	32	<0.1%
Lymphoma	31	<0.1%
Melanoma	28	<0.1%
Chronic lymphocytic leukemia	23	<0.1%

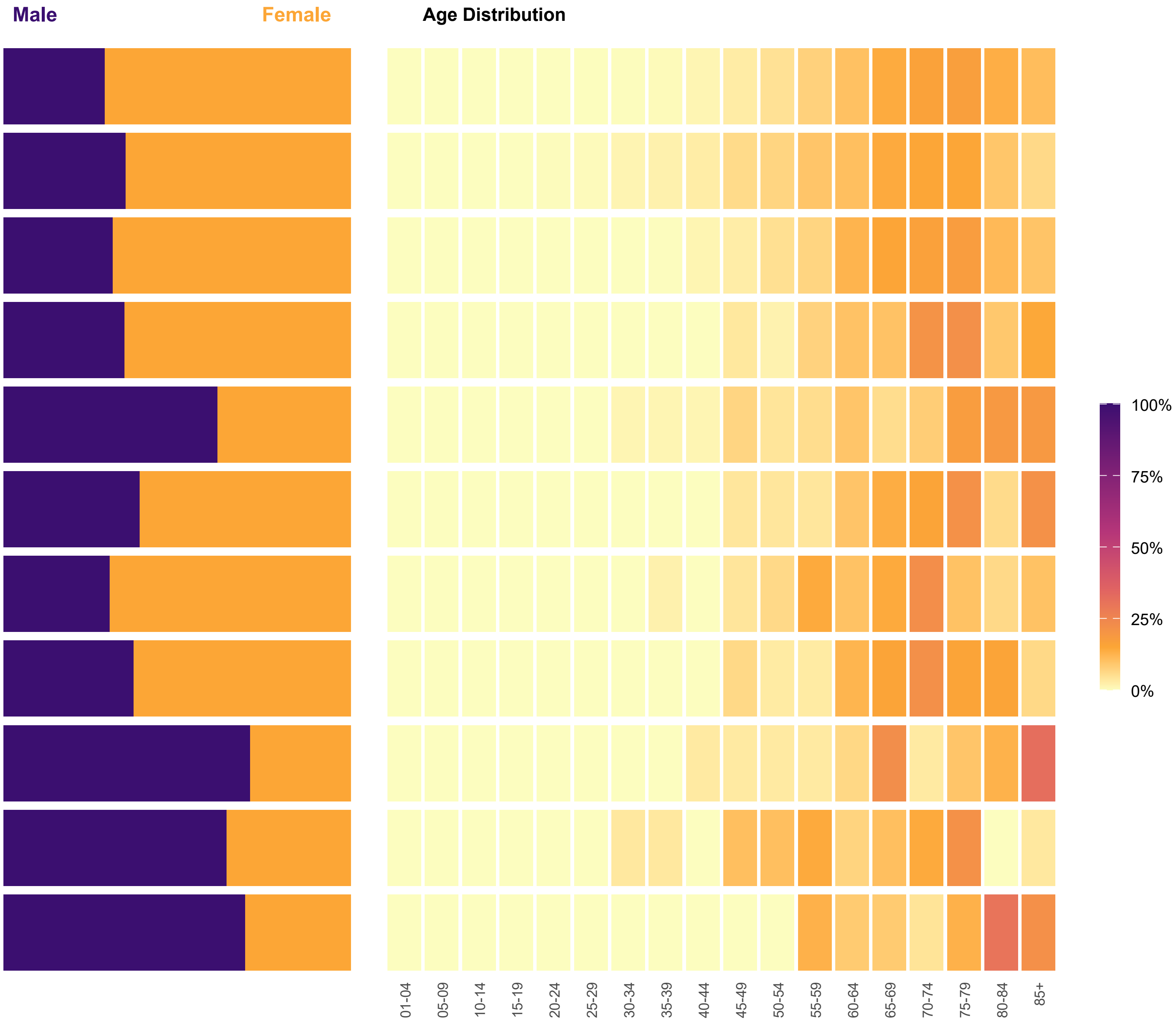

Primary Site: Gum

Top 12 Morphology Groups | cases: 28,144

Morphology Group	n	%
Squamous cell carcinoma	25,930	92.1%
Adenocarcinoma	964	3.4%
Adenoid cystic carcinoma	268	1.0%
Melanoma	261	0.9%
Diffuse large B-cell lymphoma	201	0.7%
Kaposi sarcoma	66	0.2%
Lymphoma	57	0.2%
Plasmacytoma	51	0.2%
Sarcoma	50	0.2%
Neuroendocrine tumor	30	0.1%
Myoepithelial carcinoma	20	<0.1%
Hemangiosarcoma	20	<0.1%

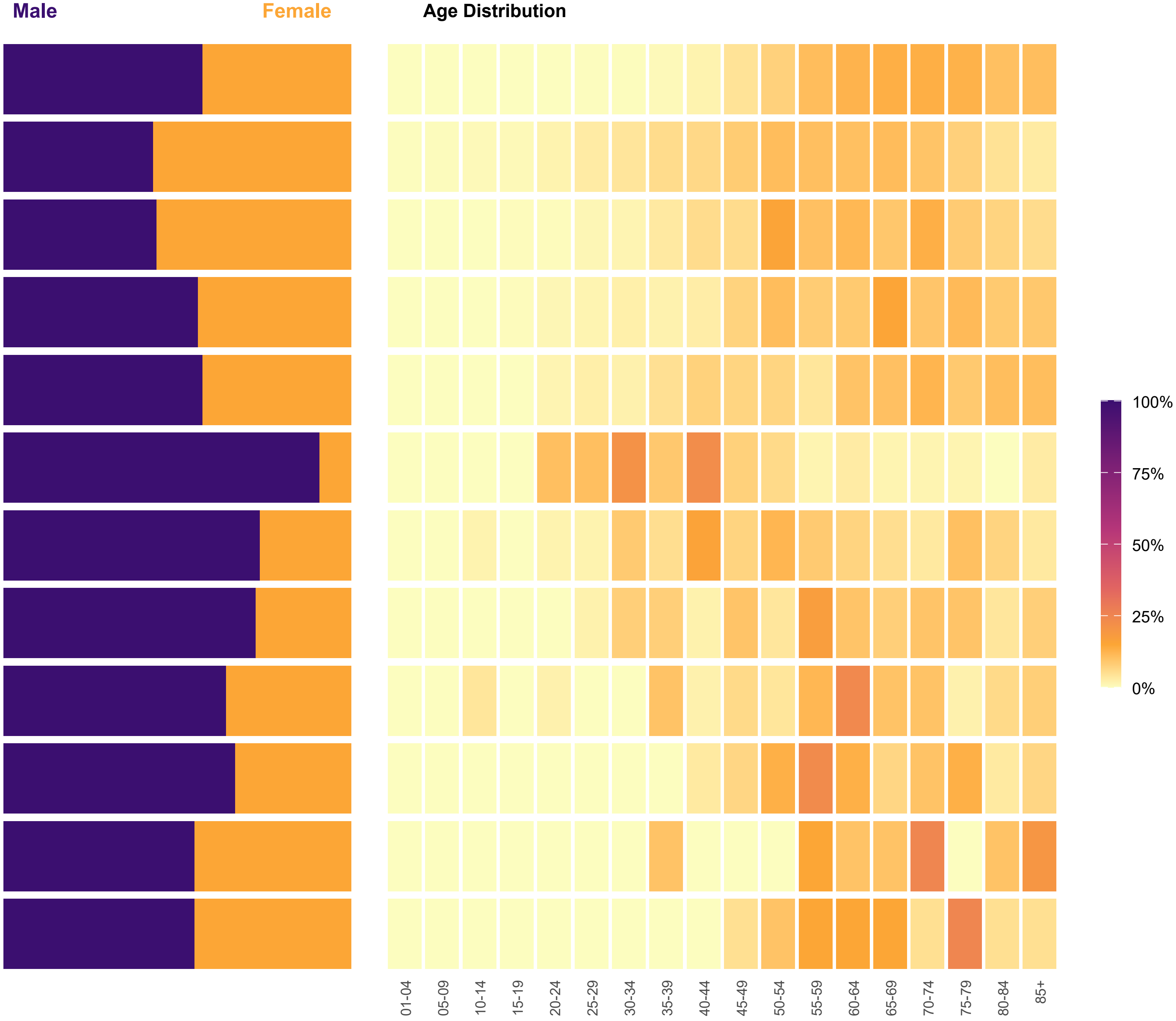

Primary Site: Heart

Top 25 Morphology Groups | cases: 55,455

Morphology Group	n	%
Mesothelioma	44,296	79.9%
Diffuse large B-cell lymphoma	1,594	2.9%
B-cell lymphoma	1,392	2.5%
Sarcoma	857	1.5%
Lymphoma	644	1.2%
Hemangiosarcoma	532	1.0%
Seminoma	433	0.8%
Germinoma	398	0.7%
Adenocarcinoma	391	0.7%
Neuroendocrine tumor	386	0.7%
Solitary fibrous tumor	371	0.7%
Hodgkin lymphoma	355	0.6%
Mixed germ cell tumor	309	0.6%
Yolk sac tumor	259	0.5%
Germ cell tumor nonseminomatous	196	0.4%
Leiomyosarcoma	189	0.3%
Neuroblastoma	181	0.3%
Squamous cell carcinoma	176	0.3%
Synovial sarcoma	166	0.3%
Thymoma	165	0.3%
Marginal zone B-cell lymphoma	139	0.3%
Teratoma	132	0.2%
Follicular lymphoma	130	0.2%
Liposarcoma	128	0.2%
Ganglioneuroblastoma	125	0.2%

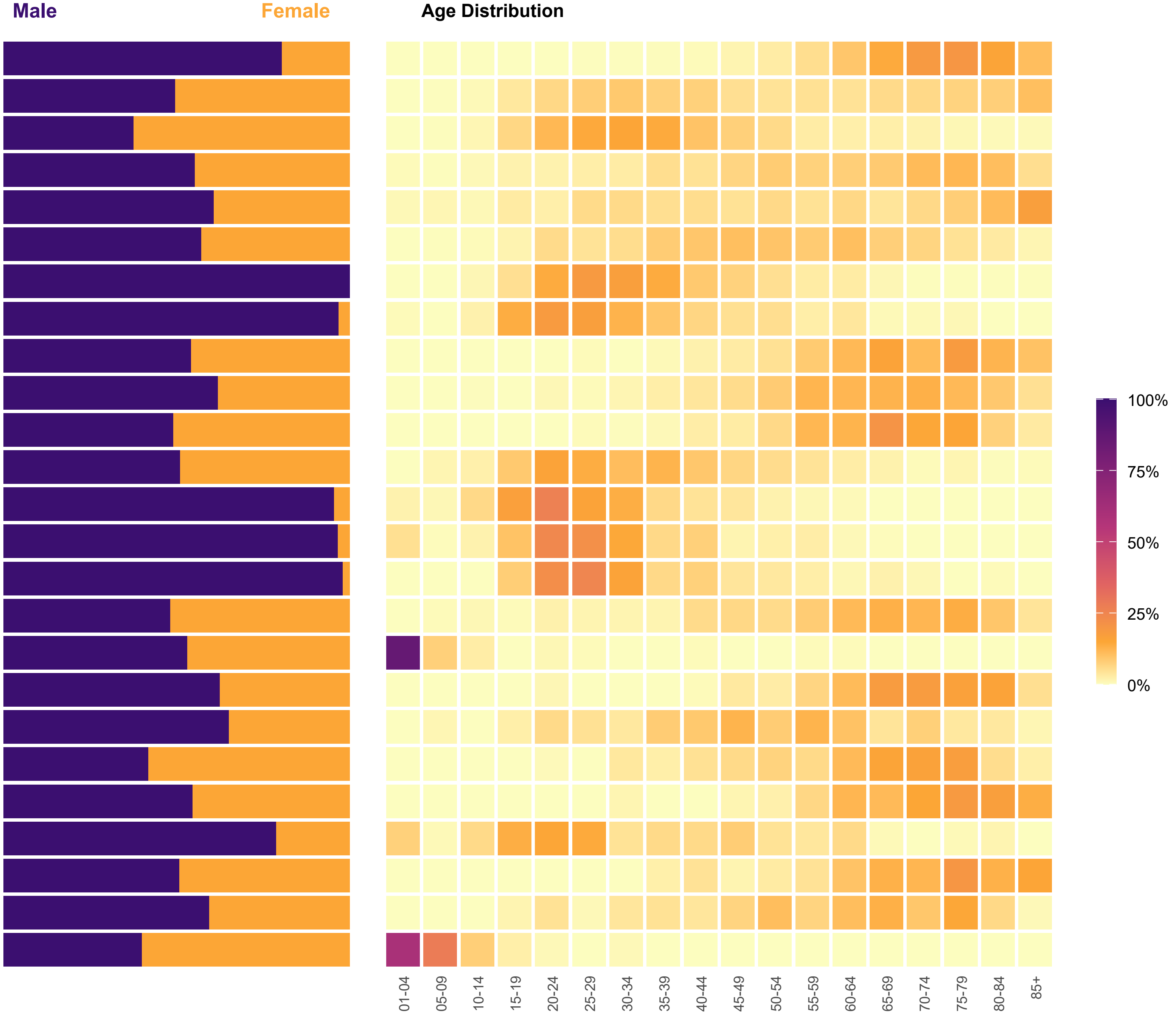

Primary Site: Hematopoetic and reticuloendothelial

Top 25 Morphology Groups | cases: 1,447,229

Morphology Group	n	%
Plasmacytoma	179,836	12.4%
Chronic lymphocytic leukemia	154,538	10.7%
Diffuse large B-cell lymphoma	123,995	8.6%
Myelodysplastic syndrome	122,176	8.4%
Adenocarcinoma	117,007	8.1%
Acute myeloid leukemia	112,624	7.8%
Follicular lymphoma	82,145	5.7%
Leukemia	75,606	5.2%
Hodgkin lymphoma	70,932	4.9%
Lymphoma	62,296	4.3%
Squamous cell carcinoma	45,052	3.1%
Polycythemia vera	41,921	2.9%
Essential thrombocythemia	40,047	2.8%
Chronic myeloid leukemia	37,880	2.6%
Neuroendocrine tumor	32,655	2.3%
Marginal zone B-cell lymphoma	19,298	1.3%
Mantle cell lymphoma	19,036	1.3%
Myeloproliferative neoplasm	16,079	1.1%
lymphoma non-Hodgkin	13,647	0.9%
Immunoproliferative disease	11,533	0.8%
Primary myelofibrosis	8,764	0.6%
T-cell lymphoma	8,714	0.6%
Hairy cell leukemia	8,213	0.6%
Lymphoplasmacytic lymphoma	7,026	0.5%
Burkitt lymphoma	6,466	0.4%

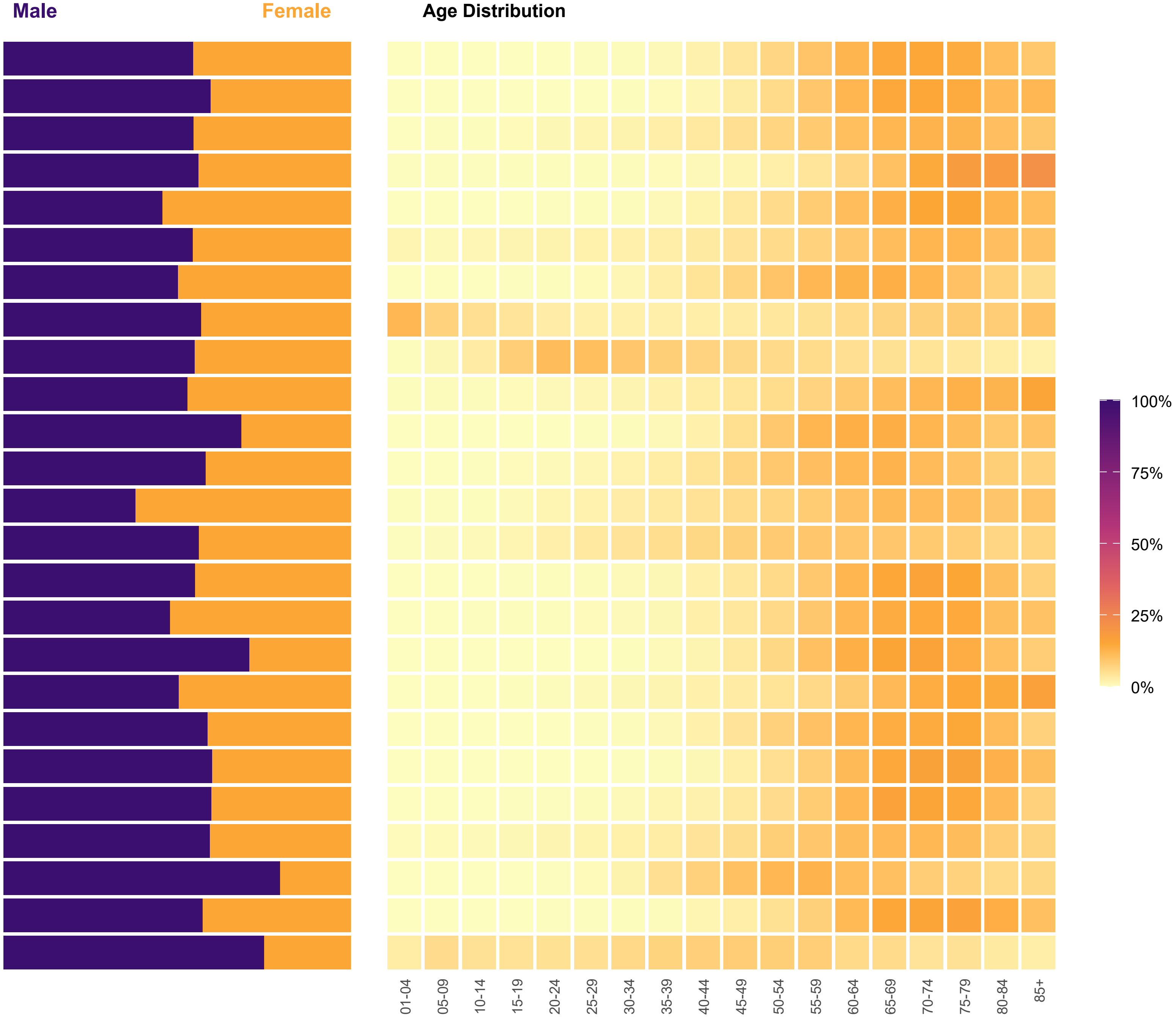

Primary Site: Hypopharynx

Top 11 Morphology Groups | cases: 44,333

Morphology Group	n	%
Squamous cell carcinoma	43,396	97.9%
Adenocarcinoma	203	0.5%
Neuroendocrine tumor	173	0.4%
Lymphoepithelial carcinoma	142	0.3%
Diffuse large B-cell lymphoma	64	0.1%
Adenoid cystic carcinoma	46	0.1%
Sarcoma	42	<0.1%
Transitional cell carcinoma	32	<0.1%
Liposarcoma	24	<0.1%
Marginal zone B-cell lymphoma	23	<0.1%
Carcinosarcoma	22	<0.1%

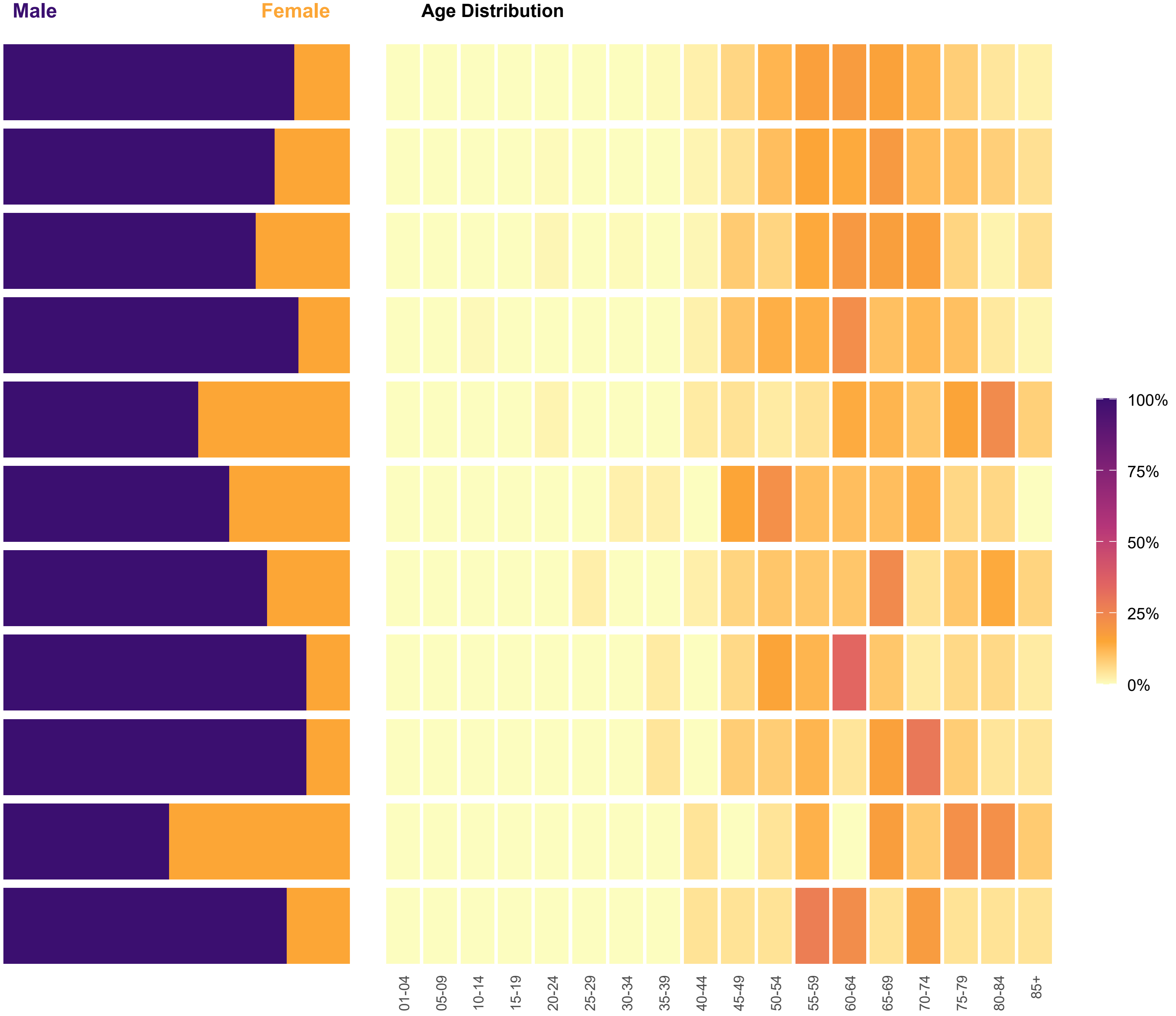

Primary Site: Intracranial Gland

Top 17 Morphology Groups | cases: 2,914

Morphology Group	n	%
Germinoma	1,018	34.9%
Pineoblastoma	835	28.7%
Pituitary carcinoma	276	9.5%
Mixed germ cell tumor	130	4.5%
Chordoma	77	2.6%
Adenocarcinoma	76	2.6%
Ependymoma	61	2.1%
Astrocytoma	45	1.5%
Glioblastoma	42	1.4%
Teratoma	37	1.3%
Diffuse large B-cell lymphoma	31	1.1%
CNS embryonal tumor	31	1.1%
Germ cell tumor nonseminomatous	30	1.0%
Neuroendocrine tumor	28	1.0%
Langerhans cell histiocytosis	24	0.8%
Glioma	21	0.7%
Choriocarcinoma	20	0.7%

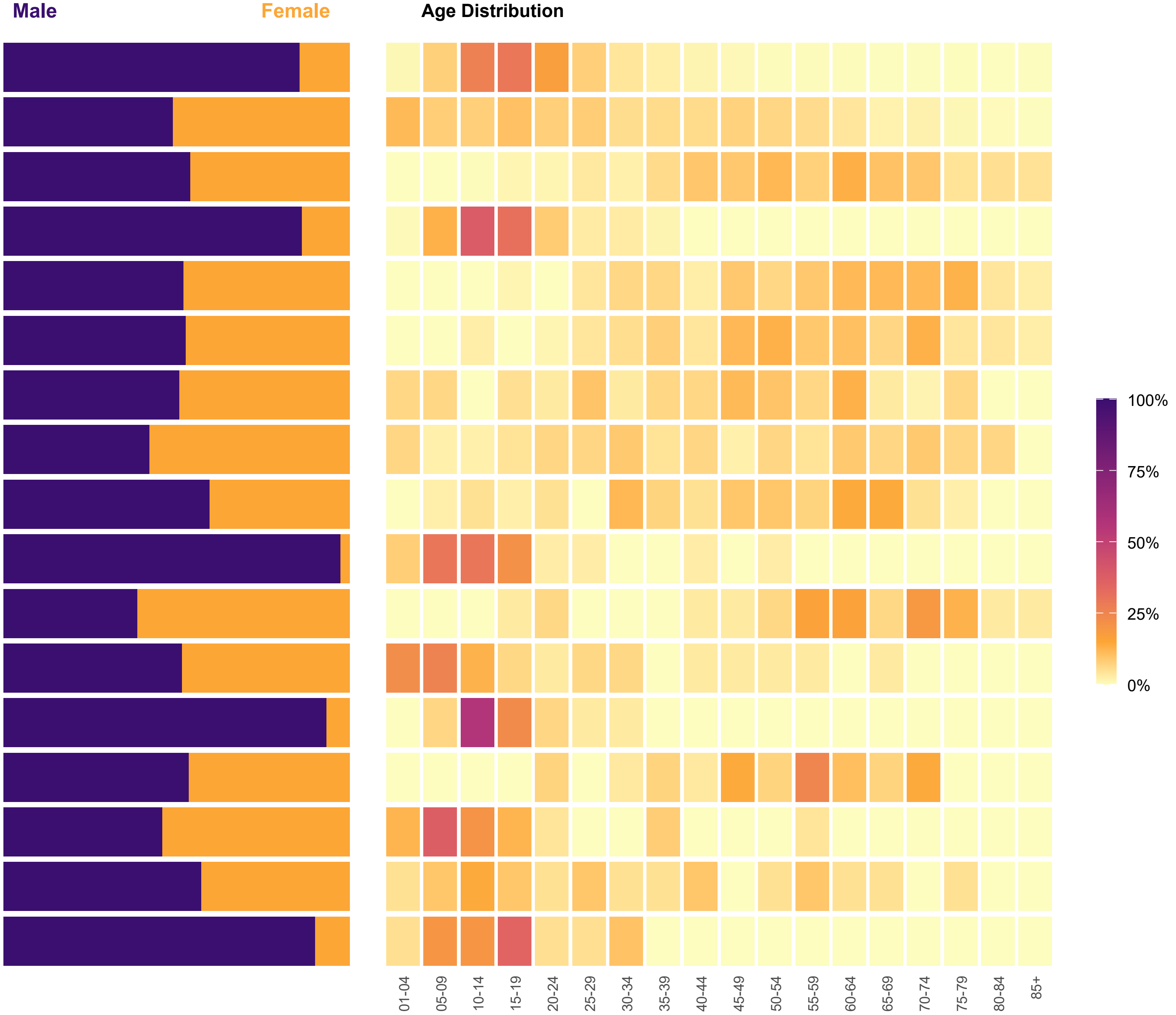

Primary Site: Kidney and Renal Pelvis

Top 25 Morphology Groups | cases: 671,586

Morphology Group	n	%
Adenocarcinoma	611,210	91.0%
Transitional cell carcinoma	45,906	6.8%
Nephroblastoma	5,228	0.8%
Granular cell carcinoma	2,521	0.4%
Squamous cell carcinoma	1,142	0.2%
Diffuse large B-cell lymphoma	887	0.1%
Neuroendocrine tumor	816	0.1%
Sarcoma	695	0.1%
Leiomyosarcoma	589	<0.1%
Liposarcoma	284	<0.1%
Marginal zone B-cell lymphoma	281	<0.1%
Lymphoma	264	<0.1%
Rhabdoid tumor	195	<0.1%
Follicular lymphoma	189	<0.1%
Malignant cystic nephroma	178	<0.1%
Dedifferentiated liposarcoma	147	<0.1%
Hemangiosarcoma	137	<0.1%
Carcinosarcoma	85	<0.1%
Synovial sarcoma	70	<0.1%
Ewing sarcoma	62	<0.1%
Neuroblastoma	58	<0.1%
CNS embryonal tumor	55	<0.1%
Plasmacytoma	44	<0.1%
Burkitt lymphoma	39	<0.1%
Rhabdomyosarcoma	38	<0.1%

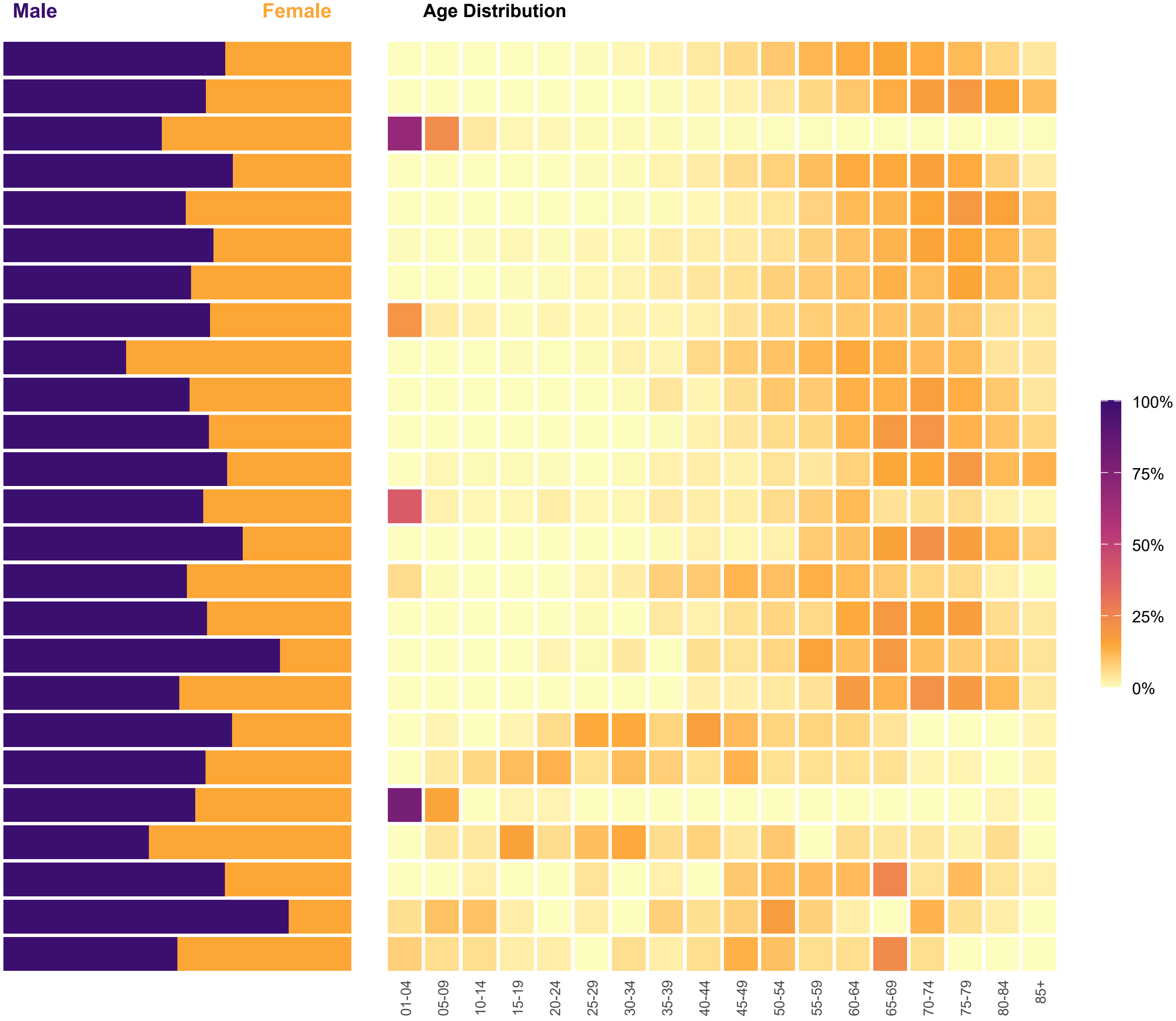

Primary Site: Larynx

Top 17 Morphology Groups | cases: 146,074

Morphology Group	n	%
Squamous cell carcinoma	143,178	98.0%
Neuroendocrine tumor	759	0.5%
Adenocarcinoma	466	0.3%
Chondrosarcoma	439	0.3%
Adenoid cystic carcinoma	201	0.1%
Sarcoma	153	0.1%
Diffuse large B-cell lymphoma	104	<0.1%
Lymphoepithelial carcinoma	103	<0.1%
Marginal zone B-cell lymphoma	92	<0.1%
Carcinosarcoma	78	<0.1%
Plasmacytoma	67	<0.1%
Leiomyosarcoma	46	<0.1%
Transitional cell carcinoma	40	<0.1%
Lymphoma	34	<0.1%
Follicular lymphoma	29	<0.1%
Liposarcoma	27	<0.1%
Rhabdomyosarcoma	24	<0.1%

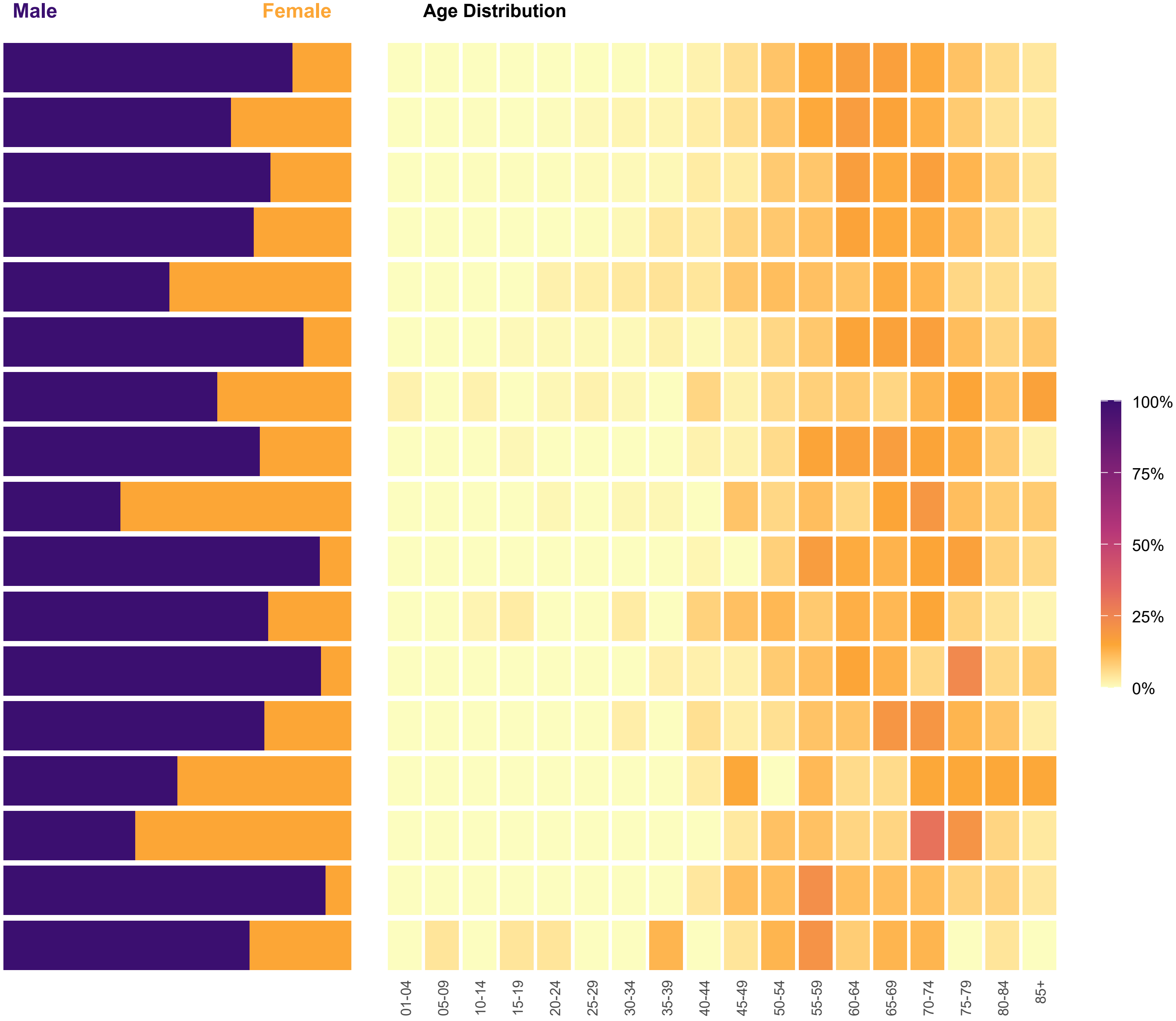

Primary Site: Lesions of anus and anal canal

Top 13 Morphology Groups | cases: 74,766

Morphology Group	n	%
Squamous cell carcinoma	61,769	82.6%
Adenocarcinoma	7,323	9.8%
Transitional cell carcinoma	3,257	4.4%
Melanoma	1,291	1.7%
Neuroendocrine tumor	754	1.0%
Diffuse large B-cell lymphoma	55	<0.1%
Basal cell carcinoma	47	<0.1%
Kaposi sarcoma	47	<0.1%
Gastrointestinal stromal tumor	42	<0.1%
Lymphoma	28	<0.1%
Plasmacytoma	27	<0.1%
Leiomyosarcoma	24	<0.1%
Sarcoma	21	<0.1%

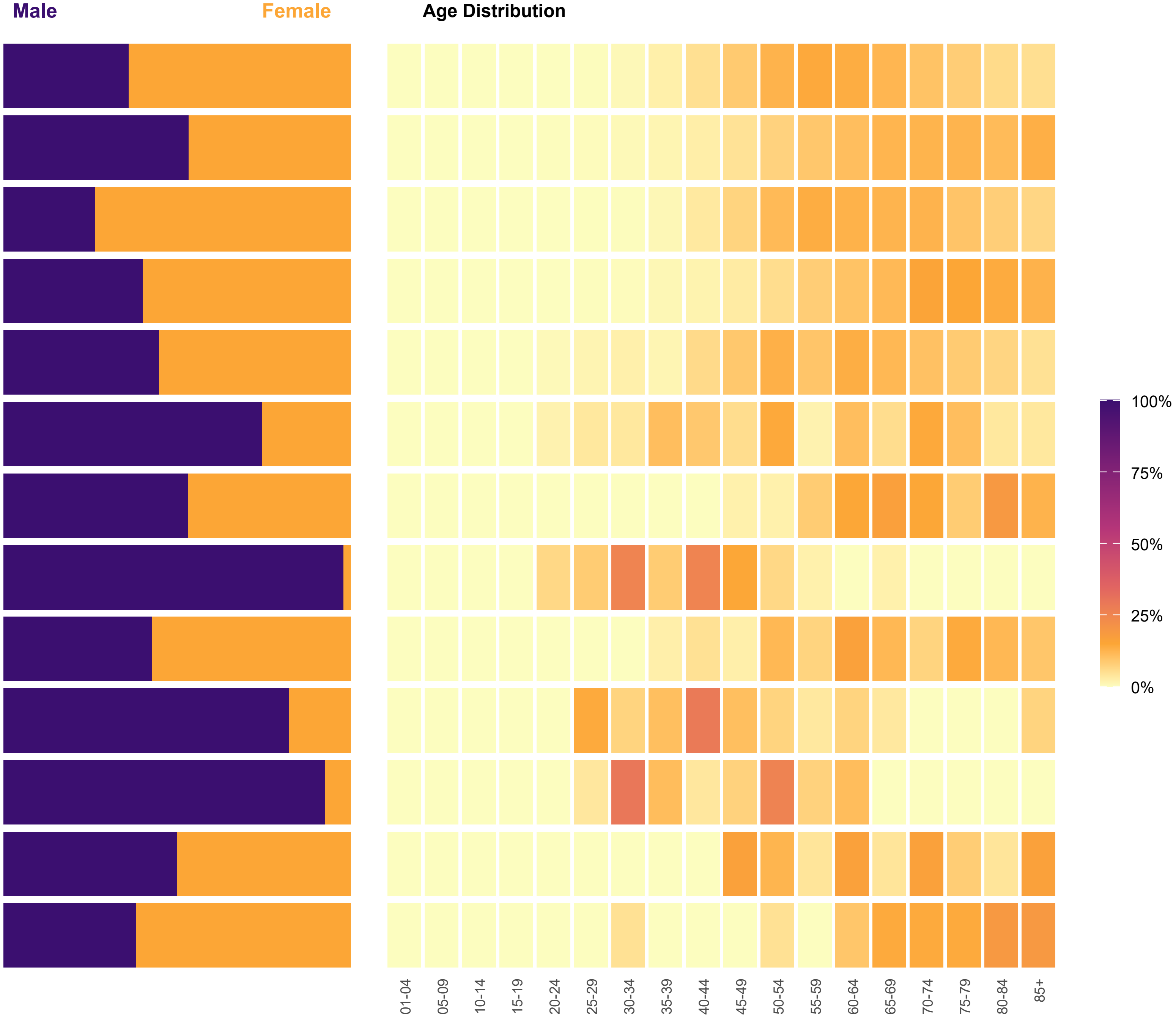

Primary Site: Lip

Top 7 Morphology Groups | cases: 25,151

Morphology Group	n	%
Squamous cell carcinoma	23,181	92.2%
Basal cell carcinoma	746	3.0%
Adenocarcinoma	686	2.7%
Adenoid cystic carcinoma	165	0.7%
Melanoma	123	0.5%
Marginal zone B-cell lymphoma	51	0.2%
Merkel cell carcinoma	42	0.2%

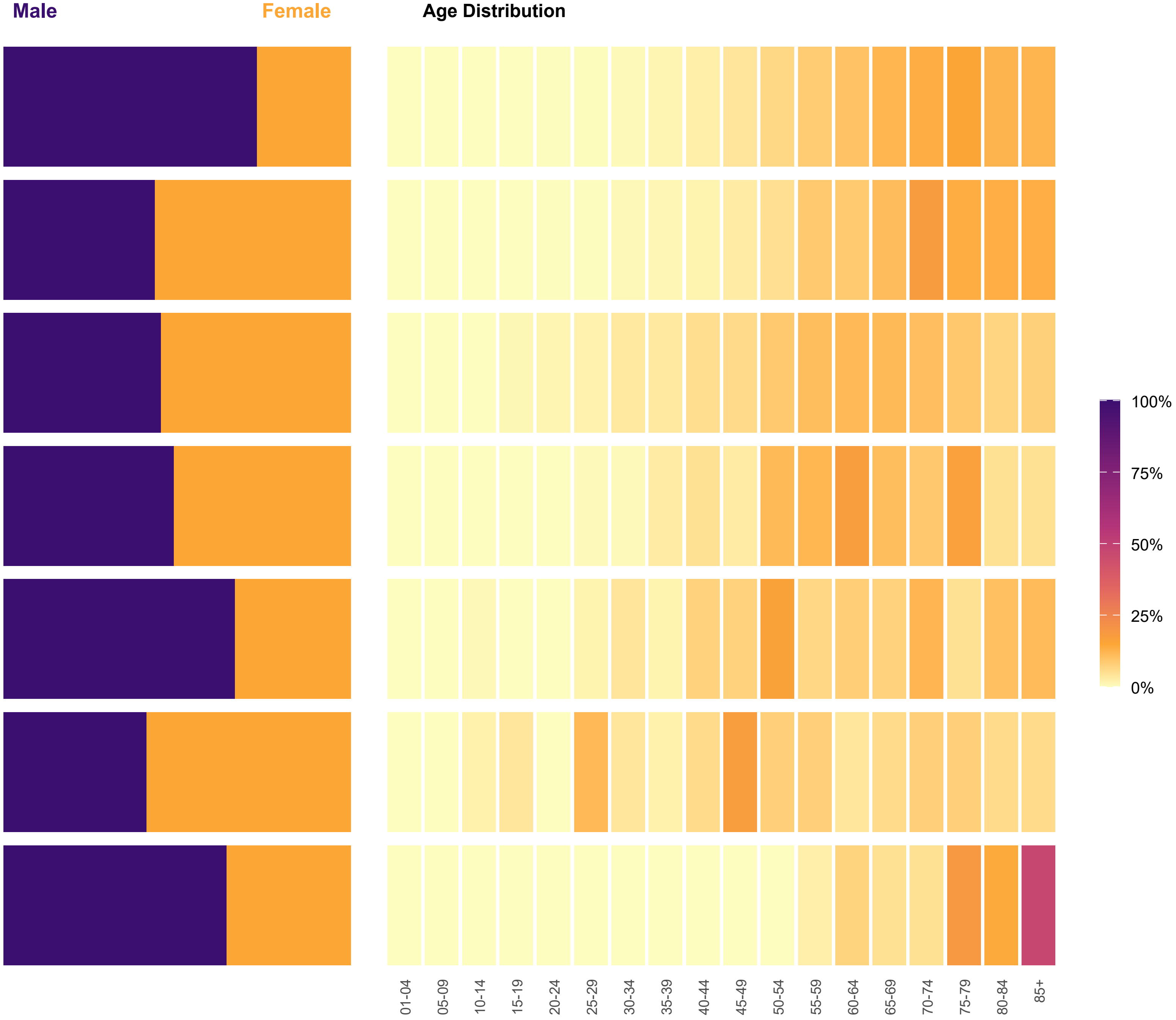

Primary Site: Liver and Intrahepatic Bile Ducts

Top 25 Morphology Groups | cases: 354,435

Morphology Group	n	%
Hepatocellular carcinoma	272,061	76.8%
Adenocarcinoma	70,005	19.8%
Combined hepatocellular carcinoma…	3,456	1.0%
Neuroendocrine tumor	1,957	0.6%
Diffuse large B-cell lymphoma	1,693	0.5%
Hepatoblastoma	1,315	0.4%
Hemangiosarcoma	1,100	0.3%
Sarcoma	415	0.1%
Lymphoma	382	0.1%
Epithelioid hemangioendothelioma	330	<0.1%
Leiomyosarcoma	217	<0.1%
Embryonal sarcoma	200	<0.1%
Marginal zone B-cell lymphoma	148	<0.1%
Squamous cell carcinoma	116	<0.1%
Follicular lymphoma	98	<0.1%
T-cell lymphoma	92	<0.1%
Hodgkin lymphoma	91	<0.1%
Carcinosarcoma	80	<0.1%
Burkitt lymphoma	80	<0.1%
Hemangioendothelioma	76	<0.1%
Kupffer cell sarcoma	39	<0.1%
Rhabdomyosarcoma	34	<0.1%
Undifferentiated sarcoma	33	<0.1%
Anaplastic large cell lymphoma	27	<0.1%
Plasmacytoma	26	<0.1%

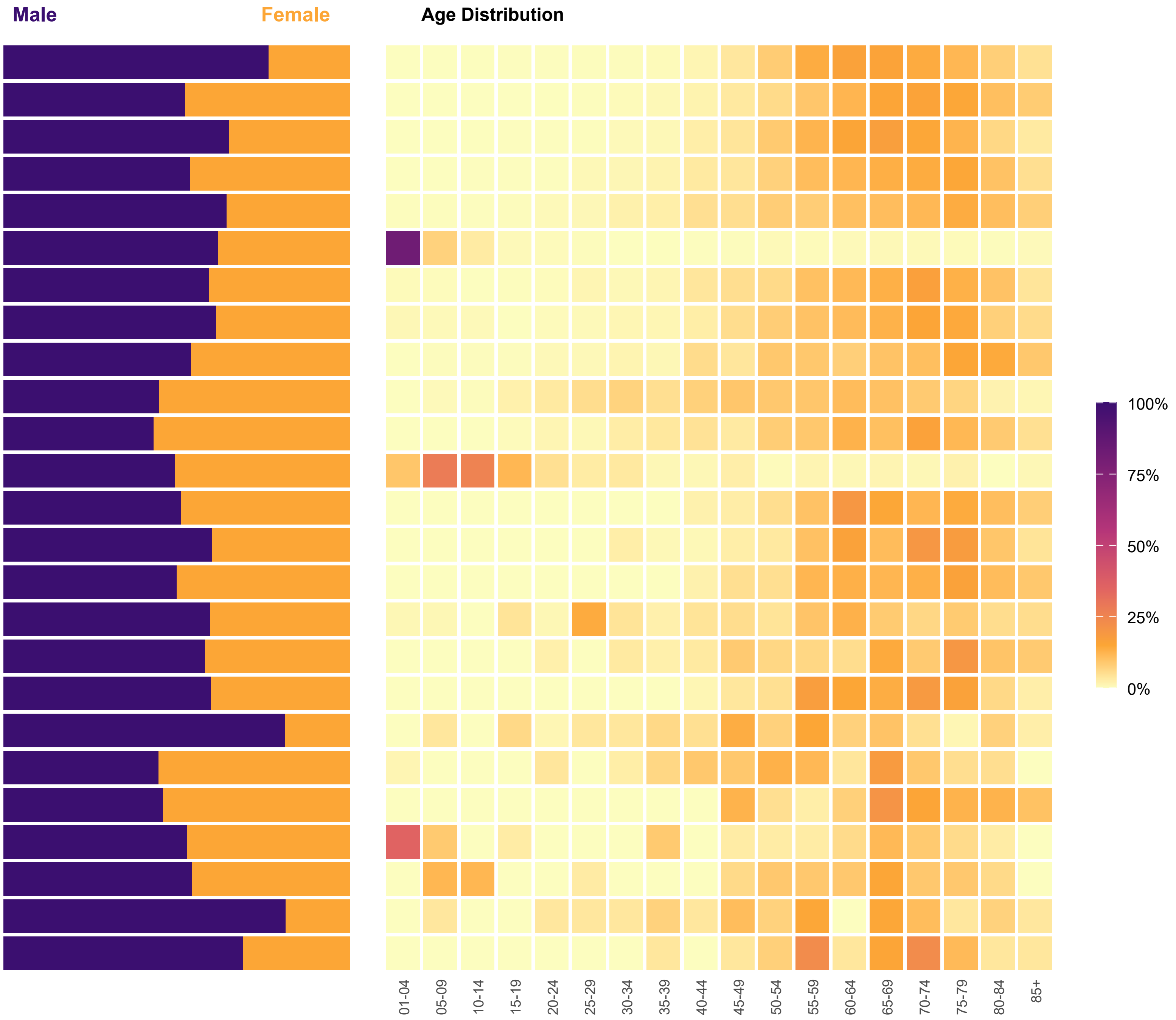

Primary Site: Long bones of lower limb and associated joints

Top 22 Morphology Groups | cases: 13,282

Morphology Group	n	%
Osteosarcoma	6,654	50.1%
Chondrosarcoma	2,895	21.8%
Diffuse large B-cell lymphoma	848	6.4%
Sarcoma	737	5.5%
Plasmacytoma	367	2.8%
malignant giant cell tumor of bone	303	2.3%
Langerhans cell histiocytosis	175	1.3%
Lymphoma	166	1.2%
Myxoid chondrosarcoma	155	1.2%
Adamantinoma of long bones	151	1.1%
Leiomyosarcoma	98	0.7%
Hemangiosarcoma	91	0.7%
Fibrosarcoma	71	0.5%
Ewing sarcoma	53	0.4%
Follicular lymphoma	52	0.4%
Undifferentiated sarcoma	48	0.4%
Synovial sarcoma	48	0.4%
Chondroblastoma	34	0.3%
Liposarcoma	28	0.2%
Myxofibrosarcoma	22	0.2%
Epithelioid hemangioendothelioma	21	0.2%
Marginal zone B-cell lymphoma	21	0.2%

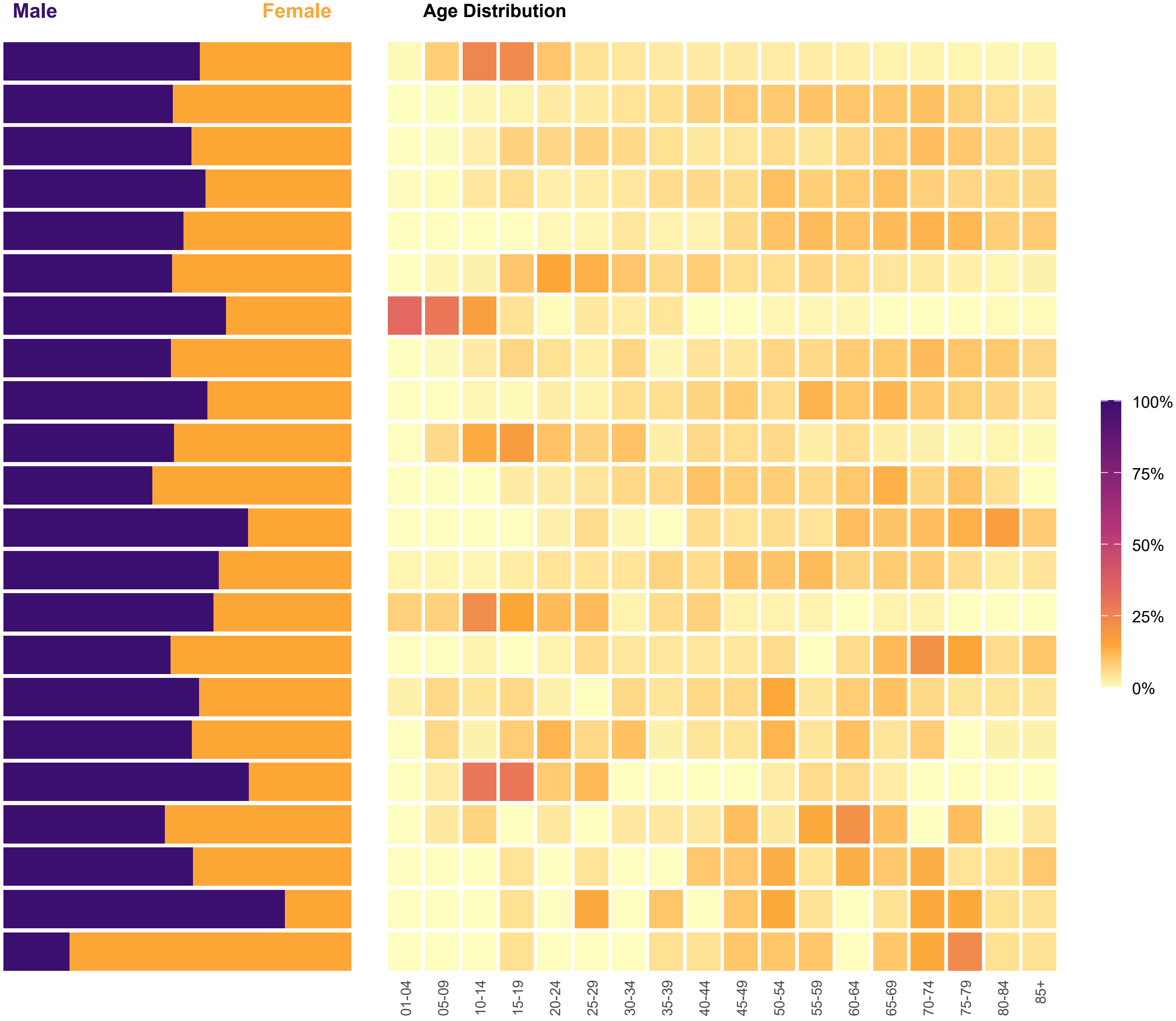

Primary Site: Long bones of upper limb and scapula and associated joints

Top 11 Morphology Groups | cases: 4,444

Morphology Group	n	%
Chondrosarcoma	1,589	35.8%
Osteosarcoma	1,311	29.5%
Plasmacytoma	442	9.9%
Diffuse large B-cell lymphoma	358	8.1%
Sarcoma	183	4.1%
Langerhans cell histiocytosis	102	2.3%
malignant giant cell tumor of bone	91	2.0%
Myxoid chondrosarcoma	56	1.3%
Lymphoma	52	1.2%
Ewing sarcoma	27	0.6%
Follicular lymphoma	23	0.5%

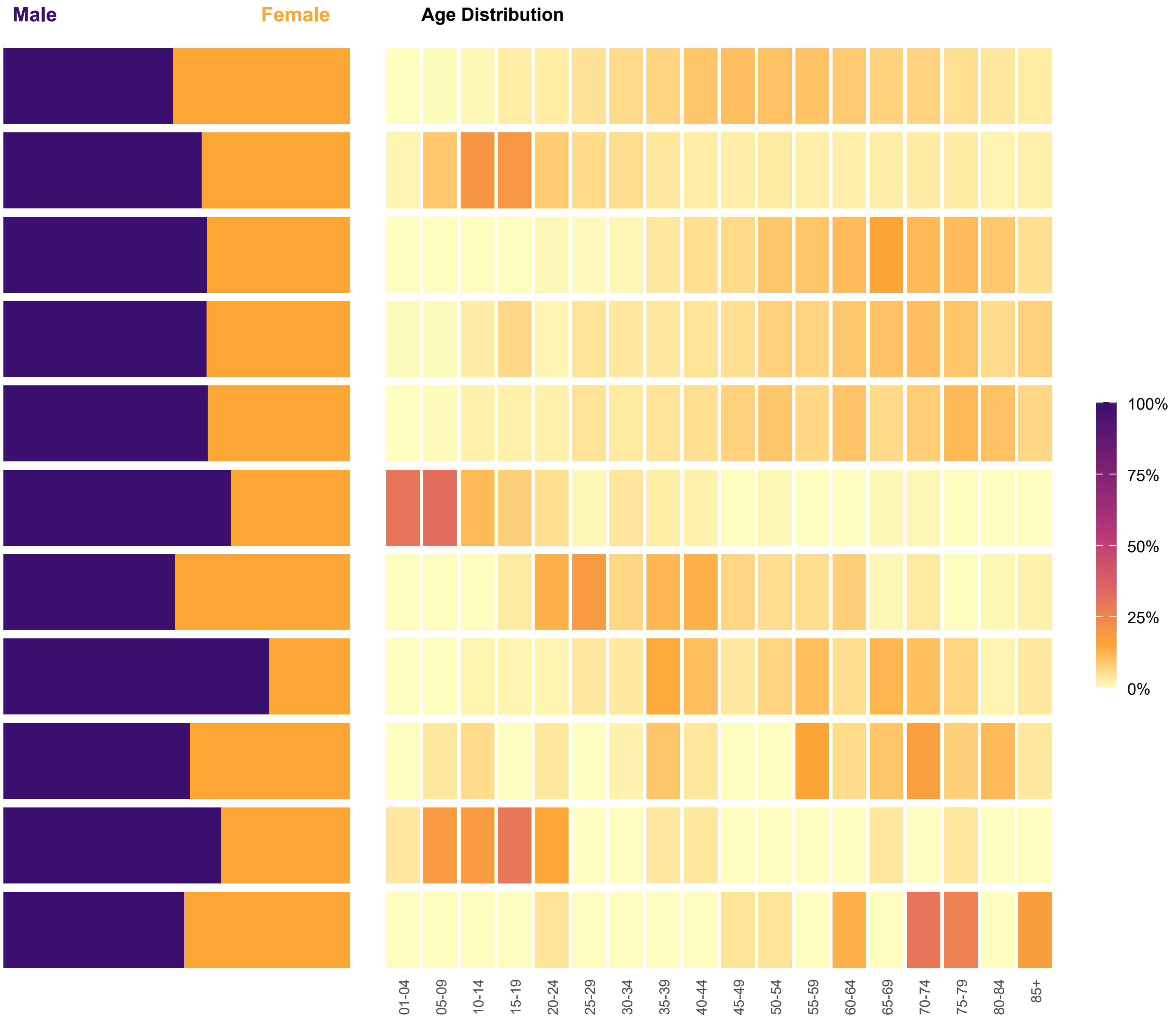

Primary Site: Lung and Bronchus

Top 25 Morphology Groups | cases: 1,844,269

Morphology Group	n	%
Adenocarcinoma	893,248	48.4%
Squamous cell carcinoma	501,819	27.2%
Neuroendocrine tumor	431,608	23.4%
Marginal zone B-cell lymphoma	3,115	0.2%
Diffuse large B-cell lymphoma	2,590	0.1%
Sarcoma	1,777	<0.1%
Carcinosarcoma	1,146	<0.1%
Lymphoma	1,108	<0.1%
Mesothelioma	1,104	<0.1%
Adenoid cystic carcinoma	795	<0.1%
Transitional cell carcinoma	502	<0.1%
Leiomyosarcoma	430	<0.1%
Synovial sarcoma	374	<0.1%
Hodgkin lymphoma	371	<0.1%
Langerhans cell histiocytosis	369	<0.1%
Follicular lymphoma	315	<0.1%
Solitary fibrous tumor	292	<0.1%
Hemangiosarcoma	275	<0.1%
Kaposi sarcoma	266	<0.1%
Plasmacytoma	241	<0.1%
Lymphoepithelial carcinoma	231	<0.1%
Epithelioid hemangioendothelioma	186	<0.1%
Pulmonary blastoma	171	<0.1%
Melanoma	145	<0.1%
T-cell lymphoma	129	<0.1%

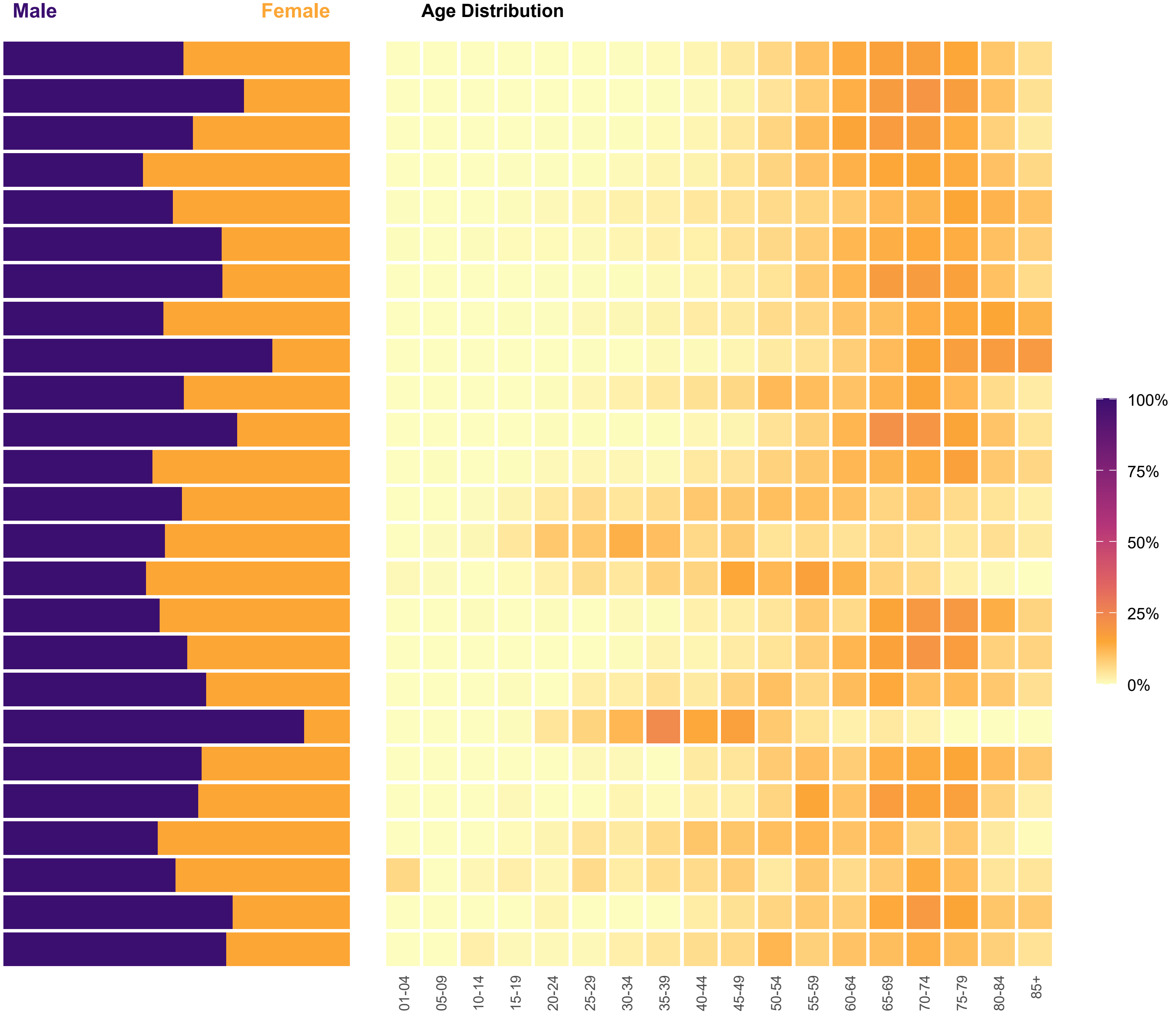

Primary Site: Major Salivary Glands

Top 25 Morphology Groups | cases: 50,258

Morphology Group	n	%
Adenocarcinoma	24,727	49.2%
Squamous cell carcinoma	10,606	21.1%
Adenoid cystic carcinoma	5,135	10.2%
Marginal zone B-cell lymphoma	2,021	4.0%
Follicular lymphoma	1,406	2.8%
Diffuse large B-cell lymphoma	1,169	2.3%
Epithelial-myoepithelial carcinoma	1,060	2.1%
Myoepithelial carcinoma	926	1.8%
Neuroendocrine tumor	920	1.8%
Lymphoma	542	1.1%
Lymphoepithelial carcinoma	342	0.7%
Sarcoma	159	0.3%
Carcinosarcoma	153	0.3%
Hodgkin lymphoma	141	0.3%
Merkel cell carcinoma	104	0.2%
Melanoma	100	0.2%
Mantle cell lymphoma	93	0.2%
Transitional cell carcinoma	59	0.1%
lymphoma non-Hodgkin	57	0.1%
Chronic lymphocytic leukemia	50	<0.1%
T-cell lymphoma	49	<0.1%
Burkitt lymphoma	48	<0.1%
Plasmacytoma	36	<0.1%
Basal cell carcinoma	33	<0.1%
Rhabdomyosarcoma	33	<0.1%

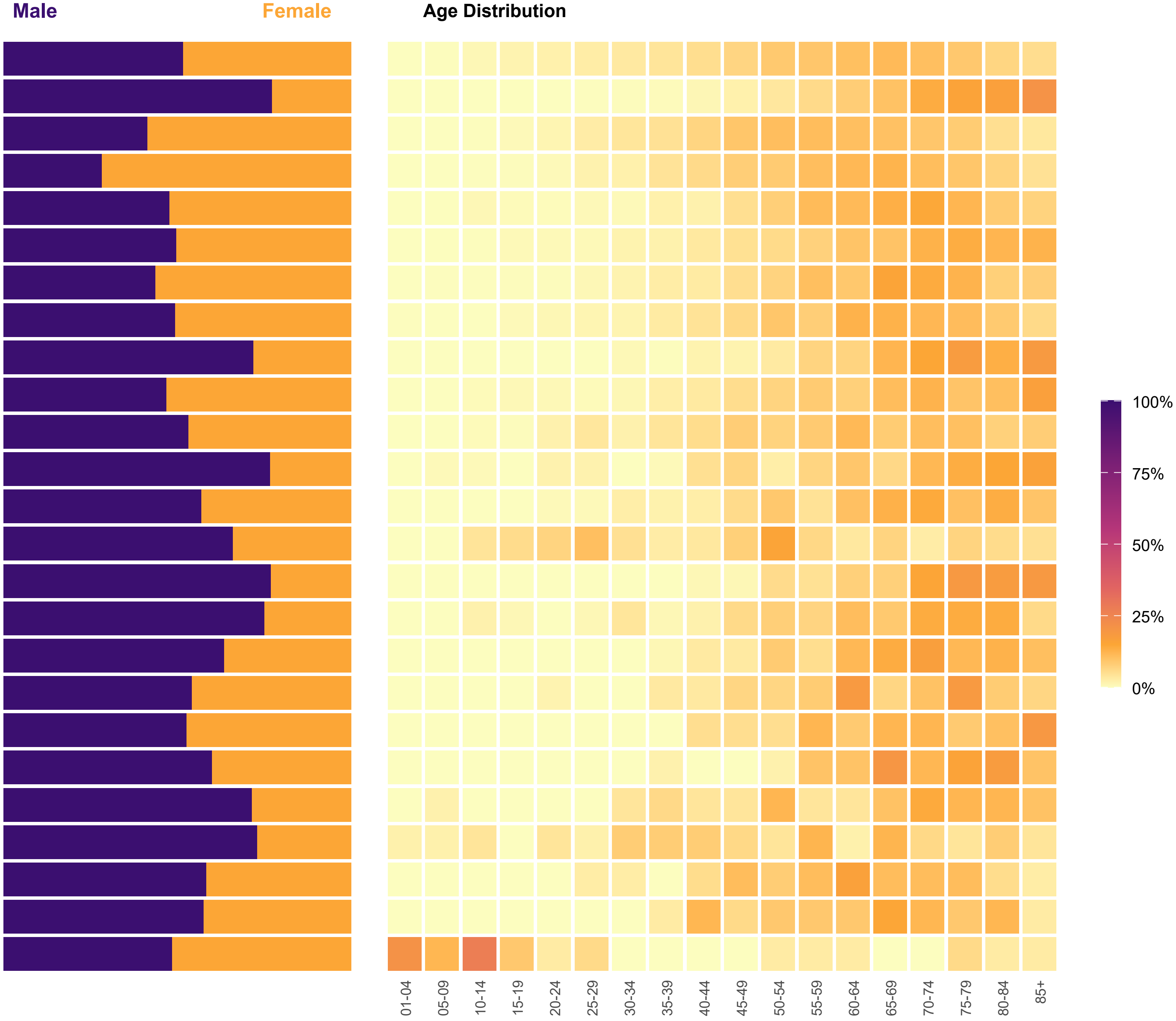

Primary Site: Mandible

Top 10 Morphology Groups | cases: 1,940

Morphology Group	n	%
Osteosarcoma	574	29.6%
Squamous cell carcinoma	330	17.0%
Ameloblastoma	241	12.4%
Odontogenic tumor	139	7.2%
Diffuse large B-cell lymphoma	138	7.1%
Plasmacytoma	96	4.9%
Sarcoma	77	4.0%
Langerhans cell histiocytosis	72	3.7%
Chondrosarcoma	65	3.4%
Lymphoma	23	1.2%

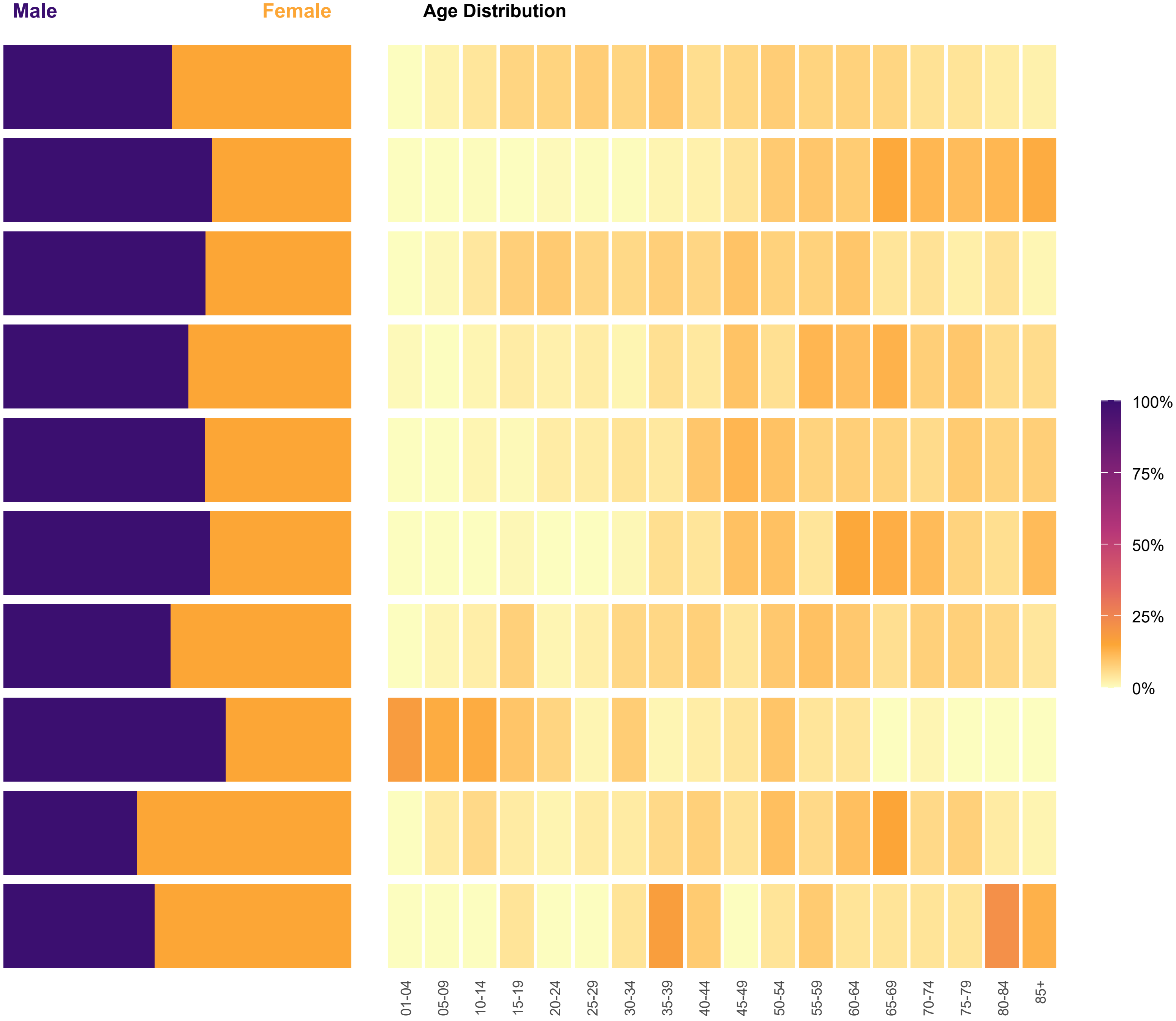

Primary Site: Meninges

Top 10 Morphology Groups | cases: 5,414

Morphology Group	n	%
Meningioma	4,789	88.5%
Ependymoma	169	3.1%
Melanoma	80	1.5%
Diffuse large B-cell lymphoma	57	1.1%
Marginal zone B-cell lymphoma	38	0.7%
Glioblastoma	33	0.6%
Lymphoma	33	0.6%
Solitary fibrous tumor	29	0.5%
Sarcoma	25	0.5%
Astrocytoma	20	0.4%

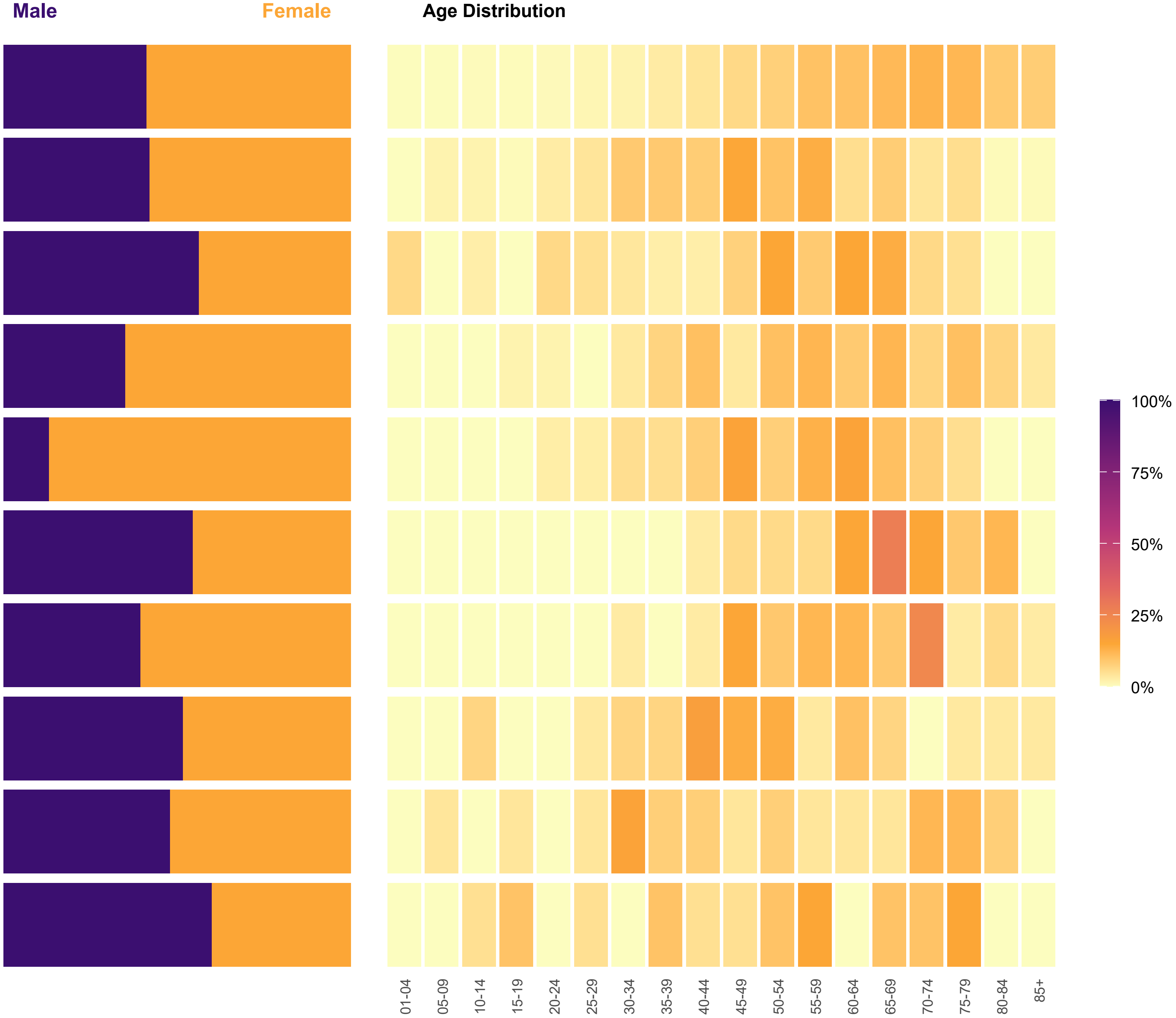

Primary Site: Mouth Other

Top 11 Morphology Groups | cases: 12,529

Morphology Group	n	%
Squamous cell carcinoma	9,371	74.8%
Adenocarcinoma	1,761	14.1%
Adenoid cystic carcinoma	578	4.6%
Diffuse large B-cell lymphoma	161	1.3%
Kaposi sarcoma	151	1.2%
Melanoma	99	0.8%
Marginal zone B-cell lymphoma	62	0.5%
Follicular lymphoma	55	0.4%
Lymphoma	53	0.4%
Myoepithelial carcinoma	49	0.4%
Plasmacytoma	30	0.2%

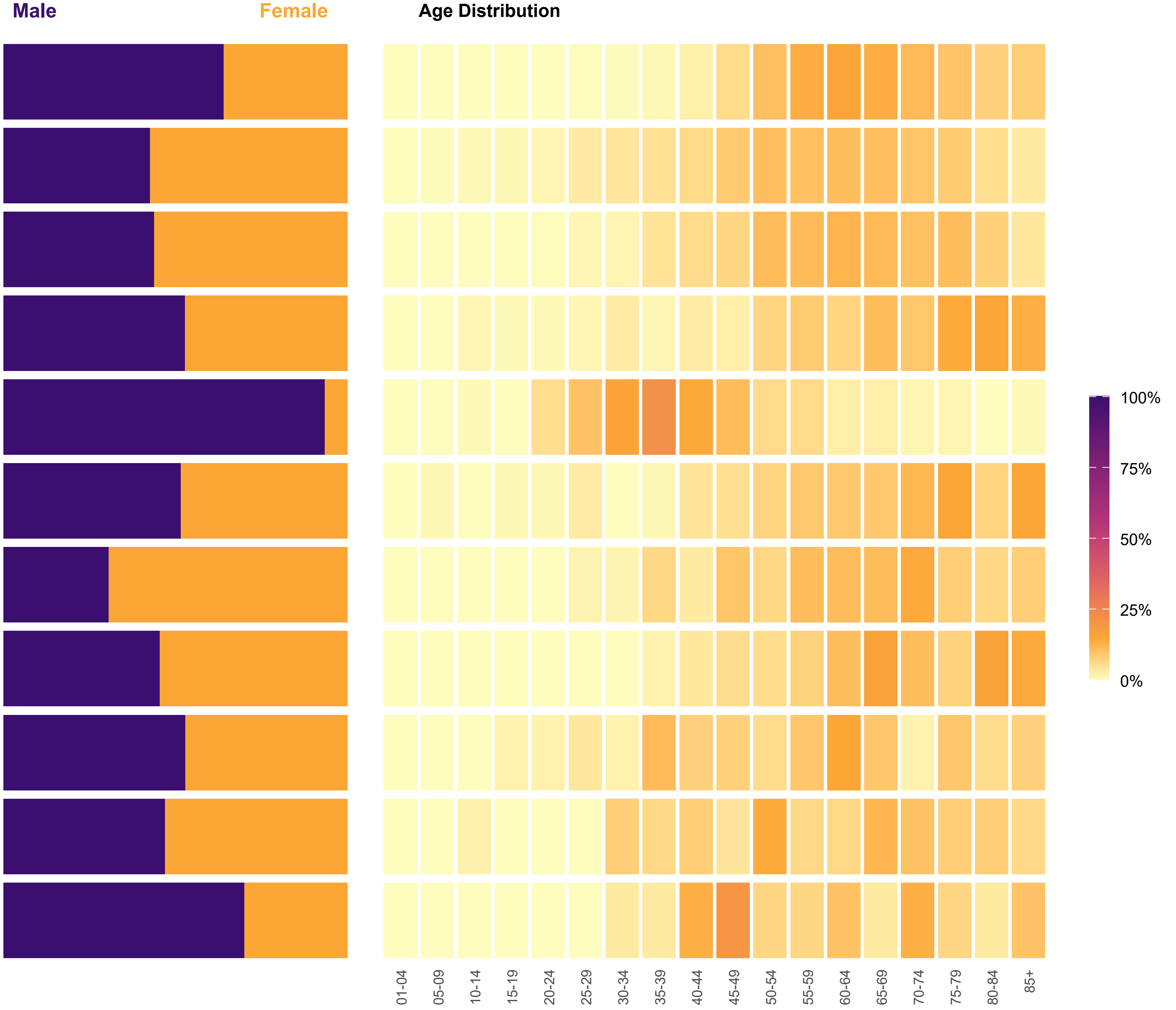

Primary Site: Nasal Cavity and Paranasal Sinuses

Top 25 Morphology Groups | cases: 28,814

Morphology Group	n	%
Squamous cell carcinoma	15,081	52.3%
Adenocarcinoma	2,344	8.1%
Melanoma	2,324	8.1%
Olfactory neurogenic tumor	1,740	6.0%
Diffuse large B-cell lymphoma	1,623	5.6%
Adenoid cystic carcinoma	1,320	4.6%
NK/T-cell lymphoma	857	3.0%
Neuroendocrine tumor	664	2.3%
Rhabdomyosarcoma	418	1.5%
Plasmacytoma	391	1.4%
Transitional cell carcinoma	274	1.0%
Sarcoma	241	0.8%
Lymphoma	226	0.8%
Chondrosarcoma	103	0.4%
Marginal zone B-cell lymphoma	96	0.3%
Basal cell carcinoma	85	0.3%
Leiomyosarcoma	80	0.3%
T-cell lymphoma	75	0.3%
Follicular lymphoma	69	0.2%
Lymphoepithelial carcinoma	58	0.2%
Epithelial-myoepithelial carcinoma	50	0.2%
Myoepithelial carcinoma	48	0.2%
Malignant peripheral nerve sheath…	48	0.2%
Burkitt lymphoma	45	0.2%
Hemangiosarcoma	44	0.2%

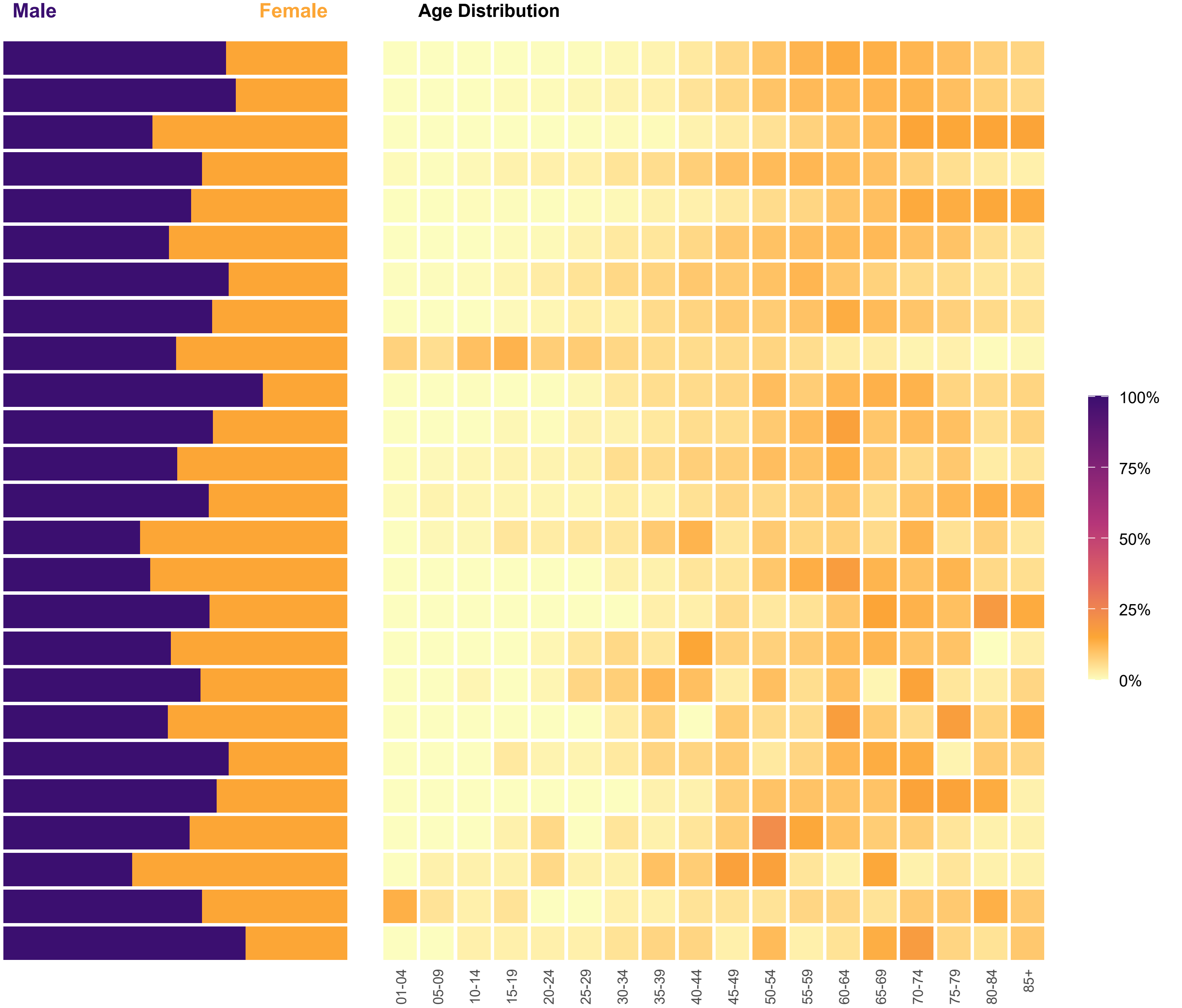

Primary Site: Nasopharynx

Top 22 Morphology Groups | cases: 20,094

Morphology Group	n	%
Squamous cell carcinoma	13,900	69.2%
Lymphoepithelial carcinoma	2,233	11.1%
Diffuse large B-cell lymphoma	1,033	5.1%
Adenocarcinoma	451	2.2%
Adenoid cystic carcinoma	334	1.7%
Neuroendocrine tumor	238	1.2%
Plasmacytoma	222	1.1%
Marginal zone B-cell lymphoma	215	1.1%
Lymphoma	207	1.0%
Follicular lymphoma	178	0.9%
Rhabdomyosarcoma	173	0.9%
Mantle cell lymphoma	149	0.7%
NK/T-cell lymphoma	149	0.7%
Burkitt lymphoma	93	0.5%
Melanoma	88	0.4%
Transitional cell carcinoma	62	0.3%
Hodgkin lymphoma	56	0.3%
T-cell lymphoma	42	0.2%
Sarcoma	39	0.2%
Chordoma	32	0.2%
Chronic lymphocytic leukemia	31	0.2%
lymphoma non-Hodgkin	25	0.1%

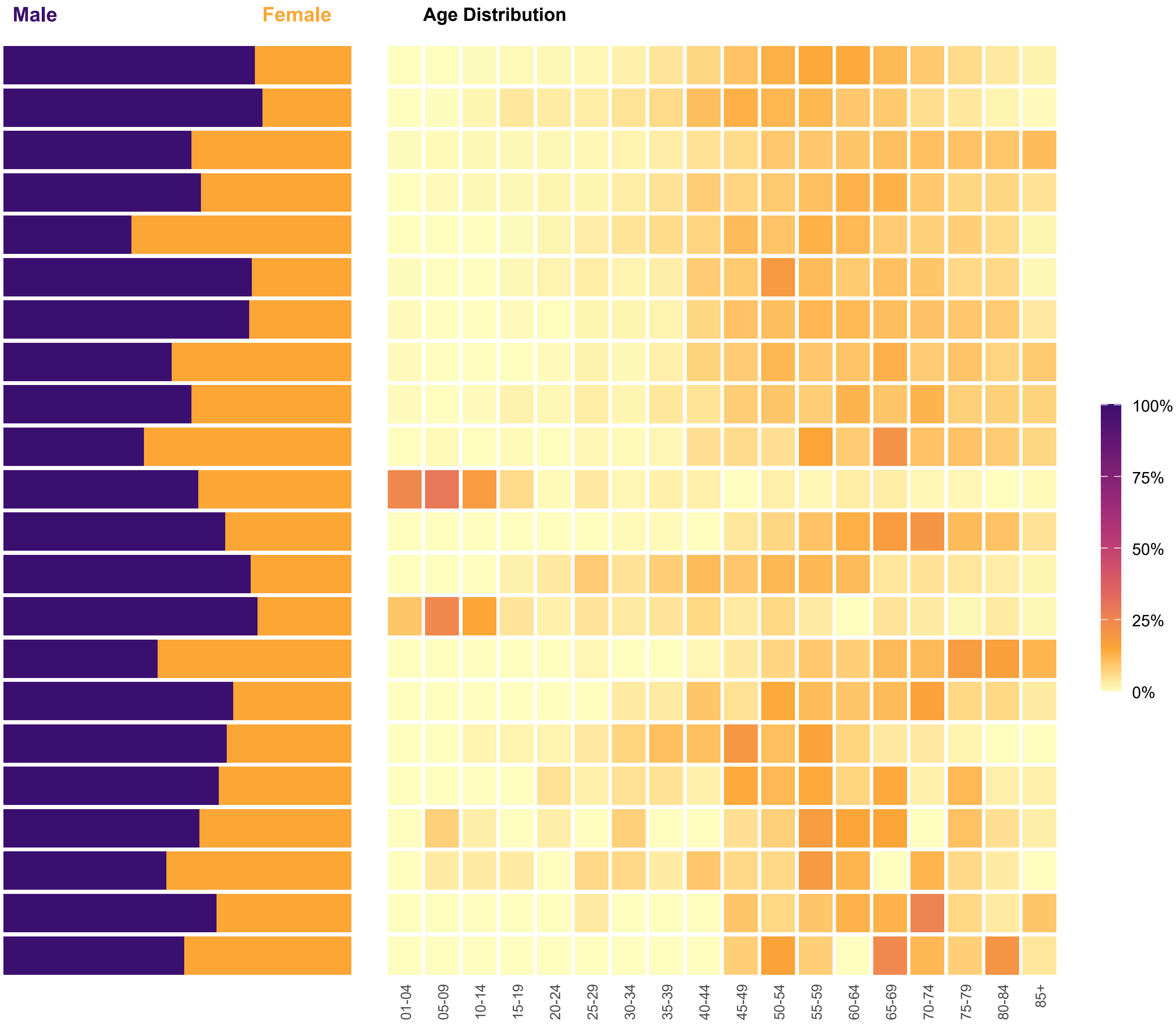

Primary Site: Oropharynx

Top 24 Morphology Groups | cases: 132,291

Morphology Group	n	%
Squamous cell carcinoma	123,055	93.0%
Diffuse large B-cell lymphoma	3,358	2.5%
Adenocarcinoma	1,519	1.1%
Follicular lymphoma	732	0.6%
Lymphoepithelial carcinoma	493	0.4%
Mantle cell lymphoma	432	0.3%
Lymphoma	425	0.3%
Adenoid cystic carcinoma	424	0.3%
Neuroendocrine tumor	258	0.2%
Marginal zone B-cell lymphoma	248	0.2%
Plasmacytoma	178	0.1%
Burkitt lymphoma	163	0.1%
Kaposi sarcoma	133	0.1%
T-cell lymphoma	132	<0.1%
Transitional cell carcinoma	78	<0.1%
Hodgkin lymphoma	77	<0.1%
Chronic lymphocytic leukemia	76	<0.1%
lymphoma non-Hodgkin	67	<0.1%
Sarcoma	64	<0.1%
Melanoma	59	<0.1%
Rhabdomyosarcoma	50	<0.1%
NK/T-cell lymphoma	45	<0.1%
Myoepithelial carcinoma	33	<0.1%
Carcinosarcoma	20	<0.1%

Male **Female**

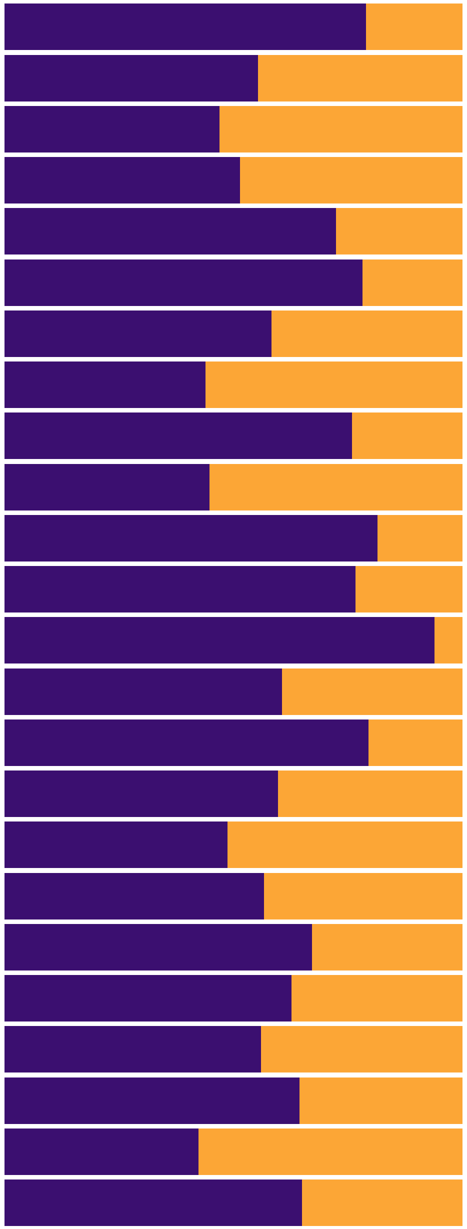

Age Distribution

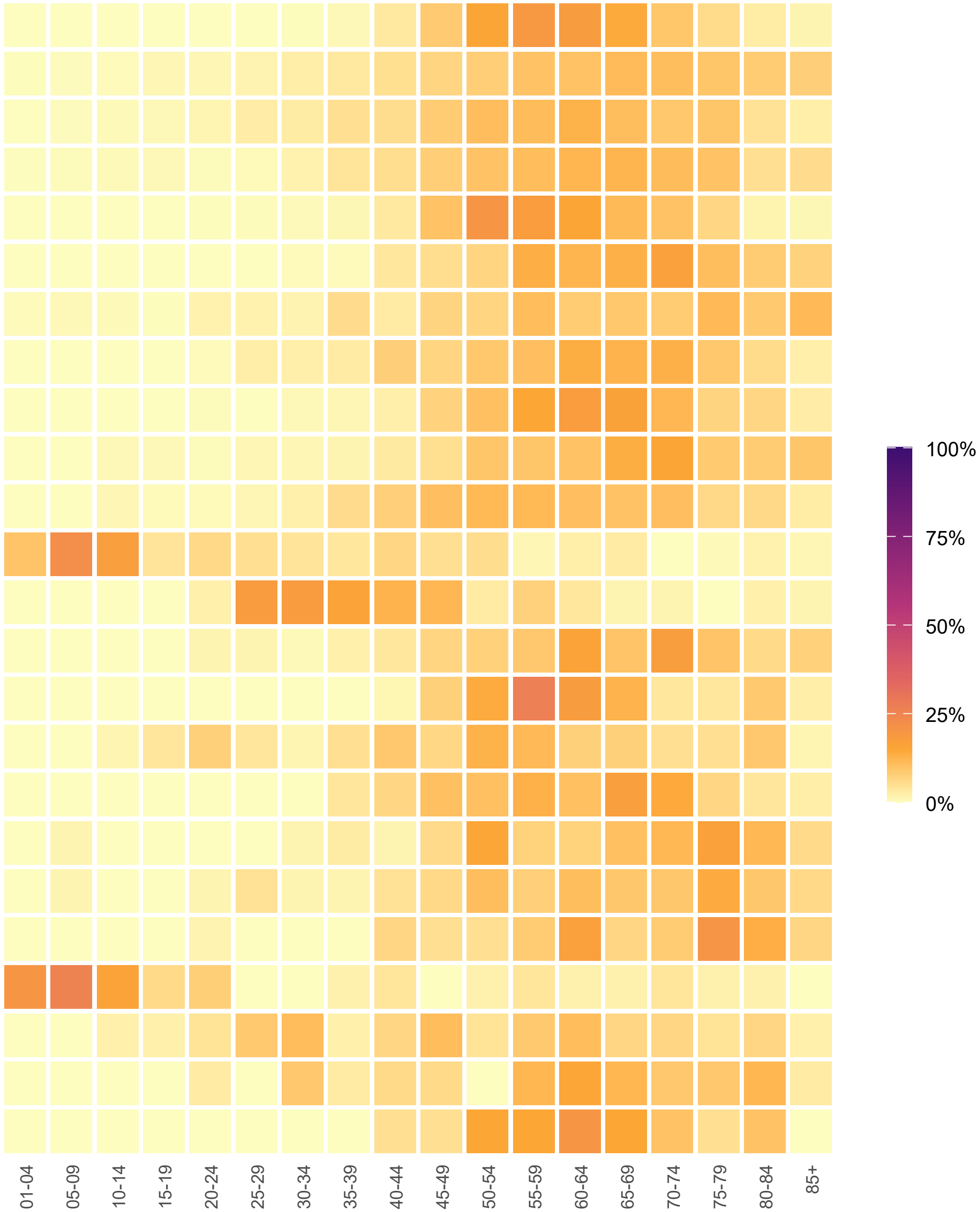

Primary Site: Ovary

Top 25 Morphology Groups | cases: 257,478

Morphology Group	n	%
Adenocarcinoma	232,941	90.5%
germ cell tumor	4,616	1.8%
Carcinosarcoma	3,144	1.2%
Mullerian mixed tumor	2,944	1.1%
Neuroendocrine tumor	2,341	0.9%
Teratoma	2,326	0.9%
Dysgerminoma	1,737	0.7%
Squamous cell carcinoma	921	0.4%
Yolk sac tumor	871	0.3%
Mixed germ cell tumor	709	0.3%
Transitional cell carcinoma	646	0.3%
malignant Brenner tumor	599	0.2%
Mesodermal mixed tumor	572	0.2%
Sertoli-Leydig cell tumor	429	0.2%
Leiomyosarcoma	295	0.1%
Sarcoma	236	<0.1%
Germinoma	225	<0.1%
Diffuse large B-cell lymphoma	212	<0.1%
Serous adenocarcinofibroma	209	<0.1%
Fibrosarcoma	149	<0.1%
Steroid cell tumor malignant	124	<0.1%
Adenosarcoma	120	<0.1%
Choriocarcinoma	120	<0.1%
Granular cell carcinoma	77	<0.1%
Endometrial stromal sarcoma	75	<0.1%

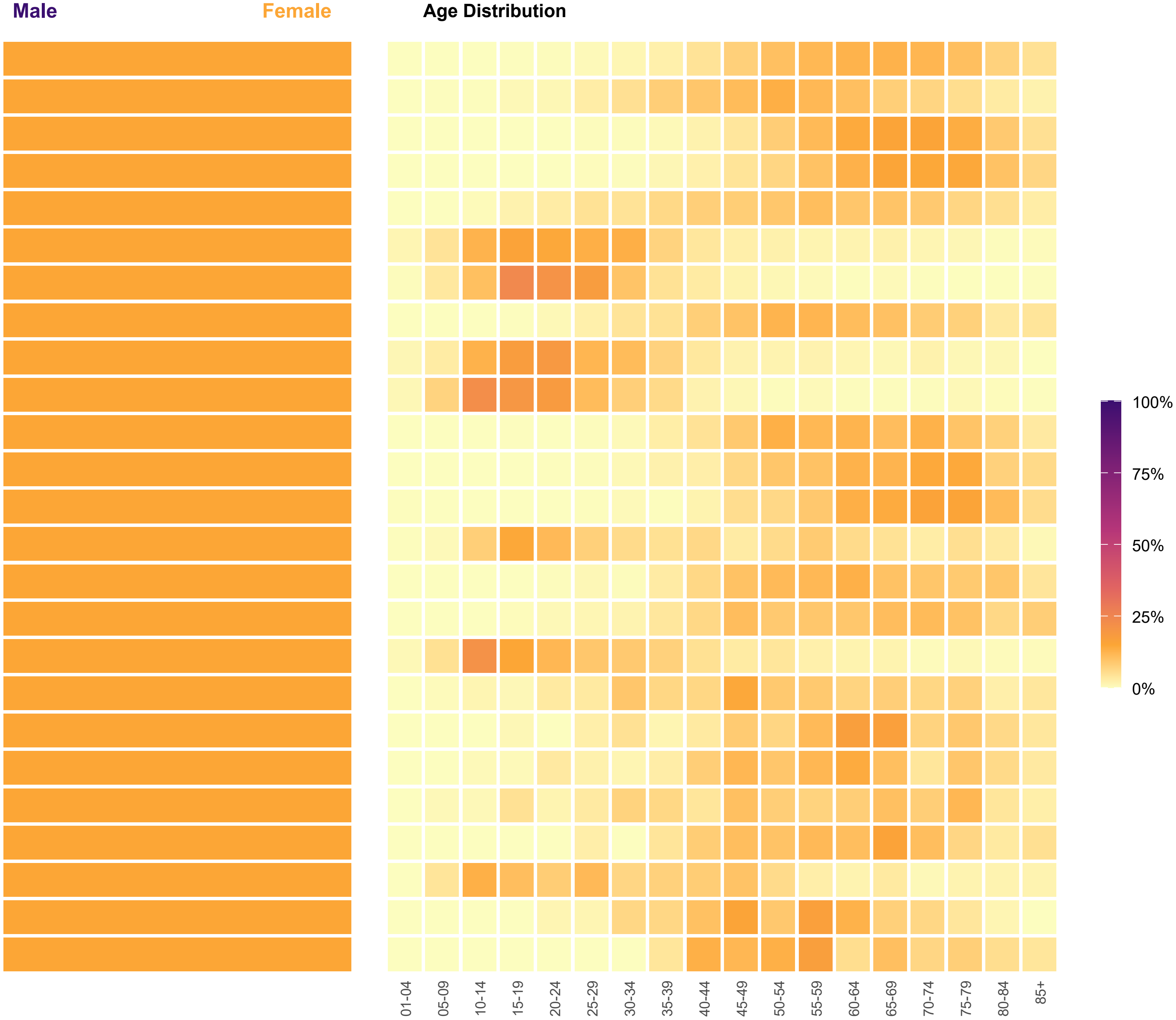

Primary Site: Palate excluding Soft and Uvula

Top 12 Morphology Groups | cases: 6,441

Morphology Group	n	%
Squamous cell carcinoma	3,055	47.4%
Adenocarcinoma	1,867	29.0%
Adenoid cystic carcinoma	686	10.7%
Melanoma	161	2.5%
Kaposi sarcoma	143	2.2%
Diffuse large B-cell lymphoma	130	2.0%
Follicular lymphoma	81	1.3%
Marginal zone B-cell lymphoma	61	0.9%
Myoepithelial carcinoma	49	0.8%
Lymphoma	45	0.7%
Epithelial-myoepithelial carcinoma	25	0.4%
Mantle cell lymphoma	23	0.4%

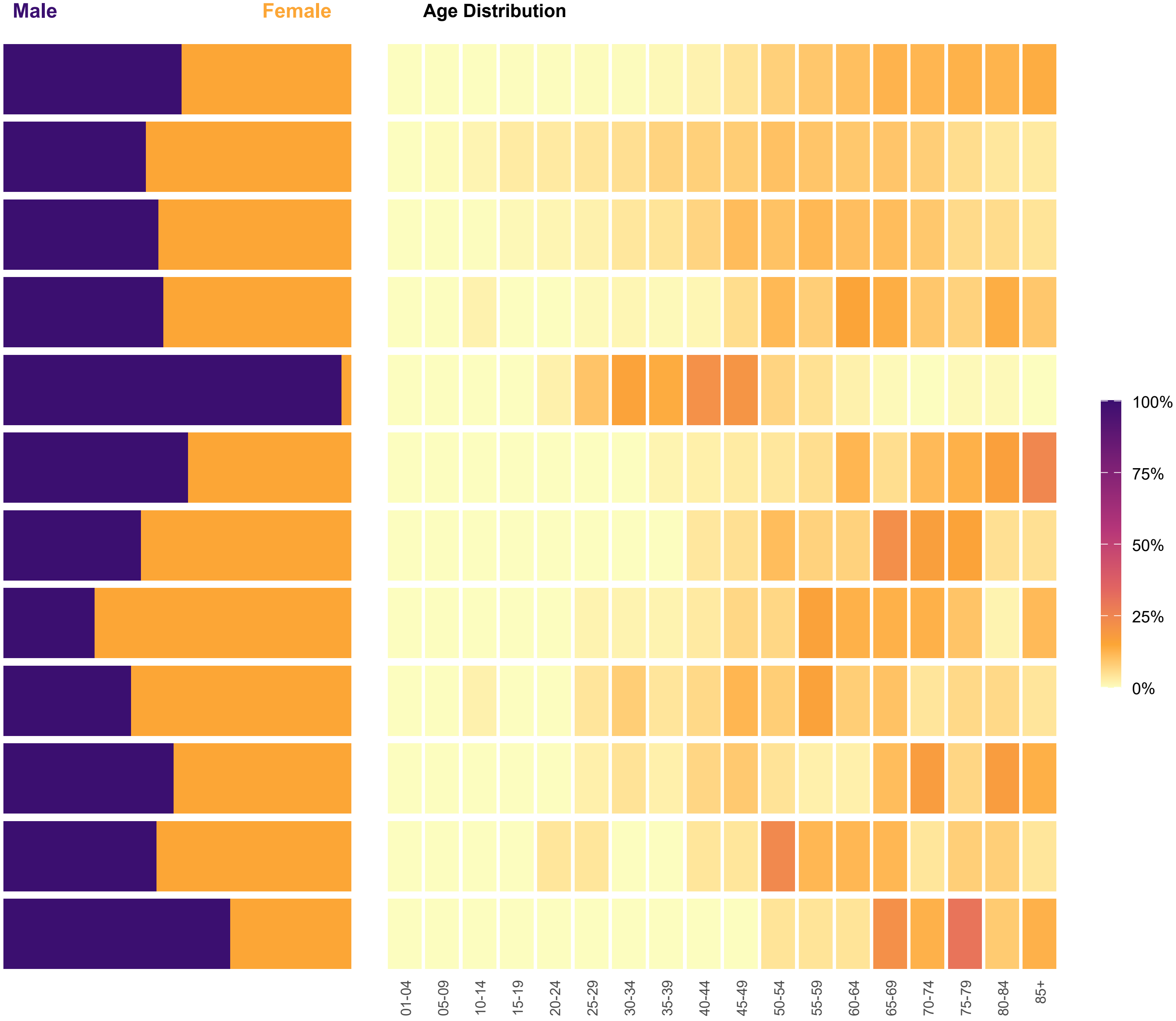

Primary Site: Pancreas

Top 19 Morphology Groups | cases: 427,934

Morphology Group	n	%
Adenocarcinoma	389,986	91.1%
Neuroendocrine tumor	33,522	7.8%
Solid pseudopapillary neoplasm of…	1,060	0.2%
Squamous cell carcinoma	995	0.2%
Diffuse large B-cell lymphoma	853	0.2%
Lymphoma	257	<0.1%
Follicular lymphoma	204	<0.1%
Sarcoma	155	<0.1%
Leiomyosarcoma	140	<0.1%
Gastrointestinal stromal tumor	113	<0.1%
Pancreatoblastoma	107	<0.1%
Carcinosarcoma	79	<0.1%
Hepatocellular carcinoma	35	<0.1%
Burkitt lymphoma	31	<0.1%
CNS embryonal tumor	27	<0.1%
Adenoid cystic carcinoma	26	<0.1%
Marginal zone B-cell lymphoma	25	<0.1%
Liposarcoma	22	<0.1%
Ewing sarcoma	22	<0.1%

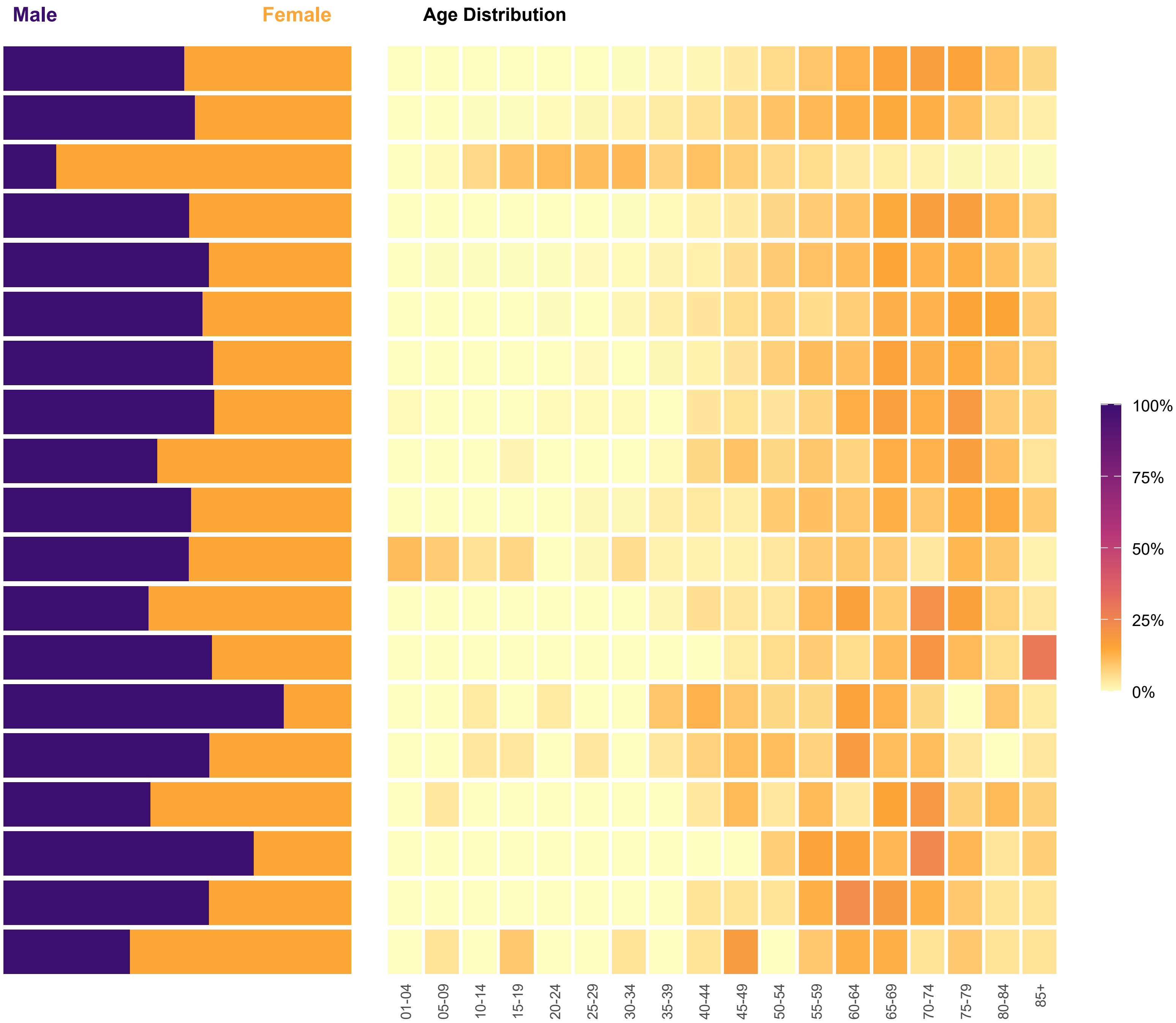

Primary Site: Parathyroid gland

Top 1 Morphology Groups | cases: 193

Morphology Group	n	%	Male	Female	Age Distribution
Adenocarcinoma	167	86.5%			

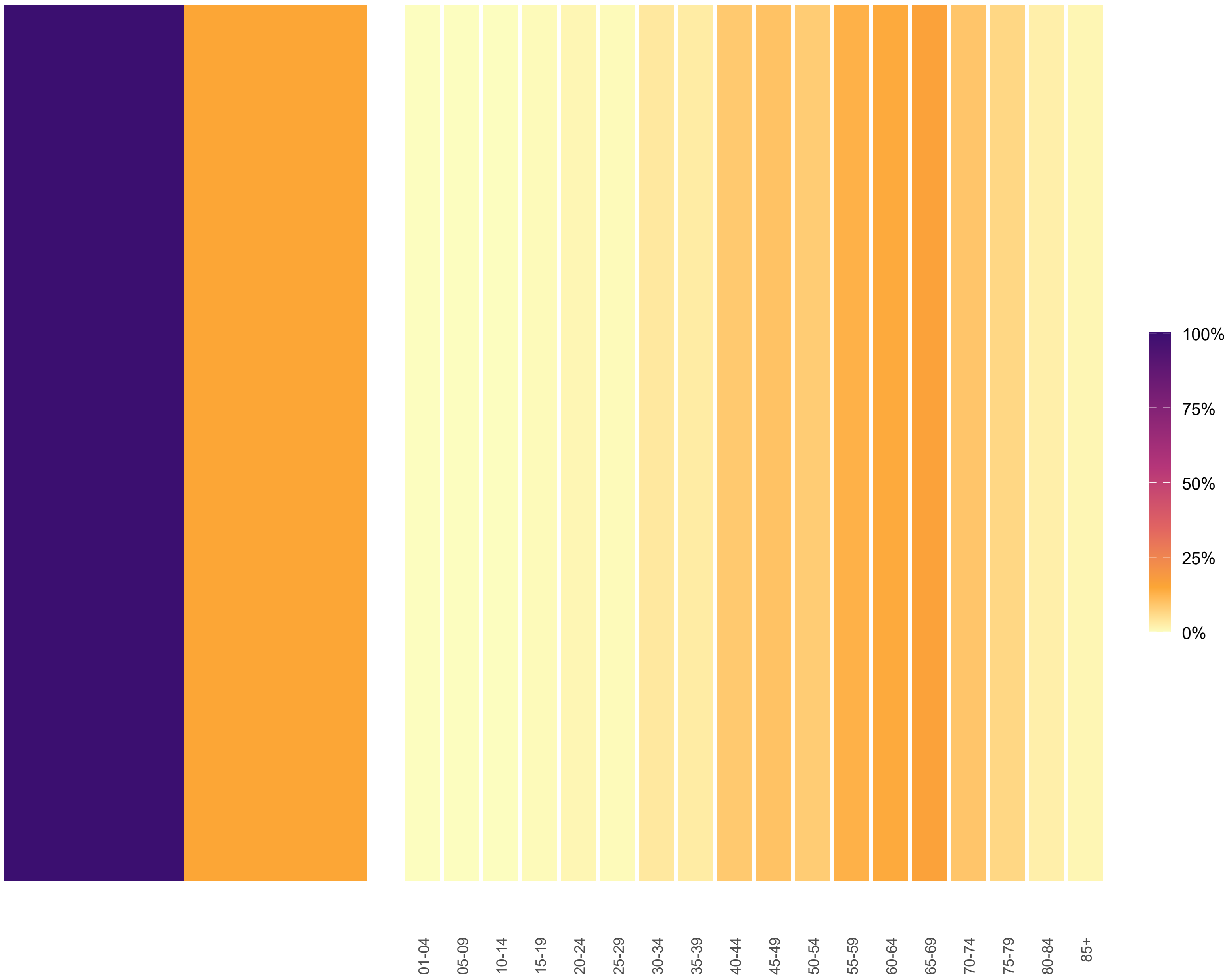

Primary Site: Pelvic bones and sacrum and coccyx and associated joints and pelvic bones

Top 18 Morphology Groups | cases: 6,985

Morphology Group	n	%
Chondrosarcoma	1,807	25.9%
Chordoma	1,237	17.7%
Osteosarcoma	1,119	16.0%
Plasmacytoma	982	14.1%
Diffuse large B-cell lymphoma	457	6.5%
Sarcoma	428	6.1%
Langerhans cell histiocytosis	113	1.6%
Lymphoma	111	1.6%
malignant giant cell tumor of bone	99	1.4%
Myxoid chondrosarcoma	88	1.3%
Ewing sarcoma	69	1.0%
Hemangiosarcoma	55	0.8%
Follicular lymphoma	52	0.7%
Leiomyosarcoma	32	0.5%
Undifferentiated sarcoma	29	0.4%
Fibrosarcoma	25	0.4%
Synovial sarcoma	22	0.3%
CNS embryonal tumor	20	0.3%

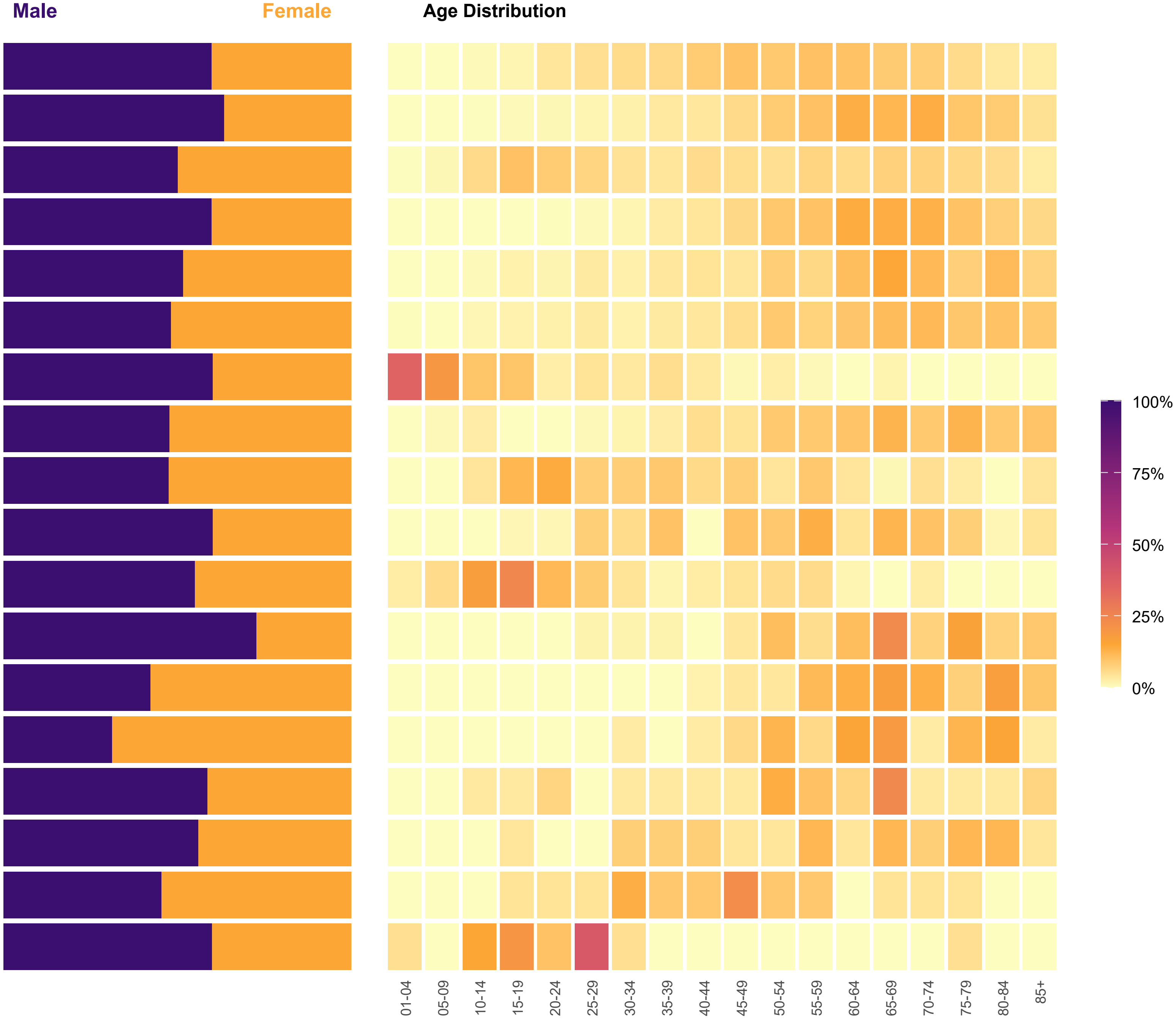

Primary Site: Penis

Top 8 Morphology Groups | cases: 21,301

Morphology Group	n	%
Squamous cell carcinoma	20,211	94.9%
Melanoma	254	1.2%
Adenocarcinoma	250	1.2%
Basal cell carcinoma	184	0.9%
Kaposi sarcoma	139	0.7%
Transitional cell carcinoma	56	0.3%
Leiomyosarcoma	45	0.2%
Sarcoma	43	0.2%

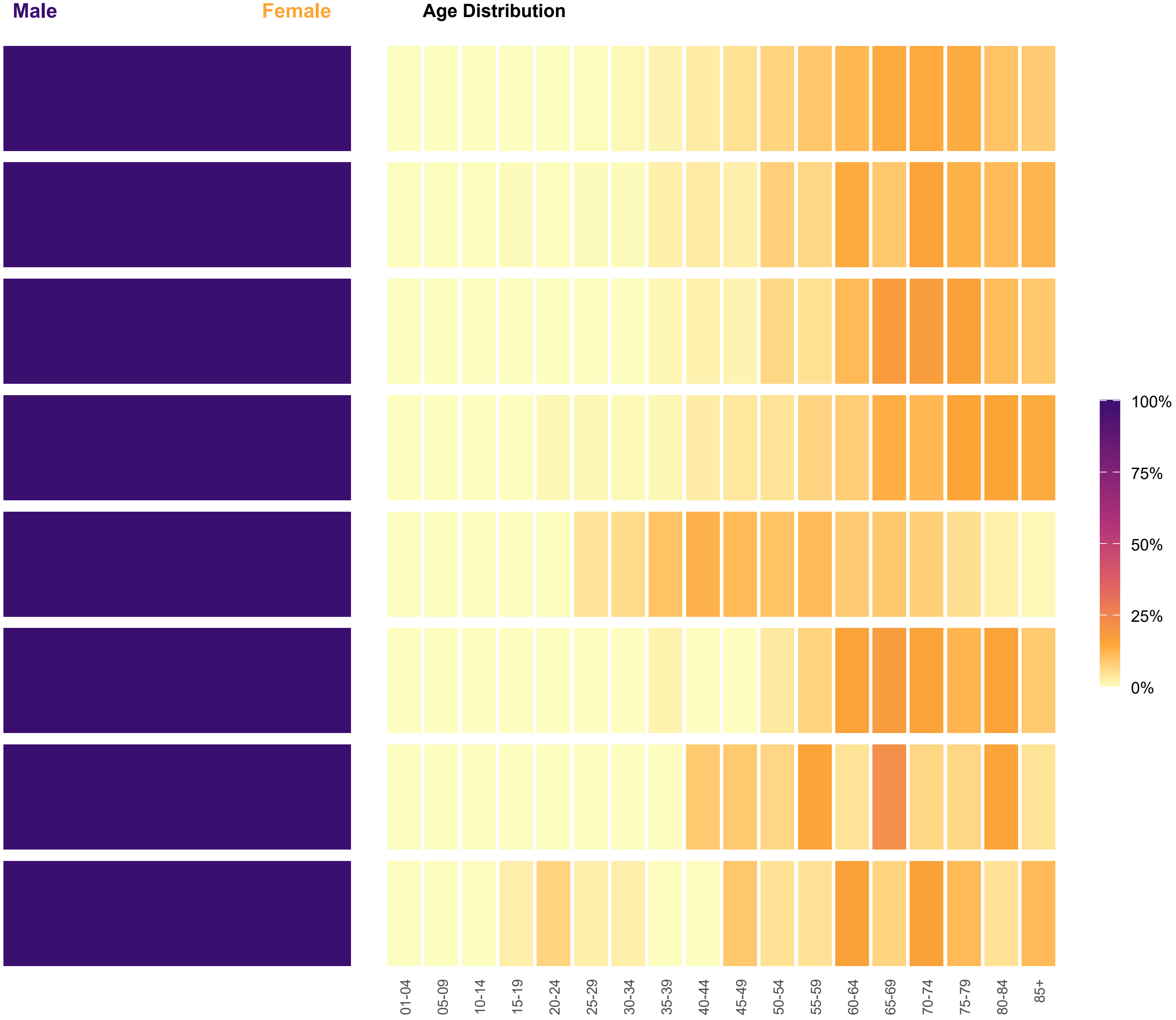

Primary Site: Peripheral nerves and autonomic nervous system

Top 25 Morphology Groups | cases: 140,477

Morphology Group	n	%
Sarcoma	39,801	28.3%
Leiomyosarcoma	16,792	12.0%
Liposarcoma	15,238	10.8%
Myxofibrosarcoma	6,990	5.0%
Synovial sarcoma	5,871	4.2%
Myxoid liposarcoma	5,761	4.1%
Malignant peripheral nerve sheath…	5,562	4.0%
Hemangiosarcoma	5,201	3.7%
Rhabdomyosarcoma	5,032	3.6%
Dermatofibrosarcoma protuberans	3,559	2.5%
Dedifferentiated liposarcoma	3,470	2.5%
Diffuse large B-cell lymphoma	2,865	2.0%
Fibrosarcoma	2,484	1.8%
Undifferentiated sarcoma	2,187	1.6%
Kaposi sarcoma	1,358	1.0%
Neuroblastoma	1,281	0.9%
Myxosarcoma	908	0.6%
Myxoid chondrosarcoma	874	0.6%
Lymphoma	827	0.6%
Clear cell sarcoma	783	0.6%
Follicular lymphoma	775	0.6%
Chondrosarcoma	770	0.5%
Squamous cell carcinoma	703	0.5%
Ewing sarcoma	670	0.5%
Solitary fibrous tumor	663	0.5%

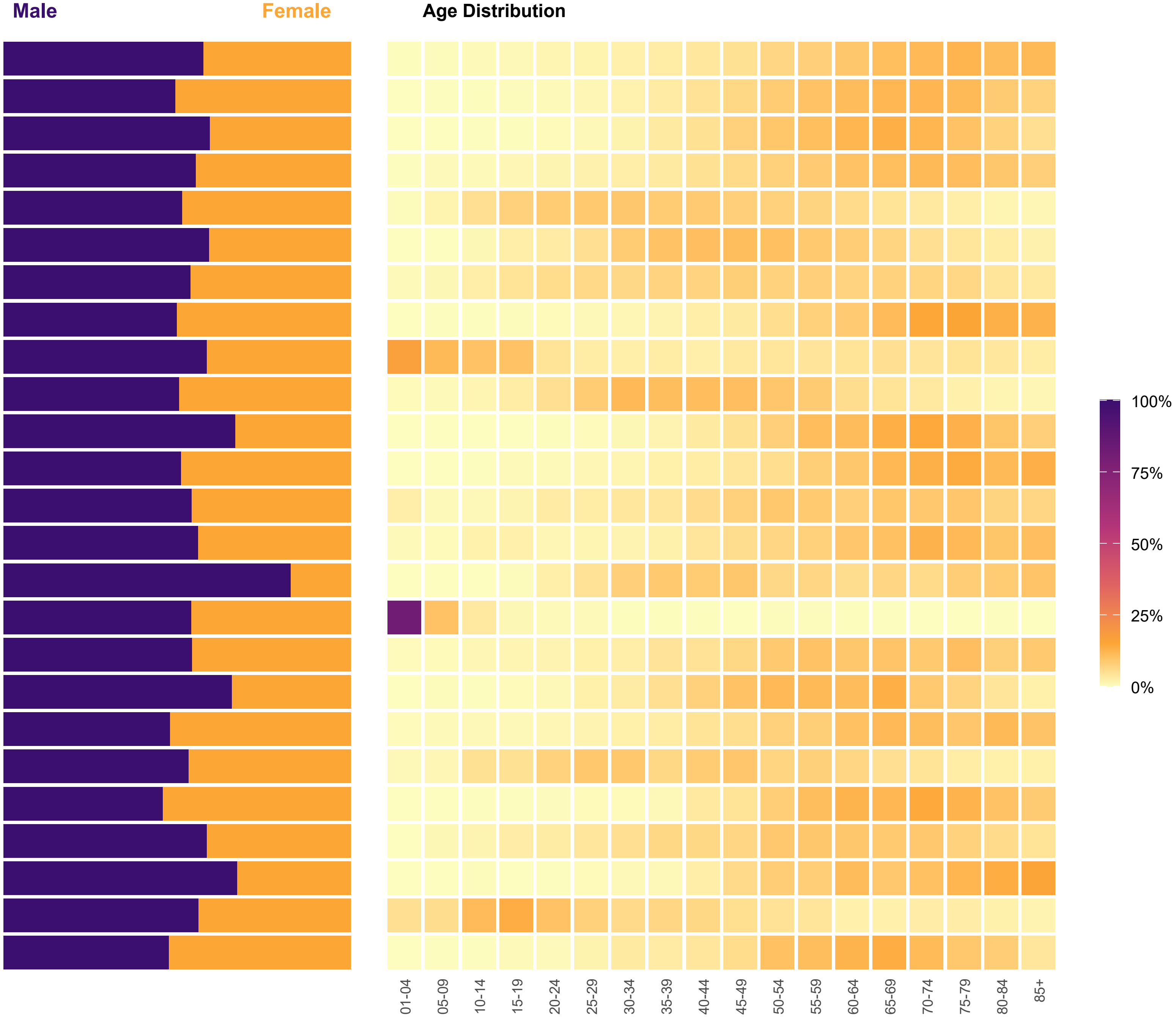

Primary Site: Pharynx and Oral Cavity Other

Top 11 Morphology Groups | cases: 9,950

Morphology Group	n	%
Squamous cell carcinoma	9,066	91.1%
Diffuse large B-cell lymphoma	229	2.3%
Adenocarcinoma	151	1.5%
Lymphoma	57	0.6%
Lymphoepithelial carcinoma	46	0.5%
Adenoid cystic carcinoma	44	0.4%
Plasmacytoma	36	0.4%
Follicular lymphoma	35	0.4%
Neuroendocrine tumor	33	0.3%
Mantle cell lymphoma	33	0.3%
Marginal zone B-cell lymphoma	32	0.3%

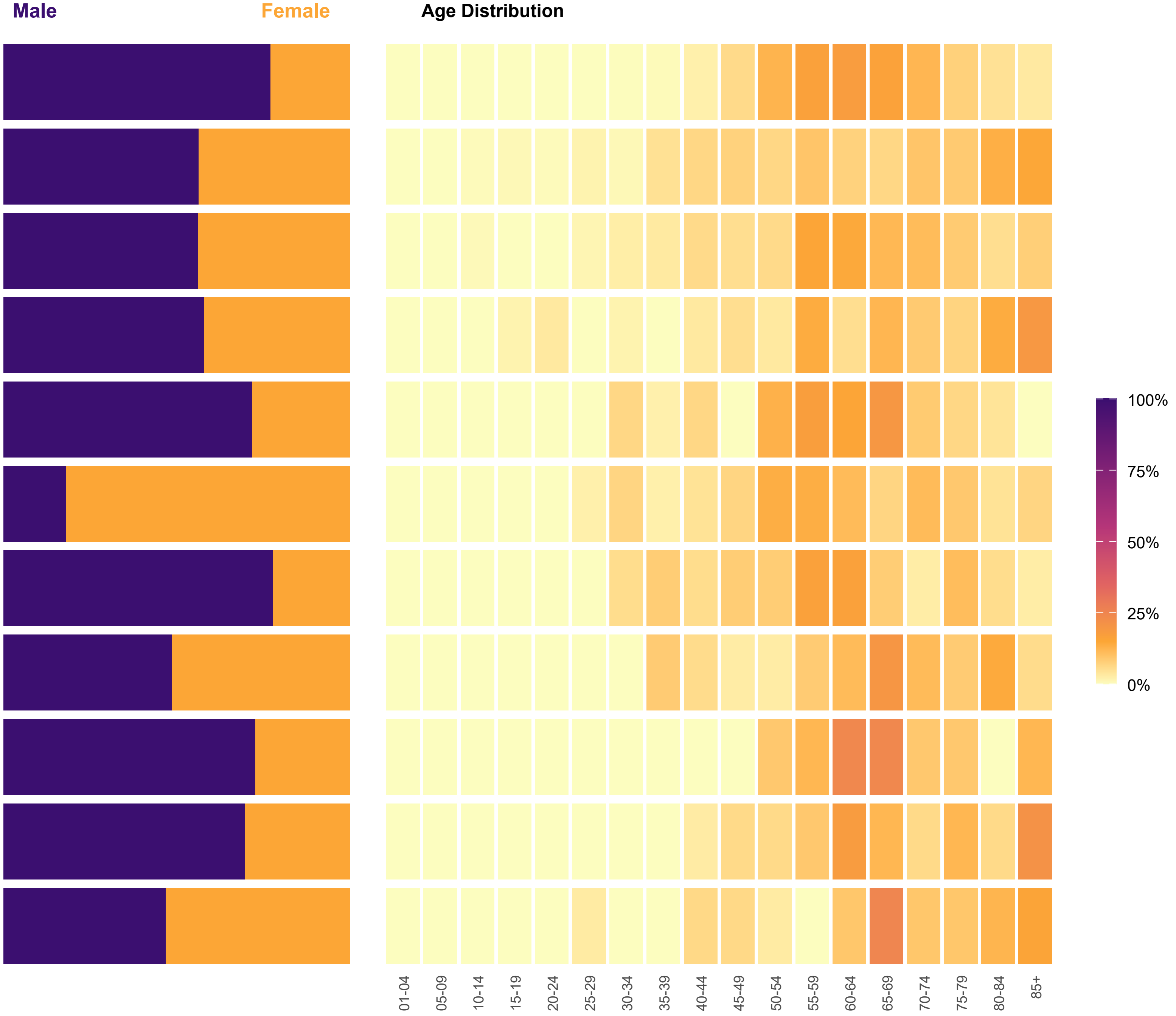

Primary Site: Placenta

Top 2 Morphology Groups | cases: 1,382

Morphology Group	n	%
Choriocarcinoma	1,237	89.5%
Trophoblastic tumor	128	9.3%

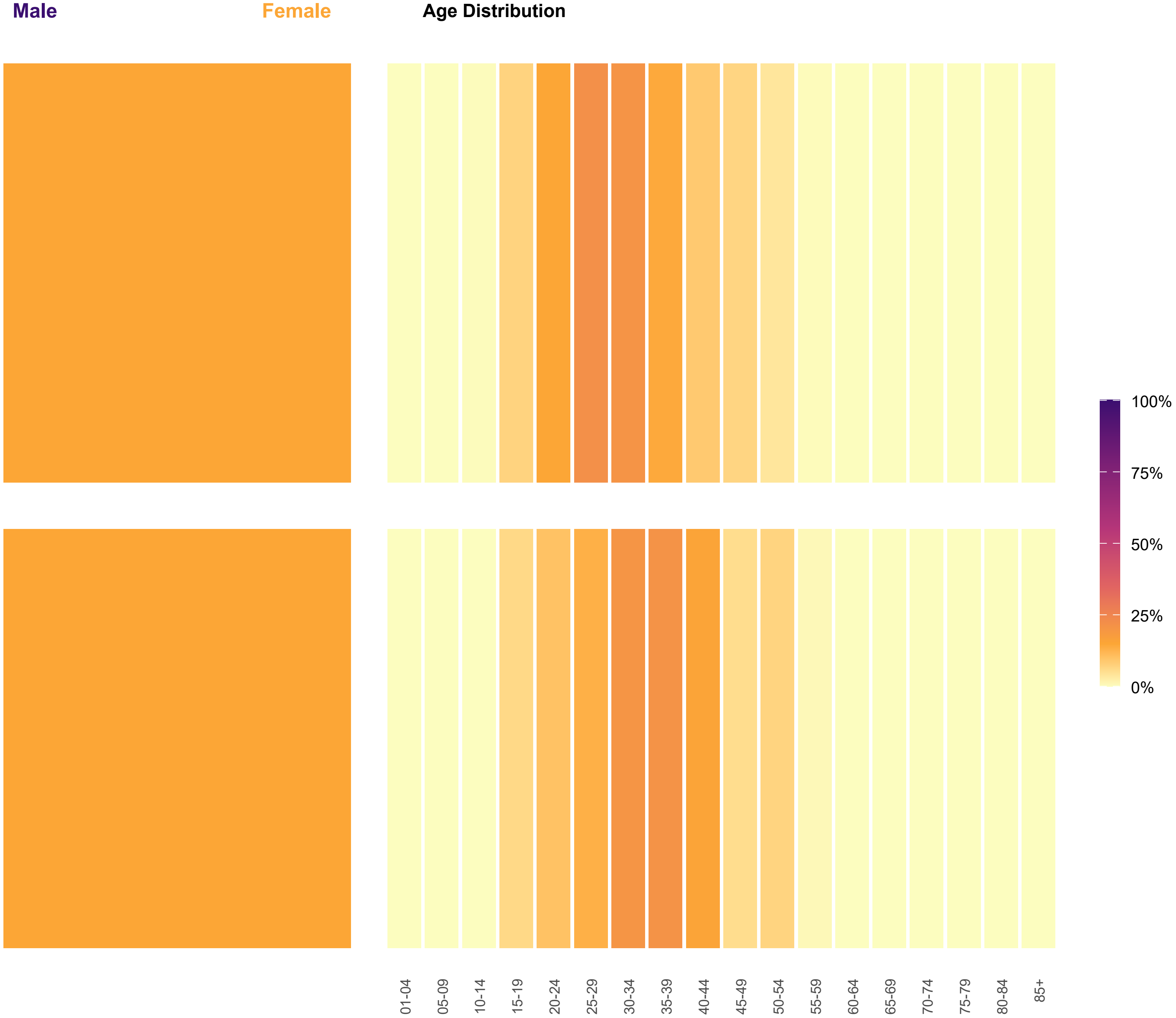

Primary Site: Prostate

Top 15 Morphology Groups | cases: 2,596,307

Morphology Group	n	%
Adenocarcinoma	2,590,939	99.8%
Neuroendocrine tumor	3,019	0.1%
Transitional cell carcinoma	1,123	<0.1%
Squamous cell carcinoma	320	<0.1%
Leiomyosarcoma	134	<0.1%
Sarcoma	124	<0.1%
Rhabdomyosarcoma	109	<0.1%
Carcinosarcoma	86	<0.1%
Diffuse large B-cell lymphoma	77	<0.1%
Marginal zone B-cell lymphoma	54	<0.1%
Lymphoma	41	<0.1%
Chronic lymphocytic leukemia	38	<0.1%
lymphoma non-Hodgkin	25	<0.1%
Basal cell carcinoma	23	<0.1%
Follicular lymphoma	22	<0.1%

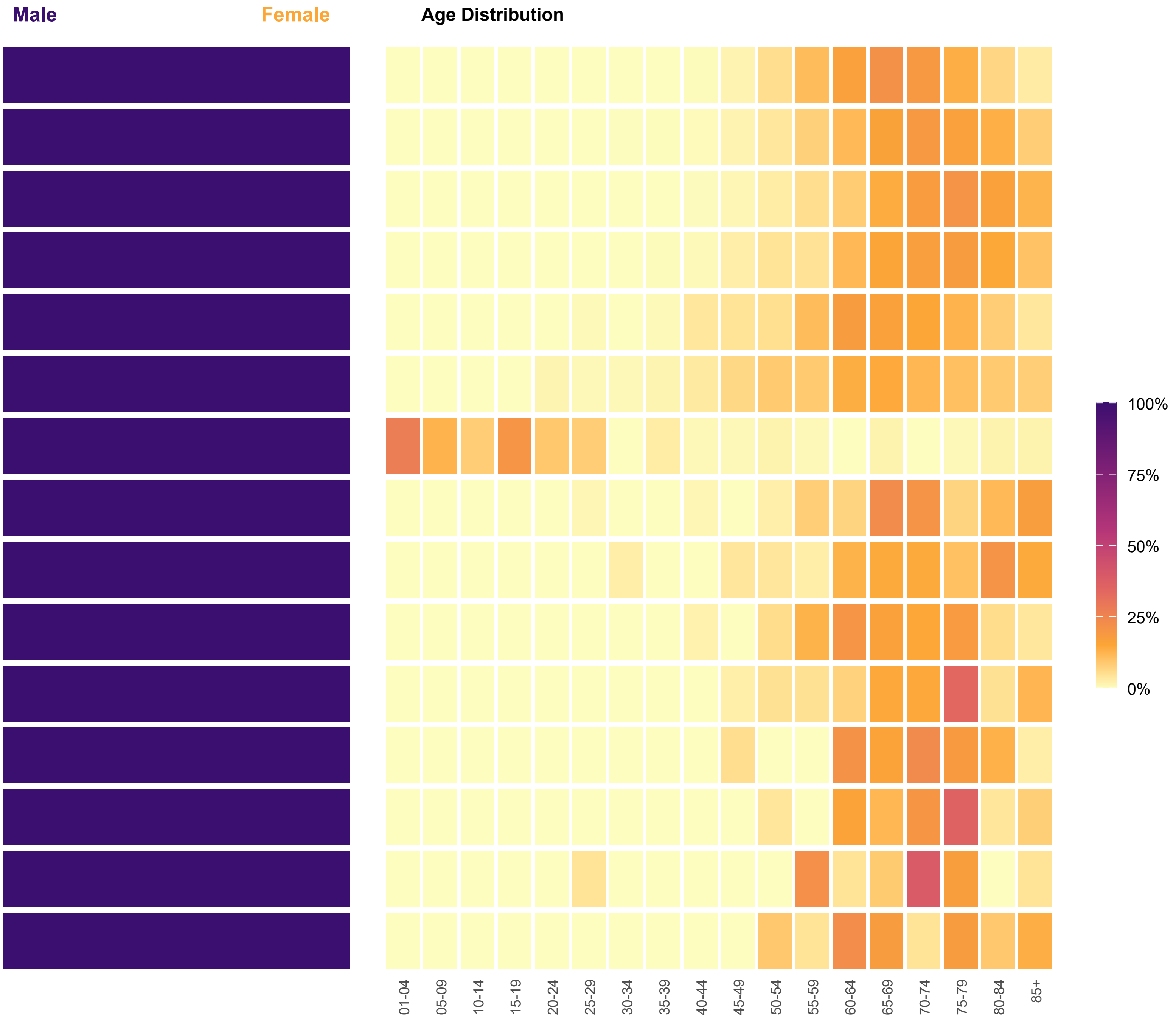

Primary Site: Retroperitoneum and Peritoneum

Top 25 Morphology Groups | cases: 40,686

Morphology Group	n	%
Adenocarcinoma	16,062	39.5%
Mesothelioma	4,802	11.8%
Liposarcoma	4,148	10.2%
Leiomyosarcoma	3,552	8.7%
Dedifferentiated liposarcoma	2,717	6.7%
Sarcoma	2,205	5.4%
Diffuse large B-cell lymphoma	836	2.1%
Neuroendocrine tumor	791	1.9%
Gastrointestinal stromal tumor	656	1.6%
Follicular lymphoma	446	1.1%
Myxoid liposarcoma	417	1.0%
Neuroblastoma	417	1.0%
Lymphoma	334	0.8%
Mullerian mixed tumor	271	0.7%
Rhabdomyosarcoma	245	0.6%
Malignant peripheral nerve sheath…	201	0.5%
Carcinosarcoma	181	0.4%
Paraganglioma	174	0.4%
Seminoma	152	0.4%
Desmoplastic small round cell tumor	147	0.4%
Ganglioneuroblastoma	144	0.4%
Germinoma	127	0.3%
Hemangiosarcoma	110	0.3%
Solitary fibrous tumor	109	0.3%
Undifferentiated sarcoma	101	0.2%

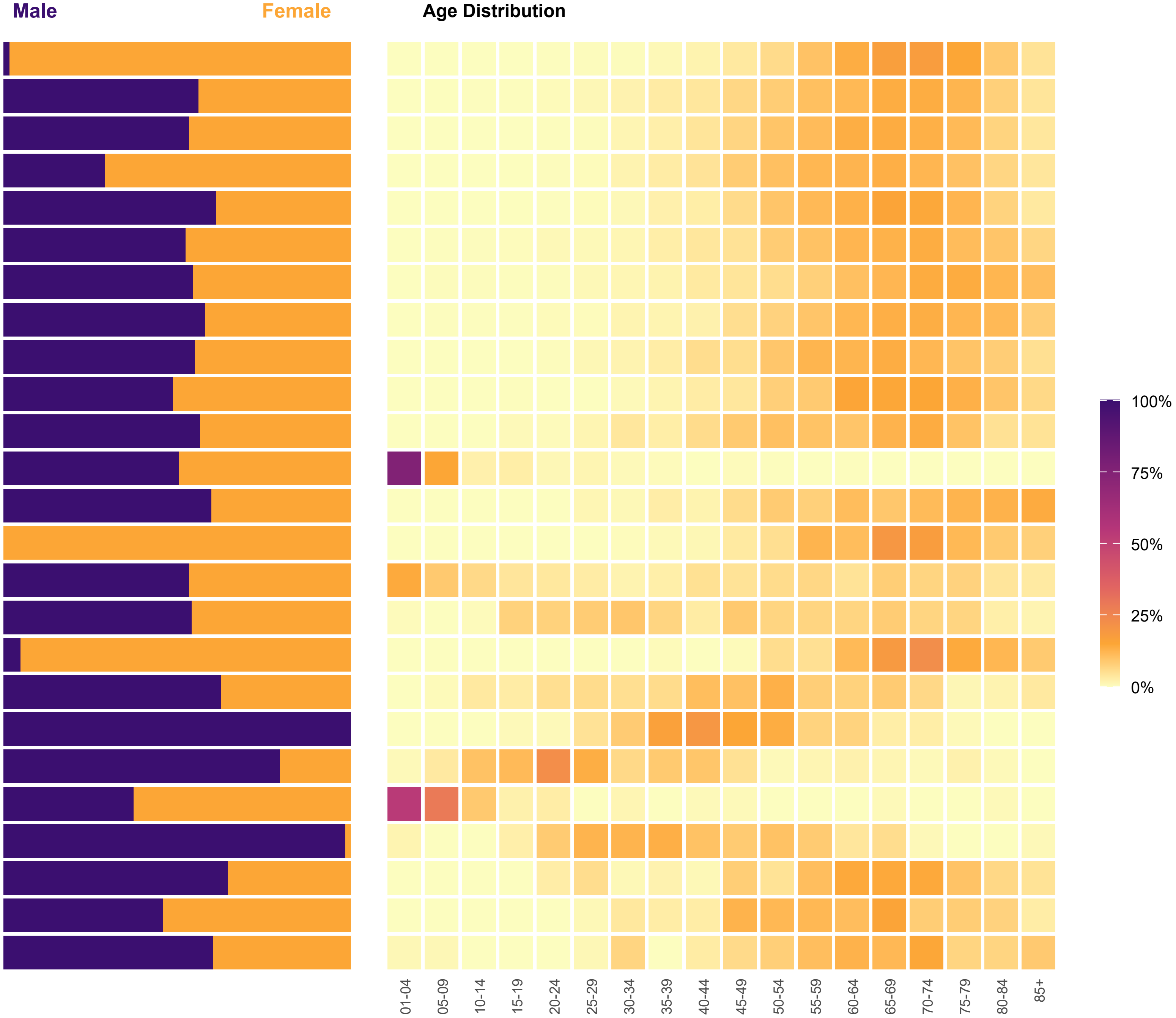

Primary Site: Rib and sternum and clavicle and associated joints

Top 9 Morphology Groups | cases: 3,496

Morphology Group	n	%
Chondrosarcoma	1,572	45.0%
Plasmacytoma	931	26.6%
Osteosarcoma	370	10.6%
Diffuse large B-cell lymphoma	140	4.0%
Langerhans cell histiocytosis	91	2.6%
Sarcoma	87	2.5%
Myxoid chondrosarcoma	74	2.1%
Ewing sarcoma	53	1.5%
Lymphoma	23	0.7%

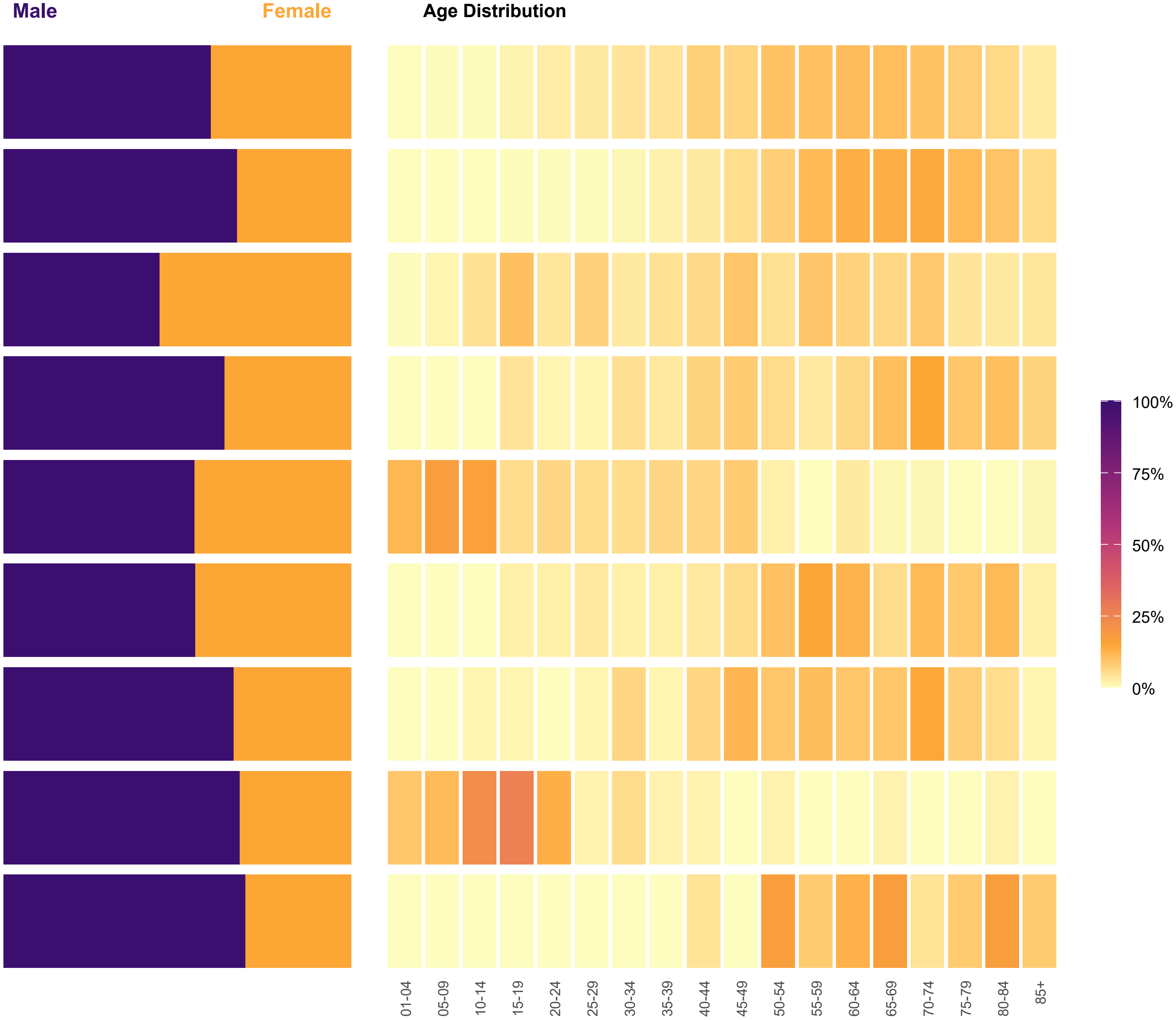

Primary Site: Short bones of lower limb and associated joints

Top 8 Morphology Groups | cases: 732

Morphology Group	n	%
Chondrosarcoma	229	31.3%
Osteosarcoma	180	24.6%
Diffuse large B-cell lymphoma	52	7.1%
Sarcoma	39	5.3%
Myxoid chondrosarcoma	30	4.1%
malignant giant cell tumor of bone	28	3.8%
Synovial sarcoma	22	3.0%
Plasmacytoma	21	2.9%

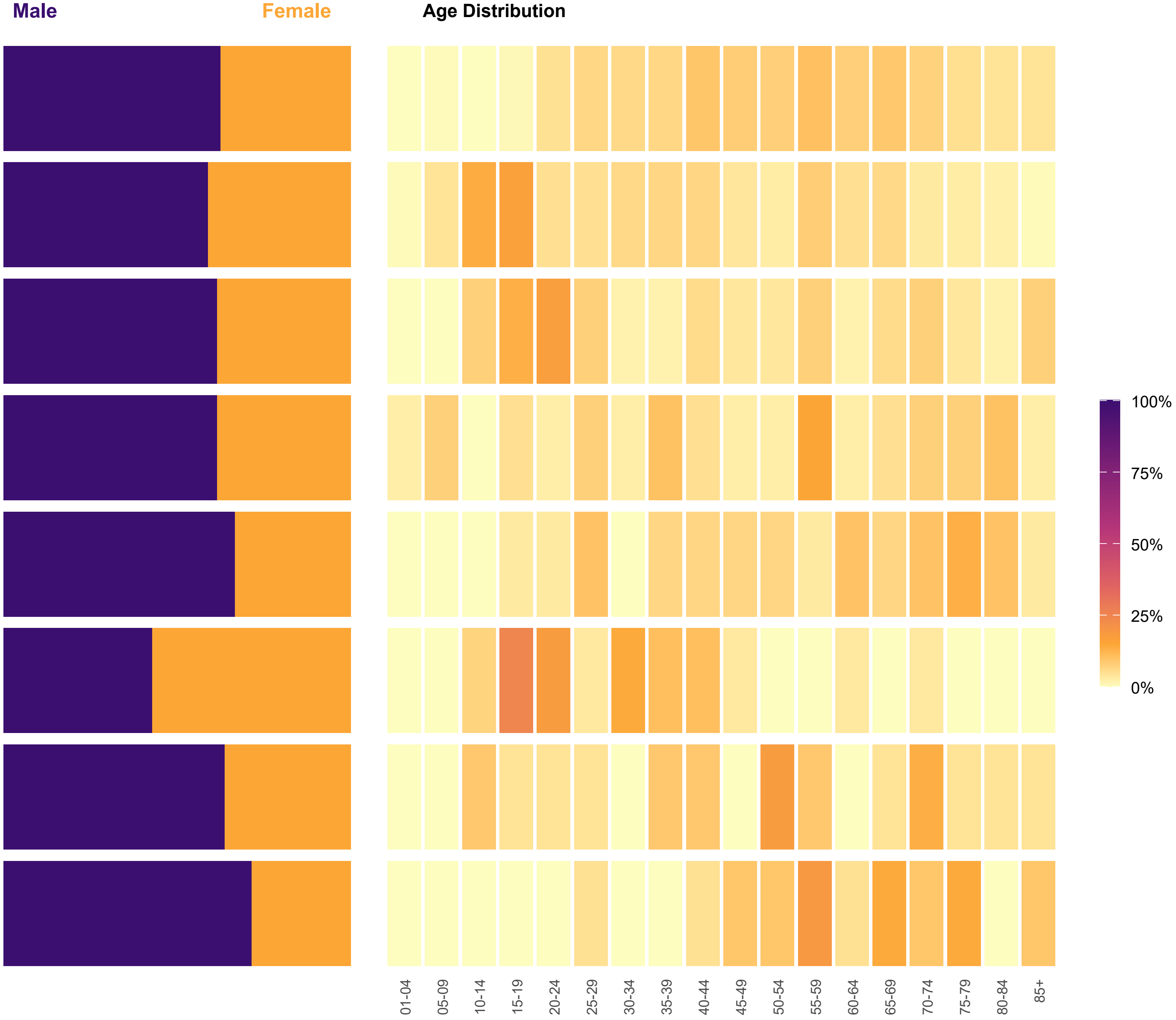

Primary Site: Short bones of upper limb and associated joints

Top 6 Morphology Groups | cases: 555

Morphology Group	n	%
Chondrosarcoma	286	51.5%
Osteosarcoma	81	14.6%
malignant giant cell tumor of bone	50	9.0%
Myxoid chondrosarcoma	25	4.5%
Plasmacytoma	25	4.5%
Sarcoma	22	4.0%

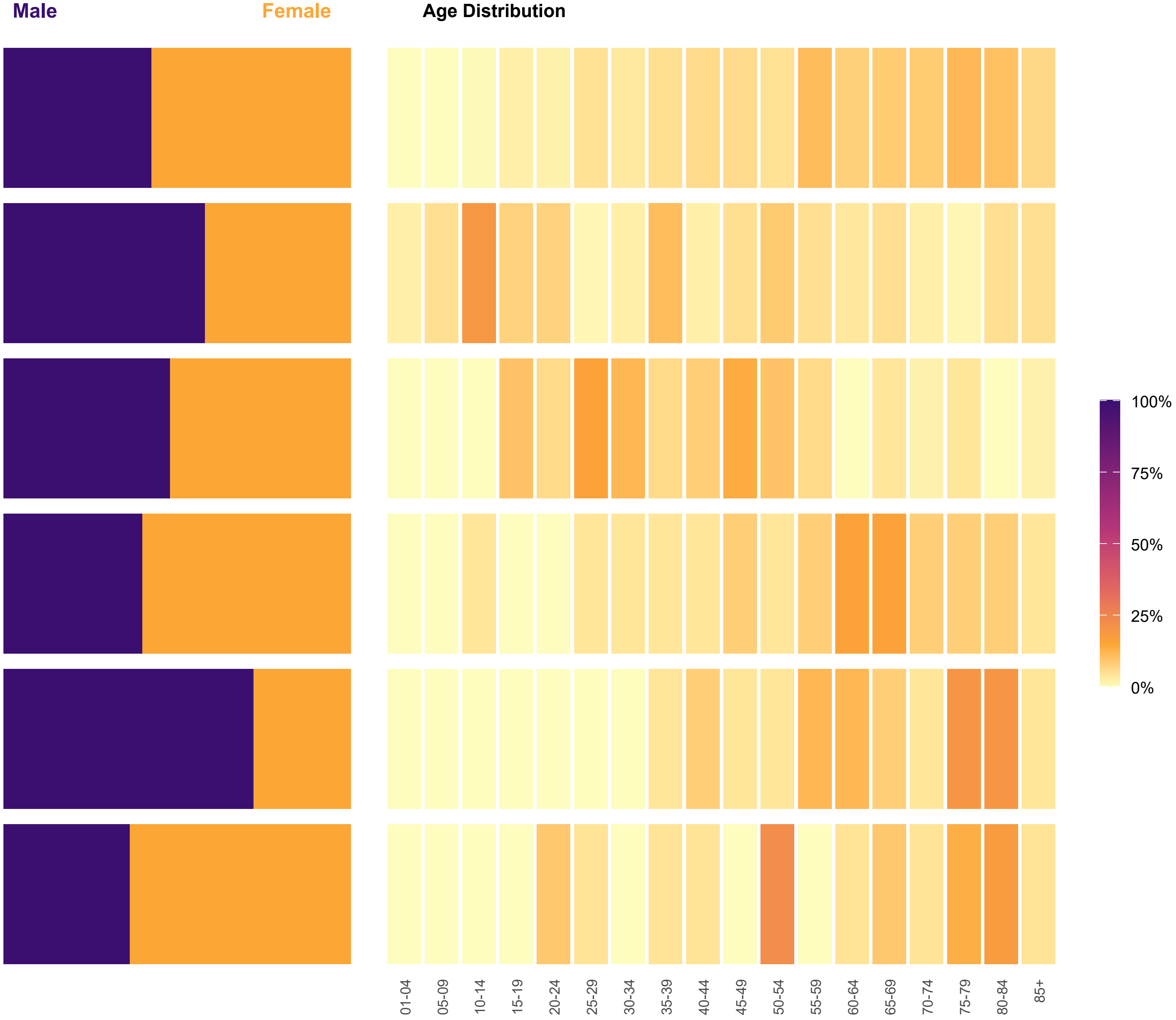

Primary Site: Sinus Other

Top 19 Morphology Groups | cases: 5,429

Morphology Group	n	%
Squamous cell carcinoma	2,273	41.9%
Adenocarcinoma	795	14.6%
Diffuse large B-cell lymphoma	379	7.0%
Melanoma	322	5.9%
Neuroendocrine tumor	305	5.6%
Adenoid cystic carcinoma	290	5.3%
Rhabdomyosarcoma	158	2.9%
Olfactory neurogenic tumor	154	2.8%
Transitional cell carcinoma	88	1.6%
NK/T-cell lymphoma	75	1.4%
Sarcoma	74	1.4%
Lymphoma	68	1.3%
Plasmacytoma	64	1.2%
Chordoma	47	0.9%
Lymphoepithelial carcinoma	32	0.6%
Marginal zone B-cell lymphoma	26	0.5%
Chondrosarcoma	23	0.4%
Follicular lymphoma	21	0.4%
T-cell lymphoma	21	0.4%

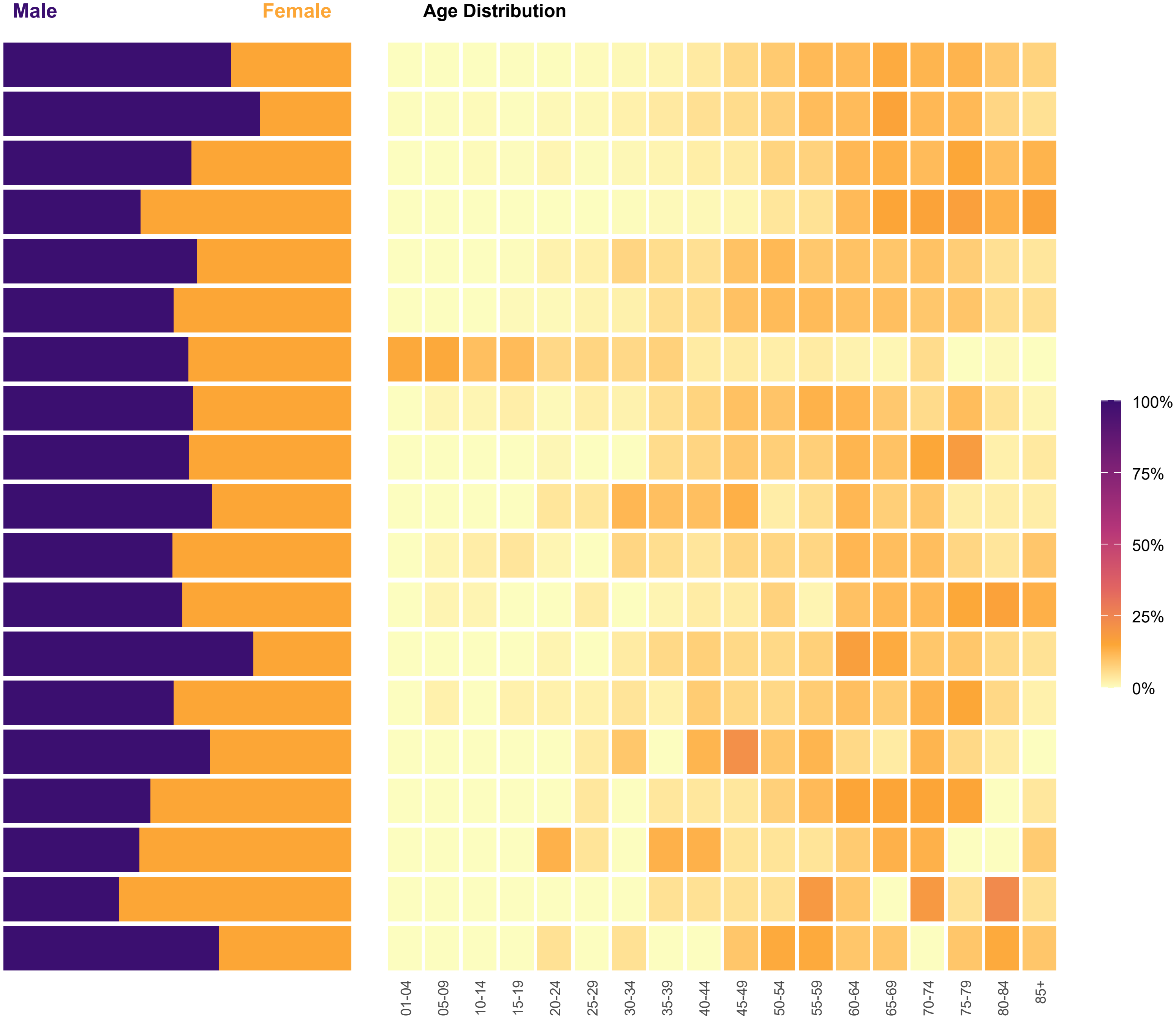

Primary Site: Skin

Top 25 Morphology Groups | cases: 3,009,712

Morphology Group	n	%
Basal cell carcinoma	1,549,330	51.5%
Melanoma	884,721	29.4%
Squamous cell carcinoma	447,132	14.9%
Merkel cell carcinoma	24,370	0.8%
T-cell lymphoma	23,027	0.8%
Adenocarcinoma	22,278	0.7%
Kaposi sarcoma	14,021	0.5%
Dermatofibrosarcoma protuberans	11,084	0.4%
Sarcoma	7,061	0.2%
Adenoid basal carcinoma	4,629	0.2%
Marginal zone B-cell lymphoma	3,261	0.1%
Anaplastic large cell lymphoma	2,878	<0.1%
Lymphoma	2,718	<0.1%
Diffuse large B-cell lymphoma	2,627	<0.1%
Follicular lymphoma	2,389	<0.1%
Leiomyosarcoma	2,276	<0.1%
Hemangiosarcoma	1,640	<0.1%
Adenoid cystic carcinoma	895	<0.1%
Trichilemmocarcinoma	415	<0.1%
Neuroendocrine tumor	322	<0.1%
Langerhans cell histiocytosis	195	<0.1%
Carcinosarcoma	176	<0.1%
Pilomatrical carcinoma	155	<0.1%
Transitional cell carcinoma	151	<0.1%
Liposarcoma	141	<0.1%

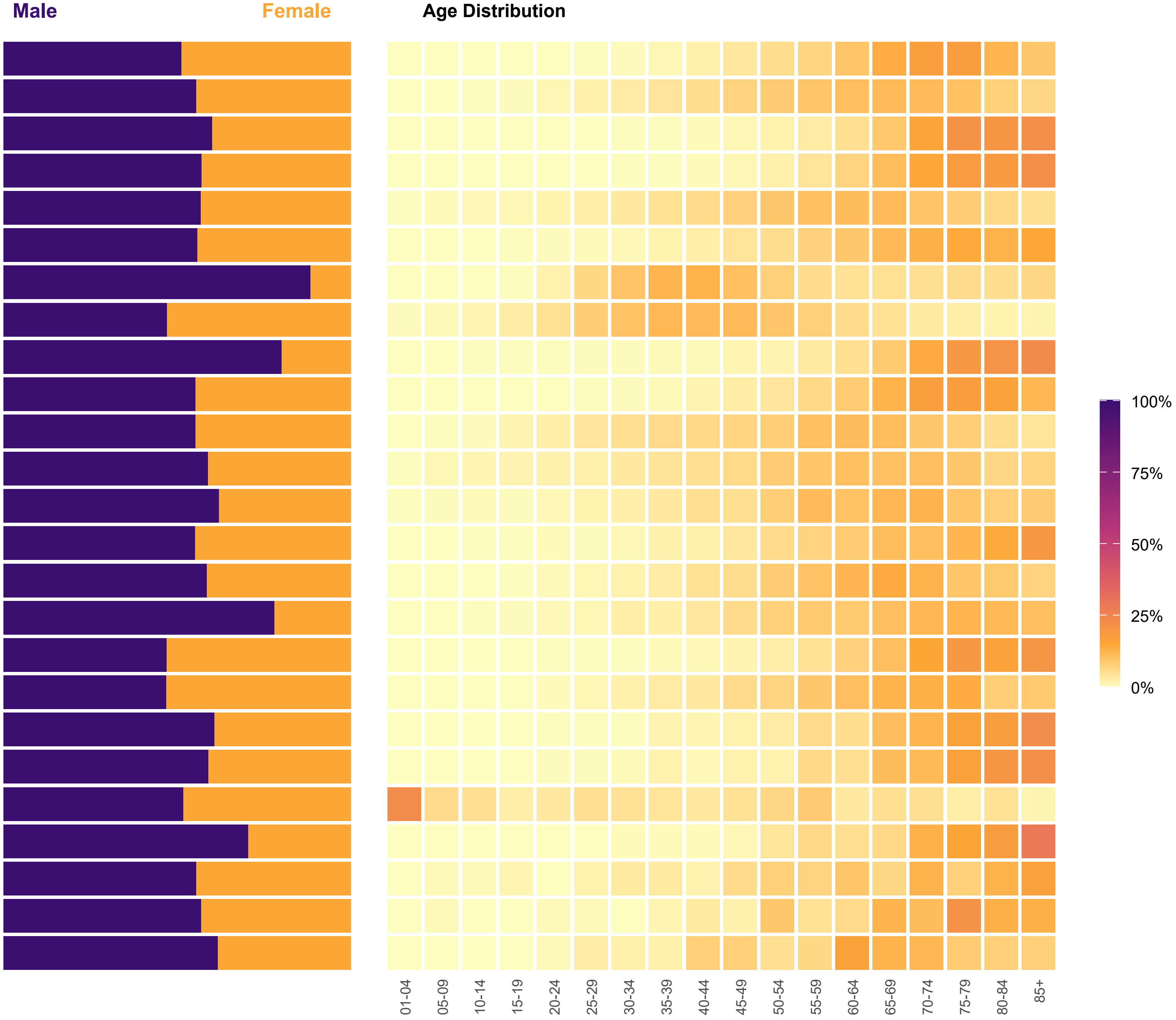

Primary Site: Small intestine

Top 25 Morphology Groups | cases: 99,767

Morphology Group	n	%
Neuroendocrine tumor	48,478	48.6%
Adenocarcinoma	29,747	29.8%
Gastrointestinal stromal tumor	9,623	9.6%
Diffuse large B-cell lymphoma	4,197	4.2%
Follicular lymphoma	2,512	2.5%
Marginal zone B-cell lymphoma	876	0.9%
Lymphoma	720	0.7%
Leiomyosarcoma	683	0.7%
Sarcoma	581	0.6%
T-cell lymphoma	539	0.5%
Burkitt lymphoma	422	0.4%
Mantle cell lymphoma	171	0.2%
Melanoma	123	0.1%
Squamous cell carcinoma	122	0.1%
Anaplastic large cell lymphoma	95	<0.1%
Hemangiosarcoma	75	<0.1%
Plasmacytoma	74	<0.1%
Kaposi sarcoma	68	<0.1%
lymphoma non-Hodgkin	51	<0.1%
Myeloid sarcoma	49	<0.1%
Liposarcoma	42	<0.1%
Goblet cell carcinoid	41	<0.1%
NK/T-cell lymphoma	35	<0.1%
Ewing sarcoma	30	<0.1%
Dedifferentiated liposarcoma	29	<0.1%

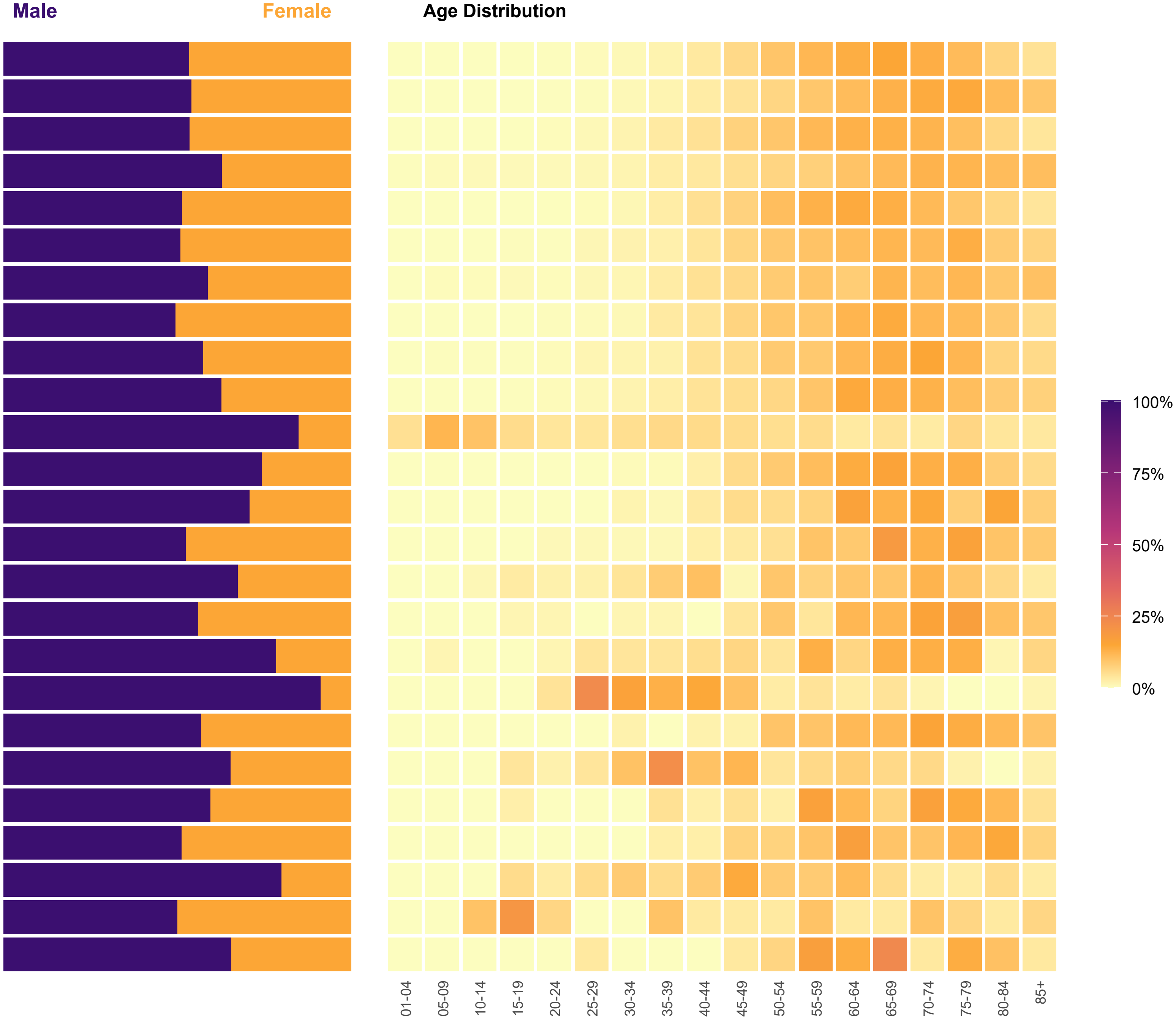

Primary Site: Stomach

Top 25 Morphology Groups | cases: 411,046

Morphology Group	n	%
Adenocarcinoma	341,272	83.0%
Gastrointestinal stromal tumor	22,327	5.4%
Neuroendocrine tumor	20,805	5.1%
Marginal zone B-cell lymphoma	9,502	2.3%
Diffuse large B-cell lymphoma	8,859	2.2%
Squamous cell carcinoma	2,742	0.7%
Lymphoma	1,738	0.4%
Sarcoma	790	0.2%
Leiomyosarcoma	619	0.2%
Follicular lymphoma	400	<0.1%
Burkitt lymphoma	309	<0.1%
Mantle cell lymphoma	255	<0.1%
Kaposi sarcoma	185	<0.1%
T-cell lymphoma	169	<0.1%
lymphoma non-Hodgkin	123	<0.1%
Plasmacytoma	118	<0.1%
Melanoma	114	<0.1%
Lymphoepithelial carcinoma	103	<0.1%
Anaplastic large cell lymphoma	71	<0.1%
Carcinosarcoma	46	<0.1%
Chronic lymphocytic leukemia	34	<0.1%
Hodgkin lymphoma	31	<0.1%
Liposarcoma	27	<0.1%
Hemangiosarcoma	26	<0.1%
Lymphoplasmacytic lymphoma	25	<0.1%

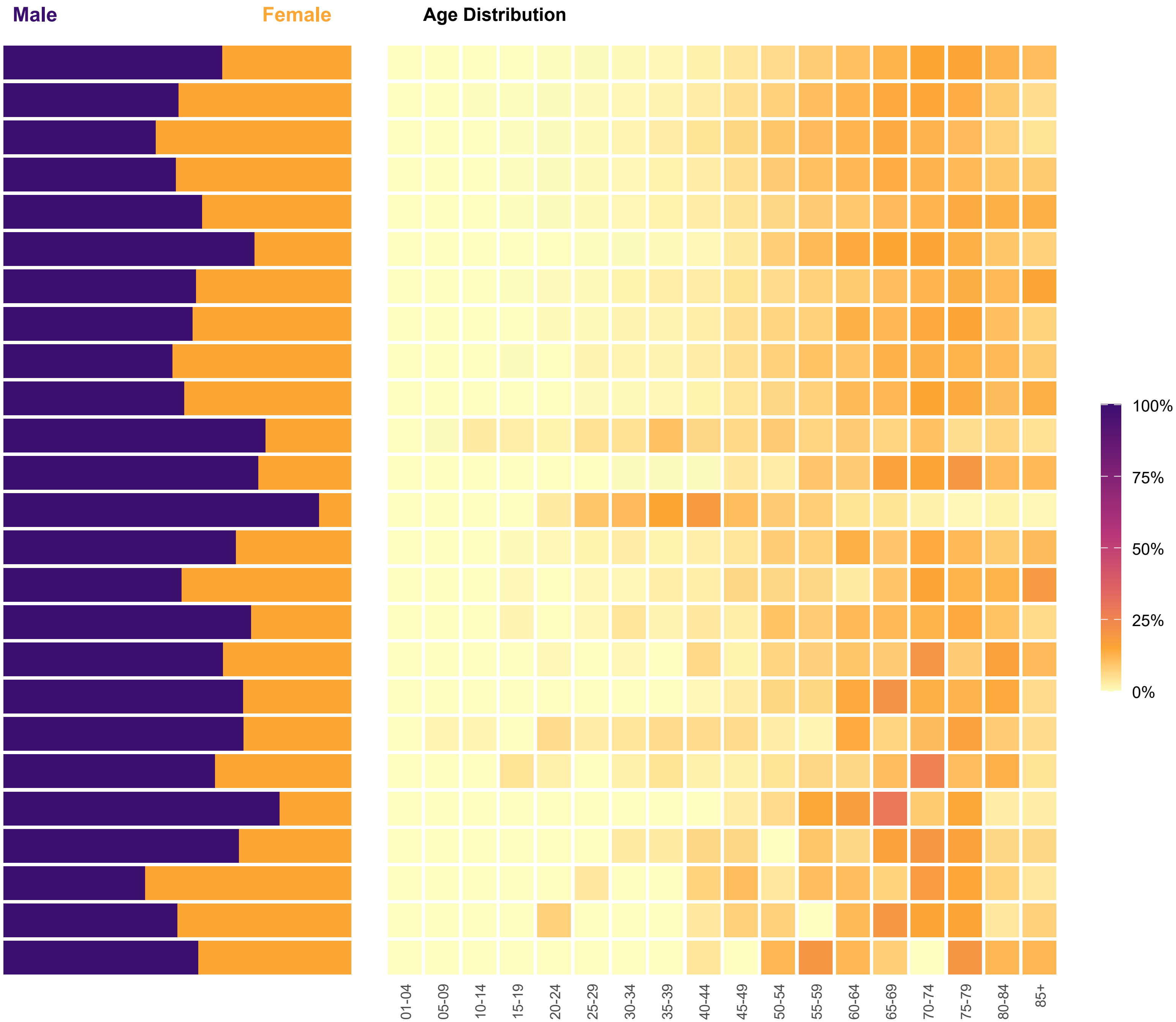

Primary Site: Testis

Top 25 Morphology Groups | cases: 140,592

Morphology Group	n	%
Seminoma	78,037	55.5%
Mixed germ cell tumor	26,762	19.0%
Embryonal carcinoma	12,070	8.6%
Teratocarcinoma	3,694	2.6%
Teratoma	3,228	2.3%
Germinoma	2,940	2.1%
Germ cell tumor nonseminomatous	2,861	2.0%
Choriocarcinoma	2,849	2.0%
Diffuse large B-cell lymphoma	2,315	1.6%
Yolk sac tumor	1,696	1.2%
Spermatocytic seminoma	830	0.6%
Seminoma anaplastic	657	0.5%
Leydig cell tumor malignant	507	0.4%
Rhabdomyosarcoma	360	0.3%
Adenocarcinoma	239	0.2%
Lymphoma	175	0.1%
Liposarcoma	173	0.1%
Sertoli cell carcinoma	145	0.1%
Leiomyosarcoma	133	<0.1%
Neuroendocrine tumor	123	<0.1%
Sarcoma	105	<0.1%
Dedifferentiated liposarcoma	101	<0.1%
Mesothelioma	101	<0.1%
Dysgerminoma	55	<0.1%
Burkitt lymphoma	48	<0.1%

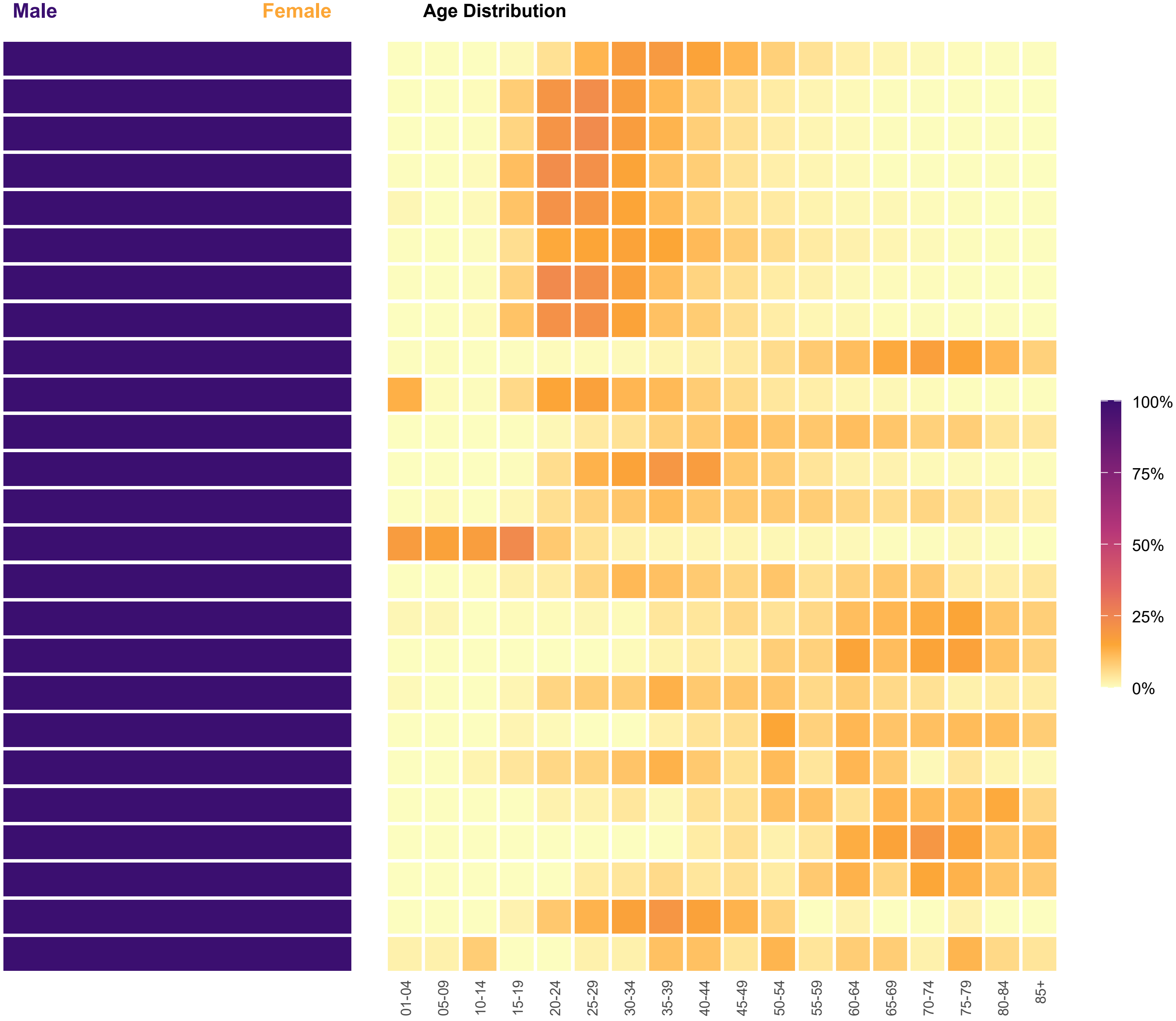

Primary Site: Thymus

Top 13 Morphology Groups | cases: 13,384

Morphology Group	n	%
Thymoma	8,696	65.0%
Thymic carcinoma	1,762	13.2%
Squamous cell carcinoma	1,282	9.6%
Neuroendocrine tumor	690	5.2%
B-cell lymphoma	293	2.2%
Diffuse large B-cell lymphoma	177	1.3%
Adenocarcinoma	132	1.0%
Hodgkin lymphoma	78	0.6%
Lymphoepithelial carcinoma	48	0.4%
Marginal zone B-cell lymphoma	38	0.3%
Lymphoma	32	0.2%
Transitional cell carcinoma	31	0.2%
Seminoma	20	0.1%

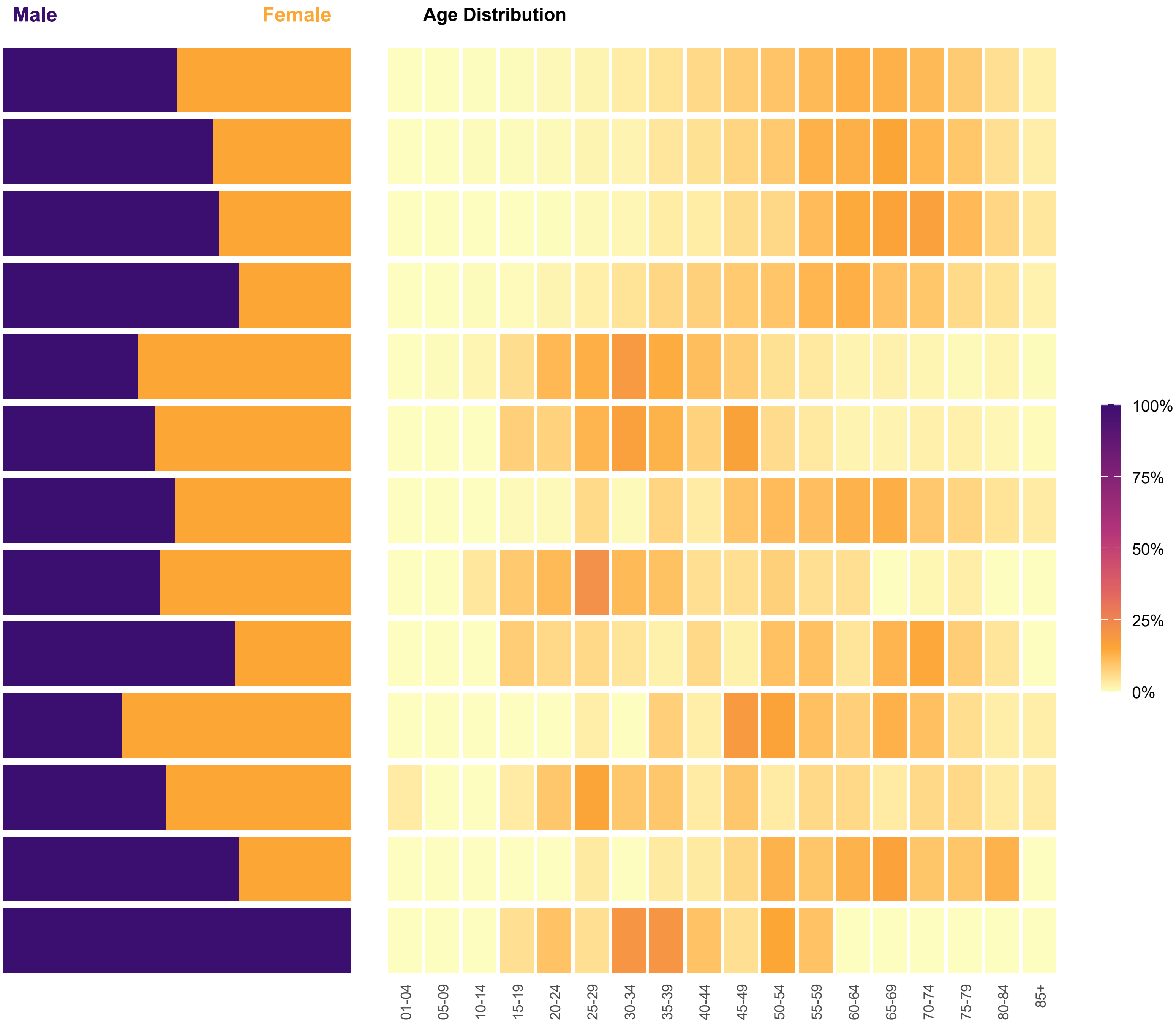

Primary Site: Thyroid

Top 14 Morphology Groups | cases: 404,410

Morphology Group	n	%
Adenocarcinoma	394,646	97.6%
Medullary thyroid carcinoma	5,404	1.3%
Diffuse large B-cell lymphoma	1,677	0.4%
Squamous cell carcinoma	703	0.2%
Marginal zone B-cell lymphoma	552	0.1%
Lymphoma	328	<0.1%
Follicular lymphoma	260	<0.1%
Neuroendocrine tumor	195	<0.1%
Hemangiosarcoma	138	<0.1%
Sarcoma	84	<0.1%
Burkitt lymphoma	72	<0.1%
Plasmacytoma	38	<0.1%
Leiomyosarcoma	35	<0.1%
Hodgkin lymphoma	27	<0.1%

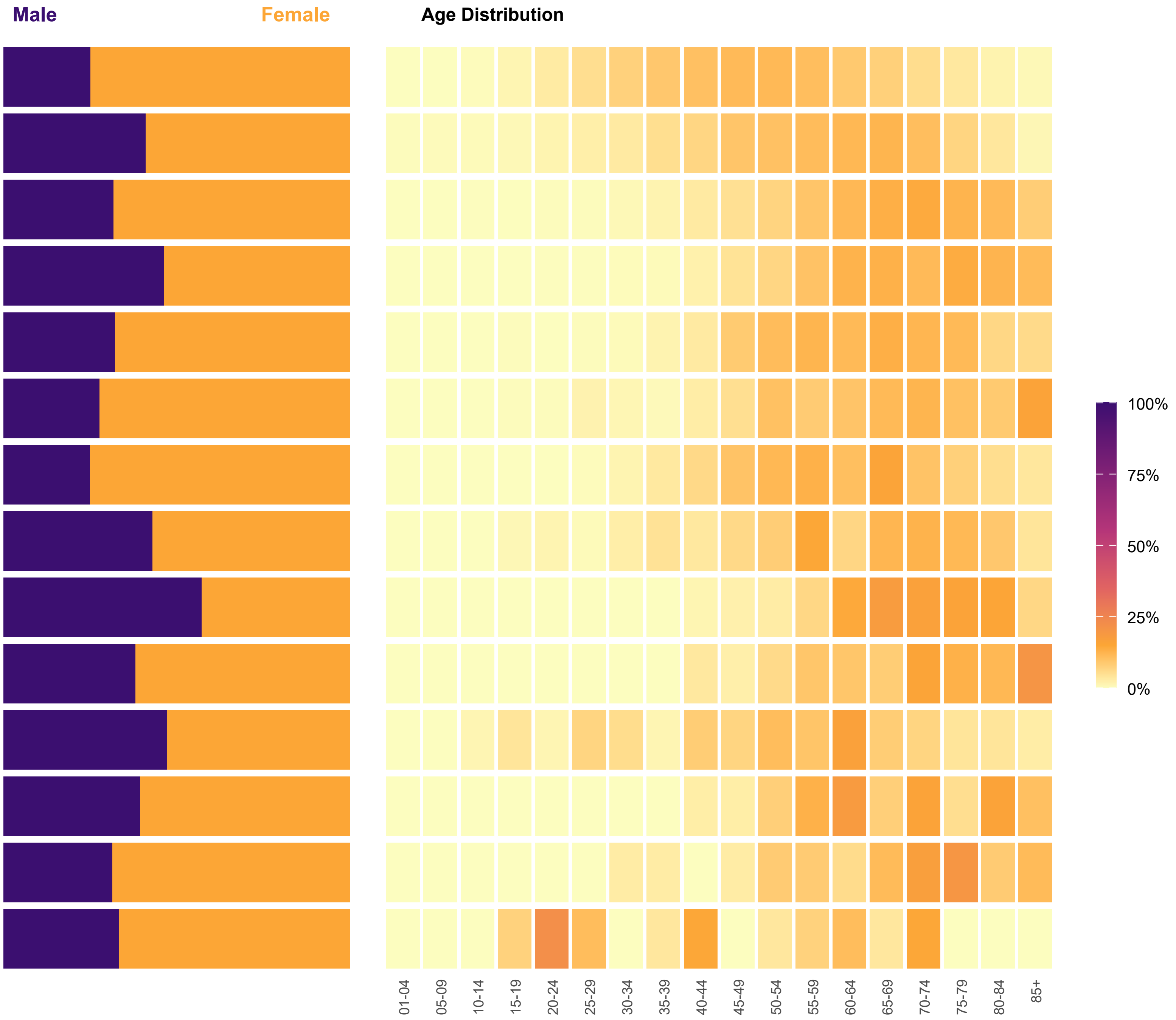

Primary Site: Tongue

Top 20 Morphology Groups | cases: 133,047

Morphology Group	n	%
Squamous cell carcinoma	128,625	96.7%
Adenocarcinoma	1,142	0.9%
Diffuse large B-cell lymphoma	1,033	0.8%
Adenoid cystic carcinoma	590	0.4%
Lymphoepithelial carcinoma	173	0.1%
Lymphoma	160	0.1%
Neuroendocrine tumor	156	0.1%
Follicular lymphoma	152	0.1%
Marginal zone B-cell lymphoma	147	0.1%
Mantle cell lymphoma	139	0.1%
Kaposi sarcoma	99	<0.1%
Sarcoma	86	<0.1%
T-cell lymphoma	75	<0.1%
Plasmacytoma	65	<0.1%
Melanoma	43	<0.1%
Rhabdomyosarcoma	36	<0.1%
Transitional cell carcinoma	30	<0.1%
Leiomyosarcoma	29	<0.1%
Burkitt lymphoma	29	<0.1%
Chronic lymphocytic leukemia	27	<0.1%

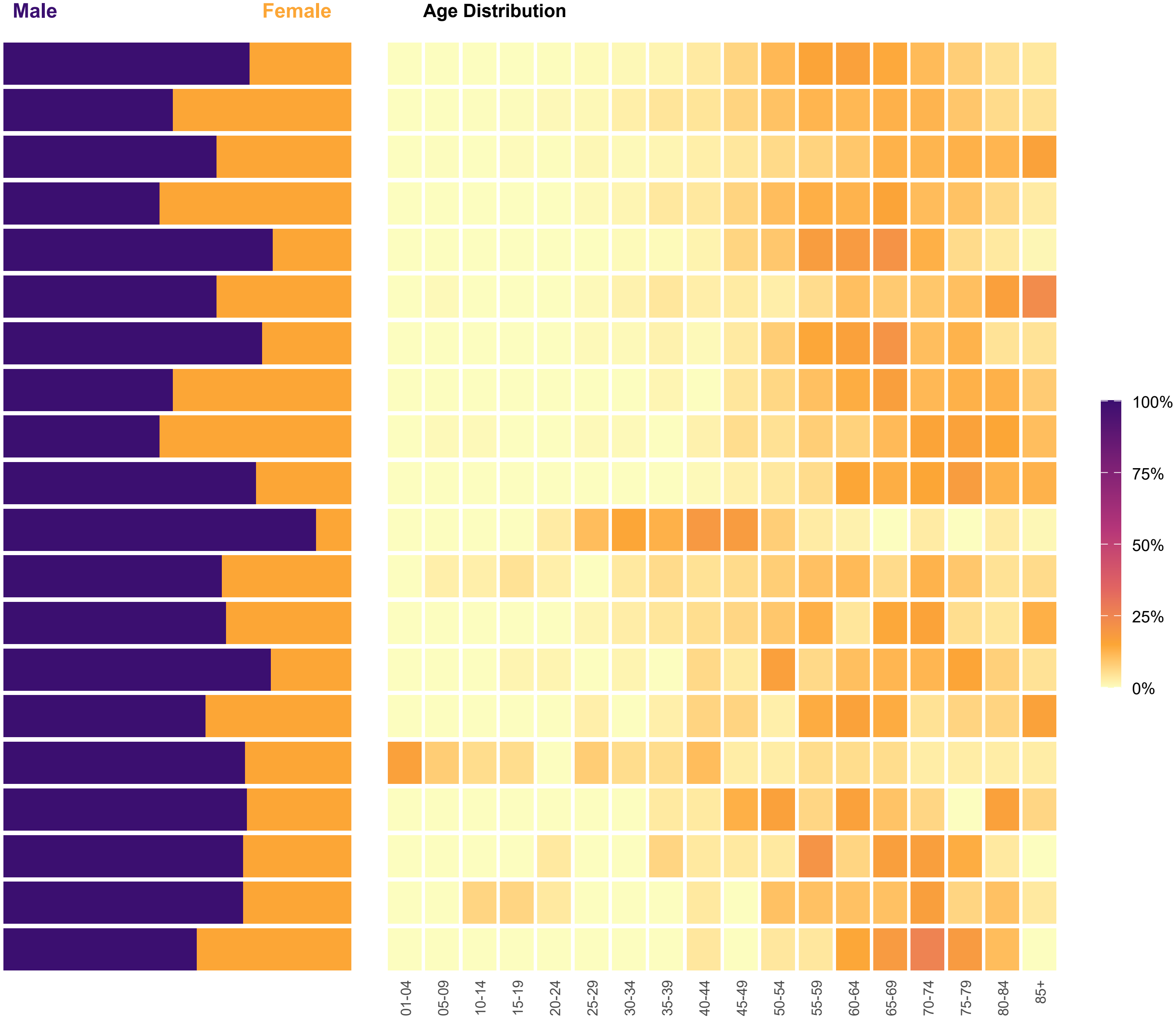

Primary Site: Trachea

Top 7 Morphology Groups | cases: 2,776

Morphology Group	n	%
Squamous cell carcinoma	1,610	58.0%
Adenoid cystic carcinoma	441	15.9%
Adenocarcinoma	270	9.7%
Neuroendocrine tumor	257	9.3%
Marginal zone B-cell lymphoma	33	1.2%
Chondrosarcoma	32	1.2%
Diffuse large B-cell lymphoma	25	0.9%

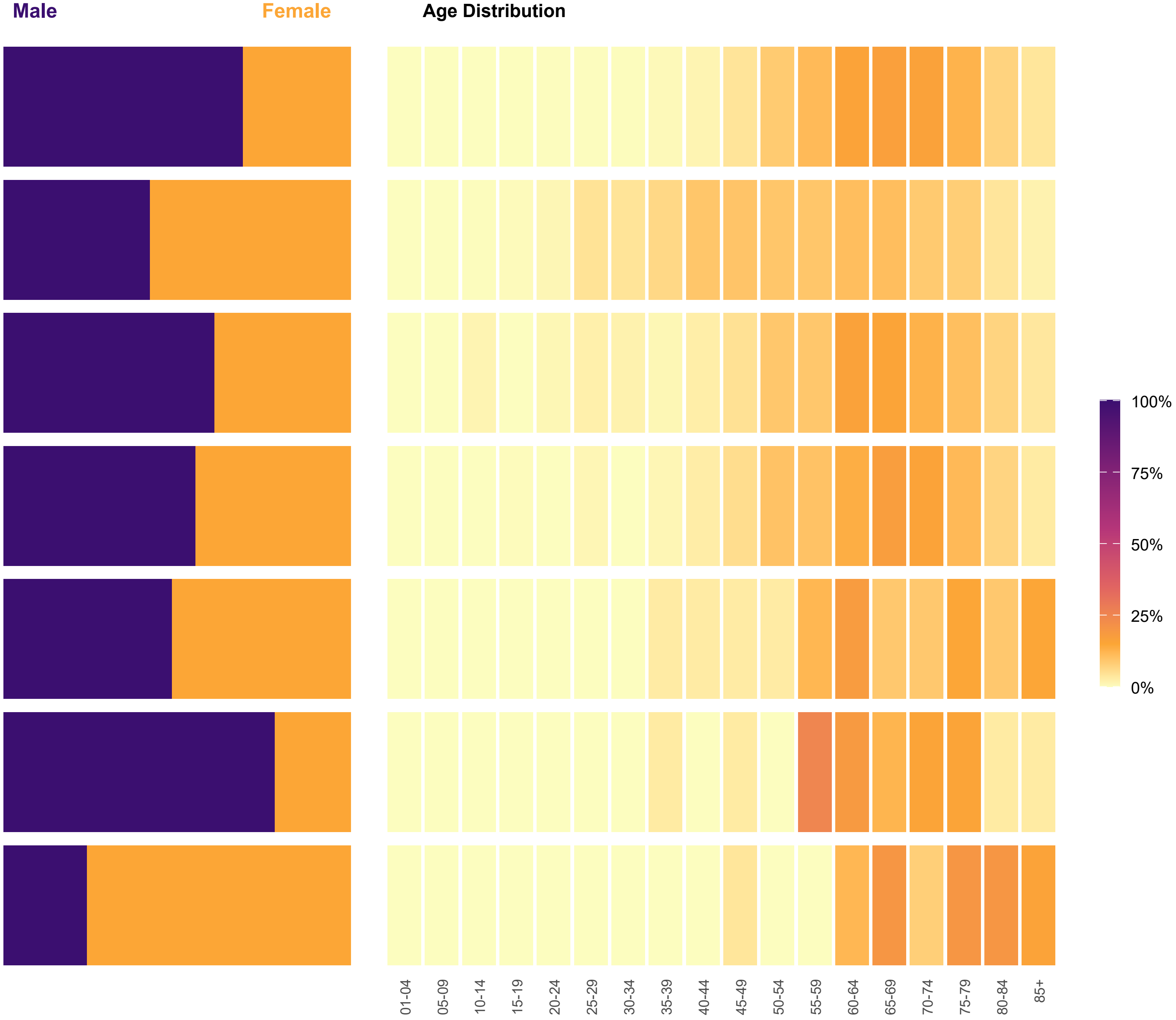

Primary Site: Ureter

Top 5 Morphology Groups | cases: 23,731

Morphology Group	n	%
Transitional cell carcinoma	22,761	95.9%
Squamous cell carcinoma	354	1.5%
Adenocarcinoma	266	1.1%
Neuroendocrine tumor	199	0.8%
Leiomyosarcoma	20	<0.1%

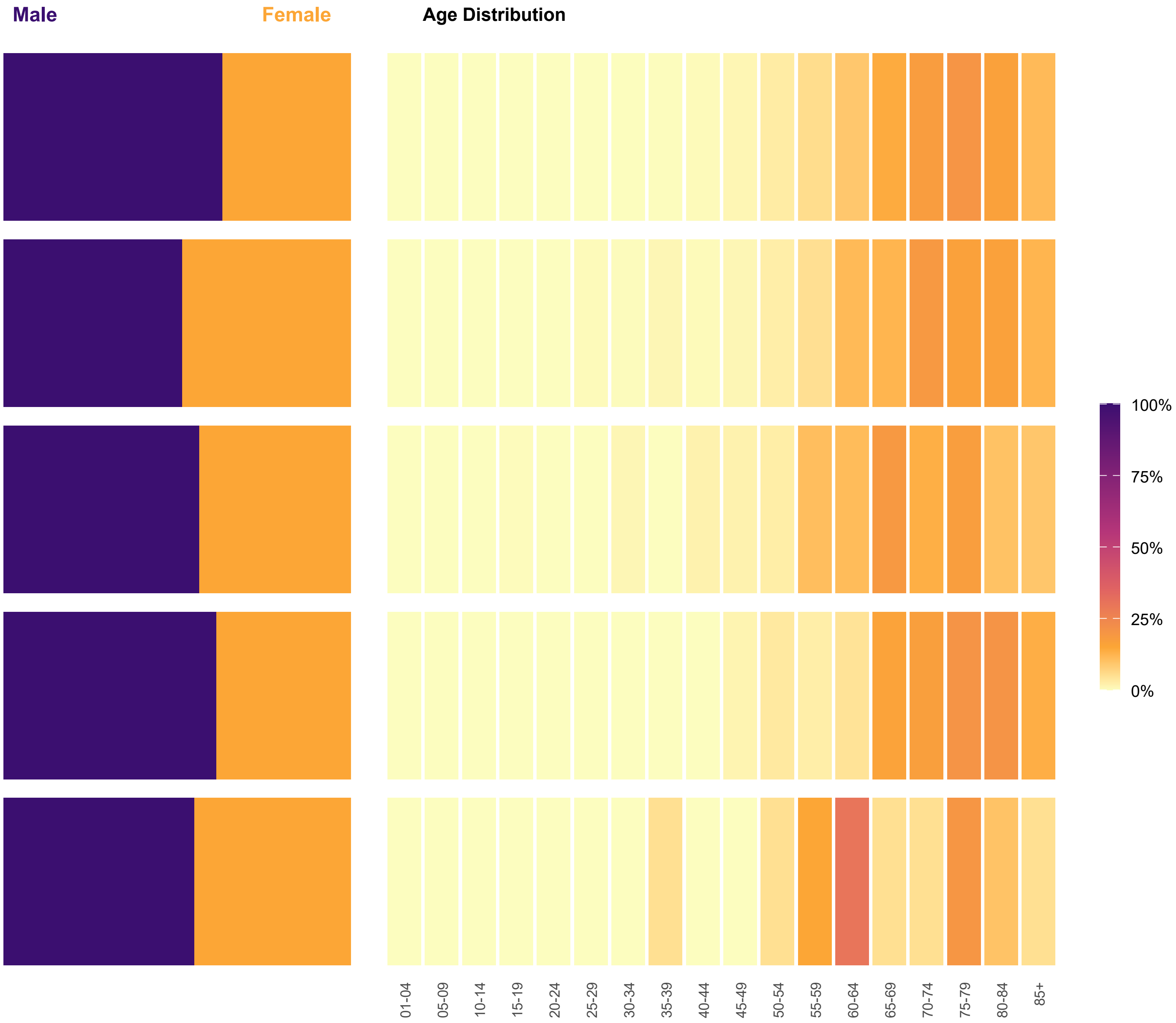

Primary Site: Urethra

Top 5 Morphology Groups | cases: 6,640

Morphology Group	n	%
Transitional cell carcinoma	3,793	57.1%
Squamous cell carcinoma	1,587	23.9%
Adenocarcinoma	927	14.0%
Melanoma	181	2.7%
Neuroendocrine tumor	68	1.0%

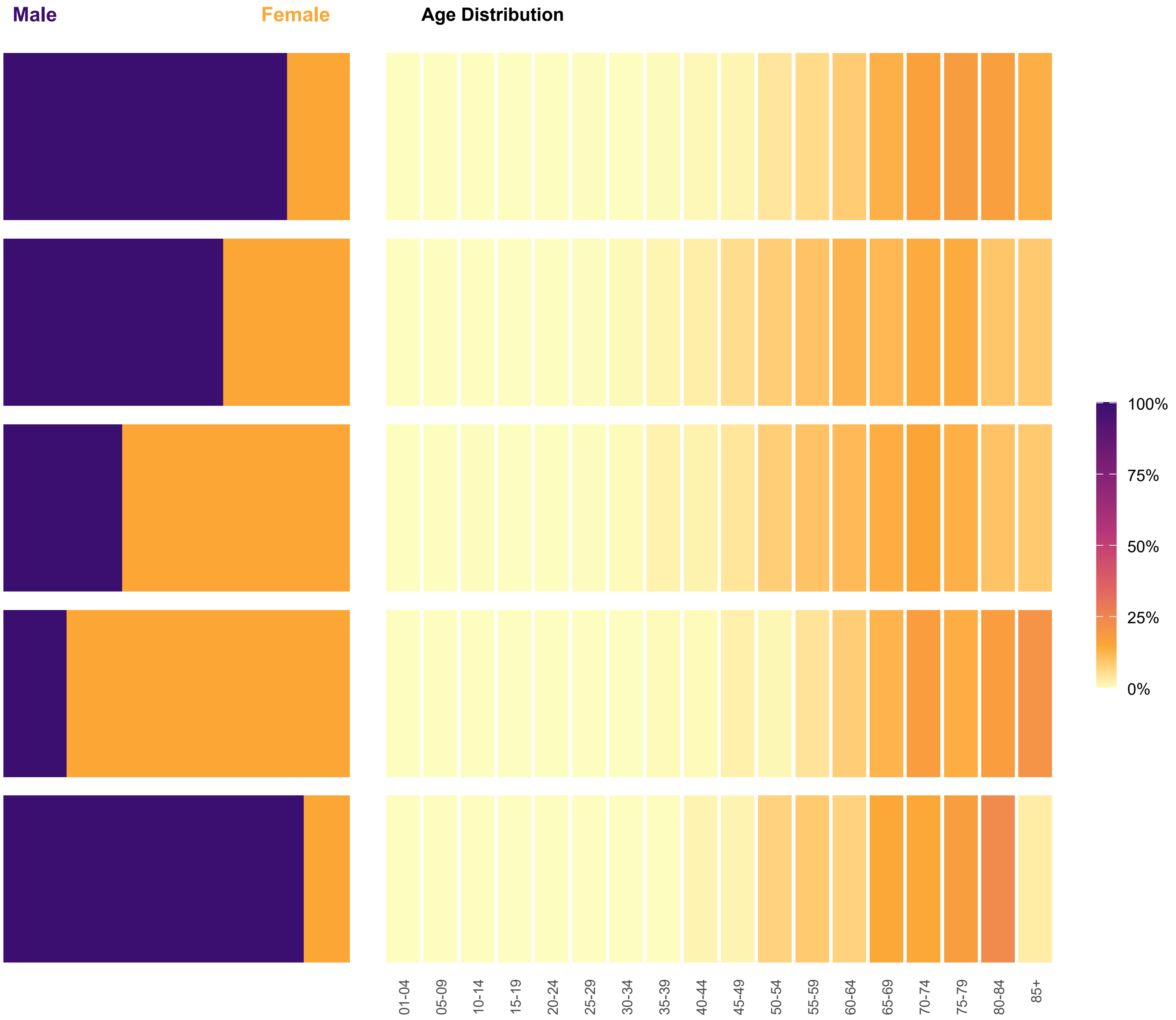

Primary Site: Urinary Bladder

Top 21 Morphology Groups | cases: 758,394

Morphology Group	n	%
Transitional cell carcinoma	727,609	95.9%
Squamous cell carcinoma	11,758	1.6%
Adenocarcinoma	9,166	1.2%
Neuroendocrine tumor	6,950	0.9%
Carcinosarcoma	532	<0.1%
Leiomyosarcoma	460	<0.1%
Sarcoma	396	<0.1%
Diffuse large B-cell lymphoma	255	<0.1%
Rhabdomyosarcoma	253	<0.1%
Lymphoepithelial carcinoma	220	<0.1%
Marginal zone B-cell lymphoma	158	<0.1%
Lymphoma	74	<0.1%
Hemangiosarcoma	62	<0.1%
Melanoma	60	<0.1%
Paraganglioma	50	<0.1%
Follicular lymphoma	34	<0.1%
Plasmacytoma	34	<0.1%
Undifferentiated sarcoma	27	<0.1%
lymphoma non-Hodgkin	22	<0.1%
Chronic lymphocytic leukemia	21	<0.1%
Osteosarcoma	20	<0.1%

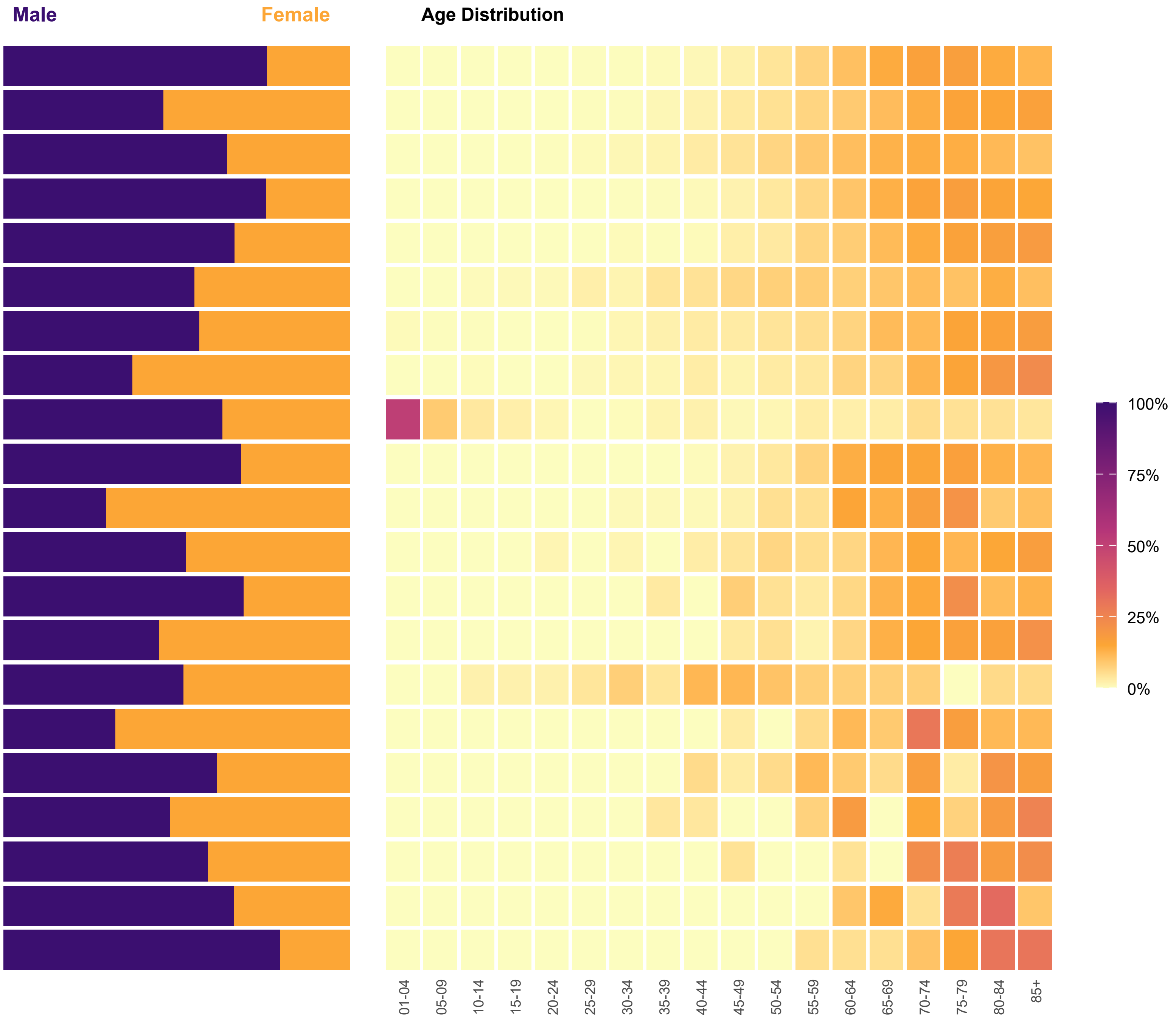

Primary Site: Urinary Other

Top 4 Morphology Groups | cases: 11,076

Morphology Group	n	%
Transitional cell carcinoma	10,636	96.0%
Adenocarcinoma	231	2.1%
Squamous cell carcinoma	134	1.2%
Neuroendocrine tumor	39	0.4%

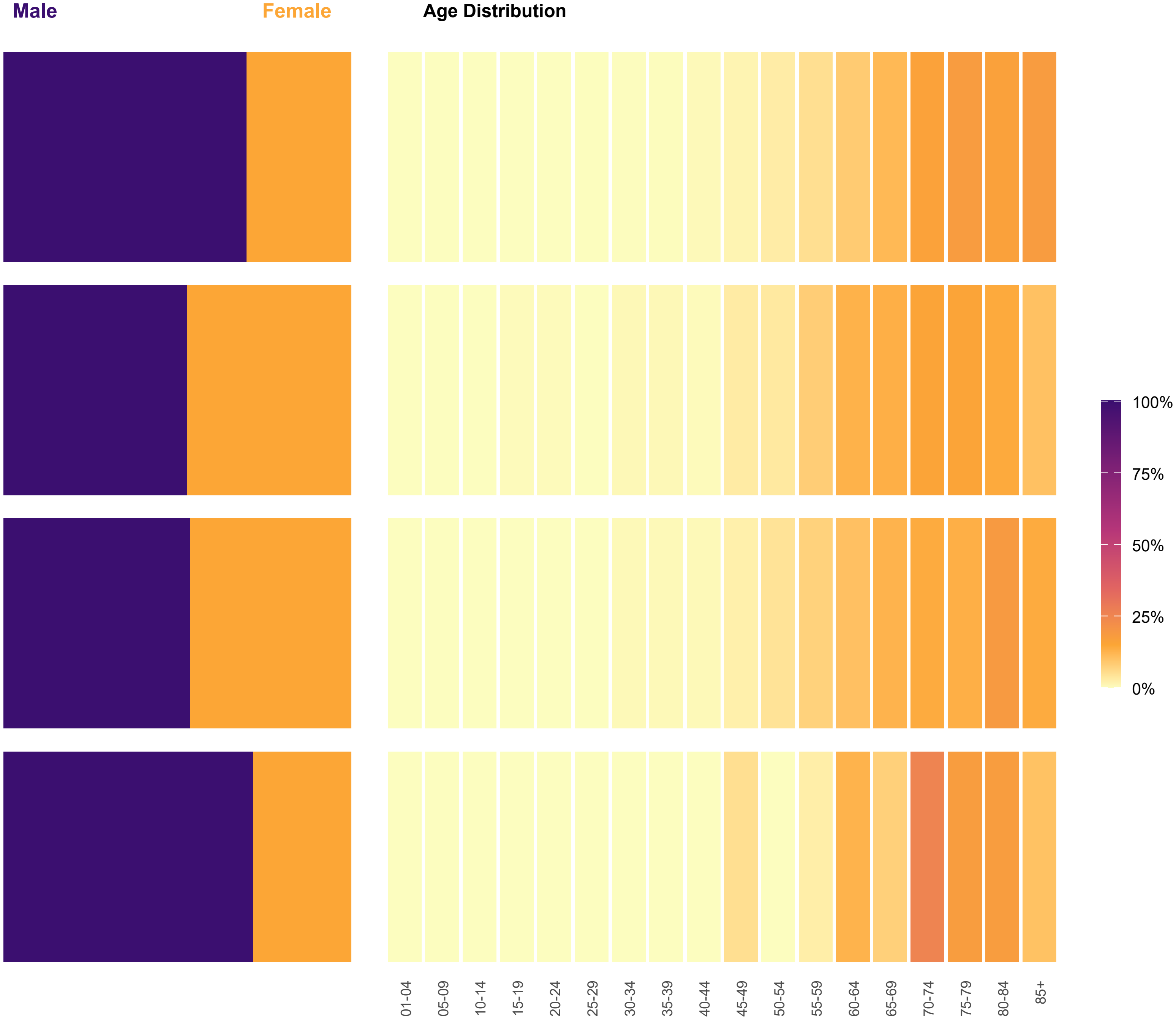

Primary Site: Vagina

Top 12 Morphology Groups | cases: 16,093

Morphology Group	n	%
Squamous cell carcinoma	11,587	72.0%
Adenocarcinoma	2,397	14.9%
Melanoma	947	5.9%
Neuroendocrine tumor	239	1.5%
Leiomyosarcoma	221	1.4%
Carcinosarcoma	123	0.8%
Sarcoma	99	0.6%
Diffuse large B-cell lymphoma	97	0.6%
Rhabdomyosarcoma	86	0.5%
Mullerian mixed tumor	41	0.3%
Transitional cell carcinoma	36	0.2%
Adenoid cystic carcinoma	20	0.1%

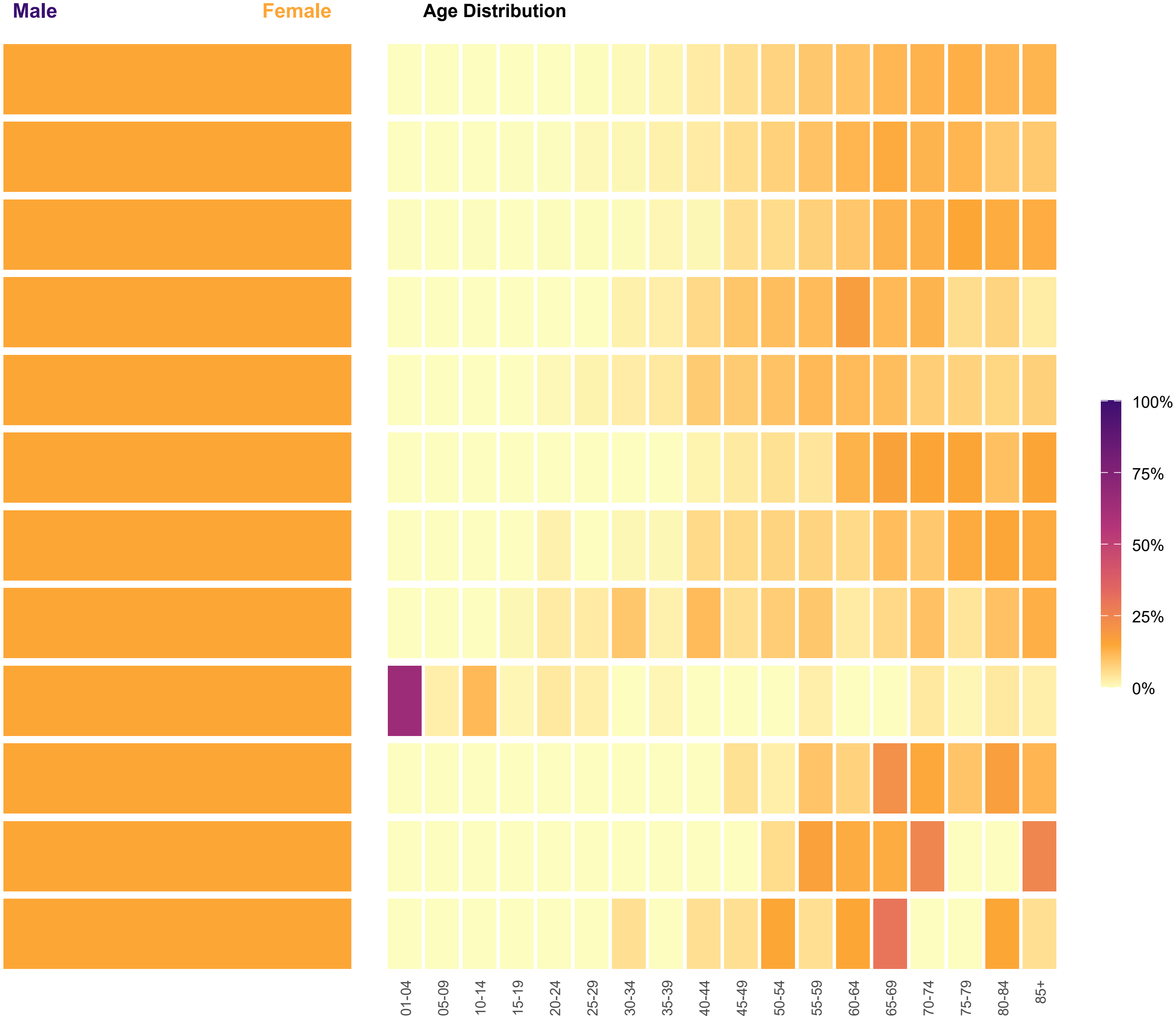

Primary Site: Vertebral column

Top 19 Morphology Groups | cases: 6,707

Morphology Group	n	%
Plasmacytoma	2,689	40.1%
Diffuse large B-cell lymphoma	948	14.1%
Chordoma	853	12.7%
Chondrosarcoma	495	7.4%
Osteosarcoma	317	4.7%
Lymphoma	261	3.9%
Follicular lymphoma	204	3.0%
Langerhans cell histiocytosis	167	2.5%
Sarcoma	141	2.1%
malignant giant cell tumor of bone	80	1.2%
Hemangiosarcoma	46	0.7%
Marginal zone B-cell lymphoma	42	0.6%
Ewing sarcoma	34	0.5%
Burkitt lymphoma	27	0.4%
Ependymoma	26	0.4%
Anaplastic large cell lymphoma	23	0.3%
Myxoid chondrosarcoma	22	0.3%
Epithelioid hemangioendothelioma	21	0.3%
Lymphoplasmacytic lymphoma	21	0.3%

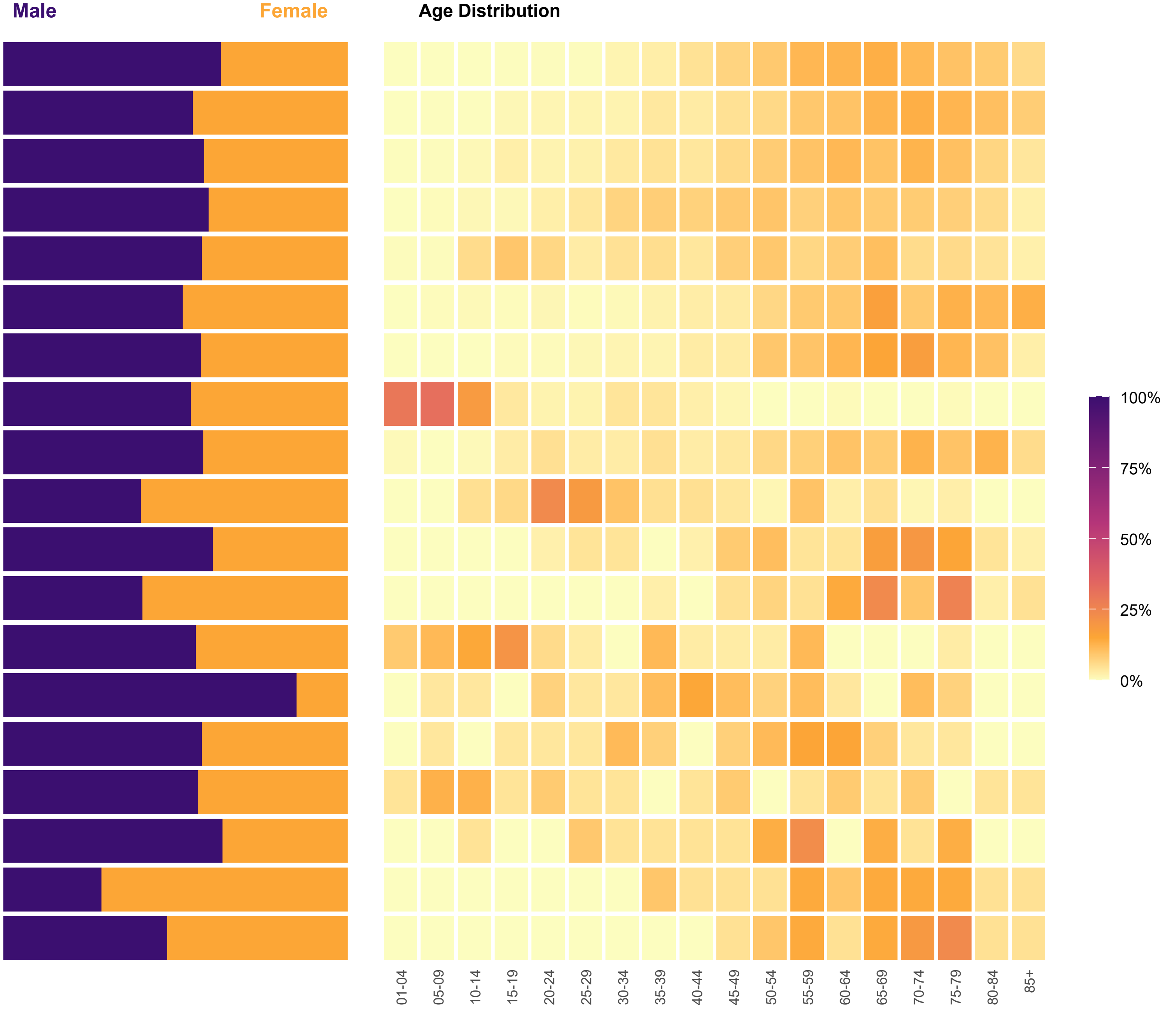

Primary Site: Vulva

Top 14 Morphology Groups | cases: 76,627

Morphology Group	n	%
Squamous cell carcinoma	63,858	83.3%
Basal cell carcinoma	4,333	5.7%
Adenocarcinoma	4,015	5.2%
Melanoma	3,174	4.1%
Sarcoma	194	0.3%
Adenoid cystic carcinoma	171	0.2%
Dermatofibrosarcoma protuberans	155	0.2%
Leiomyosarcoma	152	0.2%
Neuroendocrine tumor	88	0.1%
Transitional cell carcinoma	73	<0.1%
Diffuse large B-cell lymphoma	44	<0.1%
Rhabdomyosarcoma	30	<0.1%
Carcinosarcoma	28	<0.1%
Adenoid basal carcinoma	22	<0.1%

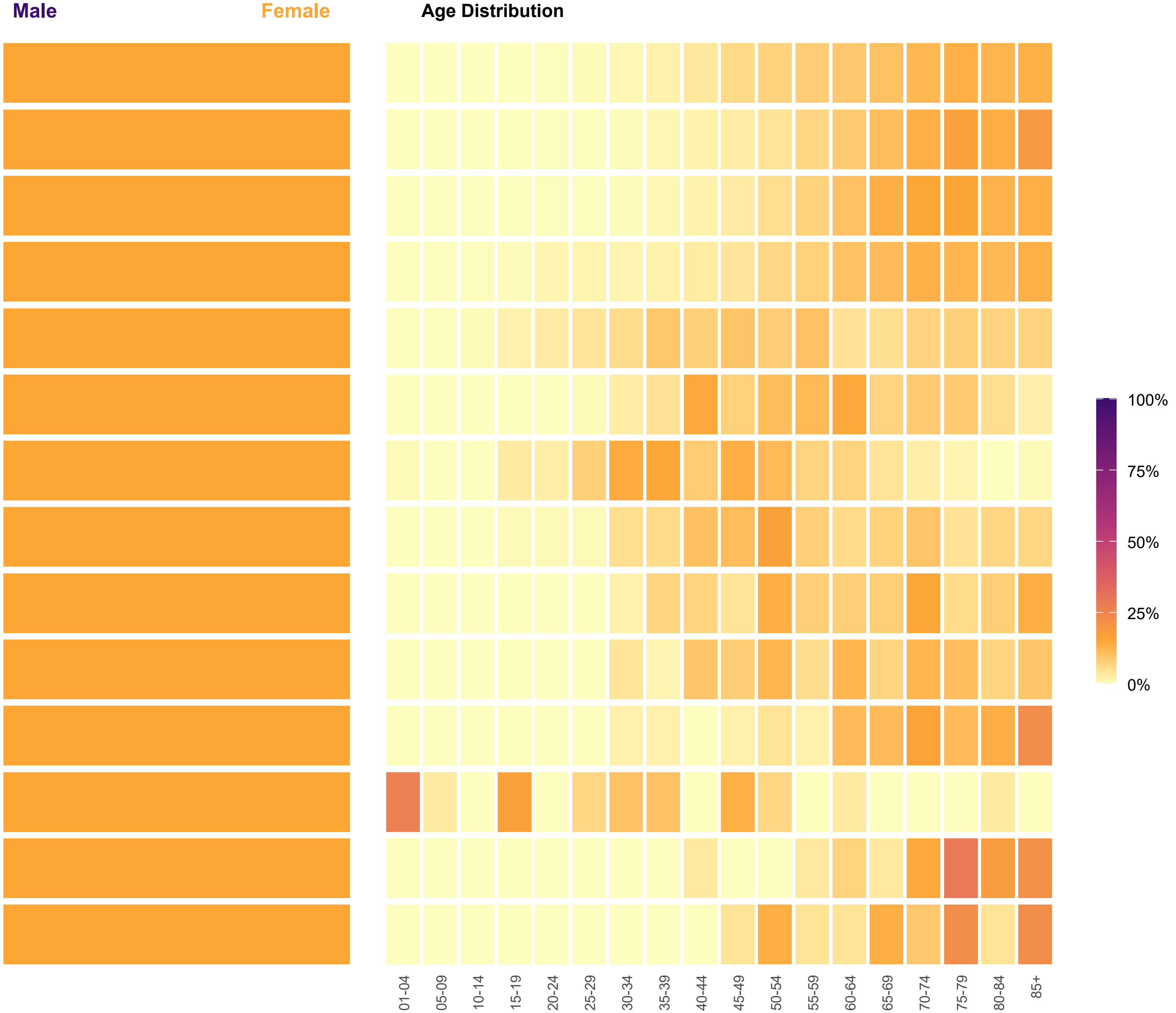

Supplement: Supplementary file 1 — ESM1: Bericht 1: Primärtumoranalyse (gruppierte Morphologie) [file 292_2026_1555_MOESM1_ESM.pdf]
