# Supplementary material for: Pathodashboard: an epidemiological atlas for tumour diagnostics. German version
Source: Pathologie (Heidelb). 2026 Apr 30;47(4):287–94. [Article in German] doi: 10.1007/s00292-026-01555-w (PMC13309514; doi:10.1007/s00292-026-01555-w)

Report 2: Primary Site + Phenotype Analysis (Detailed Morphology)

Table of Contents

Adnexa Other and Genital Female Other - epithelial 6
Adnexa Other and Genital Female Other - Grouped Phenotypes 7
Adrenal Gland - epithelial 8
Adrenal Gland - Grouped Phenotypes 9
Ampulla of Vater - epithelial 10
Ampulla of Vater - Grouped Phenotypes 11
Appendix - epithelial 12
Appendix - Grouped Phenotypes 13
Biliary Other - epithelial 14
Biliary Other - Grouped Phenotypes 15
Bones - Grouped Phenotypes 16
Bones of skull and face and associated joints - epithelial 17
Bones of skull and face and associated joints - Grouped Phen 18
Brain - epithelial 19
Brain - germ cell 20
Brain - hematopoietic 21
Brain - mesenchymal 22
Brain - mixed or multipotent stem cell 23
Brain - neuroectodermal 24
Breast - epithelial 25
Breast - Grouped Phenotypes 26
Buccal Mucosa - epithelial 27
Buccal Mucosa - Grouped Phenotypes 28
Cervix uteri - epithelial 29
Cervix uteri - Grouped Phenotypes 30
CNS Other - Grouped Phenotypes 31
Colon and Rectum (excluding Appendix) - epithelial 32
Colon and Rectum (excluding Appendix) - Grouped Phenotypes 33
Corpus uteri - epithelial 34
Corpus uteri - Grouped Phenotypes 35
Digestive Other - epithelial 36
Digestive Other - Grouped Phenotypes 37
Endocrine Other - epithelial 38
Endocrine Other - Grouped Phenotypes 39
Epididymis - epithelial 40

Report 2: Primary Site + Phenotype Analysis (Detailed Morphology)

Table of Contents

Epididymis - Grouped Phenotypes 41
Esophagus - epithelial 42
Esophagus - Grouped Phenotypes 43
Extrahepatic Bile Ducts - epithelial 44
Extrahepatic Bile Ducts - Grouped Phenotypes 45
Eye and Orbit - epithelial 46
Eye and Orbit - Grouped Phenotypes 47
Fallopian Tube - epithelial 48
Fallopian Tube - Grouped Phenotypes 49
Floor of Mouth - epithelial 50
Floor of Mouth - Grouped Phenotypes 51
Gallbladder - epithelial 52
Gallbladder - Grouped Phenotypes 53
Gum - epithelial 54
Gum - Grouped Phenotypes 55
Heart - epithelial 56
Heart - Grouped Phenotypes 57
Hematopoetic and reticuloendothelial - epithelial 58
Hematopoetic and reticuloendothelial - germ cell 59
Hematopoetic and reticuloendothelial - hematopoietic 60
Hematopoetic and reticuloendothelial - mesenchymal 61
Hematopoetic and reticuloendothelial - mixed or multipotent 62
Hematopoetic and reticuloendothelial - neuroectodermal 63
Hypopharynx - epithelial 64
Hypopharynx - Grouped Phenotypes 65
Intracranial Gland - epithelial 66
Intracranial Gland - Grouped Phenotypes 67
Kidney and Renal Pelvis - epithelial 68
Kidney and Renal Pelvis - Grouped Phenotypes 69
Larynx - epithelial 70
Larynx - Grouped Phenotypes 71
Lesions of anus and anal canal - epithelial 72
Lesions of anus and anal canal - Grouped Phenotypes 73
Lip - epithelial 74
Lip - Grouped Phenotypes 75

Report 2: Primary Site + Phenotype Analysis (Detailed Morphology)

Table of Contents

Liver and Intrahepatic Bile Ducts - epithelial 76
Liver and Intrahepatic Bile Ducts - Grouped Phenotypes 77
Long bones of lower limb and associated joints - epithelial 78
Long bones of lower limb and associated joints - Grouped Phe 79
Long bones of upper limb and scapula and associated joints - 80
Lung and Bronchus - epithelial 81
Lung and Bronchus - Grouped Phenotypes 82
Major Salivary Glands - epithelial 83
Major Salivary Glands - Grouped Phenotypes 84
Mandible - epithelial 85
Mandible - Grouped Phenotypes 86
Meninges - Grouped Phenotypes 87
Mouth Other - epithelial 88
Mouth Other - Grouped Phenotypes 89
Nasal Cavity and Paranasal Sinuses - epithelial 90
Nasal Cavity and Paranasal Sinuses - Grouped Phenotypes 91
Nasopharynx - epithelial 92
Nasopharynx - Grouped Phenotypes 93
Oropharynx - epithelial 94
Oropharynx - Grouped Phenotypes 95
Ovary - epithelial 96
Ovary - germ cell 97
Ovary - hematopoietic 98
Ovary - mesenchymal 99
Ovary - mixed or multipotent stem cell 100
Ovary - neuroectodermal 101
Palate excluding Soft and Uvula - epithelial 102
Palate excluding Soft and Uvula - Grouped Phenotypes 103
Pancreas - epithelial 104
Pancreas - Grouped Phenotypes 105
Parathyroid gland - epithelial 106
Pelvic bones and sacrum and coccyx and associated joints a 107
Penis - epithelial 108
Penis - Grouped Phenotypes 109
Peripheral nerves and autonomic nervous system - epithelial 110

Report 2: Primary Site + Phenotype Analysis (Detailed Morphology)

Table of Contents

Peripheral nerves and autonomic nervous system - Grouped Phe 111
Pharynx and Oral Cavity Other - epithelial 112
Pharynx and Oral Cavity Other - Grouped Phenotypes 113
Placenta - Grouped Phenotypes 114
Prostate - epithelial 115
Prostate - Grouped Phenotypes 116
Retroperitoneum and Peritoneum - epithelial 117
Retroperitoneum and Peritoneum - Grouped Phenotypes 118
Rib and sternum and clavicle and associated joints - Groupe 119
Short bones of lower limb and associated joints - Grouped Ph 120
Short bones of upper limb and associated joints - Grouped Ph 121
Sinus Other - epithelial 122
Sinus Other - Grouped Phenotypes 123
Skin - epithelial 124
Skin - Grouped Phenotypes 125
Small intestine - epithelial 126
Small intestine - Grouped Phenotypes 127
Stomach - epithelial 128
Stomach - Grouped Phenotypes 129
Testis - epithelial 130
Testis - Grouped Phenotypes 131
Thymus - epithelial 132
Thymus - Grouped Phenotypes 133
Thyroid - epithelial 134
Thyroid - Grouped Phenotypes 135
Tongue - epithelial 136
Tongue - Grouped Phenotypes 137
Trachea - epithelial 138
Trachea - Grouped Phenotypes 139
Ureter - epithelial 140
Ureter - Grouped Phenotypes 141
Urethra - epithelial 142
Urethra - Grouped Phenotypes 143
Urinary Bladder - epithelial 144
Urinary Bladder - Grouped Phenotypes 145

Report 2: Primary Site + Phenotype Analysis (Detailed Morphology)

Table of Contents

Urinary Other - epithelial 146
Urinary Other - Grouped Phenotypes 147
Vagina - epithelial 148
Vagina - Grouped Phenotypes 149
Vertebral column - Grouped Phenotypes 150
Vulva - epithelial 151
Vulva - Grouped Phenotypes 152

Primary Site: Adnexa Other and Genital Female Other | Phenotype: epithelial

Top 12 Morphologies | cases: 5,237

Morphology	n	%
Adenocarcinoma	1,929	36.8%
Serous carcinoma	1,115	21.3%
Squamous cell carcinoma	548	10.5%
Endometrioid adenocarcinoma	419	8.0%
Low grade serous carcinoma	359	6.9%
High grade serous carcinoma	233	4.4%
Clear cell adenocarcinoma	128	2.4%
Papillary adenocarcinoma	125	2.4%
Mixed cell adenocarcinoma	87	1.7%
Mucinous adenocarcinoma	81	1.5%
Neuroendocrine carcinoma	33	0.6%
Cystadenocarcinoma	27	0.5%

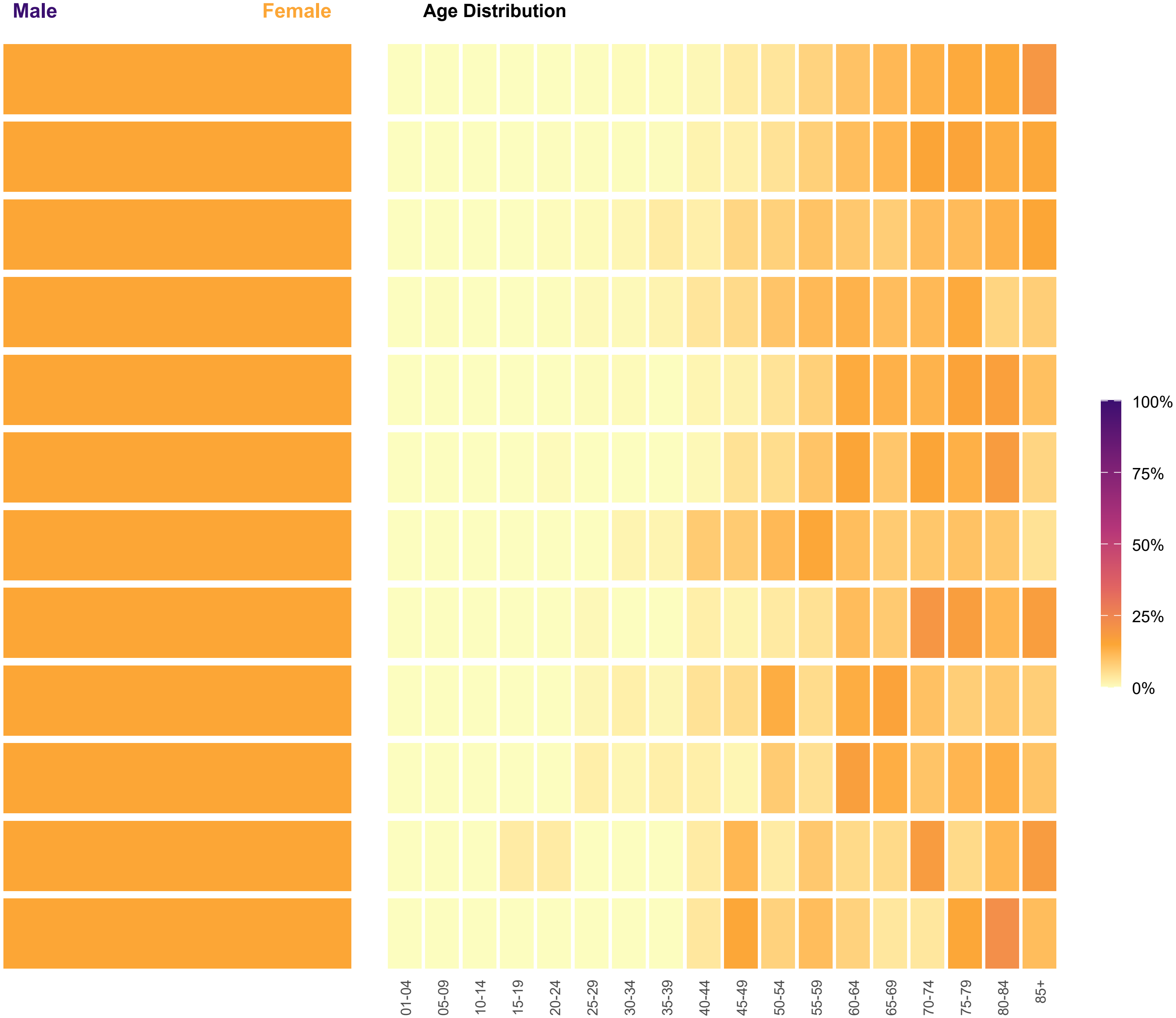

Primary Site: Adnexa Other and Genital Female Other | Phenotype: Grouped Phenotypes

Top 11 Morphologies | cases: 1,068

Morphology	n	%
Mullerian mixed tumor	205	19.2%
Carcinosarcoma	199	18.6%
Leiomyosarcoma	197	18.4%
Diffuse large B-cell lymphoma	43	4.0%
Sarcoma	41	3.8%
Small cell carcinoma	33	3.1%
Adult granulosa cell tumor of ovary	32	3.0%
Choriocarcinoma	31	2.9%
Mesodermal mixed tumor	27	2.5%
Mesonephroma	24	2.2%
Adenosarcoma	23	2.2%

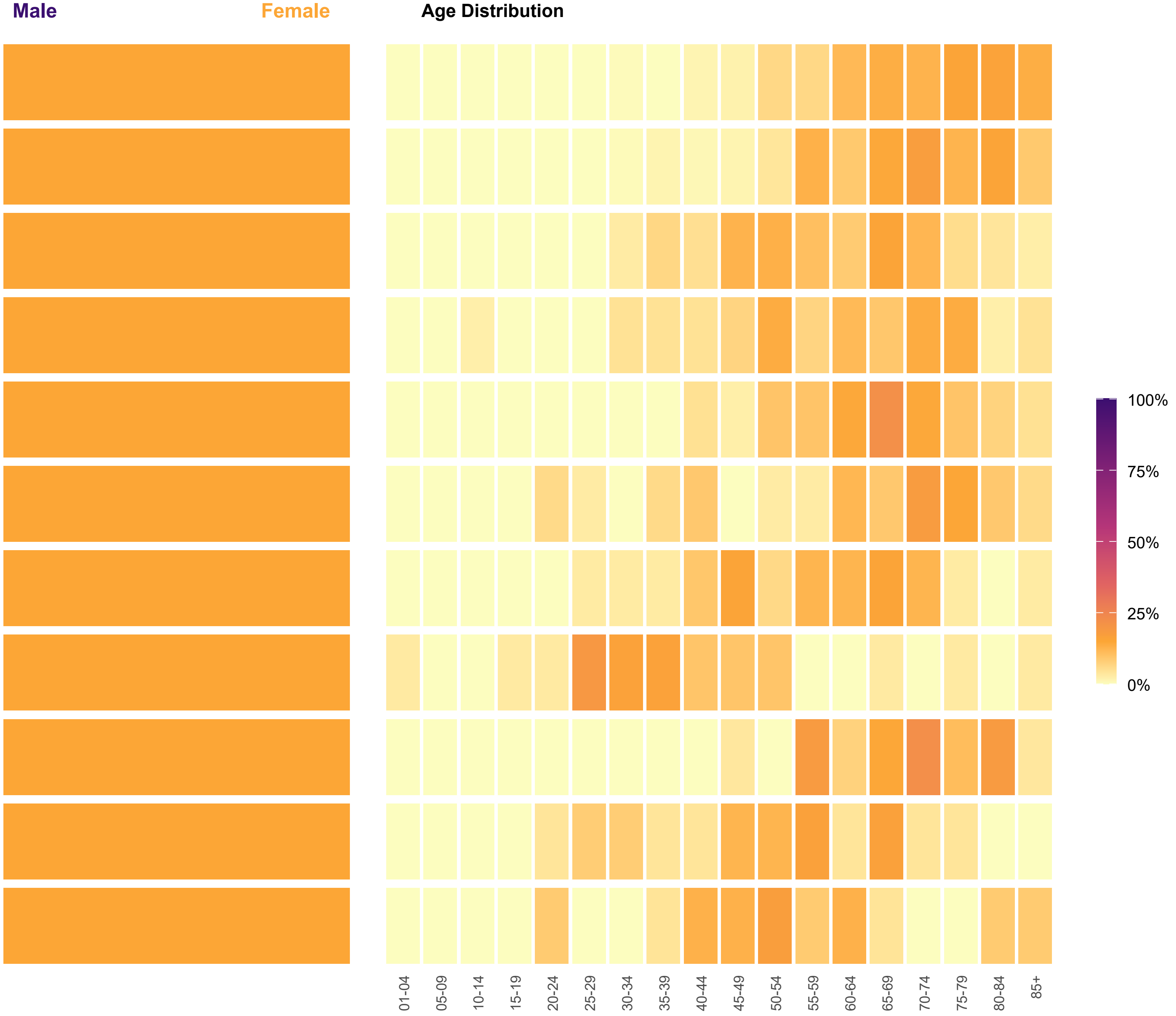

Primary Site: Adrenal Gland | Phenotype: epithelial

Top 5 Morphologies | cases: 5,141

Morphology	n	%
Adrenal cortical carcinoma	4,687	91.2%
Adenocarcinoma	210	4.1%
Neuroendocrine carcinoma	107	2.1%
Oxyphilic adenocarcinoma	42	0.8%
Clear cell adenocarcinoma	36	0.7%

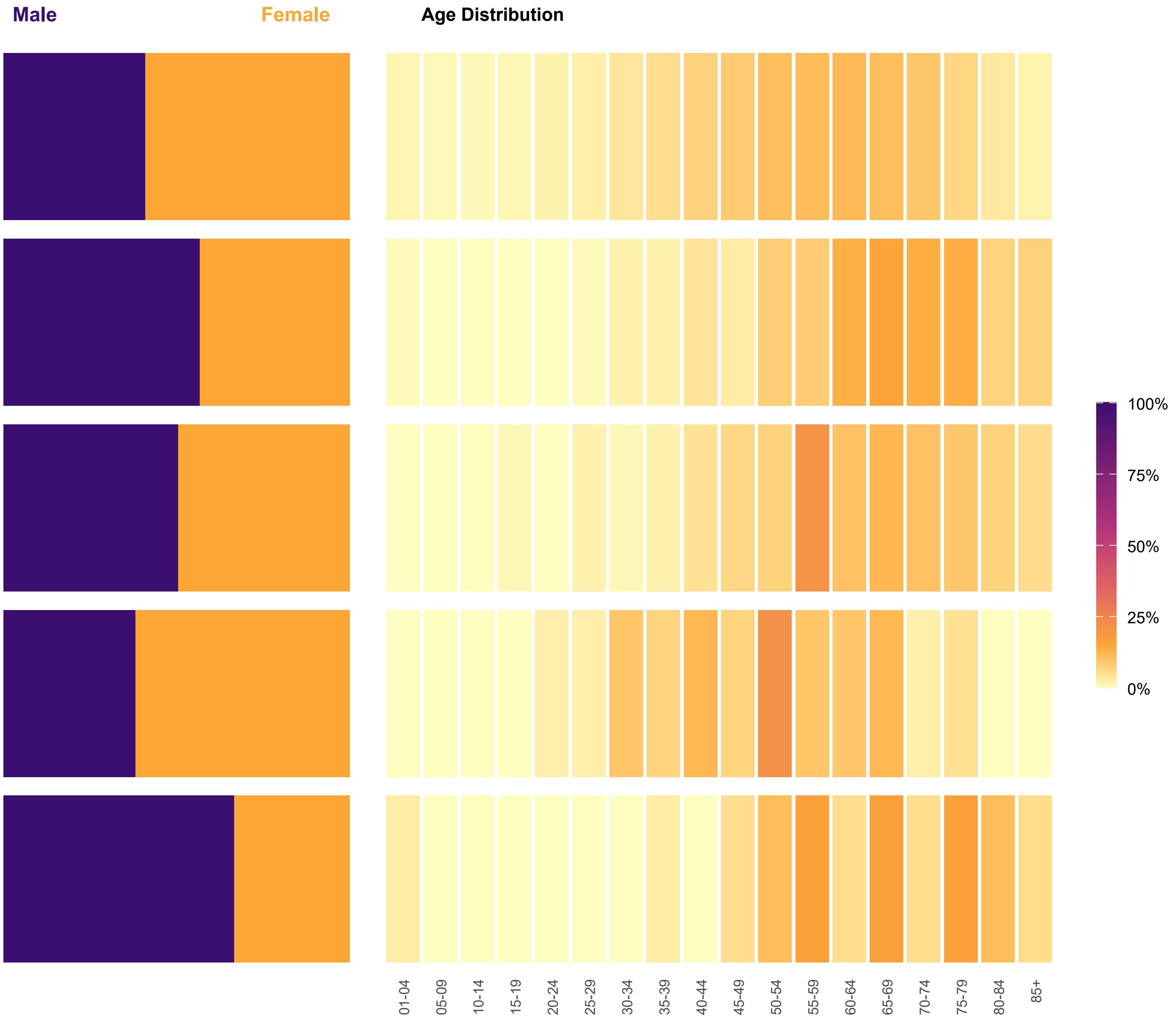

Primary Site: Adrenal Gland | Phenotype: Grouped Phenotypes

Top 10 Morphologies | cases: 5,552

Morphology	n	%
Neuroblastoma	2,527	45.5%
Pheochromocytoma	1,738	31.3%
Diffuse large B-cell lymphoma	342	6.2%
Ganglioneuroblastoma	327	5.9%
Leiomyosarcoma	120	2.2%
Hemangiosarcoma	89	1.6%
lymphoma non-Hodgkin	42	0.8%
Paraganglioma	35	0.6%
Sarcoma	31	0.6%
Small cell carcinoma	23	0.4%

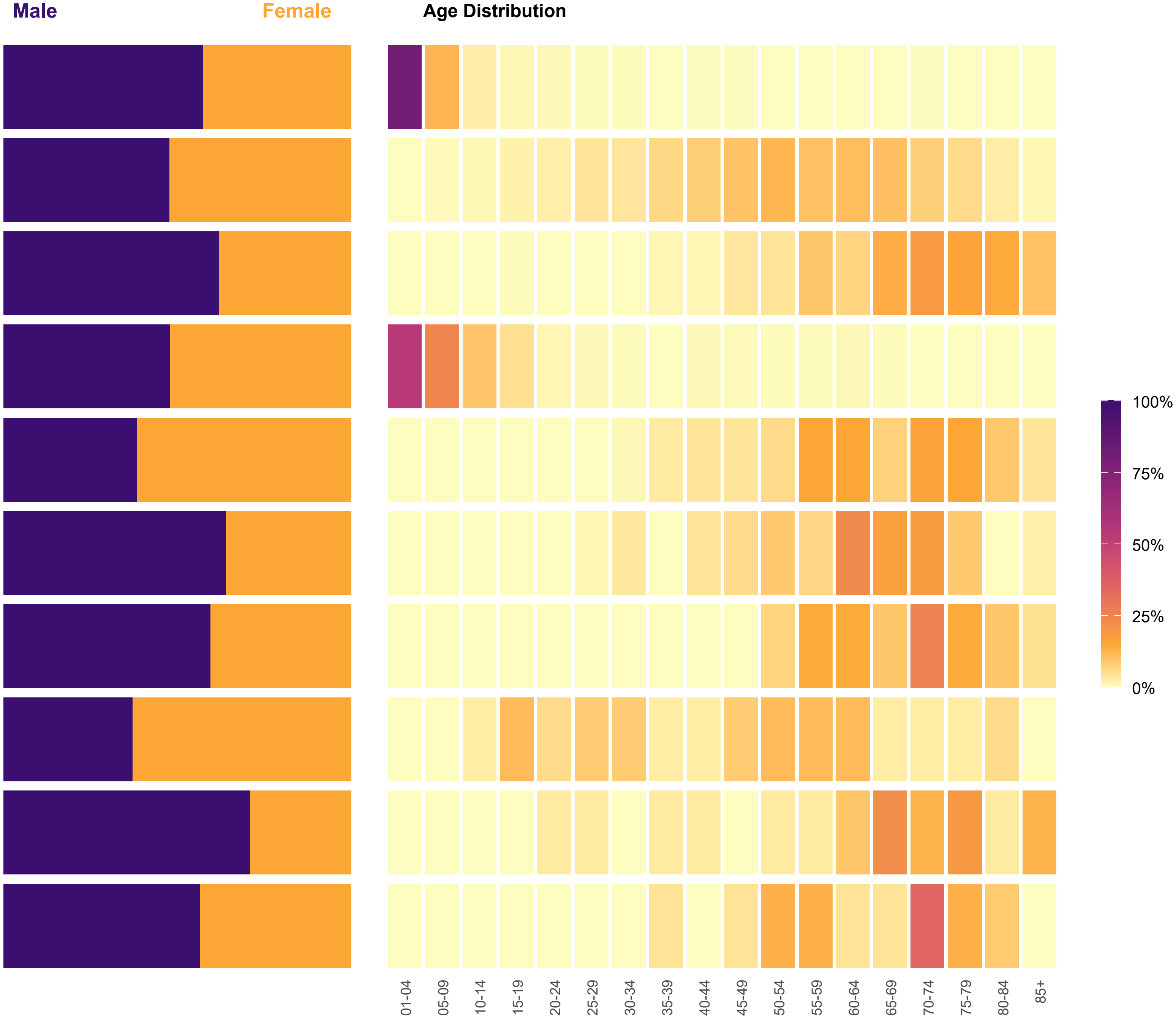

Primary Site: Ampulla of Vater | Phenotype: epithelial

Top 17 Morphologies | cases: 26,911

Morphology	n	%
Adenocarcinoma	20,323	75.5%
Adenocarcinoma in tubulovillous…	913	3.4%
Adenocarcinoma intestinal type	806	3.0%
Mucinous adenocarcinoma	758	2.8%
Pancreatobiliary type carcinoma	599	2.2%
Infiltrating duct carcinoma	509	1.9%
Papillary adenocarcinoma	409	1.5%
Neuroendocrine carcinoma	401	1.5%
Signet ring cell carcinoma	397	1.5%
Adenocarcinoma in villous adenoma	375	1.4%
Mucin-producing adenocarcinoma	295	1.1%
Adenocarcinoma in adenomatous polyp	289	1.1%
Tubular adenocarcinoma	270	1.0%
Cholangiocarcinoma	206	0.8%
Adenocarcinoma with mixed subtypes	193	0.7%
Adenocarcinoma with neuroendocrine…	38	0.1%
Squamous cell carcinoma	30	0.1%

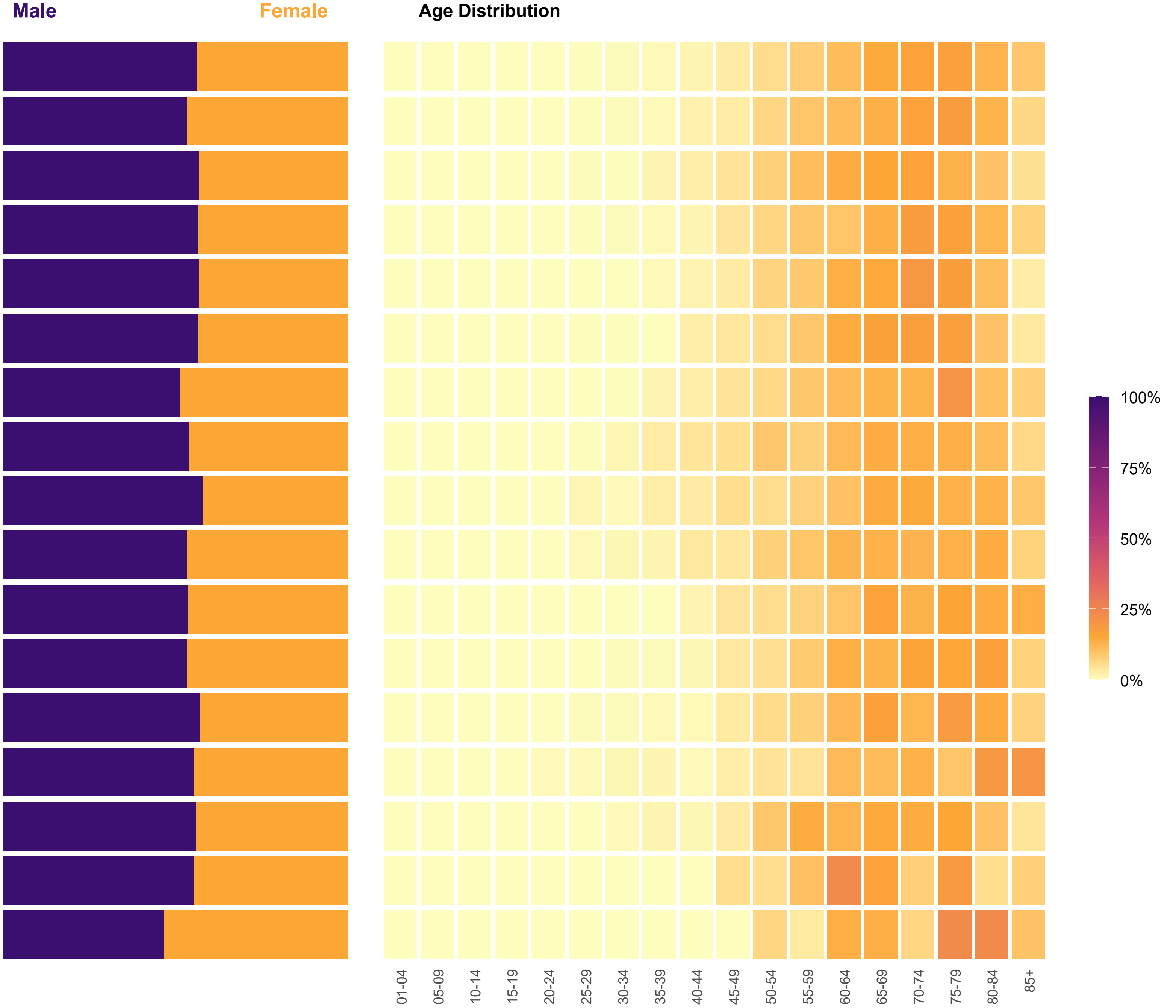

Primary Site: Ampulla of Vater | Phenotype: Grouped Phenotypes

Top 6 Morphologies | cases: 915

Morphology	n	%
Neuroendocrine tumor	528	57.7%
Small cell carcinoma	92	10.1%
Mixed adenoneuroendocrine carcinoma	61	6.7%
Large cell neuroendocrine carcinoma	49	5.4%
Neuroendocrine tumor grade 2	49	5.4%
Diffuse large B-cell lymphoma	26	2.8%

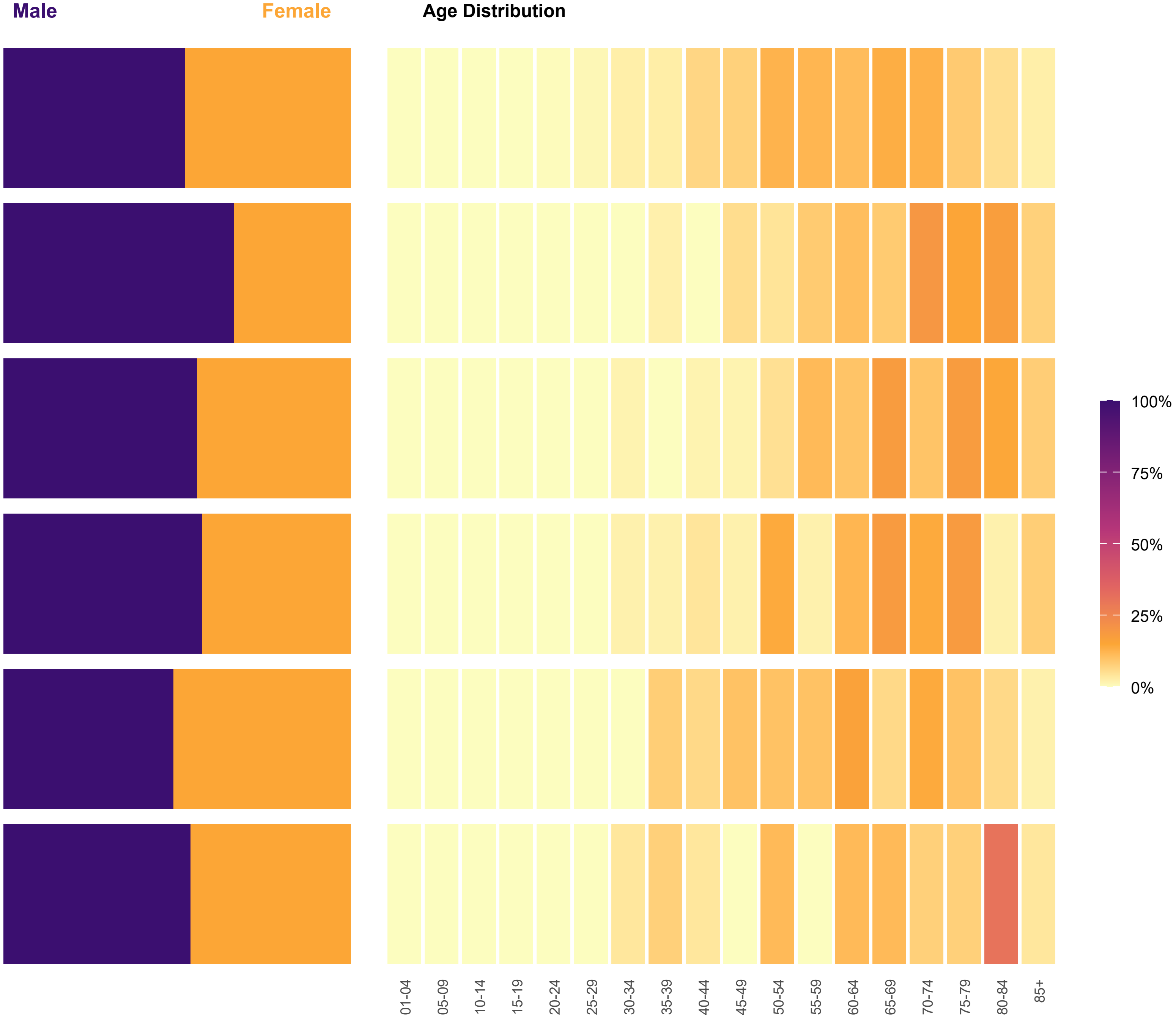

Primary Site: Appendix | Phenotype: epithelial

Top 15 Morphologies | cases: 22,759

Morphology	n	%
Mucinous adenocarcinoma	9,208	40.5%
Adenocarcinoma	6,871	30.2%
Neuroendocrine carcinoma	2,273	10.0%
Signet ring cell carcinoma	1,785	7.8%
Mucin-producing adenocarcinoma	587	2.6%
Mucinous cystadenocarcinoma	498	2.2%
Adenocarcinoma in tubulovillous…	412	1.8%
Adenocarcinoma with mixed subtypes	307	1.3%
Adenocarcinoma in villous adenoma	252	1.1%
Adenocarcinoma in adenomatous polyp	184	0.8%
Adenocarcinoma with neuroendocrine…	128	0.6%
Cystadenocarcinoma	78	0.3%
Tubular adenocarcinoma	64	0.3%
Adenocarcinoma intestinal type	34	0.1%
Papillary adenocarcinoma	24	0.1%

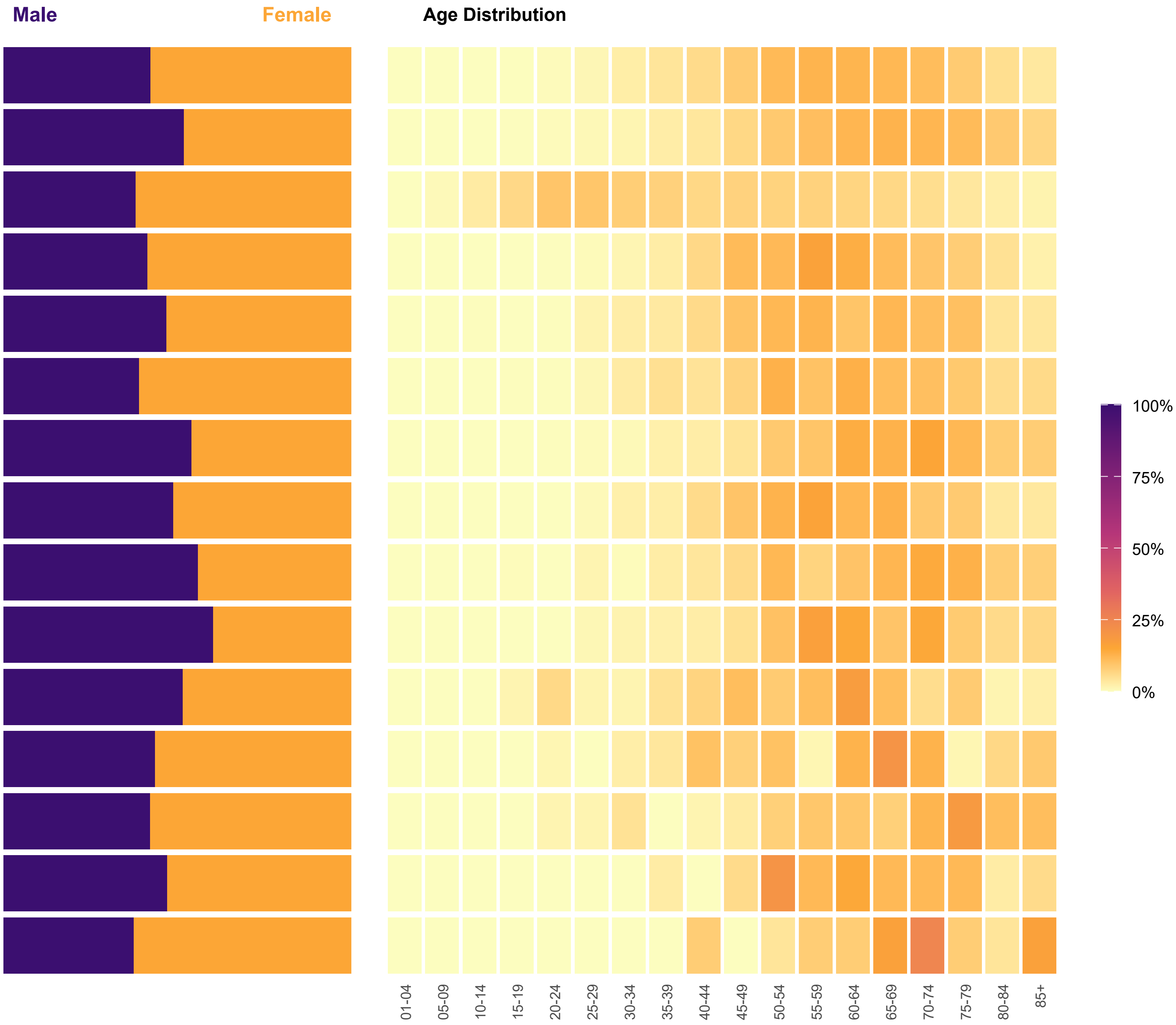

Primary Site: Appendix | Phenotype: Grouped Phenotypes

Top 12 Morphologies | cases: 18,647

Morphology	n	%
Neuroendocrine tumor	11,869	63.7%
Goblet cell carcinoid	3,934	21.1%
Mixed adenoneuroendocrine carcinoma	1,310	7.0%
Adenocarcinoid tumor	645	3.5%
Neuroendocrine tumor grade 2	383	2.1%
Diffuse large B-cell lymphoma	120	0.6%
Enterochromaffin cell carcinoid	91	0.5%
Burkitt lymphoma	56	0.3%
Gastrointestinal stromal tumor	43	0.2%
Large cell neuroendocrine carcinoma	28	0.2%
Marginal zone B-cell lymphoma	25	0.1%
Mantle cell lymphoma	21	0.1%

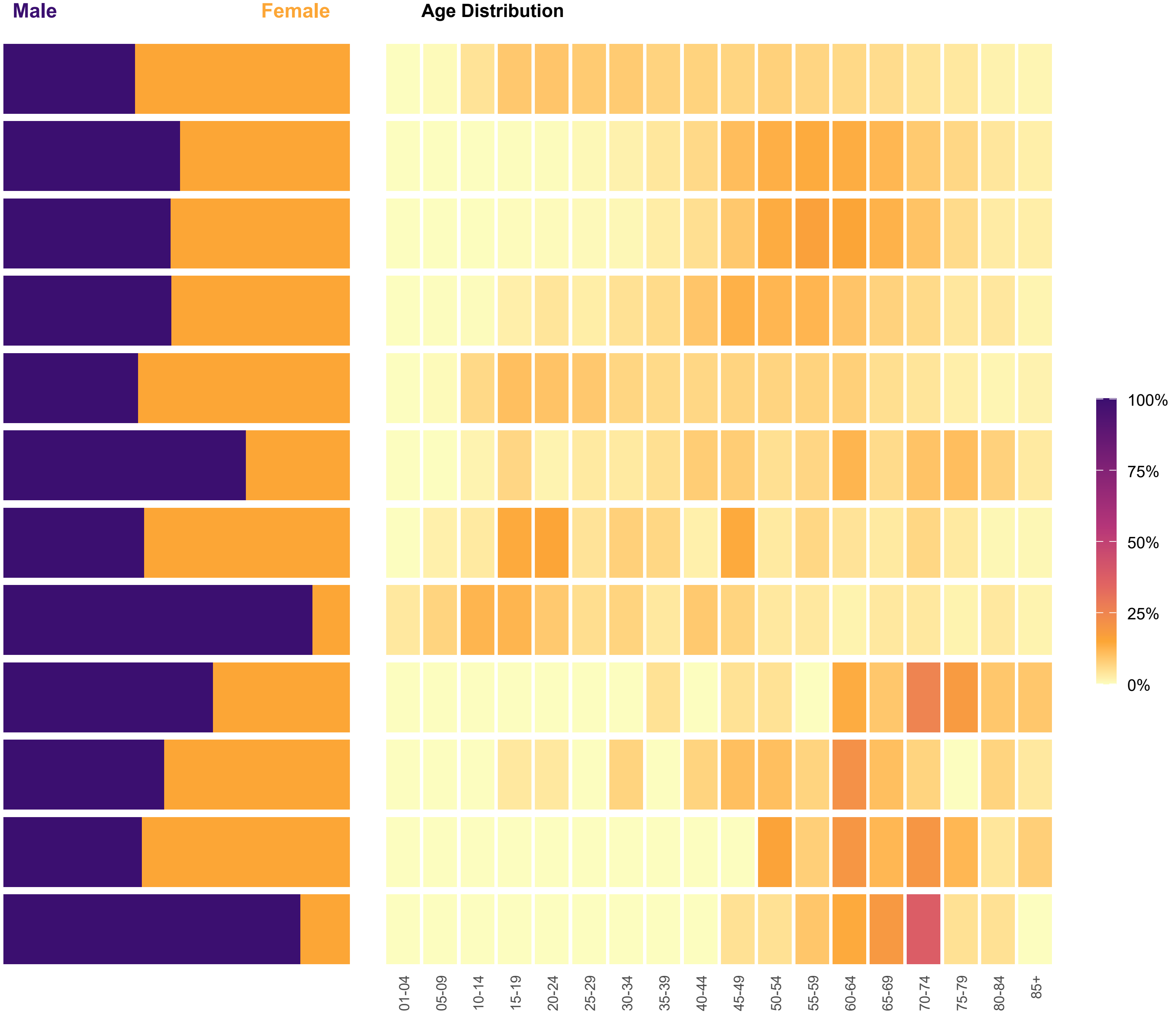

Primary Site: Biliary Other | Phenotype: epithelial

Top 15 Morphologies | cases: 10,005

Morphology	n	%
Adenocarcinoma	6,154	61.5%
Cholangiocarcinoma	2,873	28.7%
Infiltrating duct carcinoma	194	1.9%
Mucinous adenocarcinoma	149	1.5%
Mucin-producing adenocarcinoma	123	1.2%
Tubular adenocarcinoma	83	0.8%
Klatskin tumor	79	0.8%
Neuroendocrine carcinoma	45	0.4%
Papillary adenocarcinoma	43	0.4%
Signet ring cell carcinoma	43	0.4%
Squamous cell carcinoma	36	0.4%
Pancreatobiliary type carcinoma	34	0.3%
Combined hepatocellular carcinoma…	28	0.3%
Bile duct cystadenocarcinoma	21	0.2%
Adenocarcinoma in tubulovillous…	20	0.2%

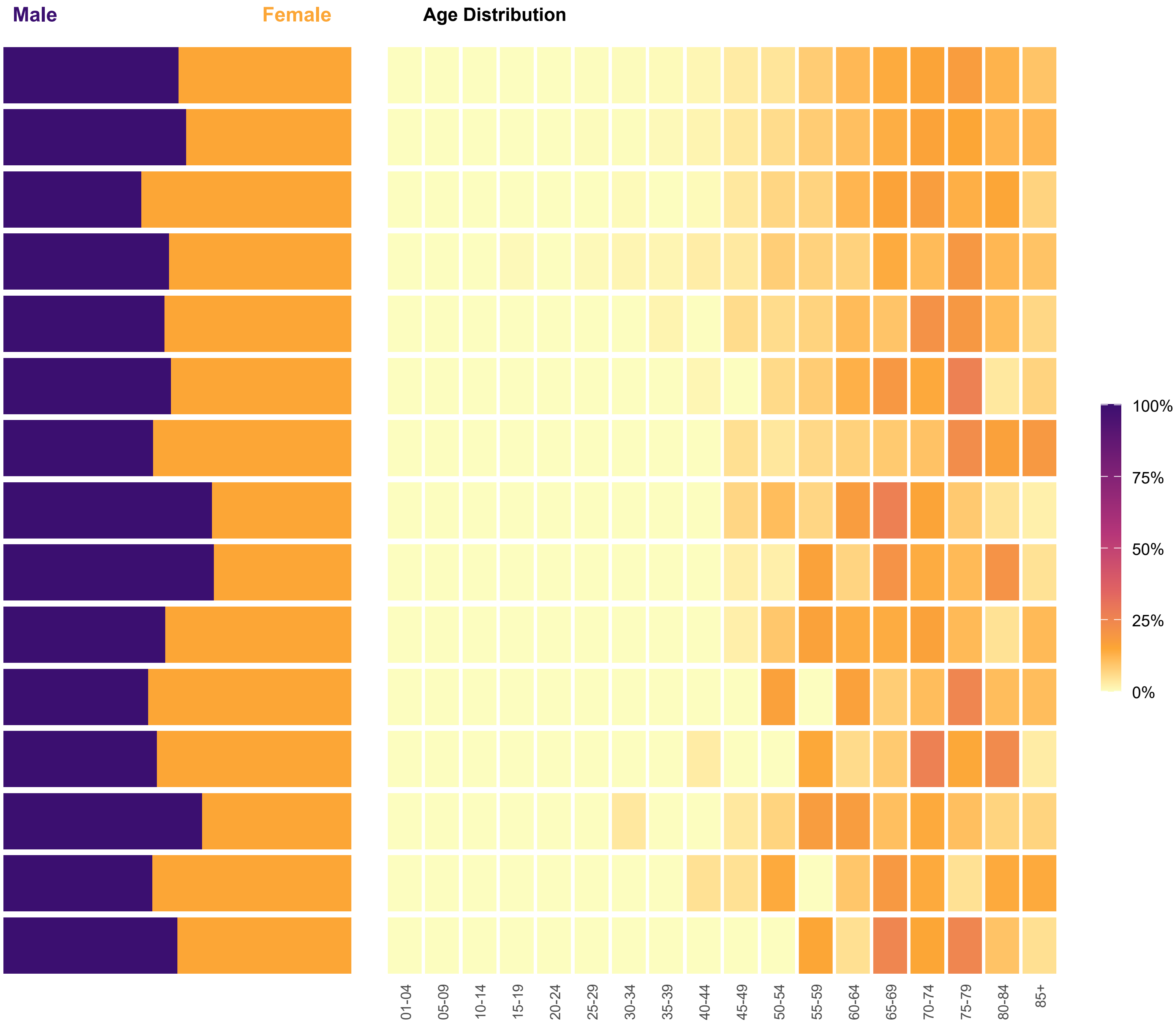

Primary Site: Biliary Other | Phenotype: Grouped Phenotypes

Top 1 Morphologies | cases: 44

Morphology	n	%	Male	Female	Age Distribution
Small cell carcinoma	20	45.5%			

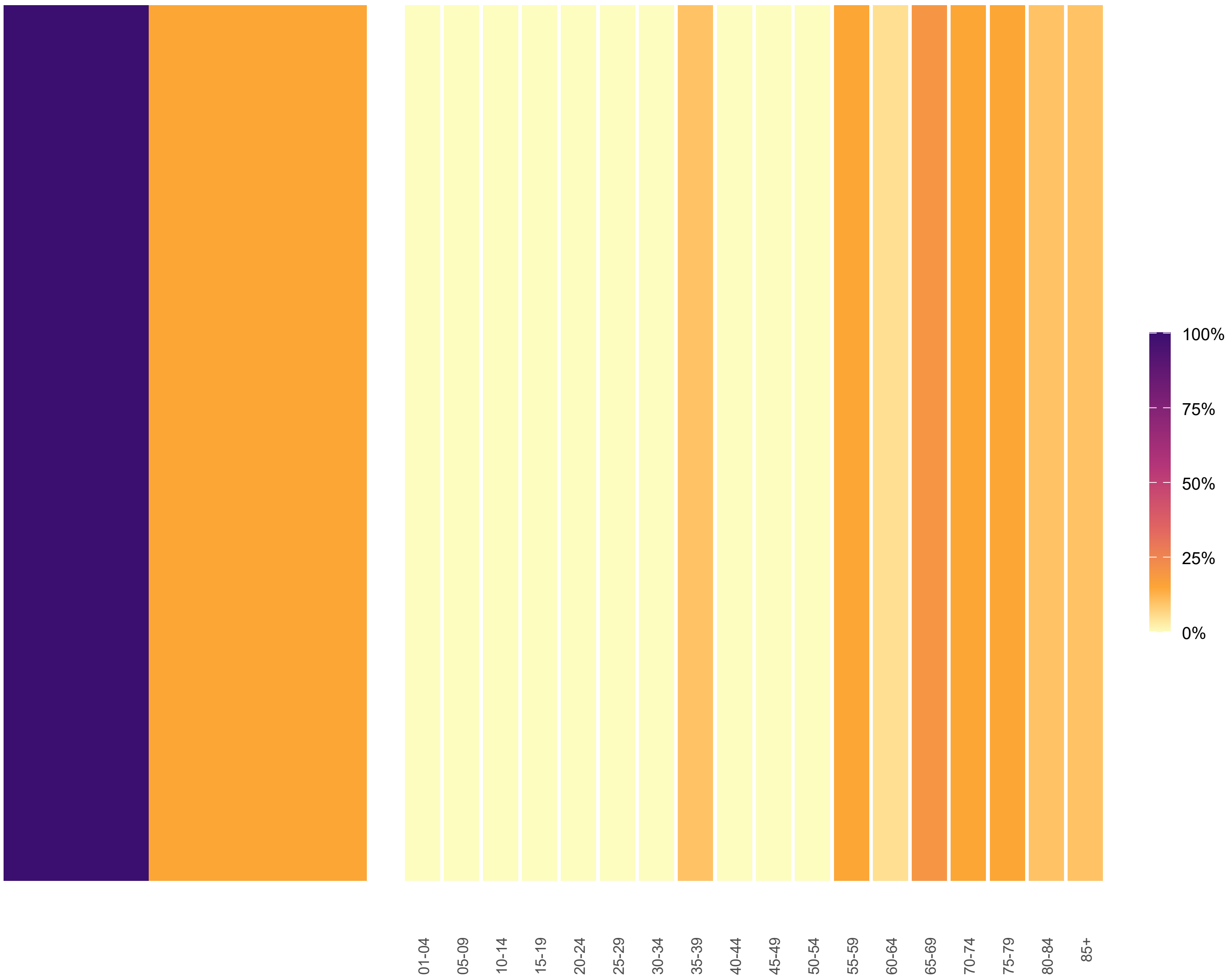

Primary Site: Bones | Phenotype: Grouped Phenotypes

Top 15 Morphologies | cases: 3,055

Morphology	n	%
Chondrosarcoma	597	19.5%
Osteosarcoma	590	19.3%
Plasmacytoma	580	19.0%
Diffuse large B-cell lymphoma	321	10.5%
Langerhans cell histiocytosis…	188	6.2%
Sarcoma	96	3.1%
Chordoma	64	2.1%
Myxoid chondrosarcoma	63	2.1%
lymphoma non-Hodgkin	57	1.9%
Giant cell tumor of bone malignant	44	1.4%
Chondroblastic osteosarcoma	36	1.2%
Follicular lymphoma	34	1.1%
Hemangiosarcoma	27	0.9%
Plasma cell myeloma	21	0.7%
Mesenchymal chondrosarcoma	20	0.7%

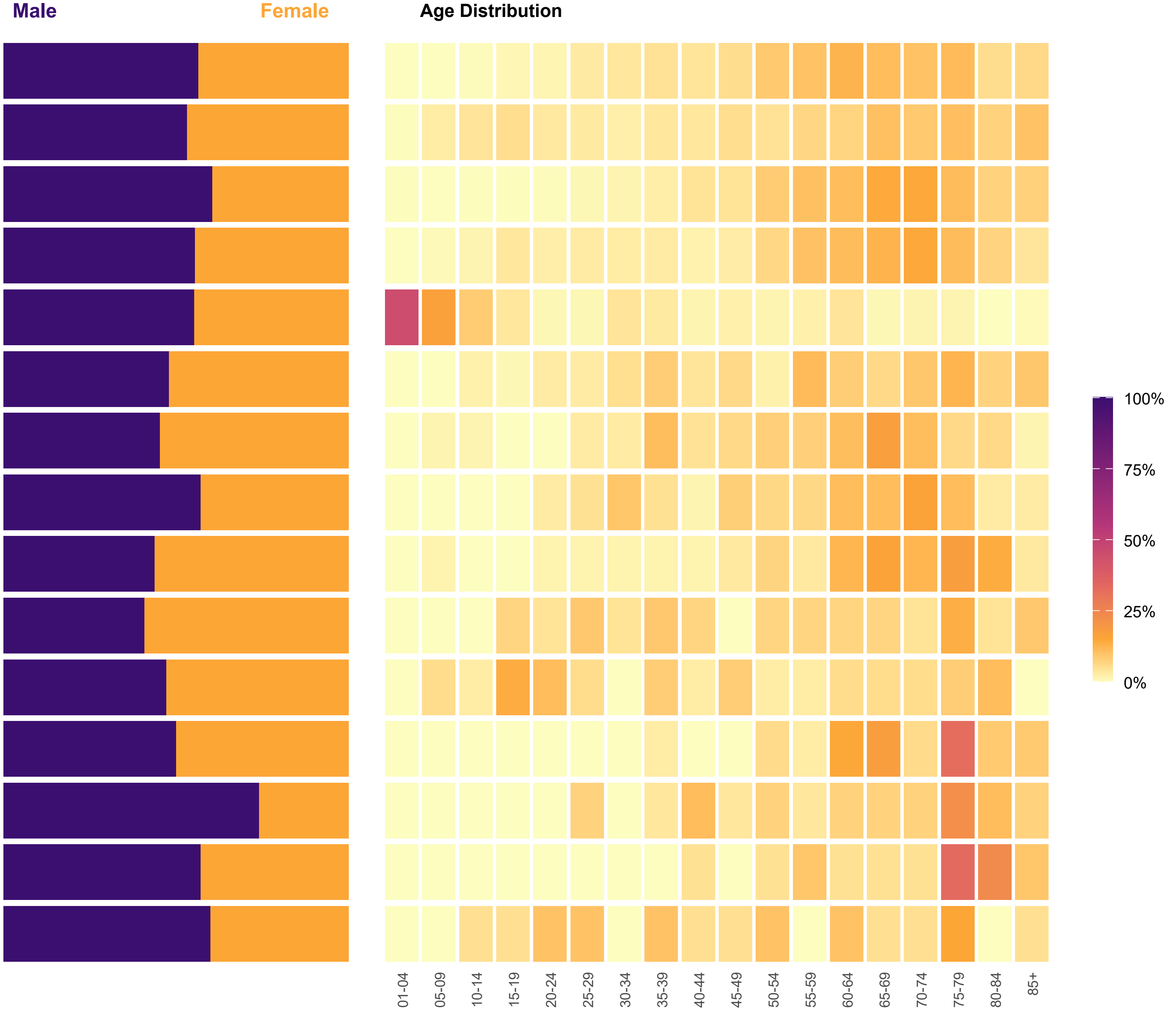

Primary Site: Bones of skull and face and associated joints | Phenotype: epithelial

Top 3 Morphologies | cases: 287

Morphology	n	%
Ameloblastoma metastasizing	106	36.9%
Squamous cell carcinoma	84	29.3%
Odontogenic tumor	80	27.9%

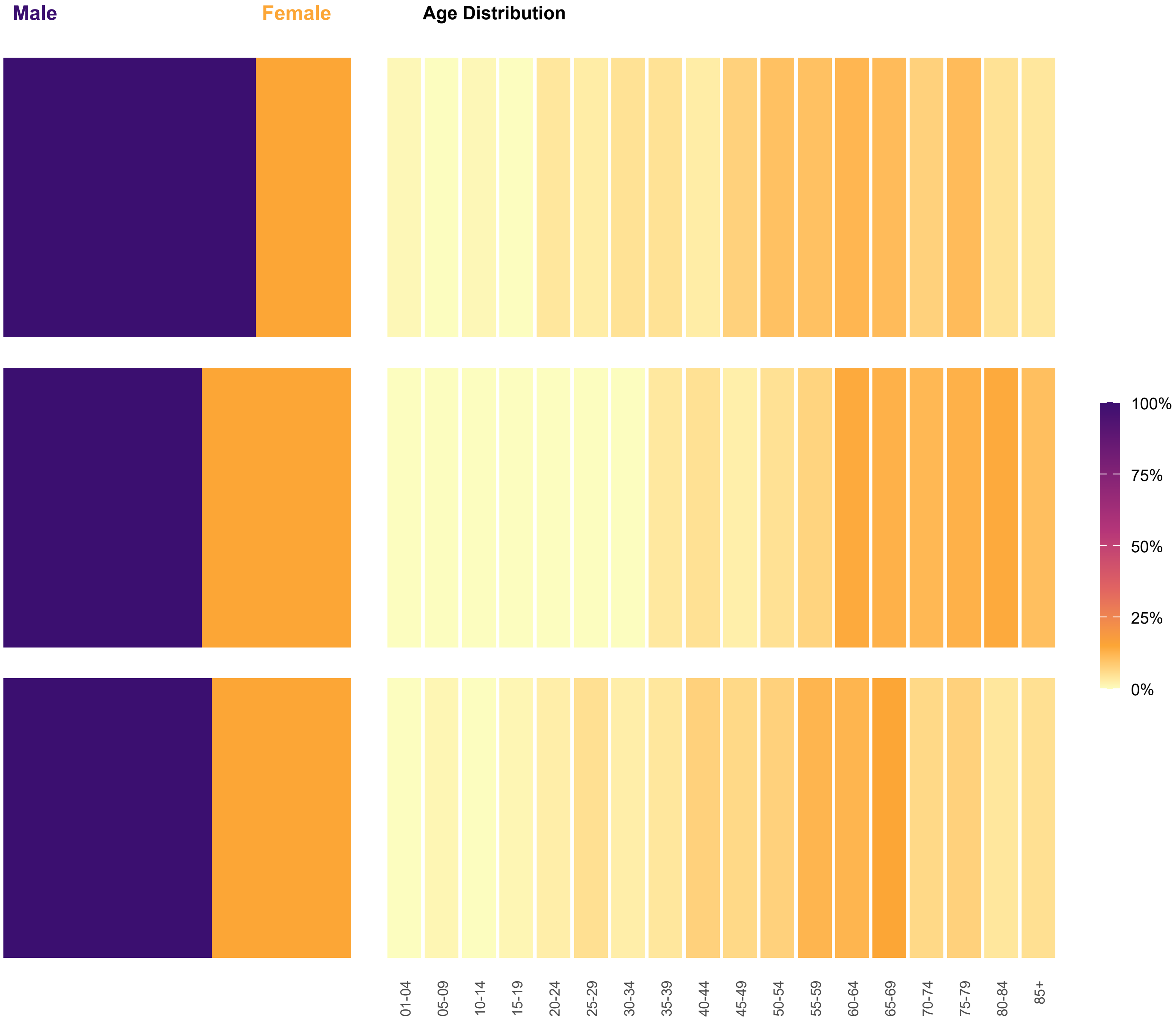

Primary Site: Bones of skull and face and associated joints | Phenotype: Grouped Phenotypes

Top 17 Morphologies | cases: 4,538

Morphology	n	%
Chordoma	1,112	24.5%
Chondrosarcoma	731	16.1%
Langerhans cell histiocytosis…	669	14.7%
Osteosarcoma	465	10.2%
Plasmacytoma	436	9.6%
Diffuse large B-cell lymphoma	199	4.4%
Chondroblastic osteosarcoma	143	3.2%
Chondroid chordoma	120	2.6%
Myxoid chondrosarcoma	98	2.2%
Sarcoma	60	1.3%
Mesenchymal chondrosarcoma	44	1.0%
Fibroblastic osteosarcoma	29	0.6%
Spindle cell sarcoma	28	0.6%
Ewing sarcoma	26	0.6%
lymphoma non-Hodgkin	25	0.6%
Giant cell tumor of bone malignant	21	0.5%
Fibrosarcoma	20	0.4%

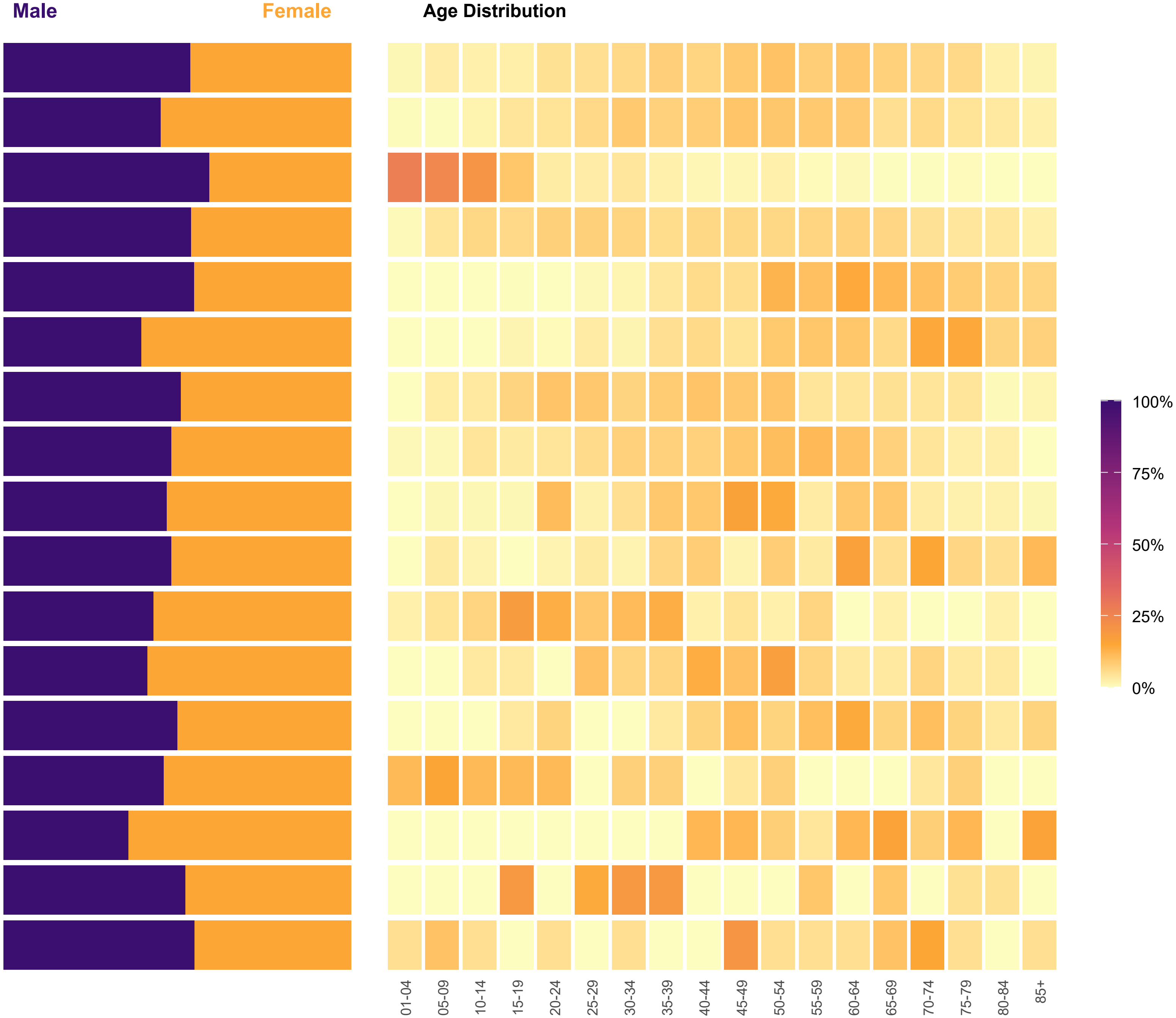

Primary Site: Brain | Phenotype: epithelial

Top 2 Morphologies | cases: 292

Morphology	n	%
Choroid plexus carcinoma	241	82.5%
Squamous cell carcinoma	21	7.2%

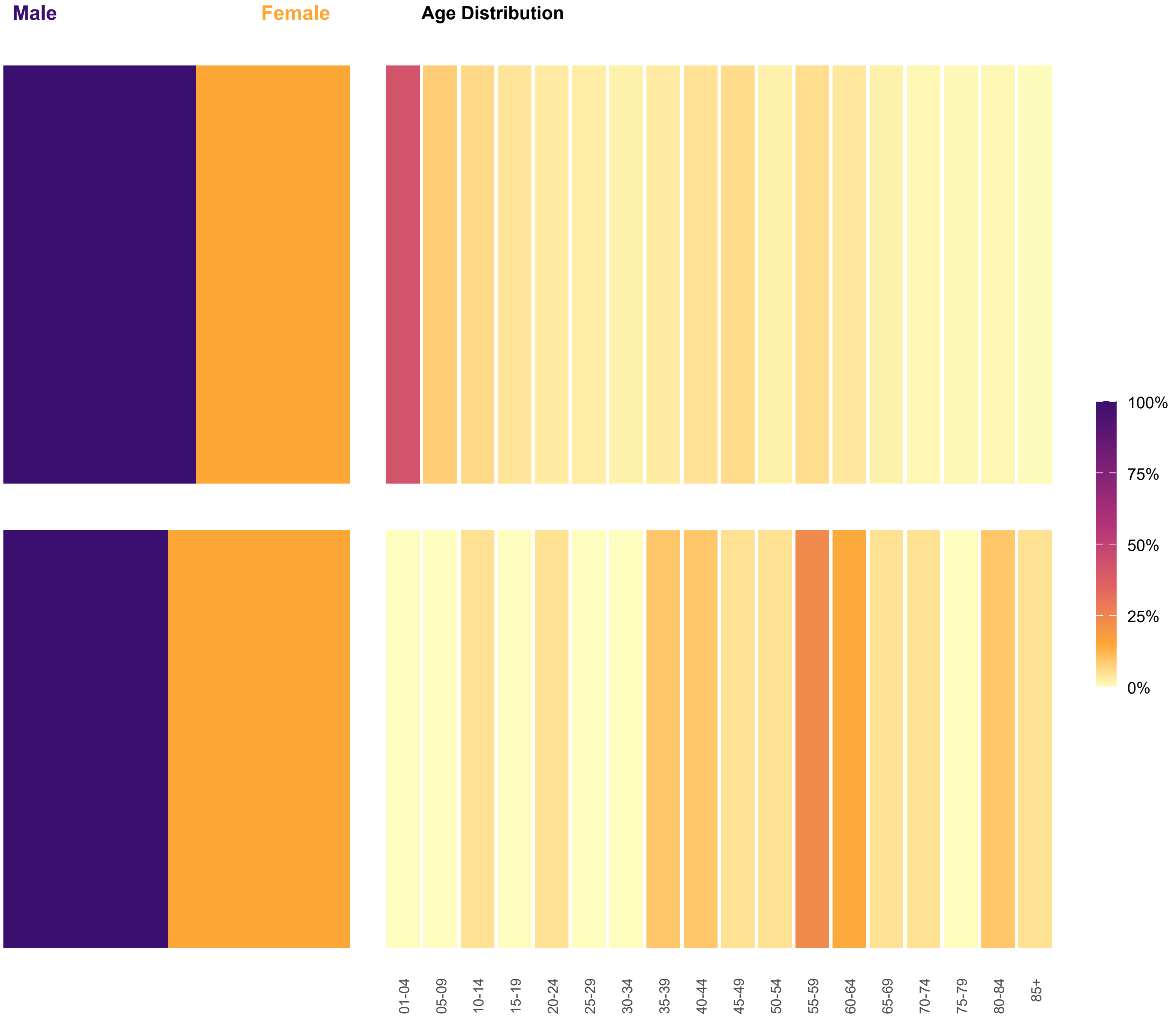

Primary Site: Brain | Phenotype: germ cell

Top 4 Morphologies | cases: 1,079

Morphology	n	%
Germinoma	849	78.7%
Mixed germ cell tumor	123	11.4%
Embryonal carcinoma	31	2.9%
Yolk sac tumor	23	2.1%

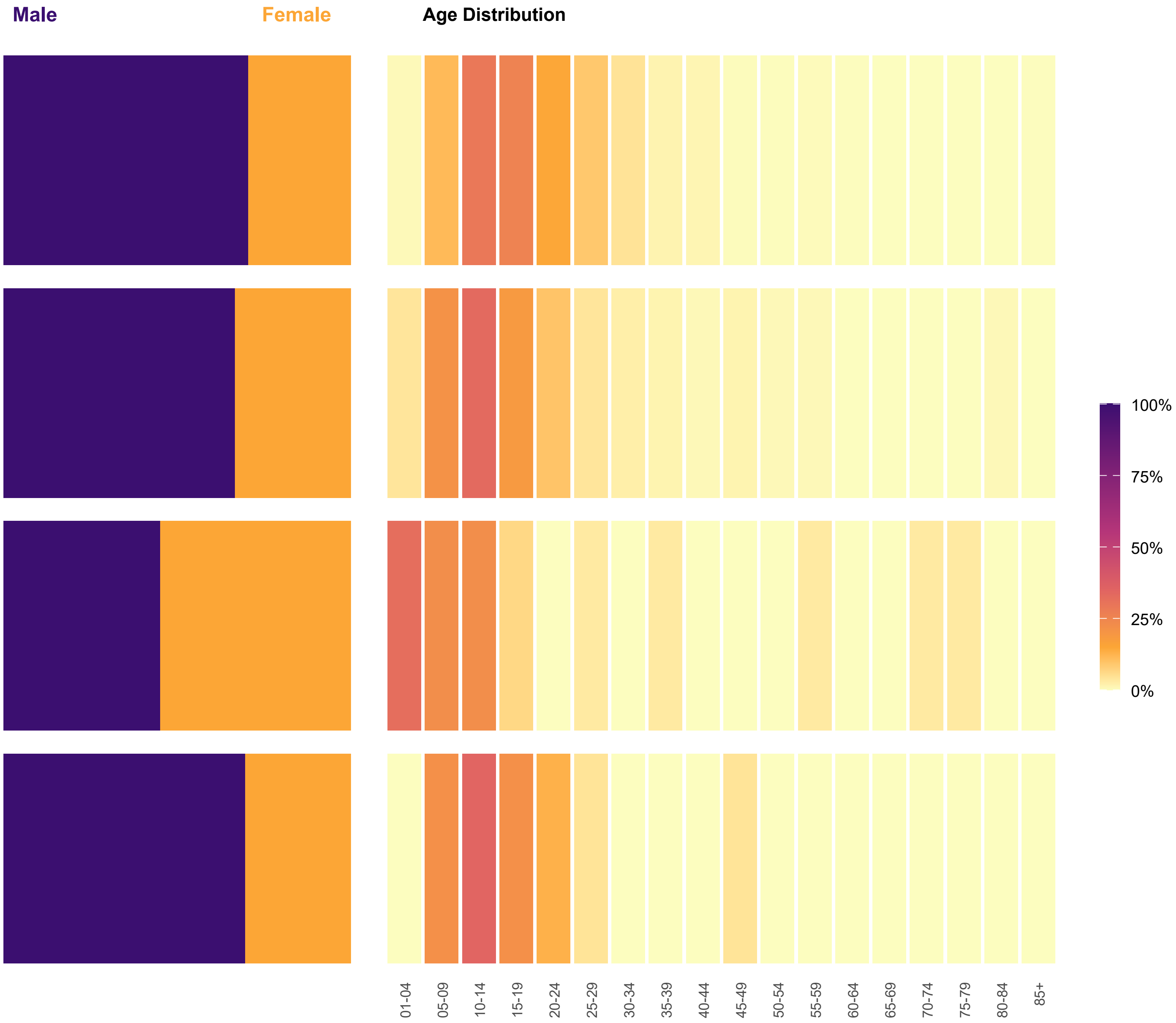

Primary Site: Brain | Phenotype: hematopoietic

Top 16 Morphologies | cases: 10,754

Morphology	n	%
Diffuse large B-cell lymphoma	7,889	73.4%
lymphoma	1,160	10.8%
lymphoma non-Hodgkin	903	8.4%
Marginal zone B-cell lymphoma	133	1.2%
Mature T-cell lymphoma	104	1.0%
Lymphoma large B-cell diffuse…	64	0.6%
Burkitt lymphoma	58	0.5%
Anaplastic large cell lymphoma…	54	0.5%
Langerhans cell histiocytosis…	48	0.4%
Follicular lymphoma	43	0.4%
Lymphoplasmacytic lymphoma	35	0.3%
Plasmacytoma extramedullary	33	0.3%
Follicular lymphoma grade 3	25	0.2%
Intravascular large B-cell lymphoma	23	0.2%
Histiocytic sarcoma	22	0.2%
Follicular lymphoma grade 1/2	21	0.2%

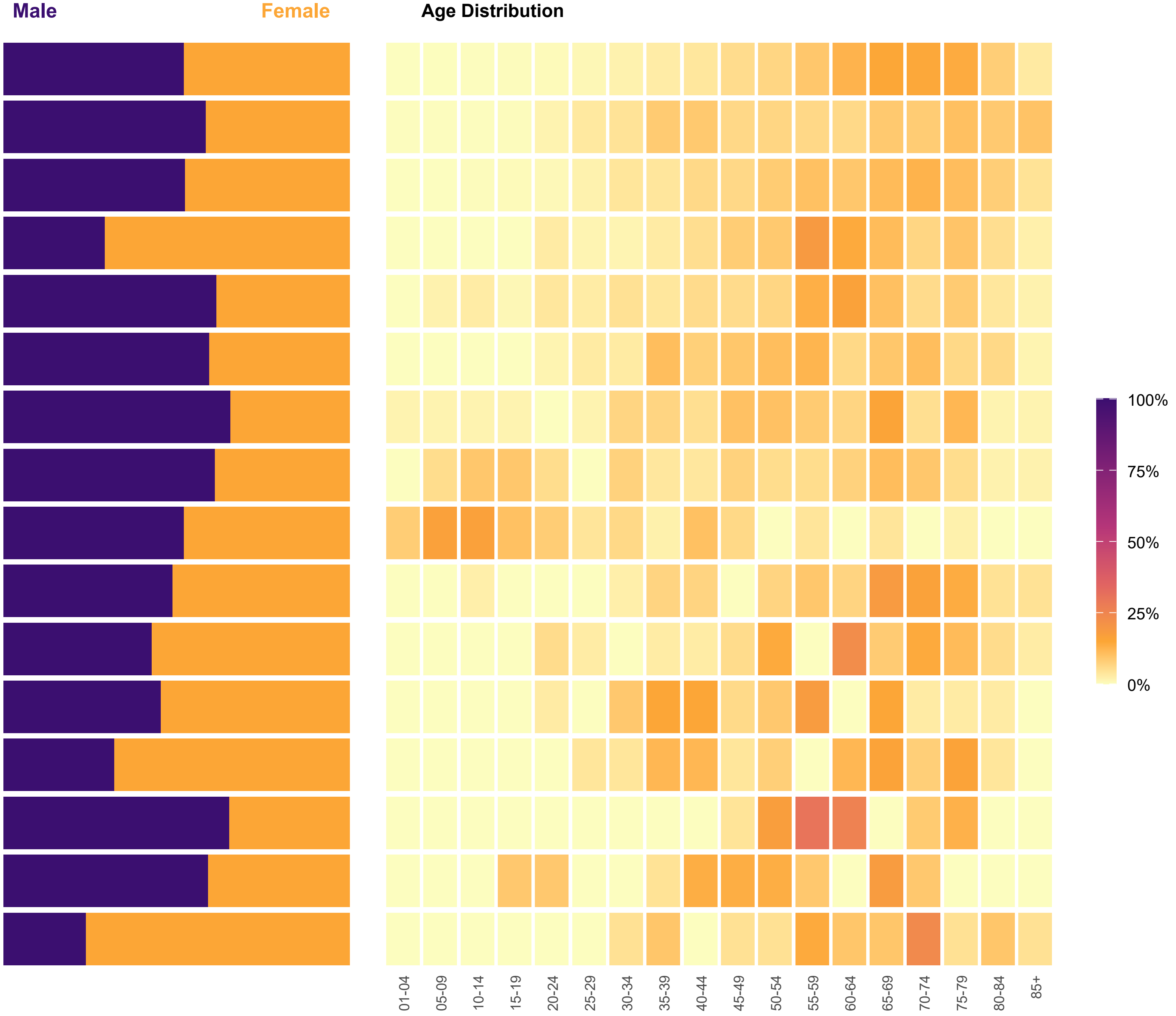

Primary Site: Brain | Phenotype: mesenchymal

Top 9 Morphologies | cases: 787

Morphology	n	%
Meningiomamalignant	249	31.6%
Sarcoma	93	11.8%
Solitary fibrous tumormalignant	89	11.3%
Spindle cell sarcoma	43	5.5%
Hemangiosarcoma	41	5.2%
Ewing sarcoma	35	4.4%
Fibrosarcoma	26	3.3%
Undifferentiated sarcoma	21	2.7%
Chondrosarcoma	20	2.5%

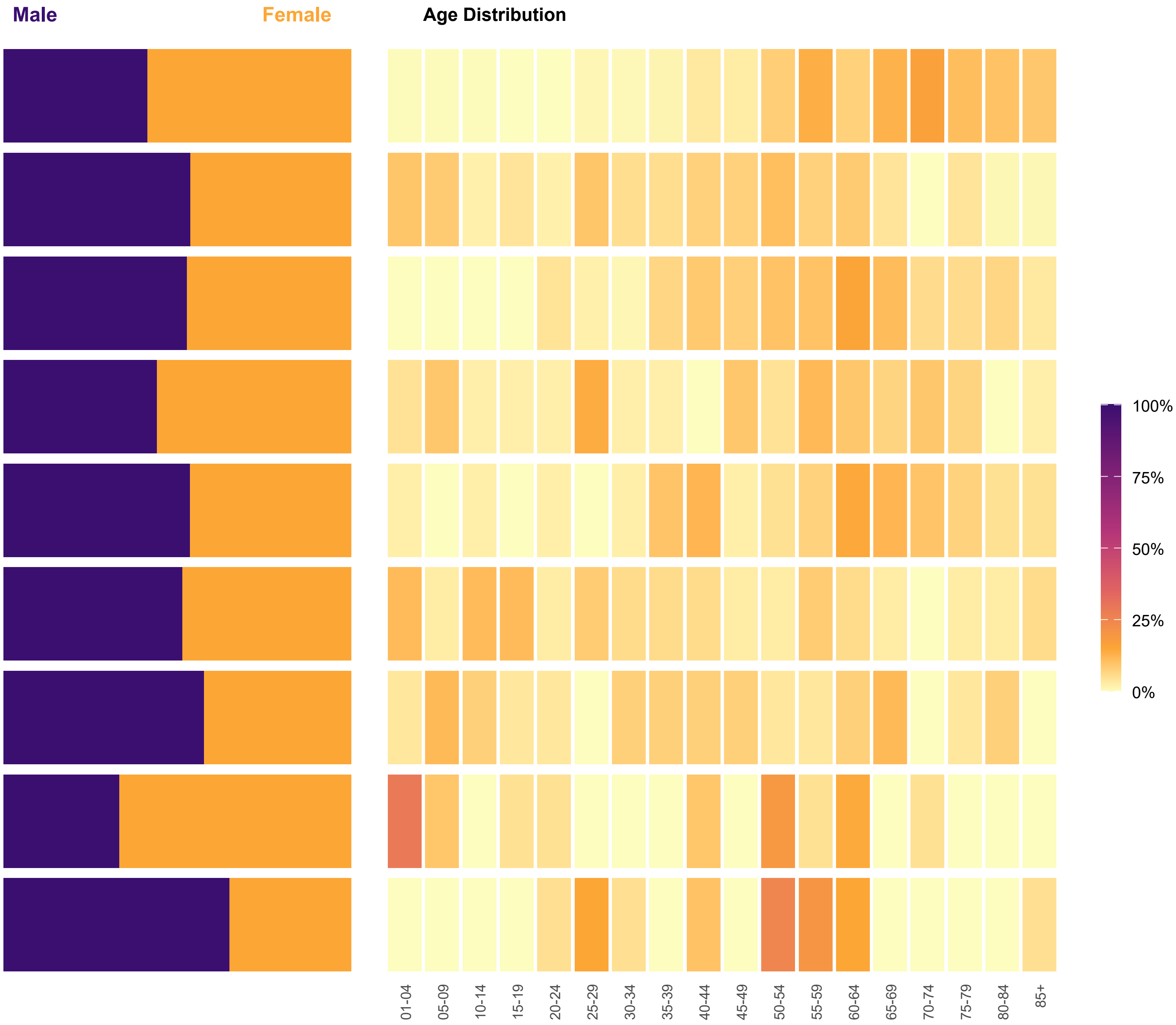

Primary Site: Brain | Phenotype: mixed or multipotent stem cell

Top 3 Morphologies | cases: 336

Morphology	n	%
Chordoma	195	58.0%
Teratoma	49	14.6%
Rhabdoid tumor	43	12.8%

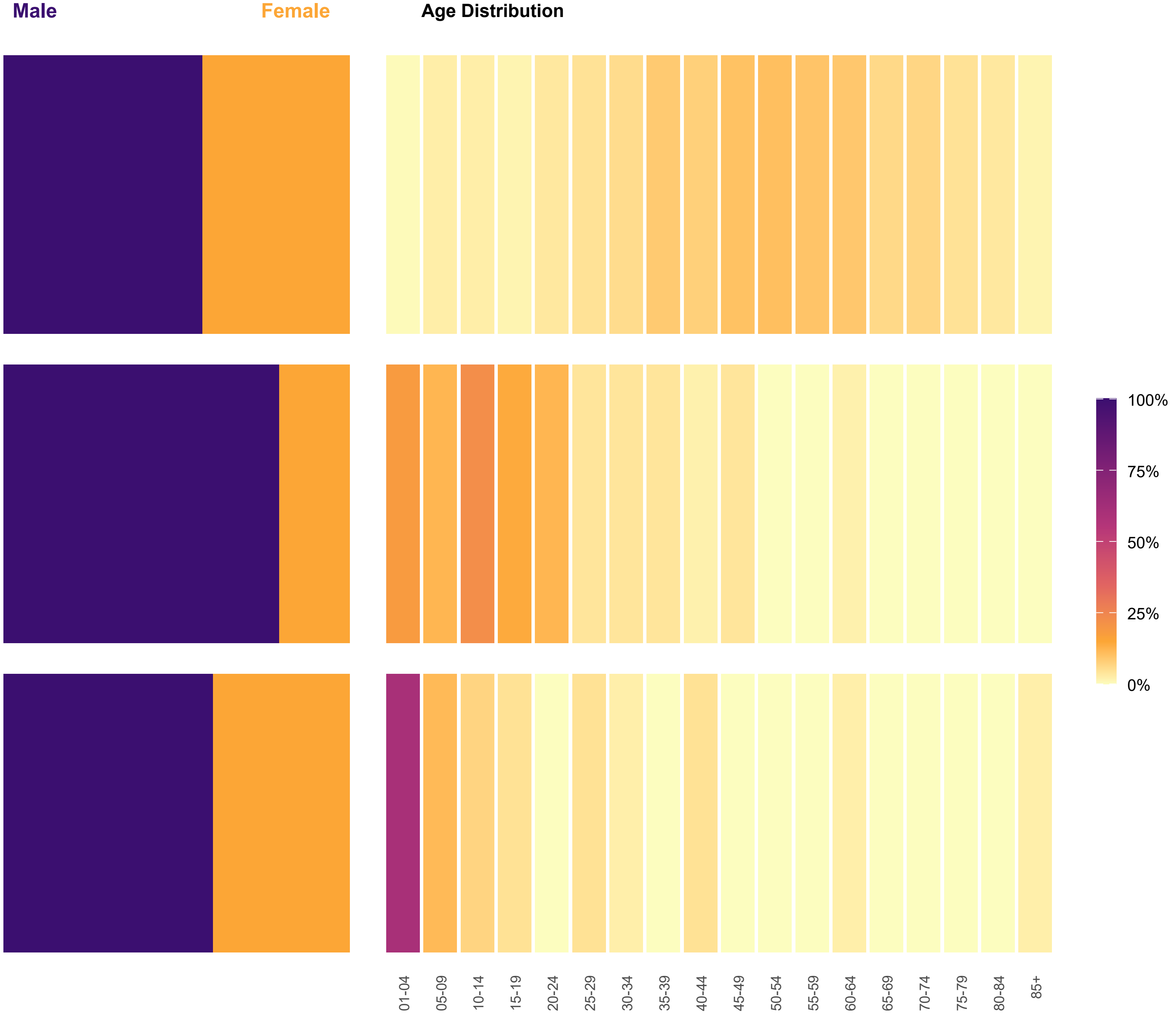

Primary Site: Brain | Phenotype: neuroectodermal

Top 25 Morphologies | cases: 244,293

Morphology	n	%
Glioblastoma	150,316	61.5%
Astrocytoma	17,807	7.3%
Astrocytoma anaplastic	17,731	7.3%
Glioma malignant	13,492	5.5%
Oligodendroglioma	9,039	3.7%
Oligoastrocytoma	5,920	2.4%
Oligodendroglioma anaplastic	4,956	2.0%
Medulloblastoma	4,293	1.8%
Ependymoma	3,714	1.5%
Gliosarcoma	3,276	1.3%
Fibrillary astrocytoma	2,503	1.0%
Ependymoma anaplastic	1,676	0.7%
Giant cell glioblastoma	1,638	0.7%
Gemistocytic astrocytoma	1,172	0.5%
CNS embryonal tumor	1,169	0.5%
Pleomorphic xanthoastrocytoma	1,074	0.4%
Desmoplastic nodular…	813	0.3%
Gliomatosis cerebri	599	0.2%
Atypical teratoid/rhabdoid tumor	534	0.2%
Glioblastoma IDH-mutant	415	0.2%
Ganglioglioma anaplastic	382	0.2%
Diffuse midline glioma H3…	311	0.1%
Large cell medulloblastoma	251	0.1%
Neuroblastoma	156	<0.1%
Astroblastoma	141	<0.1%

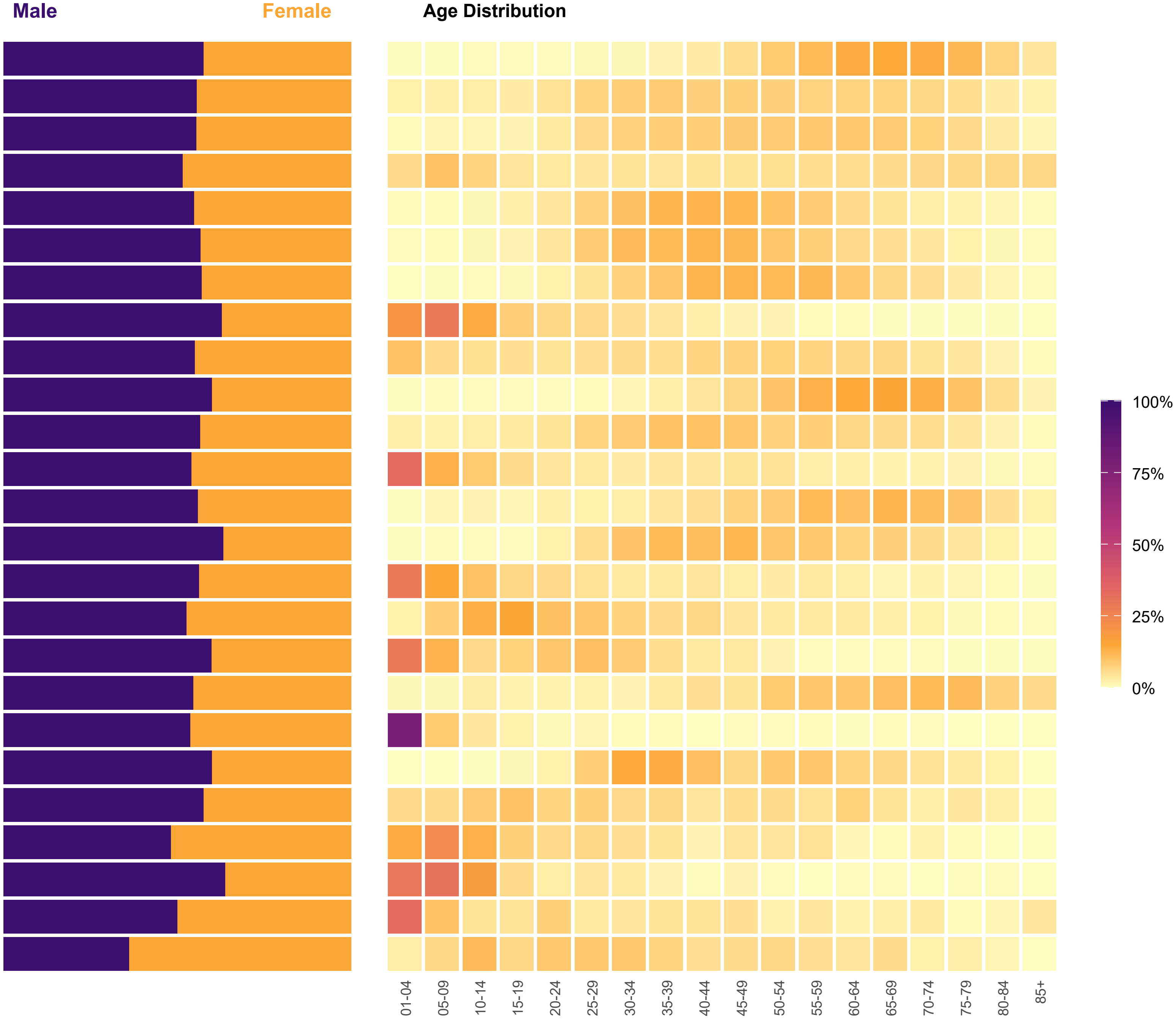

Primary Site: Breast | Phenotype: epithelial

Top 25 Morphologies | cases: 2,875,175

Morphology	n	%
Infiltrating duct carcinoma	2,159,280	75.1%
Lobular carcinoma	316,308	11.0%
Infiltrating duct and lobular…	142,813	5.0%
Infiltrating duct mixed with other…	62,254	2.2%
Mucinous adenocarcinoma	56,250	2.0%
Tubular adenocarcinoma	26,619	0.9%
Adenocarcinoma	23,135	0.8%
Intraductal papillary…	10,603	0.4%
Inflammatory carcinoma	10,583	0.4%
Infiltrating lobular mixed with…	9,731	0.3%
Invasive micropapillary carcinoma…	8,073	0.3%
Infiltrating ductular carcinoma	5,044	0.2%
Cribriform carcinoma	5,025	0.2%
Apocrine adenocarcinoma	4,726	0.2%
Paget disease and infiltrating…	4,703	0.2%
Paget disease and intraductal…	3,849	0.1%
Comedocarcinoma	3,687	0.1%
Encapsulated papillary carcinoma…	3,090	0.1%
Paget disease mammary	3,042	0.1%
Adenoid cystic carcinoma	2,200	<0.1%
Squamous cell carcinoma	1,631	<0.1%
Adenocarcinoma with apocrine…	1,630	<0.1%
Papillary adenocarcinoma	1,174	<0.1%
Neuroendocrine carcinoma	1,152	<0.1%
Adenocarcinoma with neuroendocrine…	1,146	<0.1%

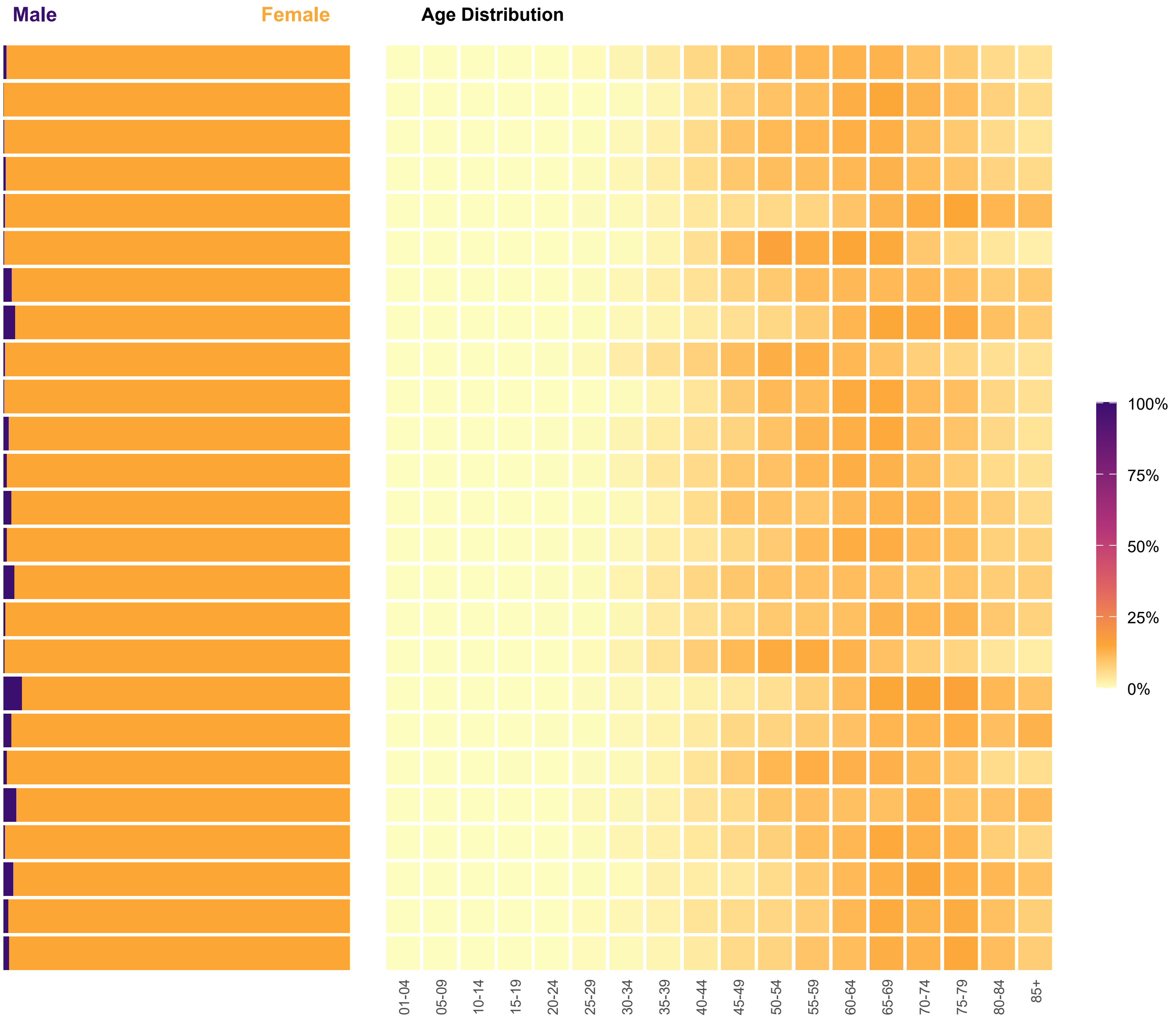

Primary Site: Breast | Phenotype: Grouped Phenotypes

Top 12 Morphologies | cases: 7,330

Morphology	n	%
Phyllodes tumor	5,116	69.8%
Carcinosarcoma	563	7.7%
Small cell carcinoma	485	6.6%
Myoepithelial carcinoma	348	4.7%
Large cell carcinoma	194	2.6%
Large cell neuroendocrine carcinoma	145	2.0%
Medullary thyroid carcinoma	96	1.3%
Neuroendocrine tumor	79	1.1%
Neuroendocrine tumor grade 2	74	1.0%
Adenomyoepithelioma with carcinoma	57	0.8%
Epithelial-myoepithelial carcinoma	48	0.7%
Malignant melanoma	24	0.3%

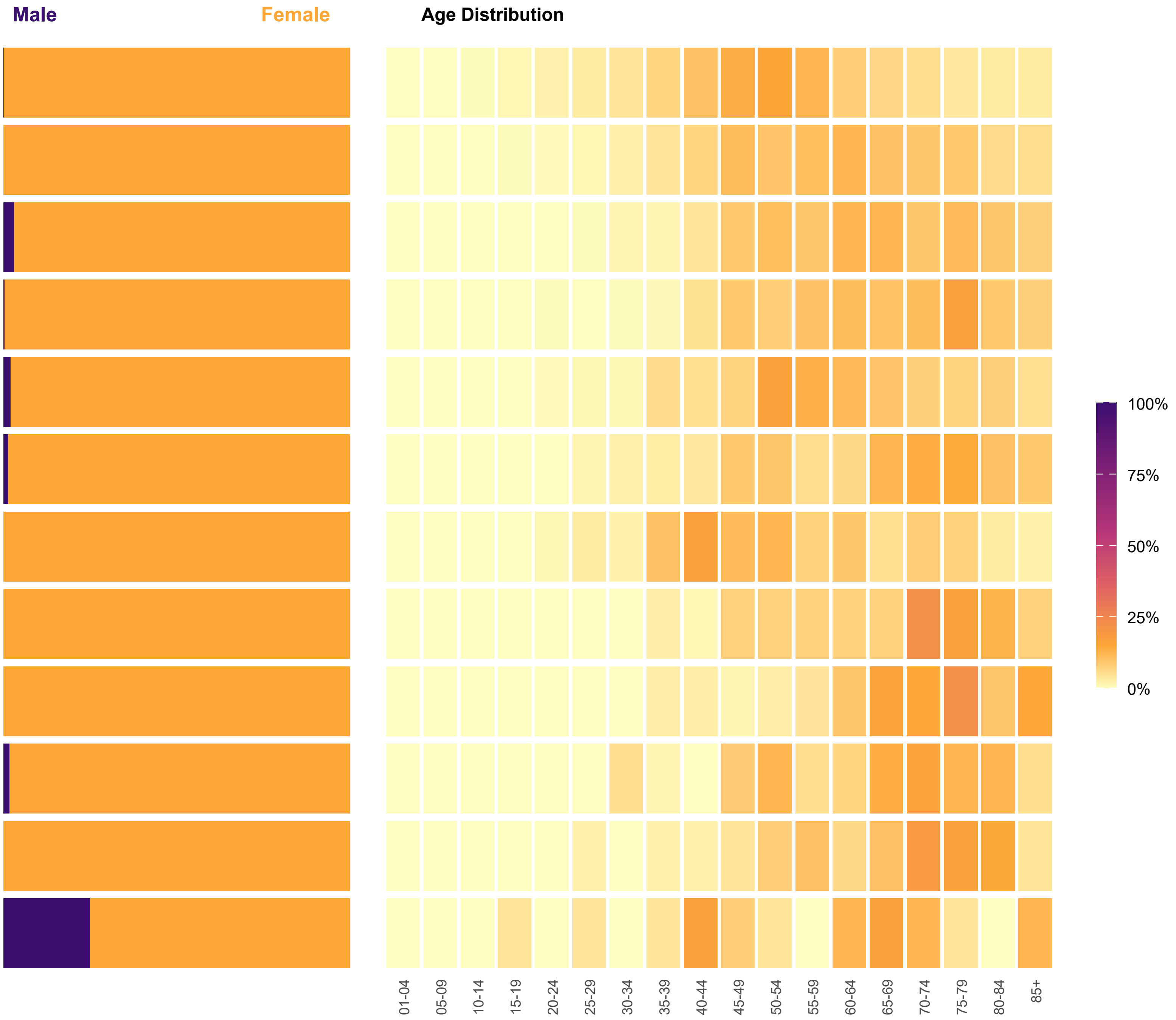

Primary Site: Buccal Mucosa | Phenotype: epithelial

Top 9 Morphologies | cases: 11,435

Morphology	n	%
Squamous cell carcinoma	9,902	86.6%
Mucoepidermoid carcinoma	521	4.6%
Adenoid cystic carcinoma	331	2.9%
Adenocarcinoma	170	1.5%
Polymorphous adenocarcinoma	115	1.0%
Papillary squamous cell carcinoma	105	0.9%
Acinar cell carcinoma	94	0.8%
Basaloid squamous cell carcinoma	31	0.3%
Secretory carcinoma	27	0.2%

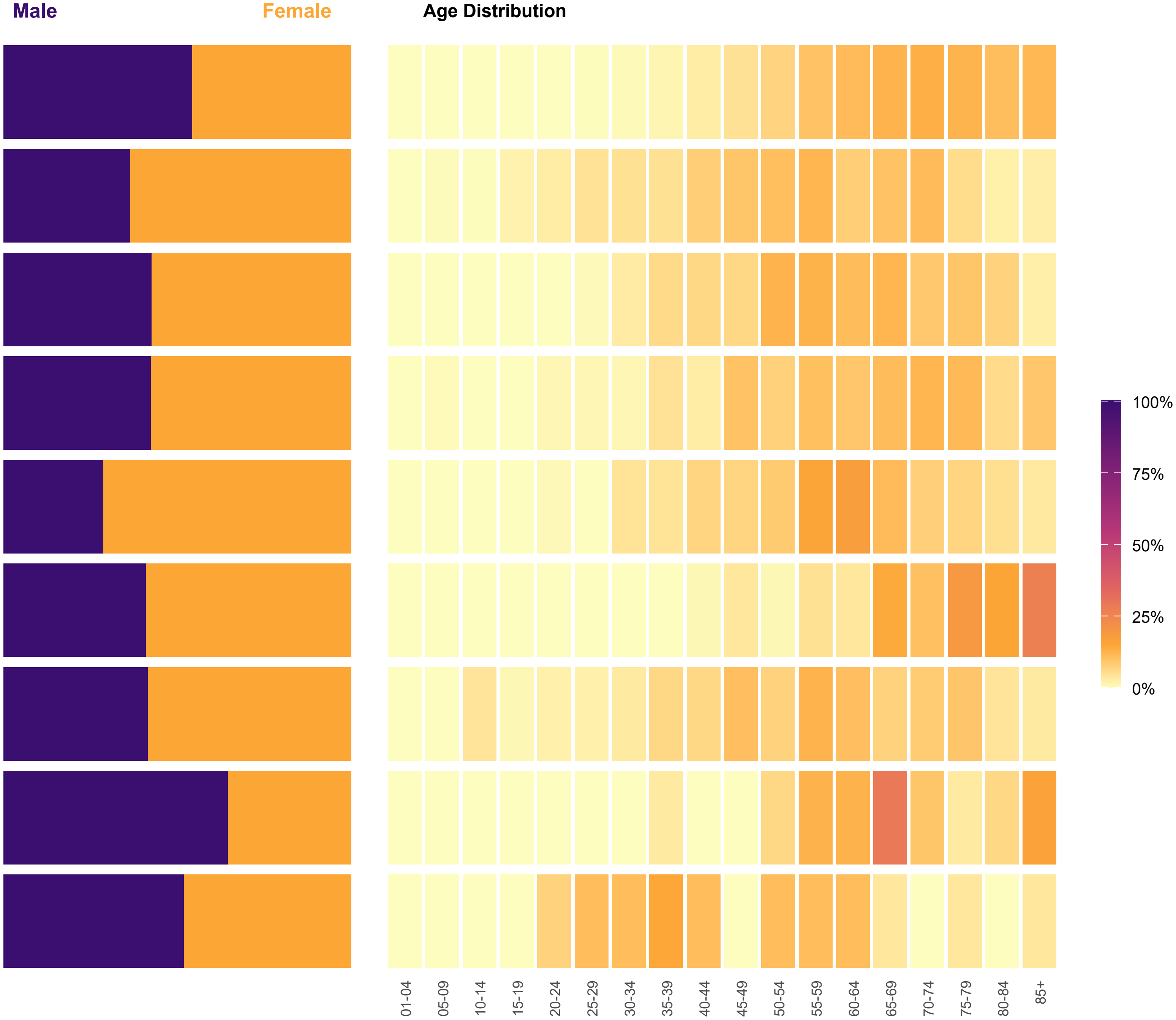

Primary Site: Buccal Mucosa | Phenotype: Grouped Phenotypes

Top 6 Morphologies | cases: 609

Morphology	n	%
Diffuse large B-cell lymphoma	135	22.2%
Marginal zone B-cell lymphoma	98	16.1%
Malignant melanoma	54	8.9%
Follicular lymphoma grade 1/2	39	6.4%
lymphoma non-Hodgkin	33	5.4%
Follicular lymphoma	21	3.4%

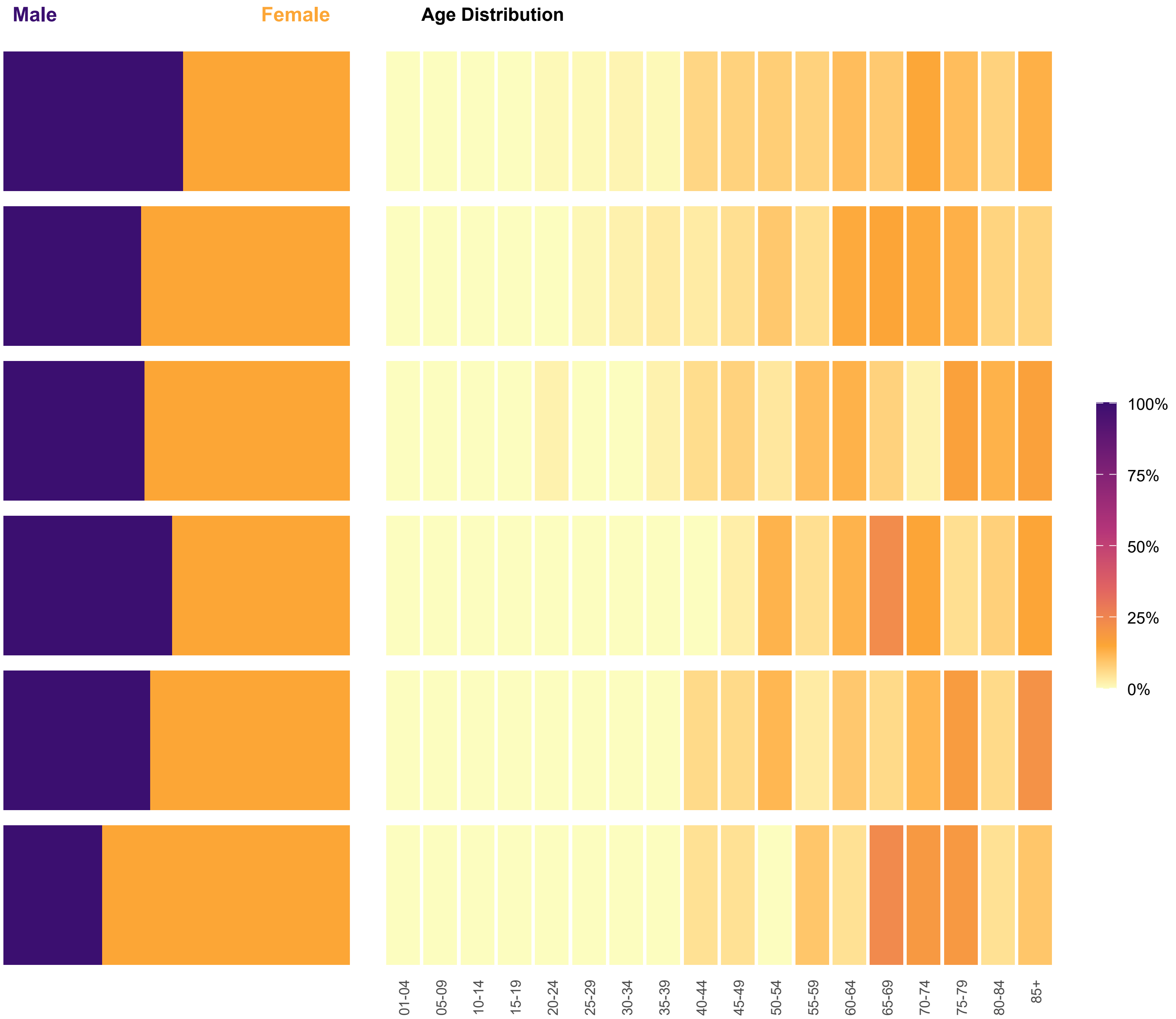

Primary Site: Cervix uteri | Phenotype: epithelial

Top 25 Morphologies | cases: 171,927

Morphology	n	%
Squamous cell carcinoma	129,427	75.3%
Adenocarcinoma	25,362	14.8%
Adenocarcinoma endocervical type	3,379	2.0%
Endometrioid adenocarcinoma	3,260	1.9%
Mucinous adenocarcinoma	1,763	1.0%
Clear cell adenocarcinoma	1,394	0.8%
Basaloid squamous cell carcinoma	894	0.5%
Mucinous carcinoma gastric type	824	0.5%
Papillary adenocarcinoma	766	0.4%
Papillary squamous cell carcinoma	721	0.4%
Mixed cell adenocarcinoma	721	0.4%
Neuroendocrine carcinoma	586	0.3%
Serous carcinoma	423	0.2%
Low grade serous carcinoma	297	0.2%
Adenocarcinoma in tubulovillous…	281	0.2%
Mucin-producing adenocarcinoma	223	0.1%
Basal cell carcinoma	199	0.1%
Villous adenocarcinoma	199	0.1%
Adenocarcinoma with mixed subtypes	148	<0.1%
Adenocarcinoma with squamous…	138	<0.1%
Adenocarcinoma with neuroendocrine…	126	<0.1%
Lymphoepithelial carcinoma	120	<0.1%
Signet ring cell carcinoma	92	<0.1%
Adenoid cystic carcinoma	83	<0.1%
Tubular adenocarcinoma	72	<0.1%

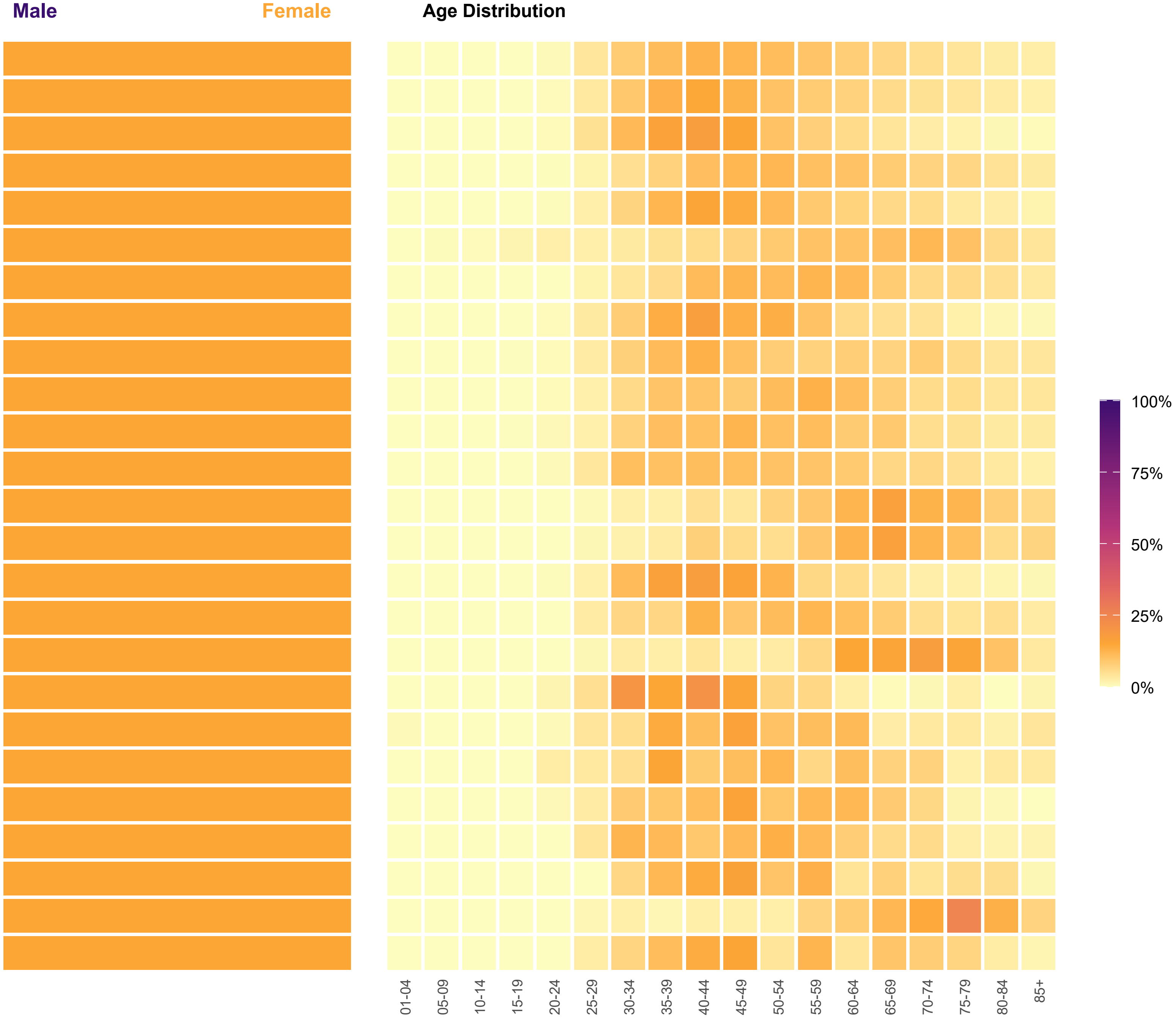

Primary Site: Cervix uteri | Phenotype: Grouped Phenotypes

Top 16 Morphologies | cases: 4,083

Morphology	n	%
Small cell carcinoma	1,278	31.3%
Carcinosarcoma	491	12.0%
Leiomyosarcoma	342	8.4%
Adenosarcoma	269	6.6%
Mullerian mixed tumor	248	6.1%
Large cell neuroendocrine carcinoma	213	5.2%
Diffuse large B-cell lymphoma	192	4.7%
Mesonephroma	139	3.4%
Embryonal rhabdomyosarcoma	122	3.0%
Malignant melanoma	99	2.4%
Sarcoma	95	2.3%
Mesodermal mixed tumor	63	1.5%
Combined small cell carcinoma	62	1.5%
Large cell carcinoma	57	1.4%
Rhabdomyosarcoma	34	0.8%
Choriocarcinoma	23	0.6%

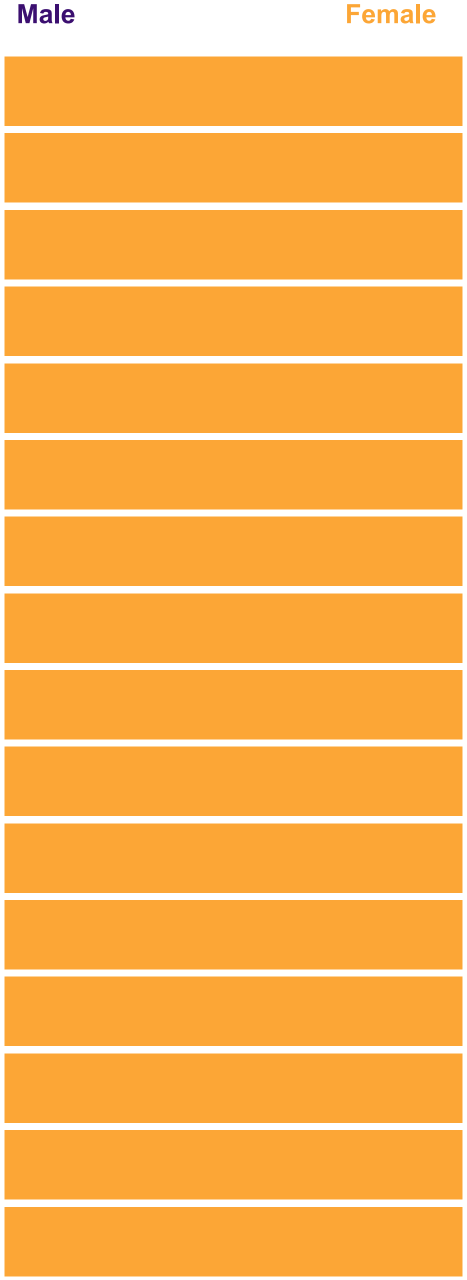

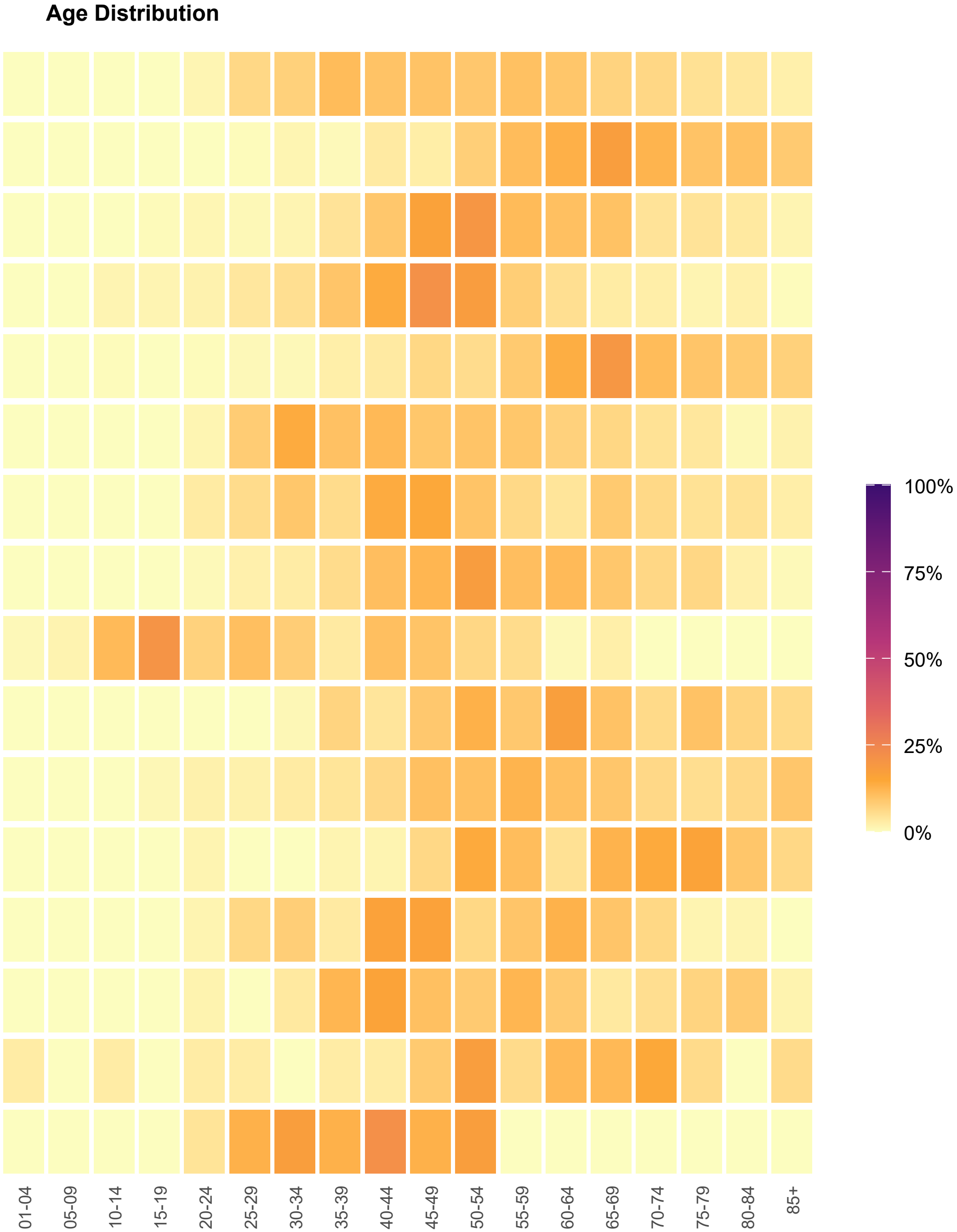

Primary Site: CNS Other | Phenotype: Grouped Phenotypes

Top 22 Morphologies | cases: 8,990

Morphology	n	%
Ependymoma	4,263	47.4%
Glioma malignant	1,868	20.8%
Astrocytoma	613	6.8%
Glioblastoma	359	4.0%
Neurilemomamalignant	307	3.4%
Malignant peripheral nerve sheath…	257	2.9%
Astrocytoma anaplastic	179	2.0%
Chordoma	170	1.9%
Ependymoma anaplastic	154	1.7%
CNS embryonal tumor	135	1.5%
Neuroblastoma	126	1.4%
Papillary ependymoma	72	0.8%
Fibrillary astrocytoma	66	0.7%
Germinoma	49	0.5%
Atypical teratoid/rhabdoid tumor	35	0.4%
Malignant melanoma	34	0.4%
Oligoastrocytoma	32	0.4%
Ganglioneuroblastoma	29	0.3%
Olfactory neuroblastoma	24	0.3%
Oligodendroglioma	22	0.2%
Diffuse midline glioma H3…	21	0.2%
Ganglioglioma anaplastic	20	0.2%

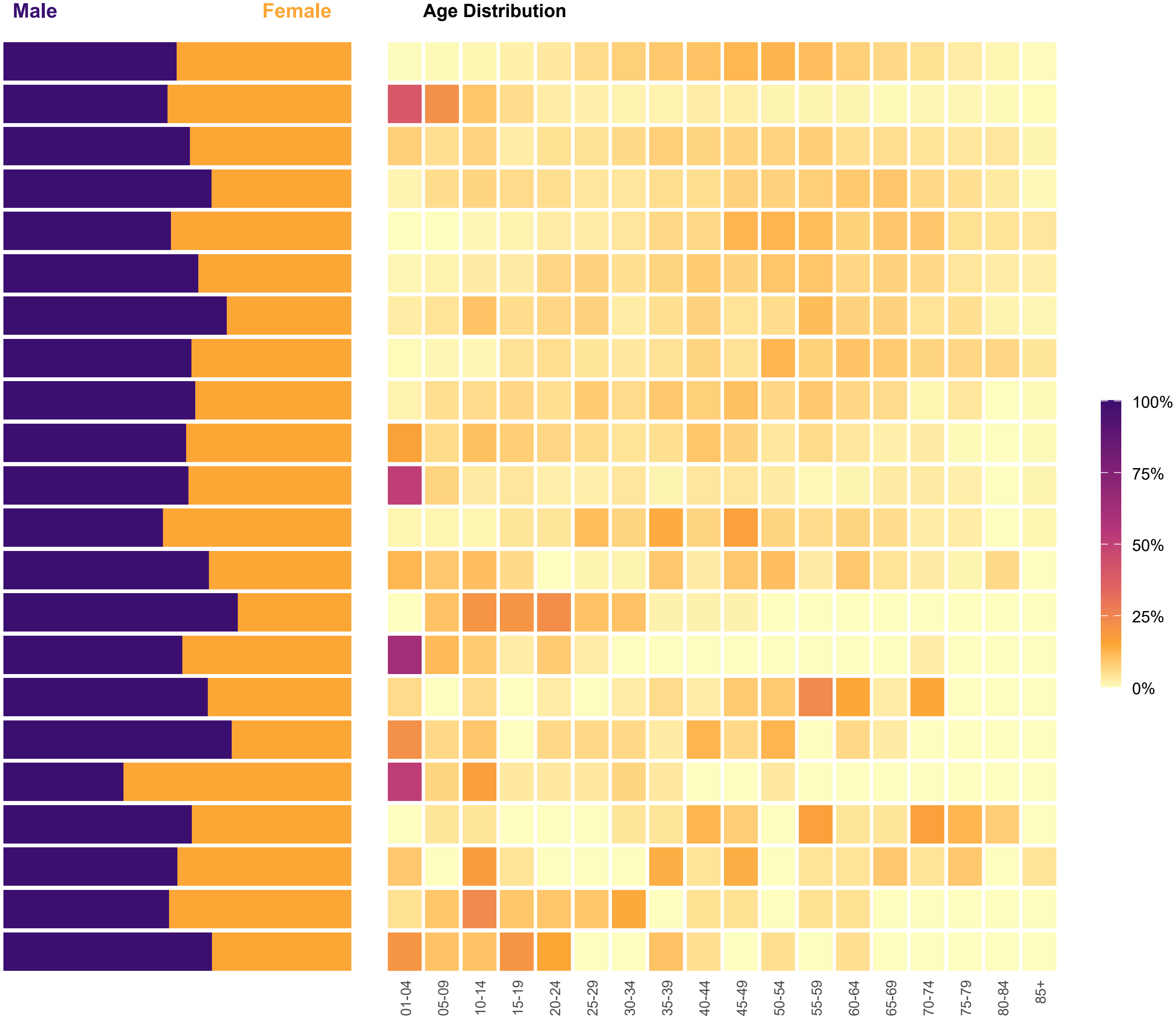

Primary Site: Colon and Rectum (excluding Appendix) | Phenotype: epithelial

Top 25 Morphologies | cases: 1,966,720

Morphology	n	%
Adenocarcinoma	1,497,999	76.2%
Mucinous adenocarcinoma	119,041	6.1%
Adenocarcinoma in tubulovillous…	116,977	5.9%
Adenocarcinoma in adenomatous polyp	82,423	4.2%
Mucin-producing adenocarcinoma	35,163	1.8%
Tubular adenocarcinoma	32,741	1.7%
Adenocarcinoma in villous adenoma	25,406	1.3%
Signet ring cell carcinoma	16,297	0.8%
Neuroendocrine carcinoma	11,088	0.6%
Squamous cell carcinoma	7,547	0.4%
Adenocarcinoma intestinal type	6,947	0.4%
Papillary adenocarcinoma	3,751	0.2%
Adenocarcinoma with mixed subtypes	3,243	0.2%
Cribriform carcinoma	2,677	0.1%
Adenocarcinoma with neuroendocrine…	1,128	<0.1%
Villous adenocarcinoma	928	<0.1%
Adenocarcinoma in adenomatous…	511	<0.1%
Basaloid squamous cell carcinoma	487	<0.1%
Serrated adenocarcinoma	430	<0.1%
Adenocarcinoma in multiple…	329	<0.1%
Cloacogenic carcinoma	199	<0.1%
Mixed cell adenocarcinoma	173	<0.1%
Adenoid cystic carcinoma	130	<0.1%
Scirrhous adenocarcinoma	105	<0.1%
Basaloid carcinoma	102	<0.1%

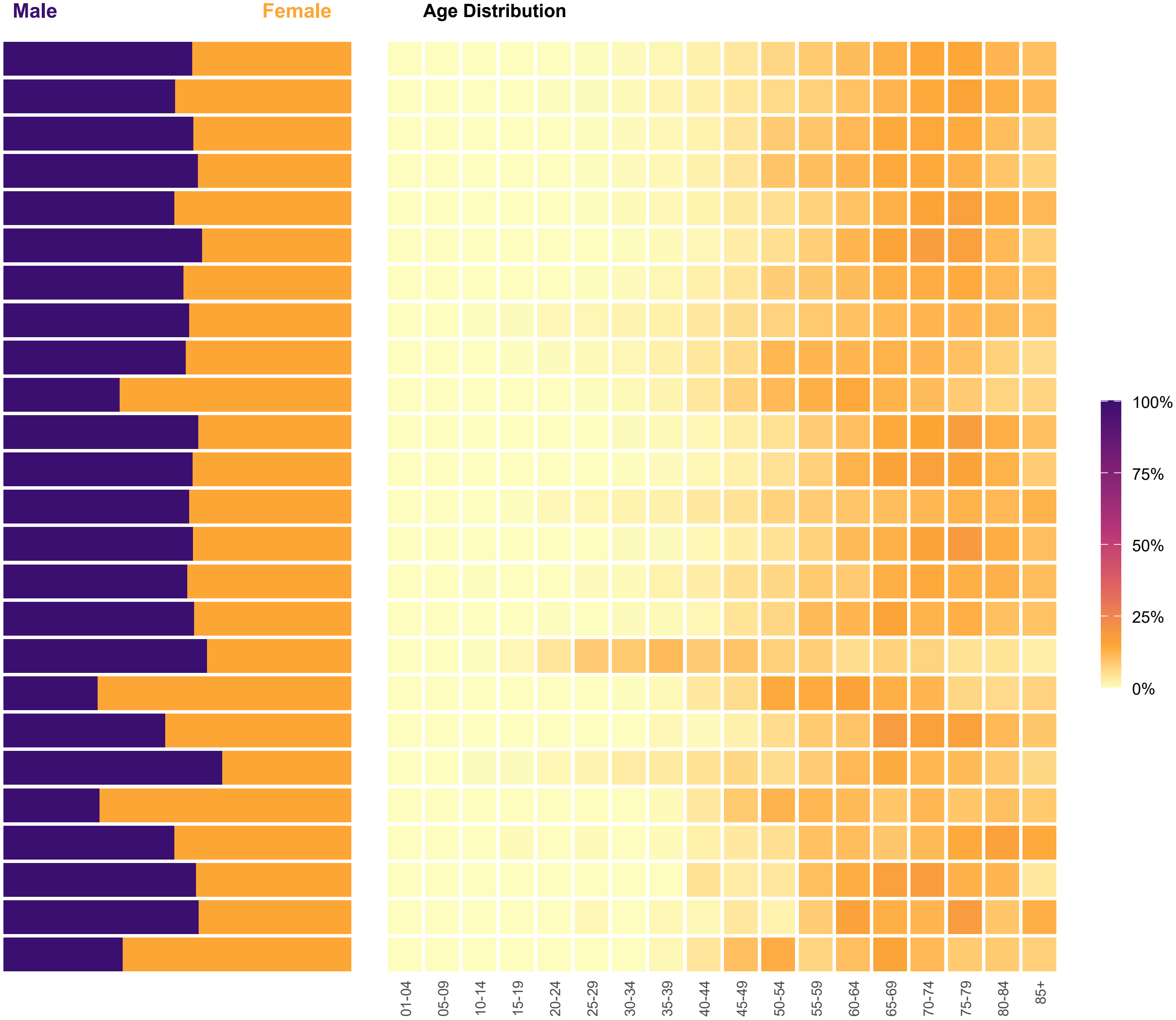

Primary Site: Colon and Rectum (excluding Appendix) | Phenotype: Grouped Phenotypes

Top 16 Morphologies | cases: 43,648

Morphology	n	%
Neuroendocrine tumor	36,650	84.0%
Small cell carcinoma	1,657	3.8%
Mixed adenoneuroendocrine carcinoma	1,252	2.9%
Large cell neuroendocrine carcinoma	1,134	2.6%
Neuroendocrine tumor grade 2	1,077	2.5%
Malignant melanoma	822	1.9%
Adenocarcinoid tumor	263	0.6%
Goblet cell carcinoid	241	0.6%
Large cell carcinoma	117	0.3%
Enterochromaffin cell carcinoid	79	0.2%
Carcinosarcoma	61	0.1%
Amelanotic melanoma	55	0.1%
Combined small cell carcinoma	39	<0.1%
Nodular melanoma	36	<0.1%
Large cell carcinoma with rhabdoid…	27	<0.1%
Medullary thyroid carcinoma	22	<0.1%

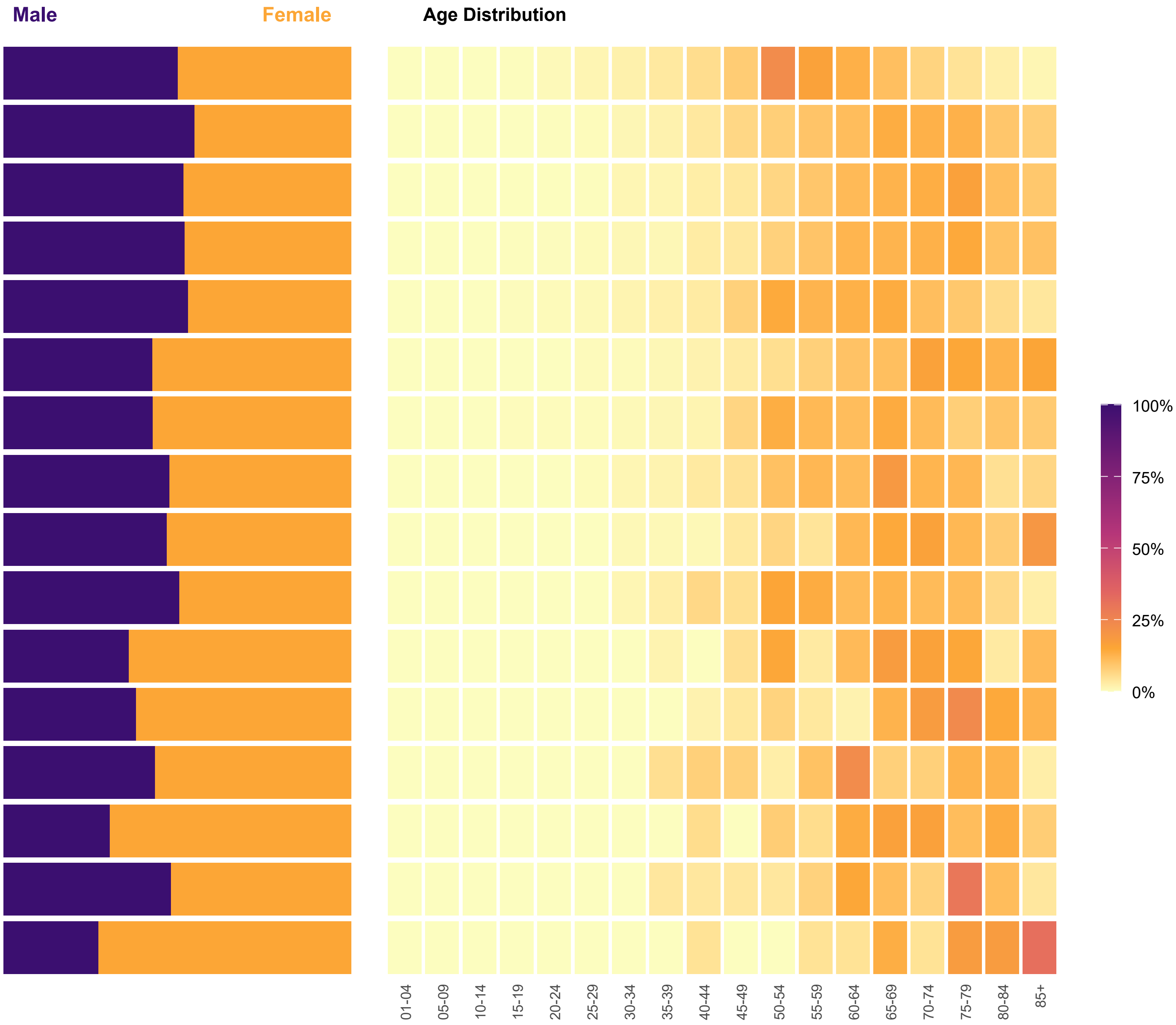

Primary Site: Corpus uteri | Phenotype: epithelial

Top 25 Morphologies | cases: 493,112

Morphology	n	%
Endometrioid adenocarcinoma	355,130	72.0%
Adenocarcinoma	56,126	11.4%
Serous carcinoma	19,725	4.0%
Mixed cell adenocarcinoma	19,617	4.0%
Low grade serous carcinoma	10,126	2.1%
Clear cell adenocarcinoma	7,540	1.5%
Adenocarcinoma with squamous…	6,907	1.4%
Mucinous adenocarcinoma	3,453	0.7%
Papillary adenocarcinoma	2,874	0.6%
Squamous cell carcinoma	2,169	0.4%
Cystadenocarcinoma	1,636	0.3%
High grade serous carcinoma	1,500	0.3%
Adenocarcinoma with mixed subtypes	1,283	0.3%
Adenocarcinoma in adenomatous polyp	1,160	0.2%
Endometrioid adenocarcinoma…	875	0.2%
Adenocarcinoma in tubulovillous…	858	0.2%
Villous adenocarcinoma	342	<0.1%
Neuroendocrine carcinoma	325	<0.1%
Tubular adenocarcinoma	313	<0.1%
Mucin-producing adenocarcinoma	306	<0.1%
Endometrioid adenocarcinoma…	190	<0.1%
Adenocarcinoma with neuroendocrine…	114	<0.1%
Mucinous carcinoma gastric type	109	<0.1%
Endometrioid adenofibroma malignant	99	<0.1%
Adenocarcinoma endocervical type	38	<0.1%

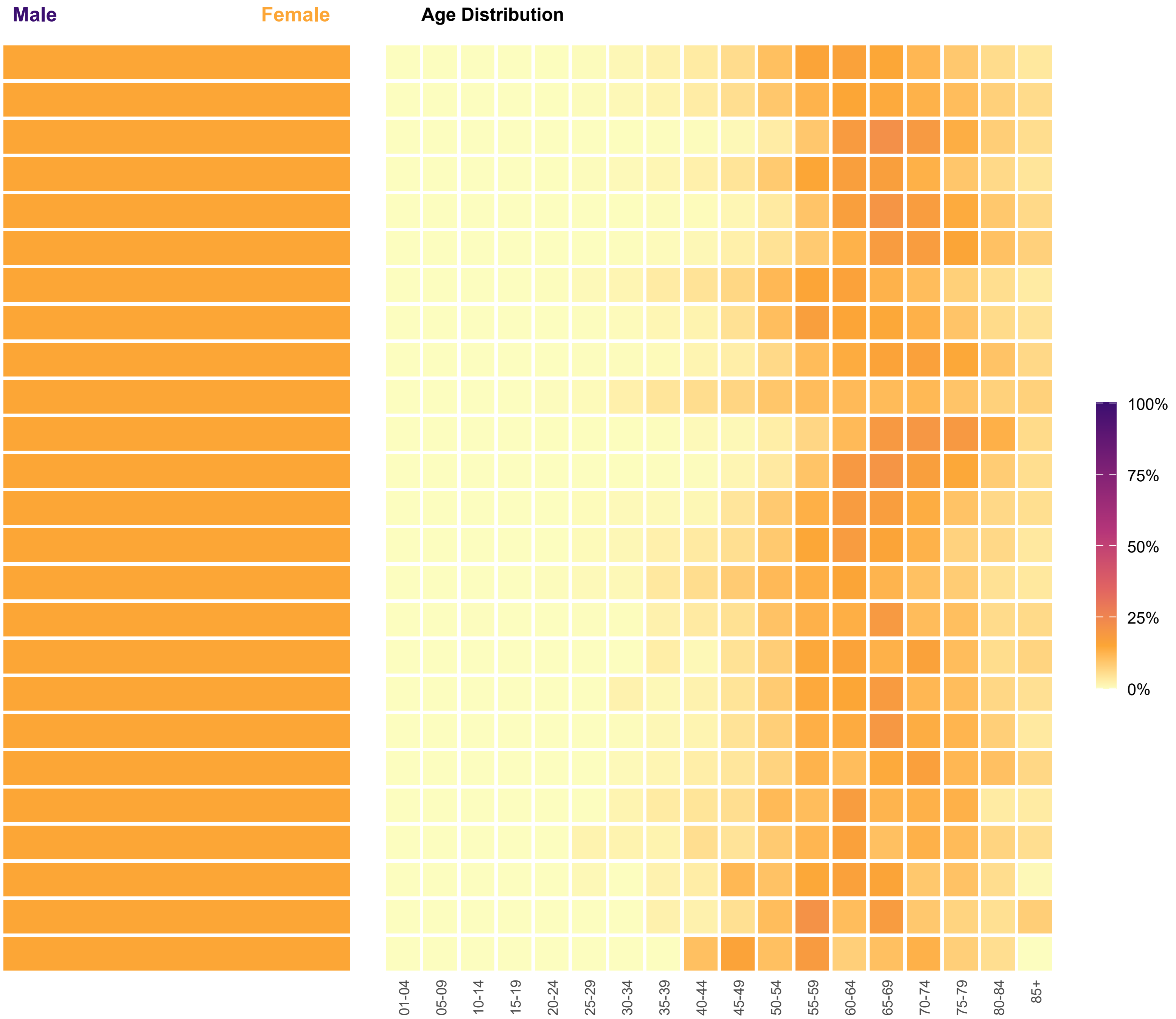

Primary Site: Corpus uteri | Phenotype: Grouped Phenotypes

Top 16 Morphologies | cases: 27,273

Morphology	n	%
Carcinosarcoma	15,985	58.6%
Mullerian mixed tumor	8,822	32.3%
Mesodermal mixed tumor	924	3.4%
Small cell carcinoma	374	1.4%
Choriocarcinoma	367	1.3%
Diffuse large B-cell lymphoma	130	0.5%
Large cell neuroendocrine carcinoma	127	0.5%
Mesonephroma	75	0.3%
Trophoblastic tumor epithelioid	70	0.3%
Mesenchymoma	42	0.2%
Large cell carcinoma	41	0.2%
CNS embryonal tumor	37	0.1%
Marginal zone B-cell lymphoma	33	0.1%
Rhabdoid tumor	27	<0.1%
Combined small cell carcinoma	22	<0.1%
Yolk sac tumor	21	<0.1%

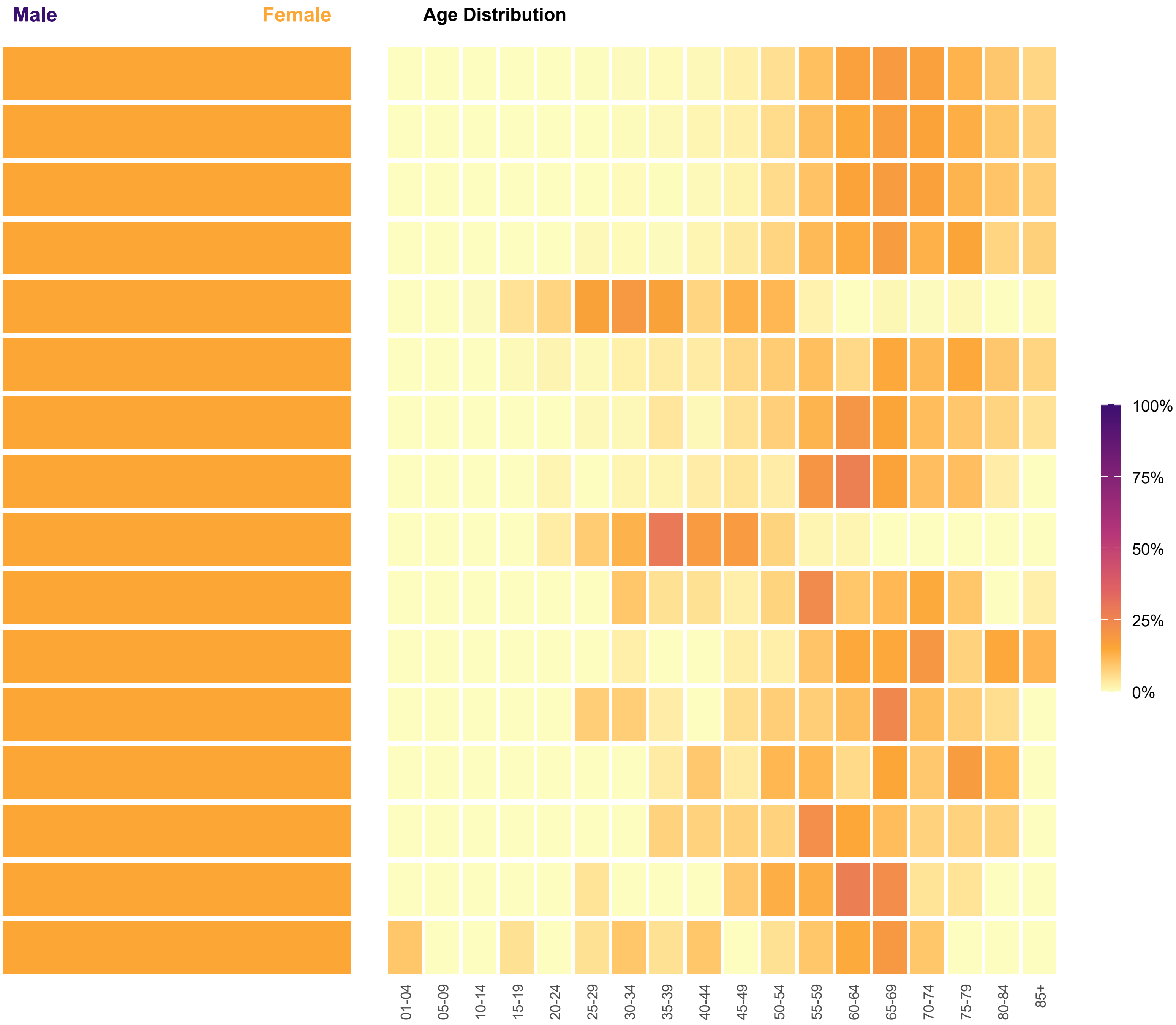

Primary Site: Digestive Other | Phenotype: epithelial

Top 16 Morphologies | cases: 18,238

Morphology	n	%
Adenocarcinoma	13,808	75.7%
Neuroendocrine carcinoma	1,059	5.8%
Mucinous adenocarcinoma	1,000	5.5%
Signet ring cell carcinoma	917	5.0%
Mucin-producing adenocarcinoma	427	2.3%
Tubular adenocarcinoma	142	0.8%
Adenocarcinoma intestinal type	129	0.7%
Infiltrating duct carcinoma	126	0.7%
Cholangiocarcinoma	90	0.5%
Squamous cell carcinoma	87	0.5%
Adenocarcinoma with mixed subtypes	84	0.5%
Adenocarcinoma in tubulovillous…	75	0.4%
Pancreatobiliary type carcinoma	70	0.4%
Adenocarcinoma with neuroendocrine…	50	0.3%
Papillary adenocarcinoma	47	0.3%
Adenocarcinoma in adenomatous polyp	22	0.1%

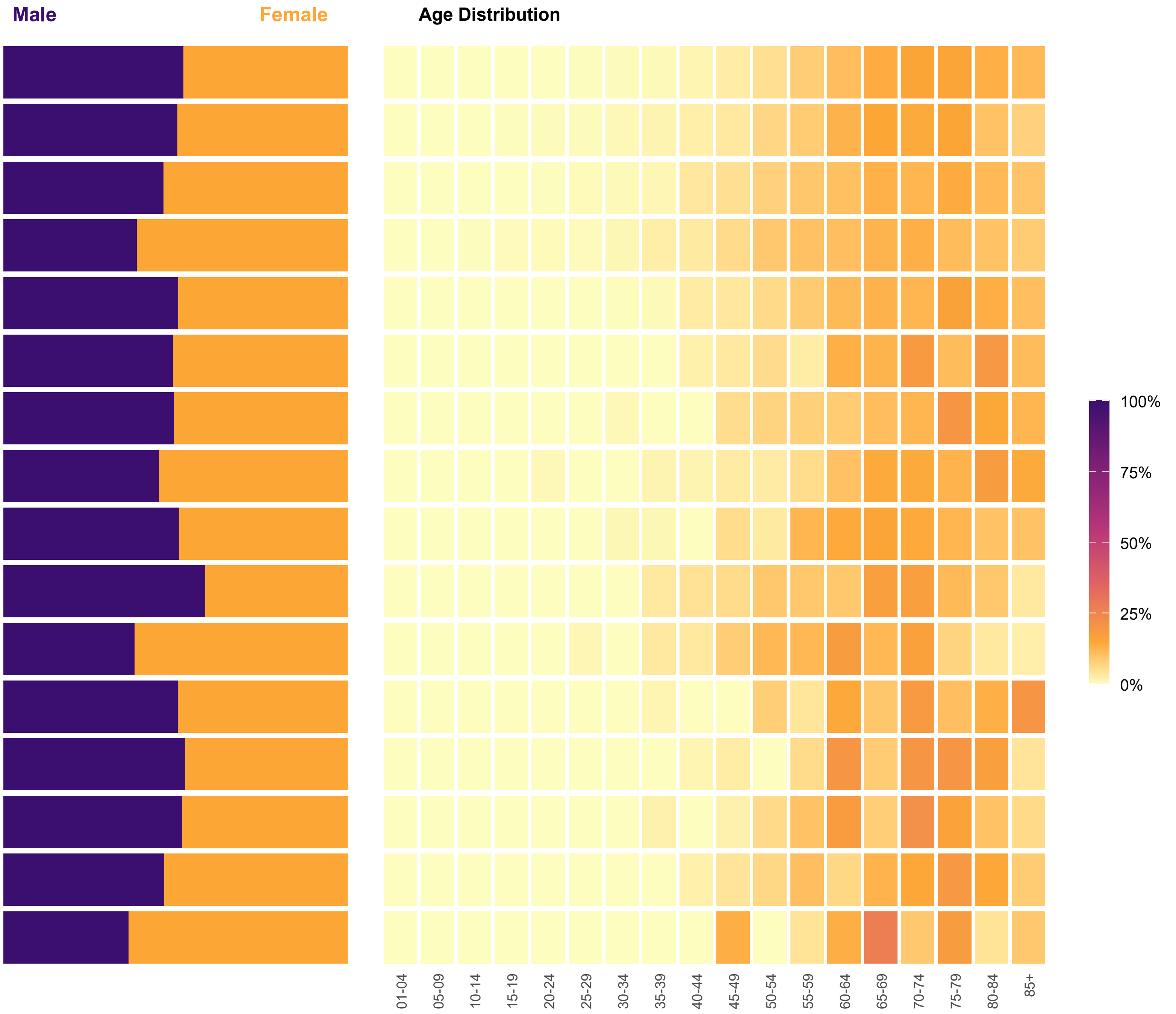

Primary Site: Digestive Other | Phenotype: Grouped Phenotypes

Top 17 Morphologies | cases: 4,252

Morphology	n	%
Gastrointestinal stromal tumor	1,882	44.3%
Neuroendocrine tumor	976	23.0%
Diffuse large B-cell lymphoma	246	5.8%
Neuroendocrine tumor grade 2	168	4.0%
Mantle cell lymphoma	109	2.6%
Marginal zone B-cell lymphoma	104	2.4%
Small cell carcinoma	100	2.4%
Sarcoma	92	2.2%
Kaposi sarcoma	76	1.8%
Large cell neuroendocrine carcinoma	72	1.7%
Burkitt lymphoma	37	0.9%
Gastrinoma	33	0.8%
lymphoma non-Hodgkin	28	0.7%
Intestinal T-cell lymphoma	26	0.6%
Leiomyosarcoma	25	0.6%
lymphoma	23	0.5%
Mature T-cell lymphoma	20	0.5%

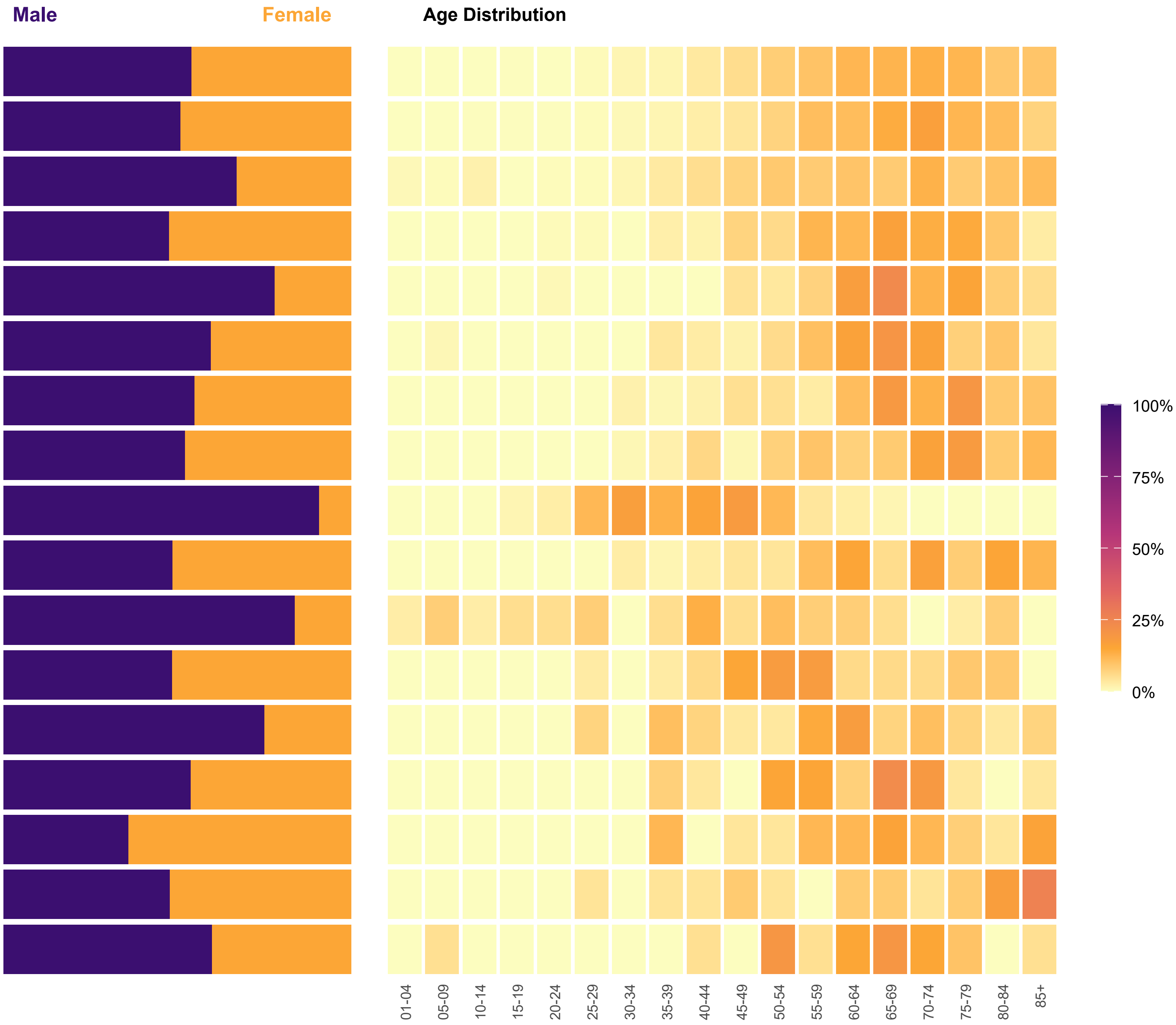

Primary Site: Endocrine Other | Phenotype: epithelial

Top 3 Morphologies | cases: 206

Morphology	n	%
Neuroendocrine carcinoma	140	68.0%
Adenocarcinoma	27	13.1%
Adenocarcinoma with neuroendocrine…	24	11.7%

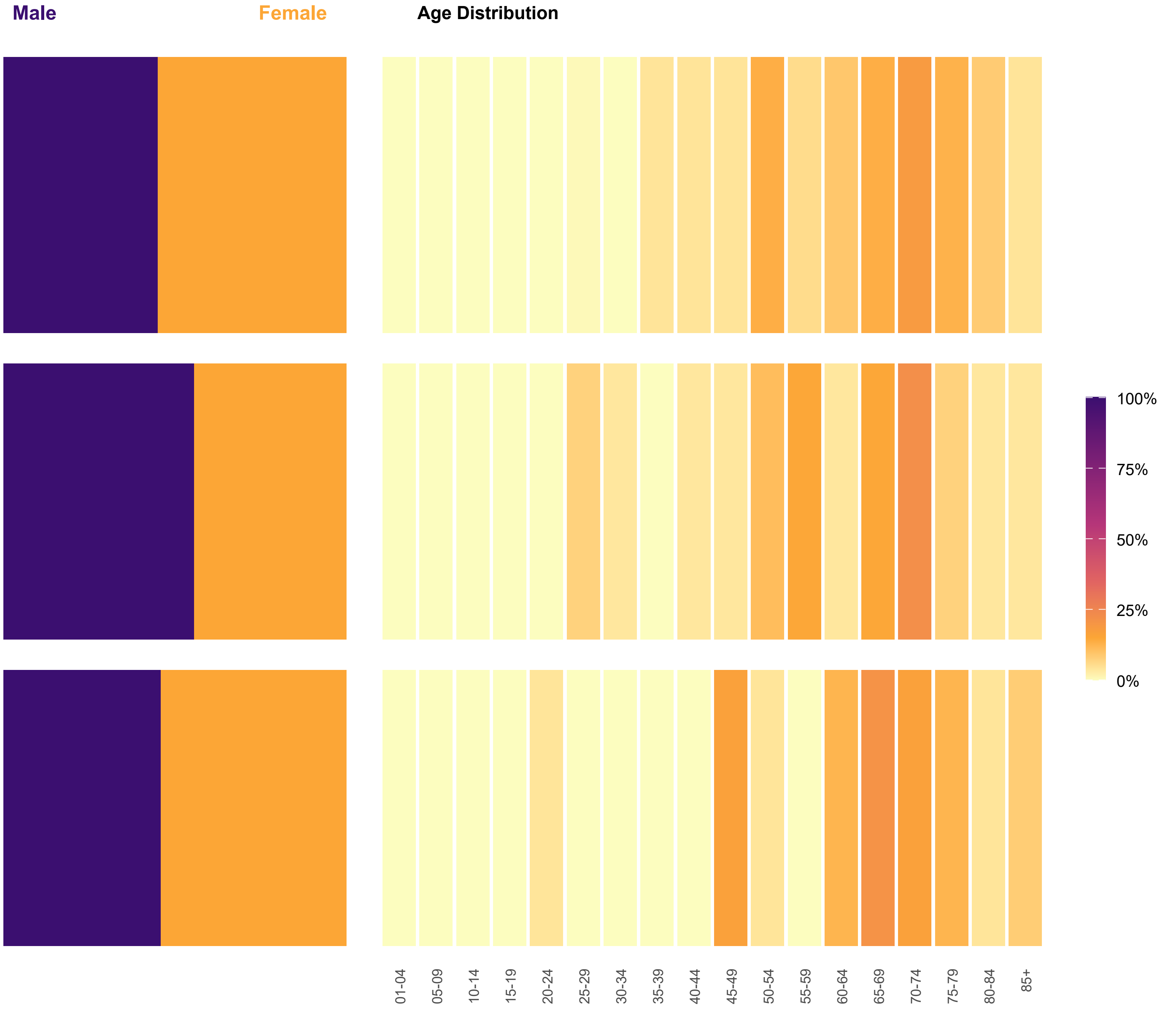

Primary Site: Endocrine Other | Phenotype: Grouped Phenotypes

Top 5 Morphologies | cases: 677

Morphology	n	%
Paraganglioma	425	62.8%
Extra-adrenal paraganglioma	55	8.1%
Glomus tumormalignant	46	6.8%
Carotid body paraganglioma	27	4.0%
Neuroendocrine tumor	20	3.0%

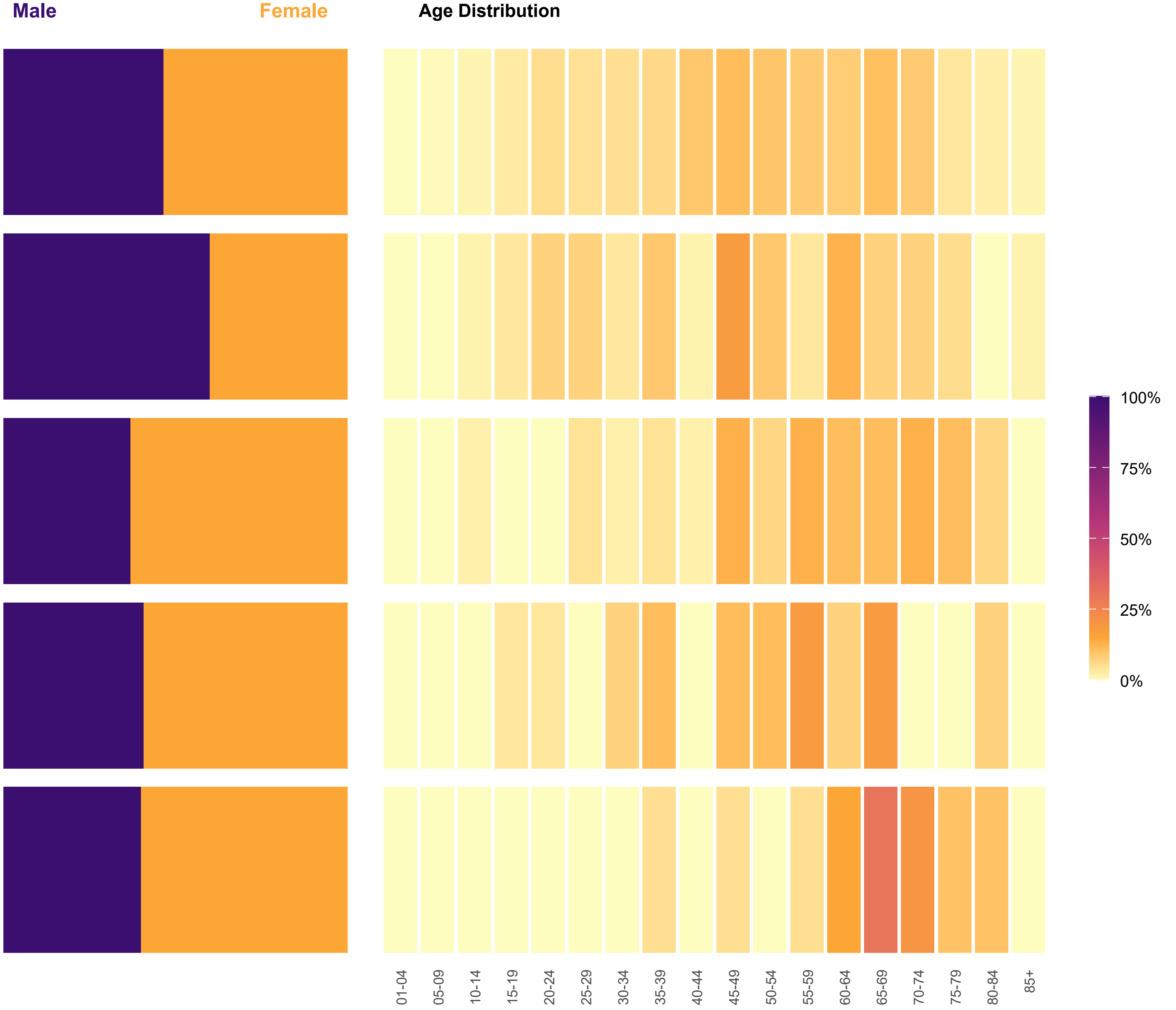

Primary Site: Epididymis | Phenotype: epithelial

Top 7 Morphologies | cases: 2,447

Morphology	n	%
Squamous cell carcinoma	963	39.4%
Basal cell carcinoma	661	27.0%
Paget disease extramammary	547	22.4%
Adenocarcinoma	85	3.5%
Basosquamous carcinoma	33	1.3%
Basaloid squamous cell carcinoma	24	1.0%
Transitional cell carcinoma	24	1.0%

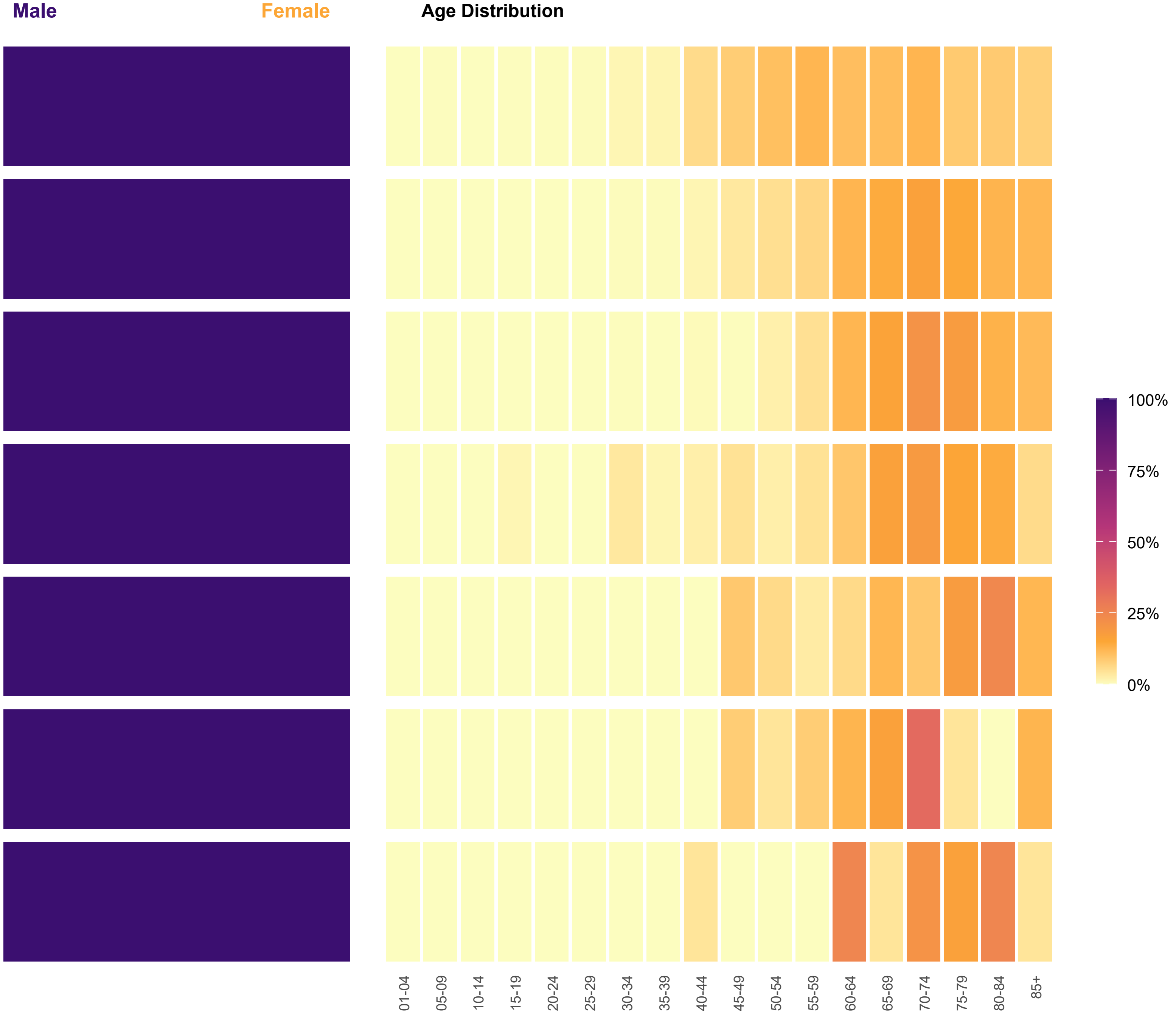

Primary Site: Epididymis | Phenotype: Grouped Phenotypes

Top 19 Morphologies | cases: 2,420

Morphology	n	%
Leiomyosarcoma	461	19.0%
Liposarcoma well differentiated	438	18.1%
Dedifferentiated liposarcoma	340	14.0%
Liposarcoma	311	12.9%
Embryonal rhabdomyosarcoma	83	3.4%
Mesotheliomamalignant	79	3.3%
Myxoid liposarcoma	54	2.2%
Epithelioid mesothelioma	50	2.1%
Malignant fibrous histiocytoma	47	1.9%
Malignant melanoma	47	1.9%
Sarcoma	37	1.5%
Rhabdomyosarcoma	24	1.0%
Spindle cell sarcoma	23	1.0%
Diffuse large B-cell lymphoma	23	1.0%
Pleomorphic liposarcoma	23	1.0%
Nodular melanoma	23	1.0%
Seminoma	22	0.9%
Low cumulative sun damage melanoma	22	0.9%
Giant cell sarcoma	21	0.9%

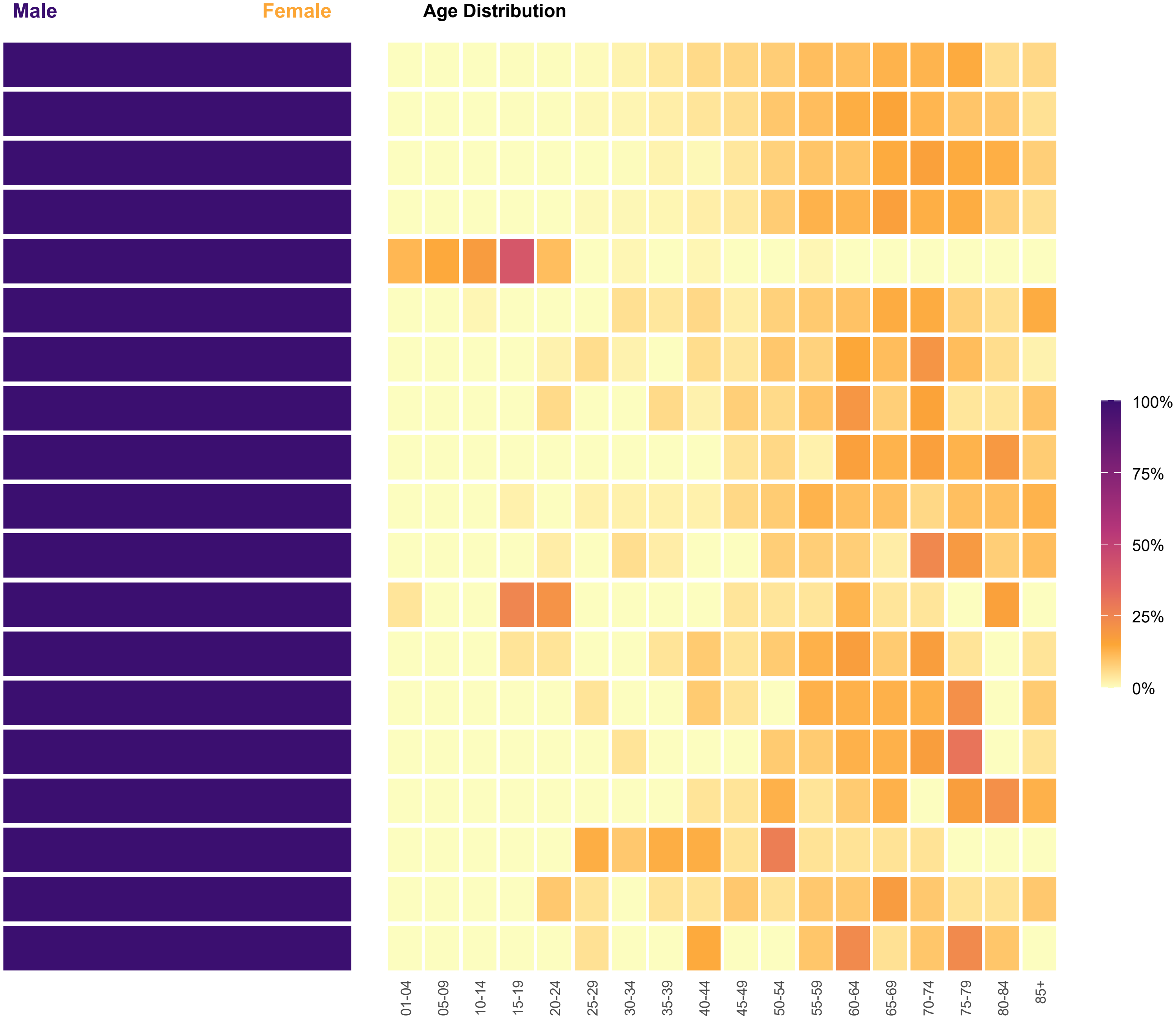

Primary Site: Esophagus | Phenotype: epithelial

Top 23 Morphologies | cases: 201,212

Morphology	n	%
Adenocarcinoma	94,956	47.2%
Squamous cell carcinoma	91,843	45.6%
Signet ring cell carcinoma	3,957	2.0%
Adenocarcinoma intestinal type	2,088	1.0%
Tubular adenocarcinoma	1,825	0.9%
Mucinous adenocarcinoma	1,638	0.8%
Mucin-producing adenocarcinoma	1,034	0.5%
Basaloid squamous cell carcinoma	897	0.4%
Neuroendocrine carcinoma	787	0.4%
Adenocarcinoma with mixed subtypes	656	0.3%
Papillary adenocarcinoma	442	0.2%
Adenocarcinoma in tubulovillous…	388	0.2%
Adenocarcinoma with neuroendocrine…	216	0.1%
Adenocarcinoma in adenomatous polyp	96	<0.1%
Adenoid cystic carcinoma	51	<0.1%
Papillary squamous cell carcinoma	38	<0.1%
Mixed cell adenocarcinoma	34	<0.1%
Cribriform carcinoma	32	<0.1%
Mucoepidermoid carcinoma	32	<0.1%
Adenocarcinoma with squamous…	27	<0.1%
Lymphoepithelial carcinoma	23	<0.1%
Clear cell adenocarcinoma	23	<0.1%
Basaloid carcinoma	20	<0.1%

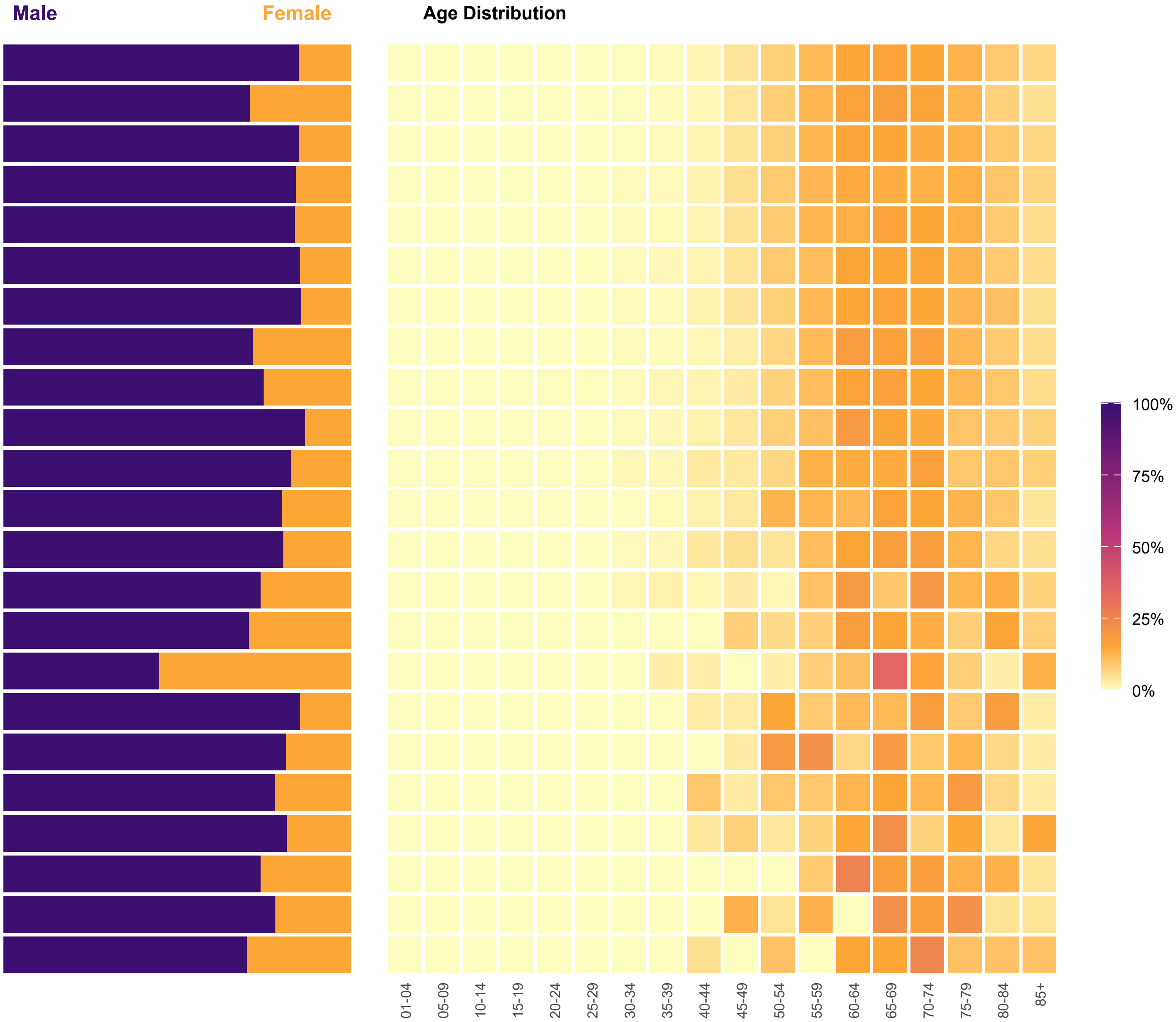

Primary Site: Esophagus | Phenotype: Grouped Phenotypes

Top 14 Morphologies | cases: 2,665

Morphology	n	%
Small cell carcinoma	1,066	40.0%
Gastrointestinal stromal tumor	251	9.4%
Malignant melanoma	172	6.5%
Large cell neuroendocrine carcinoma	158	5.9%
Diffuse large B-cell lymphoma	139	5.2%
Large cell carcinoma	116	4.4%
Mixed adenoneuroendocrine carcinoma	88	3.3%
Neuroendocrine tumor	77	2.9%
Leiomyosarcoma	74	2.8%
Sarcoma	59	2.2%
Carcinosarcoma	57	2.1%
Combined small cell carcinoma	57	2.1%
Marginal zone B-cell lymphoma	46	1.7%
lymphoma non-Hodgkin	26	1.0%

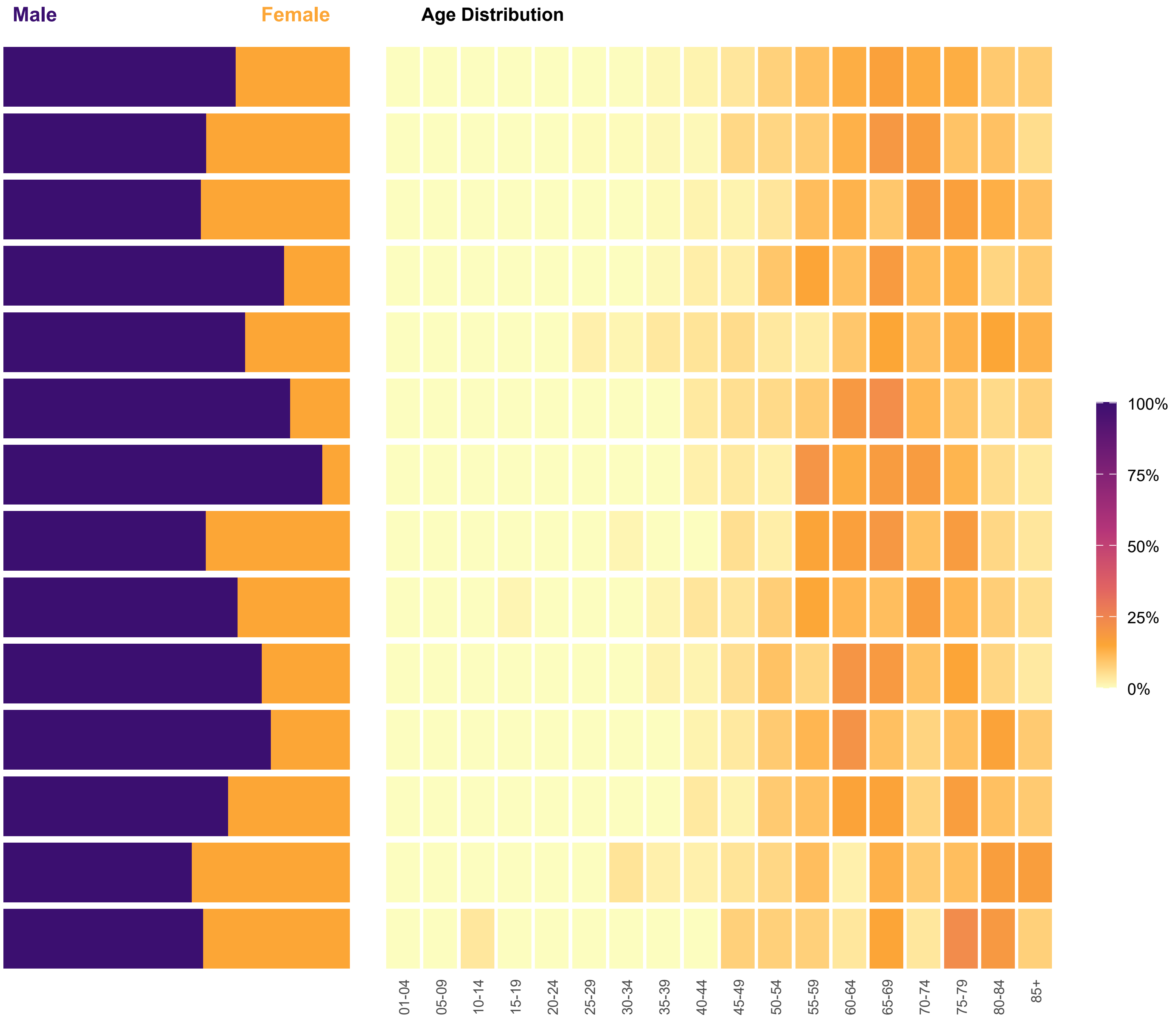

Primary Site: Extrahepatic Bile Ducts | Phenotype: epithelial

Top 20 Morphologies | cases: 50,455

Morphology	n	%
Adenocarcinoma	21,517	42.6%
Cholangiocarcinoma	16,863	33.4%
Klatskin tumor	8,842	17.5%
Infiltrating duct carcinoma	792	1.6%
Mucinous adenocarcinoma	481	1.0%
Papillary adenocarcinoma	338	0.7%
Mucin-producing adenocarcinoma	336	0.7%
Tubular adenocarcinoma	245	0.5%
Signet ring cell carcinoma	214	0.4%
Neuroendocrine carcinoma	134	0.3%
Pancreatobiliary type carcinoma	116	0.2%
Adenocarcinoma in tubulovillous…	94	0.2%
Adenocarcinoma intestinal type	66	0.1%
Squamous cell carcinoma	60	0.1%
Intraductal papillary…	55	0.1%
Combined hepatocellular carcinoma…	43	<0.1%
Bile duct cystadenocarcinoma	39	<0.1%
Adenocarcinoma with mixed subtypes	39	<0.1%
Hepatocellular carcinoma	29	<0.1%
Clear cell adenocarcinoma	25	<0.1%

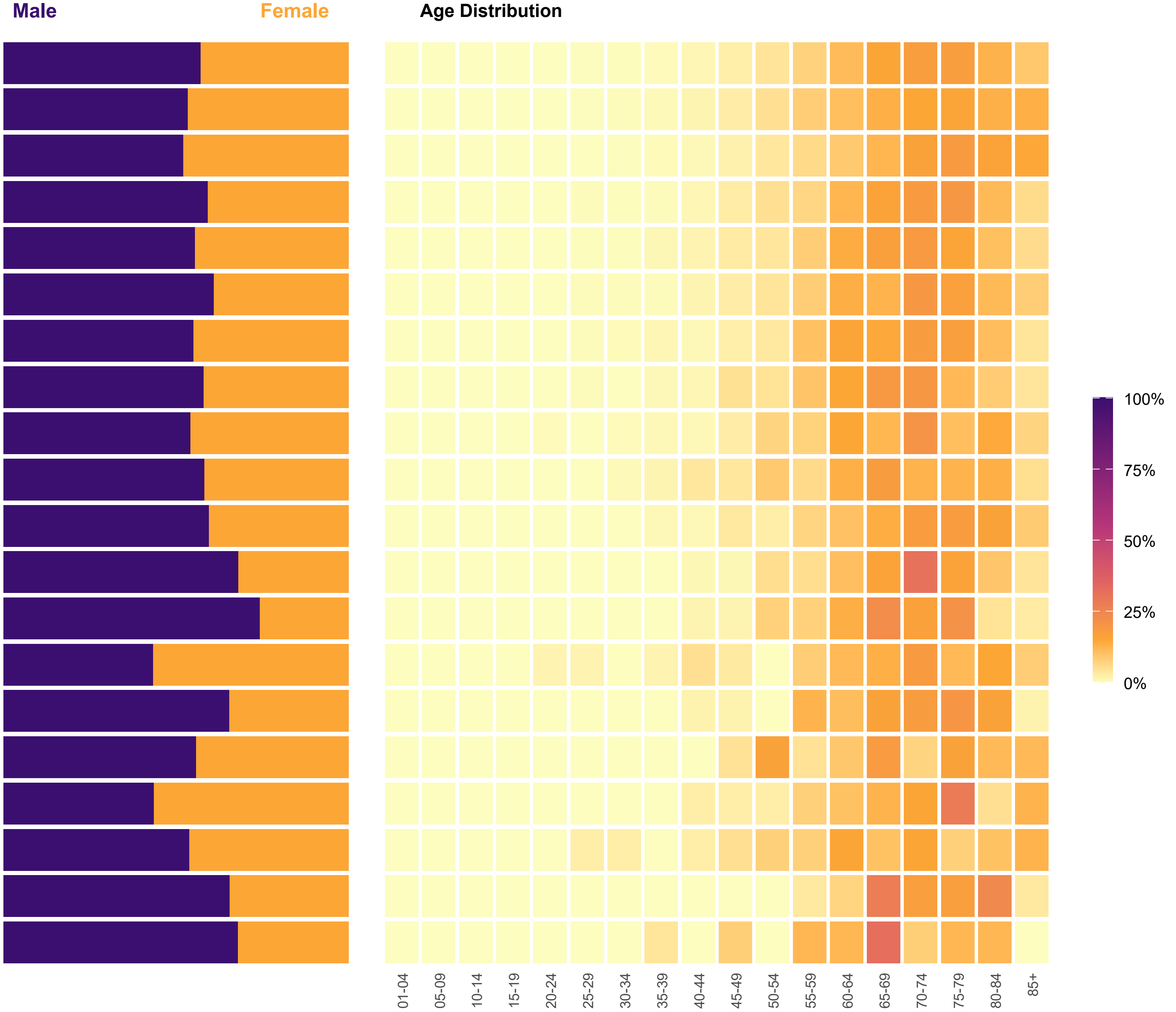

Primary Site: Extrahepatic Bile Ducts | Phenotype: Grouped Phenotypes

Top 3 Morphologies | cases: 276

Morphology	n	%
Neuroendocrine tumor	100	36.2%
Small cell carcinoma	53	19.2%
Mixed adenoneuroendocrine carcinoma	22	8.0%

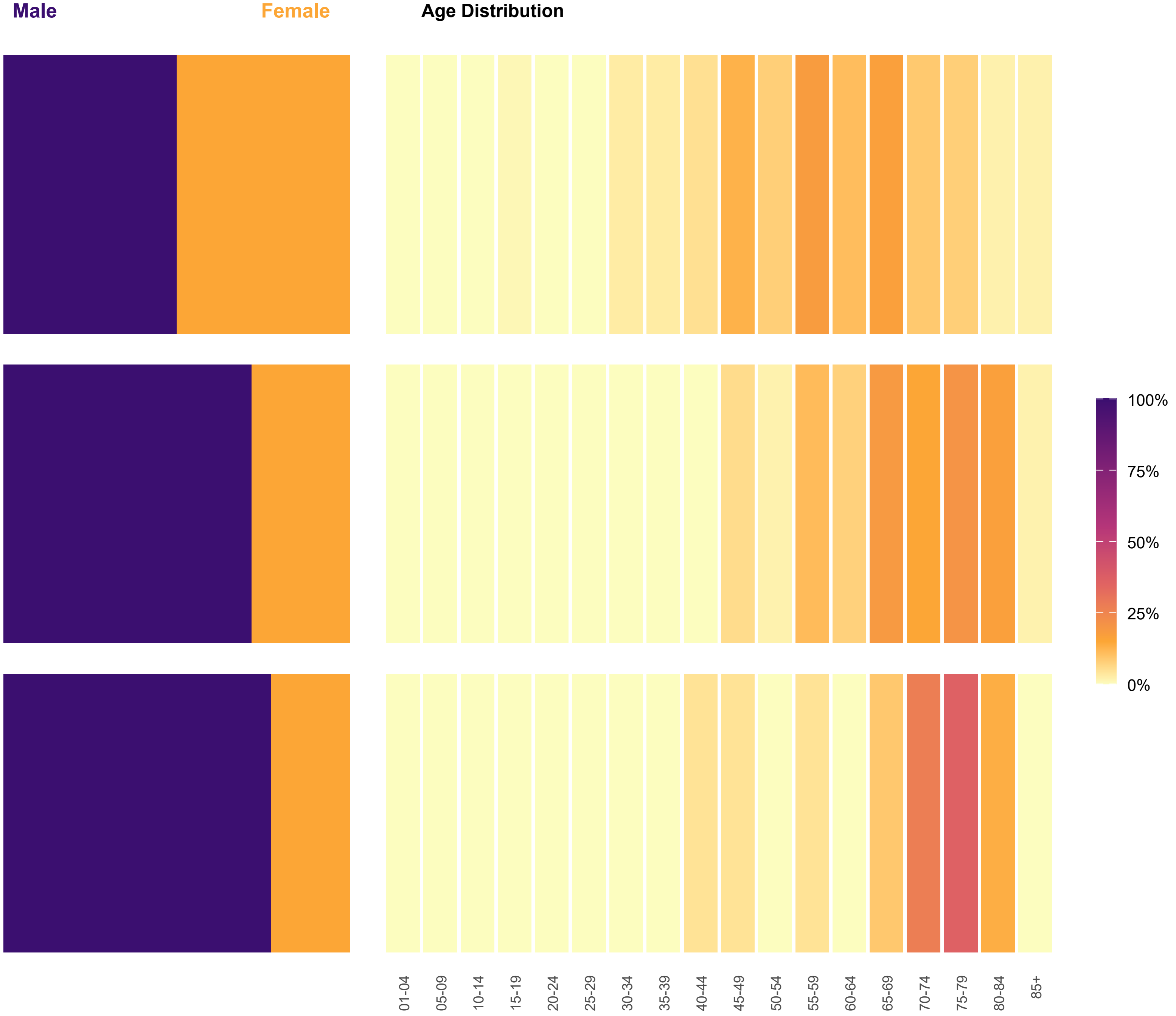

Primary Site: Eye and Orbit | Phenotype: epithelial

Top 10 Morphologies | cases: 4,629

Morphology	n	%
Squamous cell carcinoma	3,630	78.4%
Adenoid cystic carcinoma	292	6.3%
Adenocarcinoma	188	4.1%
Mucoepidermoid carcinoma	116	2.5%
Sebaceous carcinoma	75	1.6%
Basal cell carcinoma	55	1.2%
Papillary squamous cell carcinoma	41	0.9%
Basaloid squamous cell carcinoma	39	0.8%
Neuroendocrine carcinoma	24	0.5%
Carcinoma ex pleomorphic adenoma	24	0.5%

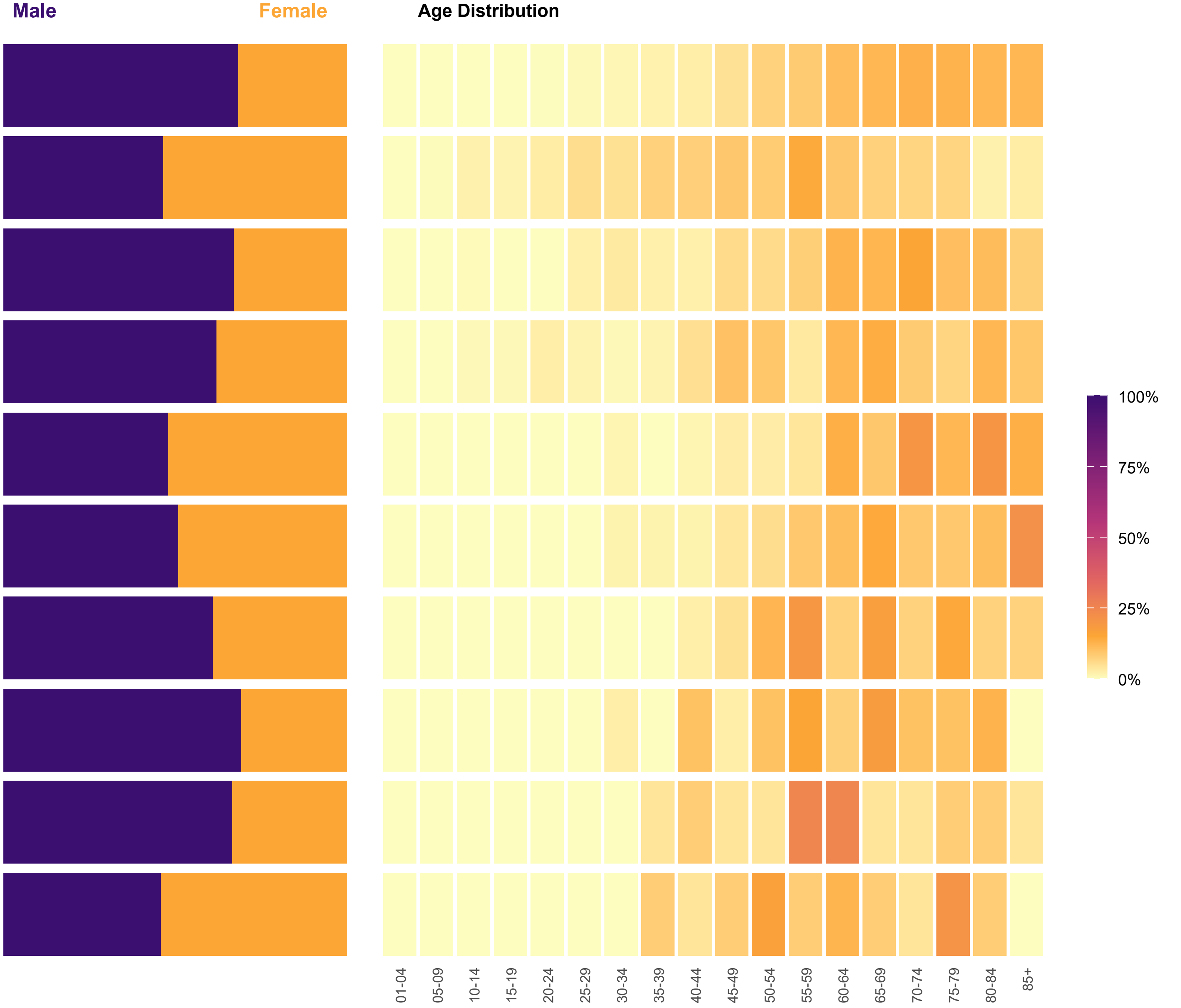

Primary Site: Eye and Orbit | Phenotype: Grouped Phenotypes

Top 14 Morphologies | cases: 30,244

Morphology	n	%
Malignant melanoma	23,644	78.2%
Retinoblastoma	1,832	6.1%
Spindle cell melanoma	1,380	4.6%
Malignant Spitz tumor	1,237	4.1%
Epithelioid cell melanoma	588	1.9%
Spindle cell melanoma type B	531	1.8%
Amelanotic melanoma	288	1.0%
Nodular melanoma	161	0.5%
Retinoblastomaundifferentiated	135	0.4%
Spindle cell melanoma type A	91	0.3%
Retinoblastoma differentiated	58	0.2%
Low cumulative sun damage melanoma	44	0.1%
Malignant melanoma in precancerous…	43	0.1%
Mucosal lentiginous melanoma	33	0.1%

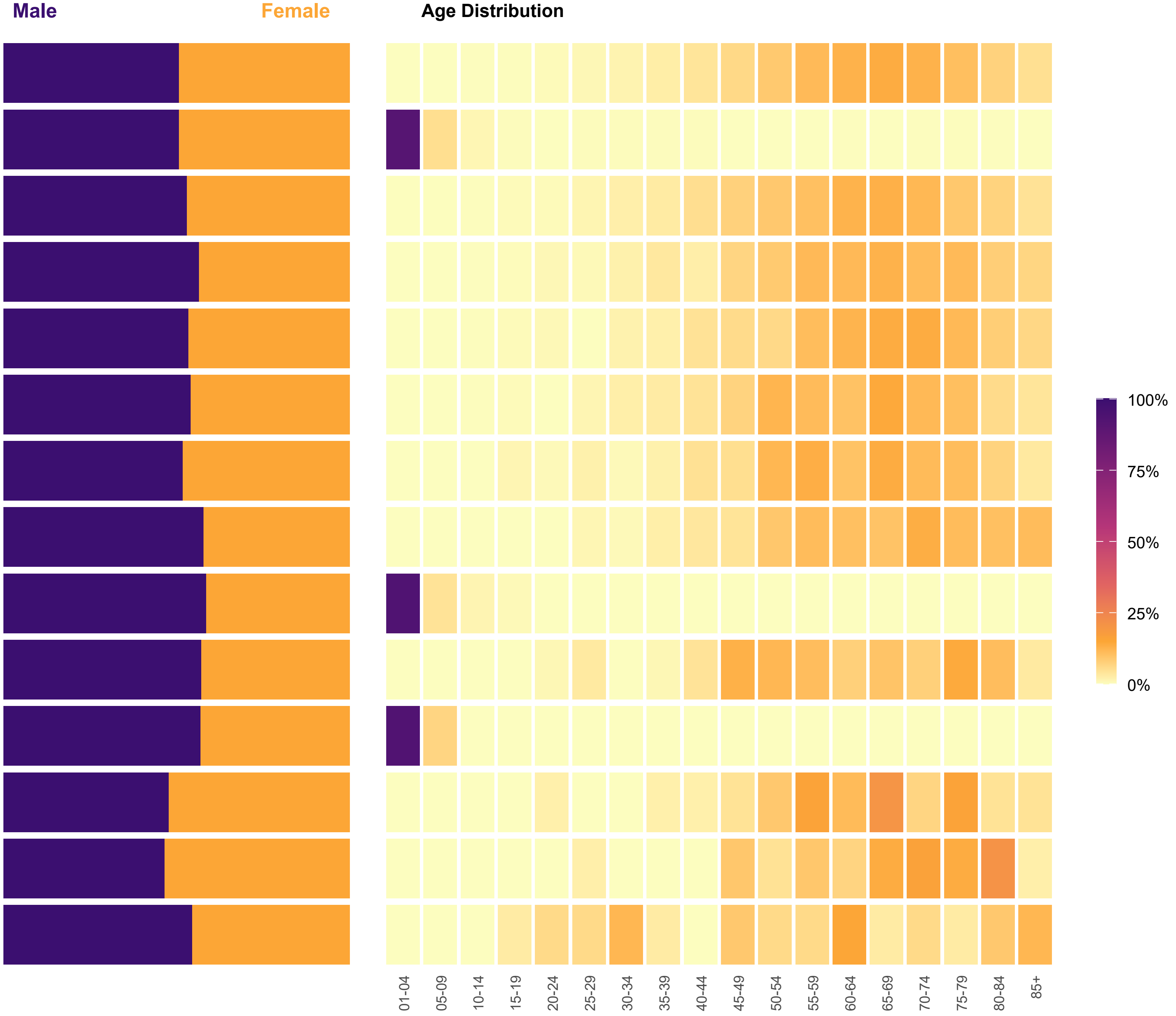

Primary Site: Fallopian Tube | Phenotype: epithelial

Top 14 Morphologies | cases: 17,054

Morphology	n	%
Serous carcinoma	8,040	47.1%
Low grade serous carcinoma	2,617	15.3%
High grade serous carcinoma	2,463	14.4%
Adenocarcinoma	1,798	10.5%
Endometrioid adenocarcinoma	976	5.7%
Papillary adenocarcinoma	412	2.4%
Mixed cell adenocarcinoma	324	1.9%
Clear cell adenocarcinoma	96	0.6%
Transitional cell carcinoma	57	0.3%
Mucinous adenocarcinoma	49	0.3%
Papillary cystadenocarcinoma	39	0.2%
Cystadenocarcinoma	37	0.2%
Squamous cell carcinoma	23	0.1%
Adenocarcinoma with mixed subtypes	20	0.1%

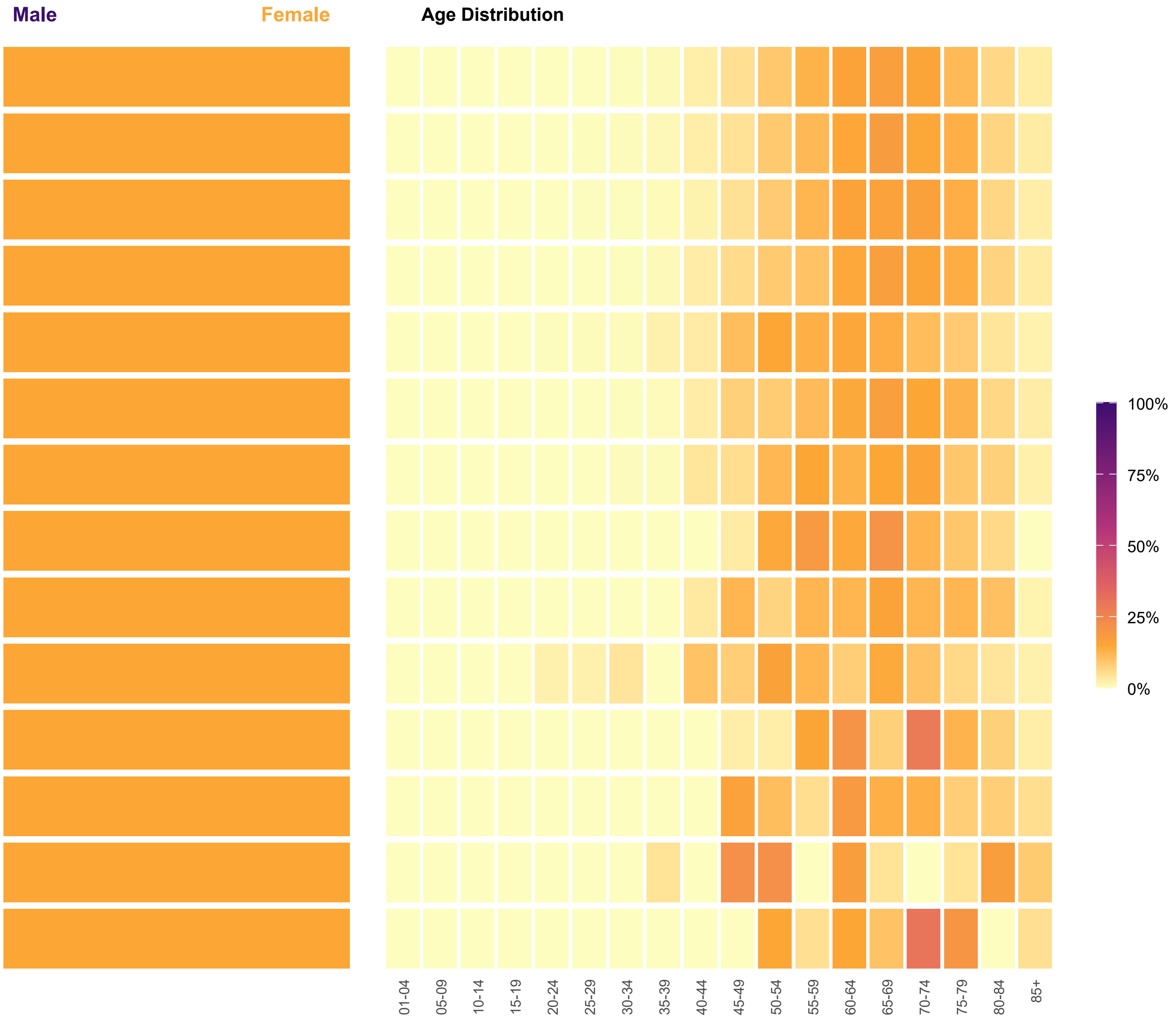

Primary Site: Fallopian Tube | Phenotype: Grouped Phenotypes

Top 3 Morphologies | cases: 667

Morphology	n	%
Carcinosarcoma	352	52.8%
Mulerian mixed tumor	180	27.0%
Choriocarcinoma	34	5.1%

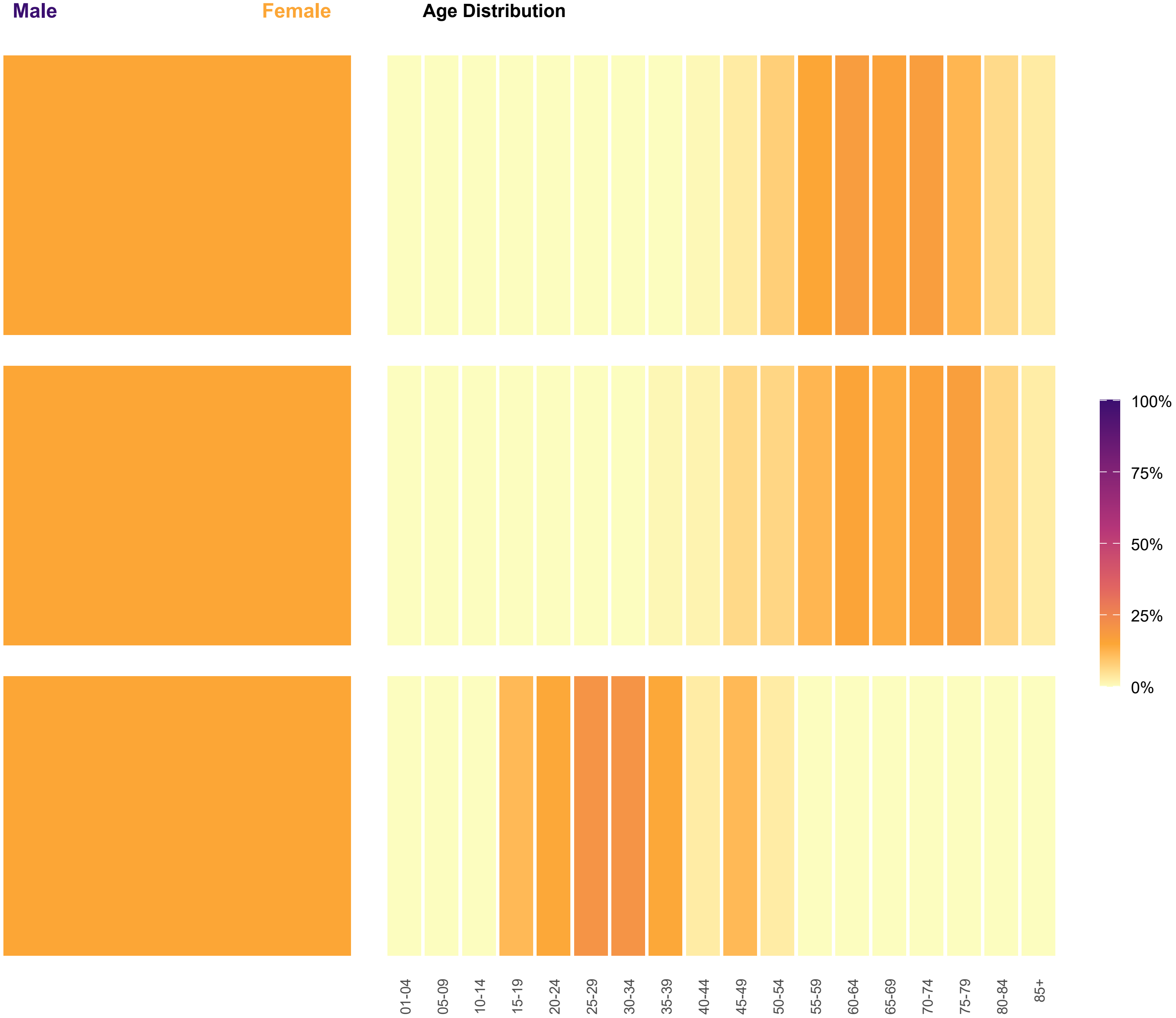

Primary Site: Floor of Mouth | Phenotype: epithelial

Top 6 Morphologies | cases: 37,252

Morphology	n	%
Squamous cell carcinoma	36,084	96.9%
Basaloid squamous cell carcinoma	328	0.9%
Mucoepidermoid carcinoma	307	0.8%
Adenoid cystic carcinoma	245	0.7%
Adenocarcinoma	113	0.3%
Papillary squamous cell carcinoma	70	0.2%

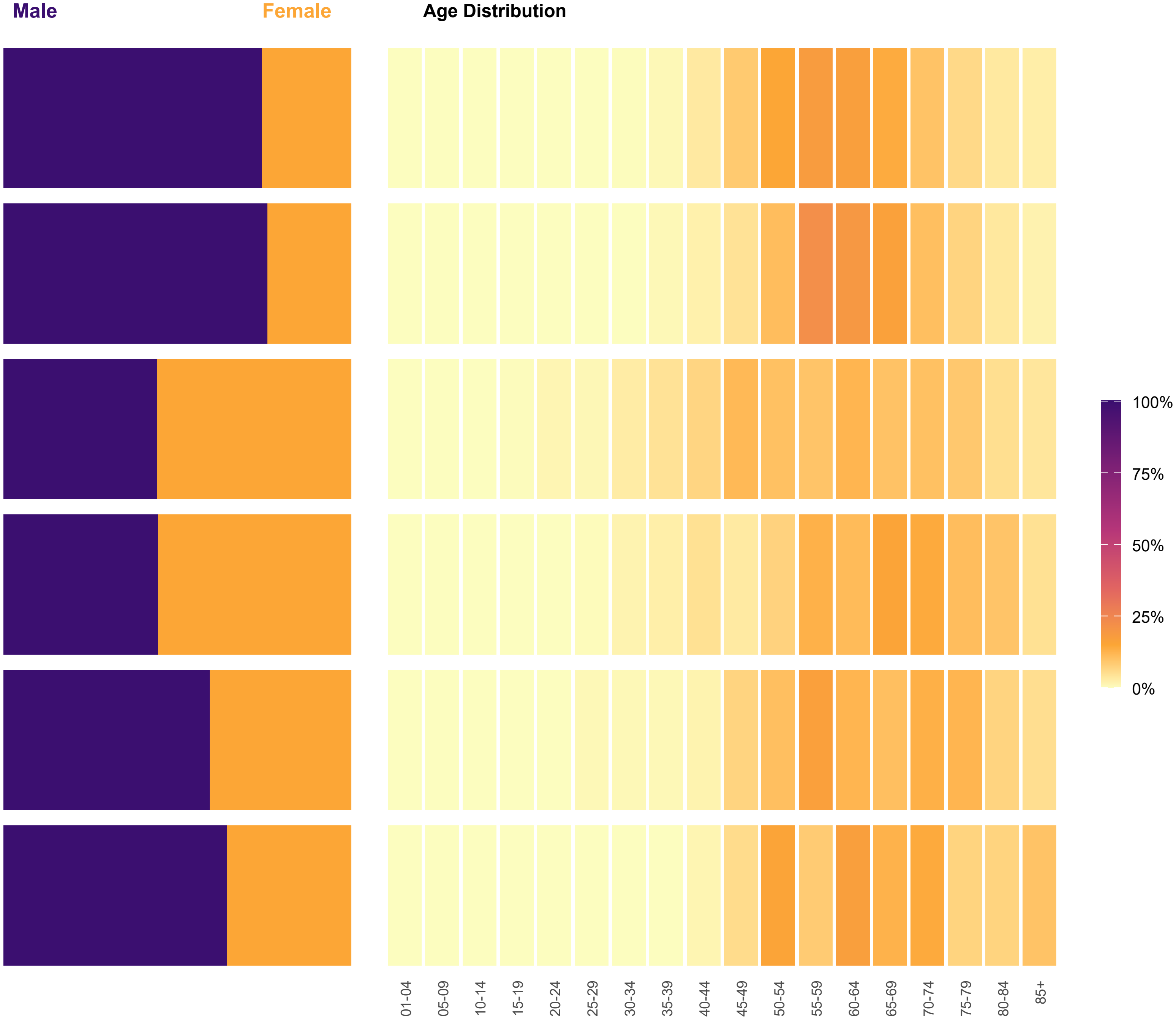

Primary Site: Floor of Mouth | Phenotype: Grouped Phenotypes

Top 1 Morphologies | cases: 161

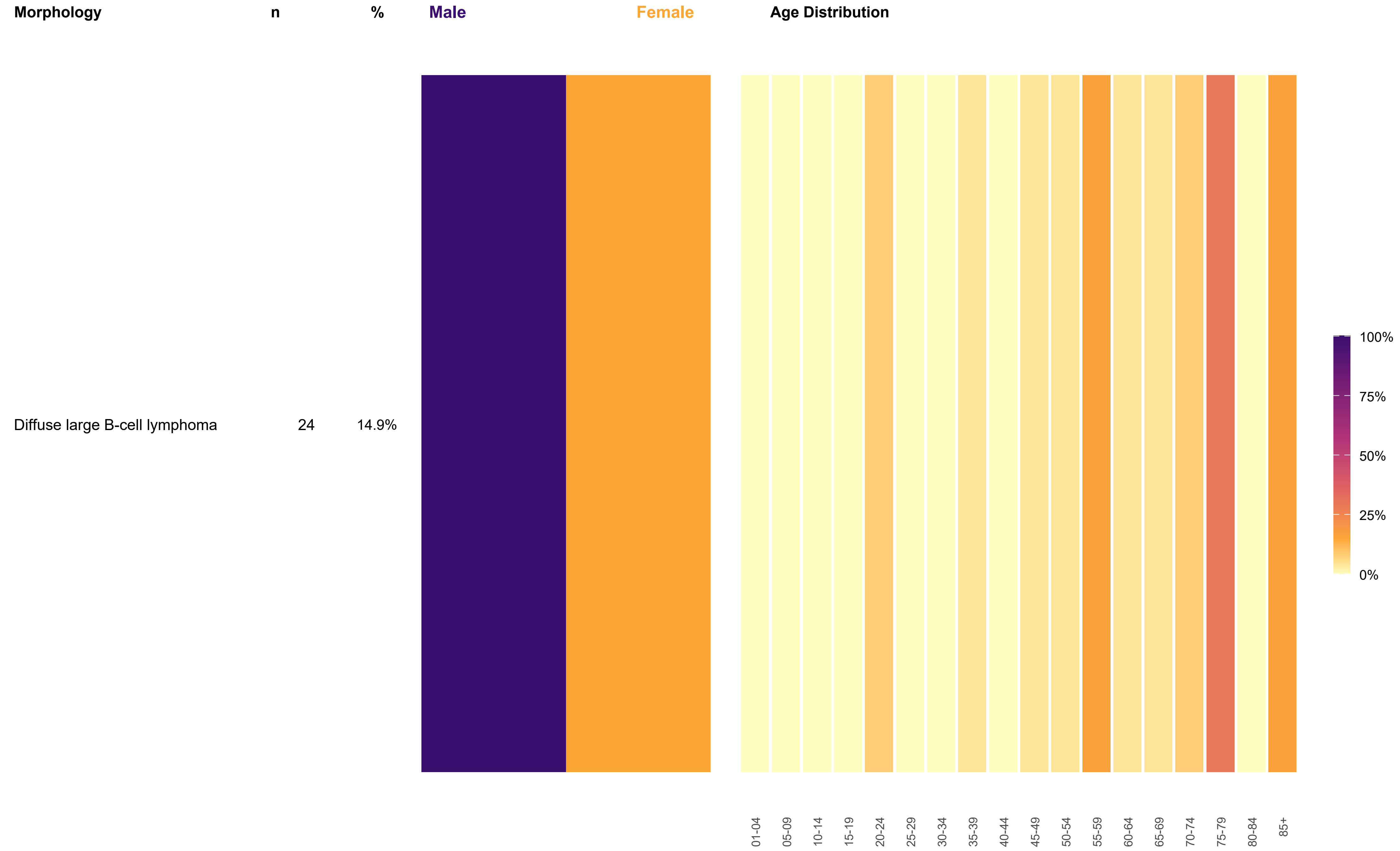

Primary Site: Gallbladder | Phenotype: epithelial

Top 20 Morphologies | cases: 48,784

Morphology	n	%
Adenocarcinoma	39,124	80.2%
Cholangiocarcinoma	2,136	4.4%
Mucinous adenocarcinoma	1,283	2.6%
Papillary adenocarcinoma	1,265	2.6%
Signet ring cell carcinoma	808	1.7%
Squamous cell carcinoma	778	1.6%
Mucin-producing adenocarcinoma	755	1.5%
Tubular adenocarcinoma	493	1.0%
Neuroendocrine carcinoma	432	0.9%
Adenocarcinoma intestinal type	289	0.6%
Adenocarcinoma in tubulovillous…	284	0.6%
Infiltrating duct carcinoma	199	0.4%
Adenocarcinoma with mixed subtypes	197	0.4%
Adenocarcinoma in adenomatous polyp	140	0.3%
Clear cell adenocarcinoma	99	0.2%
Adenocarcinoma with neuroendocrine…	83	0.2%
Scirrhous adenocarcinoma	69	0.1%
Intraductal papillary…	62	0.1%
Adenocarcinoma in villous adenoma	54	0.1%
Pancreatobiliary type carcinoma	44	<0.1%

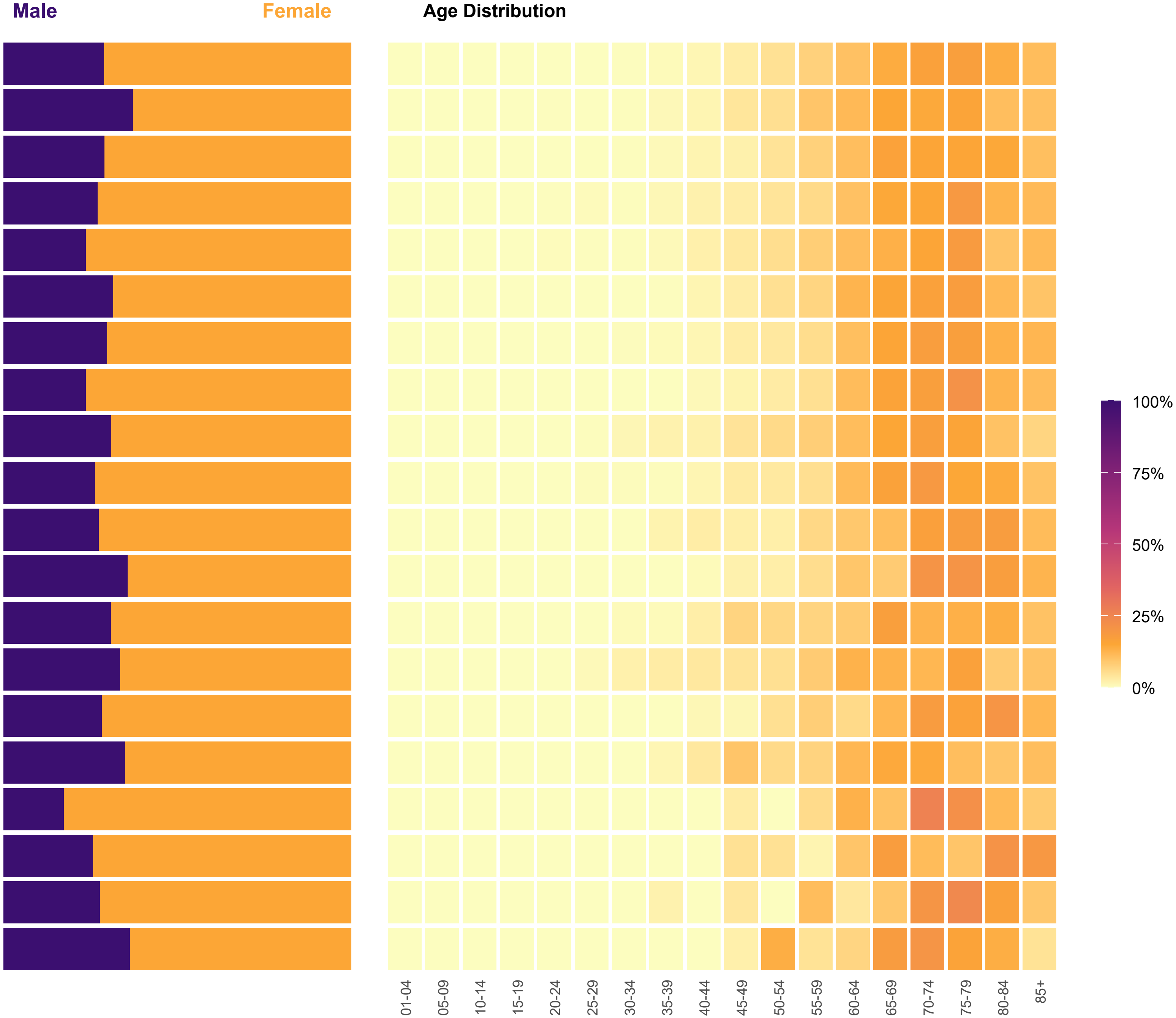

Primary Site: Gallbladder | Phenotype: Grouped Phenotypes

Top 13 Morphologies | cases: 1,347

Morphology	n	%
Small cell carcinoma	333	24.7%
Neuroendocrine tumor	257	19.1%
Carcinosarcoma	109	8.1%
Large cell neuroendocrine carcinoma	83	6.2%
Mixed adenoneuroendocrine carcinoma	76	5.6%
Diffuse large B-cell lymphoma	73	5.4%
Marginal zone B-cell lymphoma	51	3.8%
Large cell carcinoma	46	3.4%
lymphoma non-Hodgkin	31	2.3%
Combined small cell carcinoma	27	2.0%
Malignant melanoma	27	2.0%
Follicular lymphoma grade 1/2	27	2.0%
B-cell chronic lymphocytic…	23	1.7%

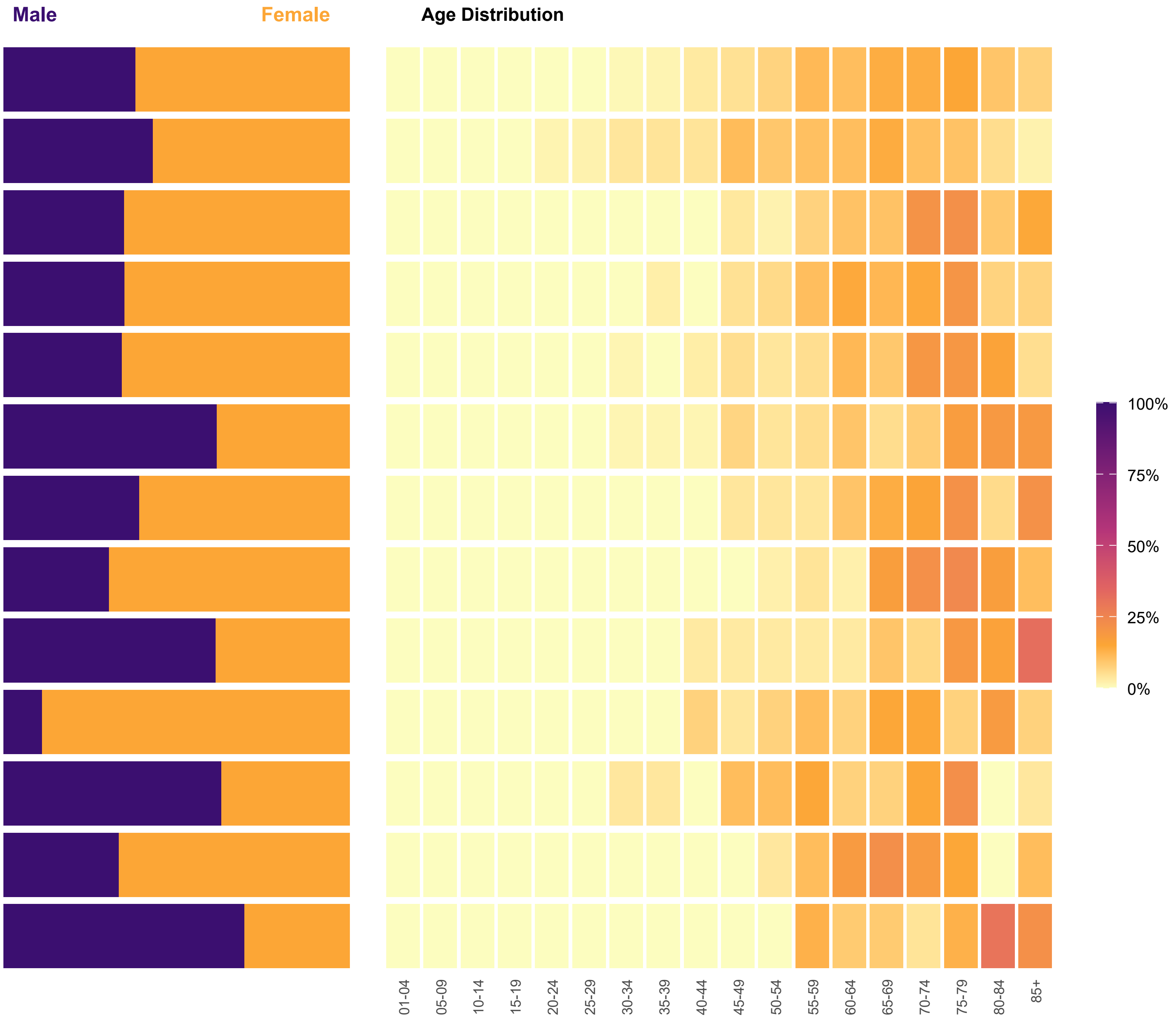

Primary Site: Gum | Phenotype: epithelial

Top 7 Morphologies | cases: 27,208

Morphology	n	%
Squamous cell carcinoma	25,538	93.9%
Mucoepidermoid carcinoma	664	2.4%
Adenoid cystic carcinoma	268	1.0%
Papillary squamous cell carcinoma	234	0.9%
Basaloid squamous cell carcinoma	155	0.6%
Adenocarcinoma	140	0.5%
Polymorphous adenocarcinoma	64	0.2%

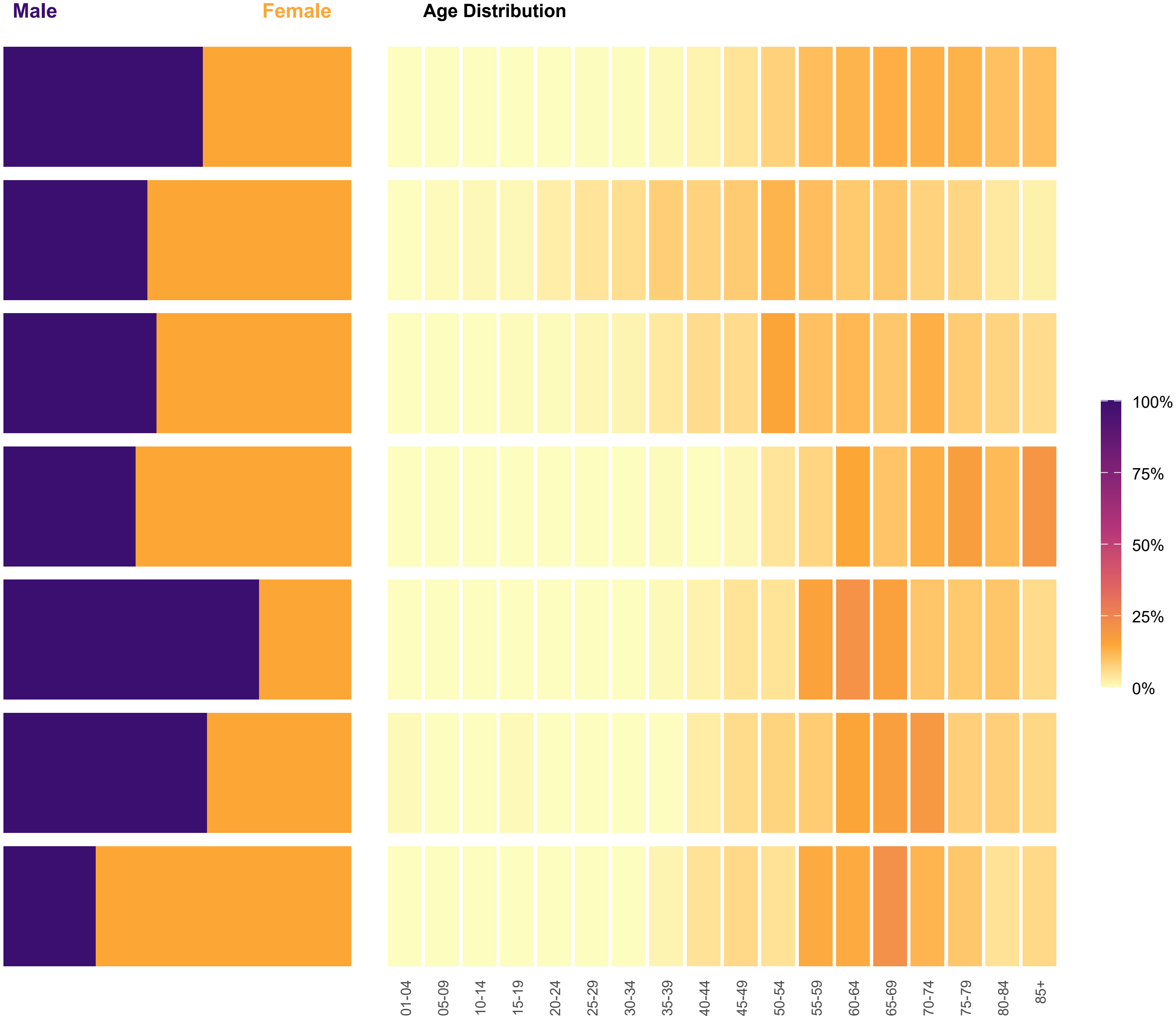

Primary Site: Gum | Phenotype: Grouped Phenotypes

Top 11 Morphologies | cases: 936

Morphology	n	%
Diffuse large B-cell lymphoma	201	21.5%
Malignant melanoma	172	18.4%
Kaposi sarcoma	66	7.1%
Plasmablastic lymphoma	24	2.6%
lymphoma non-Hodgkin	23	2.5%
Spindle cell melanoma	22	2.4%
Lymphoma large B-cell diffuse…	22	2.4%
Plasmacytoma extramedullary	22	2.4%
Nodular melanoma	20	2.1%
Myoepithelial carcinoma	20	2.1%
Hemangiosarcoma	20	2.1%

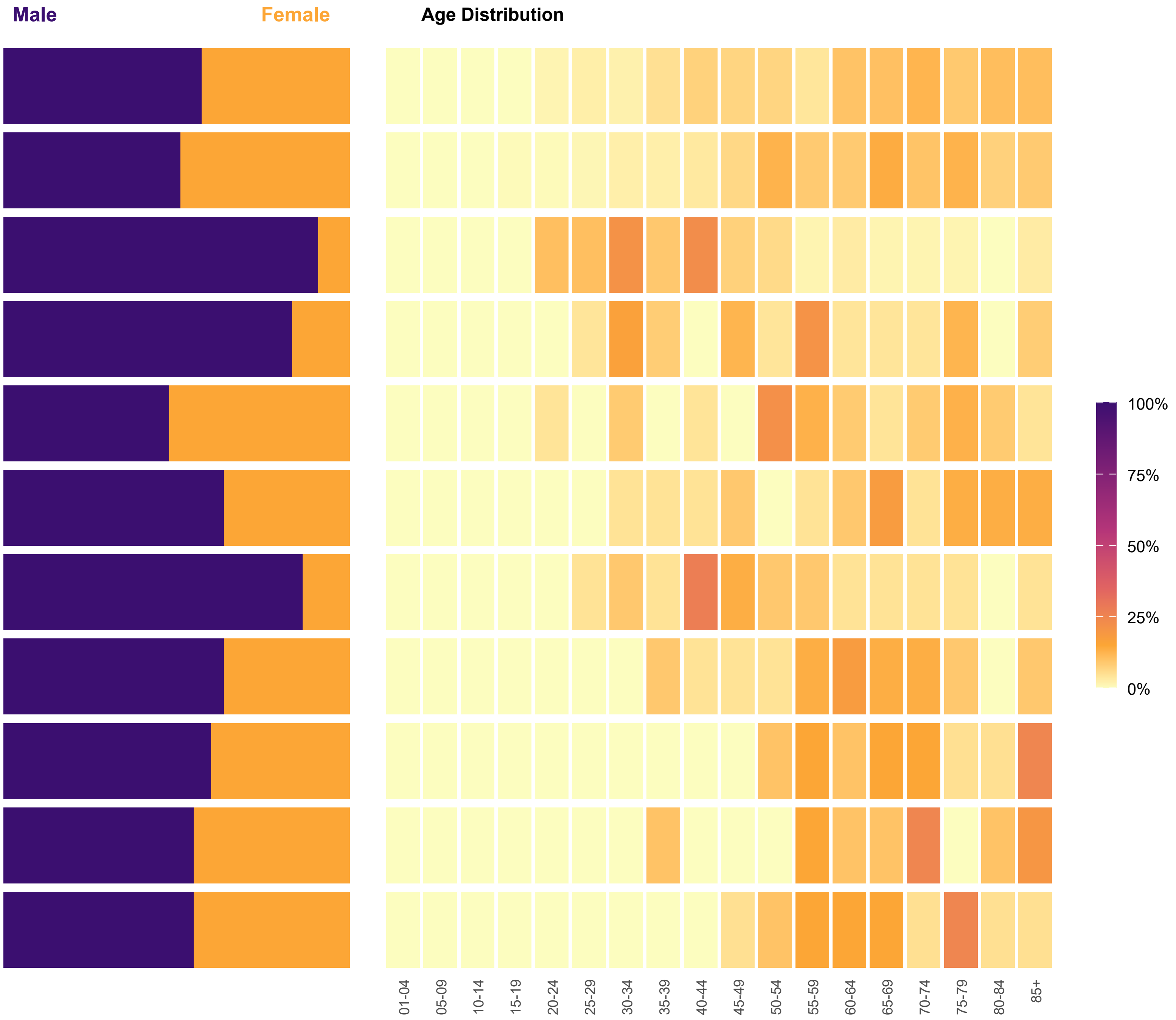

Primary Site: Heart | Phenotype: epithelial

Top 5 Morphologies | cases: 940

Morphology	n	%
Adenocarcinoma	343	36.5%
Neuroendocrine carcinoma	190	20.2%
Squamous cell carcinoma	171	18.2%
Thymoma	66	7.0%
Thymoma type AB	38	4.0%

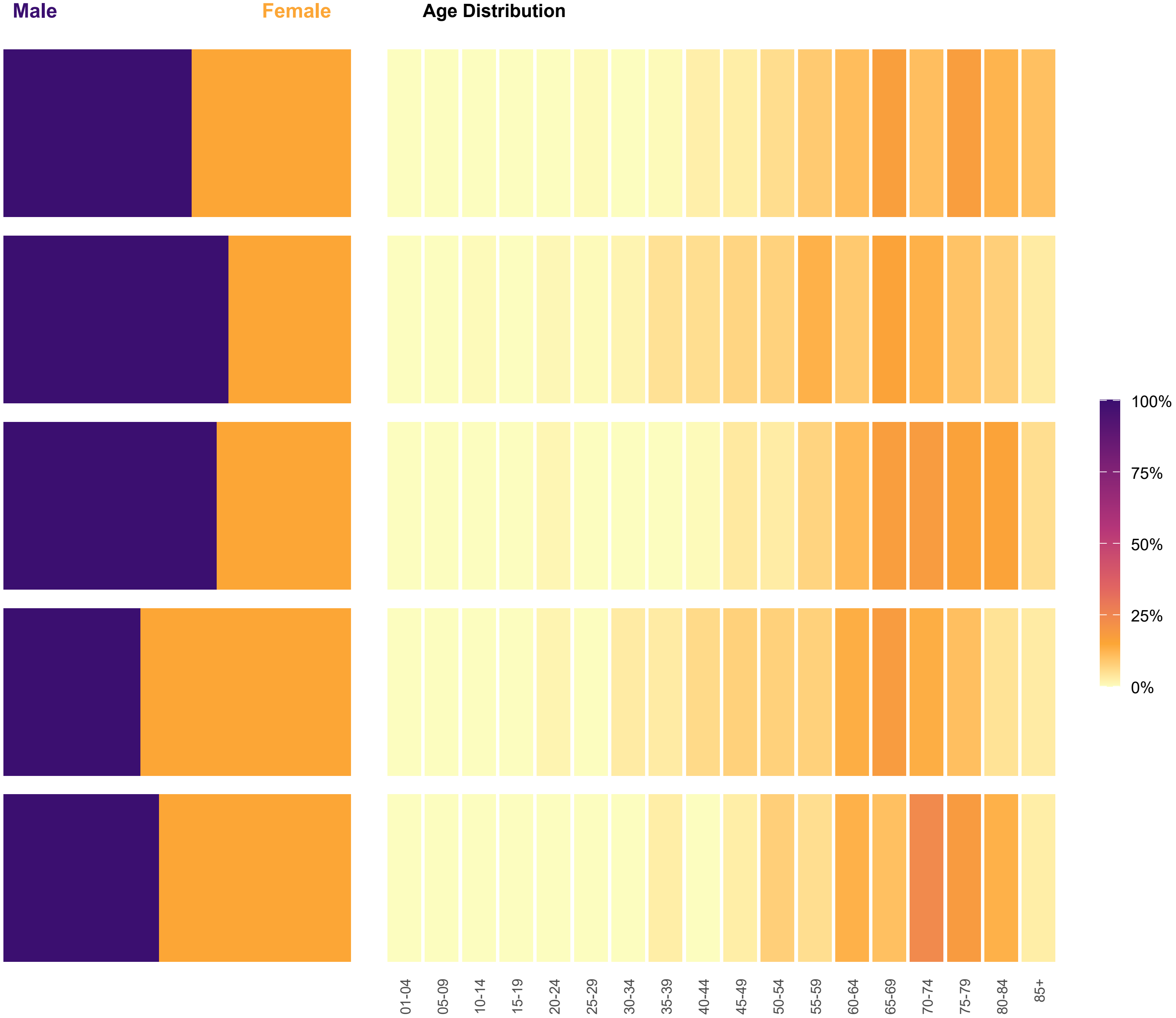

Primary Site: Heart | Phenotype: Grouped Phenotypes

Top 19 Morphologies | cases: 2,673

Morphology	n	%
Seminoma	433	16.2%
Germinoma	398	14.9%
Mixed germ cell tumor	309	11.6%
Yolk sac tumor	259	9.7%
Germ cell tumor nonseminomatous	196	7.3%
Neuroblastoma	181	6.8%
Ganglioneuroblastoma	125	4.7%
Teratoma	119	4.5%
Choriocarcinoma	86	3.2%
Malignant peripheral nerve sheath…	63	2.4%
Embryonal carcinoma	62	2.3%
Small cell carcinoma	59	2.2%
Neuroendocrine tumor	56	2.1%
Pleuropulmonary blastoma	32	1.2%
Choriocarcinoma combined with…	31	1.2%
Paraganglioma	30	1.1%
Neuroendocrine tumor grade 2	27	1.0%
Large cell neuroendocrine carcinoma	27	1.0%
Teratocarcinoma	26	1.0%

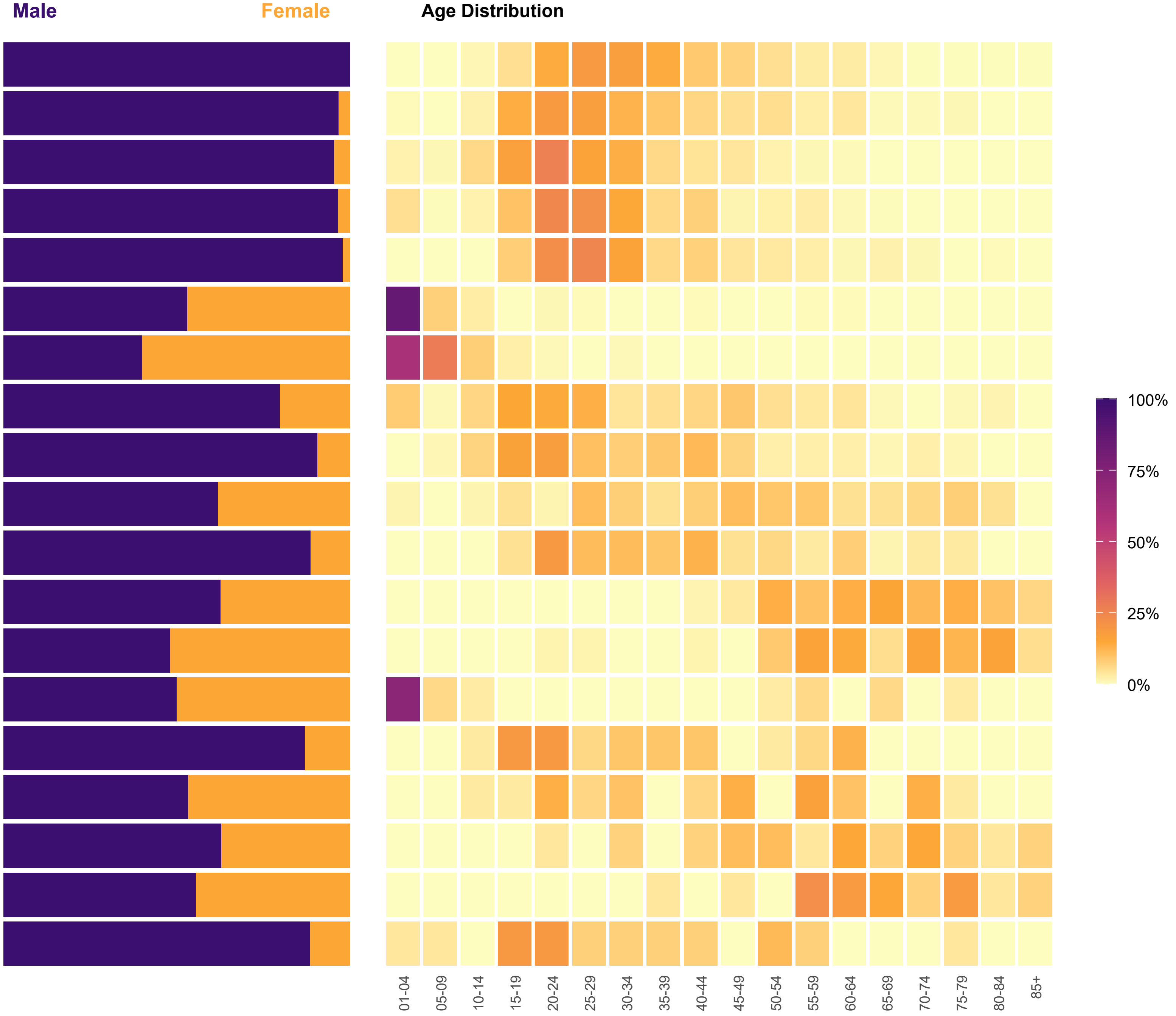

Primary Site: Hematopoetic and reticuloendothelial | Phenotype: epithelial

Top 25 Morphologies | cases: 178,368

Morphology	n	%
Adenocarcinoma	99,991	56.1%
Squamous cell carcinoma	44,295	24.8%
Neuroendocrine carcinoma	14,775	8.3%
Mucinous adenocarcinoma	4,666	2.6%
Signet ring cell carcinoma	2,722	1.5%
Mucin-producing adenocarcinoma	2,633	1.5%
Papillary adenocarcinoma	1,445	0.8%
Clear cell adenocarcinoma	914	0.5%
Basaloid squamous cell carcinoma	693	0.4%
Serous carcinoma	595	0.3%
Transitional cell carcinoma	590	0.3%
Infiltrating duct carcinoma	555	0.3%
Tubular adenocarcinoma	540	0.3%
Adenocarcinoma with neuroendocrine…	415	0.2%
Low grade serous carcinoma	356	0.2%
Adenoid cystic carcinoma	266	0.1%
Mucoepidermoid carcinoma	265	0.1%
Lymphoepithelial carcinoma	255	0.1%
Adenocarcinoma with mixed subtypes	187	0.1%
Cholangiocarcinoma	166	<0.1%
Adenocarcinoma intestinal type	139	<0.1%
Merkel cell carcinoma	135	<0.1%
Endometrioid adenocarcinoma	128	<0.1%
Acinar cell carcinoma	127	<0.1%
Trabecular adenocarcinoma	127	<0.1%

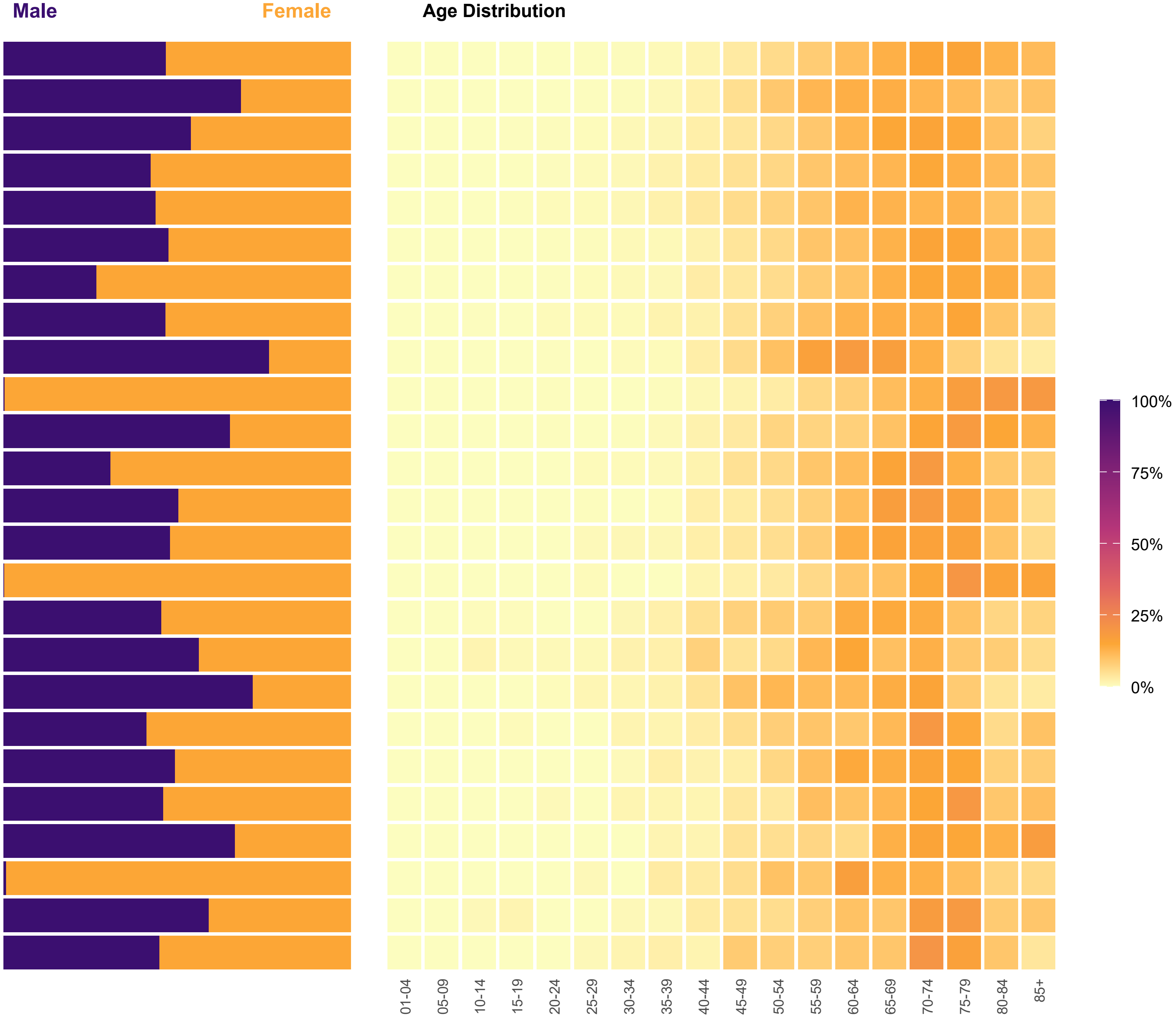

Primary Site: Hematopoetic and reticuloendothelial | Phenotype: germ cell

Top 7 Morphologies | cases: 817

Morphology	n	%
Germinoma	255	31.2%
Choriocarcinoma	143	17.5%
Embryonal carcinoma	97	11.9%
Seminoma	88	10.8%
Yolk sac tumor	86	10.5%
Mixed germ cell tumor	43	5.3%
Germ cell tumor nonseminomatous	42	5.1%

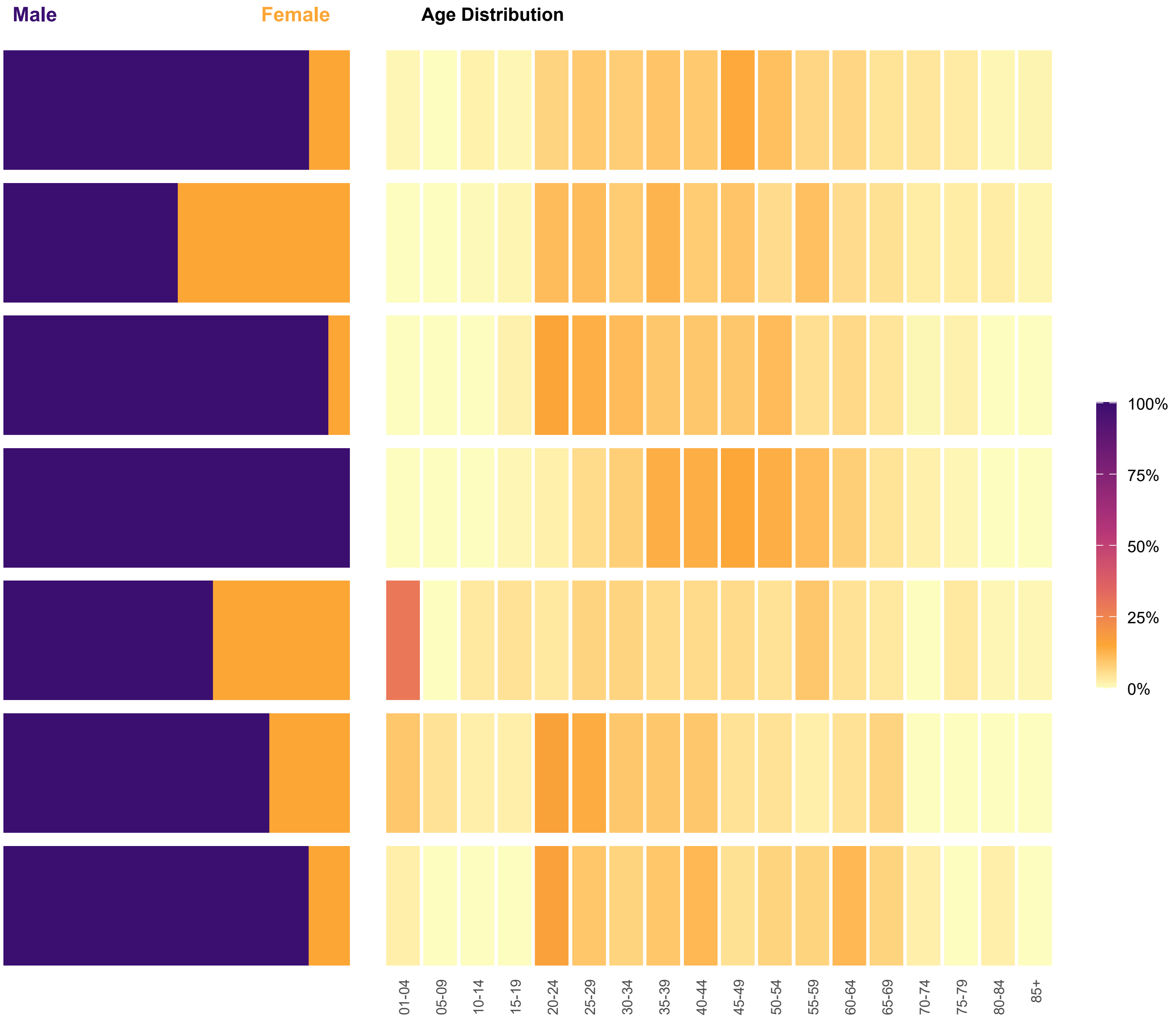

Primary Site: Hematopoetic and reticuloendothelial | Phenotype: hematopoietic

Top 25 Morphologies | cases: 1,241,685

Morphology	n	%
Plasma cell myeloma	177,738	14.3%
B-cell chronic lymphocytic…	154,538	12.4%
Diffuse large B-cell lymphoma	123,995	10.0%
Myelodysplastic syndrome	77,197	6.2%
Acute myeloid leukemia	66,216	5.3%
lymphoma non-Hodgkin	48,029	3.9%
Follicular lymphoma grade 1/2	43,848	3.5%
Polycythemia vera	41,921	3.4%
Essential thrombocythemia	40,047	3.2%
Hodgkin lymphoma nodular sclerosis	34,094	2.7%
Chronic myeloid leukemia	24,320	2.0%
Follicular lymphoma	24,285	2.0%
lymphoma	22,128	1.8%
Hodgkin lymphoma	19,505	1.6%
Marginal zone B-cell lymphoma	19,298	1.6%
B lymphoblastic leukemia/lymphoma	19,250	1.6%
Mantle cell lymphoma	19,036	1.5%
Myeloproliferative neoplasm	16,079	1.3%
Myelodysplastic syndrome with…	15,423	1.2%
Follicular lymphoma grade 3	14,012	1.1%
Chronic myelogenous leukemia	13,560	1.1%
Chronic myelomonocytic leukemia	11,958	1.0%
Waldenstrom macroglobulinemia	11,333	0.9%
Precursor cell lymphoblastic…	9,731	0.8%
Myelodysplastic syndrome with…	9,174	0.7%

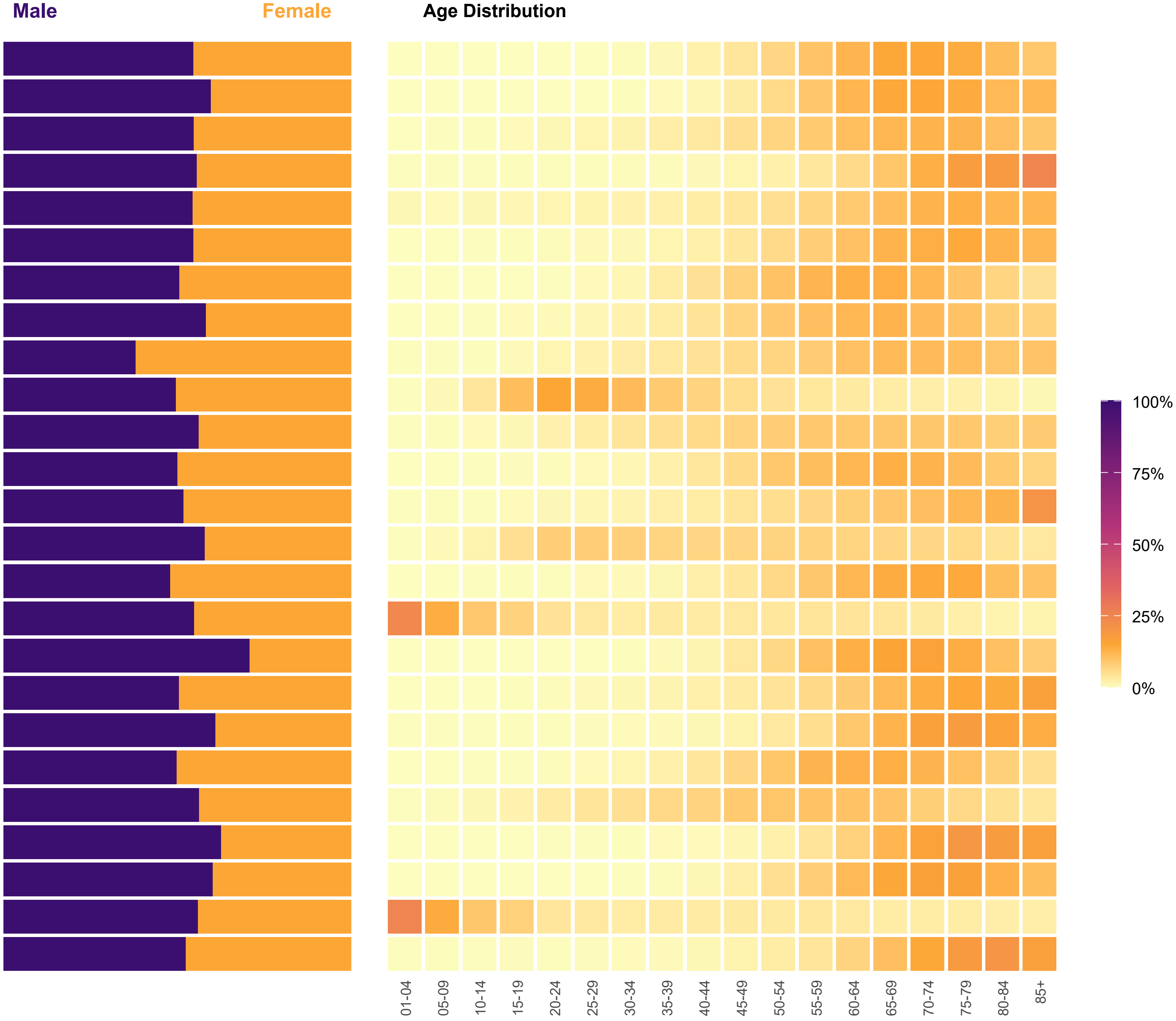

Primary Site: Hematopoetic and reticuloendothelial | Phenotype: mesenchymal

Top 25 Morphologies | cases: 4,728

Morphology	n	%
Mesotheliomamalignant	871	18.4%
Sarcoma	736	15.6%
Kaposi sarcoma	459	9.7%
Leiomyosarcoma	446	9.4%
Hemangiosarcoma	434	9.2%
Gastrointestinal stromal tumor	388	8.2%
Spindle cell sarcoma	147	3.1%
Epithelioid mesothelioma	142	3.0%
Malignant fibrous histiocytoma	99	2.1%
Desmoplastic small round cell tumor	96	2.0%
Giant cell sarcoma	75	1.6%
Ewing sarcoma	72	1.5%
Epithelioid hemangioendothelioma	65	1.4%
Liposarcoma	58	1.2%
Undifferentiated sarcoma	49	1.0%
Rhabdomyosarcoma	44	0.9%
Fibrous mesothelioma	43	0.9%
Epithelioid sarcoma	36	0.8%
Synovial sarcoma	34	0.7%
Solitary fibrous tumormalignant	29	0.6%
Fibrosarcoma	27	0.6%
Dedifferentiated liposarcoma	27	0.6%
Mesothelioma biphasic	25	0.5%
Chondrosarcoma	23	0.5%
Hemangioendothelioma	21	0.4%

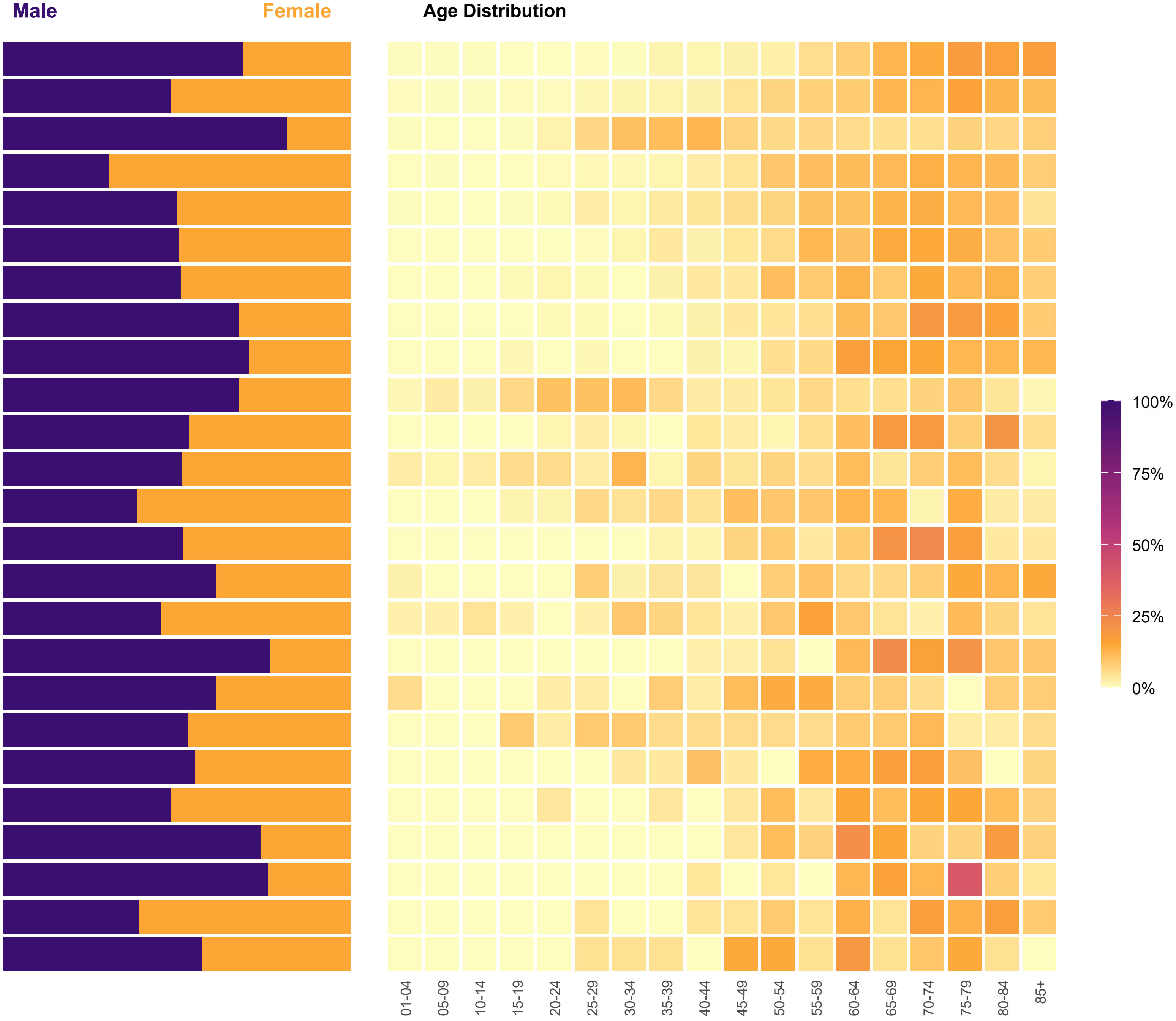

Primary Site: Hematopoetic and reticuloendothelial | Phenotype: mixed or multipotent stem cell

Top 9 Morphologies | cases: 786

Morphology	n	%
Carcinosarcoma	308	39.2%
Mullerian mixed tumor	92	11.7%
Teratoma	67	8.5%
Mixed adenoneuroendocrine carcinoma	62	7.9%
Myoepithelial carcinoma	48	6.1%
Chordoma	38	4.8%
Goblet cell carcinoid	30	3.8%
Epithelial-myoepithelial carcinoma	28	3.6%
Rhabdoid tumor	27	3.4%

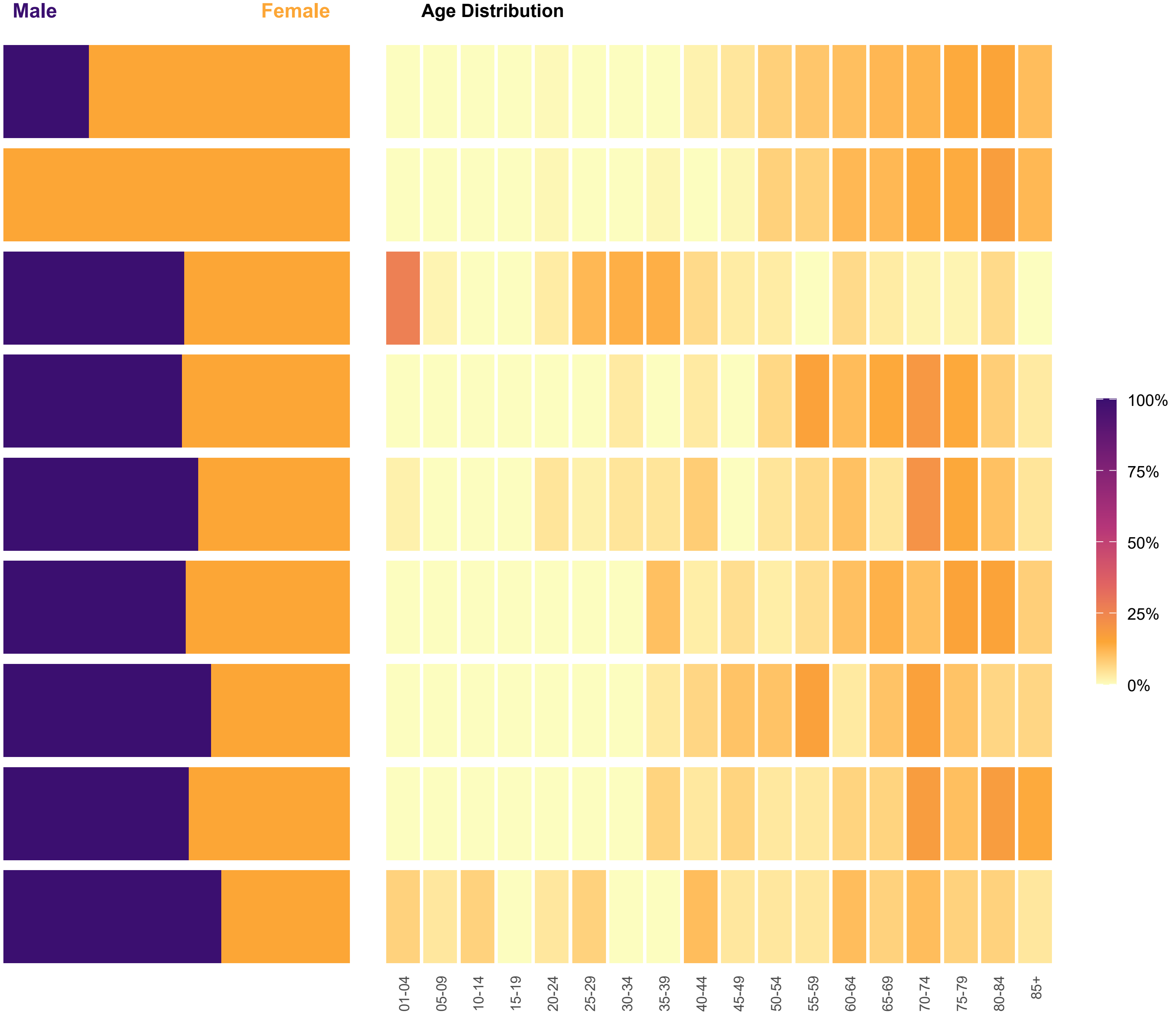

Primary Site: Hematopoetic and reticuloendothelial | Phenotype: neuroectodermal

Top 19 Morphologies | cases: 20,845

Morphology	n	%
Small cell carcinoma	8,461	40.6%
Neuroendocrine tumor	4,716	22.6%
Malignant melanoma	2,284	11.0%
Large cell carcinoma	2,184	10.5%
Large cell neuroendocrine carcinoma	1,212	5.8%
Neuroendocrine tumor grade 2	919	4.4%
Amelanotic melanoma	234	1.1%
Neuroblastoma	160	0.8%
Oat cell carcinoma	144	0.7%
Combined small cell carcinoma	83	0.4%
Paraganglioma	58	0.3%
CNS embryonal tumor	52	0.2%
Malignant peripheral nerve sheath…	52	0.2%
Gastrinoma	38	0.2%
Epithelioid cell melanoma	29	0.1%
Spindle cell melanoma	27	0.1%
Enterochromaffin cell carcinoid	25	0.1%
Nodular melanoma	22	0.1%
Neurilemomamalignant	20	<0.1%

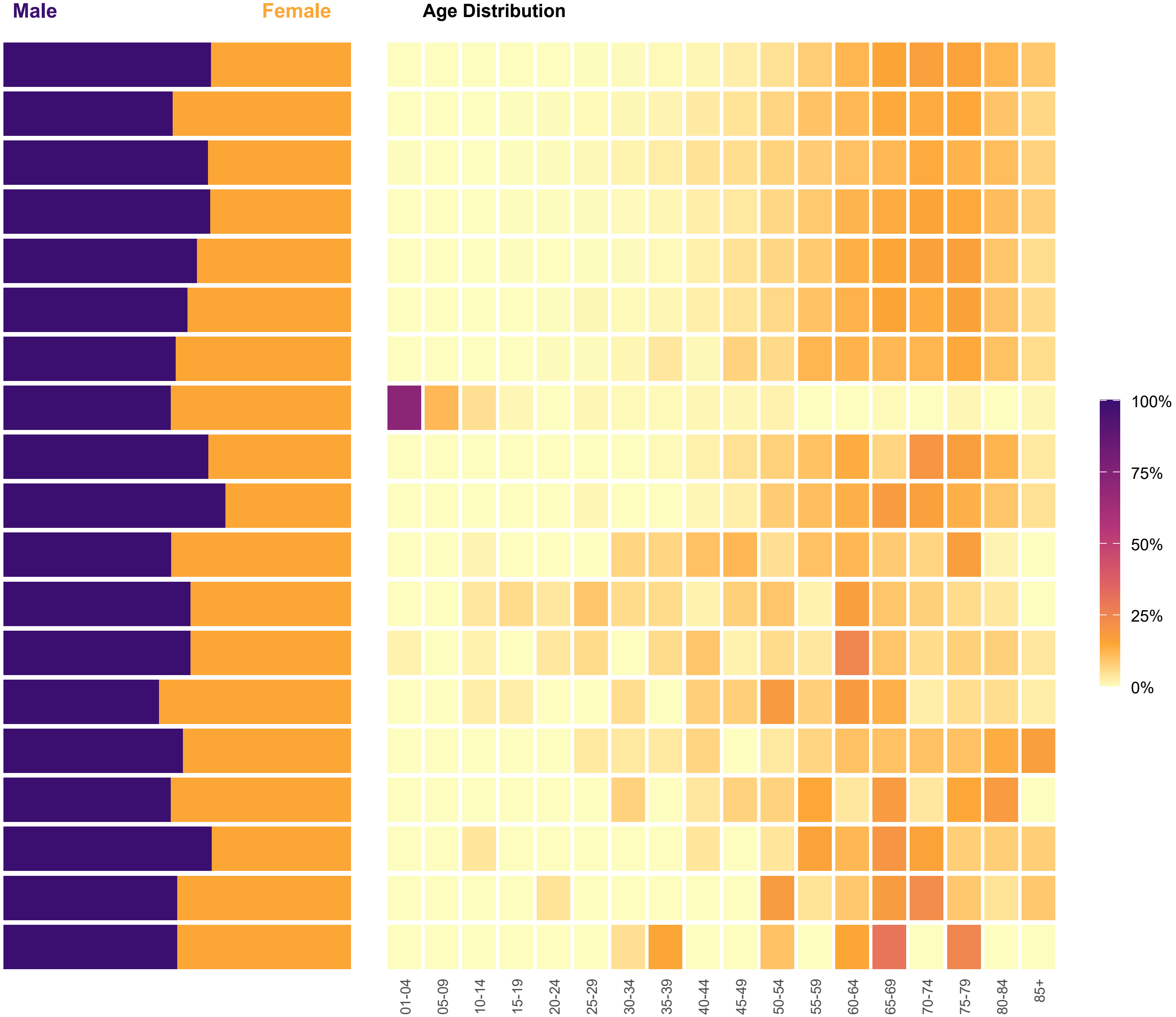

Primary Site: Hypopharynx | Phenotype: epithelial

Top 9 Morphologies | cases: 43,879

Morphology	n	%
Squamous cell carcinoma	42,417	96.7%
Basaloid squamous cell carcinoma	849	1.9%
Adenocarcinoma	144	0.3%
Lymphoepithelial carcinoma	142	0.3%
Papillary squamous cell carcinoma	115	0.3%
Neuroendocrine carcinoma	57	0.1%
Adenoid cystic carcinoma	46	0.1%
Mucoepidermoid carcinoma	40	<0.1%
Basaloid carcinoma	24	<0.1%

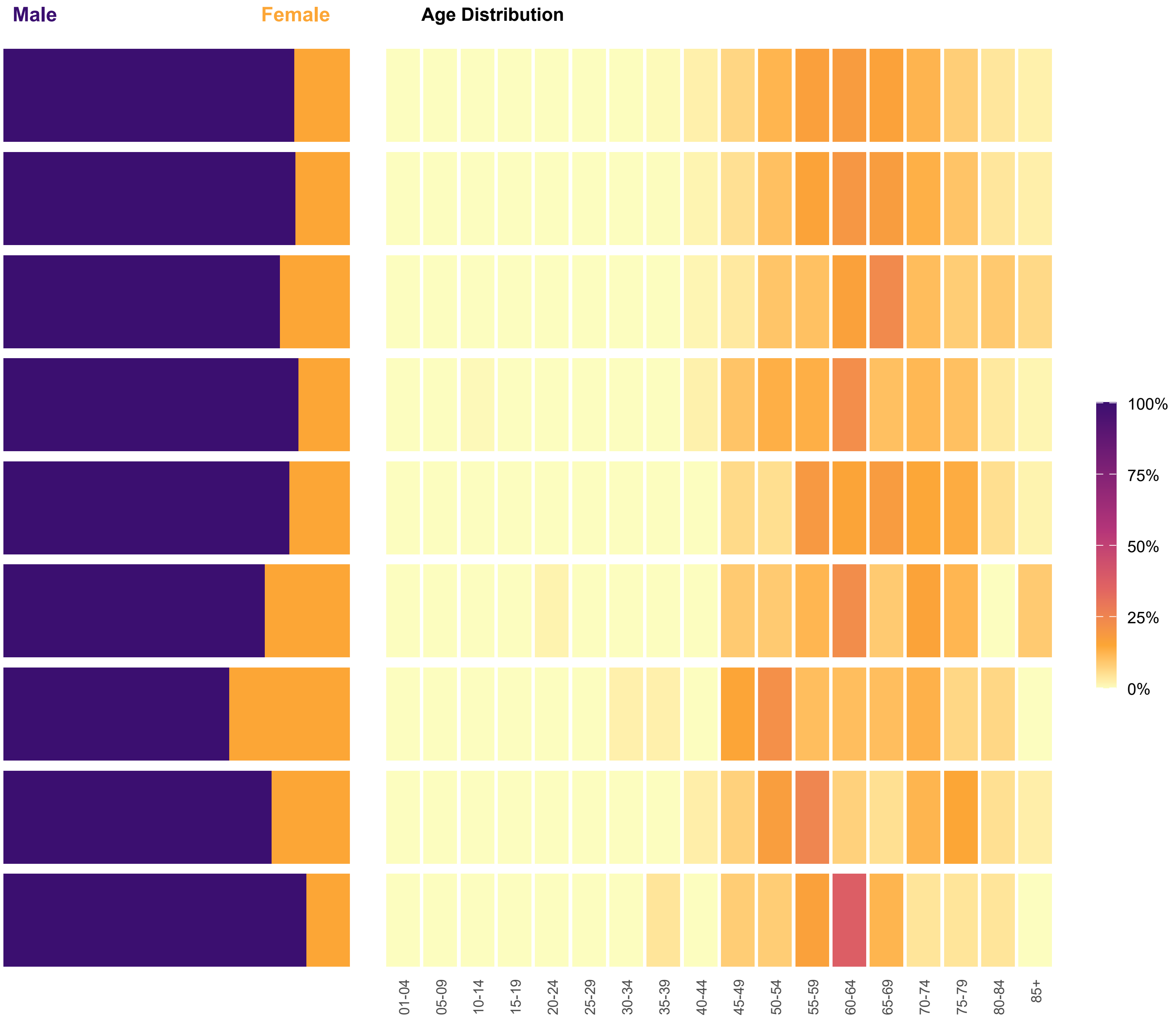

Primary Site: Hypopharynx | Phenotype: Grouped Phenotypes

Top 4 Morphologies | cases: 453

Morphology	n	%
Diffuse large B-cell lymphoma	64	14.1%
Small cell carcinoma	63	13.9%
Marginal zone B-cell lymphoma	23	5.1%
Carcinosarcoma	22	4.9%

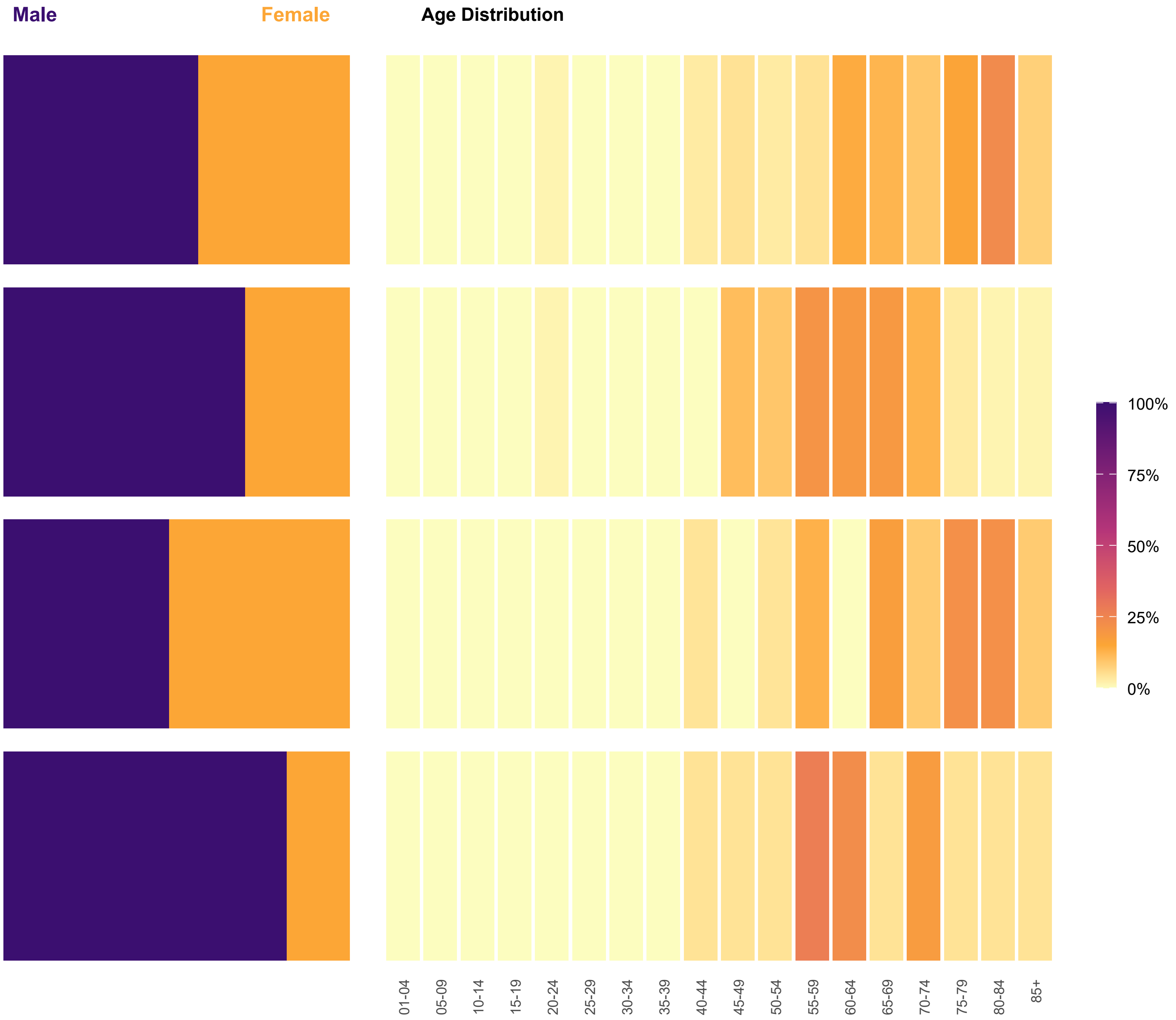

Primary Site: Intracranial Gland | Phenotype: epithelial

Top 3 Morphologies | cases: 386

Morphology	n	%
Pituitary carcinoma	276	71.5%
Adenocarcinoma	49	12.7%
Neuroendocrine carcinoma	20	5.2%

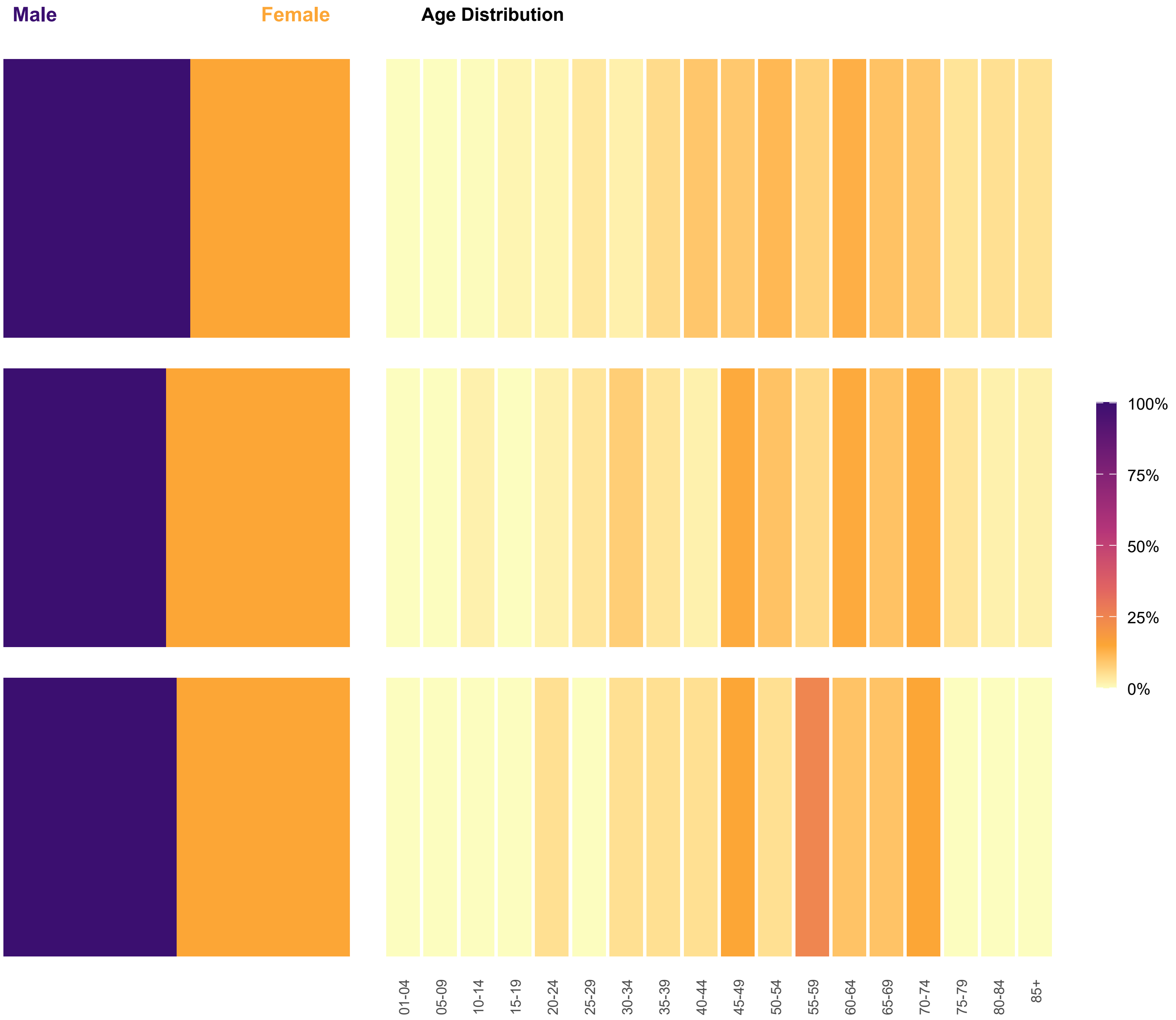

Primary Site: Intracranial Gland | Phenotype: Grouped Phenotypes

Top 13 Morphologies | cases: 2,528

Morphology	n	%
Germinoma	1,018	40.3%
Pineoblastoma	835	33.0%
Mixed germ cell tumor	130	5.1%
Chordoma	65	2.6%
Glioblastoma	40	1.6%
Papillary tumor of the pineal…	37	1.5%
Teratoma	36	1.4%
Diffuse large B-cell lymphoma	31	1.2%
CNS embryonal tumor	31	1.2%
Germ cell tumor nonseminomatous	30	1.2%
Astrocytoma	28	1.1%
Langerhans cell histiocytosis…	24	0.9%
Glioma malignant	21	0.8%

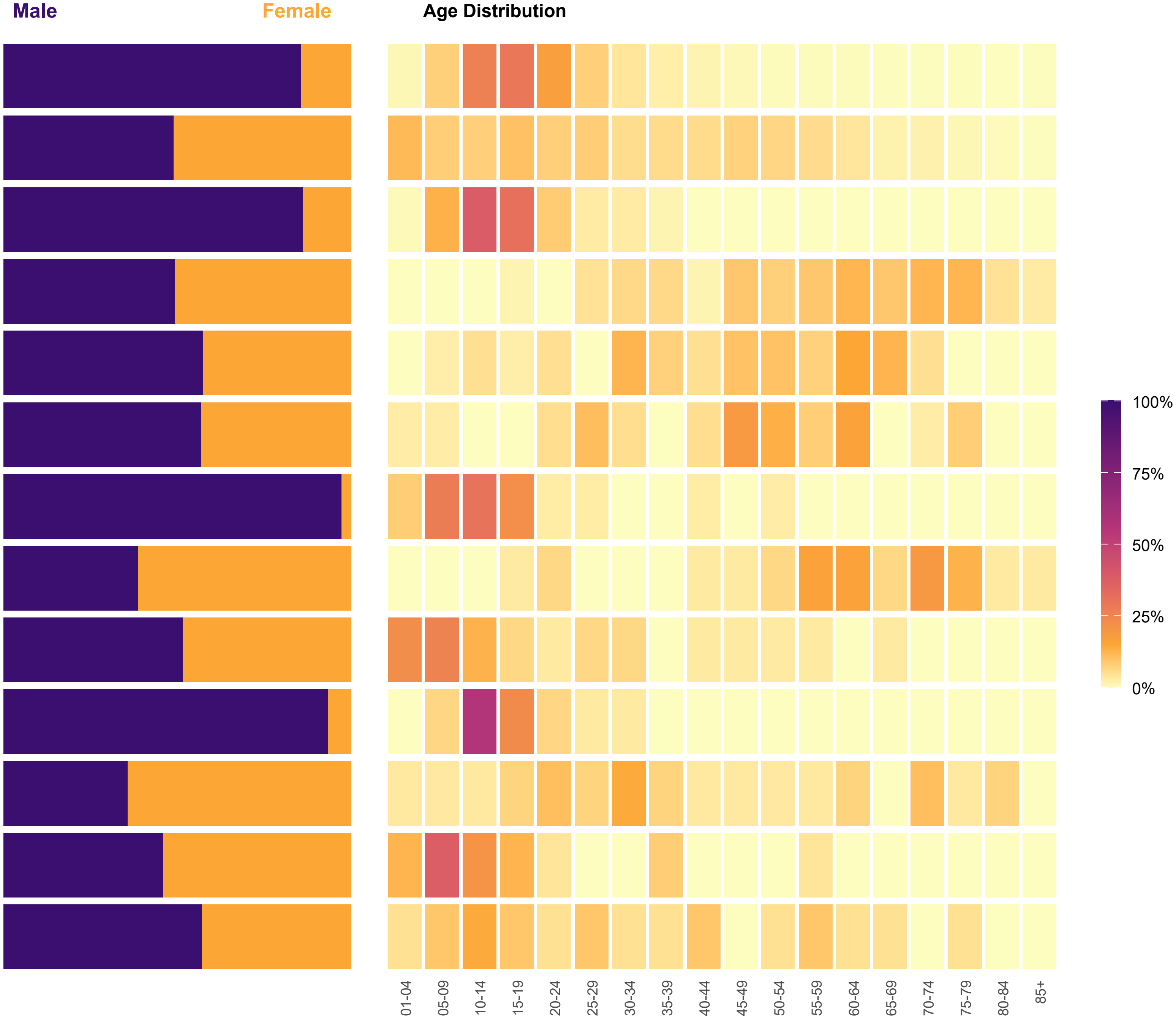

Primary Site: Kidney and Renal Pelvis | Phenotype: epithelial

Top 24 Morphologies | cases: 658,513

Morphology	n	%
Clear cell adenocarcinoma	284,923	43.3%
Renal cell carcinoma	218,031	33.1%
Papillary adenocarcinoma	60,543	9.2%
Transitional cell carcinoma	26,919	4.1%
Renal cell carcinoma chromophobe…	26,882	4.1%
Papillary transitional cell…	18,582	2.8%
Adenocarcinoma with mixed subtypes	6,019	0.9%
Renal cell carcinoma sarcomatoid	5,622	0.9%
Cyst-associated renal cell…	2,523	0.4%
Adenocarcinoma	2,210	0.3%
Collecting duct carcinoma	1,384	0.2%
Mixed cell adenocarcinoma	1,273	0.2%
Squamous cell carcinoma	1,130	0.2%
Oxyphilic adenocarcinoma	576	<0.1%
Chromophobe carcinoma	334	<0.1%
Urothelial carcinoma sarcomatoid	312	<0.1%
Mucinous adenocarcinoma	274	<0.1%
Neuroendocrine carcinoma	216	<0.1%
HRCC-associated renal cell…	134	<0.1%
Tubular adenocarcinoma	131	<0.1%
Adenocarcinoma in tubulovillous…	126	<0.1%
Urothelial carcinoma micropapillary	84	<0.1%
Acinar cell carcinoma	31	<0.1%
Trabecular adenocarcinoma	30	<0.1%

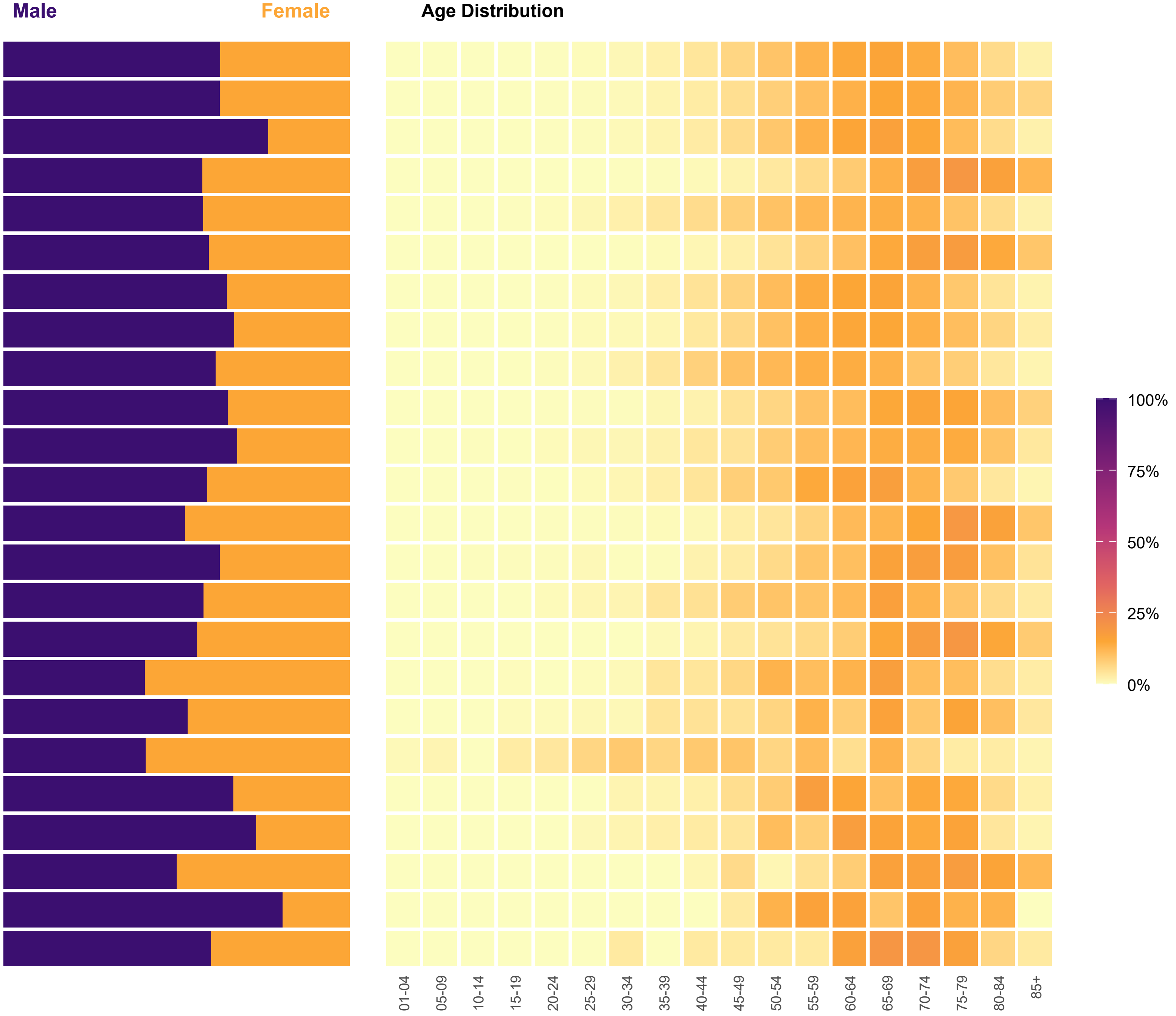

Primary Site: Kidney and Renal Pelvis | Phenotype: Grouped Phenotypes

Top 23 Morphologies | cases: 8,610

Morphology	n	%
Nephroblastoma	5,228	60.7%
Diffuse large B-cell lymphoma	887	10.3%
Clear cell sarcoma of kidney	285	3.3%
Marginal zone B-cell lymphoma	281	3.3%
Small cell carcinoma	280	3.3%
lymphoma non-Hodgkin	215	2.5%
Rhabdoid tumor	193	2.2%
Malignant cystic nephroma	178	2.1%
Neuroendocrine tumor	152	1.8%
Large cell carcinoma	107	1.2%
Follicular lymphoma grade 1/2	93	1.1%
Carcinosarcoma	85	1.0%
Follicular lymphoma	72	0.8%
Neuroblastoma	58	0.7%
lymphoma	58	0.7%
CNS embryonal tumor	55	0.6%
Burkitt lymphoma	39	0.5%
B-cell chronic lymphocytic…	37	0.4%
Lymphoplasmacytic lymphoma	35	0.4%
Large cell neuroendocrine carcinoma	33	0.4%
Plasmacytoma extramedullary	32	0.4%
Follicular lymphoma grade 3	24	0.3%
Neuroendocrine tumor grade 2	20	0.2%

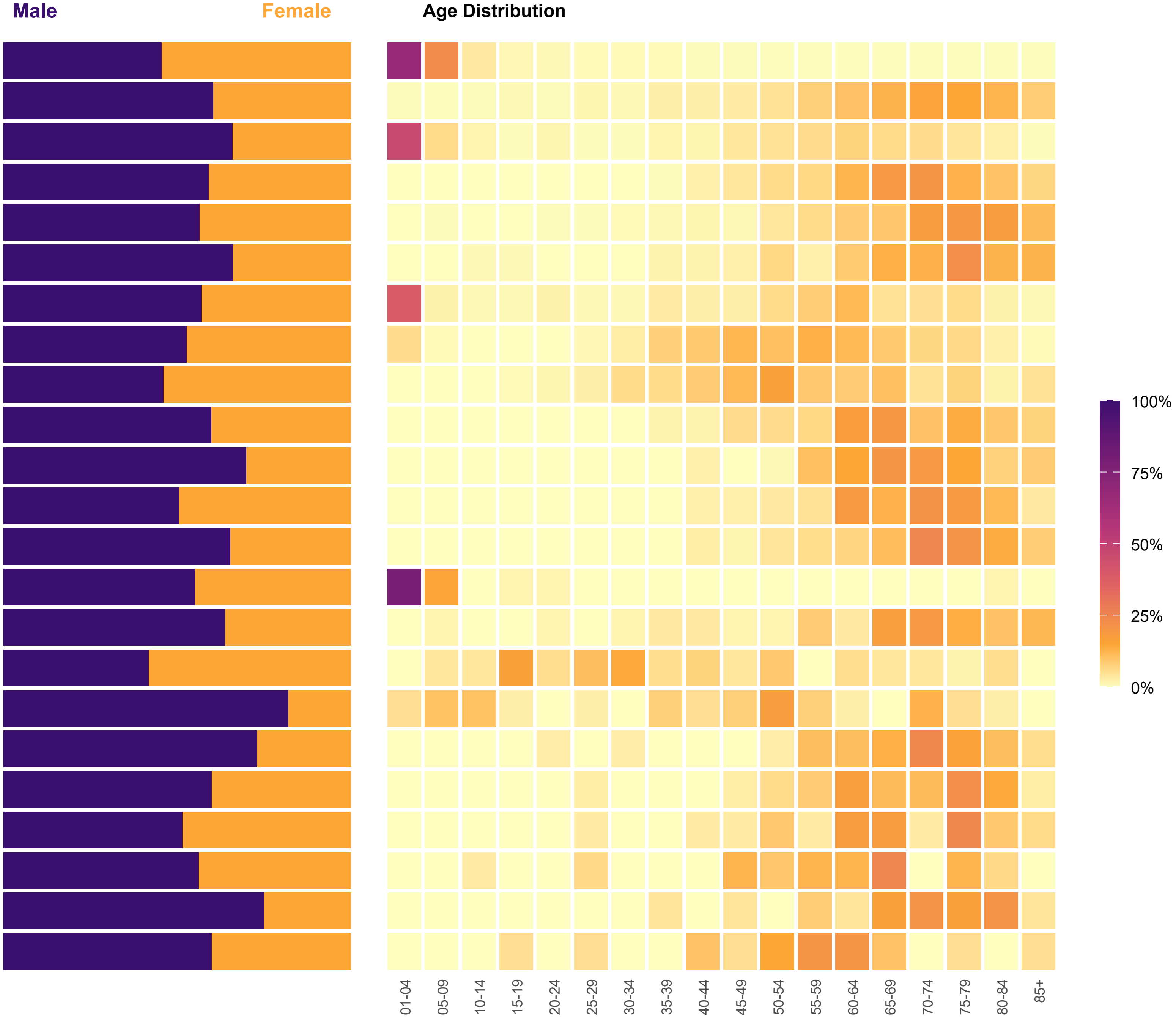

Primary Site: Larynx | Phenotype: epithelial

Top 9 Morphologies | cases: 144,236

Morphology	n	%
Squamous cell carcinoma	141,187	97.9%
Basaloid squamous cell carcinoma	1,163	0.8%
Papillary squamous cell carcinoma	810	0.6%
Adenocarcinoma	314	0.2%
Neuroendocrine carcinoma	237	0.2%
Adenoid cystic carcinoma	201	0.1%
Lymphoepithelial carcinoma	103	<0.1%
Mucoepidermoid carcinoma	74	<0.1%
Basaloid carcinoma	37	<0.1%

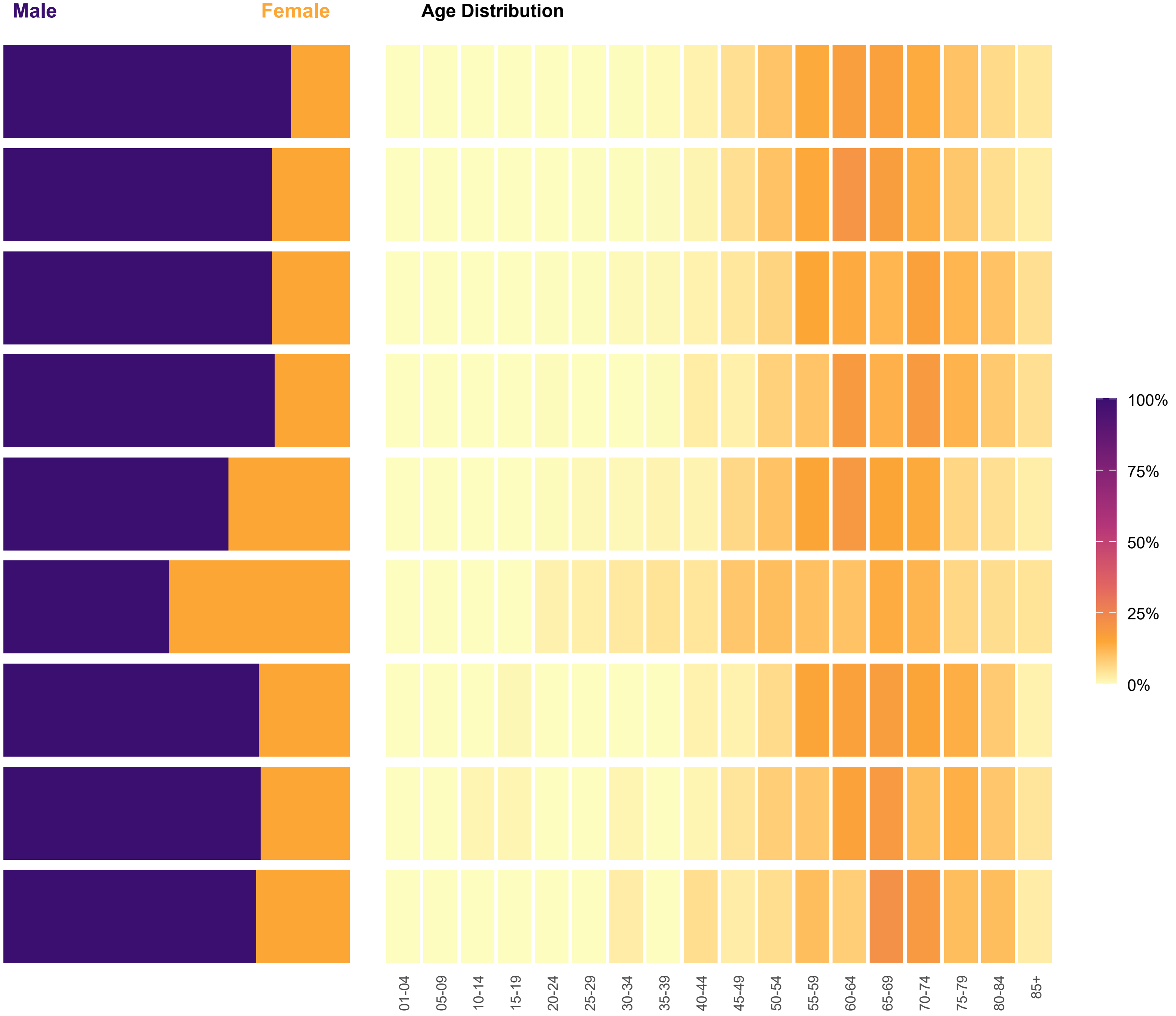

Primary Site: Larynx | Phenotype: Grouped Phenotypes

Top 14 Morphologies | cases: 1,838

Morphology	n	%
Chondrosarcoma	427	23.2%
Small cell carcinoma	368	20.0%
Diffuse large B-cell lymphoma	104	5.7%
Marginal zone B-cell lymphoma	92	5.0%
Sarcoma	81	4.4%
Carcinosarcoma	78	4.2%
Plasmacytoma extramedullary	58	3.2%
Neuroendocrine tumor grade 2	49	2.7%
Leiomyosarcoma	44	2.4%
Large cell neuroendocrine carcinoma	43	2.3%
Spindle cell sarcoma	29	1.6%
lymphoma non-Hodgkin	29	1.6%
Neuroendocrine tumor	28	1.5%
Malignant fibrous histiocytoma	25	1.4%

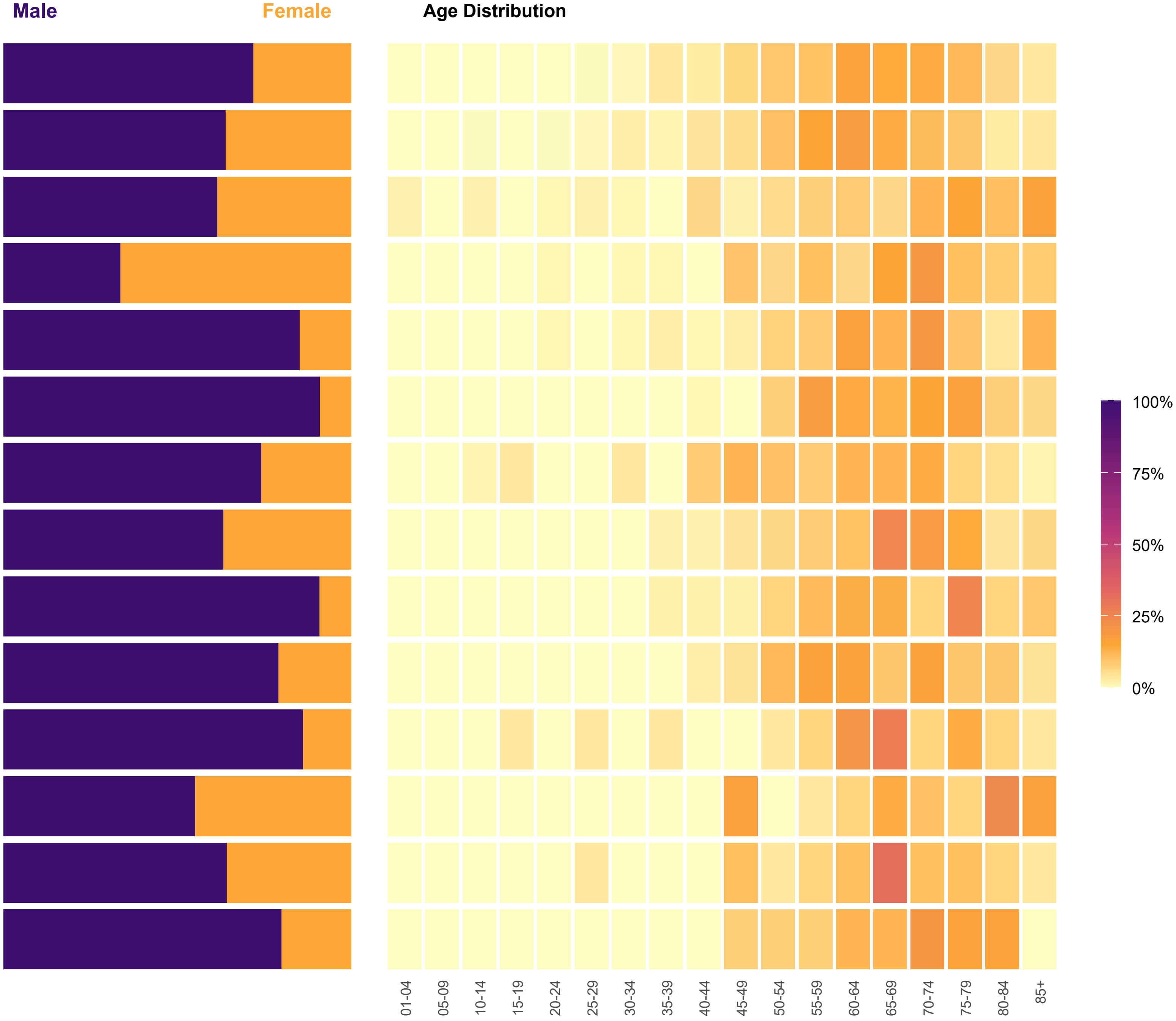

Primary Site: Lesions of anus and anal canal | Phenotype: epithelial

Top 21 Morphologies | cases: 72,674

Morphology	n	%
Squamous cell carcinoma	57,931	79.7%
Adenocarcinoma	4,961	6.8%
Basaloid squamous cell carcinoma	3,700	5.1%
Cloacogenic carcinoma	2,215	3.0%
Basaloid carcinoma	1,009	1.4%
Mucinous adenocarcinoma	770	1.1%
Adenocarcinoma in tubulovillous…	377	0.5%
Adenocarcinoma in adenomatous polyp	301	0.4%
Neuroendocrine carcinoma	265	0.4%
Paget disease extramammary	194	0.3%
Mucin-producing adenocarcinoma	162	0.2%
Adenocarcinoma in villous adenoma	148	0.2%
Signet ring cell carcinoma	131	0.2%
Basosquamous carcinoma	77	0.1%
Adenocarcinoma of anal glands	67	<0.1%
Papillary squamous cell carcinoma	61	<0.1%
Adenocarcinoma with mixed subtypes	60	<0.1%
Basal cell carcinoma	47	<0.1%
Tubular adenocarcinoma	38	<0.1%
Transitional cell carcinoma	32	<0.1%
Adenocarcinoma with neuroendocrine…	25	<0.1%

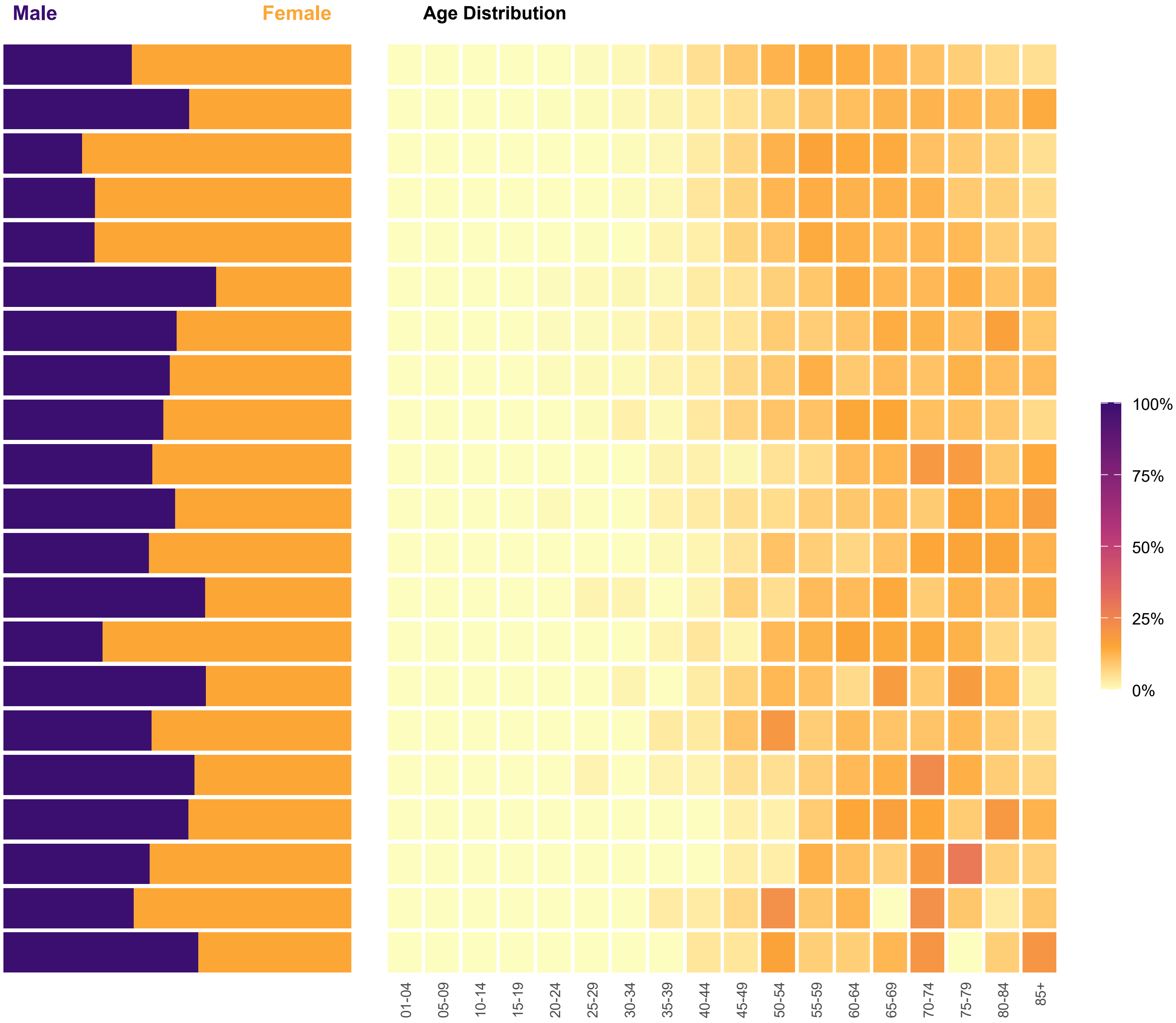

Primary Site: Lesions of anus and anal canal | Phenotype: Grouped Phenotypes

Top 13 Morphologies | cases: 2,092

Morphology	n	%
Malignant melanoma	994	47.5%
Small cell carcinoma	244	11.7%
Neuroendocrine tumor	162	7.7%
Nodular melanoma	139	6.6%
Diffuse large B-cell lymphoma	55	2.6%
Amelanotic melanoma	53	2.5%
Kaposi sarcoma	47	2.2%
Gastrointestinal stromal tumor	42	2.0%
Large cell neuroendocrine carcinoma	36	1.7%
Spindle cell melanoma	31	1.5%
Leiomyosarcoma	24	1.1%
Mucosal lentiginous melanoma	23	1.1%
Plasmablastic lymphoma	21	1.0%

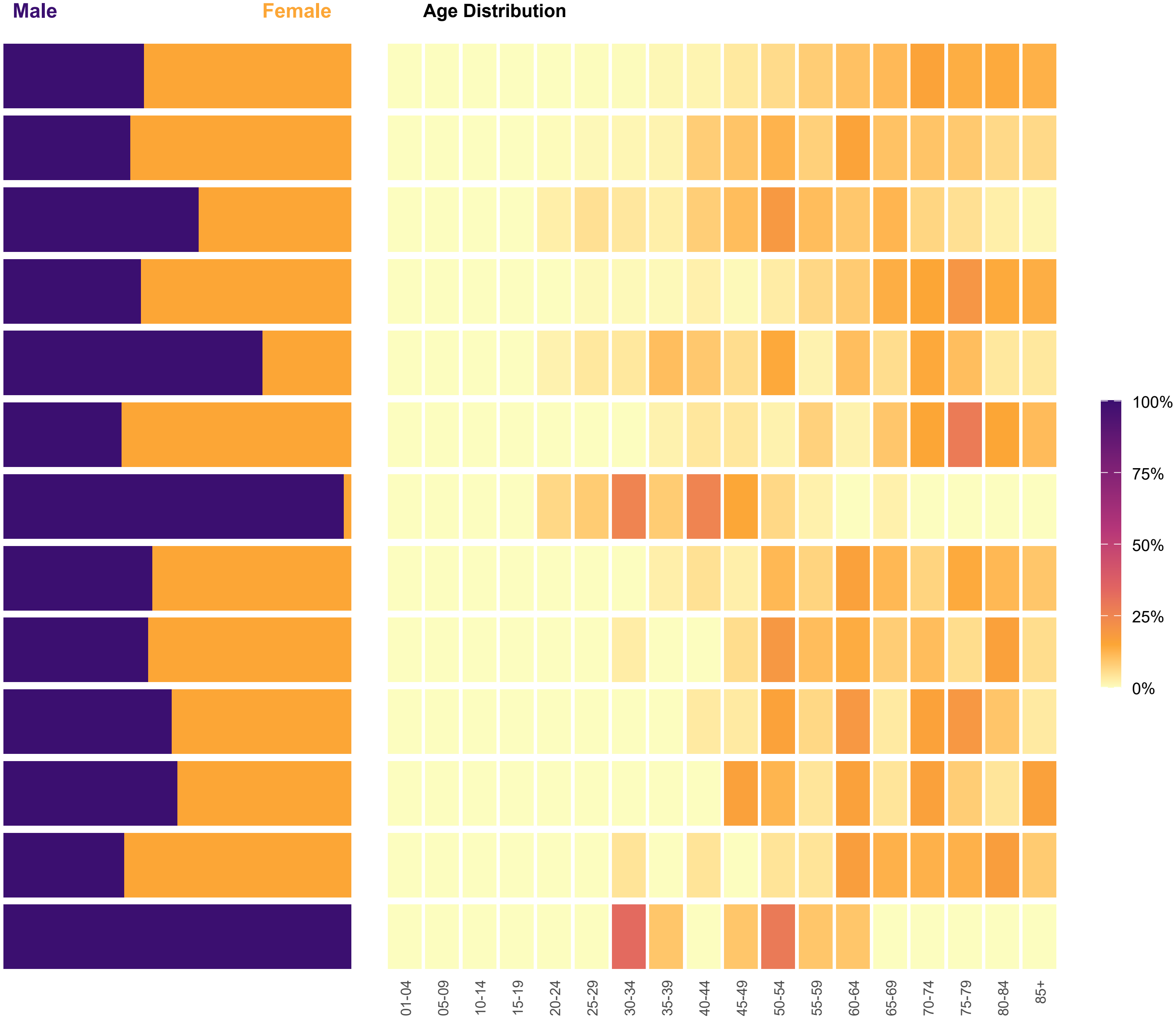

Primary Site: Lip | Phenotype: epithelial

Top 14 Morphologies | cases: 24,837

Morphology	n	%
Squamous cell carcinoma	23,034	92.7%
Basal cell carcinoma	748	3.0%
Mucoepidermoid carcinoma	265	1.1%
Adenoid cystic carcinoma	165	0.7%
Adenocarcinoma	152	0.6%
Basosquamous carcinoma	87	0.4%
Polymorphous adenocarcinoma	69	0.3%
Acinar cell carcinoma	55	0.2%
Merkel cell carcinoma	42	0.2%
Basaloid squamous cell carcinoma	40	0.2%
Basal cell adenocarcinoma	29	0.1%
Microcystic adnexal carcinoma	22	<0.1%
Secretory carcinoma	22	<0.1%
Papillary squamous cell carcinoma	20	<0.1%

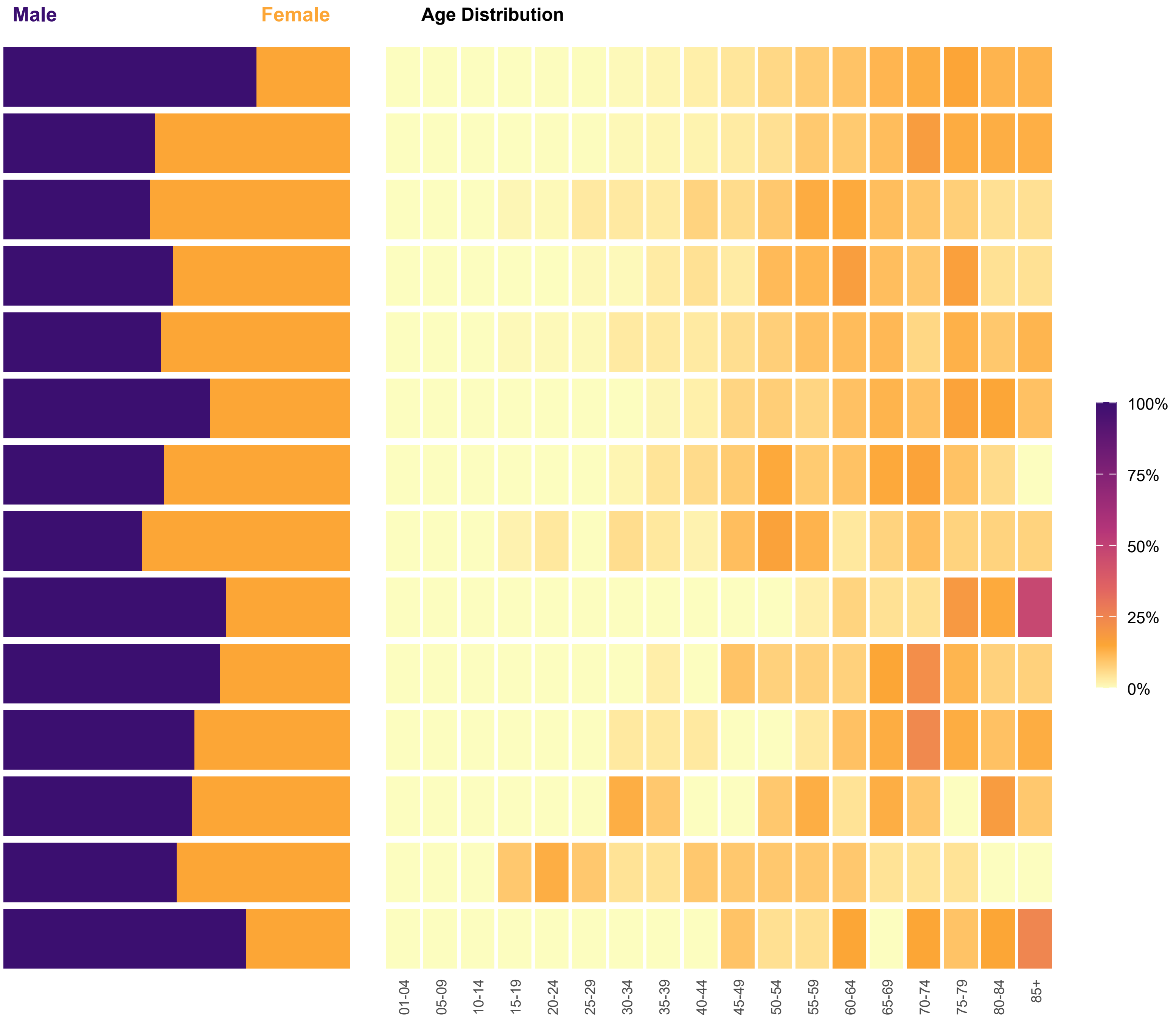

Primary Site: Lip | Phenotype: Grouped Phenotypes

Top 2 Morphologies | cases: 299

Morphology	n	%
Malignant melanoma	75	25.1%
Marginal zone B-cell lymphoma	51	17.1%

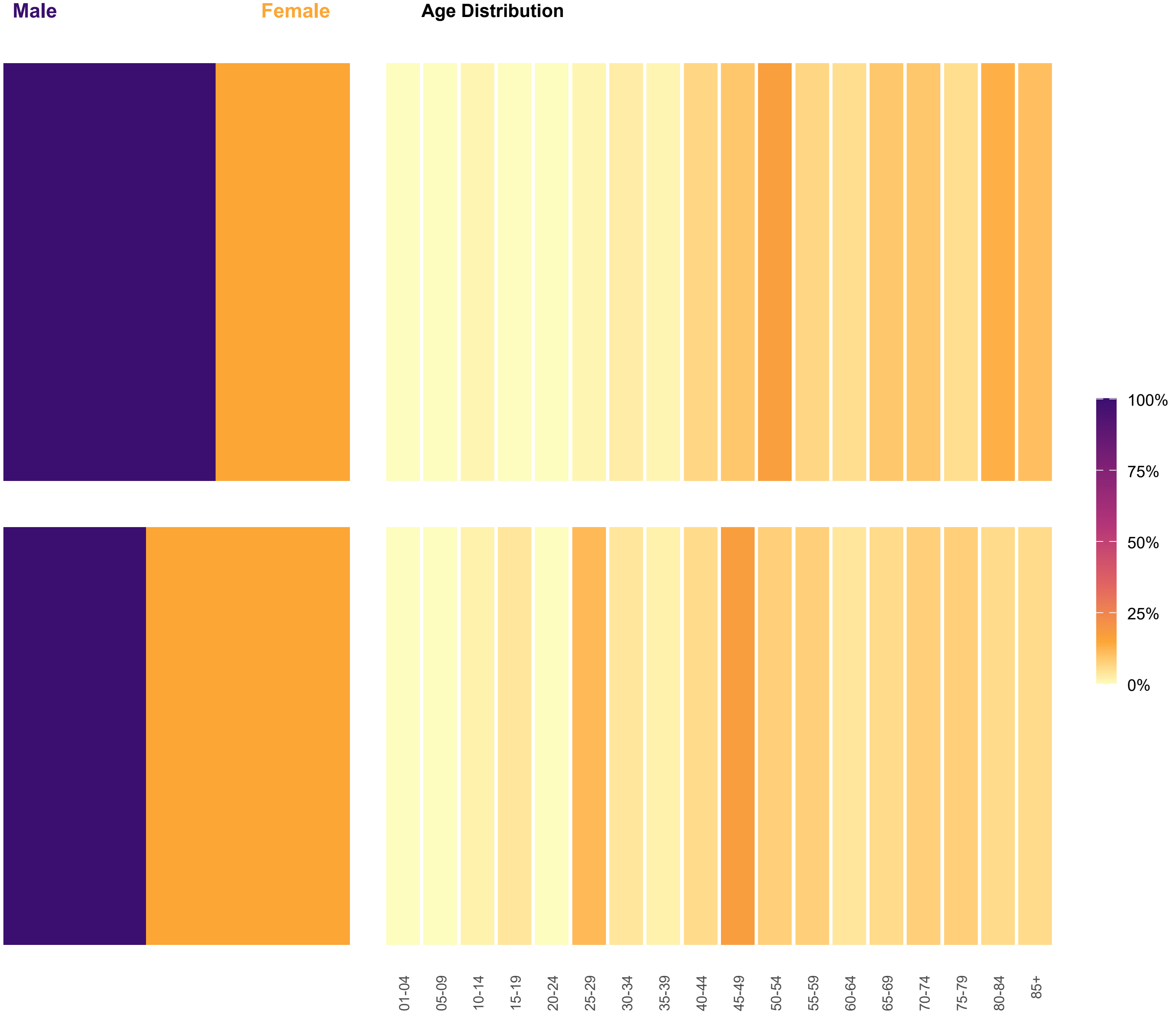

Primary Site: Liver and Intrahepatic Bile Ducts | Phenotype: epithelial

Top 25 Morphologies | cases: 346,644

Morphology	n	%
Hepatocellular carcinoma	269,342	77.7%
Cholangiocarcinoma	52,770	15.2%
Adenocarcinoma	13,986	4.0%
Combined hepatocellular carcinoma…	3,456	1.0%
Hepatocellular carcinoma clear…	1,283	0.4%
Klatskin tumor	1,063	0.3%
Neuroendocrine carcinoma	985	0.3%
Hepatocellular carcinoma…	869	0.3%
Trabecular adenocarcinoma	627	0.2%
Hepatocellular carcinoma scirrhous	259	<0.1%
Infiltrating duct carcinoma	256	<0.1%
Mucin-producing adenocarcinoma	234	<0.1%
Mucinous adenocarcinoma	223	<0.1%
Hepatocellular carcinoma spindle…	215	<0.1%
Tubular adenocarcinoma	207	<0.1%
Squamous cell carcinoma	115	<0.1%
Clear cell adenocarcinoma	99	<0.1%
Hepatocellular carcinoma…	93	<0.1%
Bile duct cystadenocarcinoma	71	<0.1%
Signet ring cell carcinoma	50	<0.1%
Papillary adenocarcinoma	46	<0.1%
Pancreatobiliary type carcinoma	44	<0.1%
Hepatoid adenocarcinoma	44	<0.1%
Adenocarcinoma with mixed subtypes	36	<0.1%
Cystadenocarcinoma	36	<0.1%

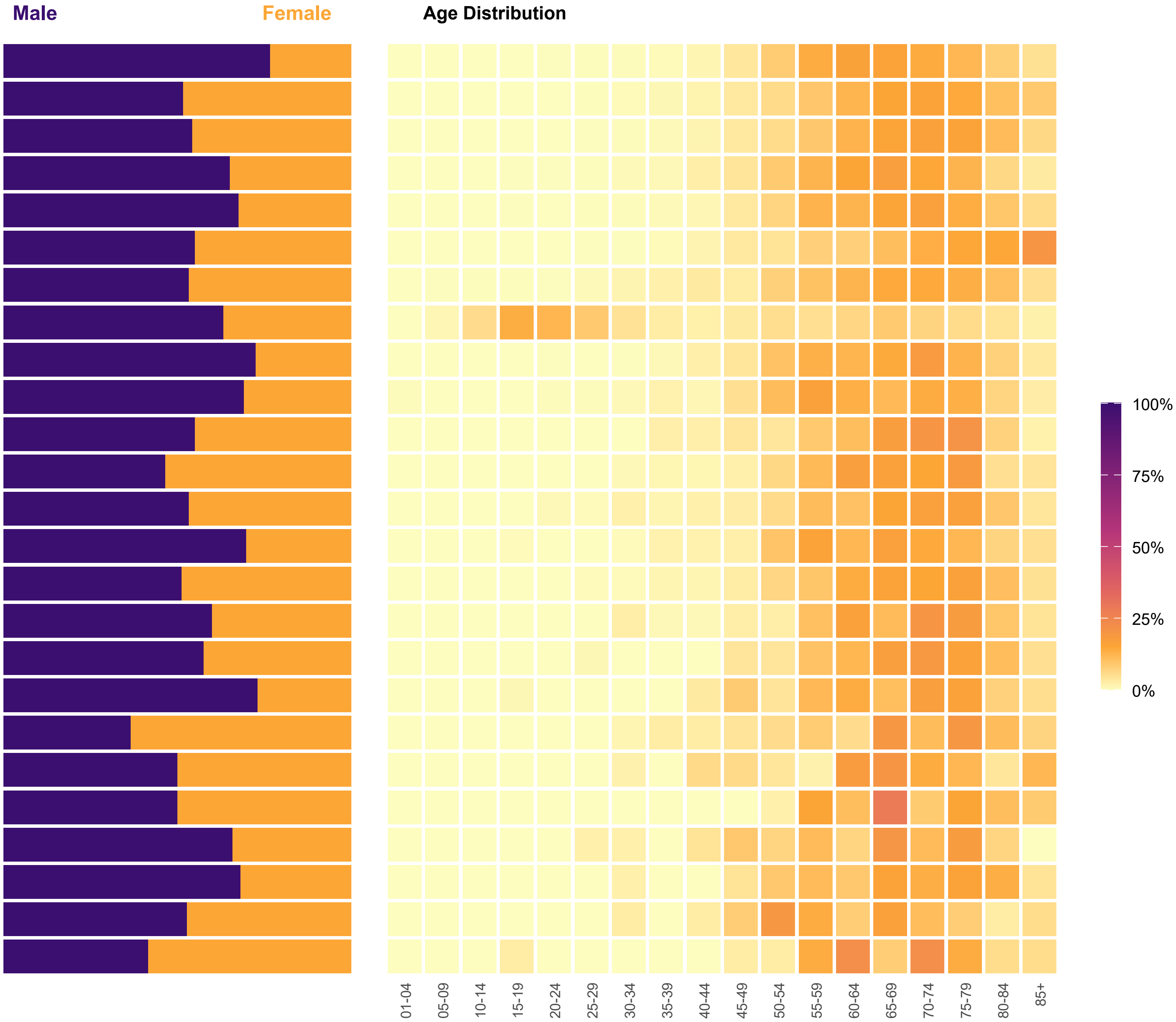

Primary Site: Liver and Intrahepatic Bile Ducts | Phenotype: Grouped Phenotypes

Top 18 Morphologies | cases: 5,190

Morphology	n	%
Diffuse large B-cell lymphoma	1,693	32.6%
Hepatoblastoma	1,315	25.3%
Neuroendocrine tumor	542	10.4%
lymphoma non-Hodgkin	262	5.0%
Small cell carcinoma	173	3.3%
Marginal zone B-cell lymphoma	148	2.9%
lymphoma	111	2.1%
Neuroendocrine tumor grade 2	87	1.7%
Carcinosarcoma	80	1.5%
Burkitt lymphoma	80	1.5%
Mature T-cell lymphoma	80	1.5%
Large cell neuroendocrine carcinoma	73	1.4%
Large cell carcinoma	72	1.4%
Hodgkin lymphoma	67	1.3%
Follicular lymphoma	41	0.8%
Follicular lymphoma grade 1/2	34	0.7%
Anaplastic large cell lymphoma…	27	0.5%
Follicular lymphoma grade 3	23	0.4%

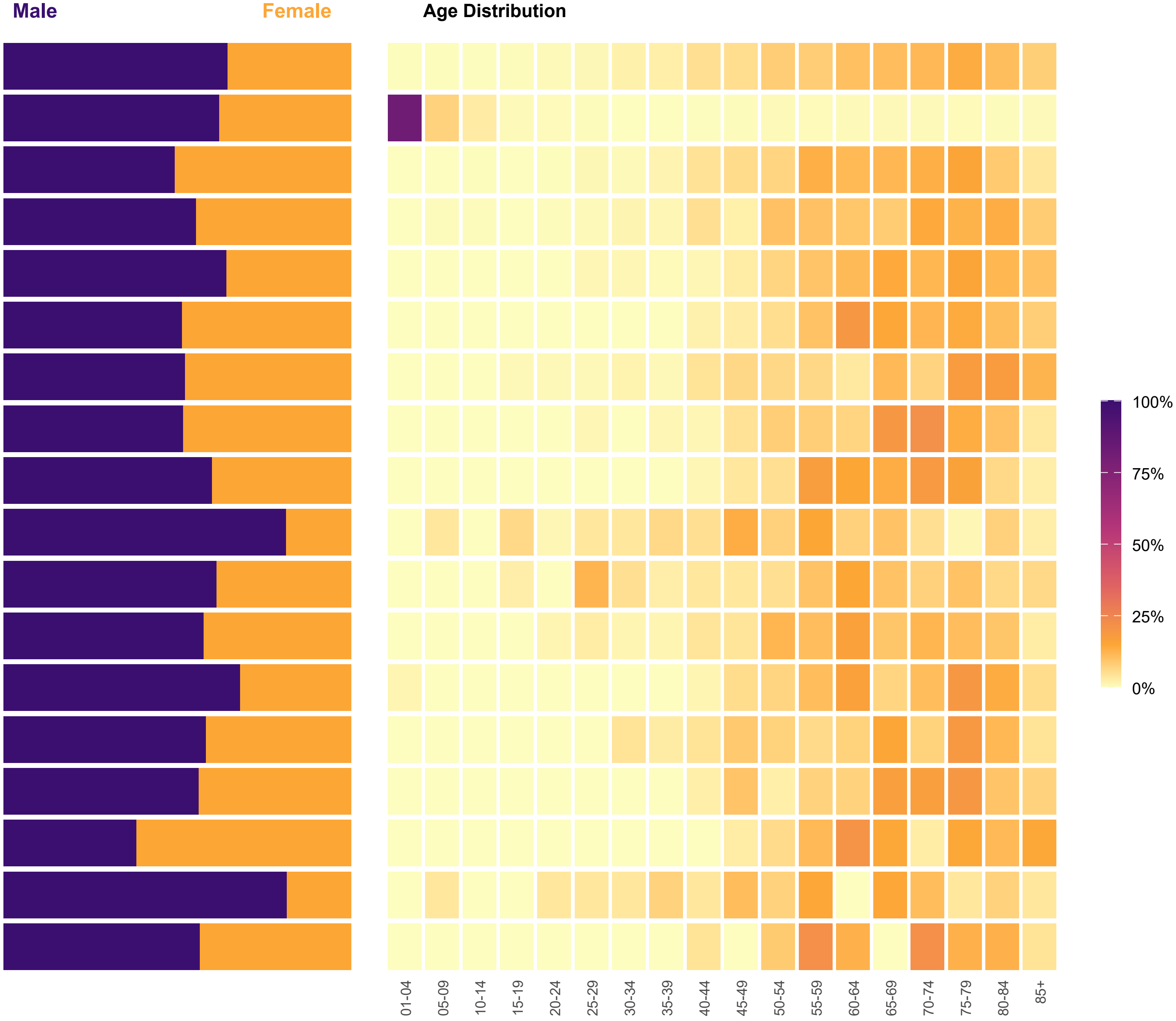

Primary Site: Long bones of lower limb and associated joints | Phenotype: epithelial

Top 1 Morphologies | cases: 174

Morphology	n	%
Adamantinoma of long bones	151	86.8%

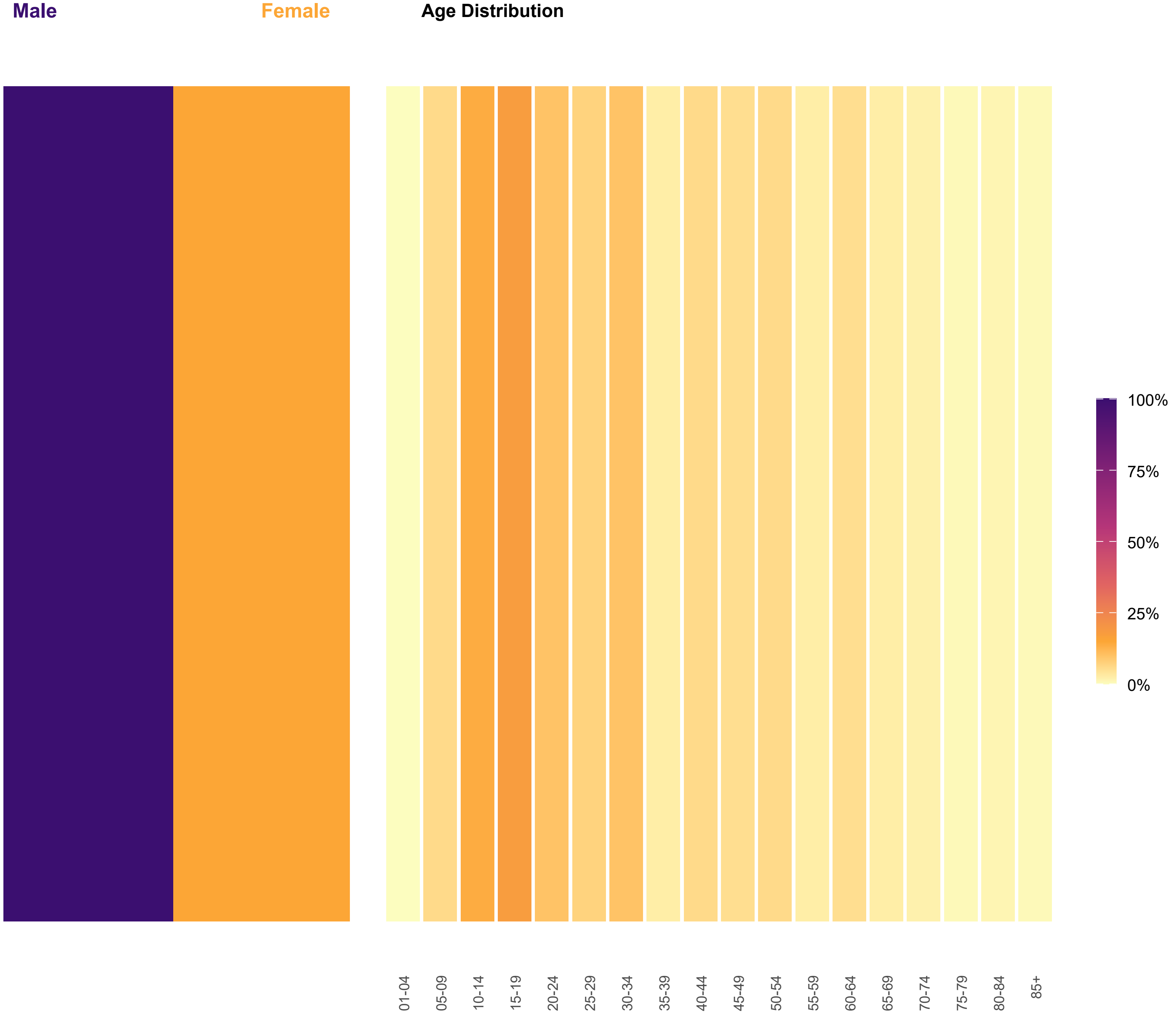

Primary Site: Long bones of lower limb and associated joints | Phenotype: Grouped Phenotypes

Top 7 Morphologies | cases: 1,747

Morphology	n	%
Diffuse large B-cell lymphoma	848	48.5%
Plasmacytoma	359	20.5%
Langerhans cell histiocytosis…	174	10.0%
lymphoma non-Hodgkin	141	8.1%
Follicular lymphoma	27	1.5%
lymphoma	25	1.4%
Marginal zone B-cell lymphoma	21	1.2%

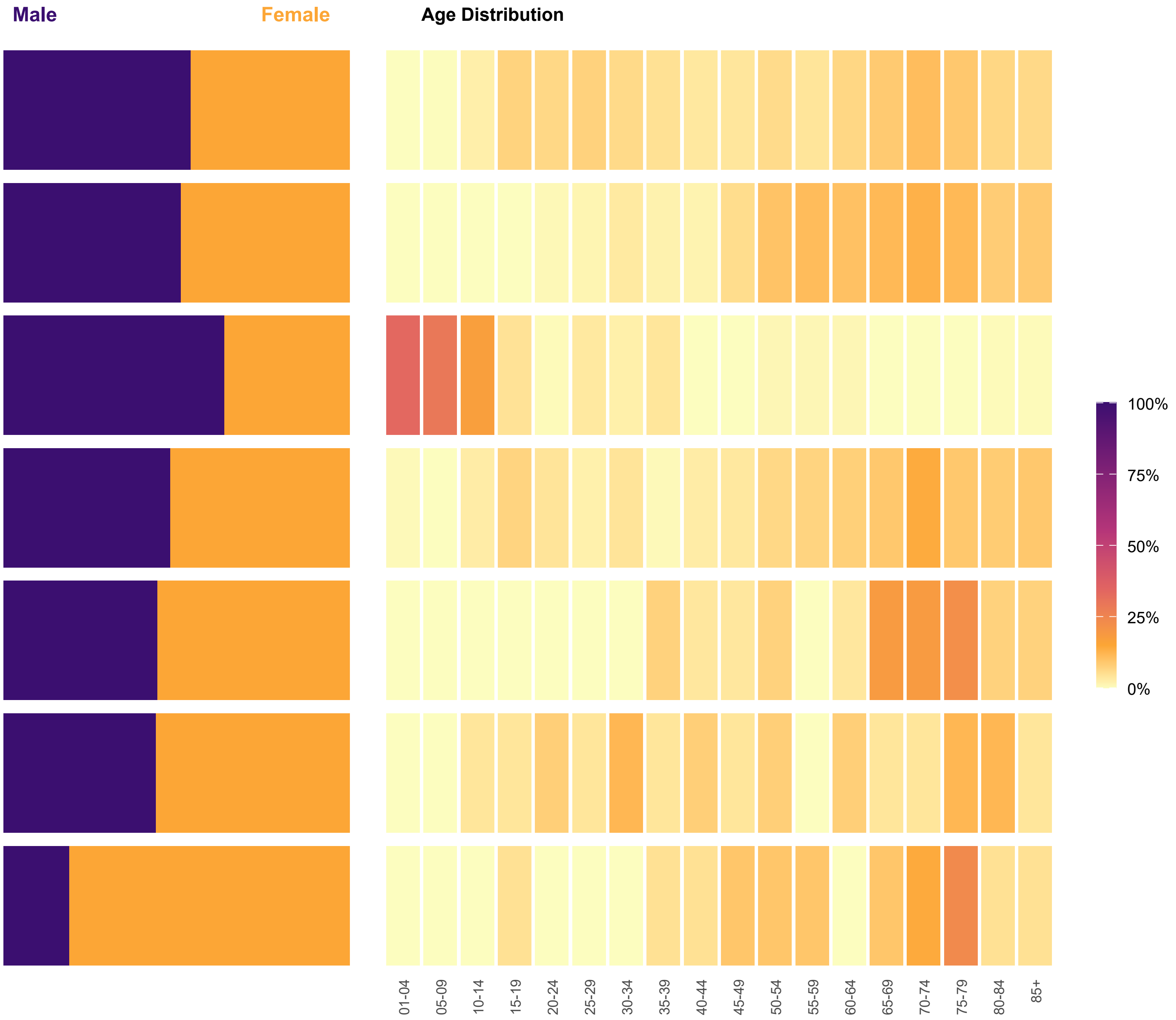

Primary Site: Long bones of upper limb and scapula and associated joints | Phenotype: Grouped

Top 20 Morphologies | cases: 4,422

Morphology	n	%
Chondrosarcoma	1,402	31.7%
Osteosarcoma	948	21.4%
Plasmacytoma	434	9.8%
Diffuse large B-cell lymphoma	358	8.1%
Dedifferentiated chondrosarcoma	143	3.2%
Chondroblastic osteosarcoma	111	2.5%
Langerhans cell histiocytosis…	102	2.3%
Giant cell tumor of bone malignant	91	2.1%
Sarcoma	74	1.7%
Parosteal osteosarcoma	64	1.4%
Myxoid chondrosarcoma	56	1.3%
Telangiectatic osteosarcoma	54	1.2%
Fibroblastic osteosarcoma	48	1.1%
Malignant fibrous histiocytoma	43	1.0%
Central osteosarcoma	33	0.7%
lymphoma non-Hodgkin	32	0.7%
Giant cell sarcoma	30	0.7%
Ewing sarcoma	27	0.6%
Spindle cell sarcoma	25	0.6%
Clear cell chondrosarcoma	20	0.5%

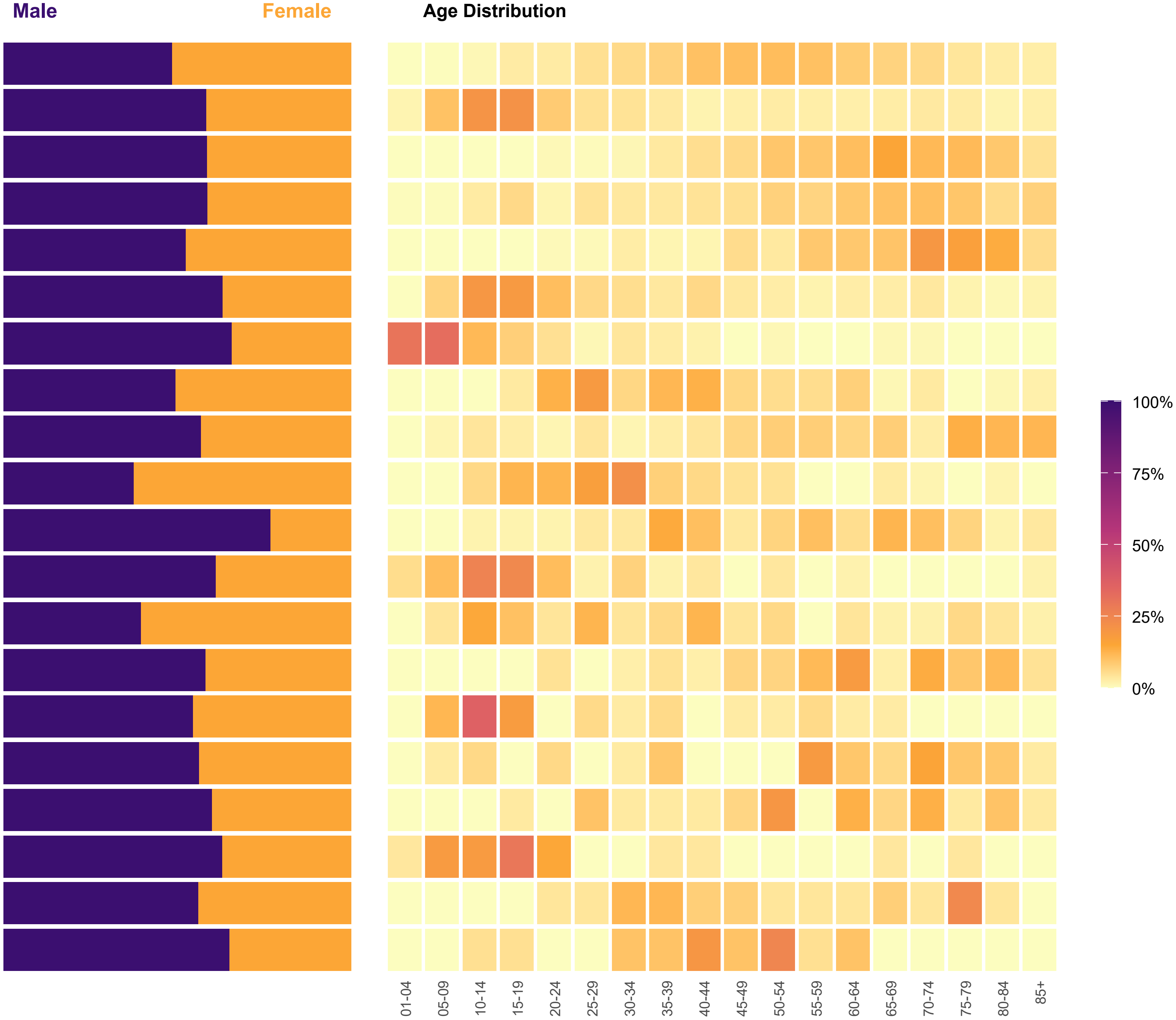

Primary Site: Lung and Bronchus | Phenotype: epithelial

Top 25 Morphologies | cases: 1,423,333

Morphology	n	%
Adenocarcinoma	743,105	52.2%
Squamous cell carcinoma	497,965	35.0%
Lepidic adenocarcinoma	37,808	2.7%
Neuroendocrine carcinoma	26,687	1.9%
Adenocarcinoma with mixed subtypes	23,047	1.6%
Acinar cell carcinoma	16,543	1.2%
Mucinous adenocarcinoma	15,989	1.1%
Papillary adenocarcinoma	14,459	1.0%
Acinar cell cystadenocarcinoma	11,749	0.8%
Mucin-producing adenocarcinoma	8,321	0.6%
Adenocarcinoma of the lung mucinous	5,665	0.4%
Basaloid squamous cell carcinoma	3,443	0.2%
Bronchiolo-alveolar carcinoma…	2,901	0.2%
Clear cell adenocarcinoma	2,177	0.2%
Signet ring cell carcinoma	2,169	0.2%
Adenocarcinoma with neuroendocrine…	1,617	0.1%
Mucoepidermoid carcinoma	1,283	<0.1%
Mixed cell adenocarcinoma	1,091	<0.1%
Micropapillary carcinoma	924	<0.1%
Adenocarcinoma of the lung mixed…	838	<0.1%
Adenoid cystic carcinoma	795	<0.1%
Tubular adenocarcinoma	773	<0.1%
Alveolar adenocarcinoma	581	<0.1%
Basaloid carcinoma	454	<0.1%
Adenocarcinoma in tubulovillous…	444	<0.1%

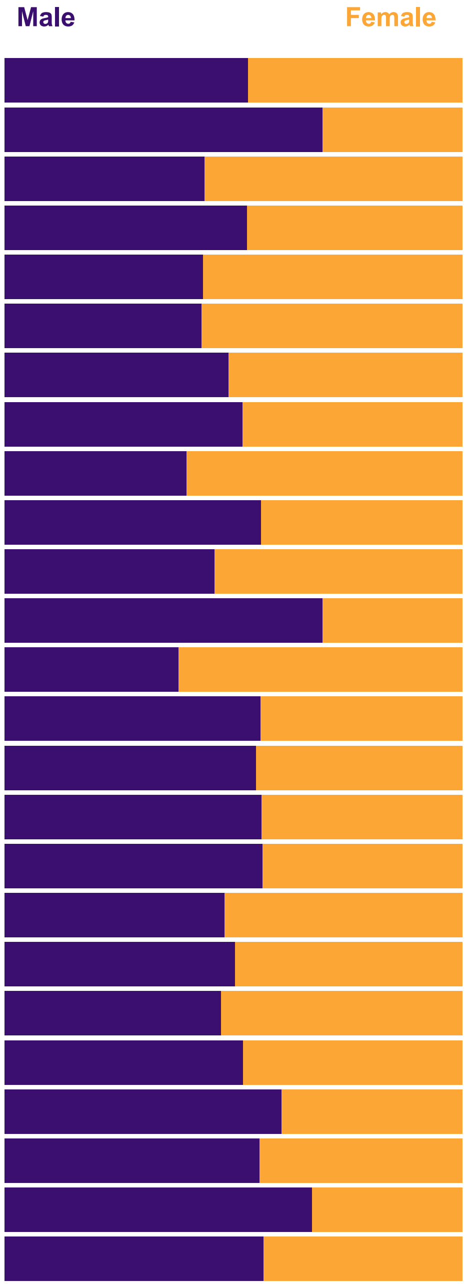

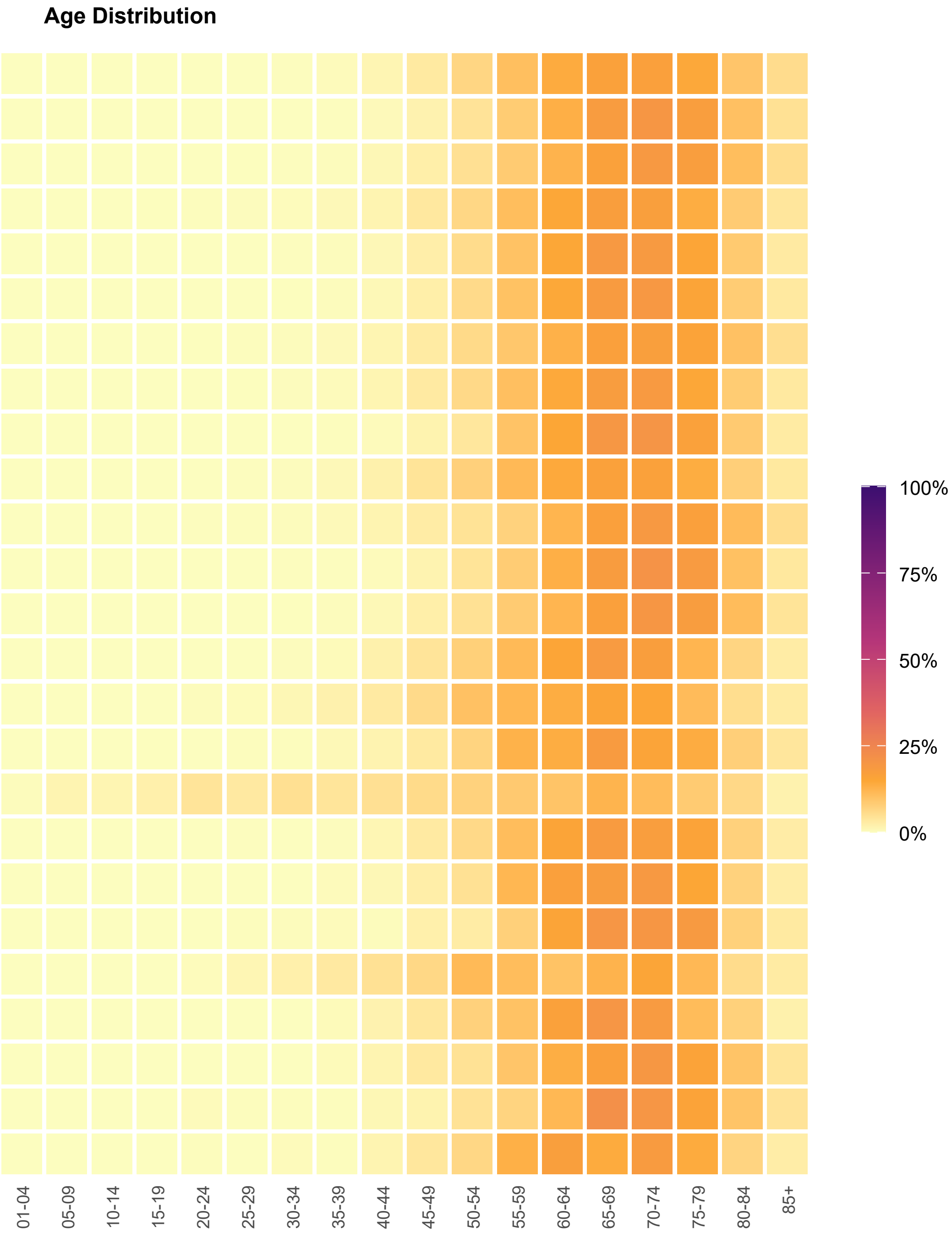

Primary Site: Lung and Bronchus | Phenotype: Grouped Phenotypes

Top 21 Morphologies | cases: 406,830

Morphology	n	%
Small cell carcinoma	284,419	69.9%
Large cell carcinoma	35,601	8.8%
Neuroendocrine tumor	28,813	7.1%
Oat cell carcinoma	28,194	6.9%
Large cell neuroendocrine carcinoma	14,962	3.7%
Combined small cell carcinoma	7,886	1.9%
Neuroendocrine tumor grade 2	4,562	1.1%
Carcinosarcoma	1,146	0.3%
Mixed adenoneuroendocrine carcinoma	291	<0.1%
Pulmonary blastoma	171	<0.1%
Malignant melanoma	125	<0.1%
Pleuropulmonary blastoma	96	<0.1%
Large cell carcinoma with rhabdoid…	95	<0.1%
Epithelial-myoepithelial carcinoma	52	<0.1%
Adenocarcinoid tumor	49	<0.1%
Enterochromaffin cell carcinoid	44	<0.1%
Malignant peripheral nerve sheath…	43	<0.1%
CNS embryonal tumor	30	<0.1%
Myoepithelial carcinoma	27	<0.1%
Rhabdoid tumor	23	<0.1%
Choriocarcinoma	22	<0.1%

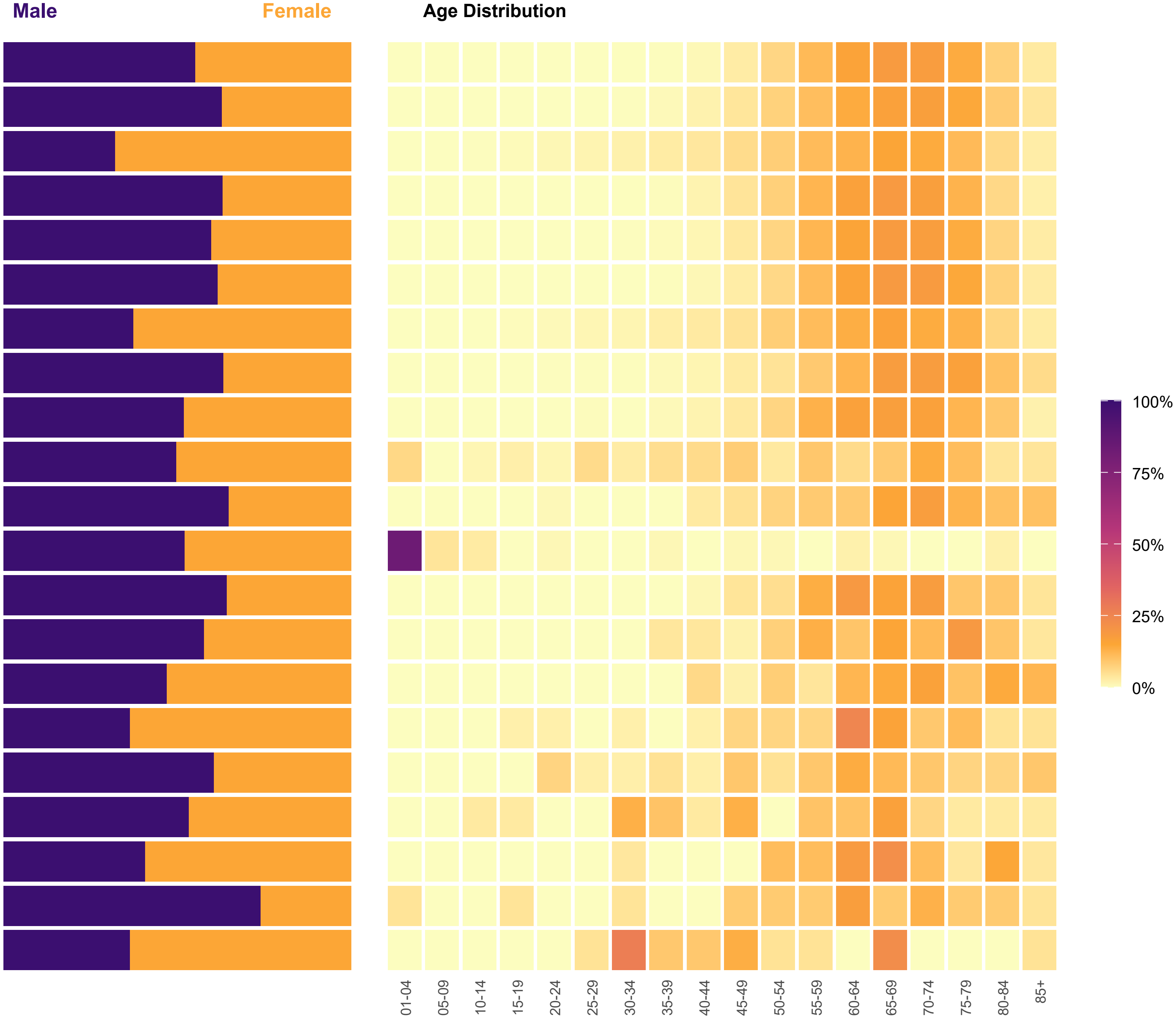

Primary Site: Major Salivary Glands | Phenotype: epithelial

Top 25 Morphologies | cases: 41,319

Morphology	n	%
Squamous cell carcinoma	10,417	25.2%
Mucoepidermoid carcinoma	9,061	21.9%
Adenoid cystic carcinoma	5,135	12.4%
Acinar cell carcinoma	4,934	11.9%
Adenocarcinoma	4,860	11.8%
Infiltrating duct carcinoma	1,645	4.0%
Carcinoma ex pleomorphic adenoma	1,575	3.8%
Basal cell adenocarcinoma	919	2.2%
Oxyphilic adenocarcinoma	350	0.8%
Lymphoepithelial carcinoma	342	0.8%
Secretory carcinoma	315	0.8%
Neuroendocrine carcinoma	308	0.7%
Polymorphous adenocarcinoma	212	0.5%
Cystadenocarcinoma	189	0.5%
Basaloid squamous cell carcinoma	169	0.4%
Clear cell adenocarcinoma	138	0.3%
Merkel cell carcinoma	104	0.3%
Mucinous adenocarcinoma	99	0.2%
Papillary adenocarcinoma	60	0.1%
Basaloid carcinoma	59	0.1%
Papillary cystadenocarcinoma	46	0.1%
Cribriform carcinoma	45	0.1%
Adenocarcinoma with mixed subtypes	44	0.1%
Sebaceous carcinoma	44	0.1%
Mucin-producing adenocarcinoma	39	<0.1%

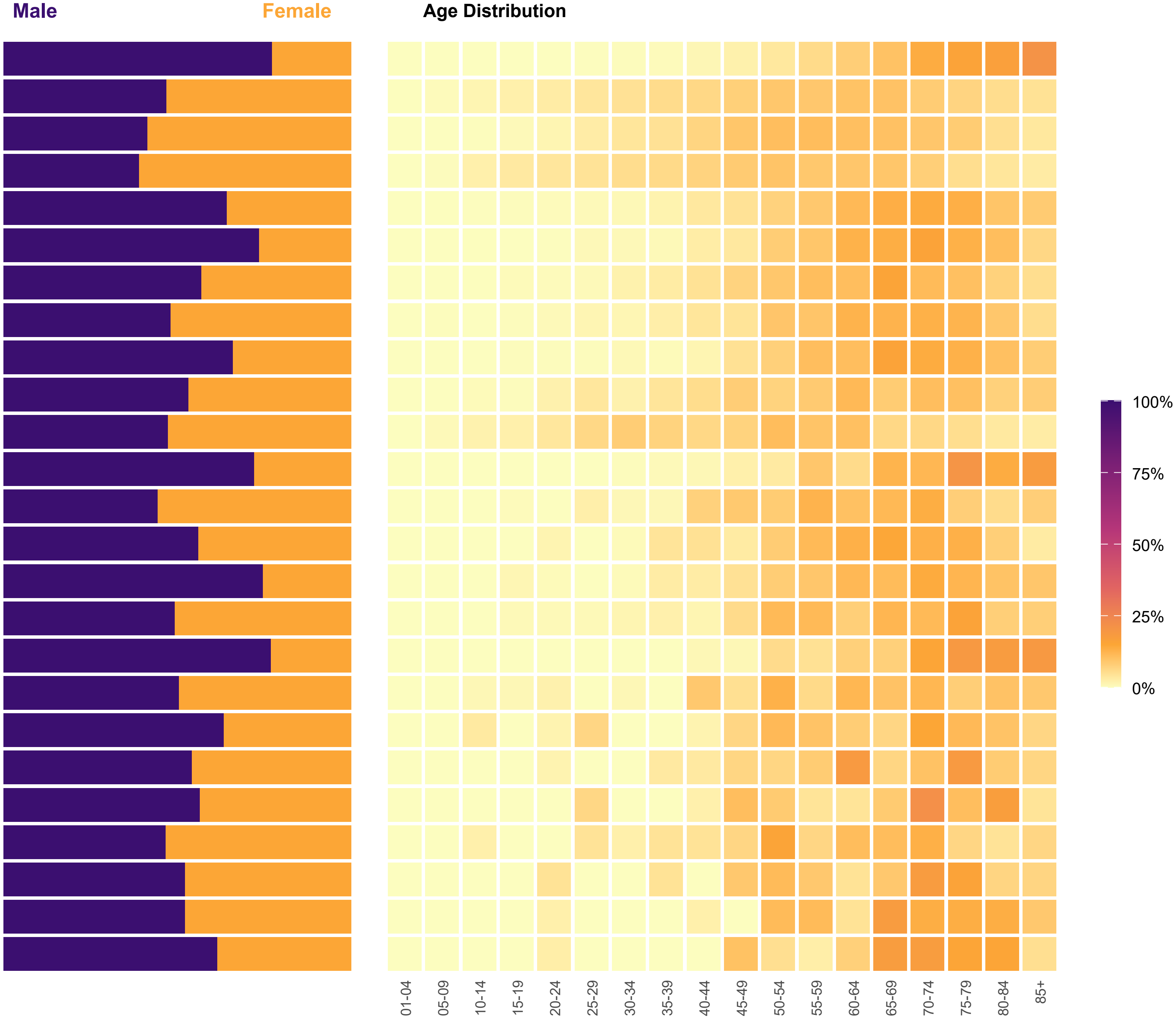

Primary Site: Major Salivary Glands | Phenotype: Grouped Phenotypes

Top 23 Morphologies | cases: 8,583

Morphology	n	%
Marginal zone B-cell lymphoma	2,021	23.5%
Diffuse large B-cell lymphoma	1,169	13.6%
Epithelial-myoepithelial carcinoma	1,060	12.3%
Myoepithelial carcinoma	926	10.8%
Follicular lymphoma grade 1/2	752	8.8%
lymphoma non-Hodgkin	420	4.9%
Follicular lymphoma	389	4.5%
Small cell carcinoma	385	4.5%
Follicular lymphoma grade 3	265	3.1%
Large cell carcinoma	165	1.9%
Carcinosarcoma	153	1.8%
lymphoma	129	1.5%
Mantle cell lymphoma	93	1.1%
Malignant melanoma	85	1.0%
B-cell chronic lymphocytic…	50	0.6%
Burkitt lymphoma	48	0.6%
Mature T-cell lymphoma	48	0.6%
Hodgkin lymphoma nodular…	46	0.5%
Large cell neuroendocrine carcinoma	39	0.5%
Anaplastic large cell lymphoma…	33	0.4%
Hodgkin lymphoma	31	0.4%
Plasmacytoma extramedullary	29	0.3%
Hodgkin lymphoma nodular sclerosis	27	0.3%

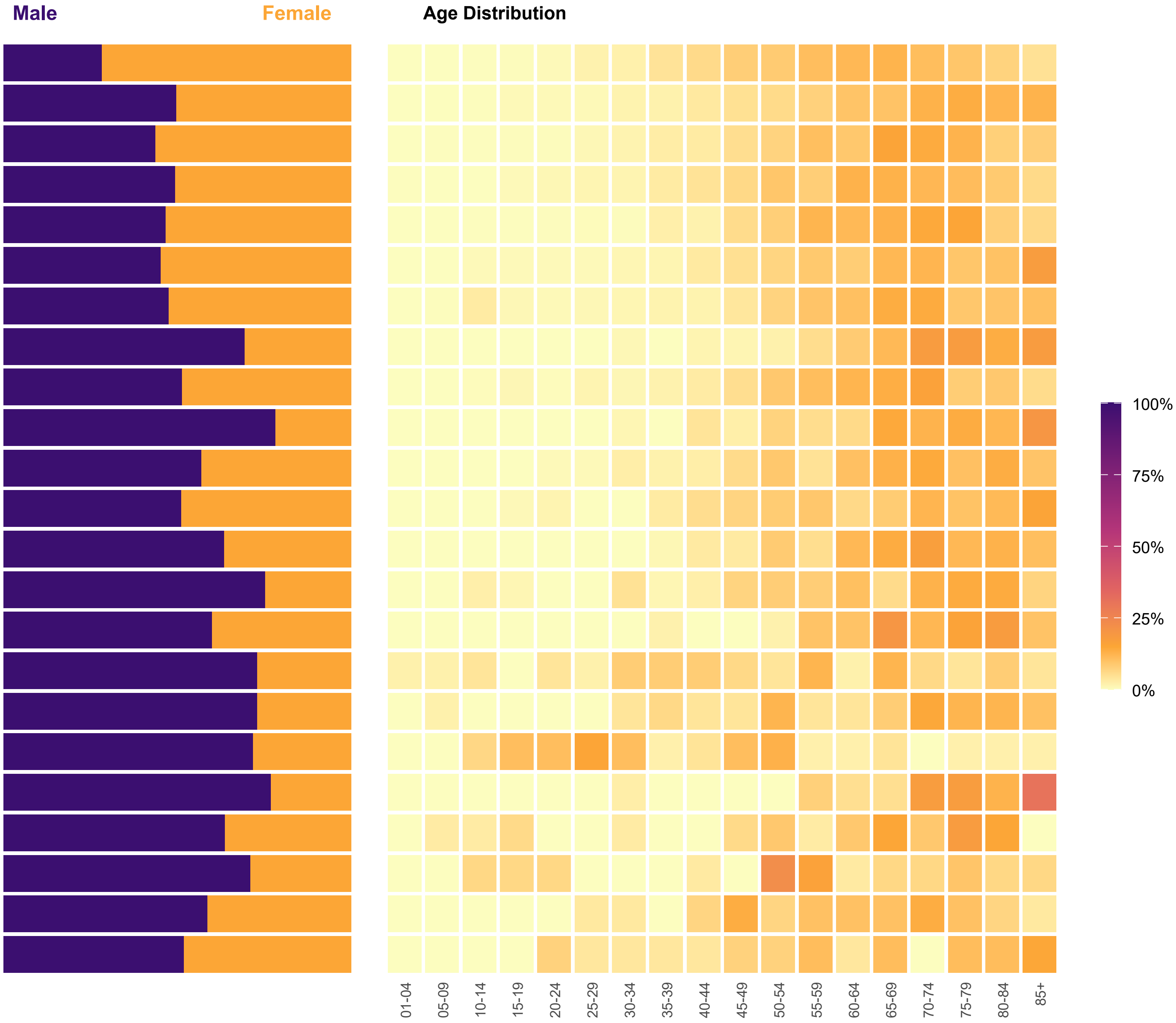

Primary Site: Mandible | Phenotype: epithelial

Top 3 Morphologies | cases: 733

Morphology	n	%
Squamous cell carcinoma	330	45.0%
Ameloblastoma metastasizing	241	32.9%
Odontogenic tumor	139	19.0%

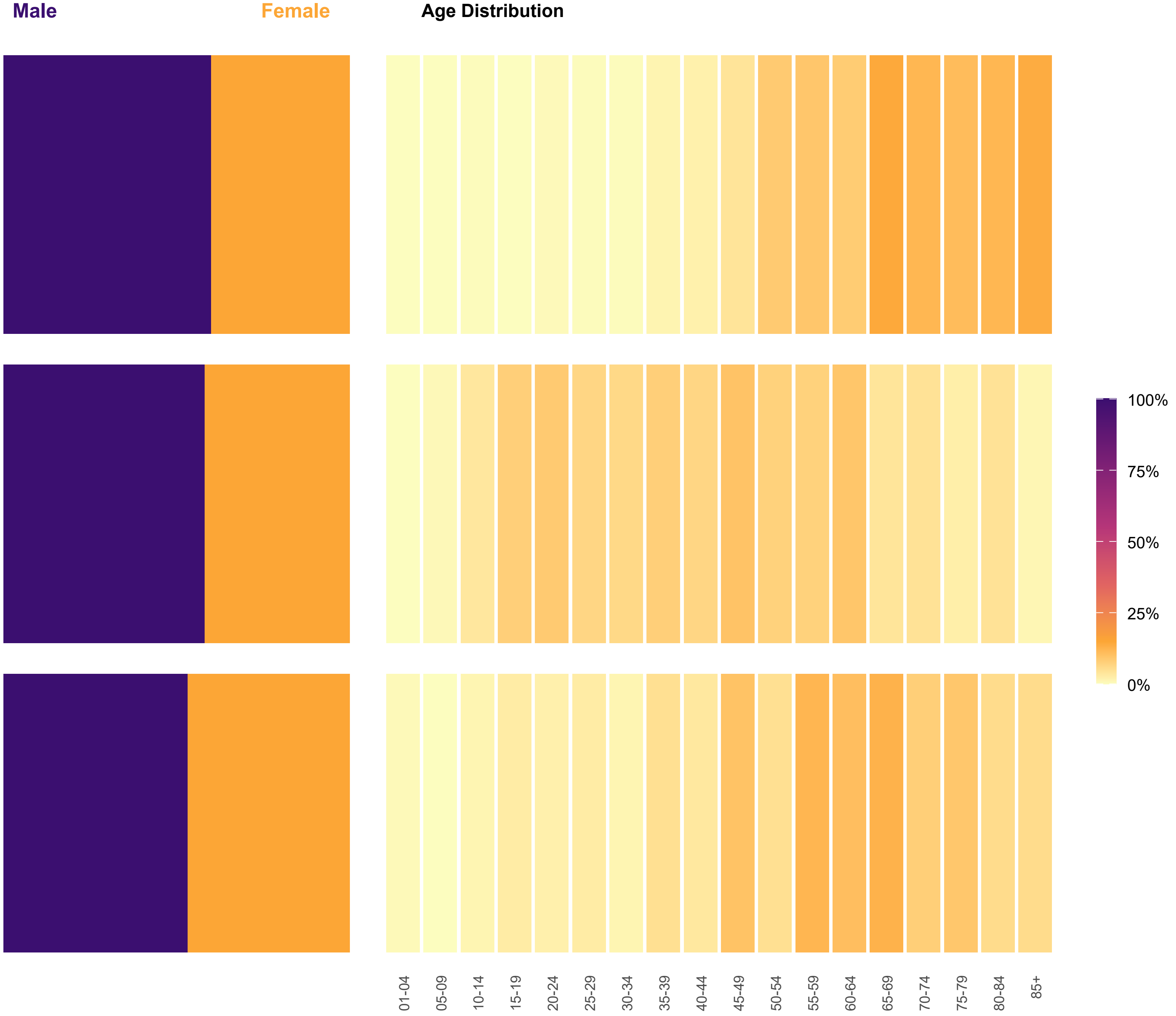

Primary Site: Mandible | Phenotype: Grouped Phenotypes

Top 9 Morphologies | cases: 1,189

Morphology	n	%
Osteosarcoma	364	30.6%
Chondroblastic osteosarcoma	139	11.7%
Diffuse large B-cell lymphoma	138	11.6%
Plasmacytoma	85	7.1%
Langerhans cell histiocytosis…	72	6.1%
Chondrosarcoma	52	4.4%
Fibroblastic osteosarcoma	39	3.3%
Sarcoma	32	2.7%
lymphoma non-Hodgkin	20	1.7%

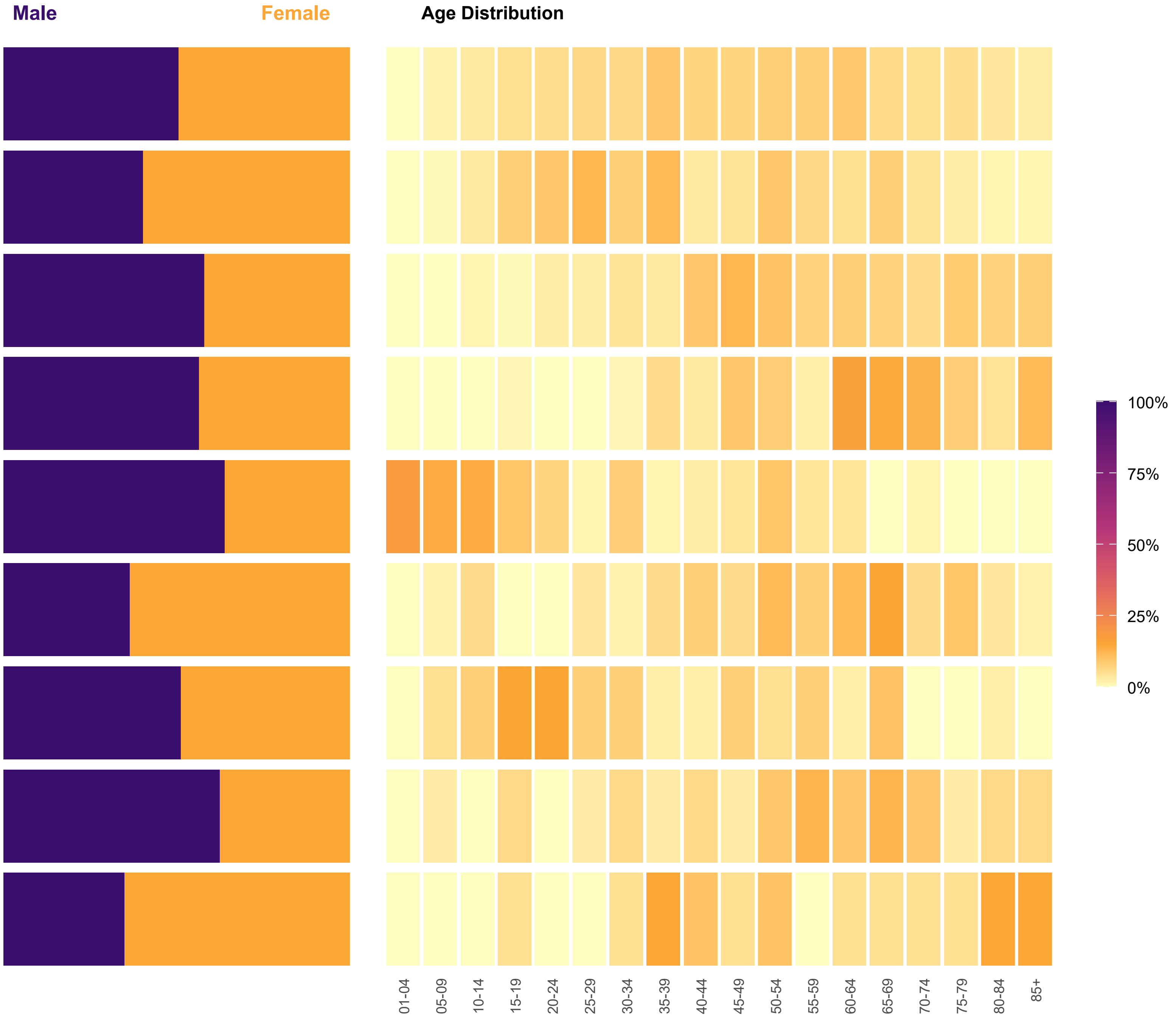

Primary Site: Meninges | Phenotype: Grouped Phenotypes

Top 10 Morphologies | cases: 5,405

Morphology	n	%
Meningiomamalignant	4,397	81.4%
Papillary meningioma	271	5.0%
Ependymoma	159	2.9%
Meningeal sarcomatosis	121	2.2%
Diffuse large B-cell lymphoma	57	1.1%
Malignant melanoma	47	0.9%
Marginal zone B-cell lymphoma	38	0.7%
Meningeal melanomatosis	31	0.6%
Glioblastoma	31	0.6%
Solitary fibrous tumormalignant	29	0.5%

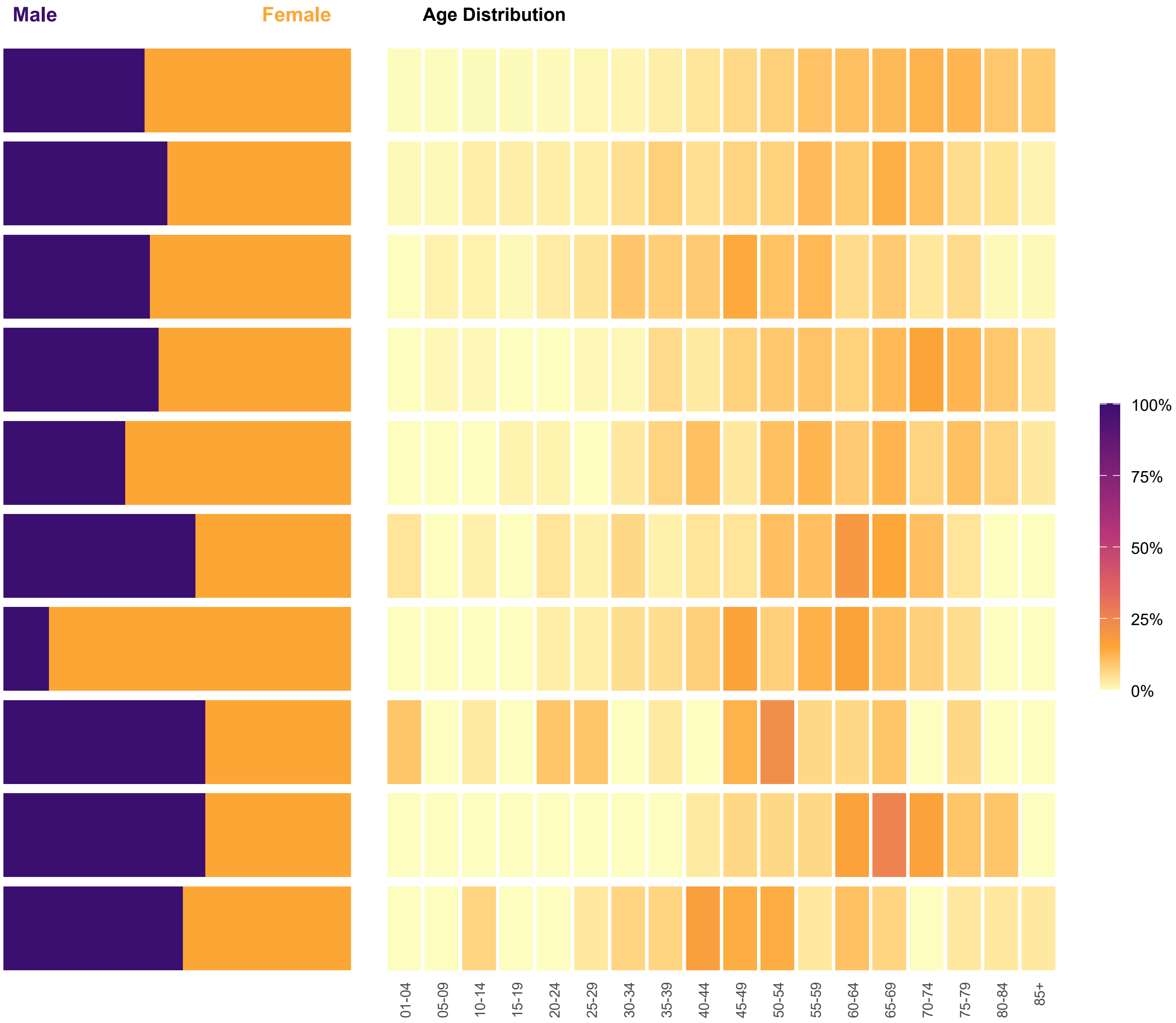

Primary Site: Mouth Other | Phenotype: epithelial

Top 11 Morphologies | cases: 11,724

Morphology	n	%
Squamous cell carcinoma	9,248	78.9%
Mucoepidermoid carcinoma	727	6.2%
Adenoid cystic carcinoma	578	4.9%
Adenocarcinoma	381	3.2%
Polymorphous adenocarcinoma	377	3.2%
Basaloid squamous cell carcinoma	78	0.7%
Acinar cell carcinoma	56	0.5%
Clear cell adenocarcinoma	54	0.5%
Papillary squamous cell carcinoma	43	0.4%
Basal cell adenocarcinoma	41	0.3%
Carcinoma ex pleomorphic adenoma	23	0.2%

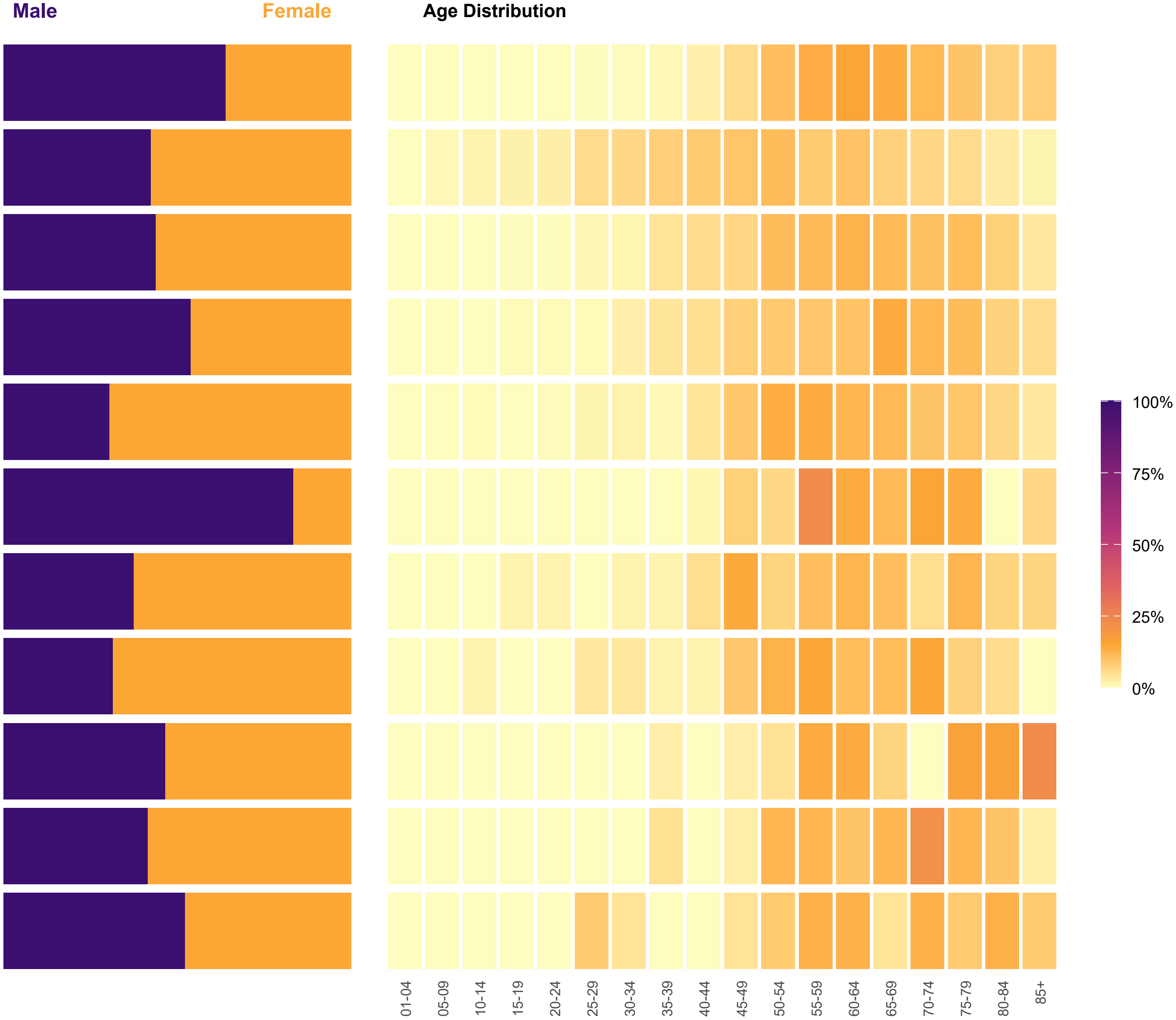

Primary Site: Mouth Other | Phenotype: Grouped Phenotypes

Top 8 Morphologies | cases: 805

Morphology	n	%
Diffuse large B-cell lymphoma	161	20.0%
Kaposi sarcoma	151	18.8%
Malignant melanoma	76	9.4%
Marginal zone B-cell lymphoma	62	7.7%
Myoepithelial carcinoma	49	6.1%
lymphoma non-Hodgkin	29	3.6%
Follicular lymphoma grade 1/2	27	3.4%
Plasmablastic lymphoma	20	2.5%

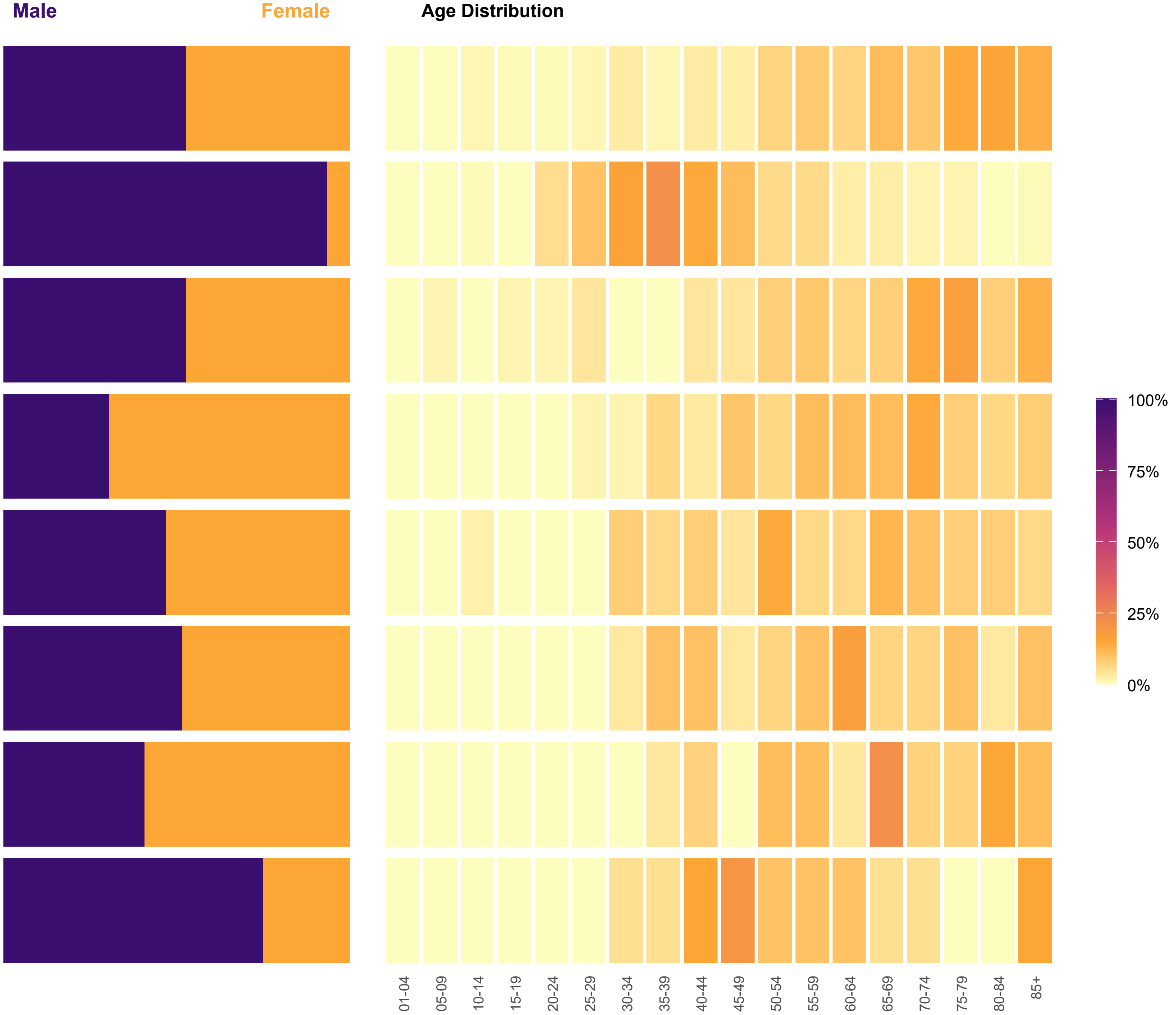

Primary Site: Nasal Cavity and Paranasal Sinuses | Phenotype: epithelial

Top 25 Morphologies | cases: 19,507

Morphology	n	%
Squamous cell carcinoma	14,378	73.7%
Adenoid cystic carcinoma	1,320	6.8%
Adenocarcinoma	1,266	6.5%
Basaloid squamous cell carcinoma	406	2.1%
Neuroendocrine carcinoma	319	1.6%
Mucoepidermoid carcinoma	281	1.4%
Papillary squamous cell carcinoma	254	1.3%
Adenocarcinoma intestinal type	249	1.3%
Schneiderian carcinoma	166	0.9%
Mucinous adenocarcinoma	125	0.6%
Basal cell carcinoma	86	0.4%
Papillary adenocarcinoma	79	0.4%
Transitional cell carcinoma	59	0.3%
Lymphoepithelial carcinoma	58	0.3%
Basaloid carcinoma	44	0.2%
Basosquamous carcinoma	43	0.2%
Polymorphous adenocarcinoma	41	0.2%
Clear cell adenocarcinoma	33	0.2%
Adenocarcinoma with neuroendocrine…	31	0.2%
Acinar cell carcinoma	29	0.1%
Basal cell adenocarcinoma	28	0.1%
Mucin-producing adenocarcinoma	27	0.1%
Merkel cell carcinoma	25	0.1%
Adenocarcinoma with mixed subtypes	23	0.1%
Oxyphilic adenocarcinoma	21	0.1%

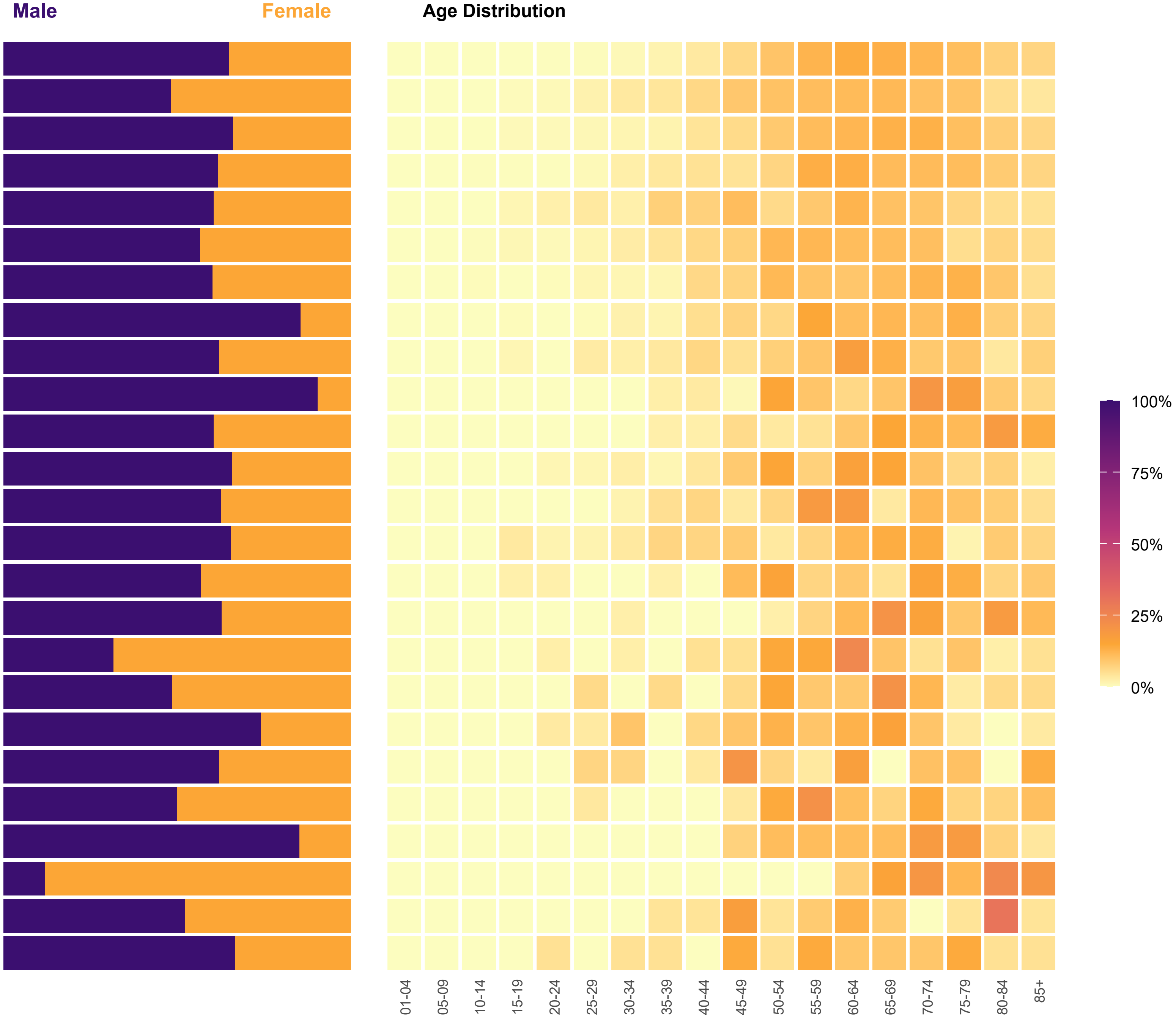

Primary Site: Nasal Cavity and Paranasal Sinuses | Phenotype: Grouped Phenotypes

Top 15 Morphologies | cases: 4,709

Morphology	n	%
Malignant melanoma	1,937	41.1%
Olfactory neuroblastoma	1,720	36.5%
Small cell carcinoma	229	4.9%
Amelanotic melanoma	141	3.0%
Mucosal lentiginous melanoma	112	2.4%
Large cell neuroendocrine carcinoma	60	1.3%
Epithelial-myoepithelial carcinoma	50	1.1%
Myoepithelial carcinoma	48	1.0%
Nodular melanoma	43	0.9%
Carcinosarcoma	42	0.9%
Teratocarcinoma	39	0.8%
Neuroblastoma	39	0.8%
Malignant peripheral nerve sheath…	37	0.8%
Spindle cell melanoma	35	0.7%
Epithelioid cell melanoma	27	0.6%

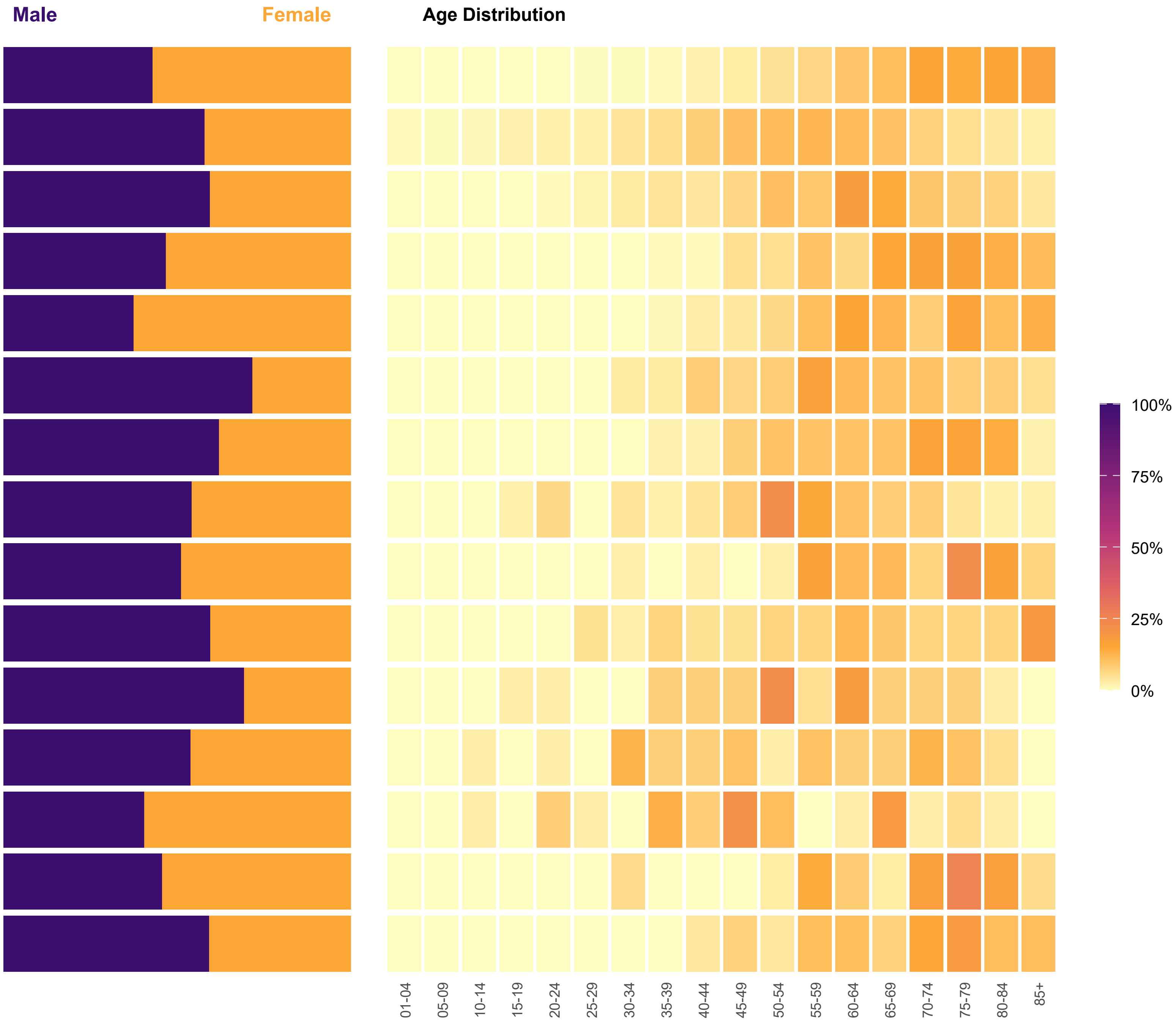

Primary Site: Nasopharynx | Phenotype: epithelial

Top 12 Morphologies | cases: 17,057

Morphology	n	%
Squamous cell carcinoma	13,544	79.4%
Lymphoepithelial carcinoma	2,233	13.1%
Adenoid cystic carcinoma	334	2.0%
Basaloid squamous cell carcinoma	255	1.5%
Adenocarcinoma	250	1.5%
Papillary squamous cell carcinoma	97	0.6%
Mucoepidermoid carcinoma	78	0.5%
Neuroendocrine carcinoma	72	0.4%
Papillary adenocarcinoma	47	0.3%
Schneiderian carcinoma	21	0.1%
Basaloid carcinoma	21	0.1%
Transitional cell carcinoma	20	0.1%

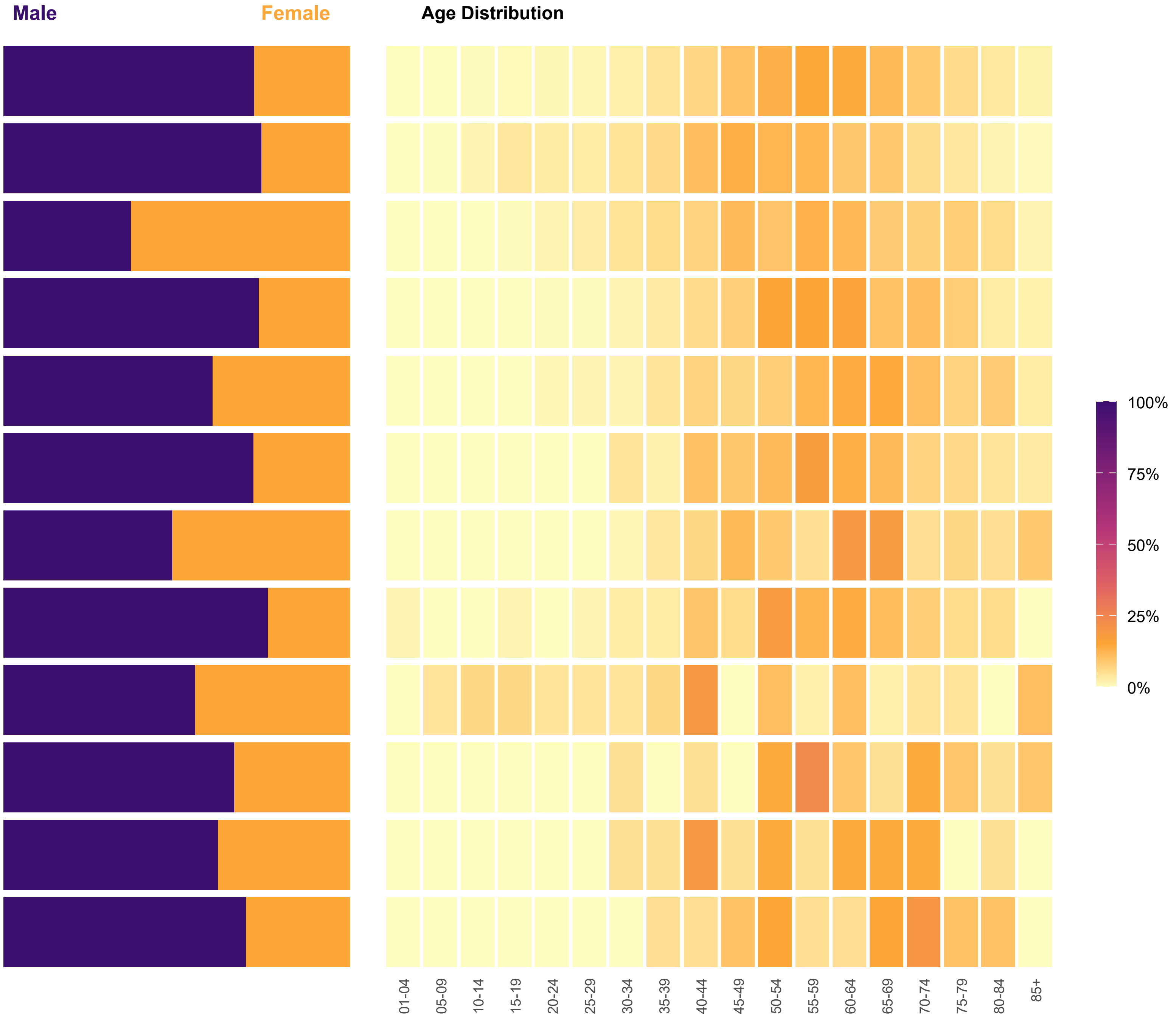

Primary Site: Nasopharynx | Phenotype: Grouped Phenotypes

Top 22 Morphologies | cases: 3,033

Morphology	n	%
Diffuse large B-cell lymphoma	1,033	34.1%
Marginal zone B-cell lymphoma	215	7.1%
Plasmacytoma extramedullary	177	5.8%
Mantle cell lymphoma	149	4.9%
NK/T-cell lymphoma nasal and nasal…	149	4.9%
lymphoma non-Hodgkin	143	4.7%
Small cell carcinoma	100	3.3%
Embryonal rhabdomyosarcoma	100	3.3%
Burkitt lymphoma	93	3.1%
Follicular lymphoma grade 1/2	86	2.8%
Malignant melanoma	78	2.6%
Follicular lymphoma	64	2.1%
lymphoma	55	1.8%
Large cell carcinoma	46	1.5%
Mature T-cell lymphoma	42	1.4%
Alveolar rhabdomyosarcoma	34	1.1%
Plasmacytoma	32	1.1%
B-cell chronic lymphocytic…	31	1.0%
Rhabdomyosarcoma	30	1.0%
Chordoma	28	0.9%
Follicular lymphoma grade 3	28	0.9%
Sarcoma	22	0.7%

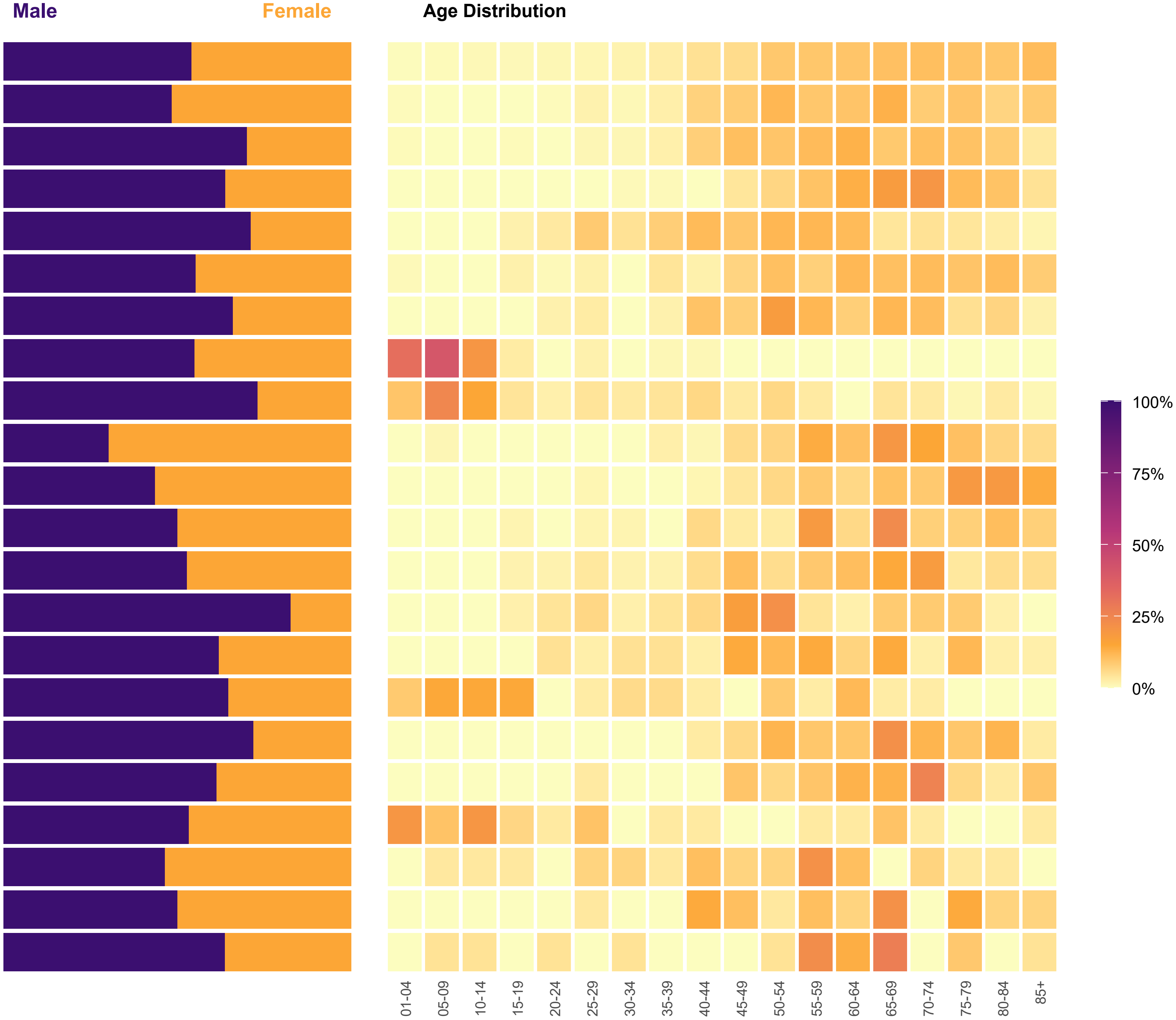

Primary Site: Oropharynx | Phenotype: epithelial

Top 14 Morphologies | cases: 125,665

Morphology	n	%
Squamous cell carcinoma	119,024	94.7%
Basaloid squamous cell carcinoma	3,513	2.8%
Mucoepidermoid carcinoma	642	0.5%
Lymphoepithelial carcinoma	493	0.4%
Papillary squamous cell carcinoma	486	0.4%
Adenocarcinoma	444	0.4%
Adenoid cystic carcinoma	424	0.3%
Polymorphous adenocarcinoma	231	0.2%
Neuroendocrine carcinoma	92	<0.1%
Basaloid carcinoma	63	<0.1%
Acinar cell carcinoma	46	<0.1%
Clear cell adenocarcinoma	33	<0.1%
Basosquamous carcinoma	32	<0.1%
Basal cell adenocarcinoma	26	<0.1%

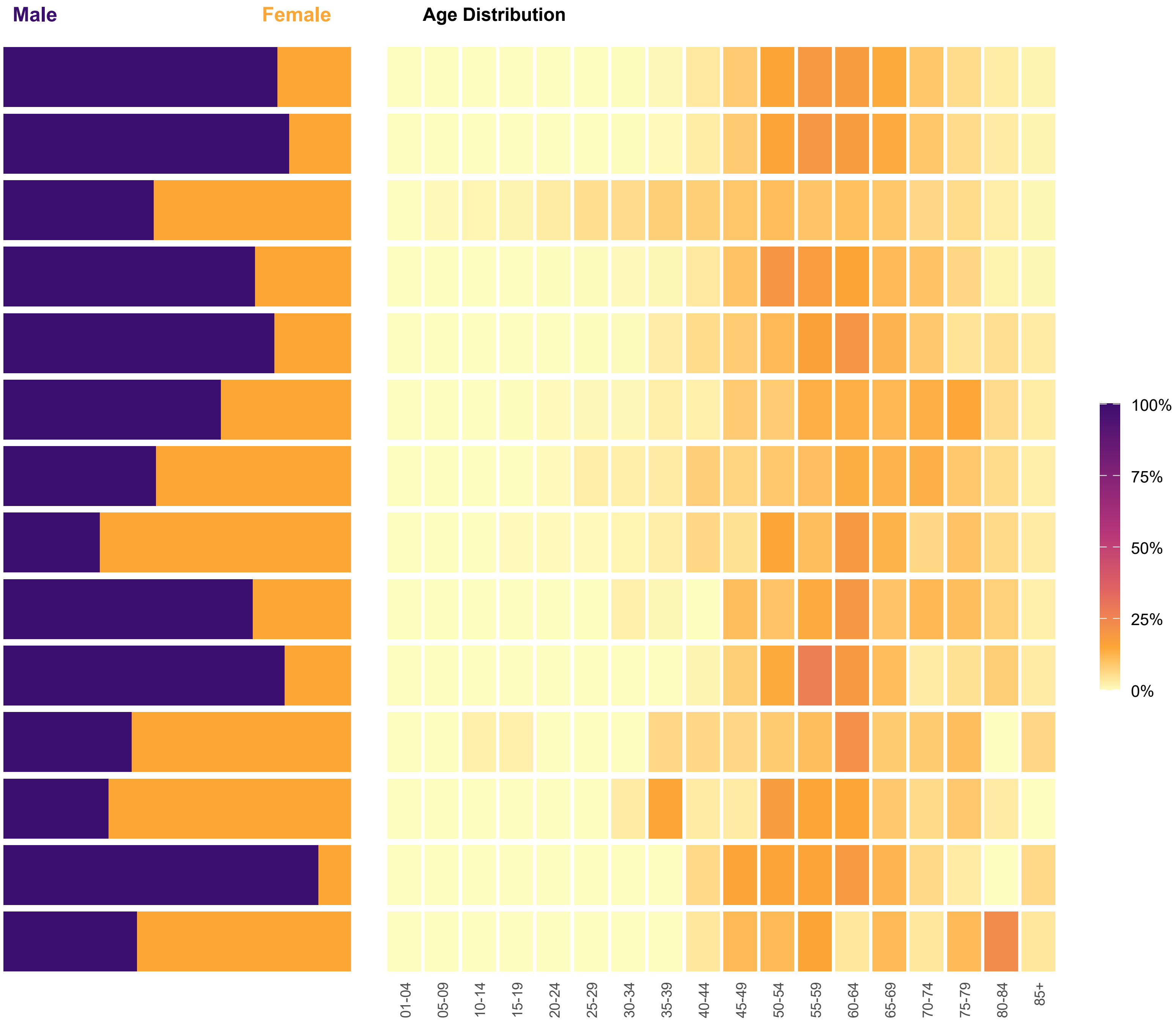

Primary Site: Oropharynx | Phenotype: Grouped Phenotypes

Top 23 Morphologies | cases: 6,304

Morphology	n	%
Diffuse large B-cell lymphoma	3,358	53.3%
Mantle cell lymphoma	432	6.9%
Follicular lymphoma grade 1/2	295	4.7%
lymphoma non-Hodgkin	293	4.6%
Follicular lymphoma grade 3	251	4.0%
Marginal zone B-cell lymphoma	248	3.9%
Follicular lymphoma	186	3.0%
Burkitt lymphoma	163	2.6%
Plasmacytoma extramedullary	140	2.2%
Mature T-cell lymphoma	132	2.1%
Small cell carcinoma	95	1.5%
lymphoma	95	1.5%
B-cell chronic lymphocytic…	76	1.2%
Malignant melanoma	52	0.8%
Lymphoma large B-cell diffuse…	51	0.8%
NK/T-cell lymphoma nasal and nasal…	45	0.7%
Myoepithelial carcinoma	33	0.5%
Large cell carcinoma	32	0.5%
Large cell neuroendocrine carcinoma	27	0.4%
Plasmacytoma	25	0.4%
Hodgkin lymphoma	24	0.4%
Carcinosarcoma	20	0.3%
Hodgkin lymphoma mixed cellularity	20	0.3%

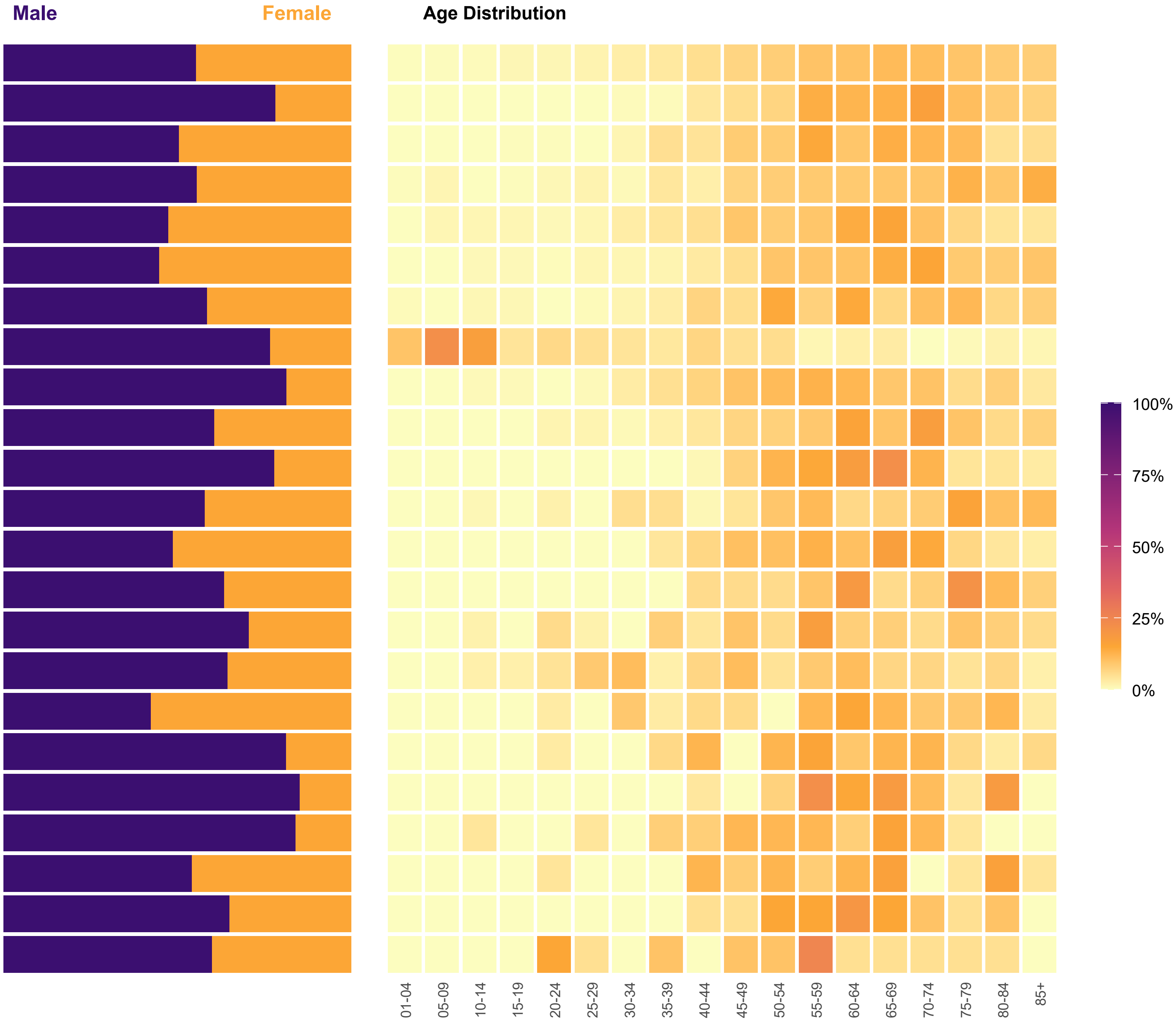

Primary Site: Ovary | Phenotype: epithelial

Top 25 Morphologies | cases: 235,884

Morphology	n	%
Serous carcinoma	59,551	25.2%
Low grade serous carcinoma	53,810	22.8%
Adenocarcinoma	32,277	13.7%
Endometrioid adenocarcinoma	24,011	10.2%
High grade serous carcinoma	17,848	7.6%
Clear cell adenocarcinoma	11,278	4.8%
Mucinous adenocarcinoma	10,391	4.4%
Mixed cell adenocarcinoma	6,068	2.6%
Papillary adenocarcinoma	5,724	2.4%
Mucinous cystadenocarcinoma	4,729	2.0%
Cystadenocarcinoma	2,102	0.9%
Papillary cystadenocarcinoma	1,880	0.8%
Squamous cell carcinoma	903	0.4%
Mucin-producing adenocarcinoma	637	0.3%
Transitional cell carcinoma	618	0.3%
Brenner tumor malignant	599	0.3%
Neuroendocrine carcinoma	486	0.2%
Adenocarcinoma with mixed subtypes	454	0.2%
Signet ring cell carcinoma	357	0.2%
Adenocarcinoma with squamous…	292	0.1%
Struma ovariimalignant	273	0.1%
Serous adenocarcinofibroma	209	<0.1%
Adenocarcinoma in tubulovillous…	158	<0.1%
Endometrioid adenofibroma malignant	145	<0.1%
Seromucinous carcinoma	138	<0.1%

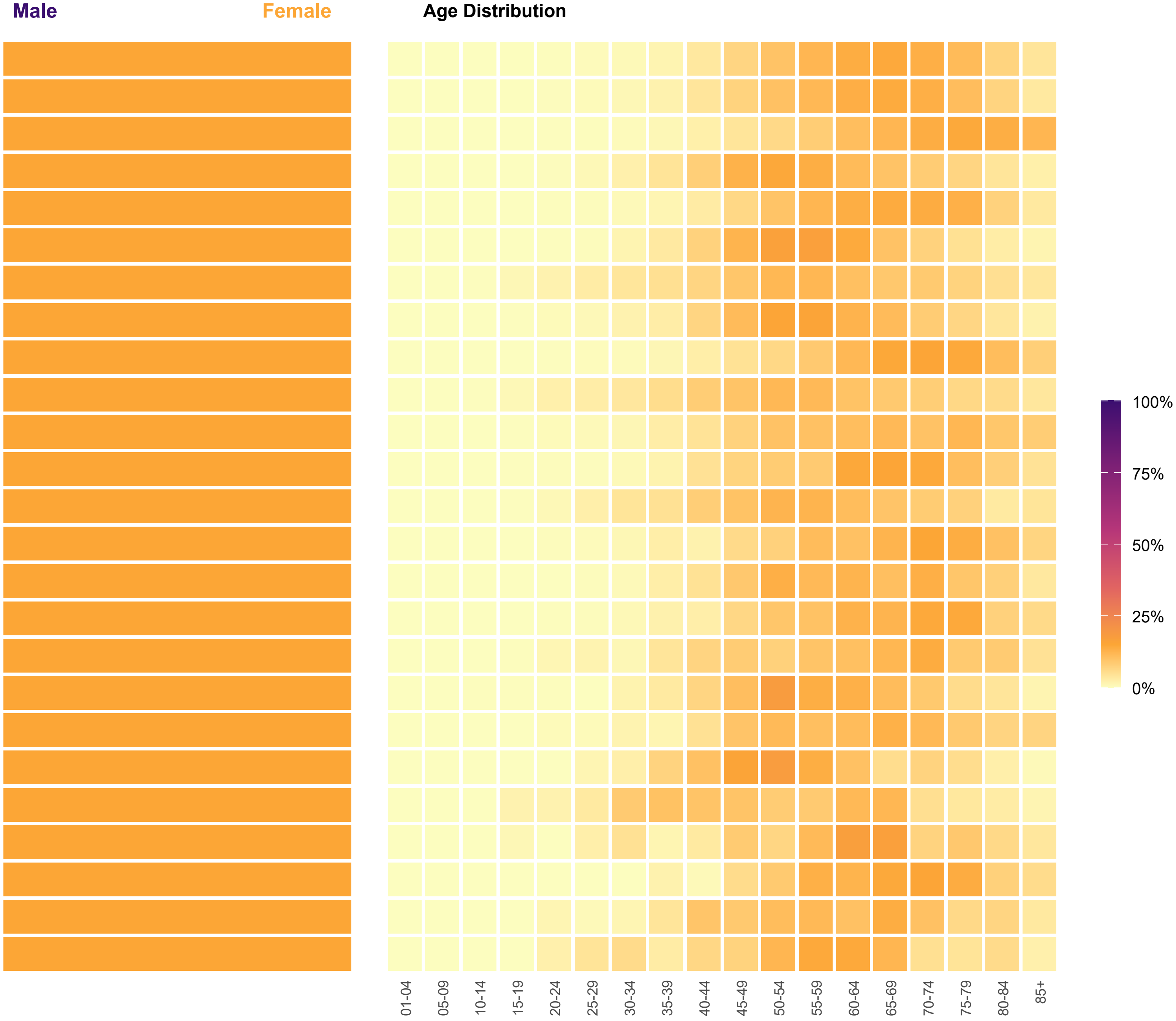

Primary Site: Ovary | Phenotype: germ cell

Top 13 Morphologies | cases: 8,988

Morphology	n	%
Adult granulosa cell tumor of ovary	4,575	50.9%
Dysgerminoma	1,737	19.3%
Yolk sac tumor	871	9.7%
Mixed germ cell tumor	709	7.9%
Sertoli-Leydig cell tumor	429	4.8%
Germinoma	225	2.5%
Steroid cell tumor malignant	124	1.4%
Choriocarcinoma	83	0.9%
Mesonephroma	46	0.5%
Sertoli cell carcinoma	40	0.4%
Choriocarcinoma combined with…	37	0.4%
Embryonal carcinoma	36	0.4%
Thecoma malignant	26	0.3%

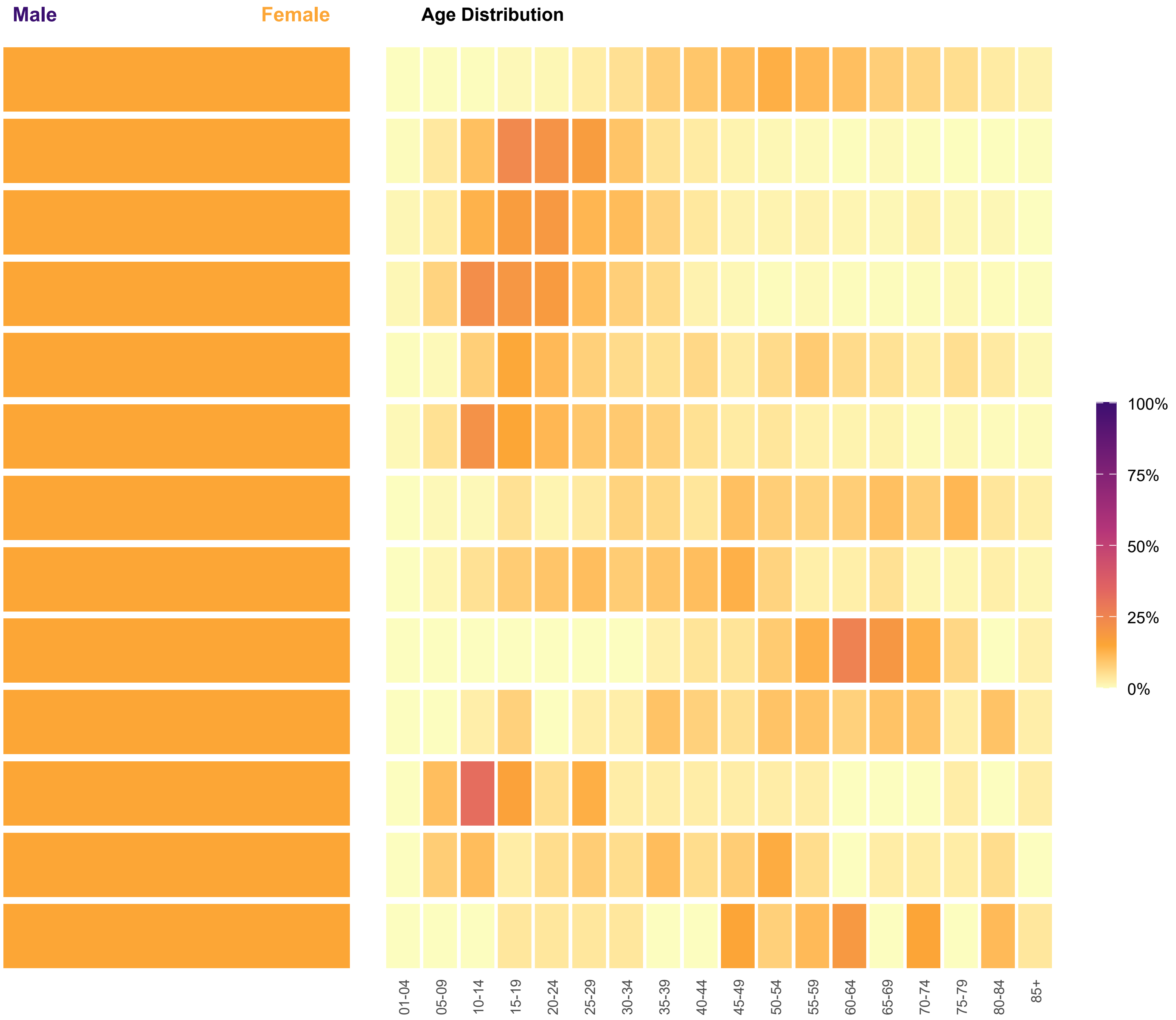

Primary Site: Ovary | Phenotype: hematopoietic

Top 3 Morphologies | cases: 361

Morphology	n	%
Diffuse large B-cell lymphoma	212	58.7%
Burkitt lymphoma	38	10.5%
Follicular lymphoma grade 1/2	22	6.1%

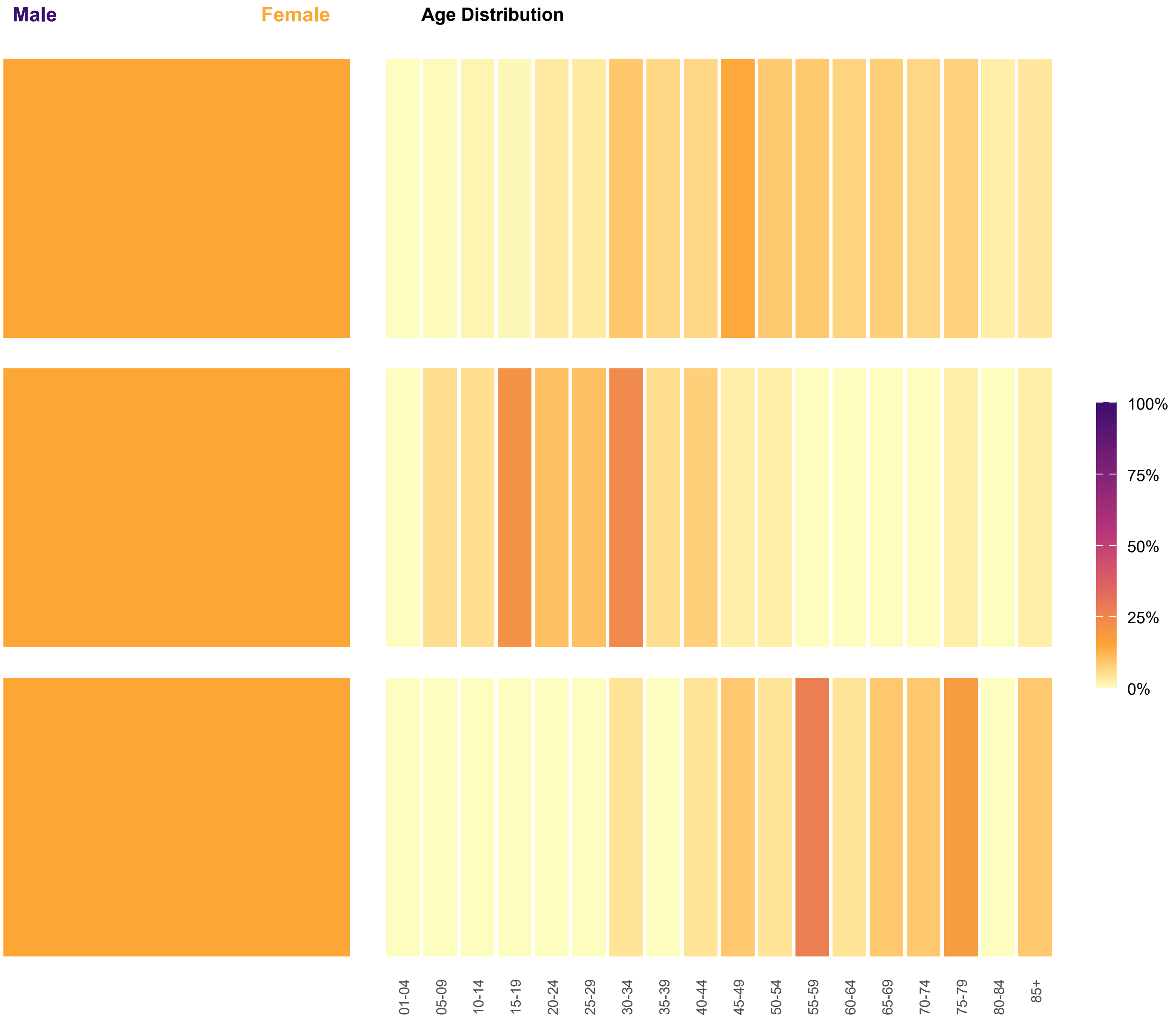

Primary Site: Ovary | Phenotype: mesenchymal

Top 12 Morphologies | cases: 1,175

Morphology	n	%
Leiomyosarcoma	274	23.3%
Fibrosarcoma	149	12.7%
Sarcoma	133	11.3%
Adenosarcoma	120	10.2%
Granular cell carcinoma	77	6.6%
Stromal sarcoma	54	4.6%
Endometrial stromal sarcoma low…	40	3.4%
Endometrial stromal sarcoma	35	3.0%
Rhabdomyosarcoma	31	2.6%
Desmoplastic small round cell tumor	27	2.3%
Mesotheliomamalignant	23	2.0%
Spindle cell sarcoma	21	1.8%

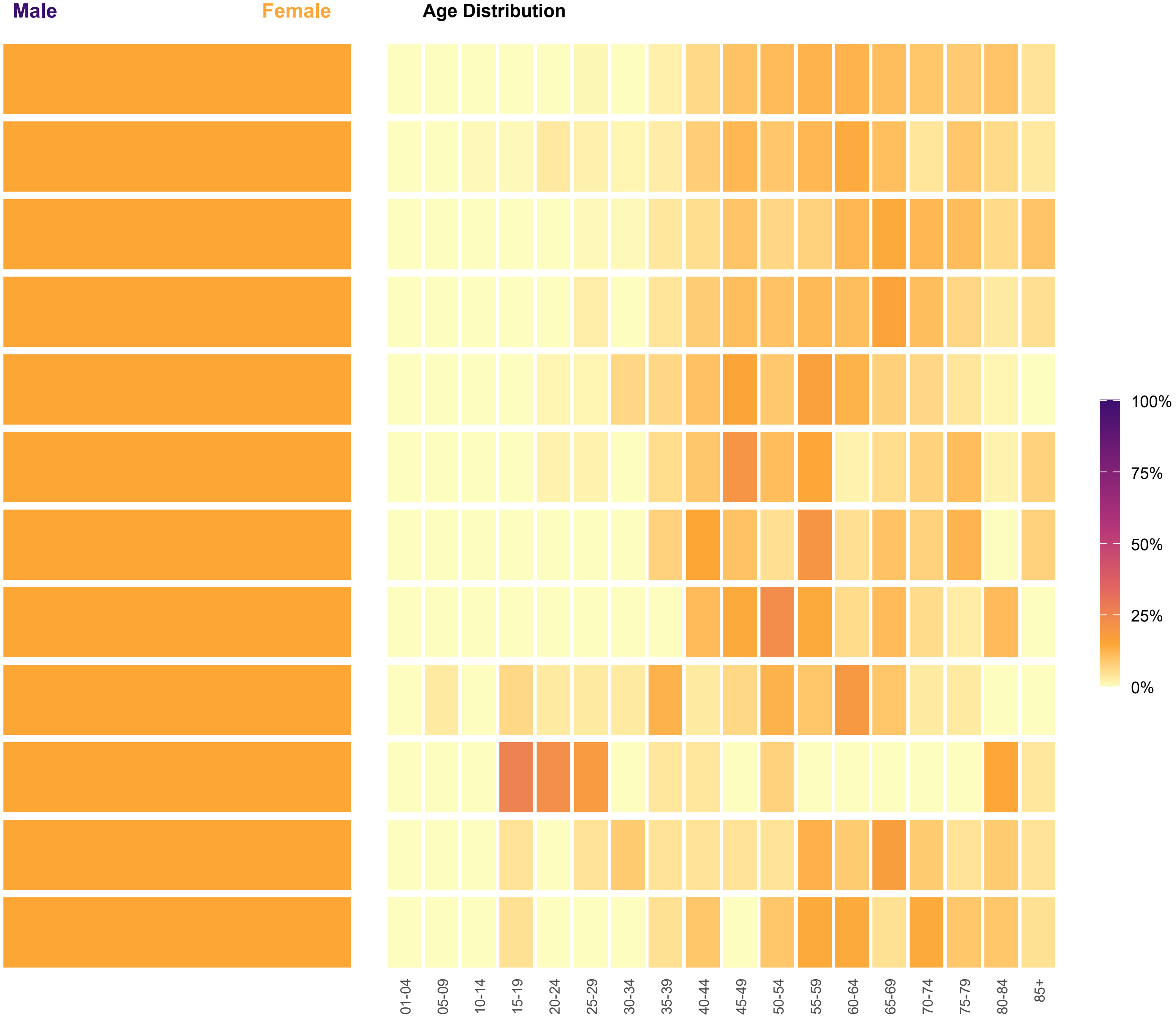

Primary Site: Ovary | Phenotype: mixed or multipotent stem cell

Top 8 Morphologies | cases: 9,138

Morphology	n	%
Carcinosarcoma	3,144	34.4%
Mulerian mixed tumor	2,944	32.2%
Teratoma	2,125	23.3%
Mesodermal mixed tumor	572	6.3%
Teratoma with malignant…	186	2.0%
Teratocarcinoma	45	0.5%
Goblet cell carcinoid	41	0.4%
Mixed adenoneuroendocrine carcinoma	23	0.3%

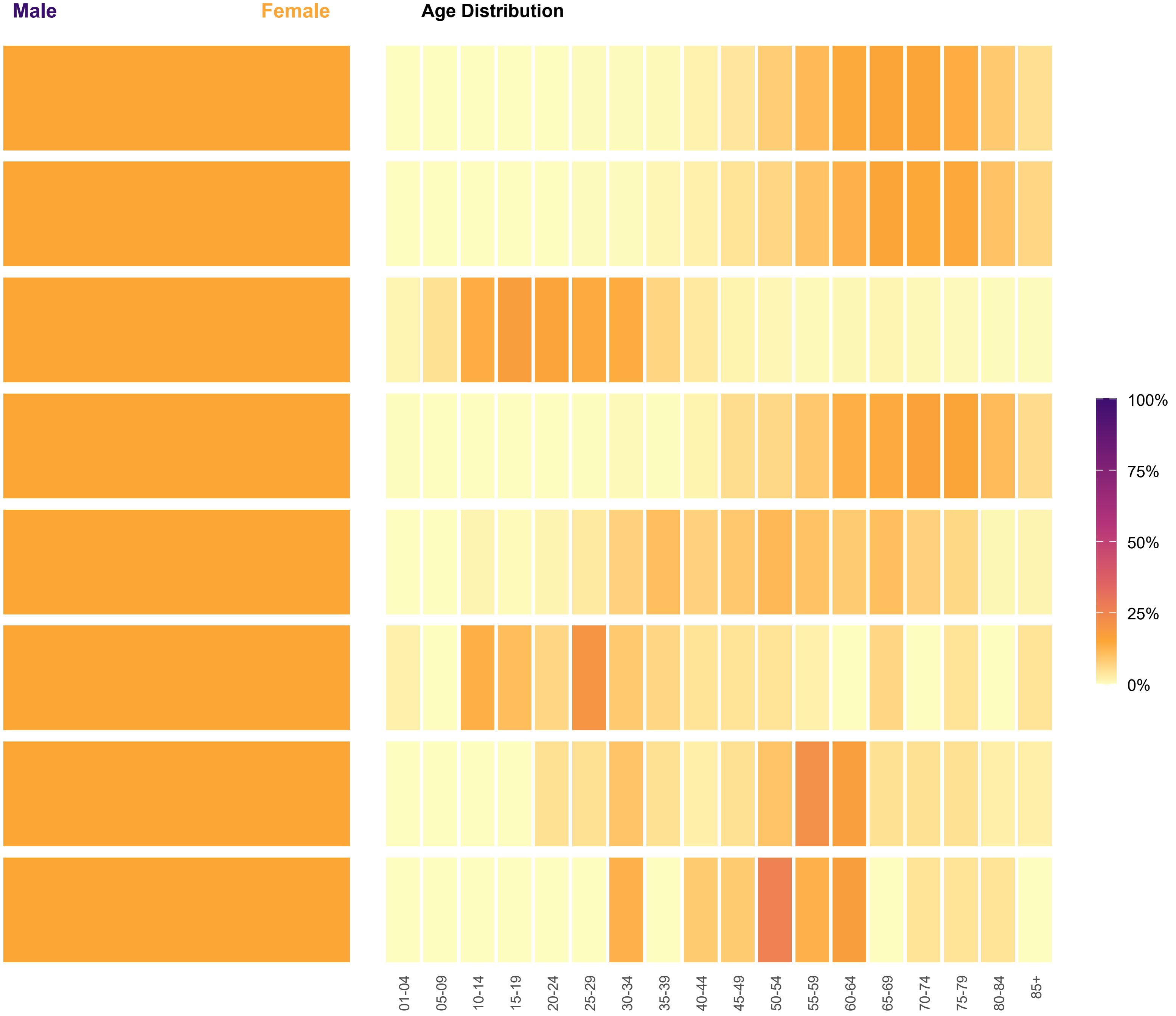

Primary Site: Ovary | Phenotype: neuroectodermal

Top 8 Morphologies | cases: 1,932

Morphology	n	%
Neuroendocrine tumor	997	51.6%
Small cell carcinoma	562	29.1%
Large cell carcinoma	125	6.5%
Large cell neuroendocrine carcinoma	81	4.2%
Granular cell tumor malignant	41	2.1%
CNS embryonal tumor	35	1.8%
Neuroendocrine tumor grade 2	34	1.8%
Combined small cell carcinoma	21	1.1%

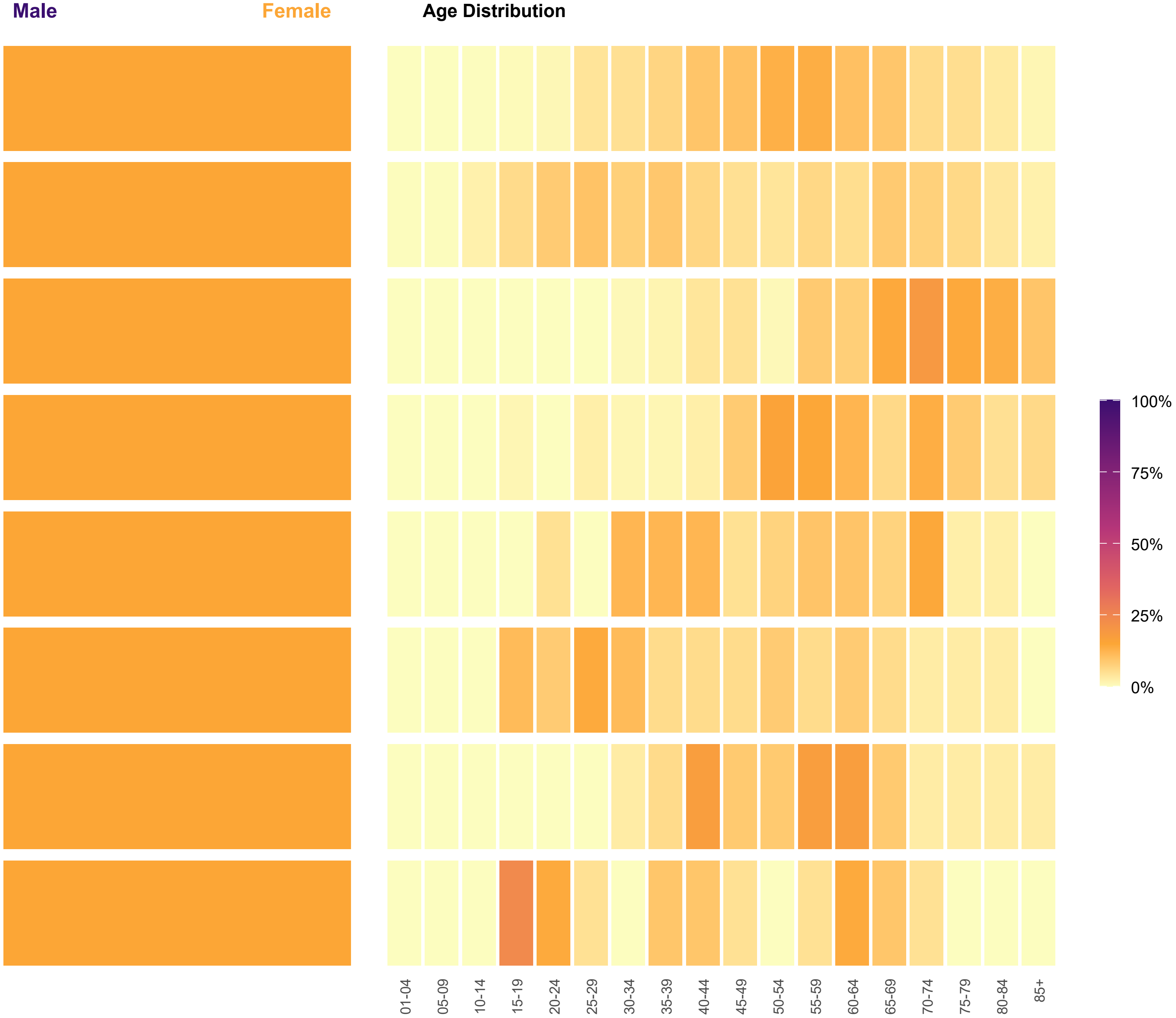

Primary Site: Palate excluding Soft and Uvula | Phenotype: epithelial

Top 9 Morphologies | cases: 5,621

Morphology	n	%
Squamous cell carcinoma	2,995	53.3%
Mucoepidermoid carcinoma	1,020	18.1%
Adenoid cystic carcinoma	686	12.2%
Polymorphous adenocarcinoma	425	7.6%
Adenocarcinoma	271	4.8%
Clear cell adenocarcinoma	33	0.6%
Papillary squamous cell carcinoma	32	0.6%
Basaloid squamous cell carcinoma	28	0.5%
Carcinoma ex pleomorphic adenoma	24	0.4%

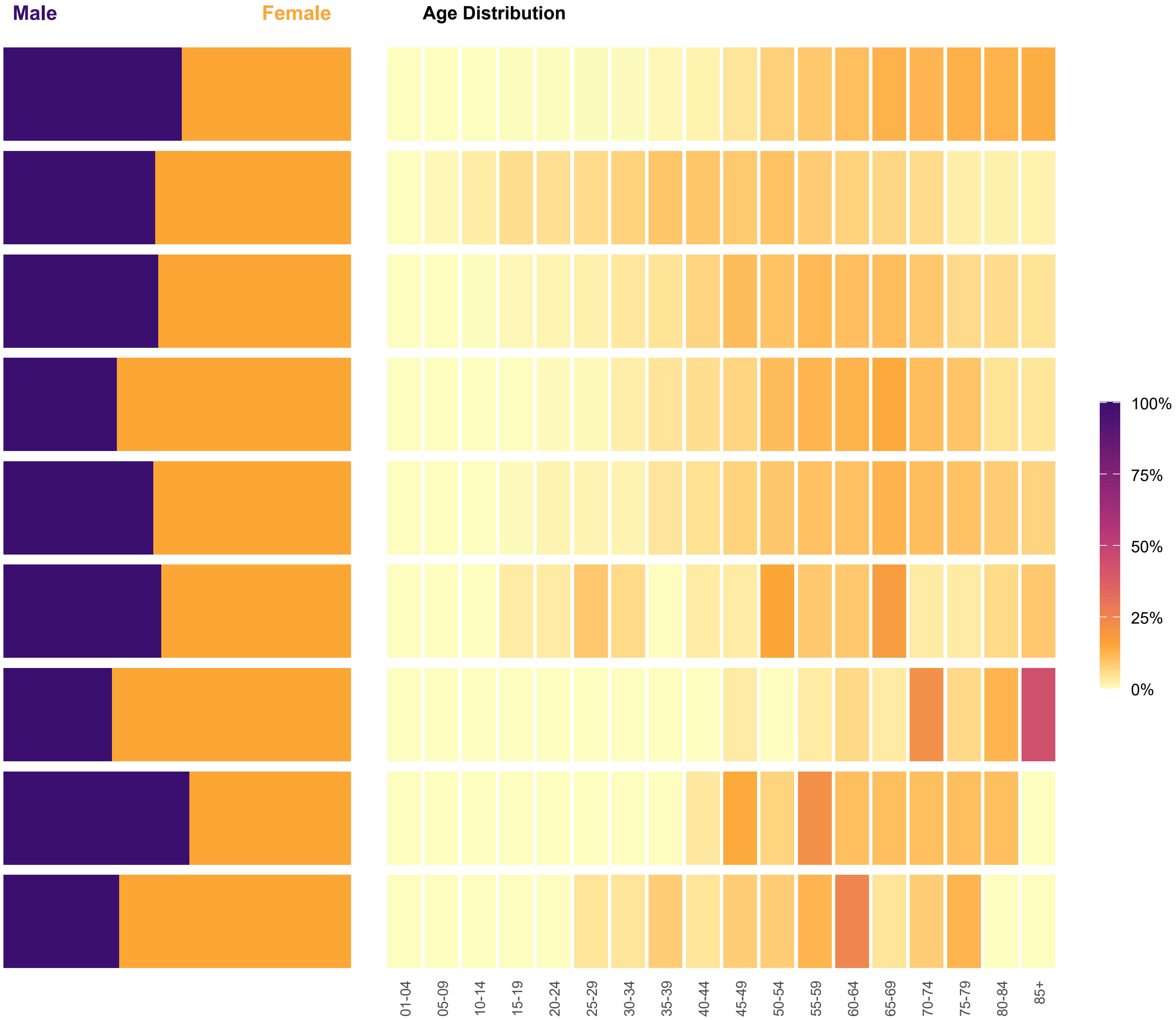

Primary Site: Palate excluding Soft and Uvula | Phenotype: Grouped Phenotypes

Top 10 Morphologies | cases: 820

Morphology	n	%
Kaposi sarcoma	143	17.4%
Diffuse large B-cell lymphoma	130	15.9%
Malignant melanoma	110	13.4%
Marginal zone B-cell lymphoma	61	7.4%
Myoepithelial carcinoma	49	6.0%
Follicular lymphoma grade 1/2	37	4.5%
lymphoma non-Hodgkin	30	3.7%
Follicular lymphoma	26	3.2%
Epithelial-myoepithelial carcinoma	25	3.0%
Mantle cell lymphoma	23	2.8%

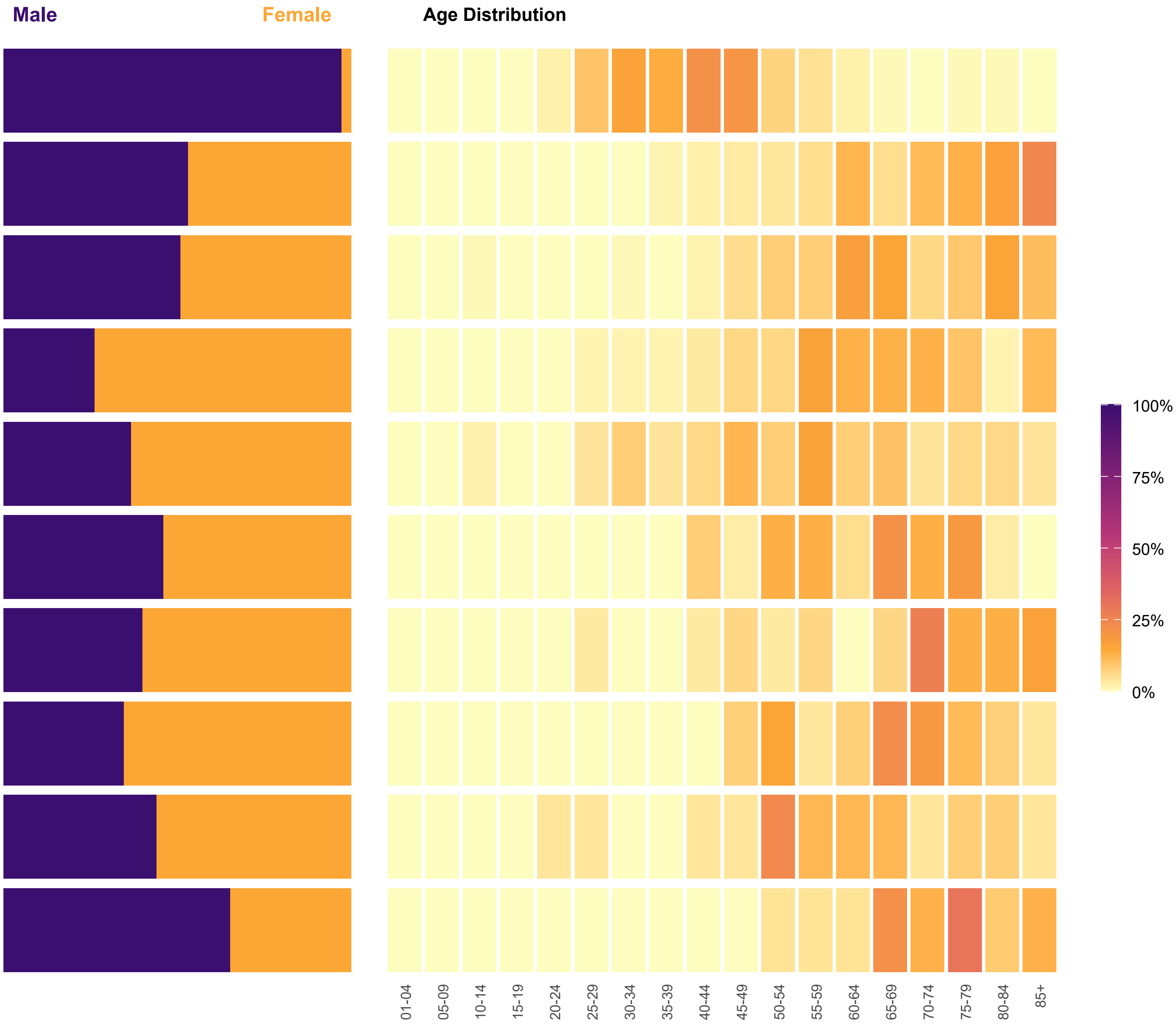

Primary Site: Pancreas | Phenotype: epithelial

Top 25 Morphologies | cases: 405,339

Morphology	n	%
Adenocarcinoma	282,671	69.7%
Infiltrating duct carcinoma	79,713	19.7%
Neuroendocrine carcinoma	14,279	3.5%
Mucinous adenocarcinoma	9,741	2.4%
Mucin-producing adenocarcinoma	5,302	1.3%
IPMN with an associated invasive…	2,068	0.5%
Acinar cell carcinoma	1,840	0.5%
Tubular adenocarcinoma	1,808	0.4%
Signet ring cell carcinoma	1,565	0.4%
Squamous cell carcinoma	987	0.2%
Mucinous cystadenocarcinoma	869	0.2%
Cystadenocarcinoma	584	0.1%
Papillary adenocarcinoma	549	0.1%
Adenocarcinoma with mixed subtypes	496	0.1%
Pancreatobiliary type carcinoma	392	<0.1%
Clear cell adenocarcinoma	310	<0.1%
Adenocarcinoma with neuroendocrine…	310	<0.1%
Cholangiocarcinoma	271	<0.1%
Scirrhous adenocarcinoma	264	<0.1%
Intraductal papillary…	219	<0.1%
Adenocarcinoma in tubulovillous…	142	<0.1%
Adenocarcinoma intestinal type	111	<0.1%
Infiltrating duct mixed with other…	78	<0.1%
Infiltrating ductular carcinoma	76	<0.1%
Duct carcinoma desmoplastic type	63	<0.1%

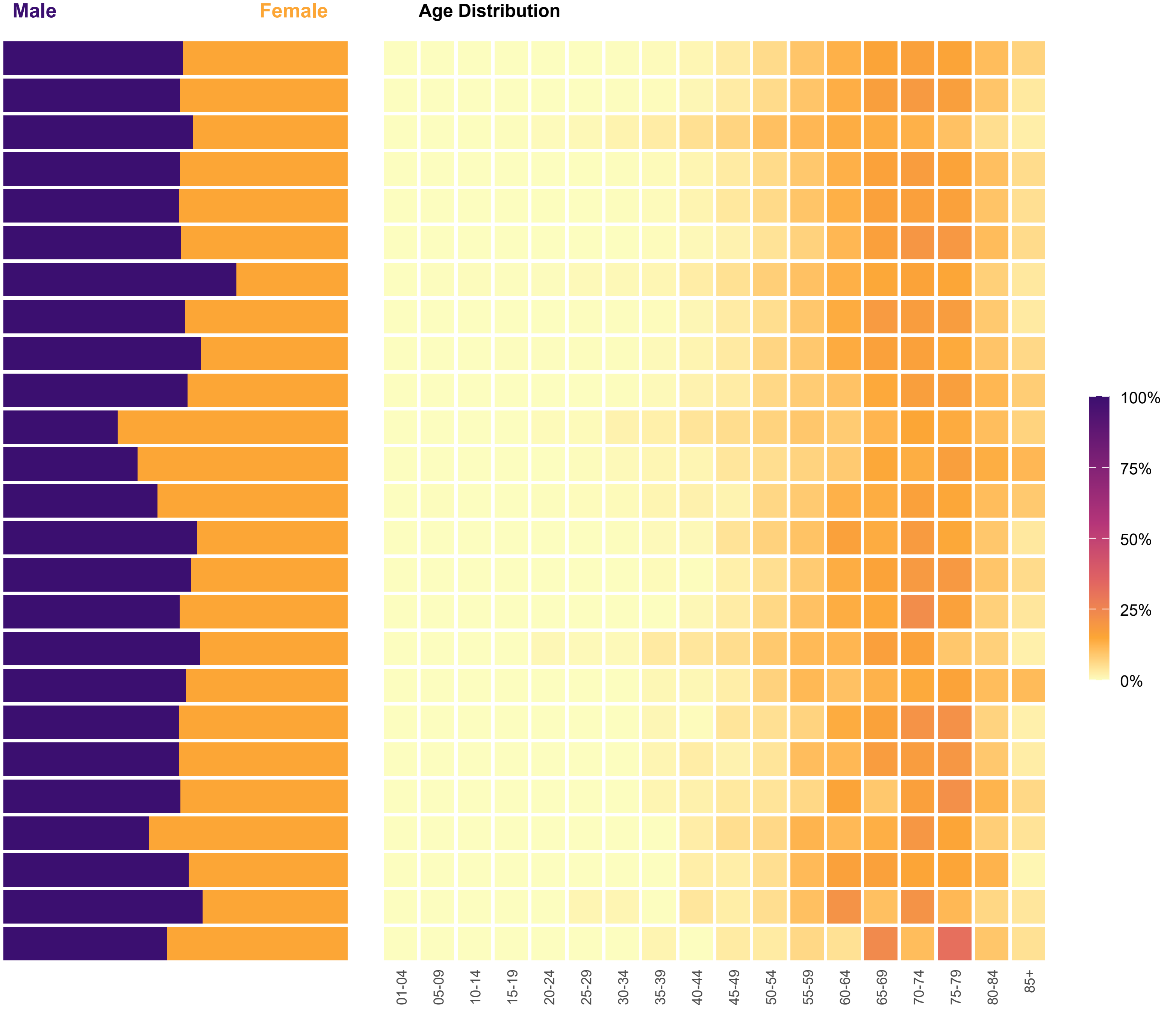

Primary Site: Pancreas | Phenotype: Grouped Phenotypes

Top 24 Morphologies | cases: 22,046

Morphology	n	%
Neuroendocrine tumor	11,153	50.6%
Neuroendocrine tumor grade 2	2,852	12.9%
Pancreatic neuroendocrine tumor…	1,940	8.8%
Small cell carcinoma	1,217	5.5%
Solid pseudopapillary neoplasm of…	1,060	4.8%
Diffuse large B-cell lymphoma	853	3.9%
Mixed neuroendocrine…	453	2.1%
Large cell neuroendocrine carcinoma	439	2.0%
Insulinoma	345	1.6%
Large cell carcinoma	339	1.5%
Gastrinoma	208	0.9%
lymphoma non-Hodgkin	171	0.8%
Pancreatoblastoma	107	0.5%
Follicular lymphoma	106	0.5%
Mixed adenoneuroendocrine carcinoma	96	0.4%
Glucagonoma	92	0.4%
Carcinosarcoma	79	0.4%
lymphoma	70	0.3%
Follicular lymphoma grade 1/2	70	0.3%
Vipoma	33	0.1%
Burkitt lymphoma	31	0.1%
Follicular lymphoma grade 3	28	0.1%
CNS embryonal tumor	27	0.1%
Marginal zone B-cell lymphoma	25	0.1%

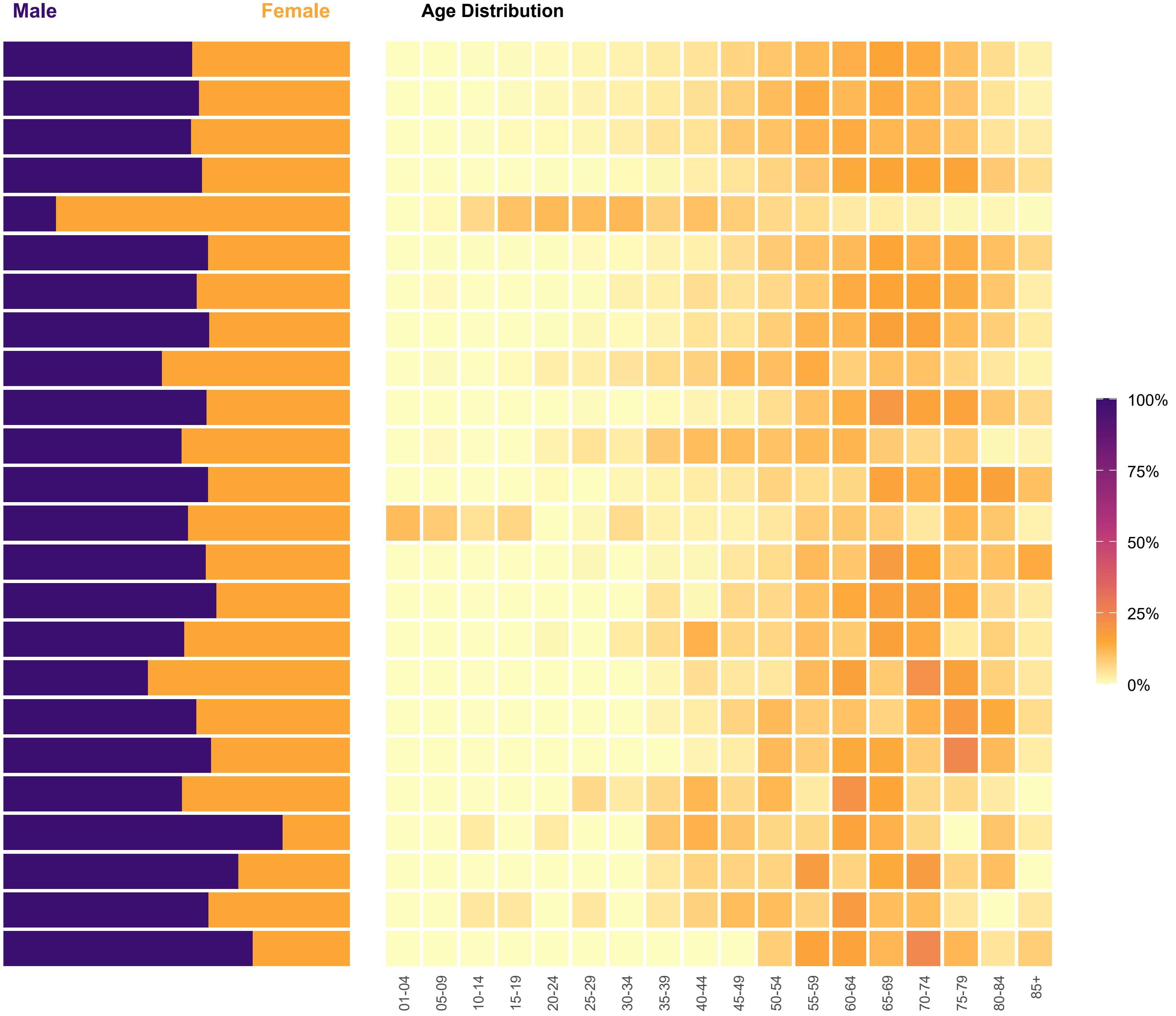

Primary Site: Parathyroid gland | Phenotype: epithelial

Top 1 Morphologies | cases: 183

Morphology	n	%
Adenocarcinoma	106	57.9%

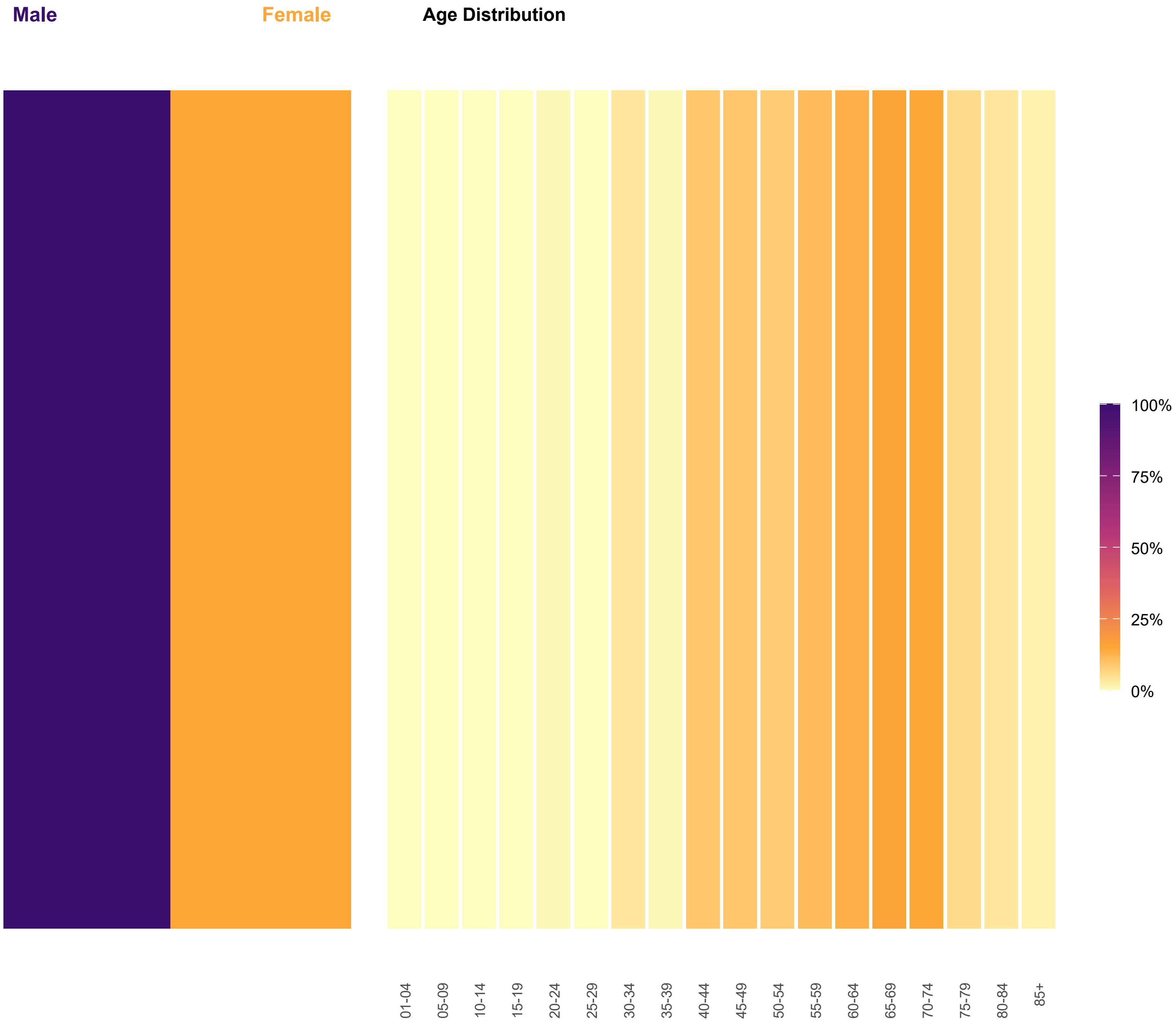

Primary Site: Pelvic bones and sacrum and coccyx and associated joints and pelvic bones | Phe

Top 9 Morphologies | cases: 3,108

Morphology	n	%
Chordoma	1,192	38.4%
Plasmacytoma	973	31.3%
Diffuse large B-cell lymphoma	457	14.7%
Langerhans cell histiocytosis…	112	3.6%
lymphoma non-Hodgkin	92	3.0%
Chondroid chordoma	31	1.0%
Follicular lymphoma	26	0.8%
Follicular lymphoma grade 1/2	21	0.7%
CNS embryonal tumor	20	0.6%

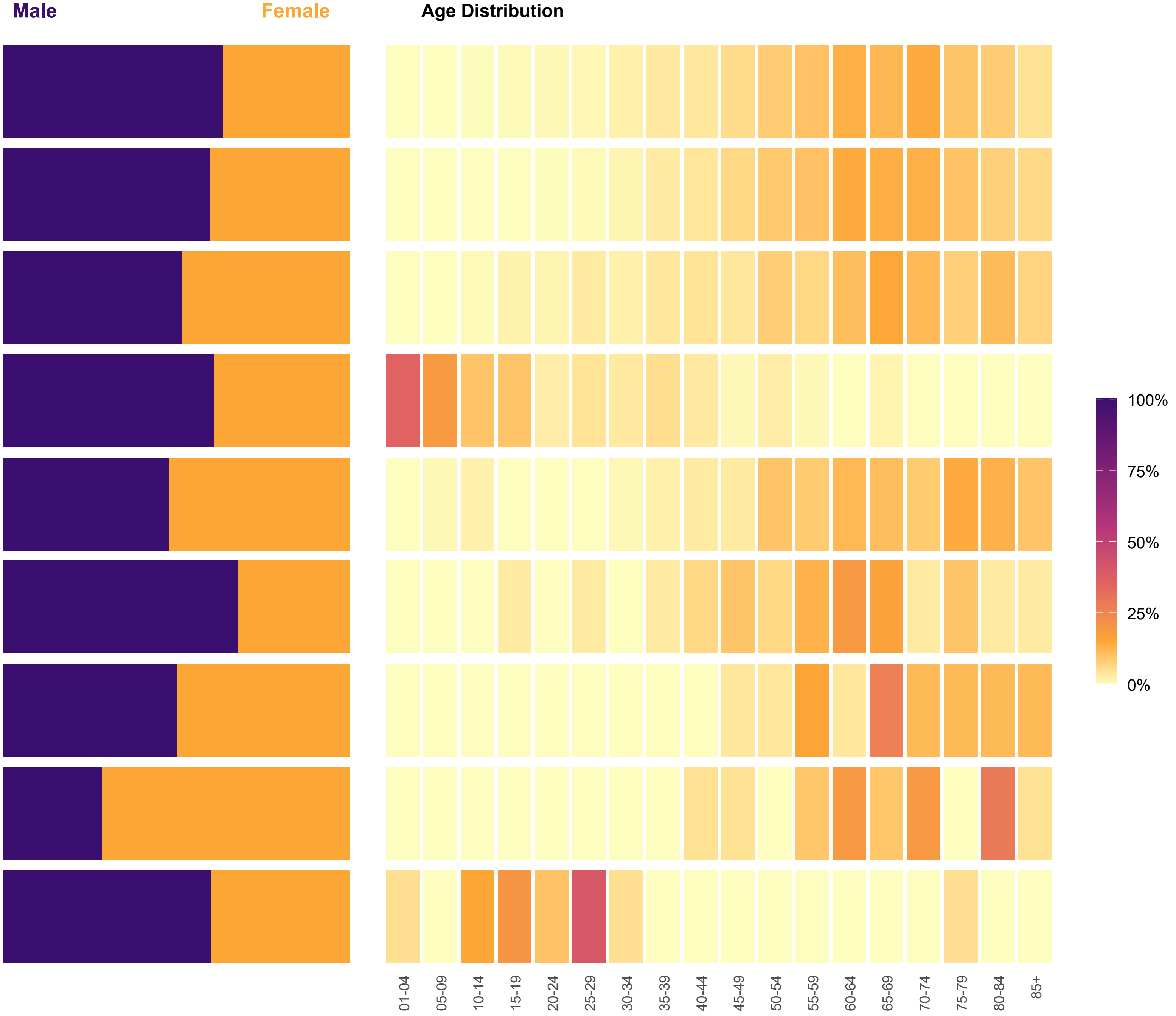

Primary Site: Penis | Phenotype: epithelial

Top 9 Morphologies | cases: 20,718

Morphology	n	%
Squamous cell carcinoma	19,661	94.9%
Basaloid squamous cell carcinoma	400	1.9%
Basal cell carcinoma	184	0.9%
Paget disease extramammary	130	0.6%
Papillary squamous cell carcinoma	90	0.4%
Adenocarcinoma	82	0.4%
Transitional cell carcinoma	38	0.2%
Basosquamous carcinoma	32	0.2%
Warty carcinoma	28	0.1%

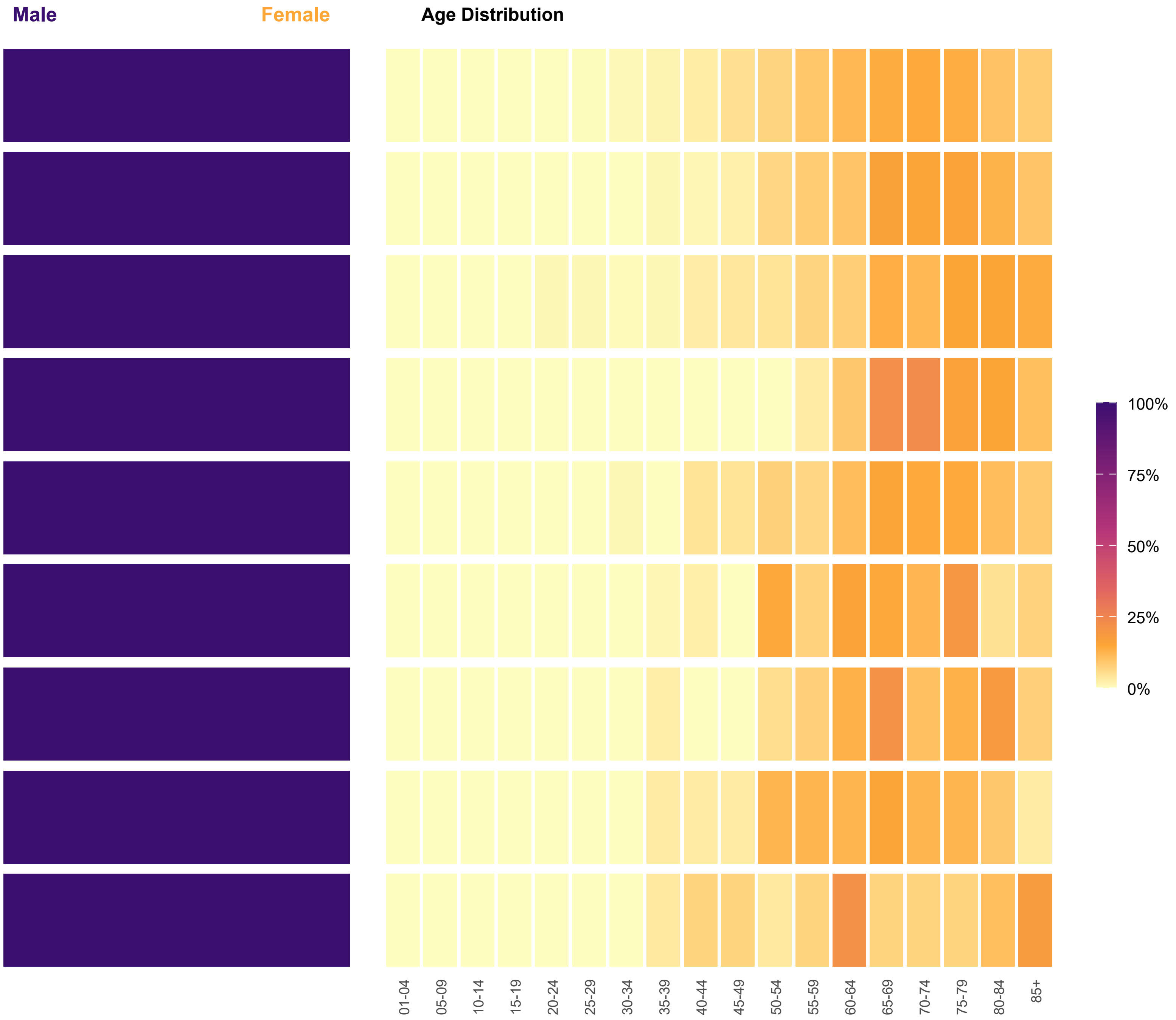

Primary Site: Penis | Phenotype: Grouped Phenotypes

Top 5 Morphologies | cases: 573

Morphology	n	%
Kaposi sarcoma	139	24.3%
Malignant melanoma	137	23.9%
Leiomyosarcoma	44	7.7%
Nodular melanoma	43	7.5%
Low cumulative sun damage melanoma	40	7.0%

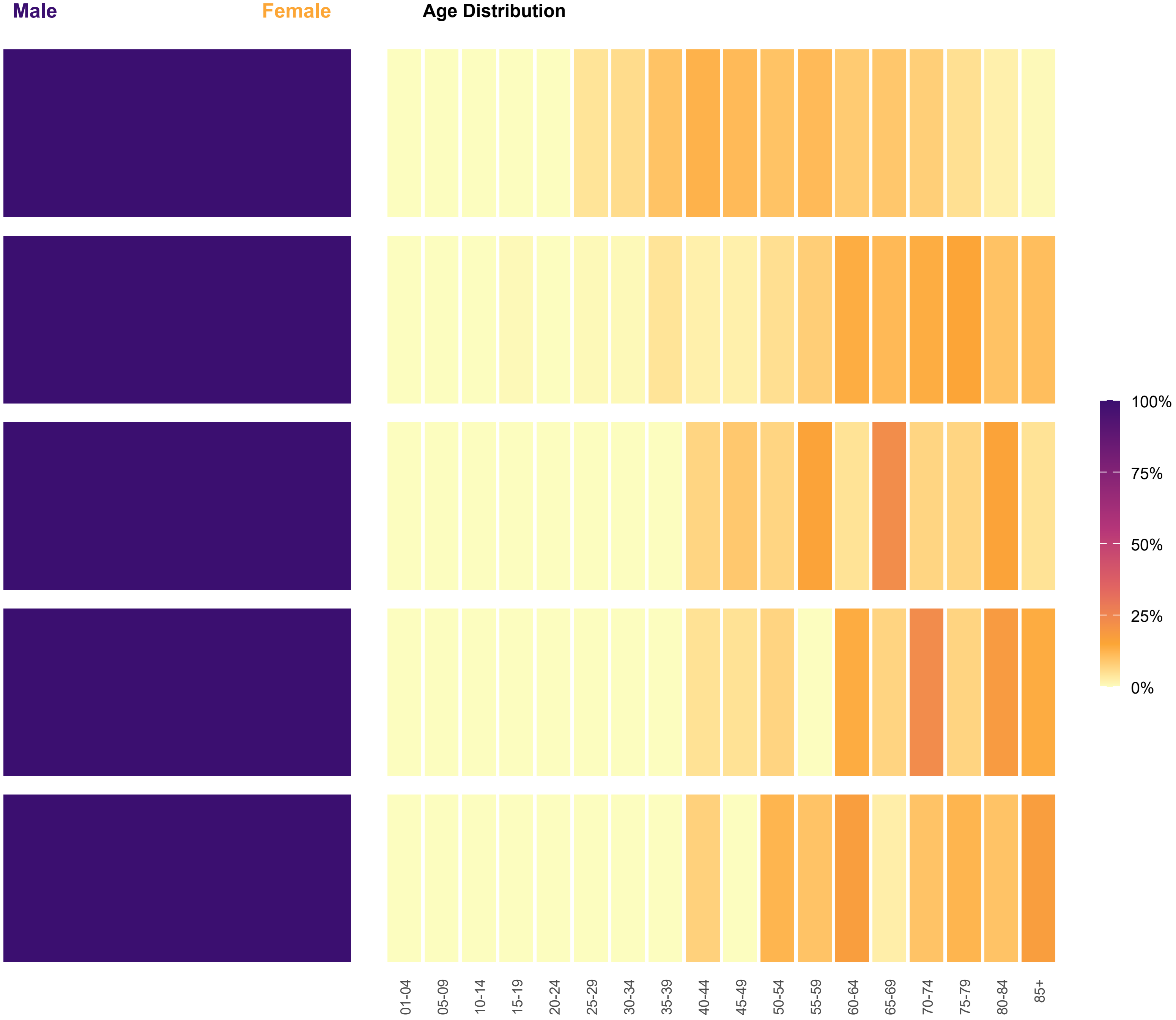

Primary Site: Peripheral nerves and autonomic nervous system | Phenotype: epithelial

Top 9 Morphologies | cases: 1,332

Morphology	n	%
Squamous cell carcinoma	683	51.3%
Neuroendocrine carcinoma	138	10.4%
Adenocarcinoma	83	6.2%
Merkel cell carcinoma	63	4.7%
Mucinous adenocarcinoma	35	2.6%
Adenoid cystic carcinoma	33	2.5%
Papillary adenocarcinoma	33	2.5%
Mucoepidermoid carcinoma	23	1.7%
Clear cell adenocarcinoma	20	1.5%

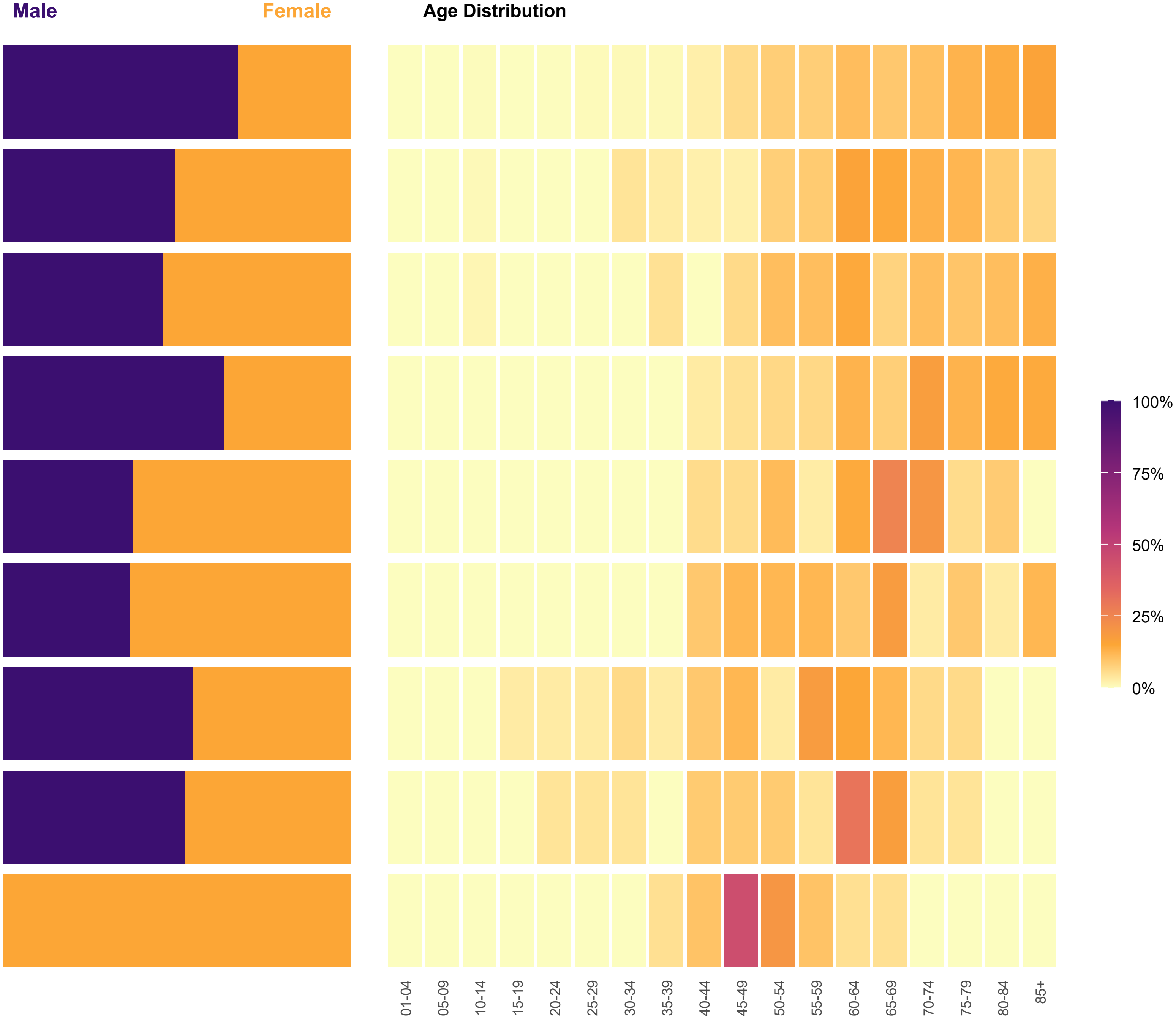

Primary Site: Peripheral nerves and autonomic nervous system | Phenotype: Grouped Phenotype

Top 25 Morphologies | cases: 9,824

Morphology	n	%
Malignant peripheral nerve sheath…	4,752	48.4%
Neuroblastoma	1,281	13.0%
Neurilemomamalignant	525	5.3%
Alveolar soft part sarcoma	518	5.3%
Ganglioneuroblastoma	421	4.3%
CNS embryonal tumor	266	2.7%
Malignant peripheral nerve sheath…	241	2.5%
Myoepithelial carcinoma	192	2.0%
Chordoma	182	1.9%
Carcinosarcoma	170	1.7%
Rhabdoid tumor	168	1.7%
Mesenchymoma	168	1.7%
Paraganglioma	132	1.3%
Granular cell tumor malignant	119	1.2%
Teratoma	86	0.9%
Yolk sac tumor	84	0.9%
Neuroendocrine tumor	66	0.7%
Neuroepithelioma	41	0.4%
Mixed germ cell tumor	40	0.4%
Small cell carcinoma	35	0.4%
Askin tumor	31	0.3%
Perineuriomamalignant	30	0.3%
Germinoma	29	0.3%
Extra-adrenal paraganglioma	27	0.3%
Mullerian mixed tumor	25	0.3%

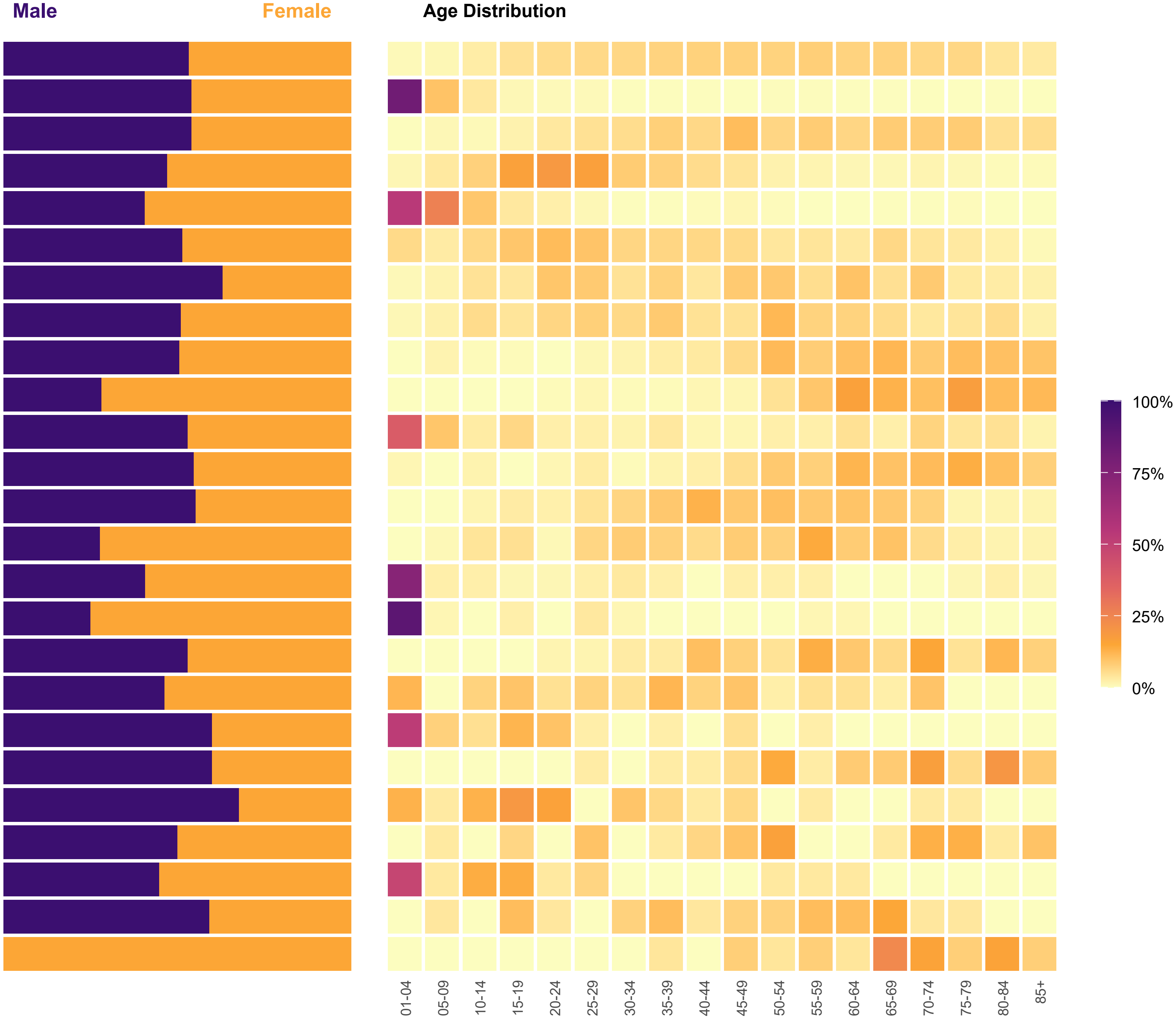

Primary Site: Pharynx and Oral Cavity Other | Phenotype: epithelial

Top 7 Morphologies | cases: 9,322

Morphology	n	%
Squamous cell carcinoma	8,824	94.7%
Basaloid squamous cell carcinoma	208	2.2%
Adenocarcinoma	77	0.8%
Lymphoepithelial carcinoma	46	0.5%
Adenoid cystic carcinoma	44	0.5%
Papillary squamous cell carcinoma	33	0.4%
Mucoepidermoid carcinoma	29	0.3%

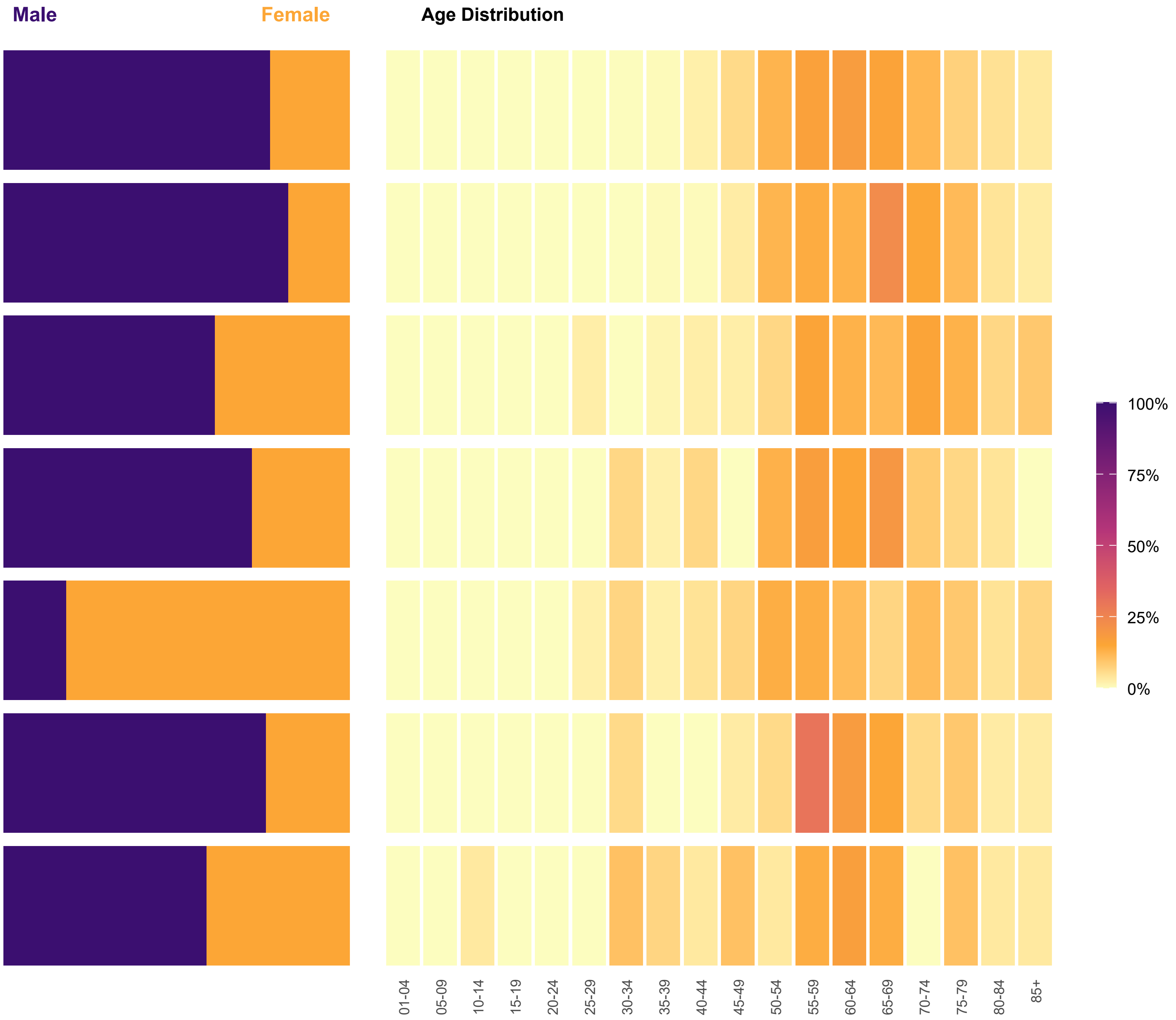

Primary Site: Pharynx and Oral Cavity Other | Phenotype: Grouped Phenotypes

Top 5 Morphologies | cases: 627

Morphology	n	%
Diffuse large B-cell lymphoma	229	36.5%
lymphoma non-Hodgkin	39	6.2%
Mantle cell lymphoma	33	5.3%
Marginal zone B-cell lymphoma	32	5.1%
Plasmacytoma extramedullary	30	4.8%

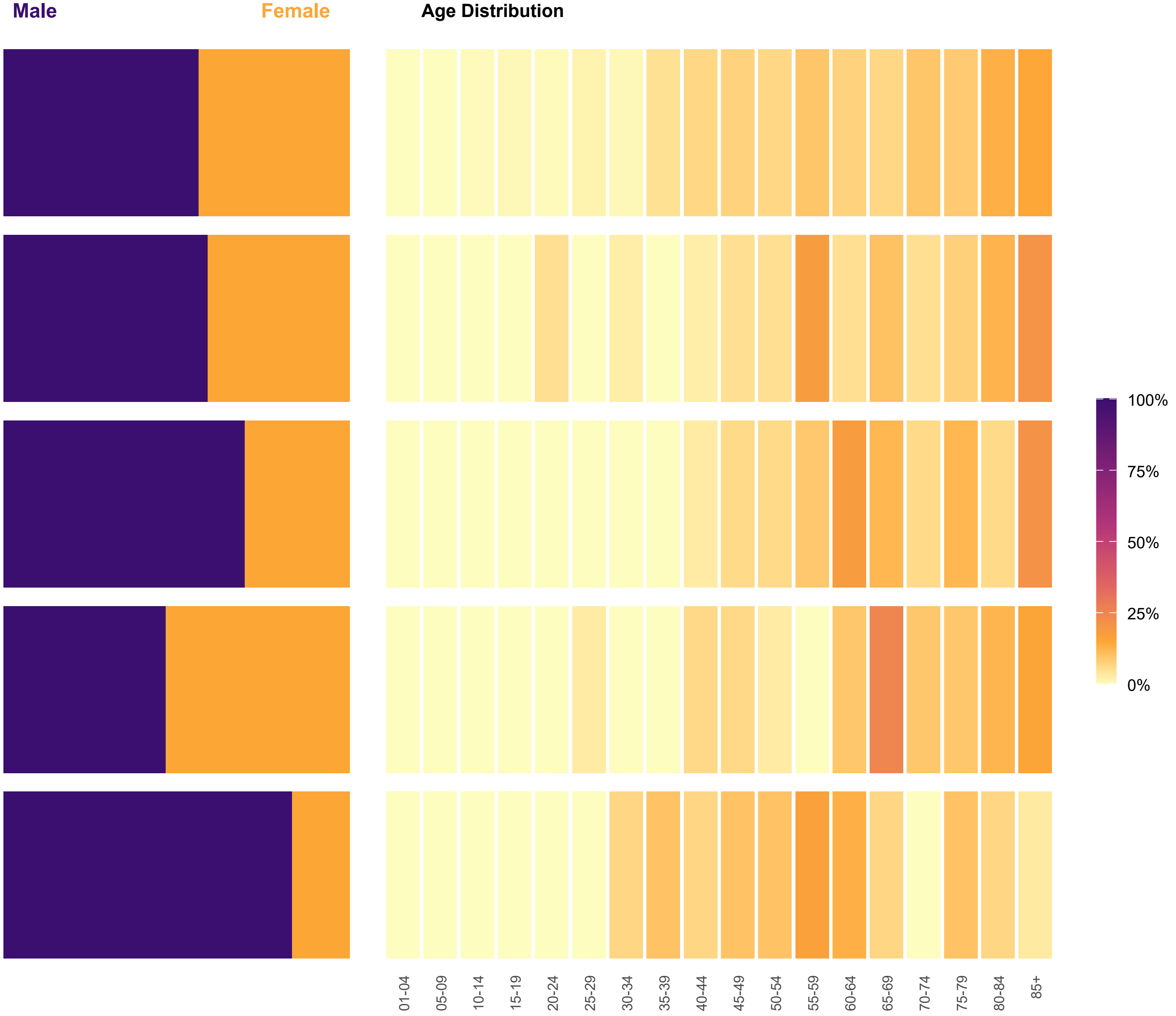

Primary Site: Placenta | Phenotype: Grouped Phenotypes

Top 2 Morphologies | cases: 1,365

Morphology	n	%
Choriocarcinoma	1,225	89.7%
Trophoblastic tumor epithelioid	128	9.4%

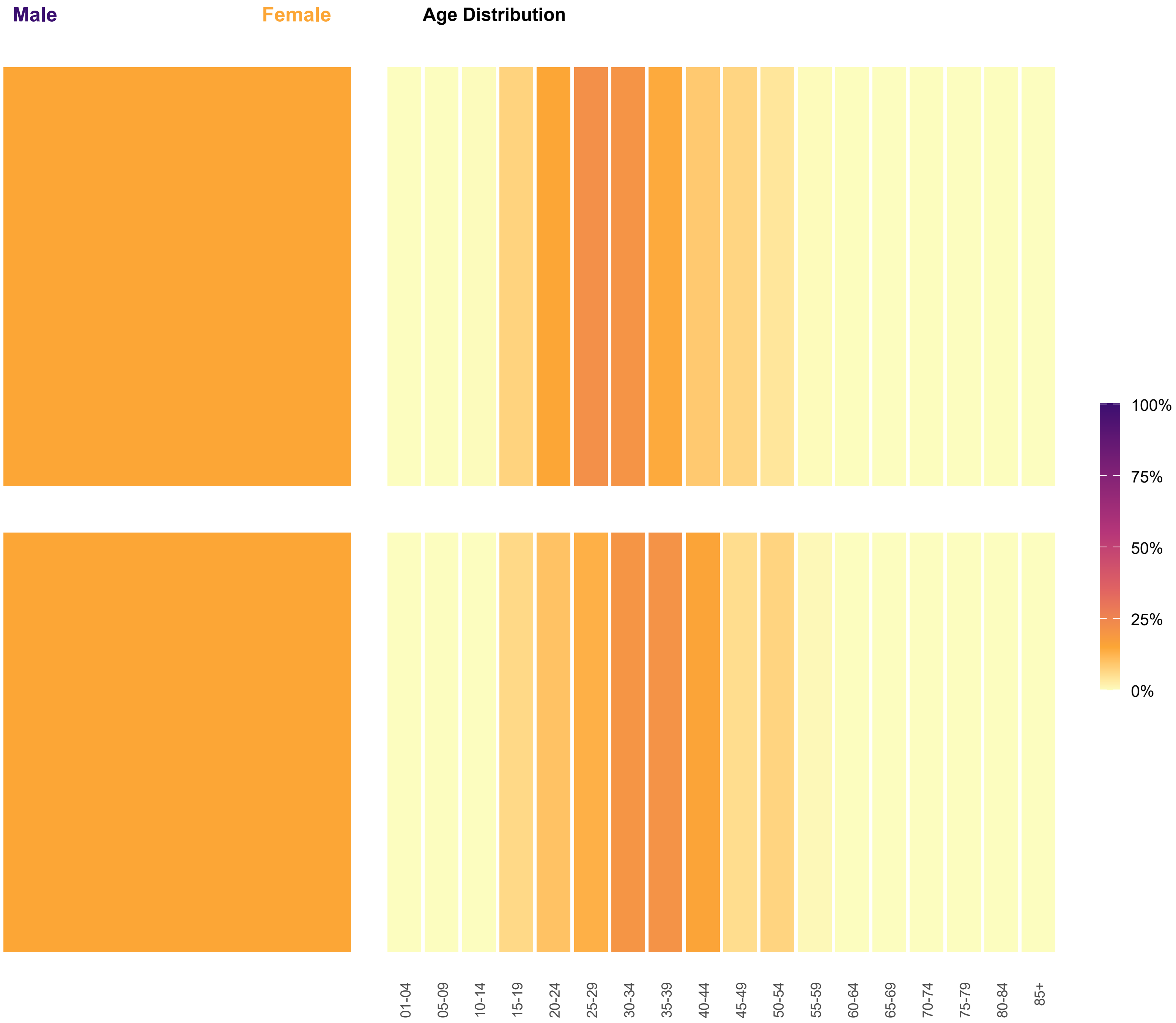

Primary Site: Prostate | Phenotype: epithelial

Top 25 Morphologies | cases: 2,593,280

Morphology	n	%
Adenocarcinoma	2,412,651	93.0%
Acinar cell carcinoma	136,988	5.3%
Tubular adenocarcinoma	15,520	0.6%
Cribriform carcinoma	12,167	0.5%
Infiltrating duct carcinoma	4,412	0.2%
Clear cell adenocarcinoma	3,450	0.1%
Mucinous adenocarcinoma	1,474	<0.1%
Adenocarcinoma with neuroendocrine…	1,189	<0.1%
Adenocarcinoma with mixed subtypes	1,157	<0.1%
Transitional cell carcinoma	902	<0.1%
Neuroendocrine carcinoma	842	<0.1%
Squamous cell carcinoma	314	<0.1%
Signet ring cell carcinoma	309	<0.1%
Papillary adenocarcinoma	288	<0.1%
Mucin-producing adenocarcinoma	277	<0.1%
Trabecular adenocarcinoma	258	<0.1%
Papillary transitional cell…	196	<0.1%
Mixed acinar-ductal carcinoma	132	<0.1%
Scirrhous adenocarcinoma	89	<0.1%
Basal cell adenocarcinoma	70	<0.1%
Endometrioid adenocarcinoma	70	<0.1%
Mixed cell adenocarcinoma	62	<0.1%
Superficial spreading…	54	<0.1%
Infiltrating duct mixed with other…	54	<0.1%
Intraductal papillary…	40	<0.1%

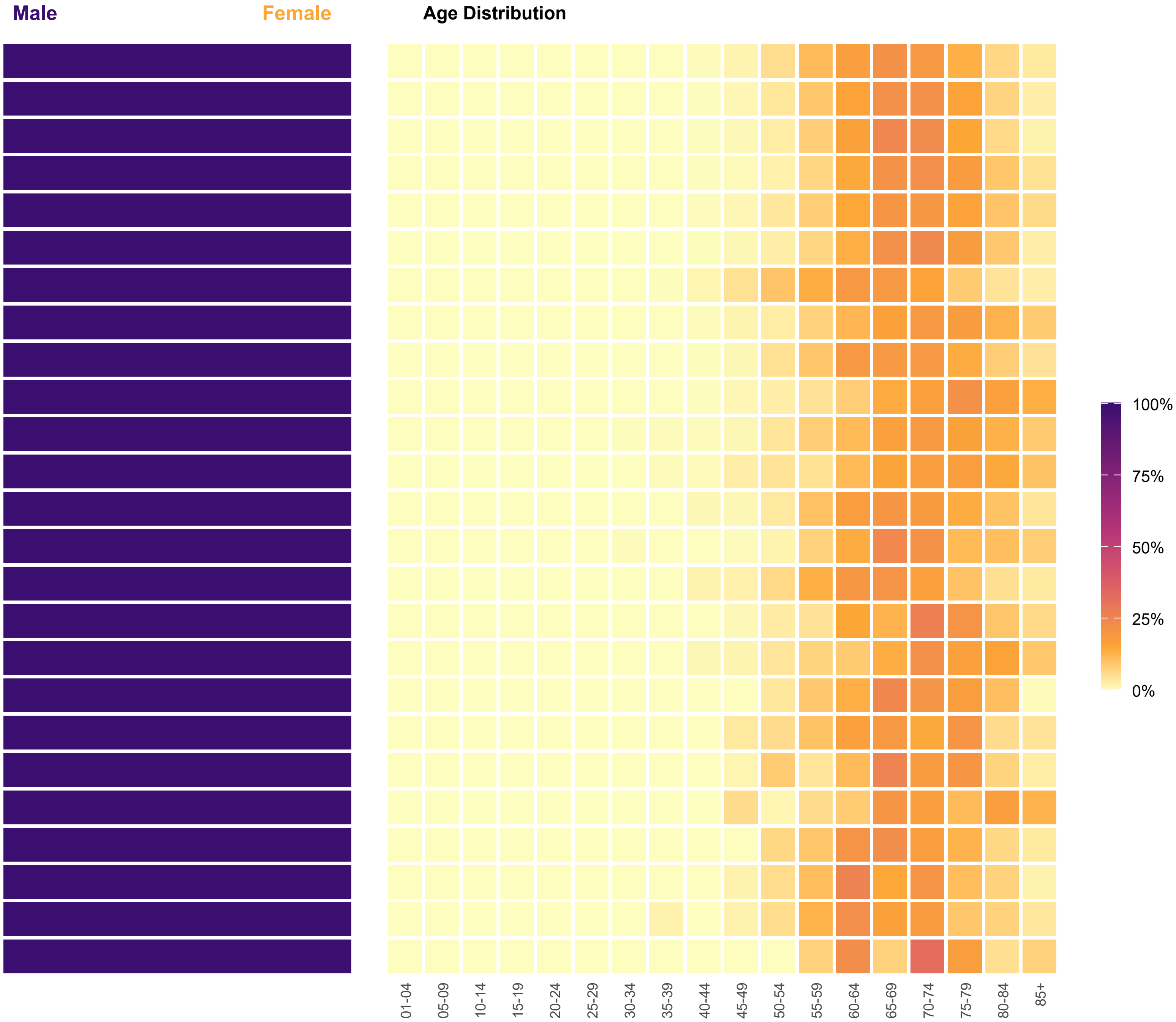

Primary Site: Prostate | Phenotype: Grouped Phenotypes

Top 17 Morphologies | cases: 3,016

Morphology	n	%
Small cell carcinoma	1,747	57.9%
Combined small cell carcinoma	162	5.4%
Leiomyosarcoma	133	4.4%
Large cell neuroendocrine carcinoma	96	3.2%
Carcinosarcoma	86	2.9%
Diffuse large B-cell lymphoma	77	2.6%
Embryonal rhabdomyosarcoma	62	2.1%
Marginal zone B-cell lymphoma	54	1.8%
lymphoma non-Hodgkin	53	1.8%
Sarcoma	52	1.7%
Mixed adenoneuroendocrine carcinoma	48	1.6%
Neuroendocrine tumor	45	1.5%
B-cell chronic lymphocytic…	38	1.3%
Large cell carcinoma	35	1.2%
Stromal sarcoma	34	1.1%
Large cell carcinoma with rhabdoid…	27	0.9%
Rhabdomyosarcoma	22	0.7%

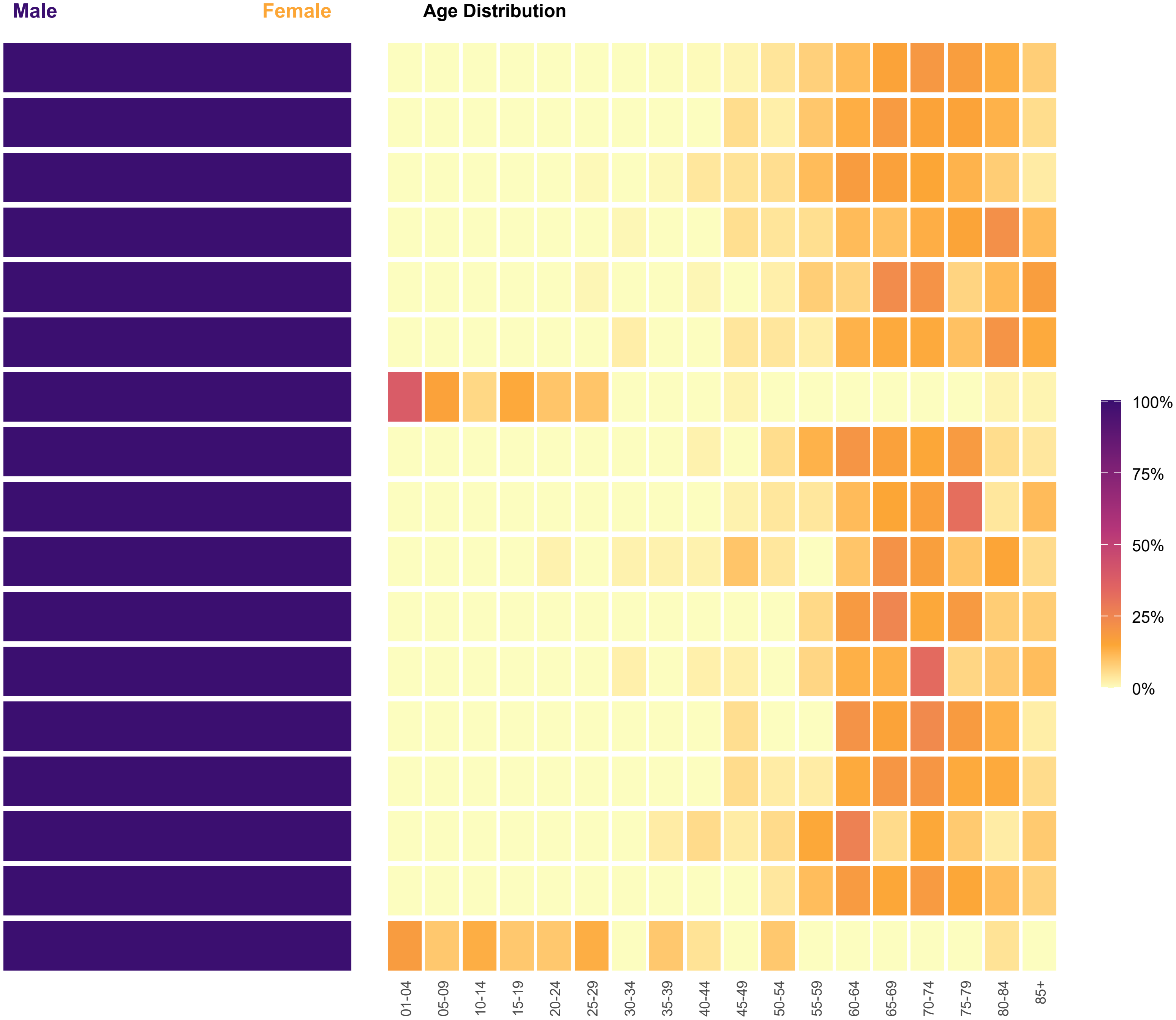

Primary Site: Retroperitoneum and Peritoneum | Phenotype: epithelial

Top 16 Morphologies | cases: 16,365

Morphology	n	%
High grade serous carcinoma	6,088	37.2%
Serous carcinoma	4,344	26.5%
Low grade serous carcinoma	2,210	13.5%
Adenocarcinoma	2,106	12.9%
Mucinous adenocarcinoma	266	1.6%
Endometrioid adenocarcinoma	263	1.6%
Papillary adenocarcinoma	254	1.6%
Neuroendocrine carcinoma	249	1.5%
Clear cell adenocarcinoma	150	0.9%
Mixed cell adenocarcinoma	124	0.8%
Cystadenocarcinoma	48	0.3%
Mucin-producing adenocarcinoma	46	0.3%
Signet ring cell carcinoma	45	0.3%
Mucinous cystadenocarcinoma	39	0.2%
Squamous cell carcinoma	34	0.2%
Adenocarcinoma with mixed subtypes	29	0.2%

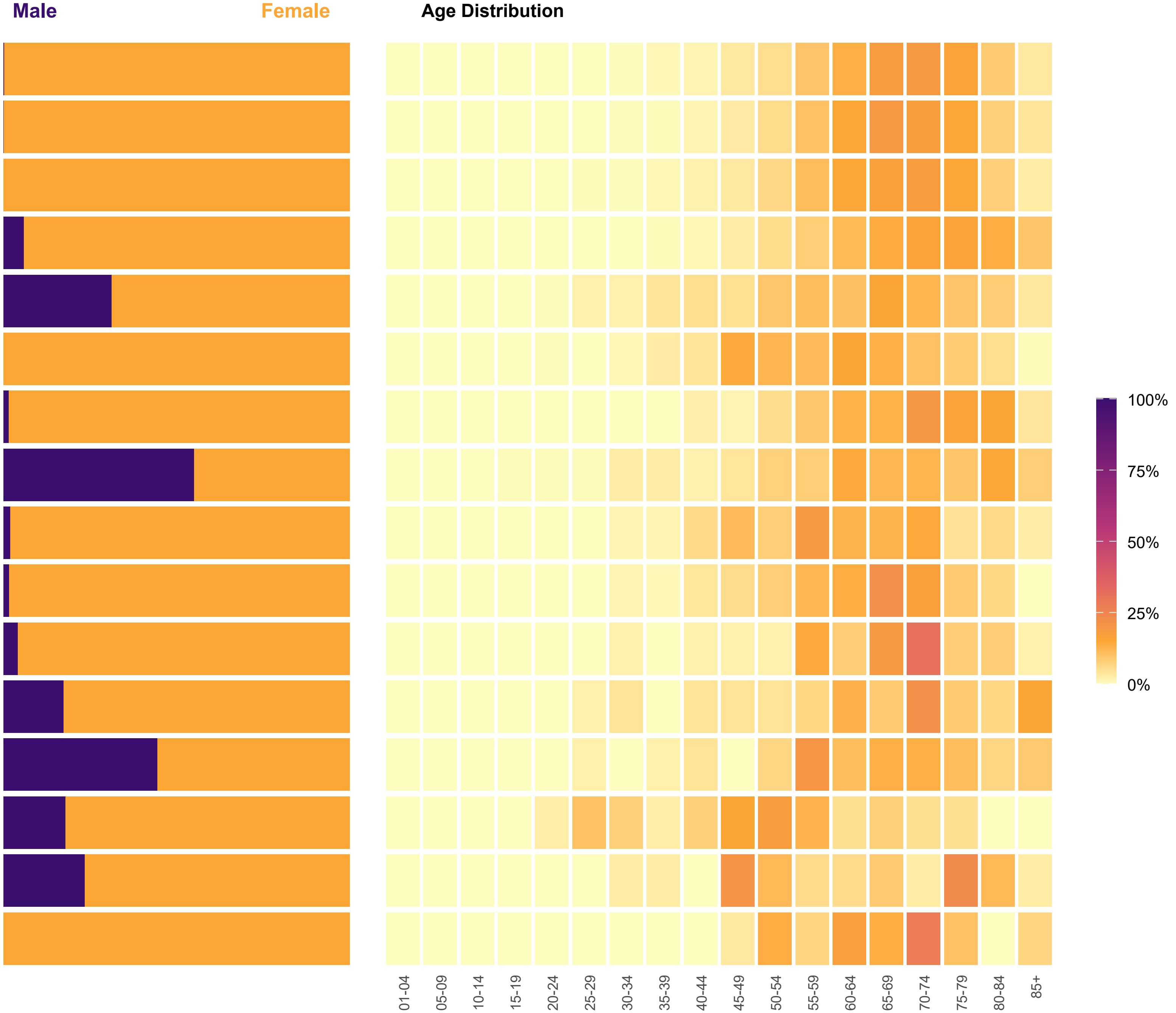

Primary Site: Retroperitoneum and Peritoneum | Phenotype: Grouped Phenotypes

Top 19 Morphologies | cases: 2,666

Morphology	n	%
Neuroendocrine tumor	452	17.0%
Neuroblastoma	417	15.6%
Mullerian mixed tumor	271	10.2%
Carcinosarcoma	181	6.8%
Seminoma	152	5.7%
Malignant peripheral nerve sheath…	146	5.5%
Ganglioneuroblastoma	144	5.4%
Germinoma	127	4.8%
Paraganglioma	120	4.5%
Yolk sac tumor	72	2.7%
Embryonal carcinoma	54	2.0%
Extra-adrenal paraganglioma	53	2.0%
Teratoma	53	2.0%
Germ cell tumor nonseminomatous	48	1.8%
Small cell carcinoma	45	1.7%
Neurilemomamalignant	37	1.4%
Mixed germ cell tumor	32	1.2%
CNS embryonal tumor	27	1.0%
Choriocarcinoma	21	0.8%

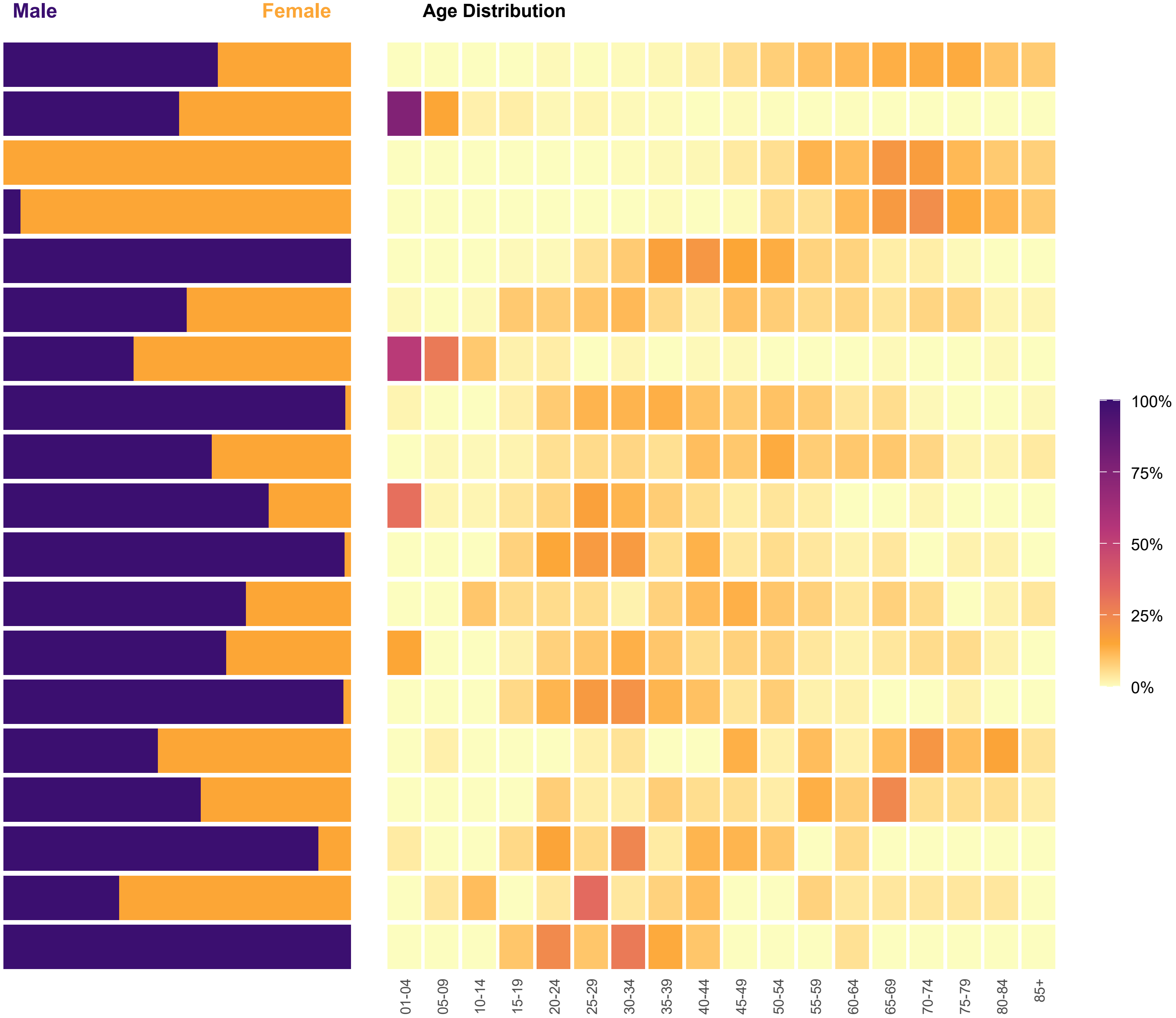

Primary Site: Rib and sternum and clavicle and associated joints | Phenotype: Grouped Phenotyp

Top 12 Morphologies | cases: 3,484

Morphology	n	%
Chondrosarcoma	1,463	42.0%
Plasmacytoma	924	26.5%
Osteosarcoma	258	7.4%
Diffuse large B-cell lymphoma	140	4.0%
Langerhans cell histiocytosis…	91	2.6%
Myxoid chondrosarcoma	74	2.1%
Chondroblastic osteosarcoma	63	1.8%
Dedifferentiated chondrosarcoma	46	1.3%
Ewing sarcoma	42	1.2%
Sarcoma	39	1.1%
Periosteal chondrosarcoma	29	0.8%
Mesenchymal chondrosarcoma	24	0.7%

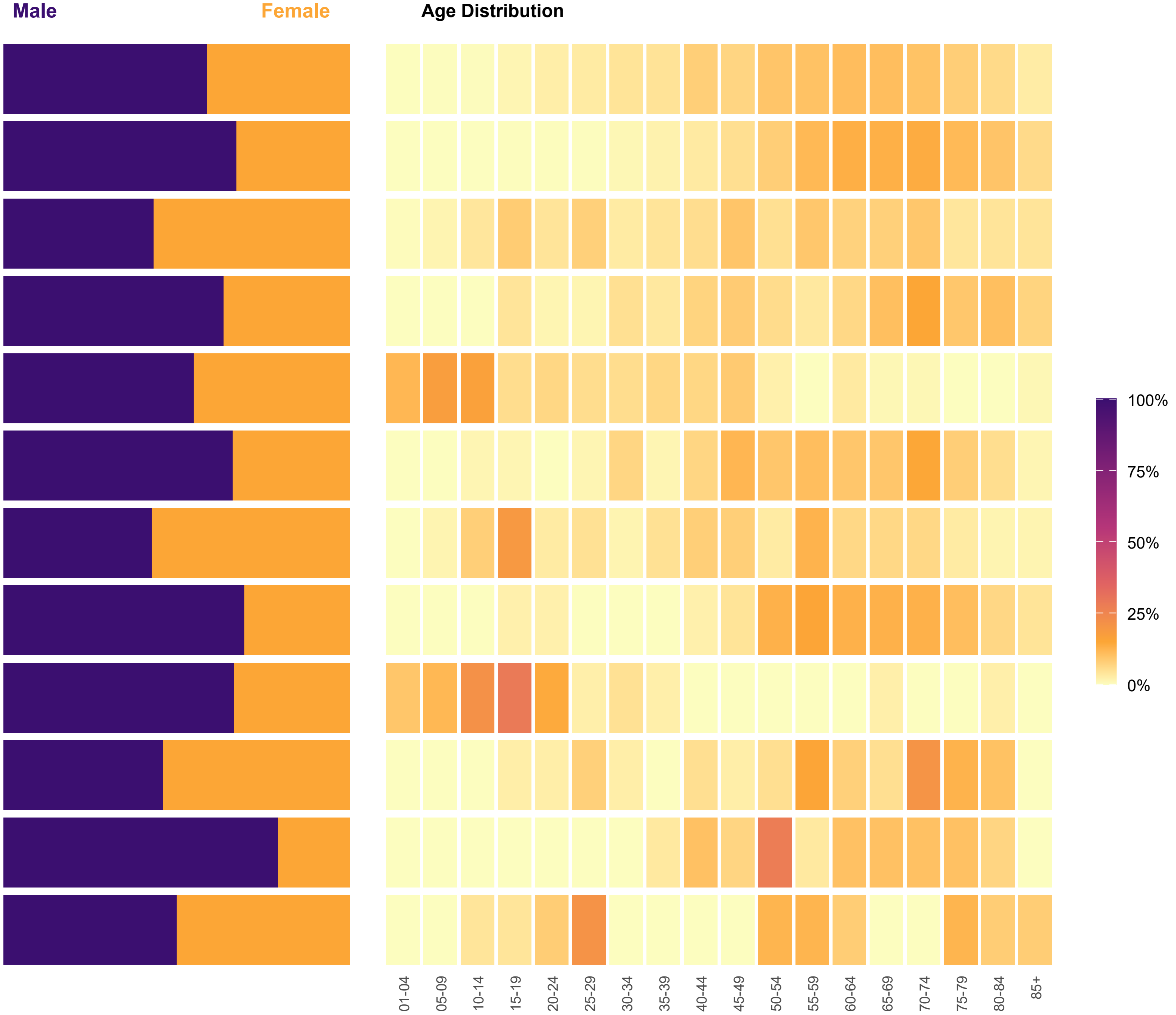

Primary Site: Short bones of lower limb and associated joints | Phenotype: Grouped Phenotypes

Top 7 Morphologies | cases: 713

Morphology	n	%
Chondrosarcoma	211	29.6%
Osteosarcoma	127	17.8%
Diffuse large B-cell lymphoma	52	7.3%
Myxoid chondrosarcoma	30	4.2%
Giant cell tumor of bone malignant	28	3.9%
Chondroblastic osteosarcoma	23	3.2%
Plasmacytoma	21	2.9%

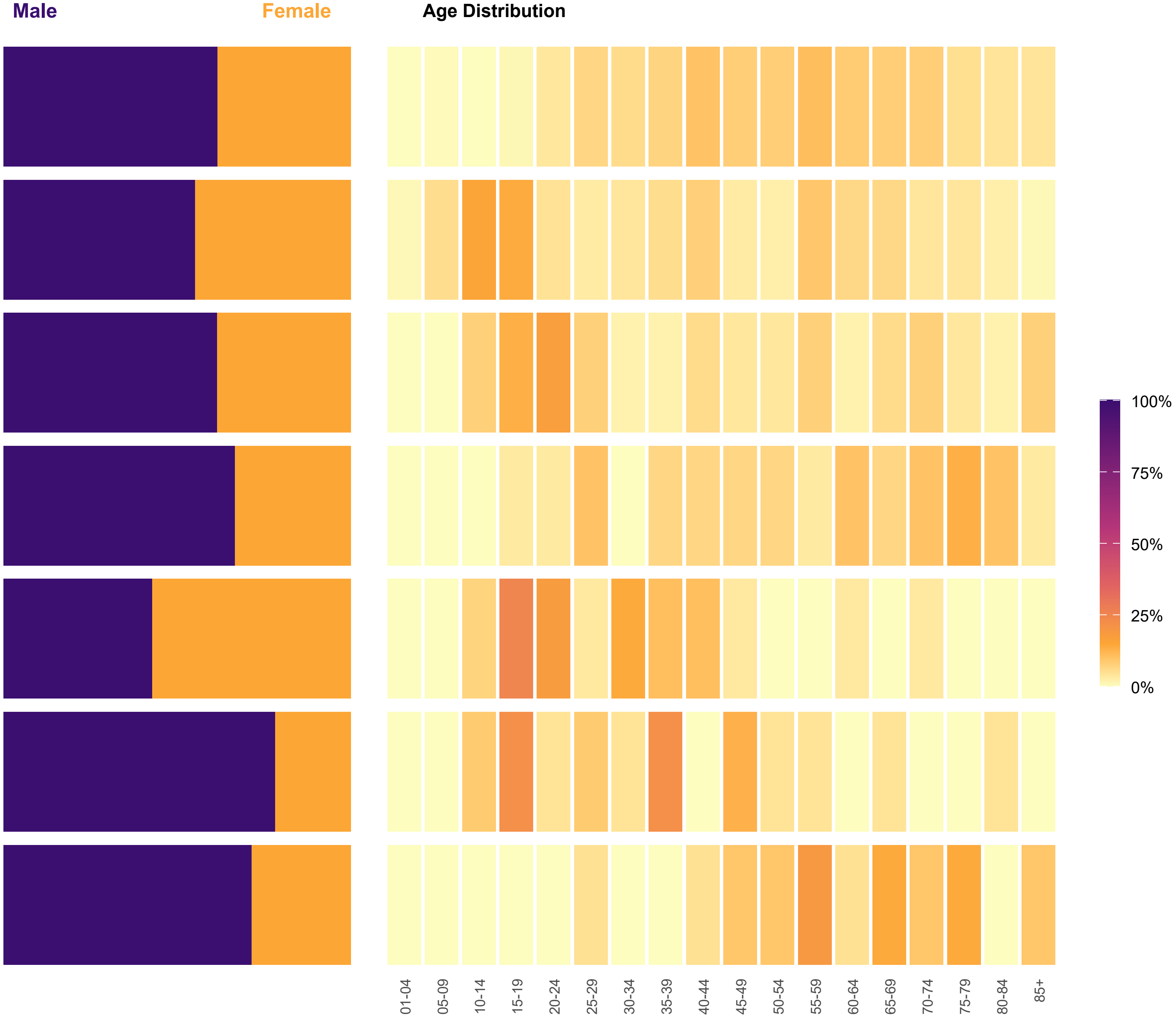

Primary Site: Short bones of upper limb and associated joints | Phenotype: Grouped Phenotypes

Top 5 Morphologies | cases: 536

Morphology	n	%
Chondrosarcoma	272	50.7%
Osteosarcoma	64	11.9%
Giant cell tumor of bone malignant	50	9.3%
Myxoid chondrosarcoma	25	4.7%
Plasmacytoma	25	4.7%

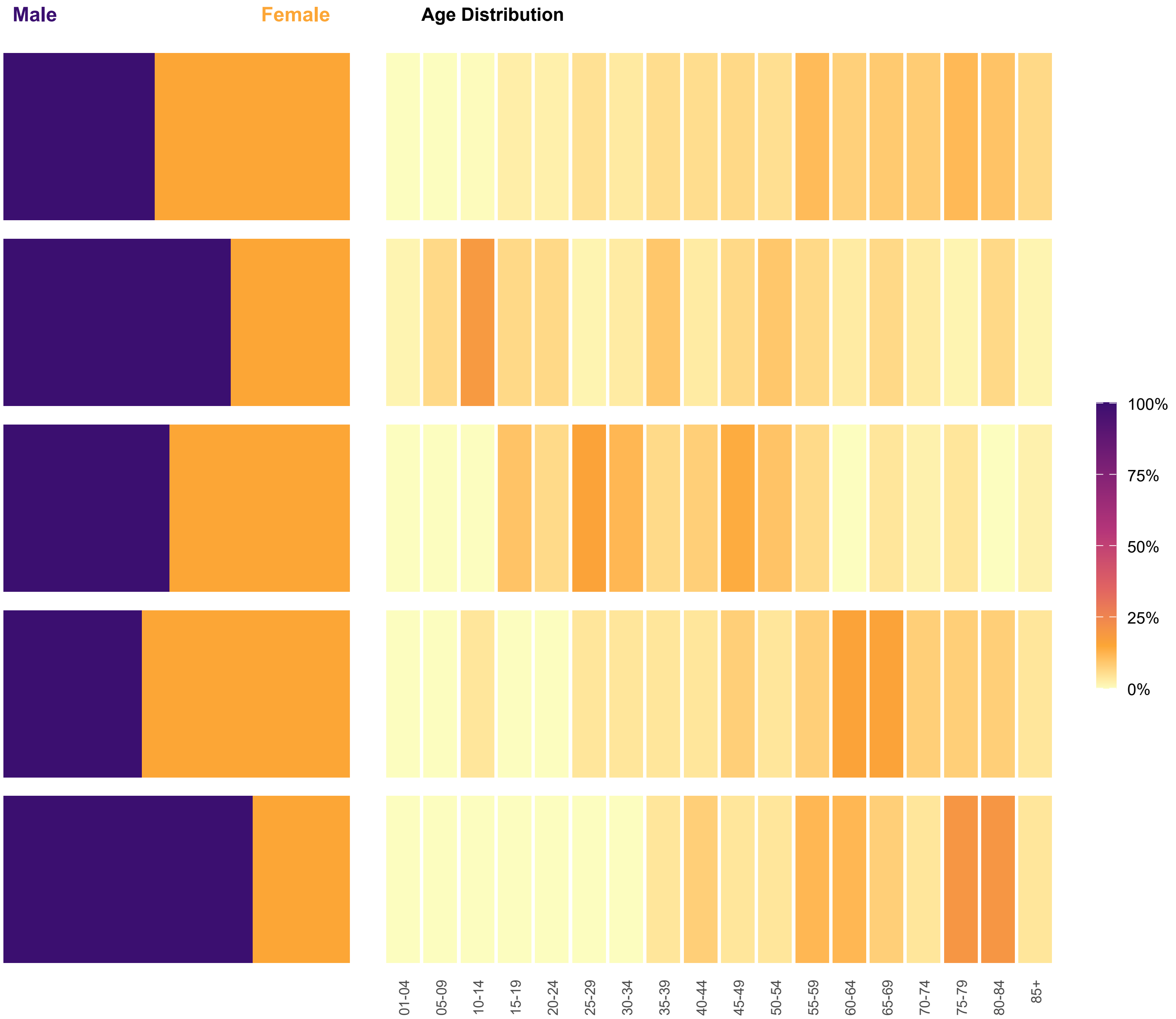

Primary Site: Sinus Other | Phenotype: epithelial

Top 13 Morphologies | cases: 3,629

Morphology	n	%
Squamous cell carcinoma	2,186	60.2%
Adenocarcinoma	507	14.0%
Adenoid cystic carcinoma	290	8.0%
Neuroendocrine carcinoma	146	4.0%
Adenocarcinoma intestinal type	61	1.7%
Mucinous adenocarcinoma	60	1.7%
Schneiderian carcinoma	56	1.5%
Papillary adenocarcinoma	55	1.5%
Basaloid squamous cell carcinoma	52	1.4%
Lymphoepithelial carcinoma	32	0.9%
Mucoepidermoid carcinoma	30	0.8%
Papillary squamous cell carcinoma	29	0.8%
Transitional cell carcinoma	27	0.7%

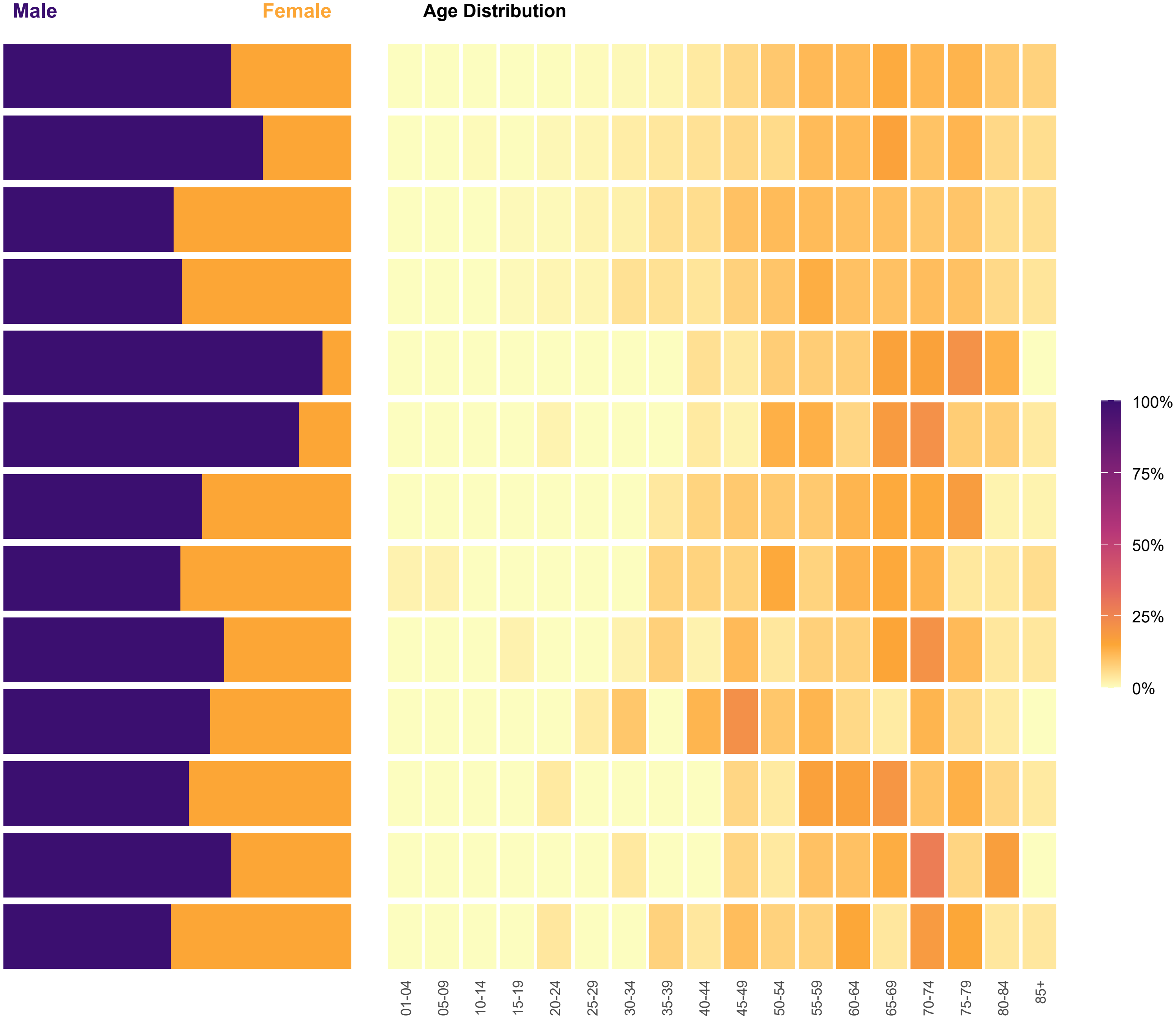

Primary Site: Sinus Other | Phenotype: Grouped Phenotypes

Top 18 Morphologies | cases: 1,798

Morphology	n	%
Diffuse large B-cell lymphoma	379	21.1%
Malignant melanoma	274	15.2%
Olfactory neuroblastoma	153	8.5%
Small cell carcinoma	75	4.2%
NK/T-cell lymphoma nasal and nasal…	75	4.2%
Alveolar rhabdomyosarcoma	71	3.9%
Embryonal rhabdomyosarcoma	58	3.2%
Neuroendocrine tumor	50	2.8%
lymphoma non-Hodgkin	44	2.4%
Plasmacytoma extramedullary	42	2.3%
Sarcoma	41	2.3%
Chordoma	41	2.3%
Amelanotic melanoma	27	1.5%
Marginal zone B-cell lymphoma	26	1.4%
Rhabdomyosarcoma	23	1.3%
Mature T-cell lymphoma	21	1.2%
lymphoma	20	1.1%
Chondrosarcoma	20	1.1%

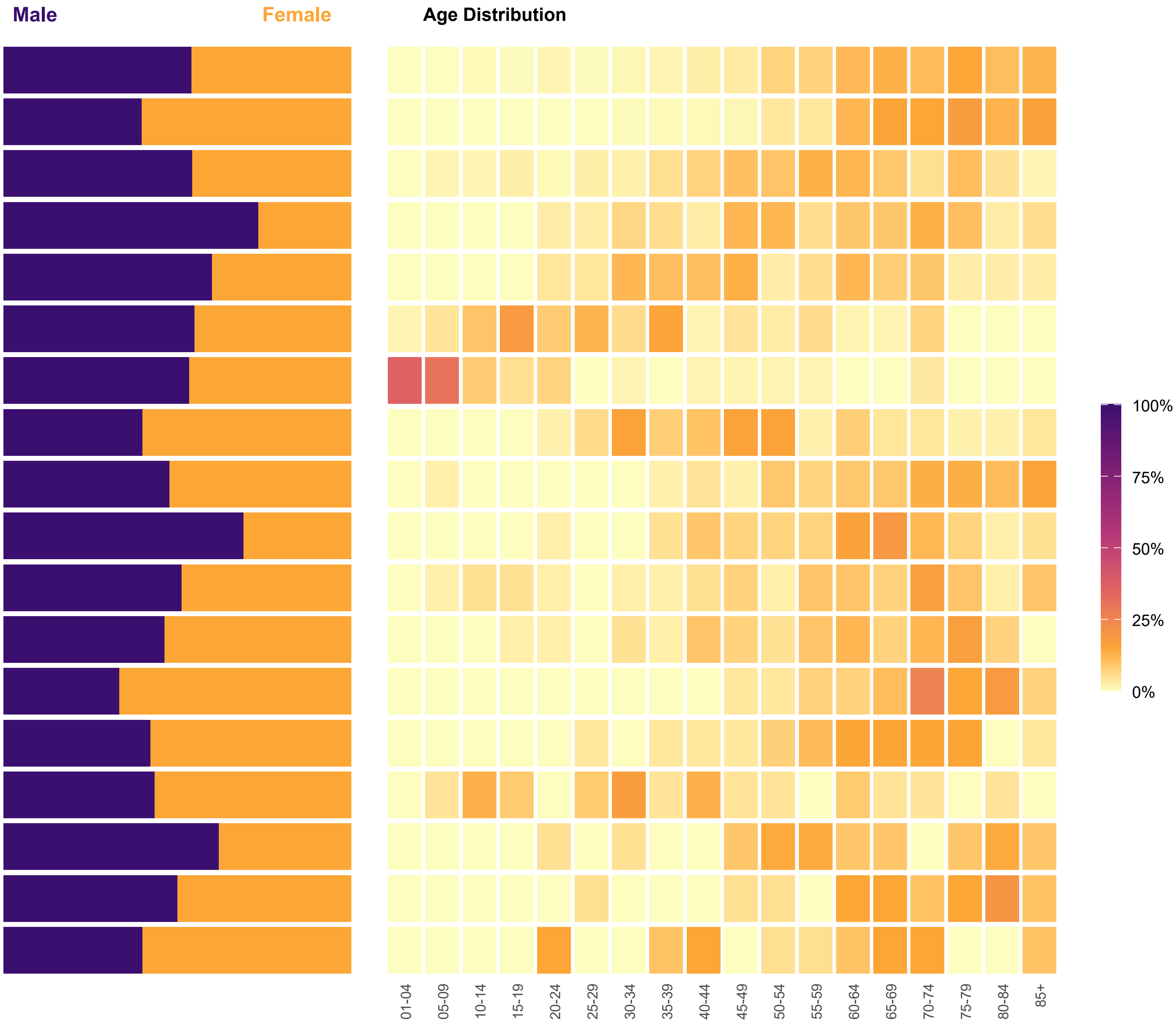

Primary Site: Skin | Phenotype: epithelial

Top 25 Morphologies | cases: 2,049,690

Morphology	n	%
Basal cell carcinoma	1,553,959	75.8%
Squamous cell carcinoma	439,360	21.4%
Merkel cell carcinoma	24,370	1.2%
Sebaceous carcinoma	7,593	0.4%
Basosquamous carcinoma	6,650	0.3%
Adnexal adenocarcinoma	2,263	0.1%
Porocarcinoma	2,184	0.1%
Microcystic adnexal carcinoma	1,523	<0.1%
Sweat gland adenocarcinoma	1,469	<0.1%
Paget disease extramammary	1,415	<0.1%
Adenocarcinoma	1,148	<0.1%
Basaloid squamous cell carcinoma	991	<0.1%
Eccrine adenocarcinoma	952	<0.1%
Mucinous adenocarcinoma	896	<0.1%
Adenoid cystic carcinoma	895	<0.1%
Hidradenocarcinoma	775	<0.1%
Apocrine adenocarcinoma	473	<0.1%
Trichilemmocarcinoma	415	<0.1%
Digital papillary adenocarcinoma	304	<0.1%
Malignant eccrine spiradenoma	248	<0.1%
Neuroendocrine carcinoma	228	<0.1%
Pilomatrical carcinoma	155	<0.1%
Mucoepidermoid carcinoma	138	<0.1%
Basaloid carcinoma	136	<0.1%
Papillary squamous cell carcinoma	131	<0.1%

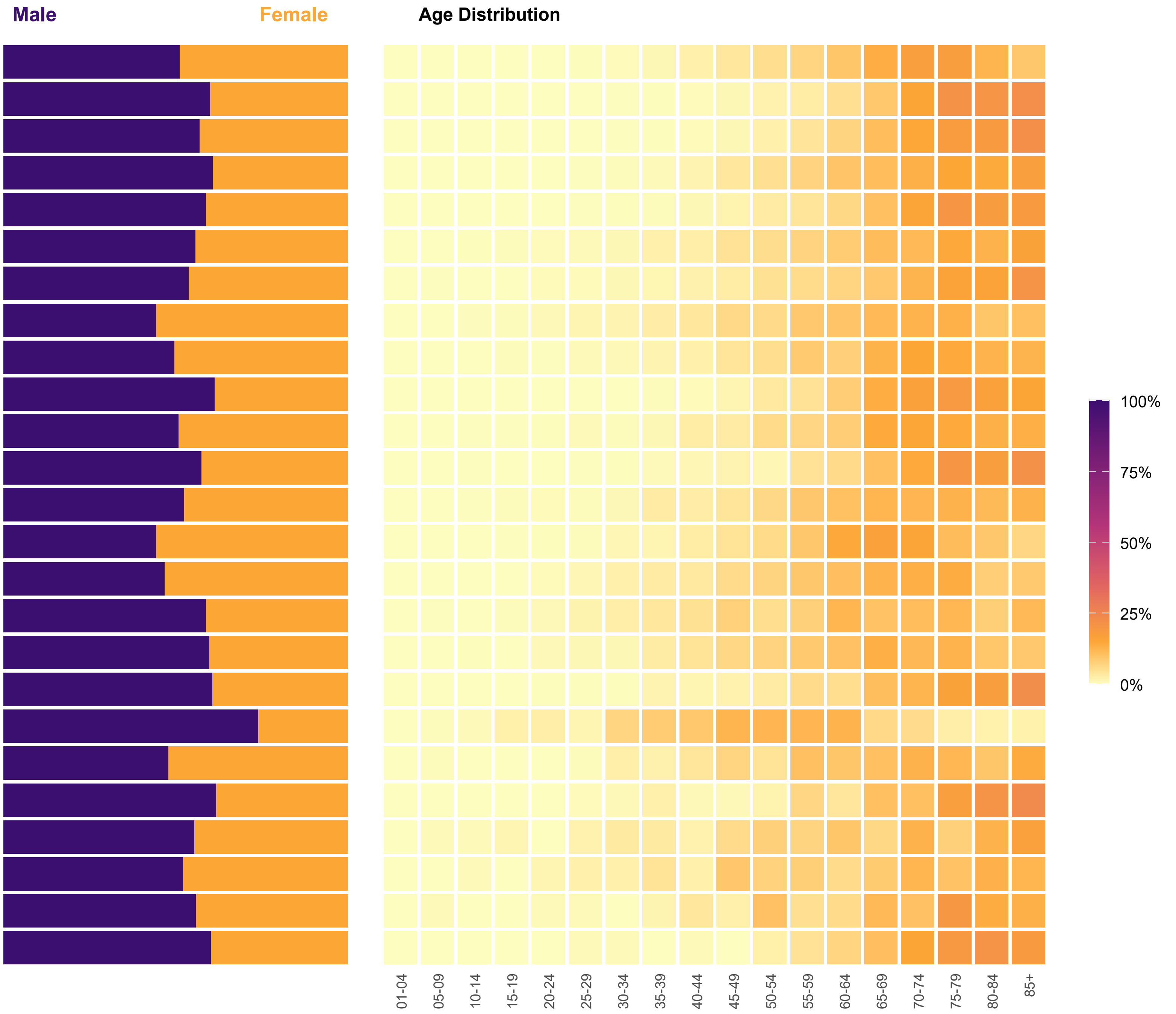

Primary Site: Skin | Phenotype: Grouped Phenotypes

Top 24 Morphologies | cases: 885,211

Morphology	n	%
Malignant melanoma	390,212	44.1%
Low cumulative sun damage melanoma	314,999	35.6%
Nodular melanoma	73,203	8.3%
Lentigo maligna melanoma	63,535	7.2%
Acral melanoma	11,891	1.3%
Desmoplastic melanoma	7,312	0.8%
Spindle cell melanoma	6,822	0.8%
Amelanotic melanoma	6,439	0.7%
Malignant melanoma regressing	2,847	0.3%
Epithelioid cell melanoma	1,940	0.2%
Malignant melanoma arising in…	1,923	0.2%
Malignant melanoma in junctional…	1,780	0.2%
Malignant Spitz tumor	1,278	0.1%
Balloon cell melanoma	199	<0.1%
Carcinosarcoma	176	<0.1%
Blue nevus malignant	172	<0.1%
Malignant melanoma in precancerous…	90	<0.1%
Myoepithelial carcinoma	66	<0.1%
Mucosal lentiginous melanoma	57	<0.1%
Small cell carcinoma	46	<0.1%
Malignant peripheral nerve sheath…	30	<0.1%
Neurilemomamalignant	26	<0.1%
Granular cell tumor malignant	25	<0.1%
Epithelial-myoepithelial carcinoma	23	<0.1%

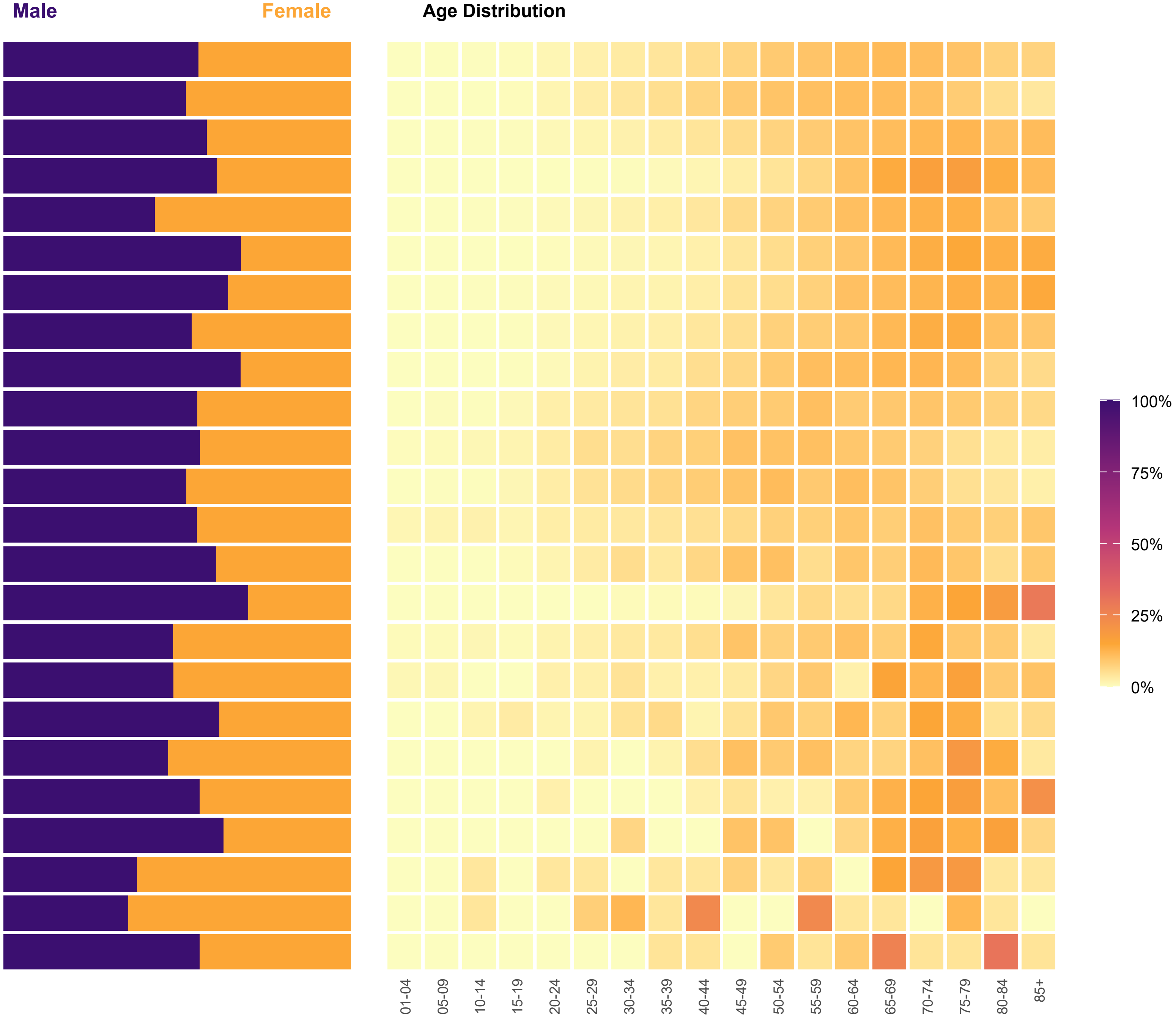

Primary Site: Small intestine | Phenotype: epithelial

Top 15 Morphologies | cases: 40,611

Morphology	n	%
Adenocarcinoma	23,236	57.2%
Neuroendocrine carcinoma	10,735	26.4%
Mucinous adenocarcinoma	1,940	4.8%
Adenocarcinoma in tubulovillous…	1,032	2.5%
Signet ring cell carcinoma	1,012	2.5%
Mucin-producing adenocarcinoma	554	1.4%
Adenocarcinoma in adenomatous polyp	439	1.1%
Adenocarcinoma in villous adenoma	371	0.9%
Adenocarcinoma intestinal type	333	0.8%
Tubular adenocarcinoma	261	0.6%
Adenocarcinoma with neuroendocrine…	171	0.4%
Adenocarcinoma with mixed subtypes	130	0.3%
Squamous cell carcinoma	121	0.3%
Papillary adenocarcinoma	113	0.3%
Villous adenocarcinoma	20	<0.1%

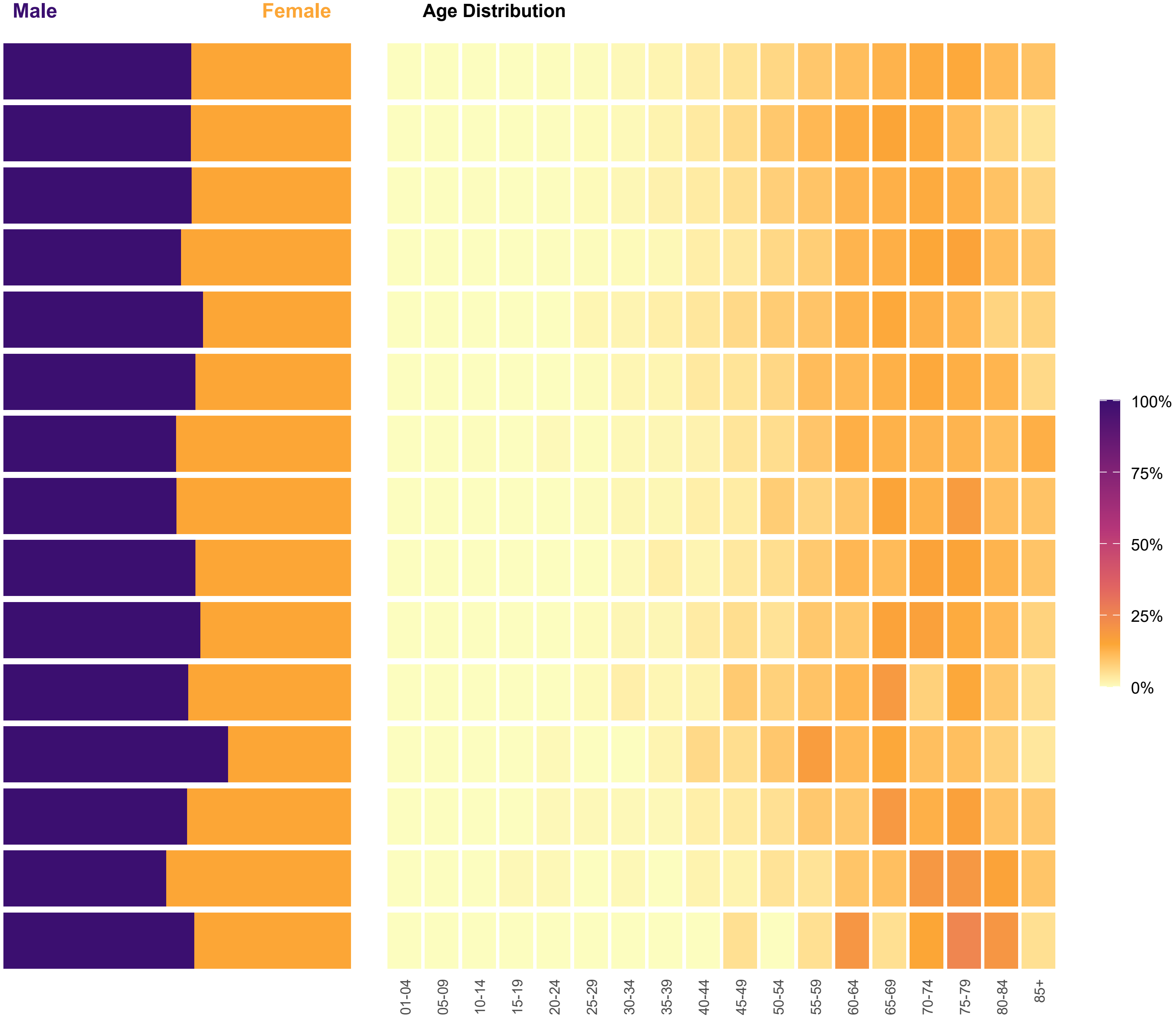

Primary Site: Small intestine | Phenotype: Grouped Phenotypes

Top 13 Morphologies | cases: 37,994

Morphology	n	%
Neuroendocrine tumor	34,100	89.8%
Neuroendocrine tumor grade 2	2,778	7.3%
Enterochromaffin cell carcinoid	159	0.4%
Small cell carcinoma	153	0.4%
Mixed adenoneuroendocrine carcinoma	145	0.4%
Gastrinoma	134	0.4%
Large cell neuroendocrine carcinoma	133	0.4%
Malignant melanoma	113	0.3%
Adenocarcinoid tumor	71	0.2%
Goblet cell carcinoid	41	0.1%
Large cell carcinoma	30	<0.1%
Carcinosarcoma	24	<0.1%
Somatostatinoma	21	<0.1%

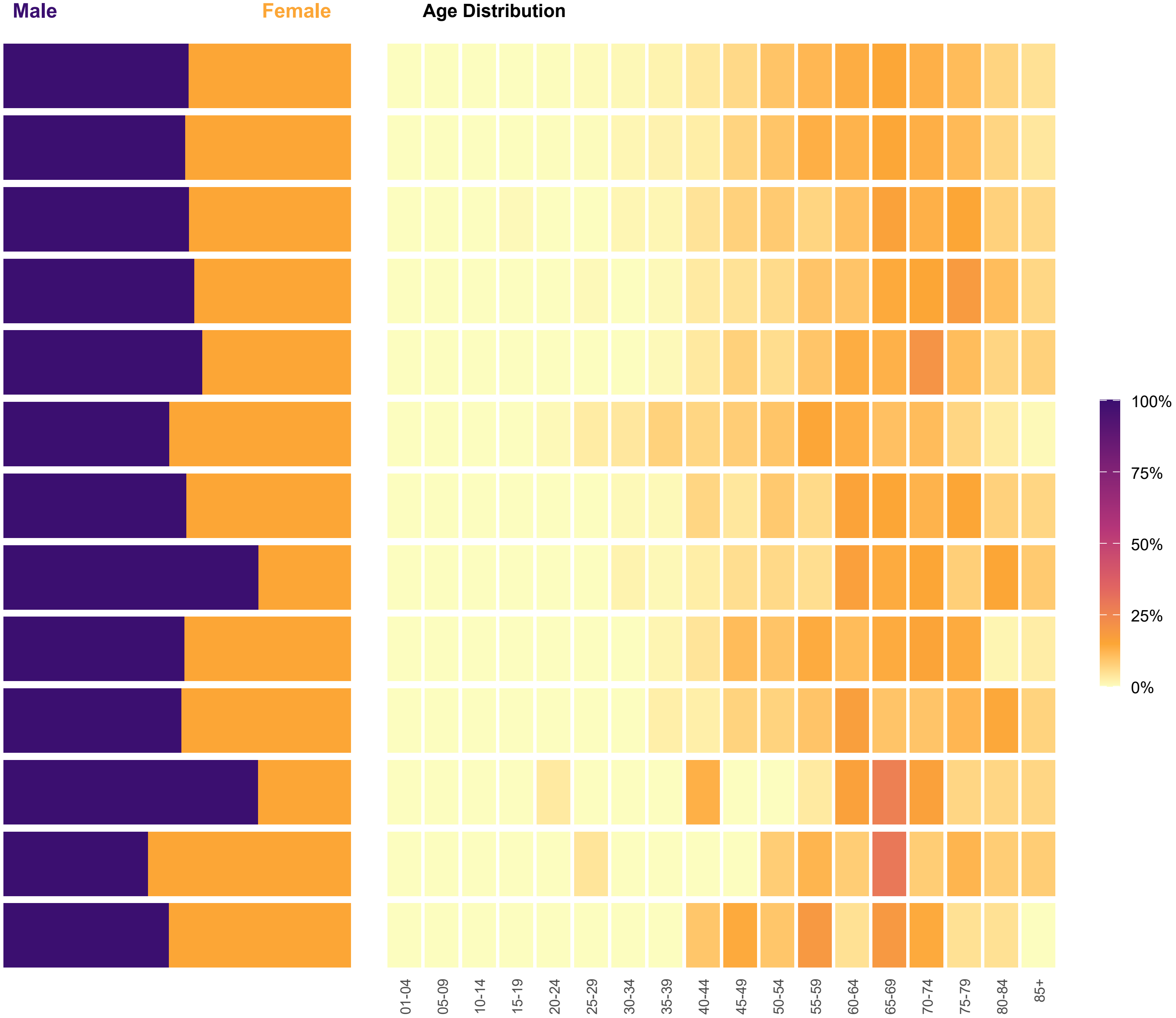

Primary Site: Stomach | Phenotype: epithelial

Top 25 Morphologies | cases: 348,860

Morphology	n	%
Adenocarcinoma	175,782	50.4%
Signet ring cell carcinoma	67,368	19.3%
Adenocarcinoma intestinal type	63,827	18.3%
Tubular adenocarcinoma	12,958	3.7%
Mucinous adenocarcinoma	6,329	1.8%
Neuroendocrine carcinoma	4,717	1.4%
Adenocarcinoma with mixed subtypes	3,881	1.1%
Mucin-producing adenocarcinoma	3,767	1.1%
Squamous cell carcinoma	2,712	0.8%
Papillary adenocarcinoma	1,502	0.4%
Adenocarcinoma in tubulovillous…	1,470	0.4%
Adenocarcinoma in adenomatous polyp	1,374	0.4%
Linitis plastica	1,372	0.4%
Adenocarcinoma with neuroendocrine…	334	<0.1%
Mixed cell adenocarcinoma	240	<0.1%
Superficial spreading…	202	<0.1%
Scirrhous adenocarcinoma	191	<0.1%
Adenocarcinoma in villous adenoma	156	<0.1%
Hepatoid adenocarcinoma	107	<0.1%
Lymphoepithelial carcinoma	103	<0.1%
Cribriform carcinoma	96	<0.1%
Clear cell adenocarcinoma	90	<0.1%
Trabecular adenocarcinoma	40	<0.1%
Basaloid squamous cell carcinoma	30	<0.1%
Acinar cell carcinoma	29	<0.1%

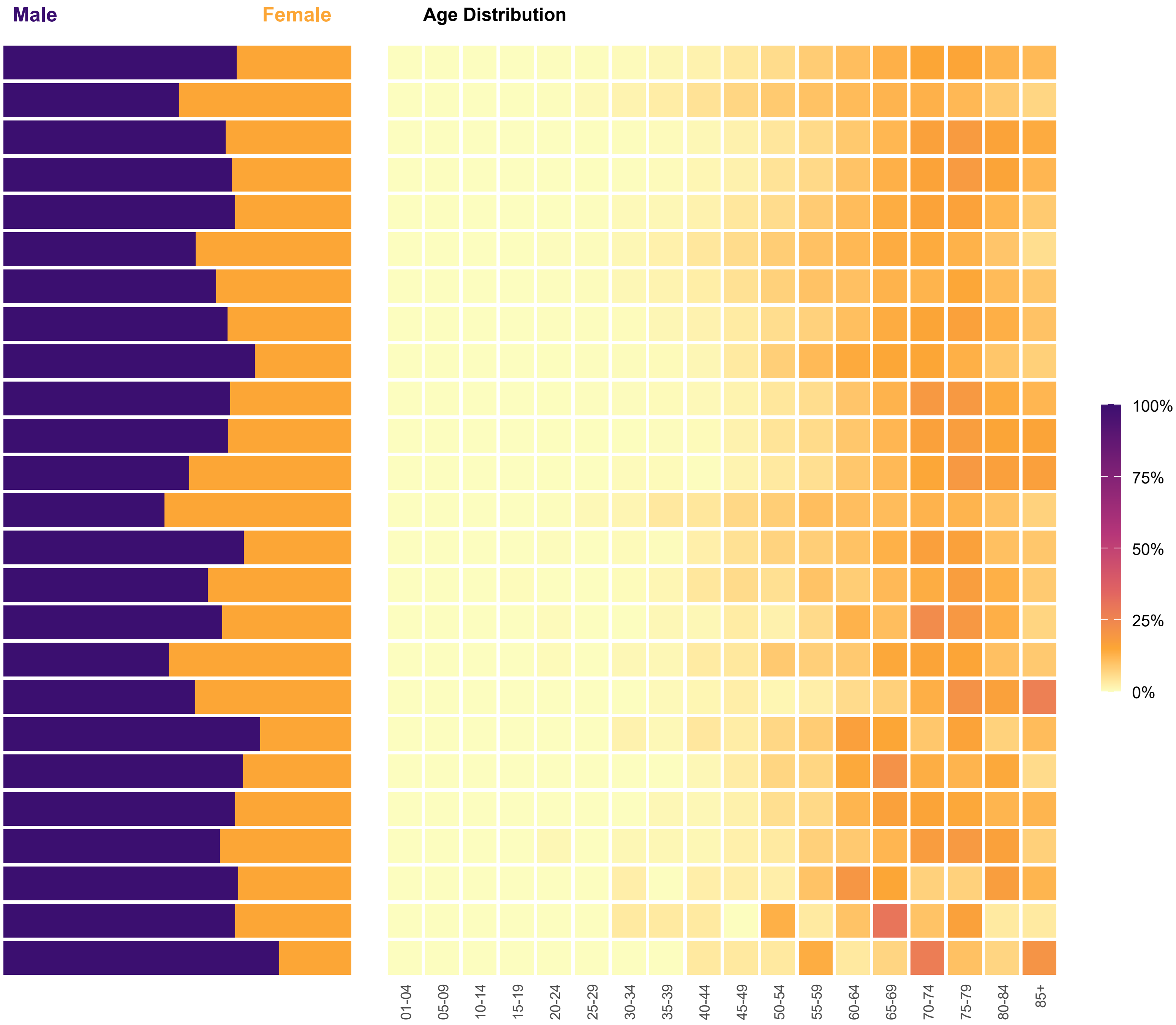

Primary Site: Stomach | Phenotype: Grouped Phenotypes

Top 14 Morphologies | cases: 16,366

Morphology	n	%
Neuroendocrine tumor	13,115	80.1%
Neuroendocrine tumor grade 2	1,269	7.8%
Small cell carcinoma	717	4.4%
Large cell neuroendocrine carcinoma	345	2.1%
Mixed adenoneuroendocrine carcinoma	332	2.0%
Large cell carcinoma	111	0.7%
Malignant melanoma	107	0.7%
Enterochromaffin cell carcinoid	48	0.3%
Adenocarcinoid tumor	48	0.3%
Carcinosarcoma	46	0.3%
Gastrinoma	41	0.3%
Enterochromaffin-like cell…	29	0.2%
Goblet cell carcinoid	23	0.1%
Rhabdoid tumor	23	0.1%

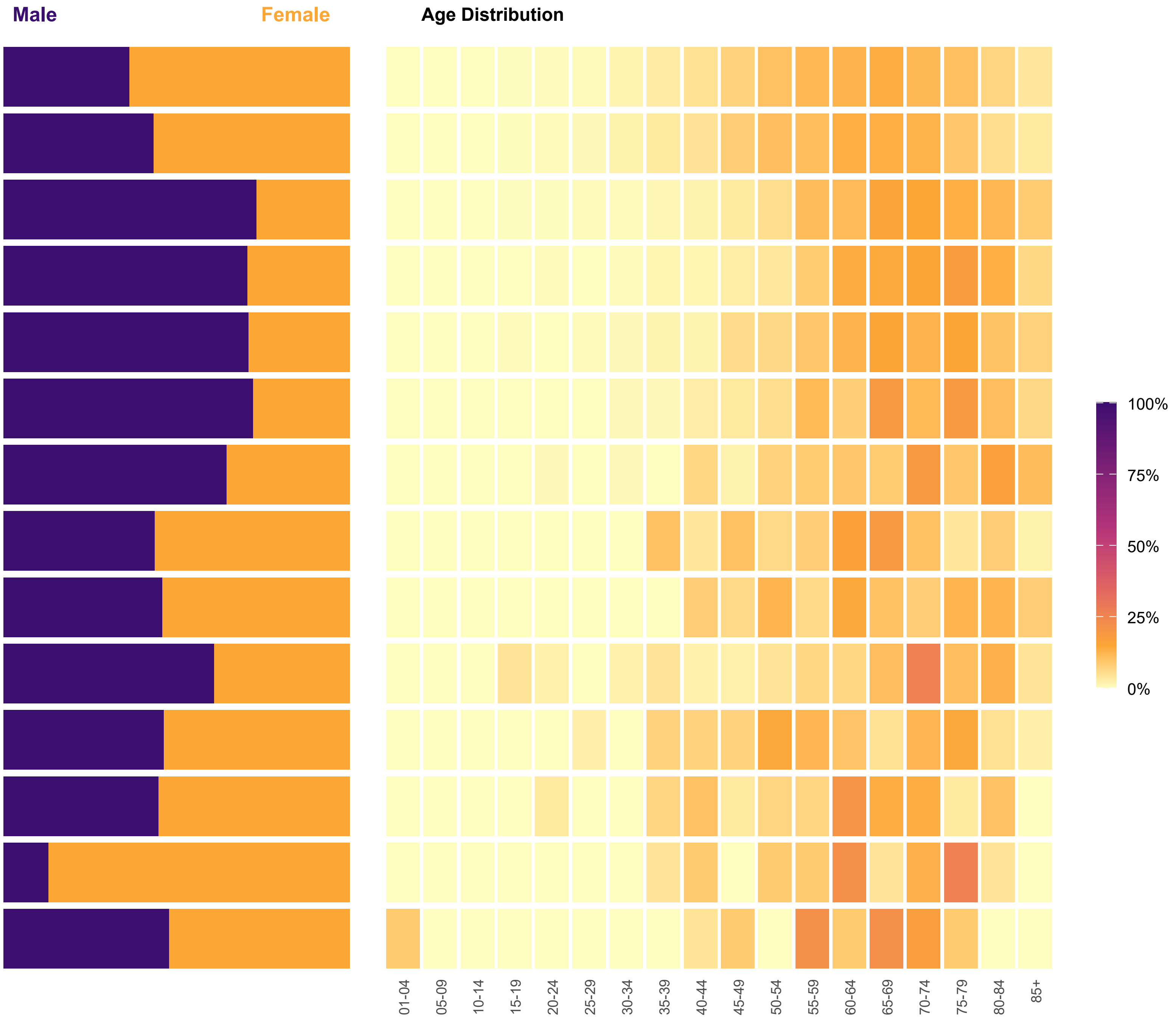

Primary Site: Testis | Phenotype: epithelial

Top 3 Morphologies | cases: 306

Morphology	n	%
Adenocarcinoma	183	59.8%
Squamous cell carcinoma	38	12.4%
Neuroendocrine carcinoma	23	7.5%

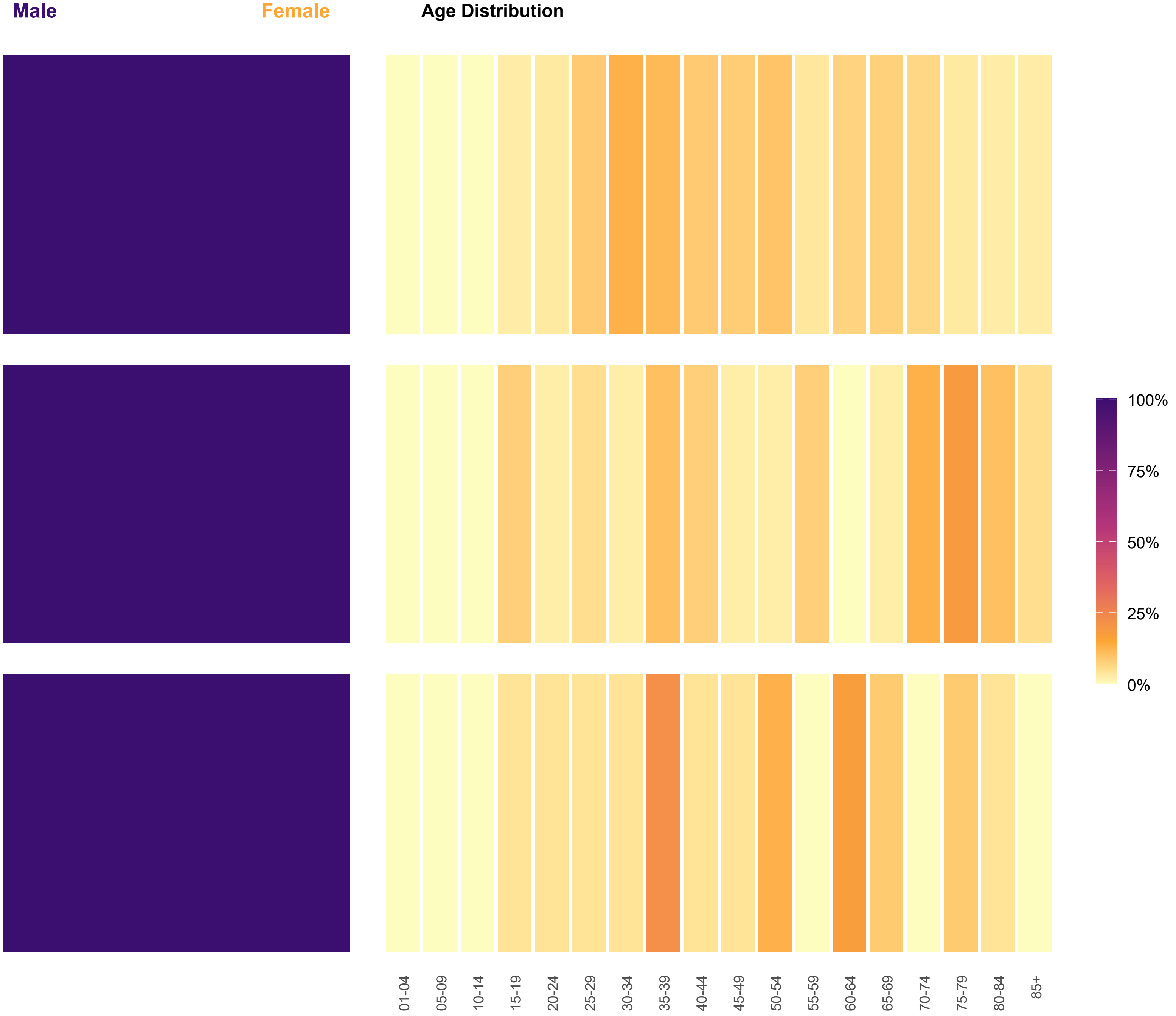

Primary Site: Testis | Phenotype: Grouped Phenotypes

Top 21 Morphologies | cases: 136,518

Morphology	n	%
Seminoma	78,037	57.2%
Mixed germ cell tumor	26,762	19.6%
Embryonal carcinoma	12,070	8.8%
Teratocarcinoma	3,694	2.7%
Germinoma	2,940	2.2%
Teratoma	2,924	2.1%
Germ cell tumor nonseminomatous	2,861	2.1%
Choriocarcinoma combined with…	2,014	1.5%
Yolk sac tumor	1,696	1.2%
Choriocarcinoma	835	0.6%
Spermatocytic seminoma	830	0.6%
Seminoma anaplastic	657	0.5%
Leydig cell tumor malignant	507	0.4%
Sertoli cell carcinoma	145	0.1%
Teratoma with malignant…	120	<0.1%
Malignant teratoma intermediate	106	<0.1%
Neuroendocrine tumor	88	<0.1%
Dysgerminoma	55	<0.1%
Malignant teratoma undifferentiated	55	<0.1%
Malignant teratoma trophoblastic	23	<0.1%
Adult granulosa cell tumor of ovary	20	<0.1%

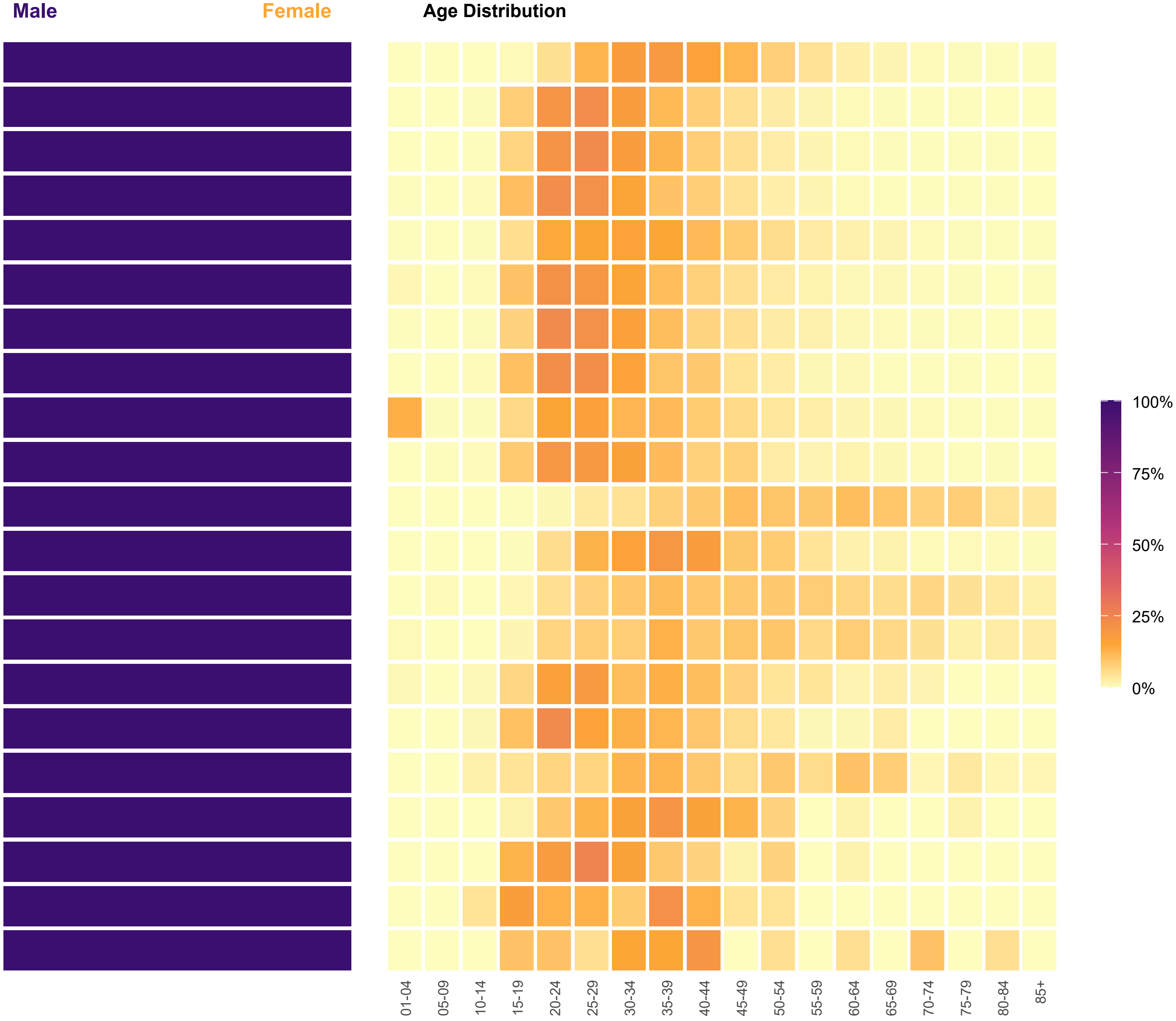

Primary Site: Thymus | Phenotype: epithelial

Top 14 Morphologies | cases: 12,188

Morphology	n	%
Thymoma	2,765	22.7%
Thymic carcinoma	1,745	14.3%
Thymoma type AB	1,563	12.8%
Thymoma type B2	1,374	11.3%
Thymoma type B3	1,264	10.4%
Squamous cell carcinoma	1,244	10.2%
Thymoma type B1	986	8.1%
Thymoma type A	744	6.1%
Neuroendocrine carcinoma	233	1.9%
Adenocarcinoma	49	0.4%
Lymphoepithelial carcinoma	48	0.4%
Mucoepidermoid carcinoma	41	0.3%
Basaloid squamous cell carcinoma	36	0.3%
Basaloid carcinoma	31	0.3%

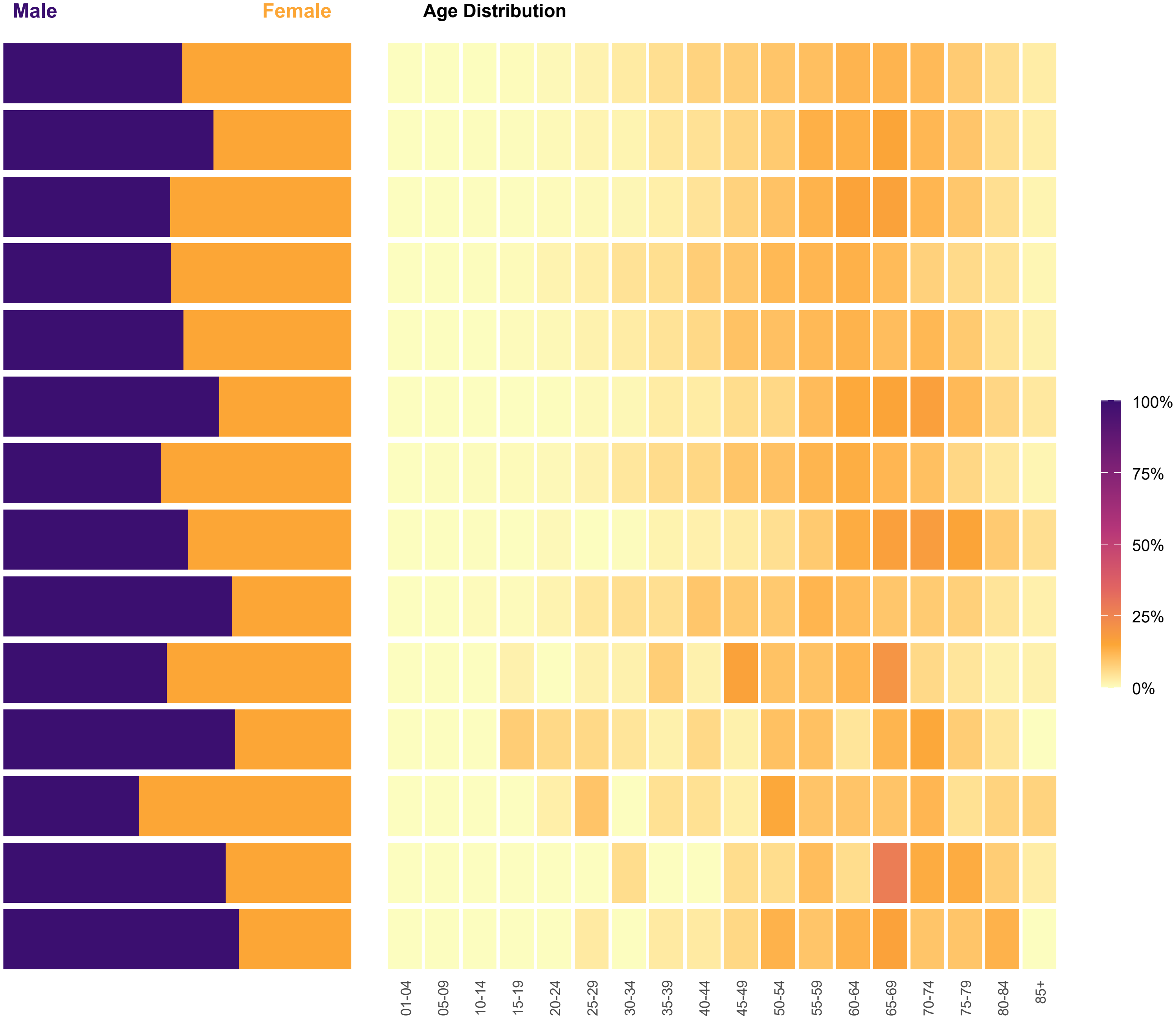

Primary Site: Thymus | Phenotype: Grouped Phenotypes

Top 10 Morphologies | cases: 1,180

Morphology	n	%
Mediastinal large B-cell lymphoma	293	24.8%
Neuroendocrine tumor	188	15.9%
Diffuse large B-cell lymphoma	177	15.0%
Neuroendocrine tumor grade 2	158	13.4%
Hodgkin lymphoma nodular sclerosis	69	5.8%
Large cell neuroendocrine carcinoma	51	4.3%
Marginal zone B-cell lymphoma	38	3.2%
Small cell carcinoma	31	2.6%
Large cell carcinoma	27	2.3%
Seminoma	20	1.7%

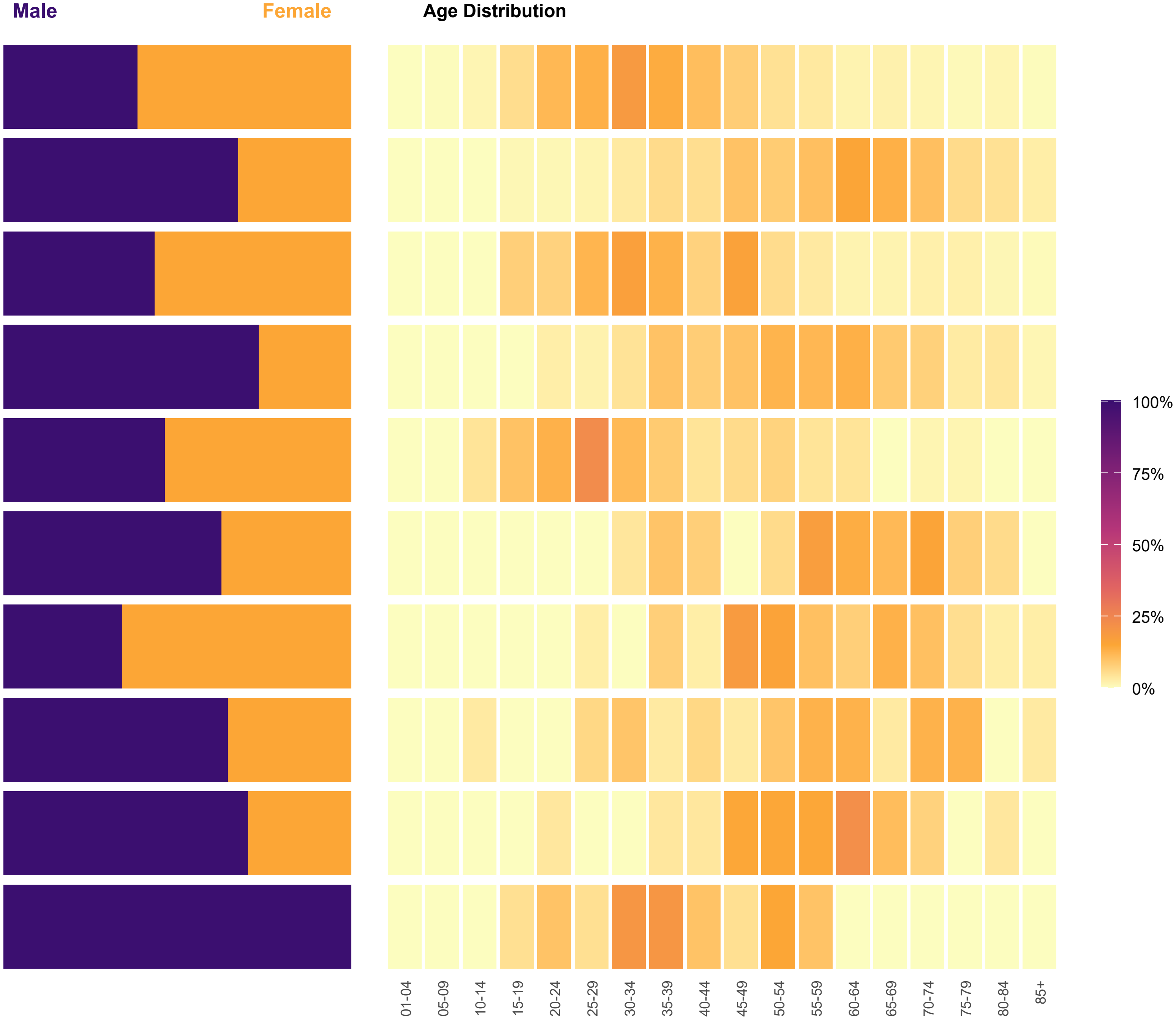

Primary Site: Thyroid | Phenotype: epithelial

Top 24 Morphologies | cases: 395,470

Morphology	n	%
Papillary adenocarcinoma	227,060	57.4%
Papillary carcinoma follicular…	91,484	23.1%
Papillary microcarcinoma	23,140	5.9%
Follicular carcinoma	22,119	5.6%
Oxyphilic adenocarcinoma	8,914	2.3%
Papillary carcinoma columnar cell	6,533	1.7%
Follicular carcinoma minimally…	5,652	1.4%
Papillary carcinoma encapsulated…	4,592	1.2%
Follicular adenocarcinoma well…	1,390	0.4%
Poorly differentiated thyroid…	882	0.2%
Papillary carcinoma oncocytic…	797	0.2%
Squamous cell carcinoma	658	0.2%
Adenocarcinoma	576	0.1%
Follicular adenocarcinoma…	433	0.1%
Follicular carcinoma encapsulated…	394	<0.1%
Invasive micropapillary carcinoma…	151	<0.1%
Adenocarcinoma with mixed subtypes	140	<0.1%
Neuroendocrine carcinoma	80	<0.1%
Mucoepidermoid carcinoma	71	<0.1%
Clear cell adenocarcinoma	68	<0.1%
Encapsulated papillary carcinoma…	61	<0.1%
Papillary squamous cell carcinoma	40	<0.1%
Trabecular adenocarcinoma	37	<0.1%
Papillary cystadenocarcinoma	24	<0.1%

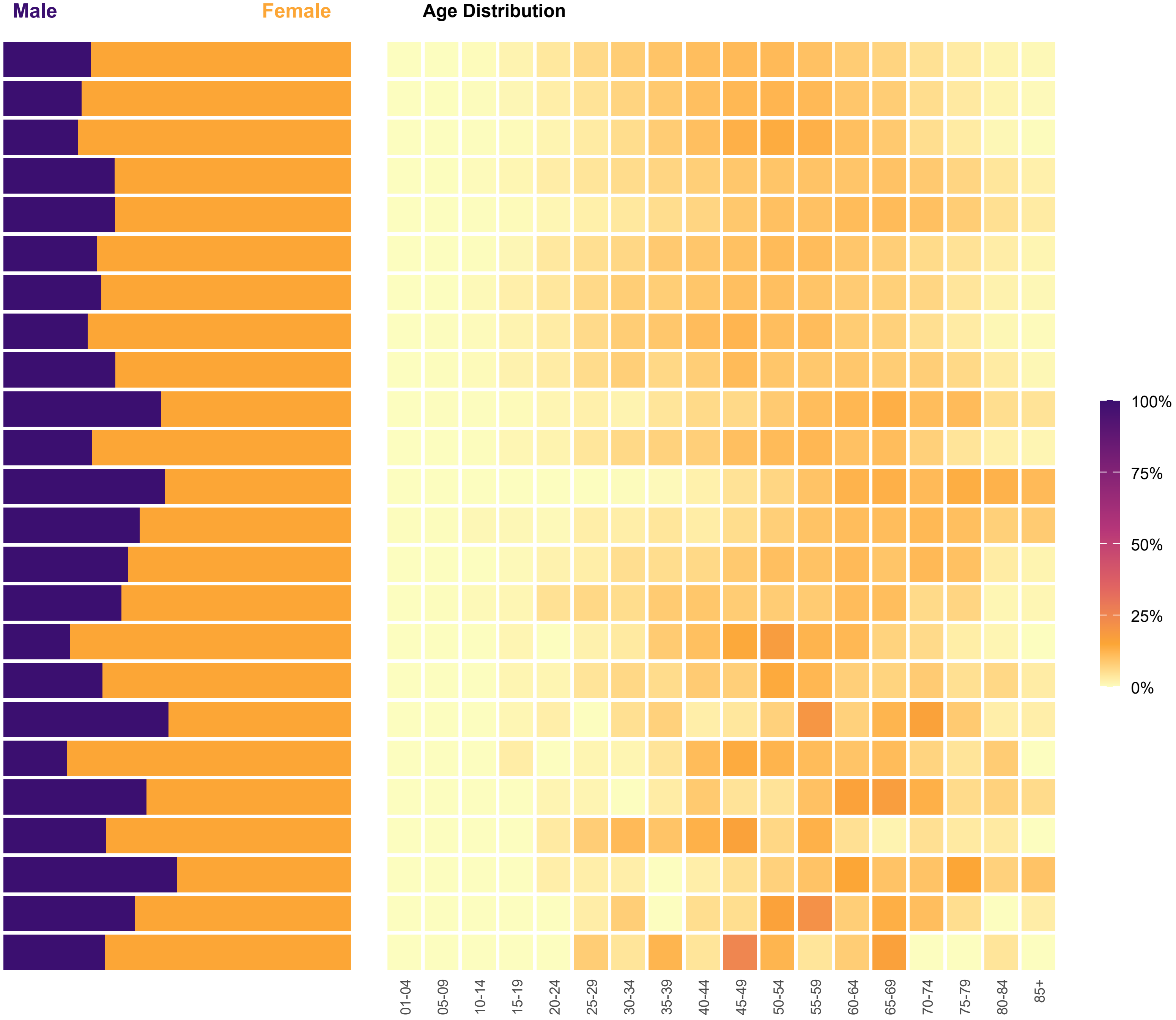

Primary Site: Thyroid | Phenotype: Grouped Phenotypes

Top 16 Morphologies | cases: 8,938

Morphology	n	%
Medullary thyroid carcinoma	5,404	60.5%
Diffuse large B-cell lymphoma	1,677	18.8%
Marginal zone B-cell lymphoma	552	6.2%
lymphoma non-Hodgkin	240	2.7%
Hemangiosarcoma	138	1.5%
Follicular lymphoma grade 3	98	1.1%
Follicular lymphoma	88	1.0%
Follicular lymphoma grade 1/2	74	0.8%
Burkitt lymphoma	72	0.8%
lymphoma	64	0.7%
Sarcoma	47	0.5%
Large cell carcinoma	45	0.5%
Small cell carcinoma	39	0.4%
Leiomyosarcoma	34	0.4%
Plasmacytoma extramedullary	30	0.3%
Neuroendocrine tumor	20	0.2%

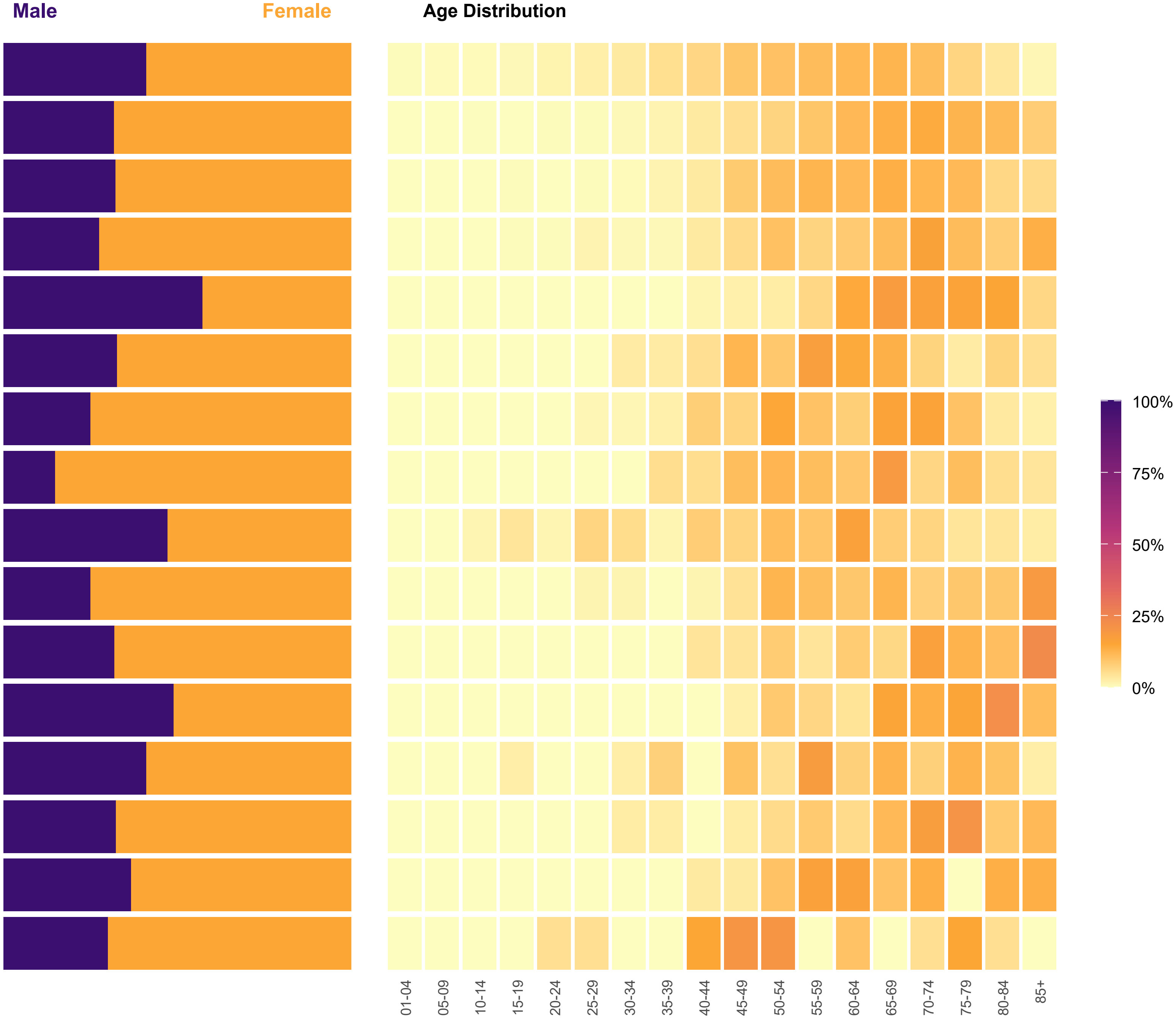

Primary Site: Tongue | Phenotype: epithelial

Top 13 Morphologies | cases: 130,633

Morphology	n	%
Squamous cell carcinoma	125,463	96.0%
Basaloid squamous cell carcinoma	2,689	2.1%
Mucoepidermoid carcinoma	615	0.5%
Adenoid cystic carcinoma	590	0.5%
Papillary squamous cell carcinoma	440	0.3%
Adenocarcinoma	304	0.2%
Lymphoepithelial carcinoma	173	0.1%
Neuroendocrine carcinoma	65	<0.1%
Clear cell adenocarcinoma	49	<0.1%
Polymorphous adenocarcinoma	46	<0.1%
Basosquamous carcinoma	33	<0.1%
Basaloid carcinoma	30	<0.1%
Mucinous adenocarcinoma	29	<0.1%

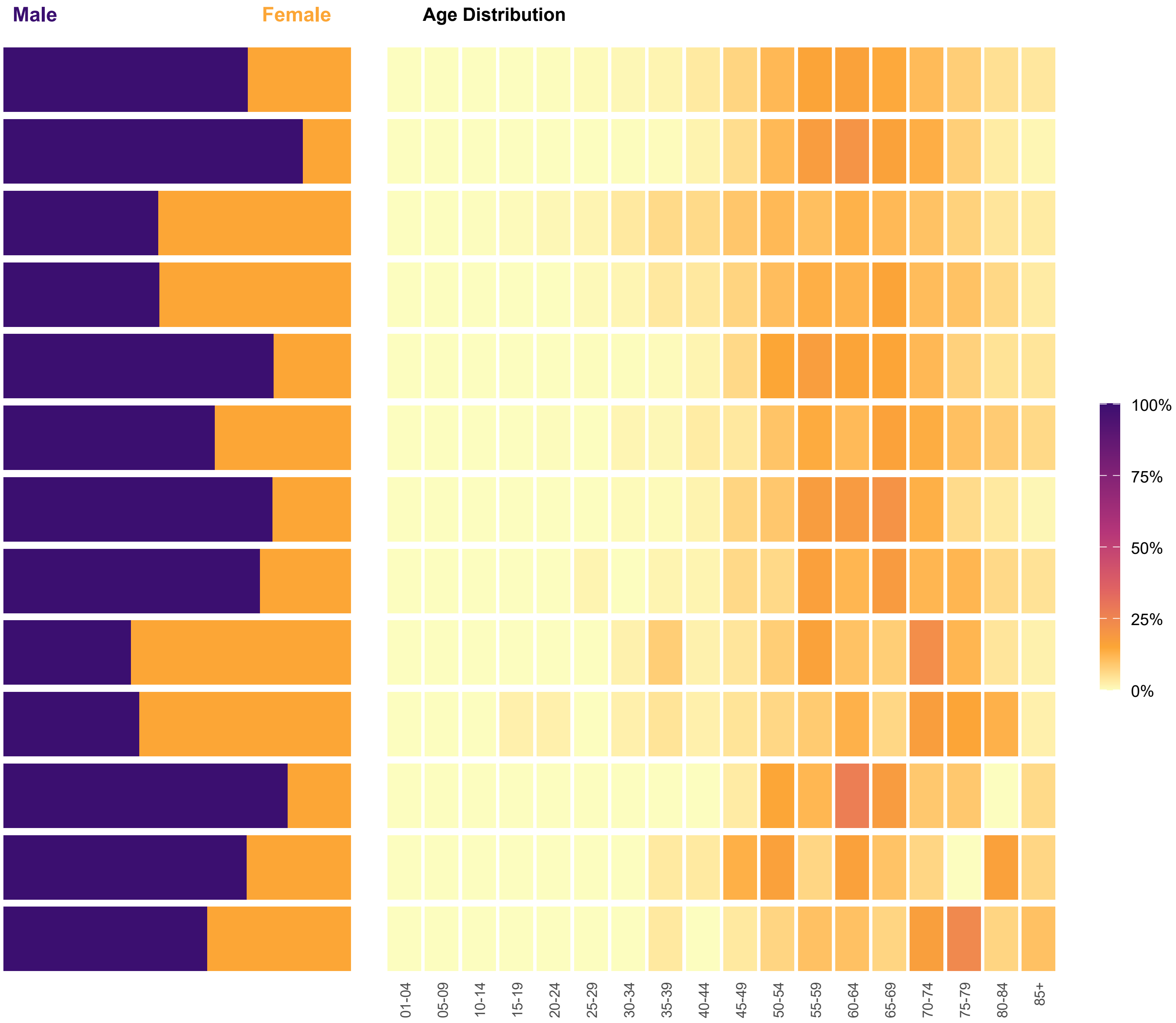

Primary Site: Tongue | Phenotype: Grouped Phenotypes

Top 17 Morphologies | cases: 2,413

Morphology	n	%
Diffuse large B-cell lymphoma	1,033	42.8%
Marginal zone B-cell lymphoma	147	6.1%
Mantle cell lymphoma	139	5.8%
lymphoma non-Hodgkin	104	4.3%
Kaposi sarcoma	99	4.1%
Mature T-cell lymphoma	75	3.1%
Follicular lymphoma grade 1/2	72	3.0%
Small cell carcinoma	64	2.7%
Plasmacytoma extramedullary	52	2.2%
lymphoma	49	2.0%
Follicular lymphoma	43	1.8%
Sarcoma	40	1.7%
Follicular lymphoma grade 3	37	1.5%
Malignant melanoma	34	1.4%
Leiomyosarcoma	29	1.2%
Burkitt lymphoma	29	1.2%
B-cell chronic lymphocytic…	27	1.1%

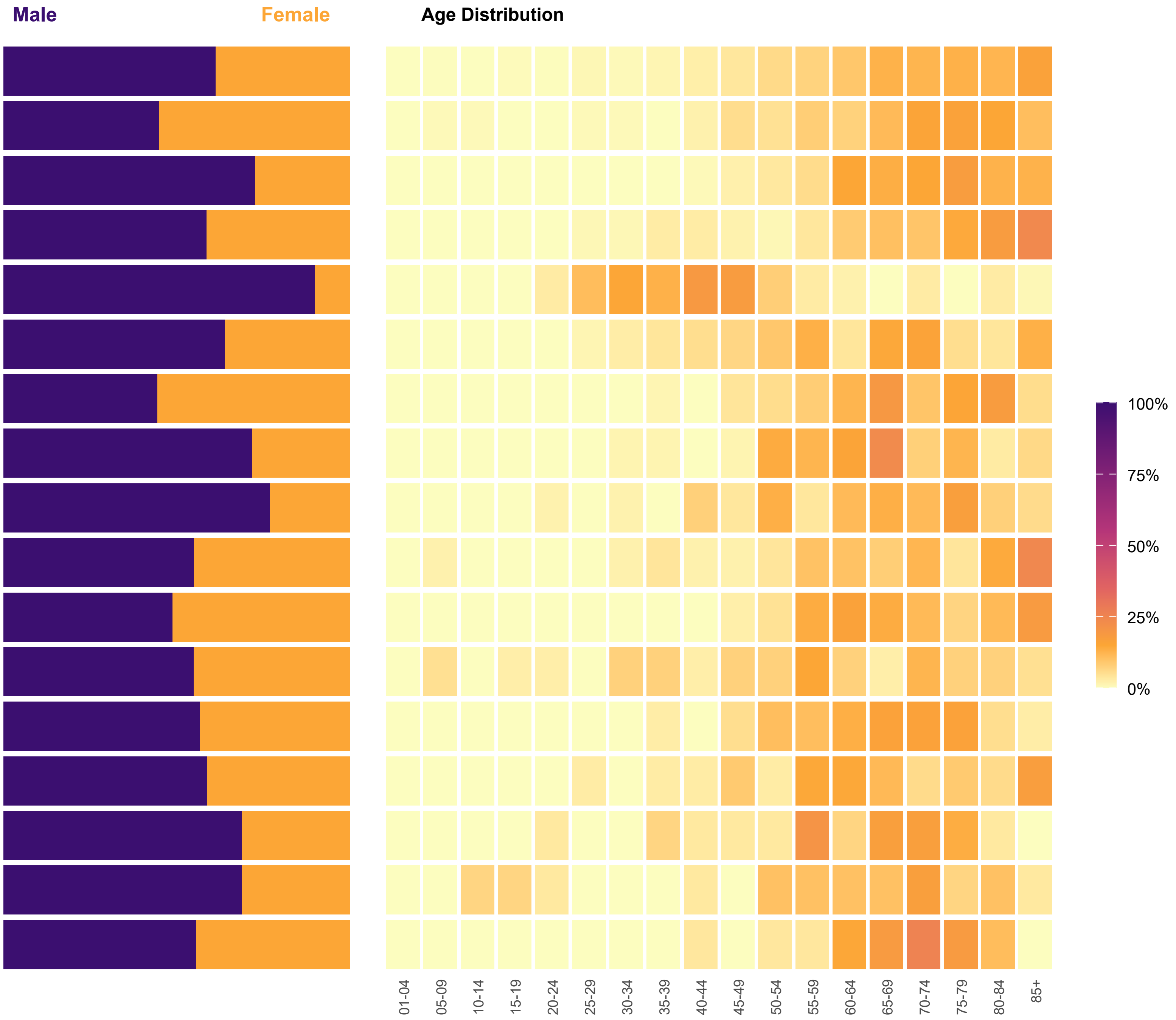

Primary Site: Trachea | Phenotype: epithelial

Top 7 Morphologies | cases: 2,359

Morphology	n	%
Squamous cell carcinoma	1,563	66.3%
Adenoid cystic carcinoma	441	18.7%
Adenocarcinoma	194	8.2%
Mucoepidermoid carcinoma	46	1.9%
Neuroendocrine carcinoma	31	1.3%
Papillary squamous cell carcinoma	27	1.1%
Basaloid squamous cell carcinoma	20	0.8%

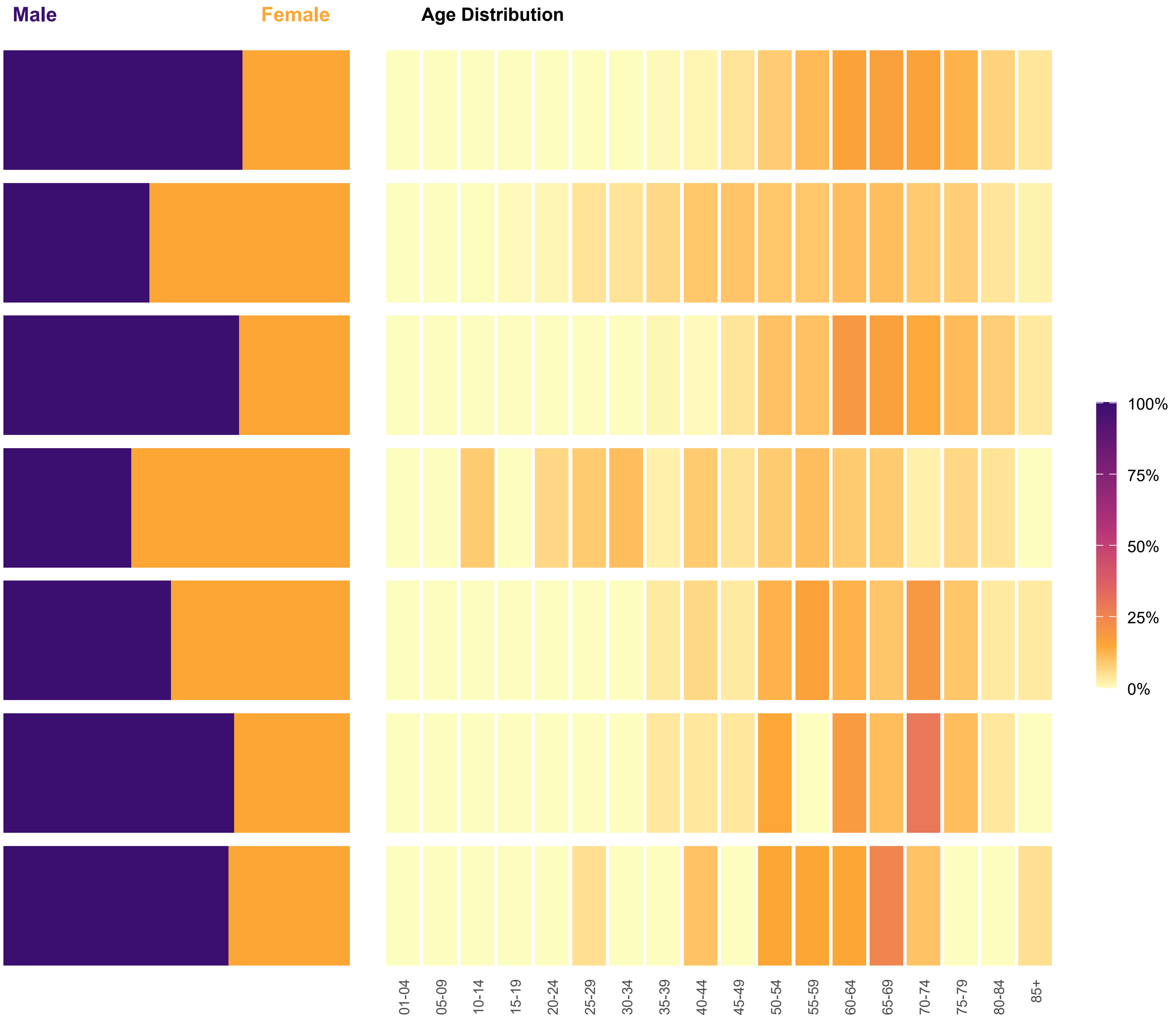

Primary Site: Trachea | Phenotype: Grouped Phenotypes

Top 6 Morphologies | cases: 417

Morphology	n	%
Small cell carcinoma	131	31.4%
Marginal zone B-cell lymphoma	33	7.9%
Chondrosarcoma	32	7.7%
Large cell carcinoma	25	6.0%
Neuroendocrine tumor	25	6.0%
Diffuse large B-cell lymphoma	25	6.0%

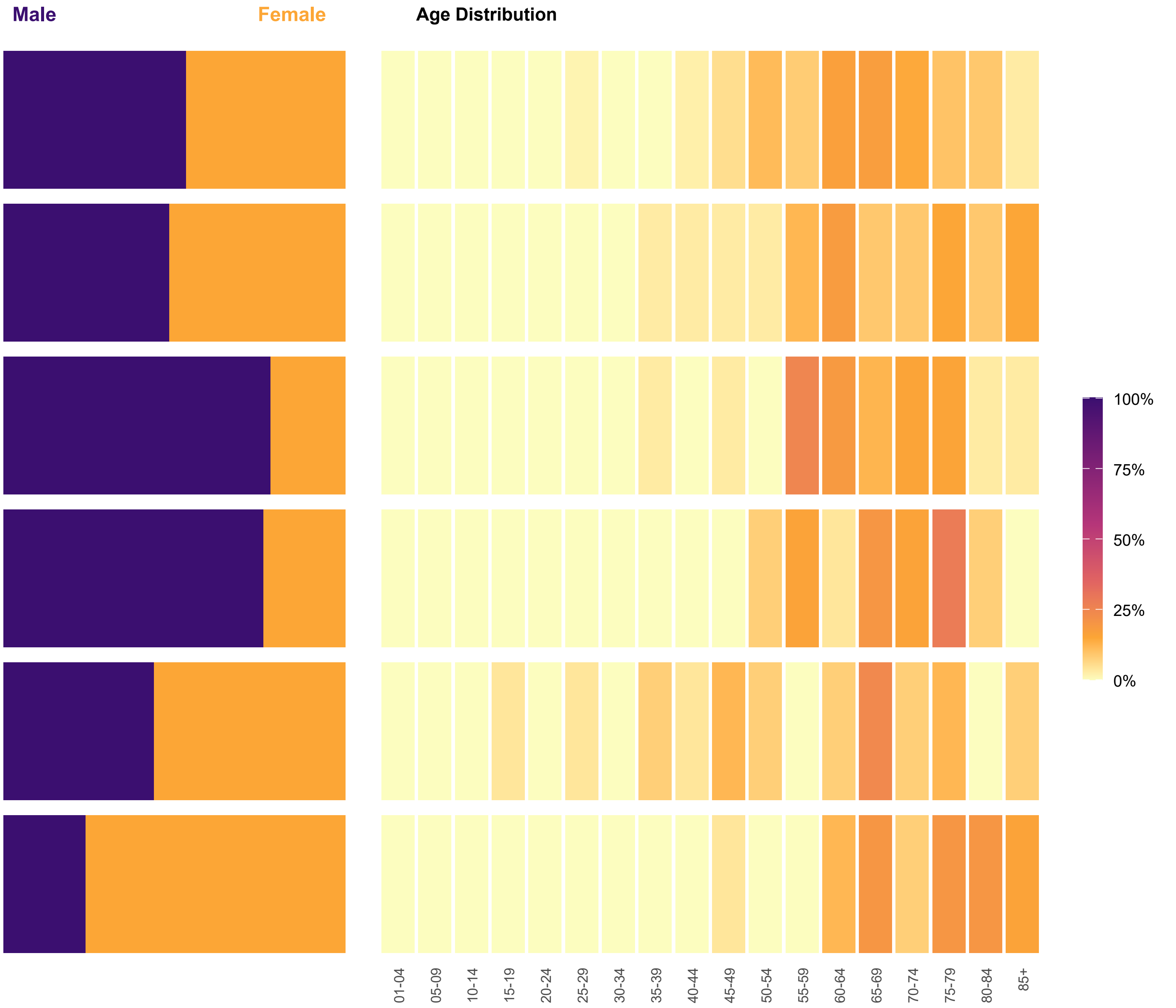

Primary Site: Ureter | Phenotype: epithelial

Top 8 Morphologies | cases: 23,428

Morphology	n	%
Transitional cell carcinoma	13,427	57.3%
Papillary transitional cell…	9,221	39.4%
Squamous cell carcinoma	348	1.5%
Adenocarcinoma	167	0.7%
Urothelial carcinoma sarcomatoid	85	0.4%
Neuroendocrine carcinoma	40	0.2%
Clear cell adenocarcinoma	31	0.1%
Urothelial carcinoma micropapillary	26	0.1%

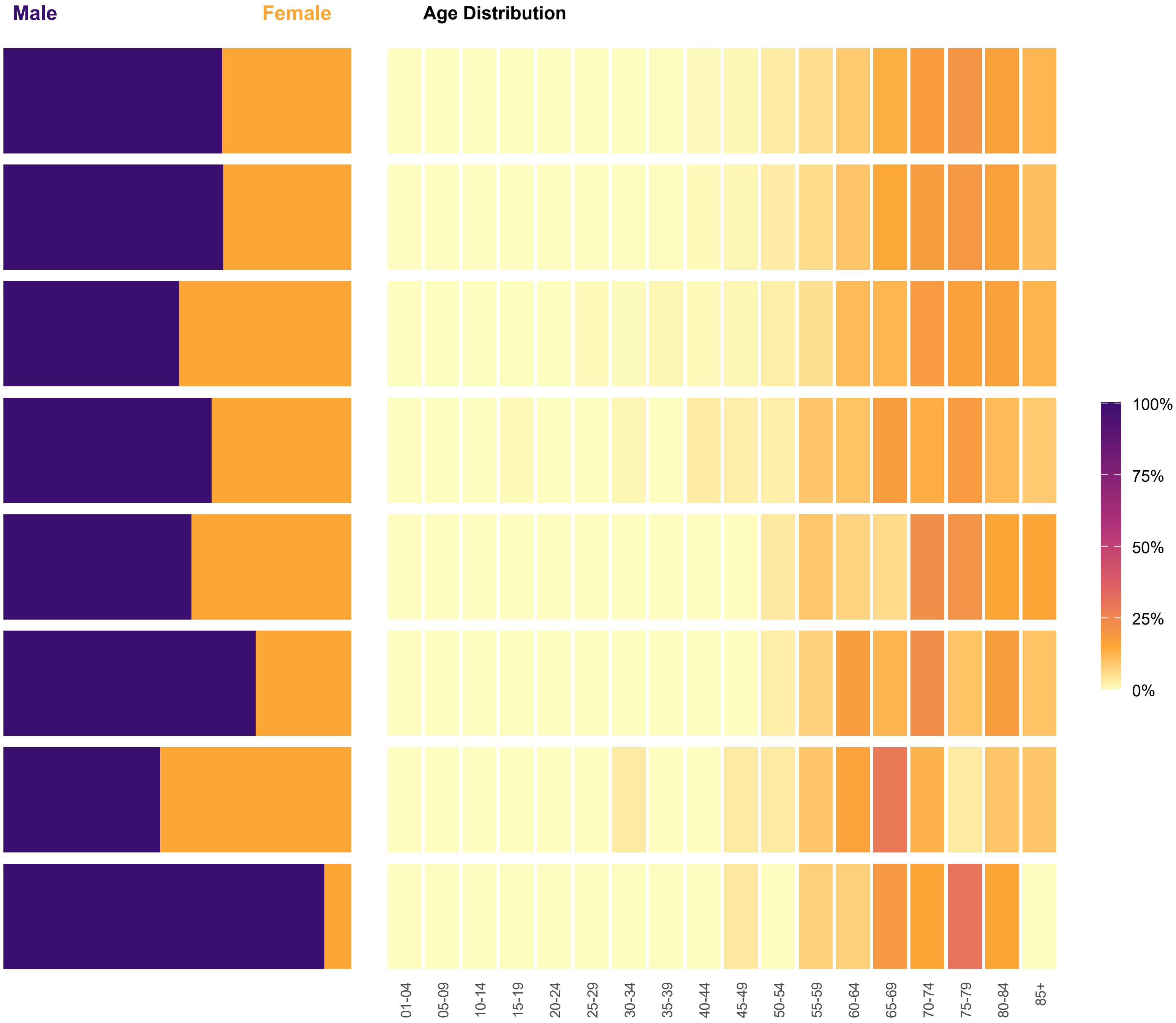

Primary Site: Ureter | Phenotype: Grouped Phenotypes

Top 2 Morphologies | cases: 303

Morphology	n	%
Small cell carcinoma	136	44.9%
Leiomyosarcoma	20	6.6%

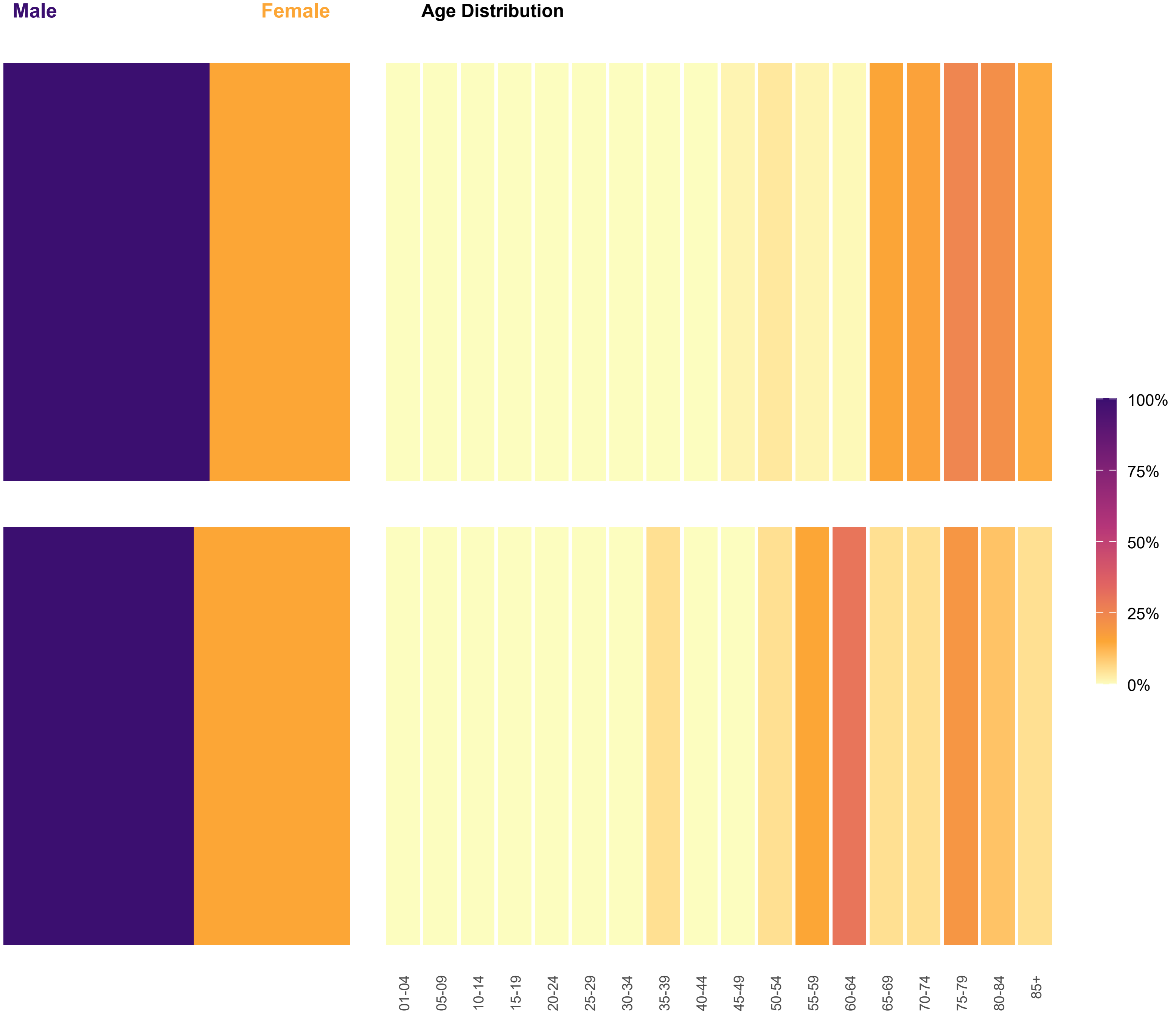

Primary Site: Urethra | Phenotype: epithelial

Top 10 Morphologies | cases: 6,331

Morphology	n	%
Transitional cell carcinoma	2,692	42.5%
Squamous cell carcinoma	1,541	24.3%
Papillary transitional cell…	1,068	16.9%
Adenocarcinoma	508	8.0%
Clear cell adenocarcinoma	169	2.7%
Mucinous adenocarcinoma	94	1.5%
Papillary adenocarcinoma	36	0.6%
Basaloid squamous cell carcinoma	26	0.4%
Urothelial carcinoma sarcomatoid	23	0.4%
Papillary squamous cell carcinoma	20	0.3%

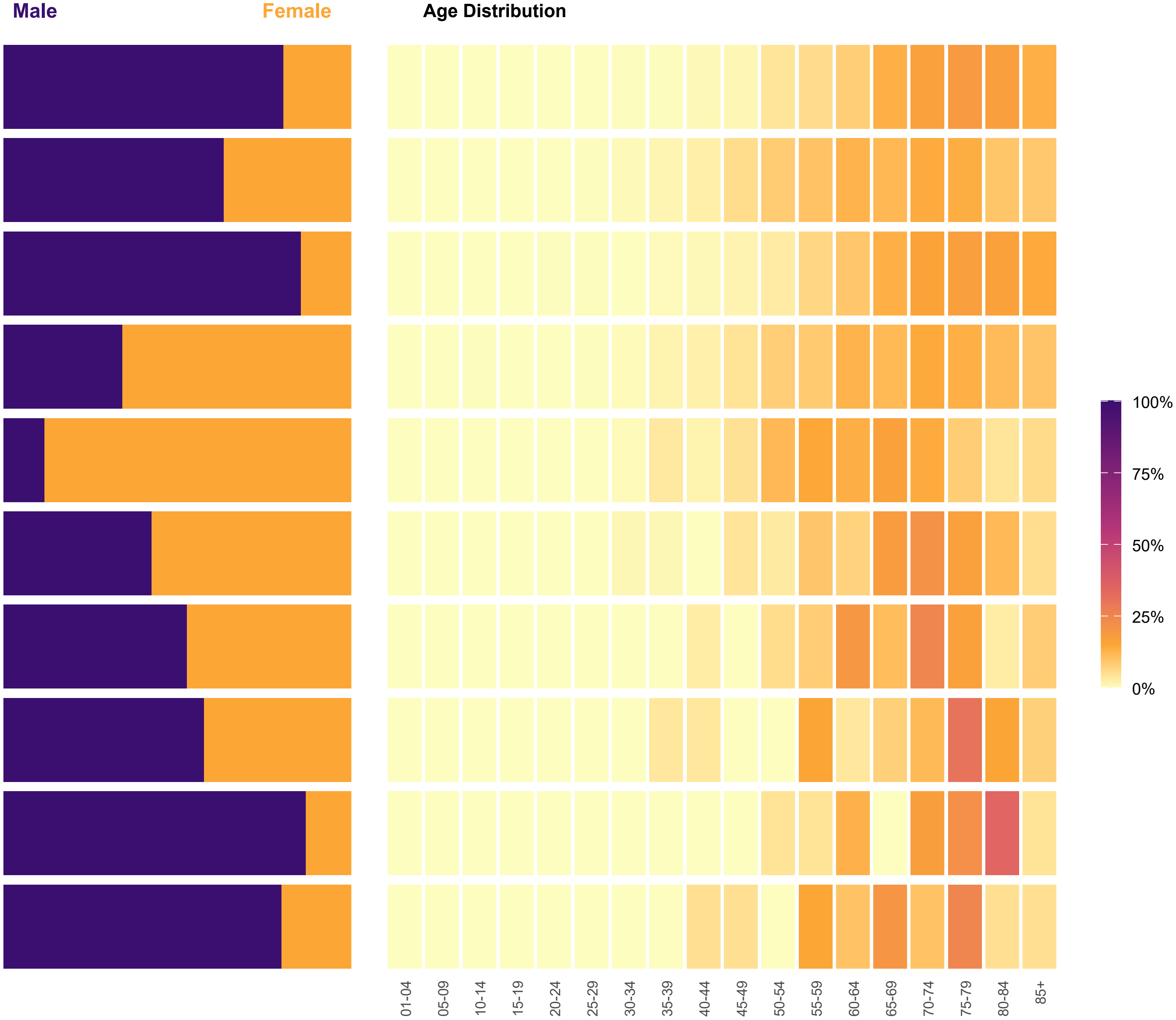

Primary Site: Urethra | Phenotype: Grouped Phenotypes

Top 2 Morphologies | cases: 267

Morphology	n	%
Malignant melanoma	151	56.6%
Small cell carcinoma	35	13.1%

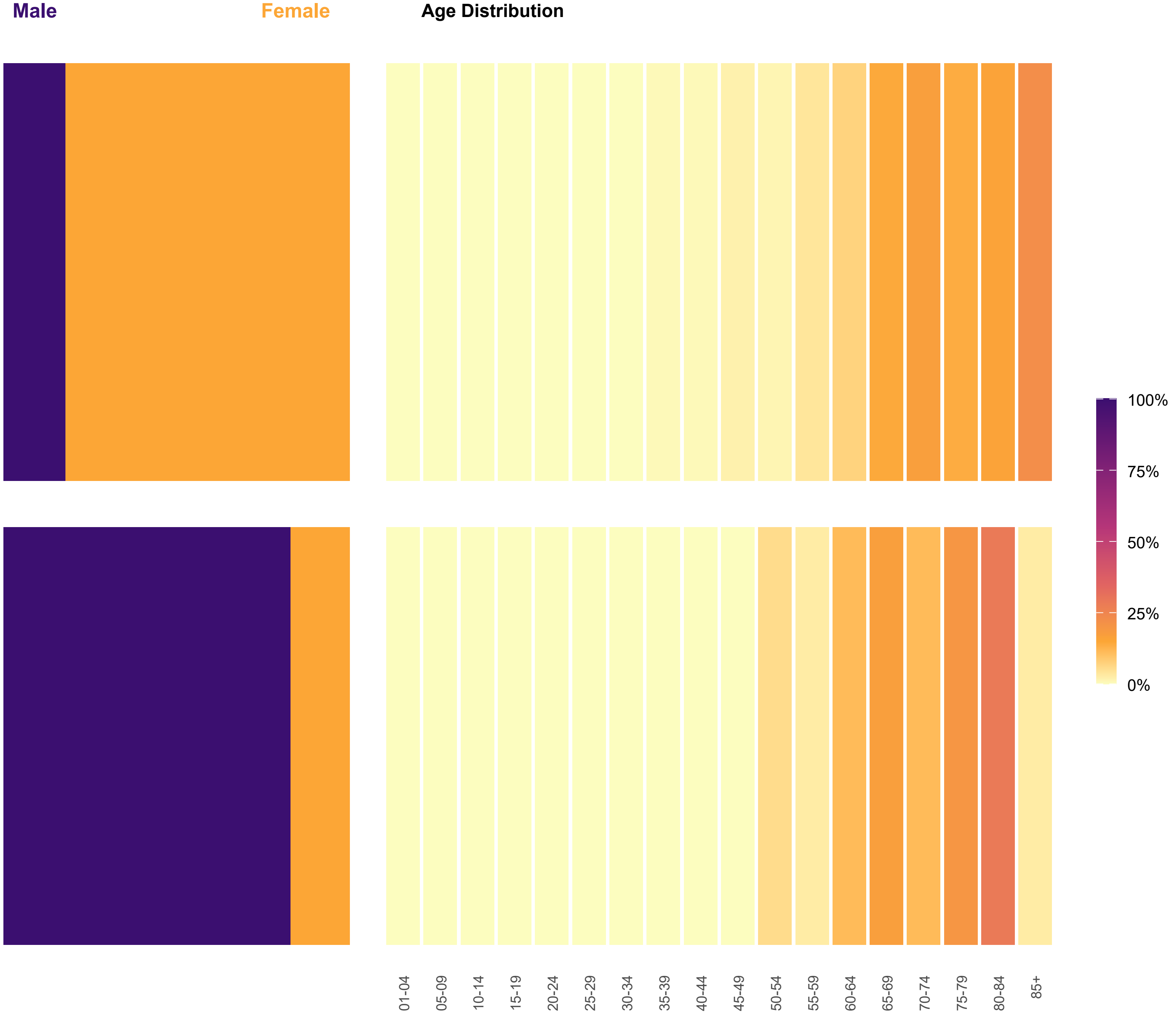

Primary Site: Urinary Bladder | Phenotype: epithelial

Top 22 Morphologies | cases: 750,255

Morphology	n	%
Papillary transitional cell…	439,304	58.6%
Transitional cell carcinoma	283,545	37.8%
Squamous cell carcinoma	11,478	1.5%
Adenocarcinoma	5,489	0.7%
Urothelial carcinoma sarcomatoid	2,801	0.4%
Urothelial carcinoma micropapillary	1,932	0.3%
Neuroendocrine carcinoma	1,486	0.2%
Mucinous adenocarcinoma	1,105	0.1%
Signet ring cell carcinoma	855	0.1%
Clear cell adenocarcinoma	383	<0.1%
Papillary adenocarcinoma	309	<0.1%
Papillary squamous cell carcinoma	268	<0.1%
Mucin-producing adenocarcinoma	256	<0.1%
Adenocarcinoma with mixed subtypes	242	<0.1%
Lymphoepithelial carcinoma	220	<0.1%
Adenocarcinoma intestinal type	136	<0.1%
Adenocarcinoma with neuroendocrine…	90	<0.1%
Mixed cell adenocarcinoma	62	<0.1%
Tubular adenocarcinoma	35	<0.1%
Adenocarcinoma in tubulovillous…	30	<0.1%
Adenocarcinoma in villous adenoma	24	<0.1%
Basaloid carcinoma	21	<0.1%

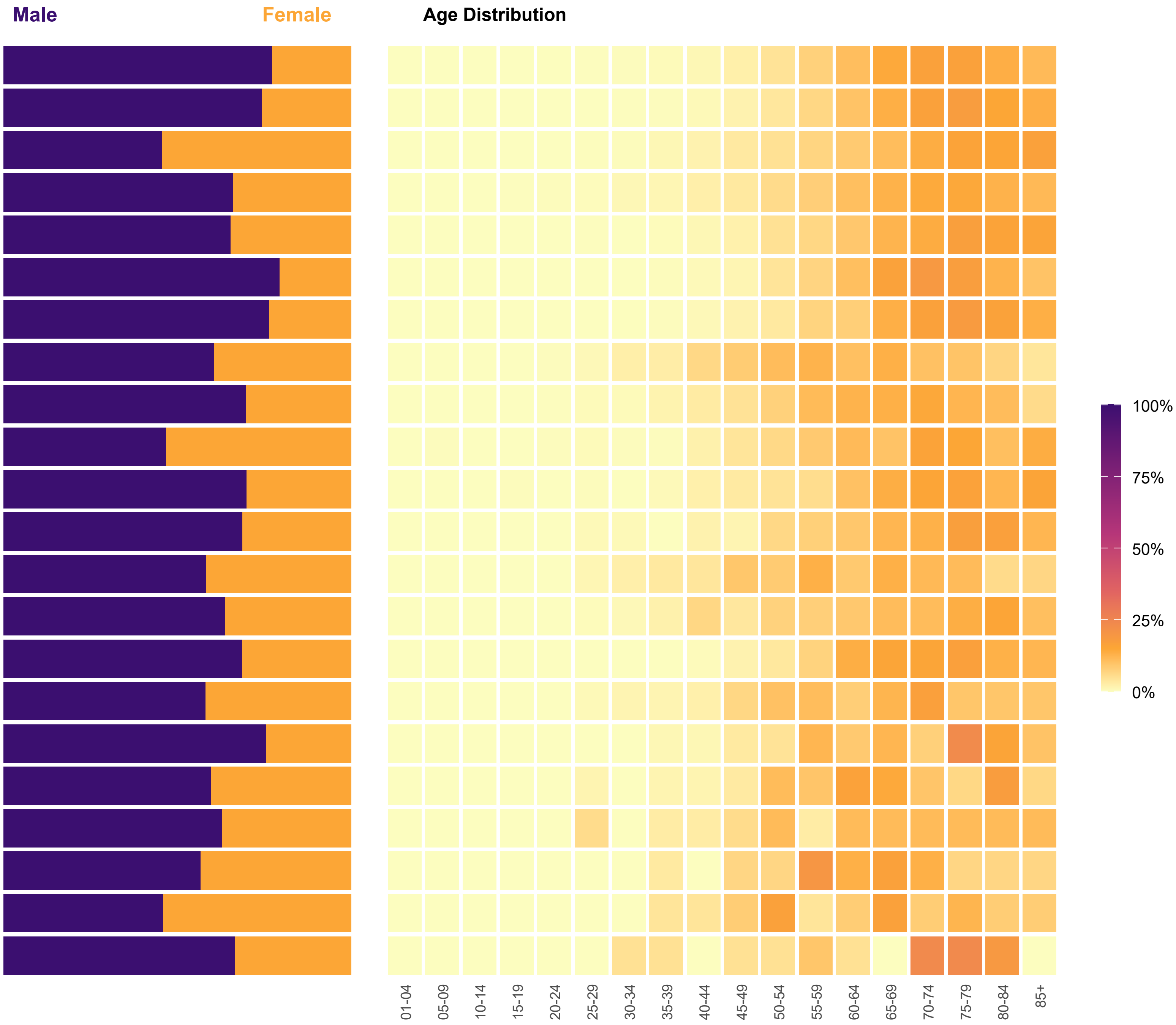

Primary Site: Urinary Bladder | Phenotype: Grouped Phenotypes

Top 25 Morphologies | cases: 8,139

Morphology	n	%
Small cell carcinoma	4,725	58.1%
Carcinosarcoma	532	6.5%
Leiomyosarcoma	446	5.5%
Combined small cell carcinoma	340	4.2%
Diffuse large B-cell lymphoma	255	3.1%
Large cell neuroendocrine carcinoma	224	2.8%
Sarcoma	222	2.7%
Embryonal rhabdomyosarcoma	163	2.0%
Marginal zone B-cell lymphoma	158	1.9%
Neuroendocrine tumor	79	1.0%
lymphoma non-Hodgkin	70	0.9%
Spindle cell sarcoma	62	0.8%
Rhabdomyosarcoma	62	0.8%
Hemangiosarcoma	62	0.8%
Large cell carcinoma	57	0.7%
Malignant melanoma	52	0.6%
Paraganglioma	45	0.6%
Malignant fibrous histiocytoma	45	0.6%
Giant cell sarcoma	44	0.5%
Undifferentiated sarcoma	27	0.3%
Plasmacytoma extramedullary	24	0.3%
Oat cell carcinoma	23	0.3%
Follicular lymphoma grade 1/2	23	0.3%
B-cell chronic lymphocytic…	21	0.3%
lymphoma	20	0.2%

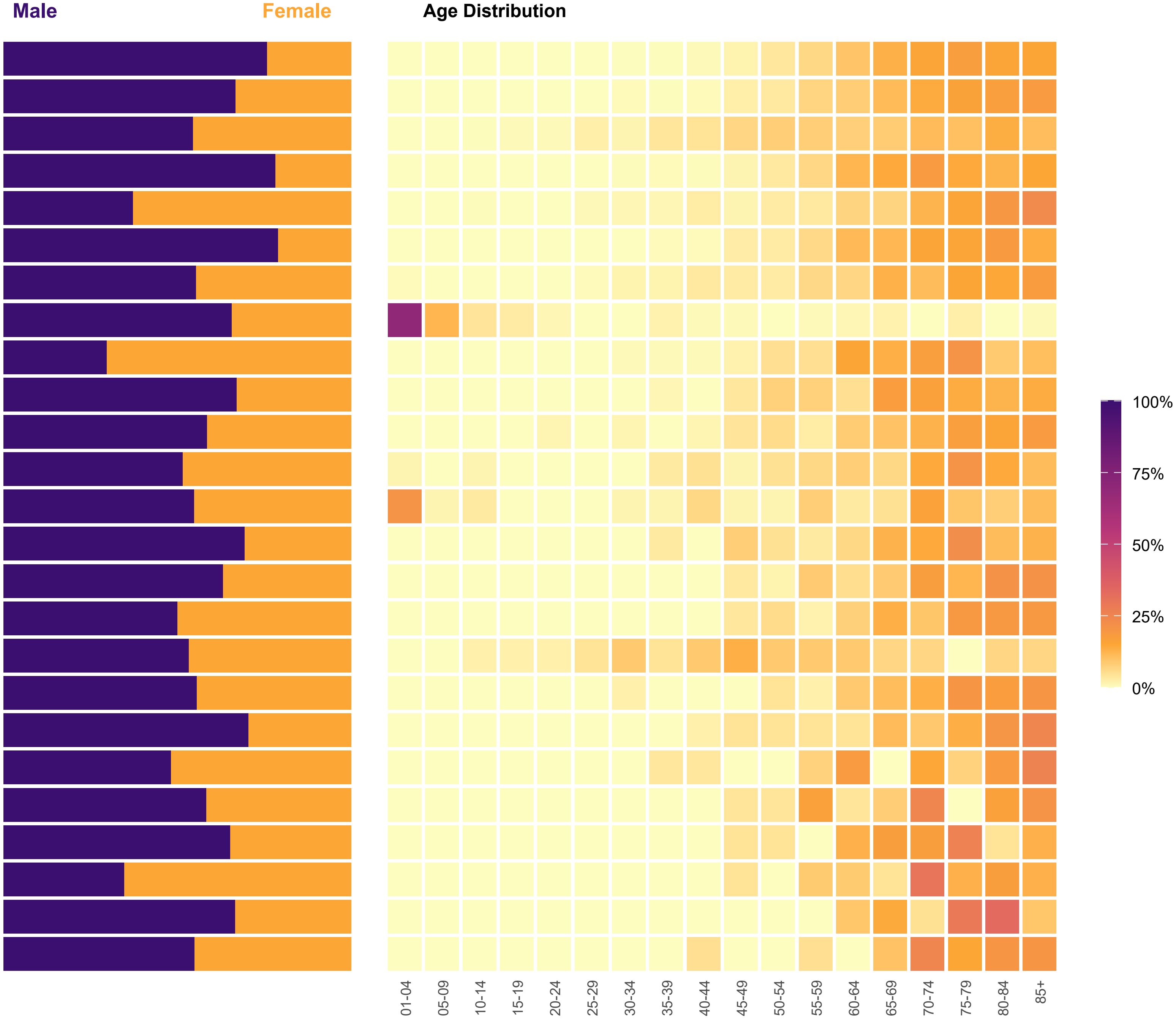

Primary Site: Urinary Other | Phenotype: epithelial

Top 7 Morphologies | cases: 11,012

Morphology	n	%
Transitional cell carcinoma	8,671	78.7%
Papillary transitional cell…	1,908	17.3%
Adenocarcinoma	150	1.4%
Squamous cell carcinoma	134	1.2%
Urothelial carcinoma sarcomatoid	32	0.3%
Clear cell adenocarcinoma	27	0.2%
Urothelial carcinoma micropapillary	22	0.2%

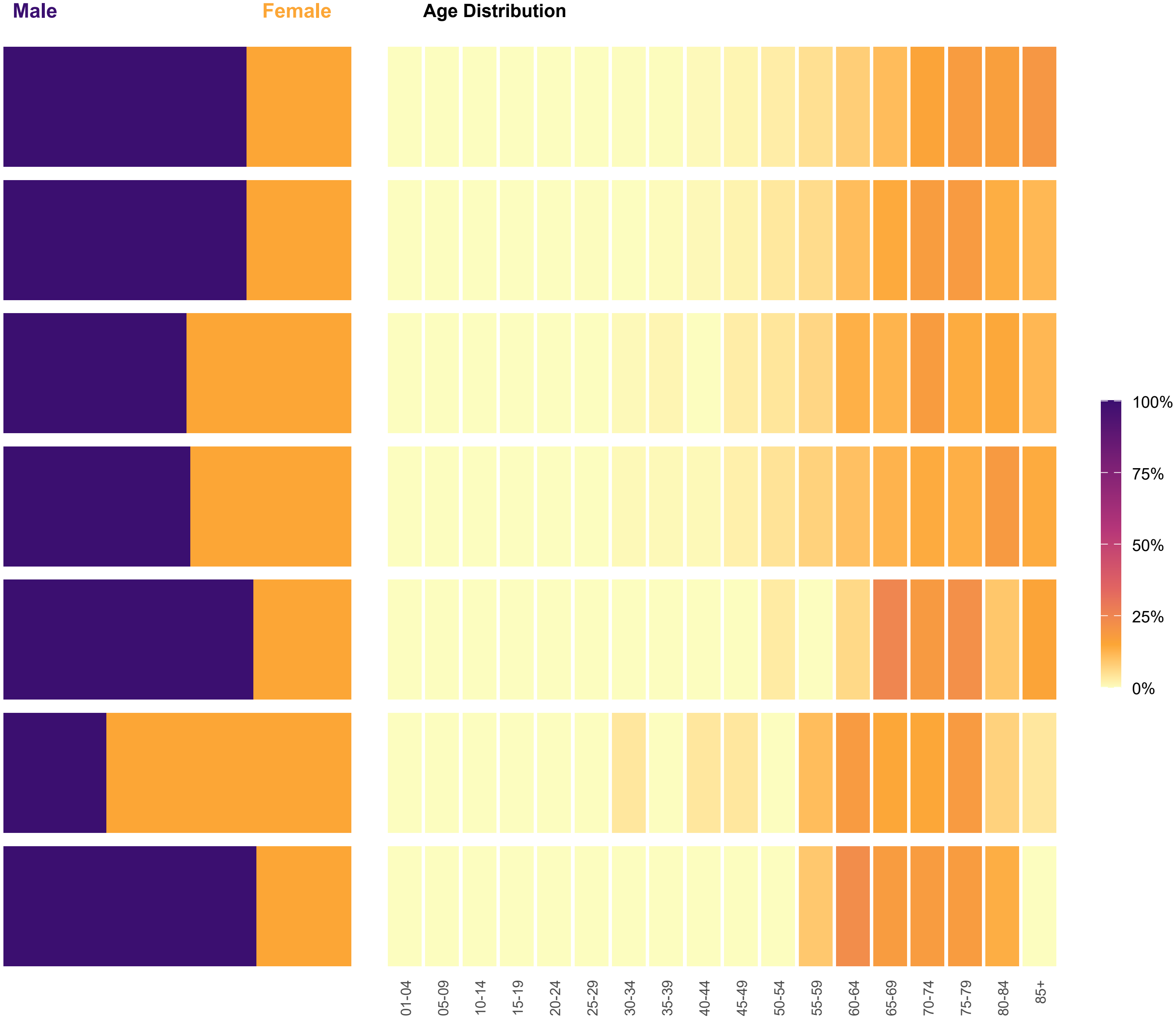

Primary Site: Urinary Other | Phenotype: Grouped Phenotypes

Top 1 Morphologies | cases: 34

Morphology	n	%
Small cell carcinoma	20	58.8%

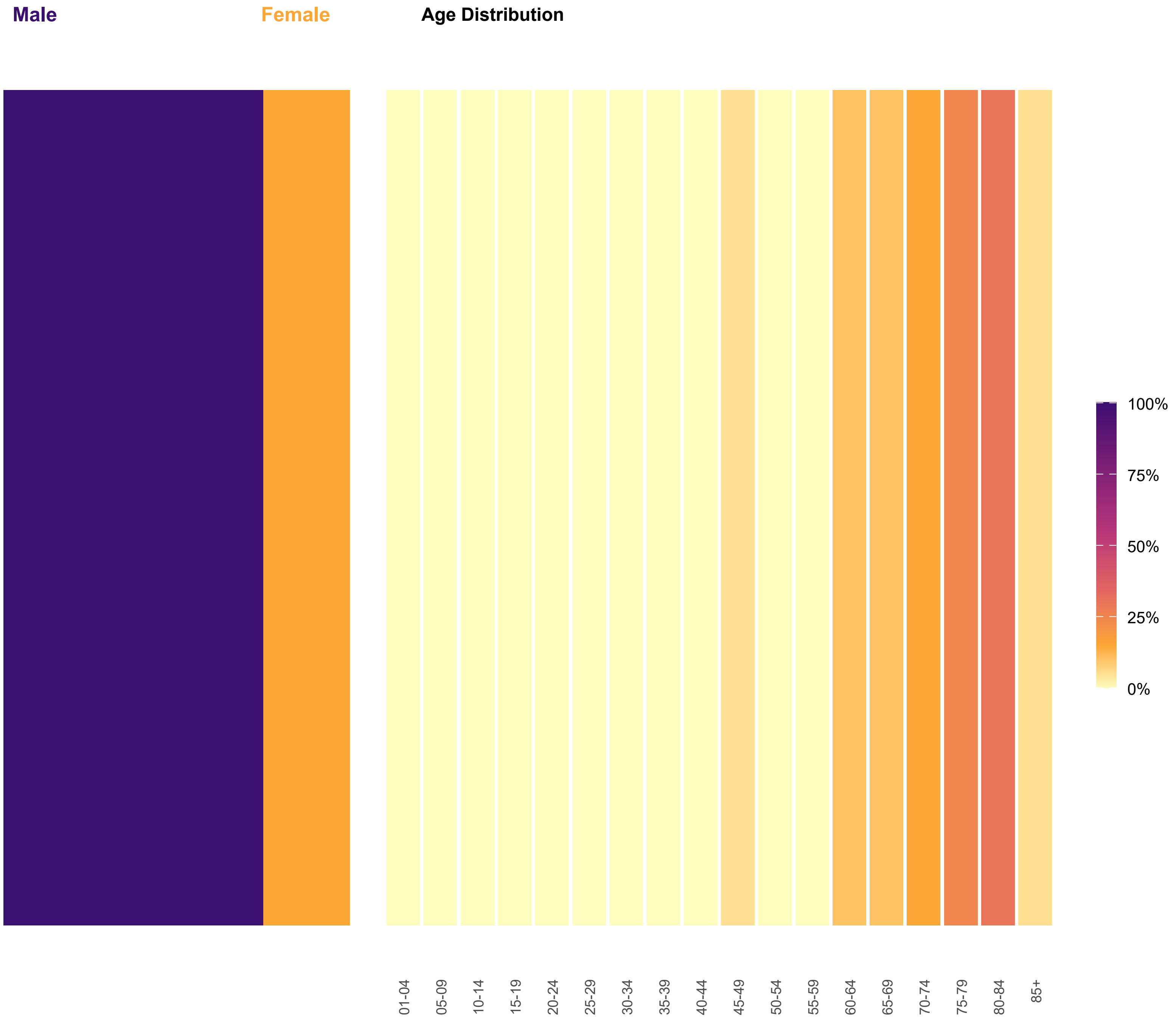

Primary Site: Vagina | Phenotype: epithelial

Top 15 Morphologies | cases: 14,129

Morphology	n	%
Squamous cell carcinoma	11,276	79.8%
Adenocarcinoma	1,286	9.1%
Endometrioid adenocarcinoma	400	2.8%
Clear cell adenocarcinoma	245	1.7%
Papillary squamous cell carcinoma	157	1.1%
Basaloid squamous cell carcinoma	150	1.1%
Mucinous adenocarcinoma	110	0.8%
Papillary adenocarcinoma	89	0.6%
Neuroendocrine carcinoma	66	0.5%
Serous carcinoma	54	0.4%
Mixed cell adenocarcinoma	42	0.3%
Low grade serous carcinoma	34	0.2%
Paget disease extramammary	24	0.2%
Mucin-producing adenocarcinoma	21	0.1%
Adenoid cystic carcinoma	20	0.1%

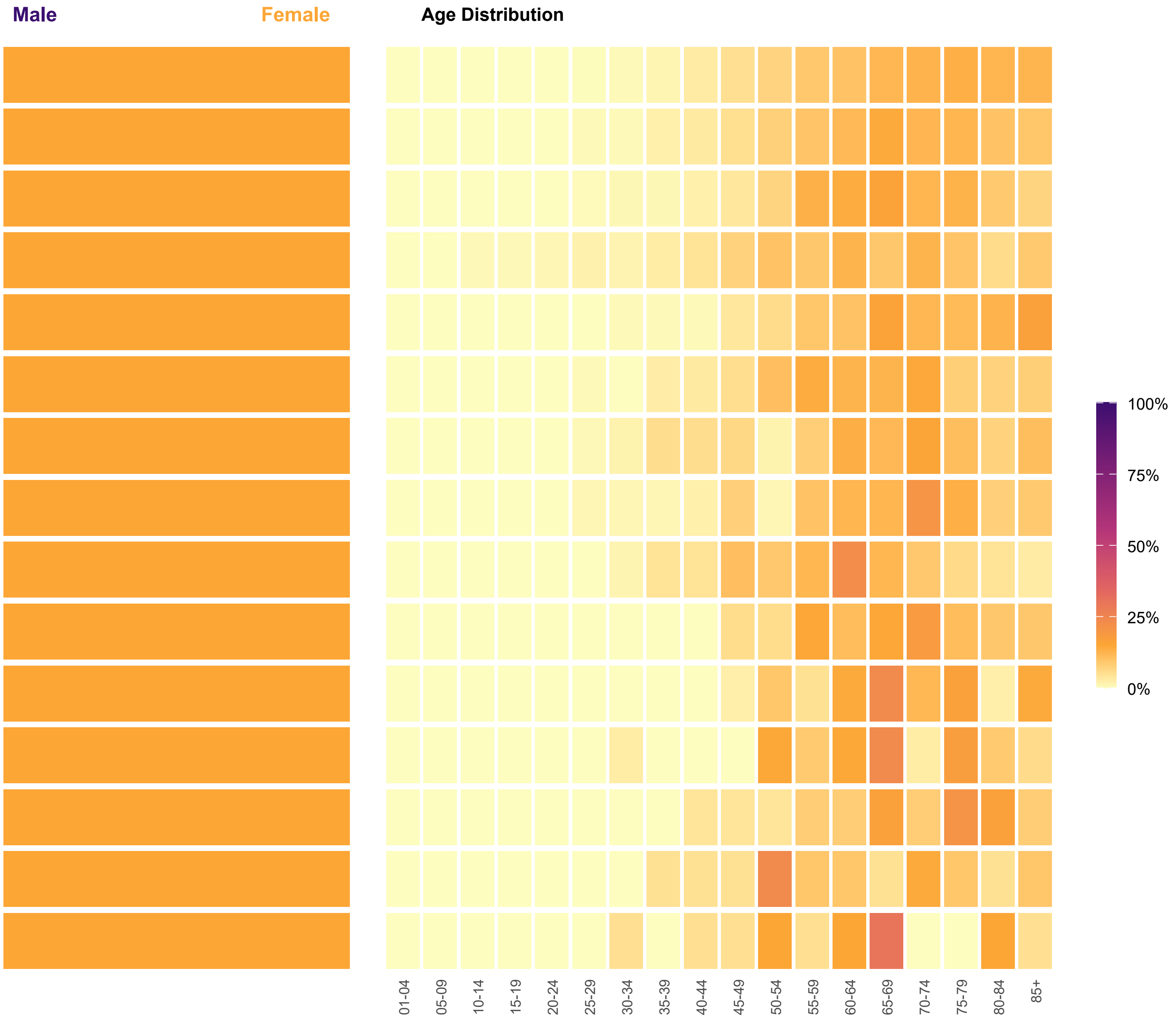

Primary Site: Vagina | Phenotype: Grouped Phenotypes

Top 11 Morphologies | cases: 1,964

Morphology	n	%
Malignant melanoma	744	37.9%
Leiomyosarcoma	205	10.4%
Small cell carcinoma	145	7.4%
Carcinosarcoma	123	6.3%
Diffuse large B-cell lymphoma	97	4.9%
Nodular melanoma	92	4.7%
Embryonal rhabdomyosarcoma	68	3.5%
Sarcoma	57	2.9%
Amelanotic melanoma	56	2.9%
Mullerian mixed tumor	41	2.1%
Spindle cell sarcoma	23	1.2%

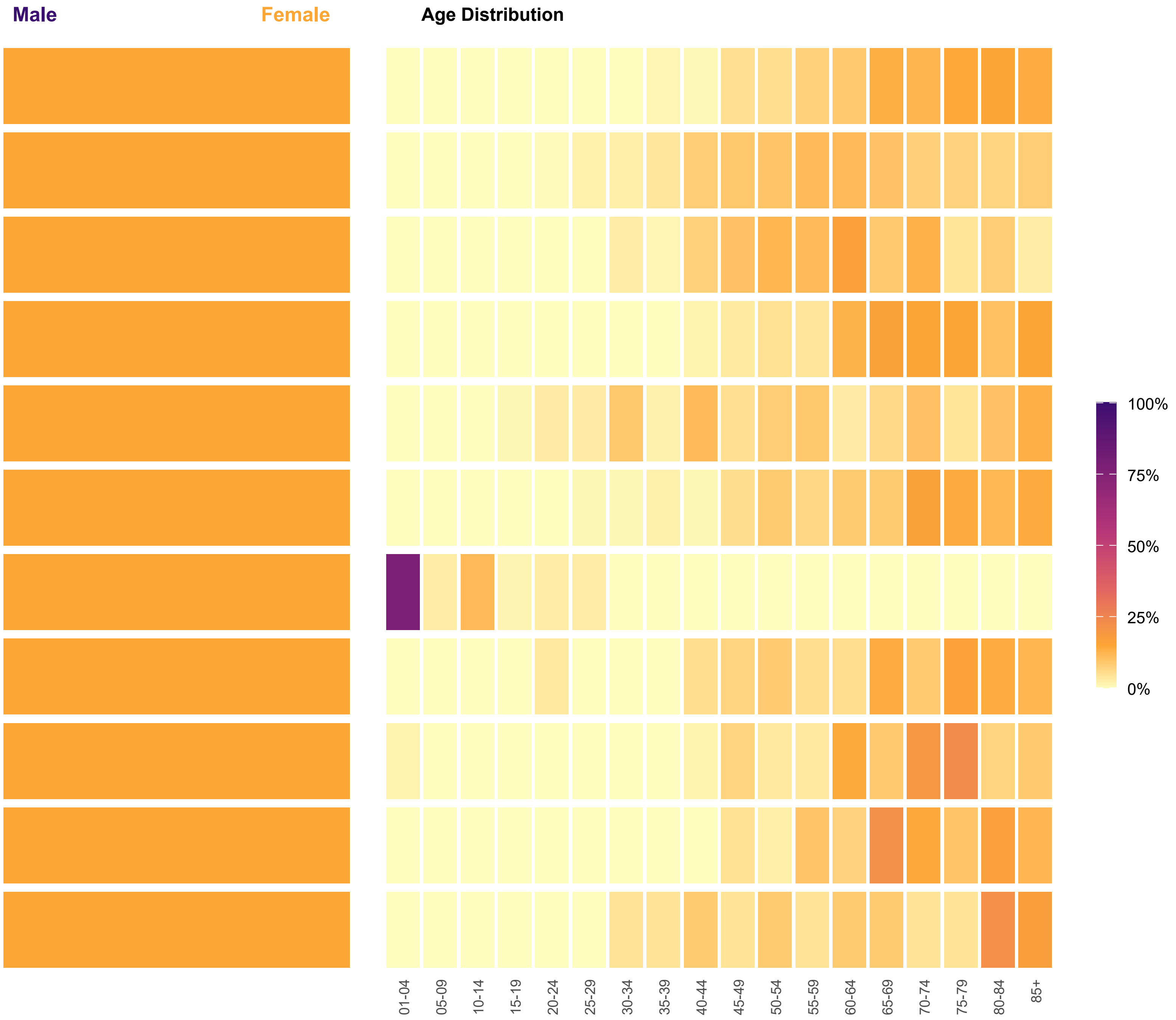

Primary Site: Vertebral column | Phenotype: Grouped Phenotypes

Top 15 Morphologies | cases: 5,435

Morphology	n	%
Plasmacytoma	2,634	48.5%
Diffuse large B-cell lymphoma	948	17.4%
Chordoma	813	15.0%
lymphoma non-Hodgkin	196	3.6%
Langerhans cell histiocytosis…	163	3.0%
Follicular lymphoma	111	2.0%
Follicular lymphoma grade 1/2	74	1.4%
lymphoma	56	1.0%
Plasma cell myeloma	46	0.8%
Marginal zone B-cell lymphoma	42	0.8%
Chondroid chordoma	33	0.6%
Burkitt lymphoma	27	0.5%
Anaplastic large cell lymphoma…	23	0.4%
Ependymoma	22	0.4%
Lymphoplasmacytic lymphoma	21	0.4%

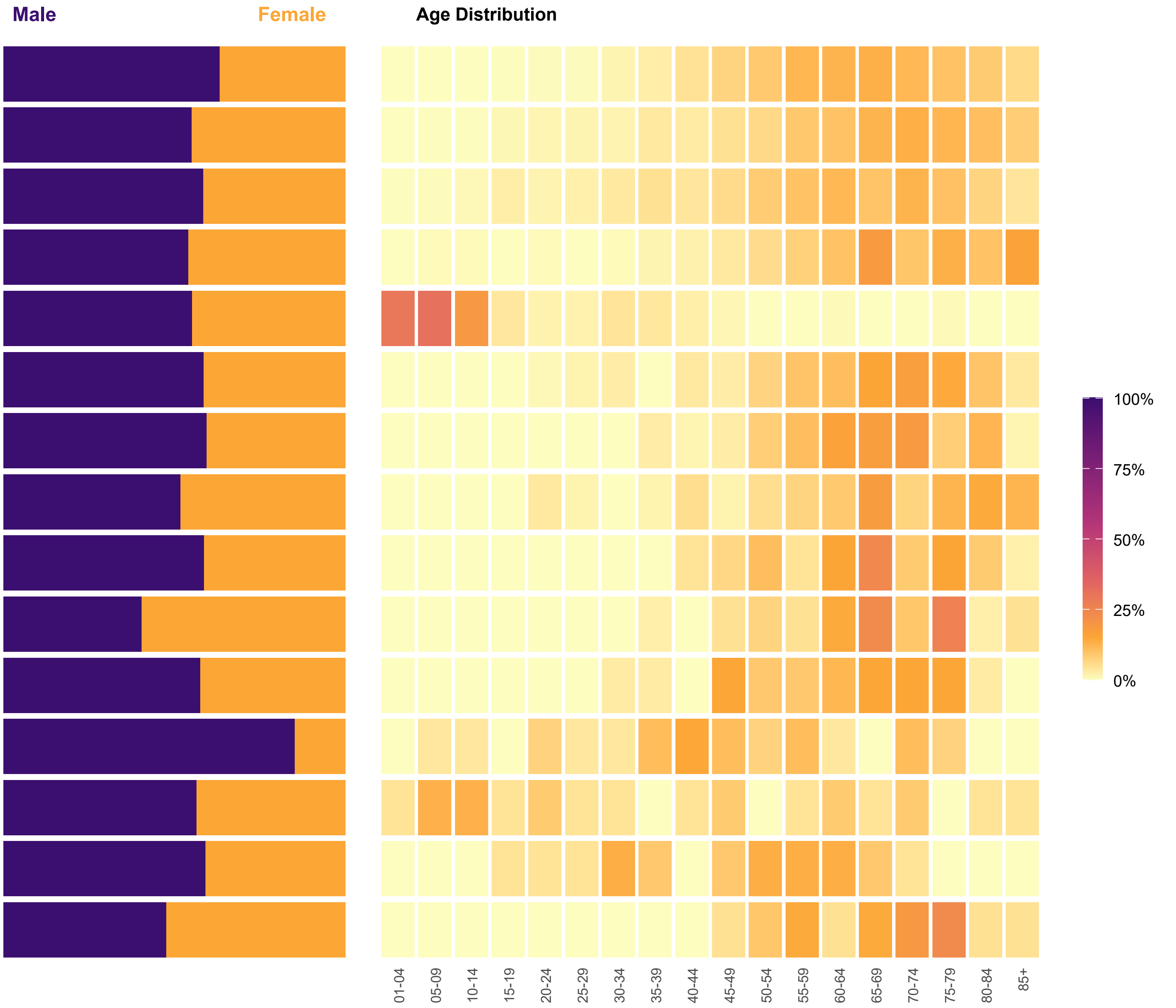

Primary Site: Vulva | Phenotype: epithelial

Top 15 Morphologies | cases: 72,543

Morphology	n	%
Squamous cell carcinoma	62,823	86.6%
Basal cell carcinoma	4,355	6.0%
Paget disease extramammary	2,896	4.0%
Basaloid squamous cell carcinoma	759	1.0%
Adenocarcinoma	661	0.9%
Basosquamous carcinoma	213	0.3%
Adenoid cystic carcinoma	171	0.2%
Mucinous adenocarcinoma	103	0.1%
Papillary squamous cell carcinoma	63	<0.1%
Basaloid carcinoma	63	<0.1%
Sebaceous carcinoma	50	<0.1%
Basal cell adenocarcinoma	36	<0.1%
Neuroendocrine carcinoma	33	<0.1%
Apocrine adenocarcinoma	30	<0.1%
Papillary adenocarcinoma	27	<0.1%

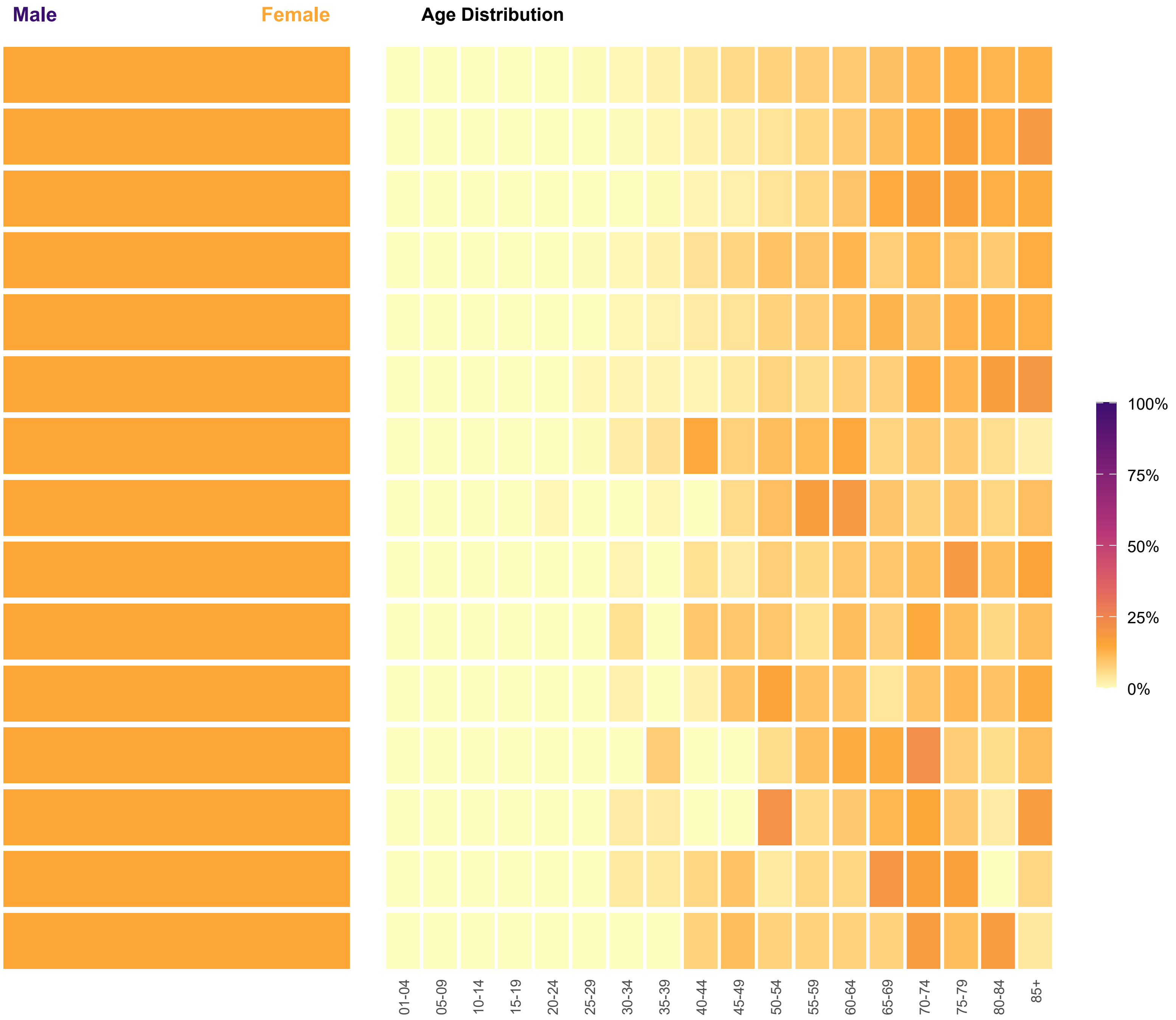

Primary Site: Vulva | Phenotype: Grouped Phenotypes

Top 17 Morphologies | cases: 4,074

Morphology	n	%
Malignant melanoma	1,694	41.6%
Nodular melanoma	610	15.0%
Low cumulative sun damage melanoma	575	14.1%
Dermatofibrosarcoma protuberans…	155	3.8%
Leiomyosarcoma	138	3.4%
Amelanotic melanoma	75	1.8%
Epithelioid sarcoma	63	1.5%
Mucosal lentiginous melanoma	61	1.5%
Sarcoma	61	1.5%
Spindle cell melanoma	53	1.3%
Diffuse large B-cell lymphoma	44	1.1%
Small cell carcinoma	42	1.0%
Spindle cell sarcoma	34	0.8%
Acral melanoma	32	0.8%
Lentigo maligna melanoma	28	0.7%
Carcinosarcoma	28	0.7%
Malignant fibrous histiocytoma	20	0.5%

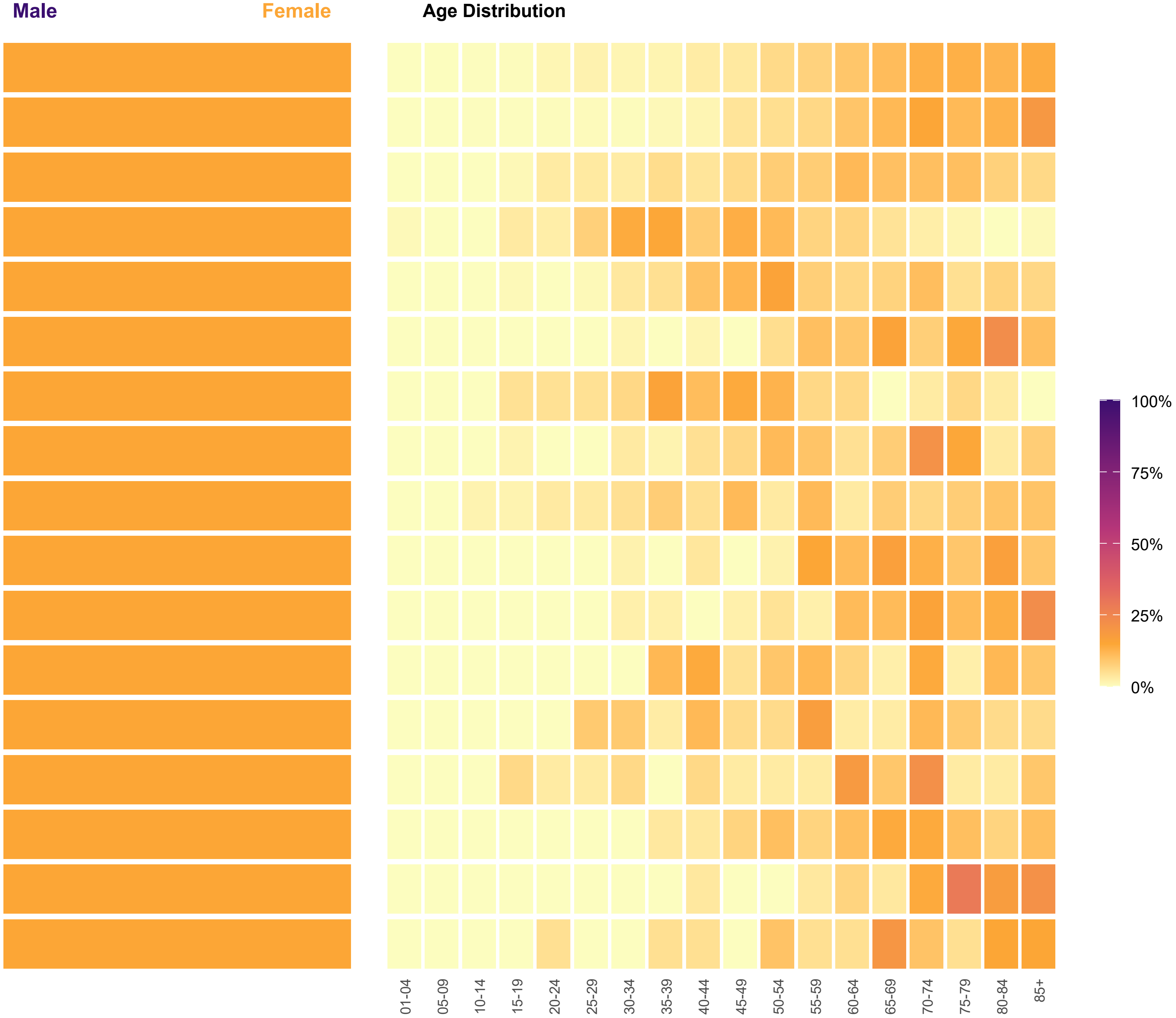

Supplement: Supplementary file 2 — ESM2: Bericht 2: Primärtumor- und Phänotypenanalyse (detaillierte Morphologiecodierung) [file 292_2026_1555_MOESM2_ESM.pdf]
